# Abstracts of the ICARE 2022 76th SIAARTI National Congress: Milan, Italy. 28-30 October 2022

**DOI:** 10.1186/s44158-022-00070-7

**Published:** 2022-10-28

## Anestesia ambulatoriale e NORA

### A1. Airway management in preterm newborns during brain MRI in lateral position: an observational study in a tertiary center

#### Maiellare F., Sbaraglia F., Della Sala F., Garra R., Spinazzola G., Spanò M.M., Tosi F., Rossi M.

##### Fondazione Policlinico Universitario A. Gemelli IRCCS - Dipartimento Scienze dell'emergenza, anestesiologiche e della rianimazione ~ Roma ~ Italia

###### **Correspondence:** Maiellare F

**Background** The diagnosis of anatomical and neurocognitive alterations in premature babies through brain magnetic resonance imaging (MRI) is becoming an essential examination. The long execution time and the need of stillness require patient sedation [1], which involves risks associated with maintaining a patent airway [2]. Thanks to a new coil for high resolution pediatric imaging (dStream Ped Neurospine, Philips) in our center, we introduced a new standard operating procedure of care able to perform MRI in lateral position. In this observational cohort study, we reported our preliminary experience considering airway-related adverse events and the number of repeated acquisitions for motion artefacts.

**Methods** We review the internal surveillance database and clinical charts of preterms undergone MRI from January 2022 until April 2022. We picked out all preterm infants born at 36 weeks Post Conceptional Age (PCA) or earlier undergoing deep sedation in lateral position for brain RMI. We excluded infants with chronic or acute respiratory diseases that required mechanical invasive ventilation. In presence of multi-parameter monitoring (EKG, SpO2, EtCO2) sedation was induced through an O2/N2O mixture (50%-50%) inhalation by a non-rebreathing mask. After few minutes, N2O was replaced by Sevoflurane (3-4%) in FiO2 0.4 to maintain anesthesia. The baby was then put in lateral position and transferred into the coil. Significant episodes of peripheral oxygen desaturation (SpO2 < 90%), of apnea (>10 sec), of bradycardia (HR < 25% of baseline) and the number of repeated acquisitions due to motion artefacts, were detected.

**Results** Twenty-three newborns (36+/-2 wks PCA and 3150 +/-268 g) were included; at birth the mean age was 31 +/-5 wks PCA and the mean weight 1875 +/-160 g. All infants were in spontaneous breathing, but two of them received high-flow nasal cannula oxygen therapy at 0.2 ml/kg. All patients were sedated according to the Internal protocol sedation for this class age. All exams were completed. No significant episode of oxygen desaturation occurred during procedures and in two case EtCO2 signal was temporarily altered, without a sign of apnea. Bradycardia was never reported. No acquisition was repeated for breathing or hemodynamic issues, but one infant required repositioning of the head during the procedure.

**Conclusions** Preterm infants with low bodyweight are at high risk for peri-procedural apnea. Several studies describe respiratory adverse events during deep sedation (Table 1), but our preliminary data suggests that lateral position is a feasible and safe option for a high-quality MRI.

**References**

1. Lei H, Chao L, Miao T, et al. Serious airway-related adverse events with sevoflurane anesthesia via facemask for magnetic resonance imaging in 7129 pediatric patients: A retrospective study. Pediatr Anesth. 2019;29:635–639

2. Walther-Larsen S, Rasmussen LS. The former preterm infant and risk of post-operative apnoea: recommendations for management. Acta Anaesthesiol Scand. 2006;50(7):888-893

3. Gomez F, Cabrera M, Sanabria P, Sanchez L, Lopez-Ortego P, Elorza MD. Sevoflurane for Short Painful Procedures in the Neonatal Intensive Care Unit. Am J Perinatol. 2019 Mar;36(4):377-382

4. Vinson AE, Peyton J, Kordun A, Staffa SJ, Cravero J. Trends in pediatric MRI sedation/anesthesia at a tertiary medical center over time. Paediatr Anaesth. 2021;31:953-961

**Table 1 (abstract A1).** Literature data about respiratory-related adverse events

	Gomez et al. [3]	Vinson et al. [4]	Our data
	n (%)	n (%)	n (%)
Peripheral desaturation	4 (10.3)	47 (12.56)	0
Respiratory apnea	3 (7.7)	No data	0
Bradycardia/hypotension	No data/8 (20.5)	10/13 (2.67/3.48)	0/no data

### A2

#### C. Stefano, M.F. Bianco, C. Biscardi, B. Calandra^1^, O.S. Cava, L. Faita, S. Filicetti, F. Gencarelli, G. Grimaldi, M.S.Ippolito, L. Mazziotti, A.Silvagni, M. Vigna, P.Pasqua

##### Azienda Ospedaliera Annunziata, UOC Intensive Care, Cosenza, Italy

**Background**

The advantages of regional anaesthesia over general anaesthesia in terms of safety, efficacy, and patient satisfaction are well known. Complications associated with regional blocks may be limited to the eye or may be systemic(1). We report a case of brainsteim anesthesia associated a subaracnoid hemorragie after retrobulbar anesthesia for intraocular surgery.

**Case report**

A 69-year-old woman patient with retinal detachment was scheduled for elective vitrectomy. The patient presented with a remote pathological history allergy to tramadol and pennicilins. A retrobulbar block of the right eye with 4 ml of levobupivacaine 7,5% using a 21 Gauge needle. Possible intravascular injection was excluded by aspiration during both procedures. After about 8–10 minutes of the block, she developed lost consciousness and important respiratory depression. Her blood pressure was 80/40 mmHg with pulse rate of 50. Manual ventilation with a face mask was instituted, due to the peristence of the clinical conditions, she was orotracheal intubated and she was urgently transferred in the emergence department. Arrived in the shock room the patient presented neurologically in Coma Glasgow Scale E (1) V (NT) M, bilateral babinski in the lower limbs, to the painful stimulus internal rotation and extension of the upper limbs, the eyes had fixed mydriasis. The patient underwent CT of the brain and MRI of the brain and she was admitted to Intensive Care. CT showed “...Linea hyperdensity in the right front-orbitary region...”, MRI showed “...in the right front-orbitary region presence of an alterated cortical signal area, hyperintense in flair with hypointensity in flair...”(Figure 1). In ICU she was subjected to sedation by continuous infusion of propofol, mannitol and dexamethasone were given intravena and connected to an artificial ventilator. She remained stable throughout the day. After 24 hours from admission to therapy, the administration of propofol was interrupted with consequent recovery of consciousness and spontaneous breathing. Therefore we proceeded to respiratory weanning and extubation of the same.

**Conclusion**

Systemic complications may arise from inadvertent injection or diffusion into orbital vasculature or injection into the subarachnoid space(2). The onset of signs and symptoms of brainstem anesthesia is usually within 10 minutes of the local anestethic injection. In our case, the clinical presentation of loss of consciousness along with hypotension, bradycardia and respiratory arrest was compatible with retrobulbar anesthesia due to puncture of dural sheath and spreading of the local anesthetic to the subarachnoid space. The spread of the local anesthetic is clearly visible as it is accompanied by blood suffusion clearly visible on the CT and MRI brain examination. Patients receiving retrobulbar anaesthesia should be carefully monitored at least 20 minutes after the block. Life support equipment should be available before performing retrobulbar block(2).

Informed consent to publish had been obtained**.**

**REFERENCE**

1. Ivan Kostadinov et al. Brainstem Anaesthesia after Retrobulbar Block. Open Med (Wars). 2019; 14: 287–291.

2. Bertini L et al. SIAARTI guidelines for safety in locoregional anaesthesia. Minerva Anestesiol. 2006 Sep;72(9):689-722.

**Fig. 1 (abstract A2).**
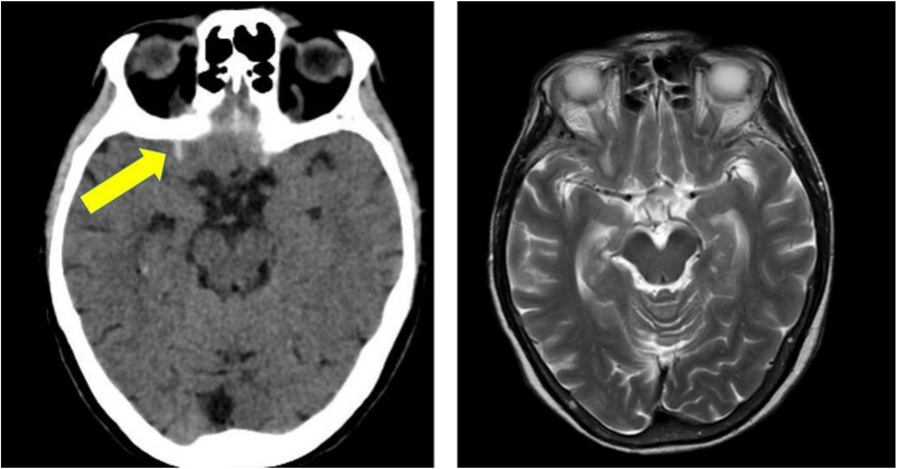
Tc and MRI of fireplace hemorragic imagine

### A3. Tailored sedation in patients undergoing hepatic and renal thermal ablation during covid 19 pandemia

#### Tosi P.F., Soriano Rodrigo M.C., Ferrari C., Roberta M.

##### IRCCS Humanitas Research Hospital-, via Manzoni 56, 20089 Rozzano, Milan Italy; Department of Anesthesia and Intensive Care Units Humanitas research Hospital. ~ Milano ~ Italia

###### **Correspondence:** Tosi P.F.

Background

Interventional angioradiology is facing great implementation, allowing percutaneous treatment of diseases, traditionally approached throughout conventional surgery, furthermore presenting comparable if not better outcome (1).

The patients approachable are many, involving more fragile and overall complex patients.

A more standardized and patient tailored anesthesiological management is of great importance. Highly skilled anesthesiologists and knowledge sharing, between all professionists involved, are mandatory (1).

COVID-19 pandemia further stressed the need for reconsider demanding patient pathways in order to limit hospital stay and minimize overall risks and failures.

Materials and Methods

Two dedicated senior anesthesiologists reviewed the patient paths in interventional radiology and introduced sedation protocols (2)(3) with Remifentanil (0,03-0,2 mcg/kg/min), Dexmedetomidine (bolus 0,1 mcg/Kg and infusion 0.2 -1,2 mcg/kg/h) and Propofol 1% (bolus only of 0,2 - 0,3 mg/kg on demand). The depth of sedation was always monitored with Bispectral Index (BIS), guided with target 60-80. Properly hydration (1-2 ml/kg/) to prevent CI- AKI (4) and normothermia (warm fluid infusion and forced air prewarming (5), were guaranteed.

Result and Discussion

During COVID- 19 pandemia, 65 patients (average age 64 yrs) underwent percutaneous thermal ablation for kidney (35 cases) and liver (30 cases) lesions.

Our new approach resulted in improved patient and staff satisfaction (assessed by specific questionnaire) and outcome (no major intraprocedural anesthesia complications and mishaps).

Conclusion

The dedicated anesthesia team and the tailored sedation achieved multiple advantages:

- Sedation with spontaneous breathing, avoiding invasive ventilation and aerosol dispersing maneuvers reducing operator and patient virus related risks in this COVID-19 pandemic era.
- More stable hemodynamics, intra and post procedure, with rapid recovery.
- Reduced anesthesia related complications.
- Higher patient collaboration with more accurate and faster procedures.

The anesthetic management in interventional radiology is evolving very quickly. According to radiologists we are moving a class of procedure and patients to ambulatory management with less ordinary admission and post-procedural monitoring requirements.

REFERENCES

1. Use of Anesthesiology Services in Radiology Hansol Kim, MDa,b, Jason Lane, MD, MPHc,*, Rolf Schlichter, MDd,Michael S. Stecker, MDa,b, Richard Taus, MDe. Adv Ther (2019)

2. The Effect of Dexmedetomidine on Postanesthesia Care Unit Discharge and Recovery: A Systematic Review and Meta-Analysis eremy Cheuk Kin Sin, MBBS,*† Alexis Tabah, MD,†‡ Matthys J. J. Campher, MB ChB,*§ Kevin B. Laupland, PhD,∥¶ and Victoria A. Eley, PhD†#. Anesthetic Clinical Pharmacology (2022)

3. Efficacy and Safety of Deep Sedation in Percutaneous Radiofrequency Ablation for Hepatocellular Carcinoma Koki Sato . Nobuhito Taniki . Ryo Kanazawa . Motonori Shimizu . Shigeto Ishii . Hideko Ohama . Masashi Takawa . Hiroaki Nagamatsu .Yasuharu Imai . Shuichiro Shiina. Springer Healthcare Ltd., part of Springer Nature 2019

4. Pathophysiology of Contrast-Induced Acute Kidney Injury (CIAKI) Georgios Vlachopanos 1 , Dimitrios Schizas 2 , Natasha Hasemaki 2 , Argyrios Georgalis 3 Curr Farm Des (2019)

5. Prewarming according to the AWMF S3 guidelines on preventing inadvertant perioperative hypothermia 2014 : Retrospective analysis of 7786 patients. R Grote 1, A J Wetz 2, A Bräuer 2, M Menzel Der Anesthesist 2018

### A4. Safety use of gastrotm-lma in nora setting for high-risk patients with difficult airways: case report

#### Di Bona R., Grasso A., Ciaravola M., Fiorito R., Frezza F., Ferraro F.

##### A.O.U. "Luigi Vanvitelli" - Dipartimento della Donna, del Bambino e di Chirurgia Generale e Specialistica ~ Napoli ~ Italy

###### **Correspondence:** Di Bona R.

**Introduction**

Percutaneous Endoscopic Gastrostomy (PEG) is a procedure performed on patients with deglutition dysfunction who depend on long-term enteral nutrition and/or administration of drugs. Gastro TM-LMA (Laryngeal Mask Airway) is a supraglottic dual-channel device, specifically developed for endoscopy and upper gastrointestinal tract interventions. Represents an alternative to endotracheal intubation with the aim of avoiding adverse outcomes linked to sedation in endoscopic procedures. On one hand, there is evidence of superior efficacy of the Gastro TM LMA compared to endotracheal intubation, as it is associated with lower complications related to the device, on the other hand, it appears less evaluated in the context of high-risk patients with Difficult Airways in NORA (Non-Operating Room Anaesthesia) setting.

**Case Presentation**

Is reported a case of an 83-years old patient, 17.6 BMI, admitted to the Geriatrics Department for aspiration bronchopneumonia. Previous clinical history shows senile dementia, Parkinson's Disease, type 2 diabetes mellitus, permanent atrial fibrillation, thyroid goiter with right-sided nodule (6 cm), chronic immobilization syndrome, and previous COVID-19 infection. Cardiologic evaluation highlighted a moderate right heart dysfunction. A thoracic CT scan highlighted a bilateral pleural effusion with contiguous lung parenchymal atelectasis and bilateral consolidation areas. The patient was unconscious, obtunded and positive Kussmaul sign. Multiple Difficult Airways criteria could be observed: trismus, nuchal rigidity (head blocked in flexion), forced position of the head on the neck (towards left) associated with right sternocleidomastoid muscle hypertrophy, lateral tracheal deviation, mandibular hypoplasia. The patient came to our attention for PEG positioning. In the NORA setting, pre-medication was given with Midazolam 1 mg + Atropine 0.5 mg, induction with Propofol 40 mg, and later positioning of GastroTM-LMA n.3. The patient was connected to the ventilator machine in PSV (PS 14 and PEEP 2 cmH20) and kept on spontaneous breathing for the whole duration of the procedure. The maintenance of the sedation was with bolus of Propofol (80 mg in total). PEG preparation, prior local anaesthesia with 2.5 ml Lidocaine 2%, was completed in about 20 minutes (including bioptic retrievals) with complete recovery from sedation in approximately 15 minutes and subsequent transfer of the patient to ICU for observation and discharged after about 12 hours.

**Discussion**

Inadequate sedation and upper airway irritation, which may arise during gastrointestinal endoscopy, often cause cough and laryngospasm, leading to hypoxemia and potential cardiac arrest. If it is correct that the occurrence of adverse events is greater for high-risk patients, however it is also true that complications might have fatal outcomes in Difficult Airways cases. GastroTm-LMA allows the separation of the operative channel of the airways, which are kept protected, and permits to avoid the use of general anesthesia.

**Conclusions**

In our case, GastroTM-LMA proved to be safe to the PEG positioning, in the NORA setting, in a high-risk patient (ASA 4) and Difficult Airways, without the necessity of general anesthesia. Ventilation was optimal and without intraoperative and postoperative adverse effects. The same experience was judged excellently by the endoscopy operators as well.

Informed consent to publish had been obtained. If consent had not be obtained then the abstract should be removed from the supplement.

### A5. Report of the experience pass (care pathway for person with special needs) in Livorno hospital

#### Nemo F.^1^, Vegnuti L.^1^, Ferro B.^1^, Pillitteri M.^2^, Roncucci P.^1^

##### ^1^azienda usl toscana nordovest ~ Livorno ~ Italy, ^[2]^azienda ospdaliera universitaria pisana ~ pisa ~ Italy

###### **Correspondence:** Pillitteri M.

Background

People with special needs require personalized care paths to perform hospital procedures and to minimize the stress for them and their family. (1)

The Tuscany’s healthcare system creates a dedicated care path for children or adults with disabilities. These patients usually require deep sedation to perform even simple physical exams and procedures. The process of the sedation can be particularly difficult, due to the fact it needs to be performed in non operating room anesthesia areas (NORA) (2) without usual systems of monitoring and the control of venous access.

In this report we describe the experience of patients treated following the PASS project at Livorno Hospital. Methods

We enrolled 12 patients with diagnosis of autistic spectrum disorder of median age of 15 years (range 5-32 years) between May 2021 - May 2022. The procedures included medical visits (laboratory exams, echocardiography, dermatologic and dental consults, radiological investigations, vaccines’ administration). The care path started with a phone call with patients’ relatives to give strict instructions.

After admission in a confortable room of the clinic (fig 1) the patient received an oral sedative (midazolam 0,8mg/kg) mixed with the favourite food or beverage. After 10 min intramuscular ketamine (3 mg/kg) was added in 5 patients to obtain a Rass of -2. A venous access let to continue the sedation with TCI guided infusion of propofol with usual monitoring.

In two cases the patients were transferred under sedation in the operating room for procedures of dental extraction under general anesthesia.

No respiratory or cardiovascular peri-procedural complications were reported, despite the minimal monitoring during sedation.

After the procedures patients were discharged with RASS=0. Only patients who underwent general anesthesia were discharged after a period of 24 hours. (3-4) A questionary to explore satisfaction of relatives was administered.

In the totality of the cases (100%) satisfaction was appreciated in consideration of the attention of the path to the care of patients’ special needs and the reduction of global stress.

Conclusion

Our report illustrates the efficacy and the safety of a personalized and multidisciplinary care path organized for the care of people with special needs. The success of this path is dependent by the presence of empathic and dedicated personnel who can adapt their profession to the special needs. Furthermore adding intramuscular low dose ketamine to oral medication appear safe even in the absence of operatory room’s monitoring. (5)

Bibliography

1) World Bank and World Health Organization. The World Report on Disability. 2011 [cited201813/04]; Available from:http://www.who.int/disabilities/world_report/2011/en/

2) SIAARTI-SARNePI Linee Guida sulla sedazione in neuroradiologia pediatrica - Minerva Anestesiologica 2004 October;70(10):675-715 - Minerva Medica

3) Flavia Petrini, Antonino Giarratano, Paolo Giaccone et altri BPC SIAARTI-SIED sull'Analgo-sedazione in endoscopia digestiva.'11 gennaio 2022

4) Julia Jockusch 1, Bernhard A J Sobotta 2, Ina Nitschke 3 2 Outpatient dental care for people with disabilities under general anaesthesia in Switzerland BMC Oral Health 2020 Aug 18;20(1):225.

5) G Dolansky, BA (Hon),1 A Shah, MD,2 G Mosdossy, MD,2 and MJ Rieder, MD PhD1,2,3 What is the evidence for the safety and efficacy of using ketamine in children? Paediatr Child Health. 2008 Apr; 13(4): 307–308.

**Fig. 1 (abstract A5).**
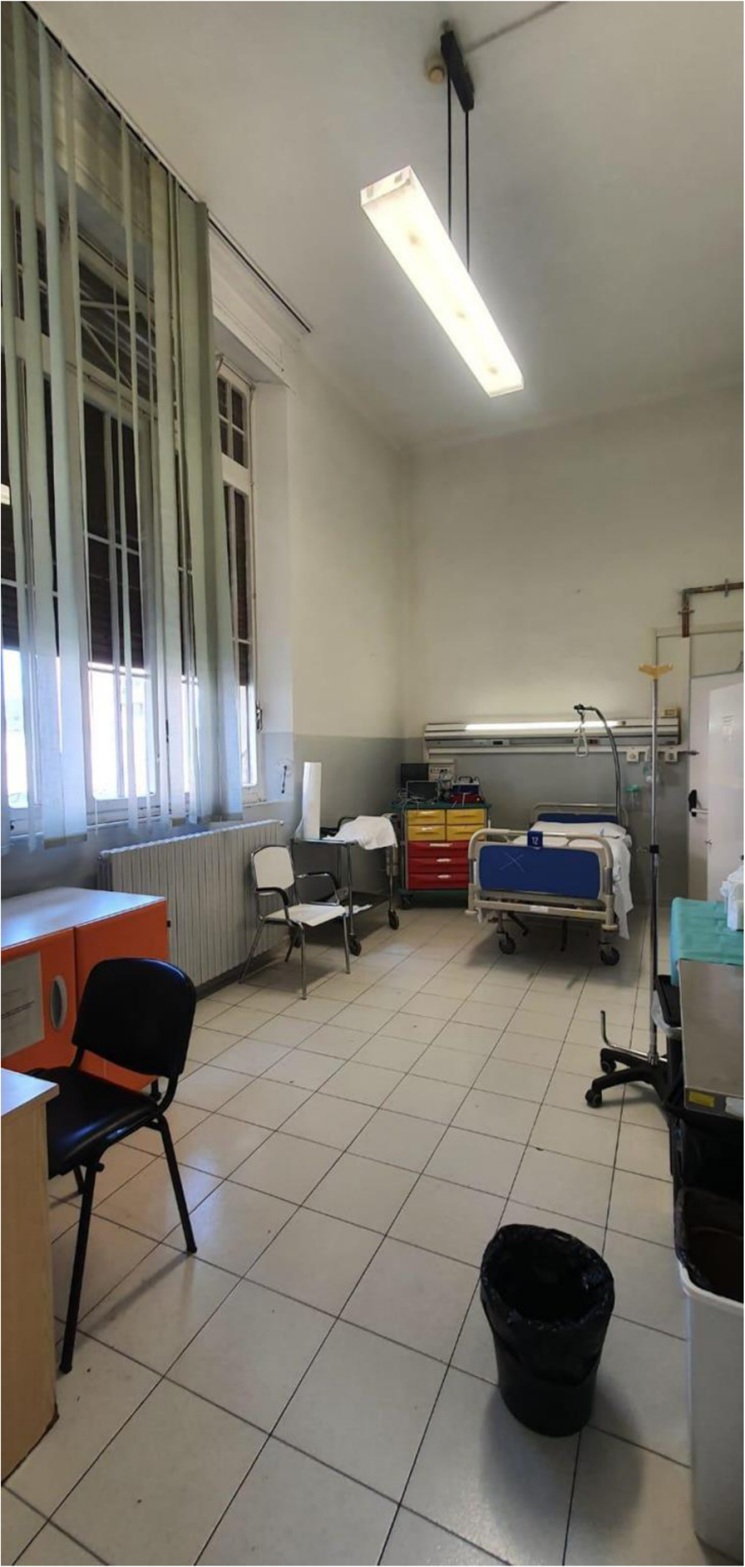
See text for description

### A6. Dexmedetomidine in paediatric brain MRI patients

#### Granata M.L., Saglimbene S.T., Messina F., De Luca S., Nicocia C., Magro G., Montalto T., Grasso I., Rapisarda G.

##### Department of Anesthesia and Resuscitation, S. Marta and S. Venera Hospital ~ Acireale ~ Italy

###### **Correspondence:** Granata M.L.

Introduction

The safety level of conscious sedation, an anesthesiologic approach inducing muscle relaxation, amnesia and control during a diagnostic procedure, depends on good physician’s experience in the previous clinical evaluation of patients and during anesthesiologic procedure. Aim of this study is to evaluate the efficacy and safety of dexmedetomidine in procedural sedation to perform brain MRI with and without contrast in a paediatric cohort of patients.

Materials and methods

We included 128 patients (75 males and 53 females) from Child Neuropsychiatric Centre with a mean age at onset of 4.7 years (range 2-6 years) and a body weight between 10 and 32 Kg. 8 patients (5 males and 3 females) were excluded because of bradycardia. 3 mcg/kg of intranasal dexmedetomidine were administered by atomizer to the 120 children 35-40 minutes before the procedure. All patients included were treated with 5 mg of sublingual midazolam 5 minutes before the administration of dexmedetomidine. The patients were monitored by optical-fibre based pulse oximeter without venous access.

Results

We induced sedation in all patients. The mean of sedation onset was of 37 minutes. The time of awakening after verbal stimulus was of 20 minutes. In addition, we had to administer intravenous midazolam to 7 patients (3 males and 4 females). 113/120 patients maintained spontaneous breathing and their reflexes were elicited. The awakening was longer in 18/113 patients (approximately 7 minutes). 3/113 needed intravenous atropine because of bradycardia during procedure. No long-term adverse events were reported in our cohort.

Conclusions

In our paediatric cohort, dexmedetomidine resulted effective and safe when used for sedation during brain MRI. Dexmedetomidine is a promising alternative to others anesthesiologic tecniques for use in this procedural sedation.

### A7. A modified approach for ultrasound-guided thoracic paravertebral block via thoracic intervertebral foramen in an adolescent patient: a case Report J Clin Med

#### Petrucci E.^1^, Pizzi B.^2^, Bianchi C.^3^, Marrocco G.^3^, Ceccaroni G.^3^, Marinangeli F.^3^

##### ^1^Department of Anesthesia and Intensive Care Unit, San Salvatore Academic Hospital of L’Aquila ~ L'Aquila ~ Italy, ^2^2Department of Anesthesia and Intensive Care Unit, SS Filippo and Nicola Academic Hospital of Avezzano ~ L’Aquila ~ Italy, ^3^Department of Life, Health and Environmental Sciences ~ L'Aquila ~ Italy

###### **Correspondence:** Bianchi C.

**Background**

Epidural analgesia and paravertebral block are the gold standard for anesthesia and thoracic analgesia, although other procedures have recently been introduced.

**Case report**

This clinical case shows a new approach to ultrasound-guided thoracic paravertebral block, through the injection of local anesthetic into the thoracic intervertebral foramen. The anesthesiological procedure was performed in the Red Room of the Emergency Department of the Hospital SS Filippo and Nicola di Avezzano in a 25-year-old male (weight: 78 kg, height: 1.78, BMI: 33.05) road accident victim. A total body CT showed: fractures of the V, VI right and IV, V, and VI left ribs, plurifocal pulmonary contusions, ruptured spleen capsule with hemoperitoneum, and minimal frontoparietal right acute subdural hematoma. During the back inspection phase, the block of the thoracic intervertebral foramen was performed at the T9 level. A linear ultrasound probe was positioned transversely to the spiny process (SP) of T9, identifying the angle between this and the transverse process (TP). A Tuohy needle (18 gauge, 90mm) was inserted into the fascial plane of the spine erector muscle with *in-plane* approach in lateral-medial direction along the SP until it reached the angle with the TP. The tip of the needle was moved in the caudo-cranial direction until it lost contact with the bone. The needle then was advanced by 2 mm and 6 ml of 0.5% levobupivacaine were injected here. An infusion catheter was inserted for about 1 cm from the tip of the needle and a continuous infusion of 0.25% levobupivacaine at the rate of 2.5-4 ml/h was started. The procedure was performed bilaterally. There were no complications related to the execution of the block. The patient was then transferred to the operating room to perform the surgery of splenectomy. General anesthesia was induced with intubation and connection to the mechanical ventilatory prosthesis. No additional opioids were used during the surgery and the patient was hemodynamically stable. After the surgical phase the patient was transferred to the ICU where after 24 hours the respiratory weaning was obtained. During the stay in ICU, there was no need for additional opioids or vasopressory support. The patient reported pain control during respiratory (NRS: 4) and at rest (NRS: 2). The catheters were removed 72 hours after surgery. A postoperative CT, documented the presence of a bilateral hypodense area in the paravertebral thoracic space from T6 to T12, suggesting the spread of local anesthetic.

**Conclusion**

This report describes a new approach to provide anesthesia and analgesia of the thoracic and abdominal region, reducing the risks related to the execution of the paravertebral block by the classical route (pneumothorax, hematoma) because the needle is always run along a bone plane. This procedure is rapid, with the possibility of reducing the use of opiates, for an early weaning of the patient and fewer complications related to mechanical ventilation. Anatomical studies on cadavers and future clinical trials, will allow to document the spread of local anesthetic in the paravertebral space and any clinical benefits.

**Consent to publish**

Informed consent to publish had been obtained.

### A8. The use of ozased® as anxiolitic in children undergoing MRI

#### Pizzo C., Garra R., Festa R., Spanò M.M., Federica T., Rossi M.

##### Fondazione A. Gemelli/ Cattolica ~ Roma ~ Italy

###### **Correspondence:** Pizzo C.

BACKGROUND

Anxiety is recognized as an independent predictive index of the appearance of behavioural alterations in children after general anaesthesia. While delirium spontaneously disappears in few minutes after emergence from anaesthesia, symptoms such as nightmares, separation anxiety, anger may also occur after some time. Non-pharmacological interventions are recognized to produce varying degrees of anxiolysis. However, the difficulty of coping with the different etiological factors of anxiety, make it necessary the use of anxiolytic drugs. Midazolam has a long experience and it is still the most common oral sedative in children being more accepted than other routes of administration, such as intravenous, intramuscular or intranasal. Ozalin® - approved by AIFA under the name of Ozased® - is a new oral formulation developed to overcome the bitter taste and the inconsistent bioavailability of the parenteral form, when administered in off-label mode. The pharmacokinetic studies carried out on the paediatric population have assessed its efficacy, safety and acceptability, both as premedication or analgo-sedations. In this cohort, retrospective study, the anxiolytic and sedative efficacy of Ozased was evaluated in paediatric patients undergoing MRI at the A. Gemelli Foundation.

MATERIALS AND METHODS

40 children 56.4% male and 43.6% female (1-10aa) were enrolled to undergo anesthesia for MRI over a period of 4 months. Exclusion criteria were: drug intolerance, ASA >3,benzodiazepine therapy, acute respiratory tract infectious, psychiatric disorders.

The primary endpoints were considered the degree of MF acceptance, according to the Mask Acceptance Scale, and the MAC of Sevoflurane needed to obtain the LOC.

Delirium at the emergence from anesthesia, analysed with the PAEDS, and the appearance of behavioral alterations according to PHBQ-AS survey, were the secondary endpoints.

The acceptance of the drug through the SAF, the Ramsey Sedation Score, and Parental Separation Anxiety Scale were also misured.

Anesthesia was induced with N2O/O2 and Sevorane increased in concentration until LOC, then maintained with O2 via MF until the end of the procedure.

RESULTS

SAF scored 4 was in 42.2% of patients; PAEDS at 15 minutes, using 10 as a cut off, was 28% at discharge. A nonparametric correlation between age and PAEDS variables was recorded, with significance for PAEDS at 15 min (p= 0.039), and a significant difference between age as a continuous variable and mask acceptance (p= 0.017).

CONCLUSIONS

Ozased® has proven to be handy, safe, effective and appreciated drug by the majority of patients. However, it is important to predict the medium-long onset time to avoid delayed turnover in high-flow patient settings such as MRI.

BIBLIOGRAPHY

Kain ZN,et al. Distress during the induction of anesthesia and postoperative behavioral outcomes. AnesthAnalg 1999 May;88(5):1042-7.

-Davies FC, et al. Oral midazolam for conscious sedation of children during minor procedures.

Emergency Medicine Journal 1998;15:244-248.

-Malinovsky JM, et al. Premedication with midazolam in children. Effect of intranasal, rectal and oral routes on plasma midazolam concentrations. Anaesthesia. 1995 Apr;50(4):351-4.

-Lyseng-Williamson K.A.: Midazolam oral solution (Ozalin®) Drugs & Therapy Perspectives (2019) 35:255-262.

- Marçon F. et al. European Journal of Pharmaceutical Sciences 114 (2018) 46–54.

## Anestesia degli animali

### A9. Intranasal atomization of ketamine, medetomidine and butorphanol for the induction on anesthesia in rabbits

#### Serpieri M., Bonaffini G., Quaranta G., Prandi I., Barbero F., Mauthe Von Degerfeld M.

##### Dipartimento di Scienze Veterinarie - Università di Torino ~ Grugliasco (TO) ~ Italia

###### **Correspondence:** Serpieri M.

Background

Rabbits can be easily stressed during restraint for administration of drugs, leading to possible stress-related metabolic changes and musculoskeletal trauma.

A non-invasive method of drug delivery, the intranasal administration with MAD Nasal™ (Mucosal Atomization Device) has shown positive results in human Medicine, especially in the pediatric field [1], and in some animal species [2]. Also, transnasal administration has been evaluated in rabbits [3].

The aim of this study was the evaluation of intranasal atomization for the induction of anesthesia in rabbits using MAD Nasal™.

Materials and methods

26 mixed-breed domestic rabbits, undergoing various types of surgery, received a combination of ketamine (20 mg/kg), medetomidine (0.4 mg/kg) and butorphanol (0.2 mg/kg), administered intranasally using MAD Nasal™ (Group A: 13 rabbits) or intramuscularly (Group B:13 rabbits).

Induction time, times of loss and reappearance of pedal and palpebral reflexes along with time of head lifting were recorded. Where required, isoflurane was dispensed through a face mask, with manual assisted ventilation in case of superficial breathing or SpO2<95%. At the end of the procedures, atipamezole (1-2 mg/kg) was administered through the same routes in the respective groups.

Results

Statistical analyses were performed using one-way Anova test and chi-squared test (Program R, version 4.1.2), considering a significance level of p<0.05. There were no statistically significant differences for the followings: induction time (p=0.096), times of loss and reappearance of palpebral (p=0.11; p=0.06 respectively) and pedal (p=0.08; p=0.37) reflexes, need of assisted ventilation (higher in Group B) (p=0.39) and its duration (p=0.29); duration of surgery (p=0.4) and anesthesia (p=0.32).

There were statistically significant differences for the need of supplemental isoflurane (higher in Group A) (p=0.02) and time of head lifting (p=0.02), occurring earlier in Group A (mean: 4 vs 8 minutes after the administration of atipamezole) suggesting a faster onset of the effects of atipamezole when administered by atomization in unconscious patients.

Conclusion

The need of isoflurane and a more efficient spontaneous breathing in Group A suggest a lighter plane of anesthesia compared to Group B, potentially related to a partial loss of the drugs due to a lack of a gold standard in the administration technique. Moreover, part of the dose could have been lost during swallowing or sneezing in some rabbits.

Overall, our data showed no differences between the routes of administration in the induction times. Intranasal atomization was therefore a good alternative for administering sedative and anesthetic drugs, less invasive than traditional methods.

References

1. Qian B, Zheng W, Shi J, Chen Z, Guo Y, Yao Y. Ketamine Enhances Intranasal Dexmedetomidine-Induced Sedation in Children: A Randomized, Double-Blind Trial. Drug Des Devel Ther. 2020; 14: 3559–3565.

2. Hess L, Votava M, Malek J, Kurzova A, Sliva J. Sedative Effects of Intranasal Oxytocin in Rabbits and Rhesus Monkeys. Physiol Res. 2016; 65: 473-480.

3. Santangelo B, Micieli F, Mozzillo T, Reynaud F, Marino f, Auletta l, Vesce G. Transnasal administration of a combination of dexmedetomidine, midazolam and butorphanol produces deep sedation in New Zealand White rabbits. Vet Anaesth Analg. 2016; 43: 209-214.

### A10. Impact of a dexmedetomidine intravenous infusion in septic dogs: preliminary study

#### Di Franco C., Batisti E., Miragliotta V., Coli A., Millanta F., Briganti A.

##### Dipartimento di Scienze Veterinarie, Università di Pisa ~ Pisa ~ Italia

###### **Correspondence:** Di Franco C.

Background: Sepsis represents an increasing health emergency both in human and veterinary medicine Dexmedetomidine is an alpha-2 agonist drug with sedative and analgesic properties recently used critically ill patient in human ICU [1]. The purpose of this work was to evaluate hemodynamics effects of a continuous infusion of dexmedetomidine in septic dogs.

Materials and methods: We enrolled 16 septic dogs arrived at the Intensive Care Unit (ICU) of the Veterinary Teaching Hospital “Mario Modenato” of the University of Pisa that underwent emergency surgery in a prospective blind randomized clinical trial (auth. n.24/2020). A clinical evaluation and blood tests (haematological and biochemical, blood gas analysis) were performed at arrival and 24, 48 and 72 hours after surgery. A qSOFA and SOFA score were calculated for all dogs upon arrival at ICU and every day during hospitalization. Patient progress, possible discharge and the 28-day survival rate were also recorded. Dogs were randomly divided into two groups: dexmedetomidine (DEX) group that received a constant rate infusion of dexmedetomidine at 1mcg/kg/h and the control group (NaCl) that received an equivalent infusion volume of NaCl. All patients were premedicated with fentanyl 5mcg / kg IV, induced with propofol and maintained with sevoflurane. The infusion (DEX or NaCl) started 10 minutes before induction of anaesthesia. Analgesia was provided by a variable fentanyl infusion (3-20 mcg/kg /h). In case of hypovolemia, Ringer lactate 10 mL/kg boluses were administered in order to restore normovolaemia; lactate, blood pressure and heart rate were monitored for the response. In case of hypotension due to vasodilation (MAP <60 mmHg), norepinephrine was administered starting from 0.05 mcg/kg/min with an increase of 0.05 mcg/kg/min until normotension was restored. In case of failure to restore normotension within 30 minutes of the initial infusion, adrenaline boluses were considered. The anaesthetist in charge of the case was not aware of the protocol administered. During the surgery, heart rate (HR), blood pressure, capillary refill time (CRT), arrhythmias, EtCO2, ET’Sevo and spirometry were recorded every 5 minutes.

At the end of the anaesthesia, the infusion of dexmedetomidine or NaCl continued in the intensive care unit (ICU) for a total of 24 hours.

Results: The number of deceased patients was statistically lower (p = 0.0034) in the DEX group (1/10) in comparison to the NaCl group (5/6). The number of patients that required norepinephrine administration was not statistically different between the two groups (p = 0.056) but the NaClgroup received a significantly higher dose (0.78 ± 0.25) than the DEX group (0.16 ± 0.26 ) (p = 0.007). Adrenaline necessity was significantly higher (p = 0.0004) in the NaCl group.

Conclusions and clinical relevance: From this preliminary results emerged that an infusion of dexmedetomidine at a dosage of 1 mcg/kg/h can contribute to increase cardiovascular stability, decrease the demand for intraoperative vasopressors and the need for emergency drugs such as adrenaline. Further studies with a larger number of cases are needed in order to confirm these results.

## Anestesia generale e medicina perioperatoria

### A11. The preliminary data of a prospective analysis about onset of postoperative acute kidney injury (AKI) after elective and urgent surgery

#### Toso F., Guzzetti L., Novazzi C., Selmo G., Carollo M., Binda S., Lanza C., Rossini G., Bacuzzi A.

##### Ospedale Di Circolo ASST SETTELAGHI S.C. Anestesia e Gestione dei blocchi Operatori ~ Varese ~ Italia

###### **Correspondence:** Toso F.

Background

AKI is a common complication after surgery and is associated with an increased risk of morbidity, mortality and development of chronic renal dysfunction. The aim of this prospective study is to determine the incidence of postoperative AKI after intermediate and high non-cardiac surgery, to recognize clinical impact of this problem in our University Hospital. to evaluate the 30-day mortality and a worsening of the quality of life through the "Duke Activity Status Index" (DASI).

Materials and Methods

The study included patients undergoing elective and urgent intermediate and high non-cardiac surgery procedures between June- October 2019 at University Hospital Ospedale di Circolo ASST-Settelaghi Varese. We define postoperative AKI according to KDIGO criteria. Our primary endpoint is the incidence of postoperative AKI onset. Additionally, we have analysed the AKI stage, the principal risk factors, 30-day mortality and the quality of life modification during immediate postoperative period according to the DASI (Duke Activity Status Index) .

Results

We enrol 210 patients. **Table 1** shows demographical and clinical characteristics of the patients. 77 patients (36%) develop AKI postoperatively: 49% grade I, 38% grade II, 13% grade III. The median time of AKI onset is between first and second postoperative day. Among the risk factors age, diabetes mellitus, preoperative diuretics, preoperative creatinine value , intraoperative hypotension and its duration were statistically significant. The worsening of quality of life, investigated with DASI, was statistically significant in AKI I/II group. None patients died during analysis period

Conclusions

Our study demonstrates an higher incidence of AKI at our hospital despite literature description. Moreover, male sex, hypertension, COPD, type of surgery and its duration were not statistically significant in our risk factors analysis. AKI negatively affects postoperative period determining a worsening of the quality of life after 30 days. Our analysis has several limitations such as cohort heterogeneity, small population and the short-term follow-up. This is a first step to approach this problem and it is necessary to implement perioperative protocol that predicts and treats this frequent complication.

**Table 1 (abstract A11).** Demographical and clinical characteristics of the patients

N° Patients	No AKI group (n=133)	AKI group (n=77)	p=
**Age, (years)**	69,5[61-76(28-89)]	72[64-81(18-89)]	0,0514
**Male Gender, n°**	85	55	0,2905
**BMI (Kg/m2)**	25,9574[22,7245-28,5648(17,4449-40,8163)]	25,2935[23,03-28,3937(12,4179-40,8588)]	0,8347
**Diabetes, n**	13	15	0,0186
**Hypertension,n°**	73	49	0,2467
**Aspirin, n°**	39	22	1,000
**Diuretics, n°**	10	15	0,0054
**Creatinine mg/dL**	0,97[0,86-1,17(0,51-1,93)]	1,12[0,88-1,41(0,58-8,6)]	0,0104
**COPD n°**	12	9	0,6341
**Smoker n°**	69	43	0,6670
**Cancer n°**	106	56	0,3062

### A12. Rocuronium 150mg and still tof counts 4/4 - ratio 100%: case report and discussion

#### Muraccini M.^1^, Manassero A.^2^, Meineri M.^2^, Coletta G.^2^

##### ^1^Università di Torino, Scuola di Specializzazione in Anestesia, Rianimazione, terapia intensiva e del dolore, Dipartimento di Scienze Chirurgiche ~ Torino ~ Italia, ^2^Ospedale Santa Croce e Carle ~ Cuneo ~ Italia

###### **Correspondence:** Muraccini M.

Introduction

We report the case of a patient who showed a complete resistance to 150mg of rocuronium. Years before he was anesthetized several times and rocuronium was always used successfully. We investigated potential pharmacological interactions, but we didn’t find a solid explanation for the complete absence of effect of rocuronium during this case.

Case report

A 79-year-old man was scheduled incisional hernia repair. He was in therapy with atenolol, amlodipine, metformin, insulin, alopurinol, citalopram and dutasteride.

His previous surgeries were pancreaticoduodenectomy, explorative laparotomy for bleeding, ERCP, and cholecystectomy. In those cases, rocuronium was used with the expected effect.

In the OR, general anaesthesia was induced with remifentanil 0.2mcg/kg/min, propofol 150mg and rocuronium 50mg but TOF was still 4/4 – 100% after 5 minutes. Endotracheal intubation was performed with the patient gagging, and the surgery started in these conditions. Desflurane was used for anaesthesia maintenance.

After 10 minutes with TOF always 4/4 -100%, an additional dose of rocuronium (50mg) from a different batch was given, but the patient didn’t show any response.

After 10 more minutes with TOF always 4/4 -100%, a third dose of 50mg rocuronium was given, but nothing changed.

Finally, 8mg of cisatracurium were administered, and the neuromuscular blockade was achieved.

Discussion

We report the first case of totally unexplained complete resistance to triple dose of rocuronium.

First, we excluded improper storage conditions, and we changed three batches for the three boluses.

Then we checked the functioning of the venous catheter and we put another one, but nothing changed.

Pharmacokinetic changes like an increase of volume distribution, protein binding or clearance can alter the response to NDMBA, usually prolonging the drug’s effect and the recovery time [1].

Because of pharmacokinetic mechanisms, the elderly population is usually more sensitive to NMBAs, in opposition to what happened in our patient.

At the same way, lots of pharmacodynamic mechanisms can interfere with the normal physiology of the neuromuscular junction. Denervation injuries, immobilization, use of anticonvulsants and infections are reported to cause resistance to NDMBA [1], but none of these seems to fit with the history of our patient.

Cases of resistance to steroidal neuromuscular blocking agents due to glucocorticoids exists, which usually manifests as an attenuation of the block, and just in one case manifested as a complete absence of the block[2]. Our patient did not receive any corticosteroid therapy.

A systematic review published in the EJA [3] investigated the resistance to NDMBA, and focused on prolonged onset time as the main result of a resistance, without even mentioning complete absence of drug’s effect, demonstrating that it is a unique event, reported only in two cases [2].

Informed consent to publish had been obtained

Bibliography

[1] K. T. Jung *et al* «Updated review of resistance to neuromuscular blocking agents», *Anesth. Pain Med.*, vol. 13, n. 2, pagg. 122–127, apr. 2018, doi: 10.17085/apm.2018.13.2.122.

[2] A. Capuano *et al.*, «Complete resistance after maximal dose of rocuronium», *J. Pharmacol. Pharmacother.*, vol. 6, n. 3, pag. 175, 2015, doi: 10.4103/0976-500X.162012

[3] E. L. Mørk *et al* «Resistance towards nondepolarising muscle relaxants: prolonged onset time A systematic review», *Eur. J. Anaesthesiol.*, vol. 36, n. 7, pagg. 477–485, lug. 2019, doi: 10.1097/EJA.0000000000000991

Informed consent to publish had been obtained

### A13. Opiod-sparing during skin allograft in severely burned patients: comparison of propofol and ketamine combination (ketofol), and desflurane and fentanyl

#### Fiume D.^1^, Peverini M.^1^, Arciuolo M.^1^, Comis G.^2^, Carlini S.^2^, Galletti M.^1^

##### ^1^Department of Anaesthesia and Intensive Care Unit, Sant'Eugenio Hospital ~ Rome ~ Italia, ^2^Department of Anaesthesia and Intensive Care Unit, University of Tor Vergata ~ Rome ~ Italia

###### **Correspondence:** Fiume D.

Background

Severe burn wound is a very painful clinical condition, and requires a large-dose of opiods. Sometimes side effects can occur, even serious ones. Alternative pharmacological strategies can help clinical management during severe-burned patients caring. Propofol and Ketamine combination (Ketofol) is widely used, especially in the pediatric population [1], or in respiratory compromised patients too [2], with opioid-sparing for procedural sedation [3].

Materials and Methods

In our one year experience in Operating Room Burn Center (May 2021/April 2022), twenty patients were observed, treated with skin allograft for severe burn wounds (TBSA>20%). Ten patients were administered Propofol and Ketamine combination 1:1 (Ketofol) 1mg/kg and 1mg/kg for induction, and then 0.3 mg/kg/h (Group A); ten patients received Desflurane 0.8 MAC and Fentanyl 0.003mg/kg, then 0.3-1.8 mg with 0.7mg median (Group B). Surgical procedures was longer than 180 minutes, side effects were noted.

Results

Gender distribution was 12 males and 8 females, with median age 53.1 (21-81). Mortality rate was 65%. Numeric Rating Scale (NRS) and Visual Analog Scale (VAS) were kept below 3. In Ketofol group (Group A) only one patient had liver enzyme increased over the upper limit of normal: alanine transaminase, aspartate transaminase and gamma-glutamyl transferase. In Group B two patients had costipation, and one post-operative nausea and vomiting.

Conclusions

In our experience we noted a lower incidence of side effects in Ketofol group. Propofol and Ketamine combination can be an additional strategy in the severely burn patients care. Further studies are needed.

References

1 Foo TY, Mohd Noor N, Yazid MB, Fauzi MH, Abdull Wahab SF, Ahmad MZ. Ketamine-propofol (Ketofol) for procedural sedation and analgesia in children: a systematic review and meta-analysis. BMC Emergency Medicine 2020; 20(1):81

2 Nazemroaya B, Majedi MA, Shetabi H, Salmani S. Comparison of Propofol and Ketamine combination (Ketofol) and Propofol and Fentanyl combination (Fenofol) on quality of sedation and analgesia in the lumpectomy: a randomized clinical trial. Advanced Biomedical Research 2018; 7:134

3 Green SM, Andolfatto G, Krauss BS. Ketofol for procedural sedation revisited: Pro and Con. Annals of Emergency Medicine 2015; 65(5):489-91

### A14. Postoperative pulmonary complications (PPC) in demolitive head and neck surgery (HNS) and reconstruction with free flaps: results of a multicentric retrospective, observational 5 -year period study

#### La Rosa E.^1^, Cappellini I.^2^, Gallo O.^3^, Sarno A.^4^, Adembri C.^1^

##### ^1^Department of Health Sciences, Section of Anesthesiology and Critical Care, Careggi University Hospital ~ Florence ~ Italia, ^2^Department of Critical Care Section of Anesthesiology and Intensive Care, Santo Stefano Hospital ~ Prato ~ Italia, ^3^Department of Otorhinolaryngology, Careggi University Hospital ~ Firenze ~ Italia, ^4^Department of Otorhinolaryngology, Santo Stefano Hospital ~ Prato ~ Italia

###### **Correspondence:** La Rosa E.

Background

Demolitive head and neck surgery (HNS) and reconstruction with free flaps has a high rate of complications, with Pulmonary Postoperative Complications (PPC) mostly represented, from atelectasis to desaturation and pneumonia. Several variables have been described to affect their occurrence, but there is still no definitive consensus on them. This study aimed to measure the occurrence of general and PP complications and to evaluate whether there are predictive risk factors.

Material and Methods

The study was observational and retrospective, including patients diagnosed with HNC undergoing demolitive and reconstructive surgery with free flaps between 2016 and 2021. Data were collected from electronic clinical reports of Careggi Hospital, Firenze and Santo Stefano Hospital, Prato. Perioperative complications occurring during hospital stay were investigated; PPC were defined as: low arterial saturation values (SpO2< 90% with spontaneous breathing); the need for ventilatory support; pneumonia, documented on imaging or with microbiological findings. Each patient was retrospectively rated with two risk-scores. The Adult Comorbidity Evaluation Index (ACE-27) [1] which investigates several systemic comorbidities (e.g. cardiovascular, respiratory, renal) scoring from 0 to 3; the Clavien Dindo Classification [2] refers, instead, to the severity of postoperative complications, from grade 1 to 5.

Results

A total of 132 patients met the inclusion criteria, with a median age of 68 years (58-74), mostly men (67%) and severe comorbidities (ACE-27 > 2) in about 60%; the oral cavity was the most frequent cancer site (87,5%), with a consistent smoking exposure (63%). Between free flaps, fibula has shown a higher prevalence, followed by radial and anterolateral tight. The median duration of surgery was 380 minutes [320-460]. PPC occurred in 26% of patients, without sex difference (29 vs 21%, p=0.41). Several factors were associated to the occurrence of PPC: COPD (p<0.001) hypercreatininemia (p=0.004), hyperglycemia (p<0.001), age (p=0.0026), smoking exposure (p=0.026), sex (p=0.05) and previous Rx therapy (p=0.02). After the exclusion of confounding variables, the association of ACE-27 score > 2 and PPC was explored in a multivariate analysis, confirming that it was the only statistically associated with the risk of developing PPC (RR 2,6922 (1,088-6,656), p=0.032). In the analysis considering the CD, statistical significance was only found with tumor site (p=0,0411).

Conclusions

Results have shown that PPC occur in 26% of patients and that ACE-27 score is the only risk factor associated with their development; CD is exclusively related to tumor site.

References

1. Piccirillo, J. F. and A. R. Feinstein . "Clinical symptoms and comorbidity: significance for the prognostic classification of cancer." Cancer (1996) 77(5): 834-842.

2. Dindo, D., N. Demartines and P. A. Clavien "Classification of surgical complications: a new proposal with evaluation in a cohort of 6336 patients and results of a survey." Ann Surg (2004) 240(2): 205-213.

### A15. Peridia-score: an update on 2018 statistical analysis

#### Andresciani L.^1^, Galdini F.^1^, Cariddi C.^1^, Andresciani C.^2^, De Summa S.^3^, Calabrò C.^3^, Carravetta G.^3^, Mastrandrea G.^3^

##### ^1^Università degli studi di BARI ~ Bari ~ Italia, ^2^Università degli Studi La Sapienza ~ Roma ~ Italia, ^3^IRCCS Istituto Tumori Giovanni Paolo II - BARI ~ Bari ~ Italia

###### **Correspondence:** Andresciani L.

Background

PERIDIA-SCORE is a new tool developed by a multidisciplinary group of healthcare stakeholders in order to increase safety and to select best medical strategies for cancer patients who frequently need advanced and demolitive surgical techniques and post operatory intensive care hospitalization.

This score was built starting from a selection of clinical parameters most frequently used to assess peri-operative period in daily clinical practice with the aim of obtain a new single one which could guide a tailored therapeutic path through the whole pre, intra and post operative phases (figure 1).

Primary data acquired from the first phase of the PERIDIA01 study, a retrospective-perspective and still ongoing study, confirmed the cut-off of 16/48 overall points complications’ onset [1] and highlighted a good sensitivity and specificity for the whole score.

Materials and methods

A cohort of 83 patients who underwent peridiaphragmatic thoraco-abdominal surgery (e.g., esophagectomy, duodenocephalopancreatectomy, gastrectomy or lung resection) during 2018 in the surgical ward of the Cancer Institute Giovanni Paolo II in Bari was extrapolated from the PERIDIA01 dataset.

First of all, a ROC-analysis was performed to assess the predictability of the comprehensive PERIDIA-score and the partials (PERIDIA pre, intra and post), then, the same analysis was used to evaluate single items’ significance. Finally, a multivariate logistic regression was also implemented considering the results of the single one in order to assess which items are most significant.

Results

The ROC-analysis showed overall PERIDIA-score’s predictivity (AUC 0.8688, sensitivity 0.86, specificity 0.75) which is also higher than the single PERIDIA-pre (AUC 0,679, sensitivity 0.86, specificity 0.55), PERIDIA-intra (0,679, sensitivity 0.81, specificity 0.65) and PERIDIA-post (AUC 0.7266, sensitivity 0.47, specificity 0.87).

The multivariate logistic regression underlined three parameters with high correlation to complications' incidence: Nutritional Status for the PERIDIA-pre (OR 0.24, MR 0.08-0.71, p-value 0.01), FCIS for the PERIDIA-intra (OR 0.29, MR 0.10-0.85, p-value 0.03) and Breathing-code for the PERIDIA-post (OR 0.11, MR 0.03-0.32, p-value <0.001).

Conclusions

These results show PERIDIA-SCORE’s predictability and then its role as an easy tool in clinical practice for the peri-operative evaluation.

Moreover, another strong point is the PERIDIA-INTRA section, due to both its higher predictivity compared to those in the other two phases and the lack in literature of similar scores, that make this kind of score a useful innovation. In addition to this, it is important to highlight that this score was developed through the analysis of several observational studies. According to the multivariate logistic regression, the most significant variables are the frailty and nutritional status in the pre-operative period while the breathing evaluation in the post-operative one.

Acknowledgements

Next steps of this study are increasing the sample size and starting the perspective phase to understand the score’s effectiveness in the clinical practice.

References

1 Andresciani L, Calabrò C, Mastrandrea G et all. A new score to assess the perioperative period of the cancer patient undergoing non-palliative elective surgery: a retrospective evaluation of a case report by PERIDIA score. Front oncol. 2021 Oct 11;11:733621.

**Fig. 1 (abstract A15).**
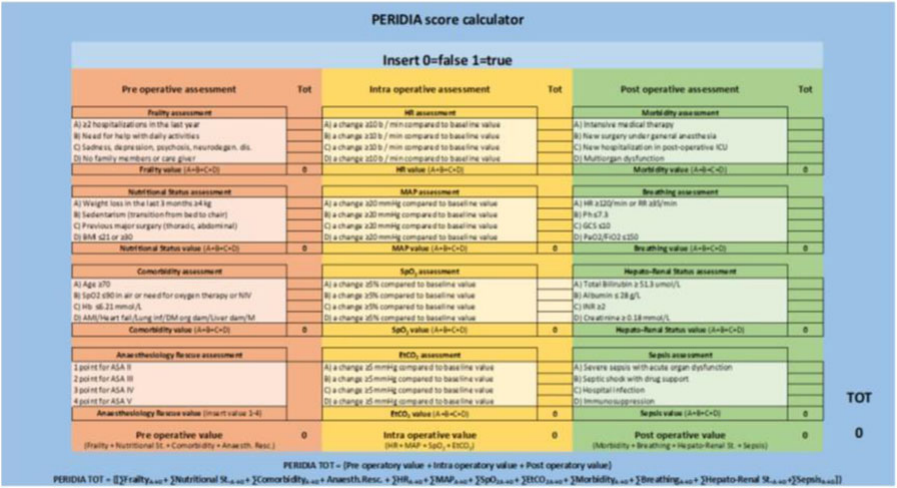
The PERIDIA-calculator for the overall score

### A16. The hemodynamic effects of etilefrine: original contributions and potential applications in a case series

#### Baggio F.^1^, Guzzetti L.^1^, Bacuzzi A.^1^, Severgnini P.^1^, Boghi D.^2^, Legnani L.A.^1^

##### ^1^Department of Anesthesia and Intensive Care, Circolo University Hospital ~ Varese ~ Italia, ^2^asst settelaghi, Ospedale F. Del Ponte ~ varese ~ Italia

###### **Correspondence:** Baggio F.

**Background**

Etilefrine is a common drug used to treat intraoperative hypotension. It is a sympathomimetic agent. Its clinical effects are mediated through activation of the β1, β2 and α1 adrenergic receptors. In clinical practice, different hemodynamic responses are related to volemic status, vascular tone and cardiac function.

**Materials and methods**

We performed a prospective analysis of 13 patients undergoing general anesthesia (8 elective surgery, 5 urgent surgery), with continuous invasive or non-invasive (ClearSight™) hemodynamic monitoring, in which it was necessary to administer etilefrine for hypotension (SAP <90 mmHg or MAP <65 mmHg or <20% of baseline). We aimed to discover the adrenergic receptors activation through blood pressure wave analysis after etilefrine bolus. We registered preoperative data focusing on routinely drugs administration and intraoperative data in particular fluids administration and hemodynamic parameters.

**Results**

The blood pressure values (SAP, MAP and DAP) after a bolus of etilefrine assumed a triphasic pattern (Figure 1) in all 13 cases (27 administrations) independently from routinely preoperative drugs administration.

The analysis of the interaction of etilefrine with doxazosin (α1 antagonist) (Figure 2) and urapidil (β1 and α1 antagonist) (Figure 3) allowed to demonstrate this sequence of adrenergic receptors effects: β1 (blood pressure and heart rate increase), β2 (blood pressure drop), α1 (pressure increase) (Figure 4).

The duration of β1 and β2 phases is less than α1 phase.

There was a delay between the inotropic and chronotropic effect ꞵ1 mediated 19[(18-22)12-48] seconds (Figure 5-6).

The β2 response entity to a subsequent etilefrine administration is not related to fluid load.

In a septic patient there was a different curve in response to etilefrine bolus (low β2 response) (Figure 7).

**Conclusions**

This work provides some original contributions to understand etilefrine effects. The study design, the small cohort analyzed, the lack of standardized drug dose and the miscellaneous use of CO and SVV monitoring in the population are the main limits. The pressure/time curve morphology analysis after etilefrine administration (inotropic, vasodilating and vasopressor agent) could provide some useful informations to understand patient’s hemodynamic status and support clinical choices. This hypothesis has to be probed in subsequent specifically well designed studies.

**References**

1. Hengstmann JH, Weyand U, & Dengler HJ: The physiological disposition of etilefrine in man. Europ J Clin Pharmacol 1975; 9:179-187.

2. Frost BR, Frewin DB, & Downey JA: Vascular effects of etilefrin. Further studies to substantiate the predominant indirect sympathomimetic action of this agent. Aust J Exp Biol Med Sci 1979; 57(4):443-446.

3. Frost BR, Frewin DB, & Gerke DC: The effects of etilefrine on blood vessels in the rat tail. J Pharm Pharmac 1977; 29:272-275.

4. Frost BR, Halloran TN, Frewin DB, et al: The indirect sympathomimetic activity of etilefrine - a comparison with tyramine and ephedrine using 3H-noradrenaline. J Pharm Pharmacol 1978; 30:638-631.

5. Coleman AJ, Leary WP, & Asmal AC: The cardiovascular effects of etilefrine. Eur J Clin Pharmacol 1975; 8(1):41-45.

6. Stanton-Hicks M, Hoeck A, Stuehmeier K, et al: Venoconstrictor agents mobilize blood from different sources and increase intrathoracic filling during epidural anesthesia in supine humans. Anesthesiology 1987; 66:317-322.

7. Wajima Z, Shiga T, Imanaga K. Bolus administration of ephedrine and etilefrine induces transient vasodilation just after injection in combined epidural and general anesthesia patients: A randomized clinical study. Biosci Trends. 2018 Sep 19;12(4):382-388. doi: 10.5582/bst.2018.01074. Epub 2018 Aug 10. PMID: 30101824.

8. Meng L, Tran NP, Alexander BS, Laning K, Chen G, Kain ZN, Cannesson M. The impact of phenylephrine, ephedrine, and increased preload on third-generation Vigileo-FloTrac and esophageal doppler cardiac output measurements. Anesth Analg. 2011 Oct;113(4):751-7. doi: 10.1213/ANE.0b013e31822649fb. Epub 2011 Aug 4. PMID: 21821516.

9. Kong R, Liu Y, Mi W, Fu Q. Influences of different vasopressors on stroke volume variation and pulse pressure variation. J Clin Monit Comput. 2016 Feb;30(1):81-6. doi: 10.1007/s10877-015-9687-6. Epub 2015 Mar 26. PMID: 25808454.

10. Hadian M, Severyn DA, Pinsky MR. The effects of vasoactive drugs on pulse pressure and stroke volume variation in postoperative ventilated patients. J Crit Care. 2011 Jun;26(3):328.e1-8. doi: 10.1016/j.jcrc.2010.08.018. Epub 2010 Oct 30. PMID: 21036528; PMCID: PMC3103641.

11. Rebet O, Andremont O, Gérard JL, Fellahi JL, Hanouz JL, Fischer MO. Preload dependency determines the effects of phenylephrine on cardiac output in anaesthetised patients: A prospective observational study. Eur J Anaesthesiol. 2016 Sep;33(9):638-44. doi: 10.1097/EJA.0000000000000470. PMID: 27164015.

12. Kalmar AF, Allaert S, Pletinckx P, Maes JW, Heerman J, Vos JJ, Struys MMRF, Scheeren TWL. Phenylephrine increases cardiac output by raising cardiac preload in patients with anesthesia induced hypotension. J Clin Monit Comput. 2018 Dec;32(6):969-976. doi: 10.1007/s10877-018-0126-3. Epub 2018 Mar 22. PMID: 29569112; PMCID: PMC6209056.

**Fig. 1 (abstract A16).**
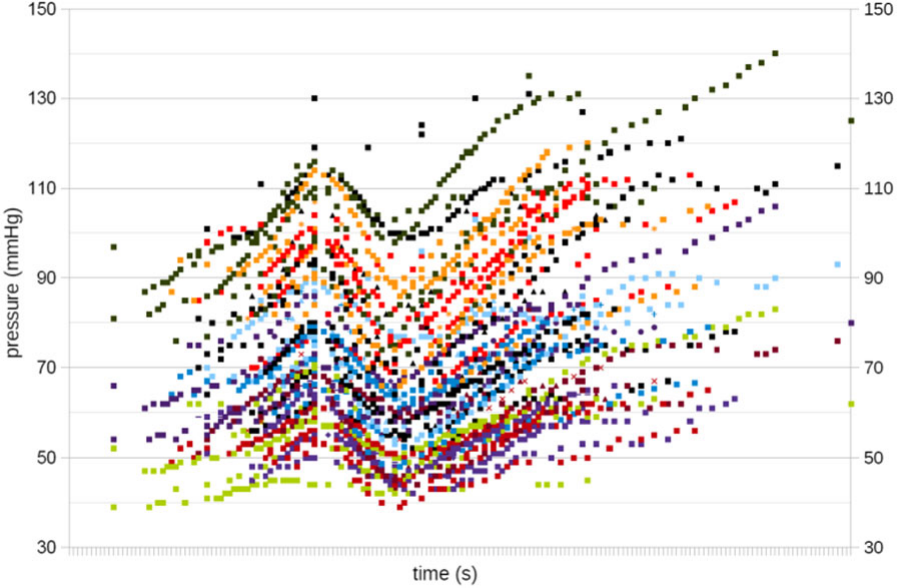
Triphasic pattern. Pressure trend after etilefrine administration (n=27). Data synchronized with the first BP peak

**Fig. 2 (abstract A16).**
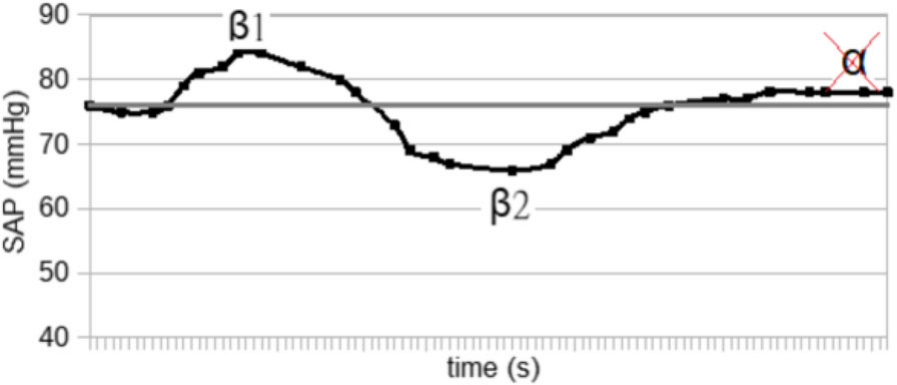
Interaction etilefrine-doxazosin

**Fig. 3 (abstract A16).**
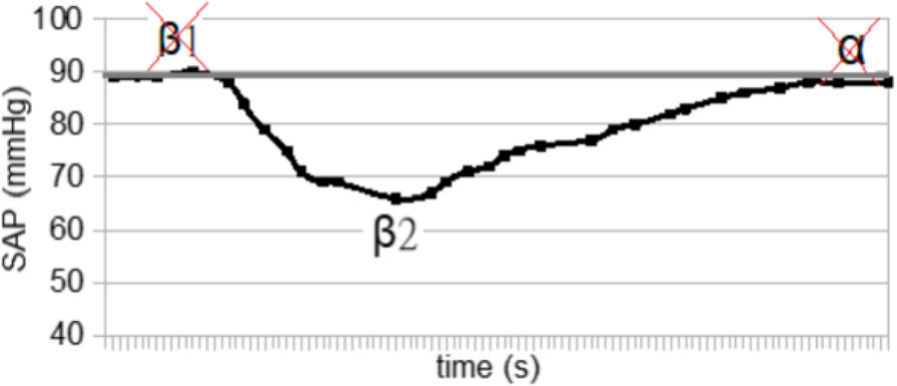
Interaction etilefrine-urapidil

**Fig. 4 (abstract A16).**
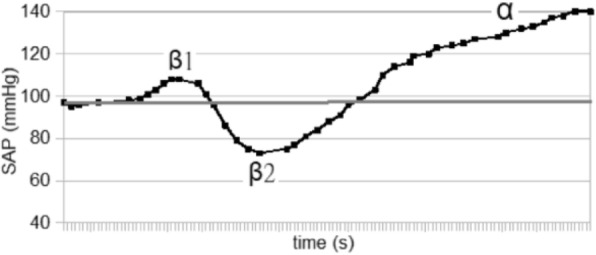
Etilefrine without interaction

**Fig. 5 (abstract A16).**
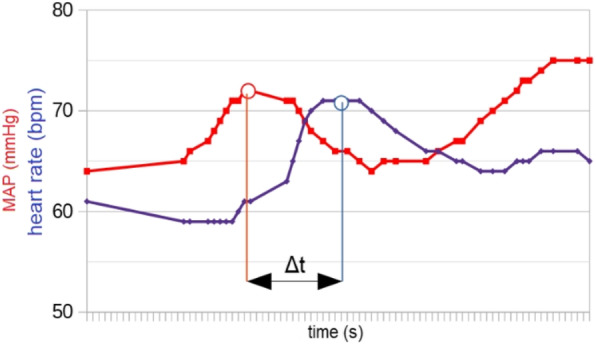
See text for description

**Fig. 6 (abstract A16).**
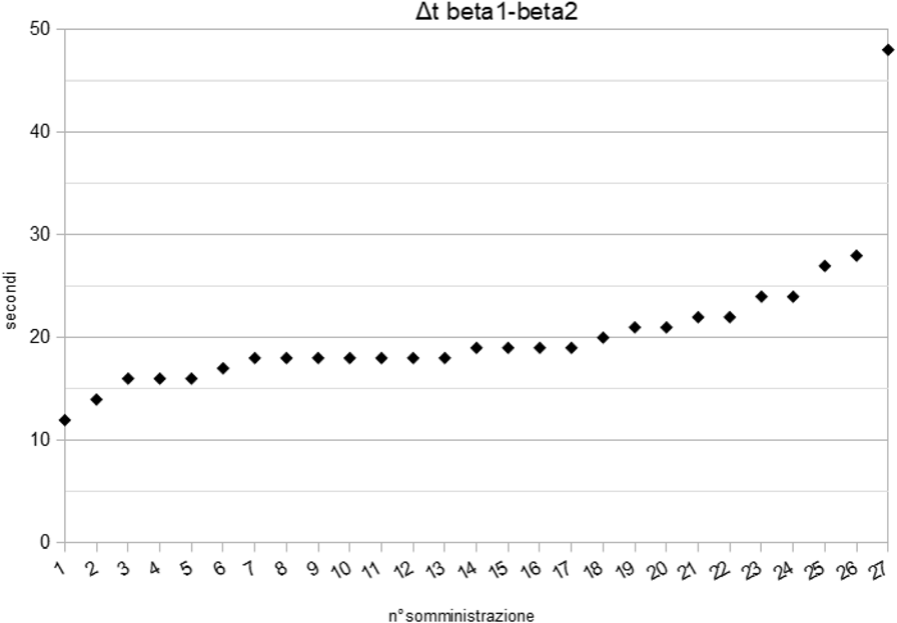
See text for description

**Fig. 7 (abstract A16).**
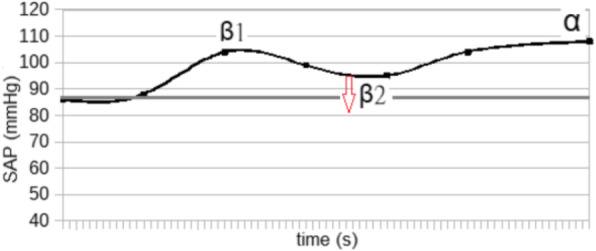
Septic patient

### A17. Serum total cholesterol concentration: a propofol washout marker

#### Fiume D.^1^, Carlini S.^2^, Galletti M.^1^

##### ^1^Department of Anaesthesia and Intensive Care Unit, Sant'Eugenio Hospital ~ Rome ~ Italia, ^2^Department of Anaesthesia and Intensive Care Unit, University of Rome Tor Vergata ~ Rome ~ Italia

###### **Correspondence:** Fiume D.

Background

In particular conditions such as low-perfusion syndromes, time of awakening can be longer. Propofol is a very fast-acting IV anesthetic agent, widely used all over the world. Propofol is a lipid emulsion solution based on soybean oil and medium chain triglycerides, purified egg phosphatides, glycerol, oleic acid and sodium hydroxide, in an aqueous injectable solutions. The effect of prolonged infusion on cholesterol and triglyceride metabolism has been known for a long time [1]. Furthermore, gender differences have been observed, but only for short-term infusions [2]. Regarding triglycerides, plasmatic peak was seen a little later than awakening after short-term infusion [3].

Materials and Methods

In our one-year experience in Post-Operative Intensive Care Unit (April 2020/April 2021), 10 patients were observed. Propofol (Fresenius Kabi Austria GmbH Hafnerstrasse 36 A-8055 Graz – Austria) infusion was longer than 60 hours. 5 patients were from General Surgery, 1 from Coronary Intensive Care Unit, 1 from Emergency Medicine, 1 from Neurology and 1 from Neurosurgery. Diagnosis was low-perfusion syndrome with different etiopathogenesis (Emorragic shock, Cardiogenic shock, Septic shock). Gender distribution was 7 males and 3 females, with median age 68.1 (25-86). Mortality rate was 60%. Neurological status was defined by Ramsey score (1-6).

Results

From our clinical observation there was a marked improvement in neurological status (Ramsey score 2/3) corresponding to a decreased total cholesterol serum concentration. In particular we noticed a Ramsey 2/3 achievement when total cholesterol serum concentration was below 200 mg/dl (5,17 mmol/L). No gender differences observed.

Conclusions

In our experience we noted a clinical features corresponding to serum total cholesterol concentration after long-term Propofol infusion. Serum total cholesterol concentration results in many metabolic pathways and could be changed by artificial nutrition or liver function too. If confirmed by other studies these findings suggest usefullness during pharmacological switch, difficult weaning or in differential diagnosis of coma.

References

1. Gottardis M, Khunl-Brady KS, Roller W, Sigl G, Hackl JM. Effect of ptolonged sedation with Propofol on serum triglyceride and cholesterol concentrations. Br J Anaesth 1989; 62: 393-396

2. Gan TJ, Glass PS, Sigl J, Sebel P, Payne F, Rosow C, Embree P. Women emerge from general anesthesia with propofol/alfentanil/nitrous oxide faster than men. Anesthesiology 1999; 90:1283–7

3. Ward DS, Norton JR, Guivarc’h PH, Litman RS, Bailey PL. Pharmacodynamics and Pharmacokinetics of Propofol in a Medium-Chain Triglyceride Emulsion. Anesthesiology 2002; 97, No 6

### A18. Intelligent operating room: a dream is becoming a reality

#### Bellini V.^1^, Craca M.^1^, Panizzi M.^1^, Pellegrino M.^2^, Mordonini M.^2^, Bottani E.^2^, Bignami E.^1^

##### ^1^Anesthesiology, Critical Care and Pain Medicine Division, Department of Medicine and Surgery, University of Parma, Viale Gramsci 14, 43126 Parma, Italy. ~ Parma ~ Italia, ^2^Department of Engineering and Architecture, Parco Area delle Scienze, 181/A - 43124 Parma, Italy ~ Parma ~ Italia

###### **Correspondence:** Bellini V.

Background

Perioperative medicine has many facets. The clinical management of the patient with highly specialized interventions makes it very complex, through the interaction of different professional figures. Optimizing the general organization from the earliest stages would mean having a better and more accurate management of the resources available, while guaranteeing a high quality of care, increasing safety for patients and healthcare professionals. With this study we want to highlight how it is feasible to use AI (artificial intelligence) models capable of tracing the patient along his entire perioperative care path, with the aim of leading to a more targeted organization of the OR (Operating Room), ultimately creating an integrated technological-organizational model capable of exploiting the data coming directly from the OR (1).

Materials and methods

The current study is in its initial phase. An innovative tracking system is used, easy and intuitive for the healthcare professional, however comfortable and in total respect of the patient involved in the study. The architecture provides for an internal localization of BLE (Bluetooth low energy) tags through a Raspberry Pi v4. The patient is informed of the study and once informed consent is obtained, a Tag is applied to him. Once worn, the Tag is hooked to the internal system and patients are assigned a progressive number. In addition, the use of a tablet in the Ricovery Room (RR) allows you to monitor in real time the movements in and out of the patient both from the operating room and from the RR itself. This architecture therefore allows you to create a solid database, which with the use of AI allows you to accurately predict the perioperative times, in particular those of occupation of the OR and therefore to create an adequate scheduling system early on, optimizing resources (1).

Results

The analysis of the first data is promising, making the study undoubtedly feasible and functional. First of all, it is possible to monitor the patient in all the perioperative phases. It immediately emerged that the times recorded in these phases are much more precise and in real time than those present in the computerized system of the SB (operating block). The time difference expressed in mean% varies from 6.90% (19.3 min) for patients present in the SB, to 11.10% (17.1min) for the stay in the OR and even 27.80% (24 , 5 min) in RR. The AI can easily predict the timing of the intervention based on some variables including: the operator's skills, the complexity of the surgery, the patient's ASA score and the type of anesthesia performed. (Figure1)

Conclusions

It is therefore possible, right from the start, to optimize the organization times of the OR and, more generally, of the OS, in every phase of the clinical path and patient management, thus improving scheduling, even on a daily basis. Ultimately, it is essential to create a database that is able to implement the technological-organizational system already outlined in the initial phase.

**Reference**

1. Dagli MM, Rajesh A, Asaad M, Butler CE. The Use of Artificial Intelligence and Machine Learning in Surgery: A Comprehensive Literature Review. Am Surg. 2021 Dec 27:31348211065101. doi: 10.1177/00031348211065101. Epub ahead of print. PMID:34958252.

**Fig. 1 (abstract A18).**
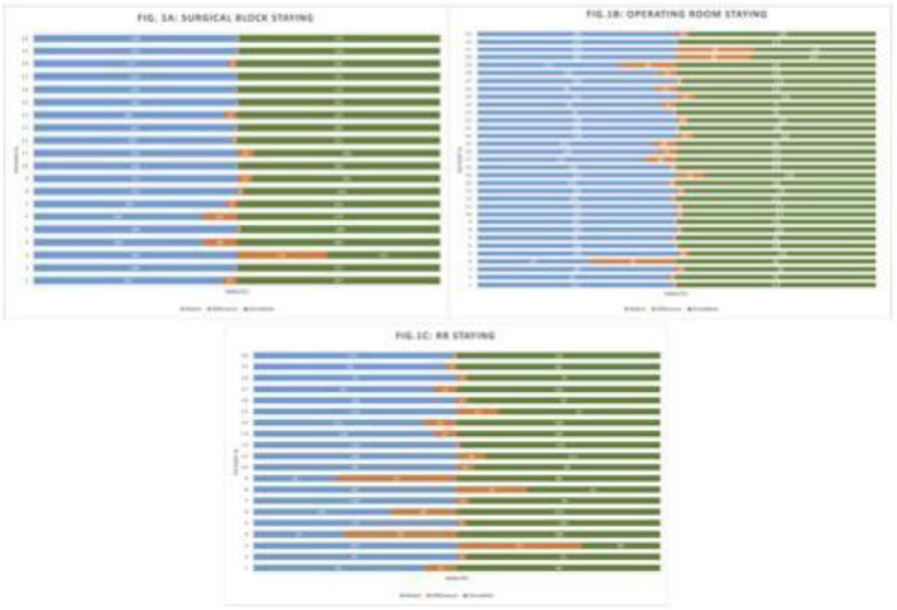
**A**: Surgical block staying. In blue time (min) recorded by the watch (min). In green time (min) recorded on OrmaWeb. In orange the difference (min) between the two records. The mean difference between the two records is 19.3 minutes with a mean % difference of 6.9%. **B**: Operating room staying. In blue time (min) recorded by the watch (min). In green time (min) recorded on OrmaWeb. In orange the difference (min) between the two records. The mean difference between the two records is 17.1 minutes with a mean % difference of 11.1%. **C**: Recovery room staying. In blue time (min) recorded by the watch (min). In green time (min) recorded on OrmaWeb. In orange the difference (min) between the two records. The mean difference between the two records is 24.5 minutes with a mean % difference of 27.8%

### A19. Hypotension prediction index in liver and pancreatic surgery: prelimary experience

#### Picchio F.^2^, Briozzo M.^2^, Tavoletti D.^1^, Cerutti E.^1^, Cerchiara P.^1^, Lisanti I.^1^

##### ^1^AOU Ospedali Riuniti Ancona Department of Anesthesia, Transplant and Surgical Intensive Care ~ Ancona ~ Italia, ^2^Università Politecnica delle Marche ~ Ancona ~ Italia

###### **Correspondence:** Cerchiara P.

**Background**

Hypotension is common during anesthesia and associated with adverse outcomes. A new hemodynamic monitoring Hypotension Prediction Index (HPI), Edwards Lifesciences, has the ability, using artificial intelligence to analyse arterial waveforms, to predict hypotensive events up to 15 minutes before they occur allowing a proactive treatment of hypotension (1). Our aim is to describe the incidence, duration and severity of hypotension when using HPI compared with FloTrac monitoring in patients submitted to liver and pancreatic surgery.

**Materials and methods**

Data collected intraoperatively were downloaded retrospectively from the HemoSphere platform and analyzed using the Acumen Analytics Software, Edwards Lifesciences. All patients monitored with either FloTrac or HPI-Acumen IQ sensor undergoing hepato-pancreatic surgery from October 2019 to July 2021 at our centre were included. A radial arterial line was placed before anesthesia induction and monitoring started immediately after insertion. The decision to use advanced hemodynamic monitoring was based on the risk assessment by senior anesthesiologist. Exclusion criteria were cardiac arrhythmias, aortic regurgitation, emergency. Hypotension was defined as a mean arterial pressure (MAP) < 65 mmHg for at least 1 minute. The study endpoints were the number of patients with hypotension, the average number and total duration of hypotensive events per patient, the duration of hypotensive events per patient in percentage of monitoring time to capture post-induction hypotension, the area under threshold (AUT) for MAP <65mmHg per patient and time weighted average (TWA) of AUT for MAP <65mmHg per patient indicating severity and duration of hypotension in relation to surgery time. Data are presented as median and interquartile range [IQR], variables analyzed with Wilcoxon Rank Test, P-value < 0.05 considered statistically significant.

**Results**

The study included 103 patients submitted to major open or laparoscopic hepato-pancreatic surgery . In FloTrac group 67/71 (94%) patients experienced hypotension versus 27/32(84%) in HPI group. The duration and severity of hypotension were significantly reduced in HPI group as shown in Table 1.

**Conclusions**

In our experience incidence, duration and severity of intraoperative hypotension are high when compared with literature data (2). Many factors as vascular clamping, volume restriction in liver surgery, bleeding, high-risk patients, learning curve in laparoscopic surgery could explain our findings. HPI use results in reduction of duration and severity of hypotension, more relevant if total monitoring time instead of surgery time is considered. HPI seems to be a useful parameter to increase vigilance to hemodynamics in high-risk surgery expecially in preventing hypotensive events not related to surgical manipulations.

**References**

1) Hatib F, Jian Z, Buddi S et al. Machine-learning Algorithm to Predict Hypotension Based on High-fidelity Arterial Pressure Waveform Analysis. Anesthesiology. 2018 Oct;129(4):663-674.

2) Wijnberge M, Geerts BF, Hol L, Lemmers N et al. Effect of a Machine Learning-Derived Early Warning System for Intraoperative Hypotension vs Standard Care on Depth and Duration of Intraoperative Hypotension During Elective Noncardiac Surgery: The HYPE Randomized Clinical Trial. JAMA. 2020 Mar 17;323(11):1052-1060.

**Table 1 (abstract A19).** Characteristics of hypotension

	FLO TRAC (71)	HPI (32)	P-value
Number of hypotensive events per patient	6 [3 11]	5 [2 10]	0.15
Total duration of hypotensive events per patient (minutes)	31.3 [12.4 52.3]	15.2 [3.2 44.7]	0.037
Total duration of hypotensive events per patient in % of monitoring time (%)	10.8 [3.9 21.8]	6.7 [1.3 13.3]	0.008
AUT for MAP< 65mmHg per patient (mmHg*min)	200.7 [59.1 423.7]	122 [13.2 304.6]	0.067
TWA of AUT for MAP< 65mmHg per patient ( mmHg)	0.72 [0.25 1.64]	0.36 [0.05 0.96]	0.039

### A20. Optimization of intraoperative ventilation guided by electric impedance tomography in obese patients undergoing robot-assisted radical prostatectomy: randomized prospective study - preliminary results

#### Torregiani G., Covotta M., Fabbrocile L., Tola G., Claroni C., Forastiere E.M.A.

##### IRCCS Istituto Nazionale Tumori Regina Elena ~ Roma ~ Italia

###### **Correspondence:** Fabbrocile L.

Abstract

Background and Goal of Study: Robotic-assisted radical prostatectomy is performed with the use of the pneumoperitoneum and with the extreme trendelenburg position. These conditions cause important alterations in respiratory mechanics and cerebrovascular dynamics. The obese patient has reduced chest expandability and increased cardiac stress and oxygen requirements. The primary objective of our study will be to verify a possible improvement in arterial oxygenation if the ventilation will be guided by electrical impedance tomography (EIT) rather than by peripheral saturation alone. Materials and Methods: This prospective trial was approved by the local ethics committee. Between September 2020 and December 2021, 32 Obese patients (> 30 BMI) scheduled for robotic-assisted radical prostatectomy were enrolled. Patients were randomized into two groups of 16 patients each. In group S, only tissue oxygenation monitoring was performed using SpO2. In the S+E group, additional monitoring with electrical impedance tomography (EIT) was performed. Gas exchanges, ventilatory, hemodynamic and tomographic parameters were recorded at the following time points: in supine post induction position (T1), 10min post pneumoperitoneum and trendeleburg position (T2), 1h after T2 (T3), in supine pre extubation position (T4) Results and Discussion: Baseline characteristics were similar in both groups. The PaO2/FiO2 ratio was significantly higher in the S+E group at T2 (303 ± 128 vs 232 ± 39 P <0.05). Tidal volume was significantly lower in the S+E group at T2 (480 ± 52 vs 525 ± 39 P <0.05), T3 (475 ± 62 vs 530 ± 47 P <0.05) and T4 (492 ± 47 vs 551 ± 25 P <0.05). The tidal volume / ideal weight ratio (ml/kg) was lower in the S + E group at T2 (7.23 ± 0.8 vs 7.8 ± 0.8 P <0.05), T3 (7.14 ± 0.8 vs 7.9 ± 0.8 P <0.05) and T4 (7.43 ± 0.75 vs 8.2 ± 0.7 P <0.05). Peak pressure and plateau pressure were lower in the S + E group at T4 (20.7 ± 5.7 vs 26.7 ± 5 P <0.05 and 14.9 ± 3.7 vs 19.6 ± 7.3 P <0.05). Conclusion(s): The use of EIT has led to an intraoperative improvement in arterial oxygenation and a significant reduction in tidal volume, peak pressures and plateau pressures.

**Table 1 (abstract A20).** See text for description

	SpO2 (n=16)			SpO2 + EIT (n=16)					
	T2	T3	T4	T2	T3	T4	P (T2)	P (T3)	P (T4)
PaO2/FiO2	232 ± 39	286 ± 76	209 ± 97	303 ± 128	246 ± 64	275 ± 128	**< 0,05**	0,2	0,115
Tidal Volume (cmH2O)	525 ± 39	530 ± 47	551 ± 25	480 ± 52	475 ± 62	492 ± 47	**< 0,05**	**< 0,05**	**< 0,05**
Tidal Volume/PBW (ml Kg-1)	7,8 ± 0,8	7,9 ± 0,8	8,2 ± 0,7	7,23 ± 0,8	7,14 ± 0,8	7,43 ± 0,75	**< 0,05**	**< 0,05**	**< 0,05**
PEEP (cmH2O)	3,6 ± 1,7	5 ± 2,1	4,5 ± 1	4,4 ± 1	4,5 ± 1,2	4,6 ± 1,2	0,144	0,2	0,879
Peak Pressure (cmH2O)	37,3 ± 5,3	39,6 ± 2,9	26,7 ± 5	37,2 ± 3,3	38,4 ± 3,4	20,7 ± 5,7	0,93	0,3	**< 0,05**
Plateaux Pressure (cmH2O)	29 ± 3,5	32 ± 3	19,6 ± 7,3	29,3 ± 3,6	31,1 ± 3,9	14,9 ± 3,7	0,919	0,678	**< 0,05**
Minute Ventilation (L min-1)	9,9 ± 1,7	10,4 ± 1,8	10 ± 1,7	8,5 ± 1,1	9,3 ± 1,1	8,7 ± 1,2	0,15	**< 0,05**	**< 0,05**
PaCO2 (mmHg)	42,3 ± 4,1	41,5 ± 6,4	43 ± 4,8	39,3 ± 2,7	44 ± 3,2	40,4 ± 6,8	**< 0,05**	0,425	0,173
Sp02 (%)	97,8 ± 1,2	97,1 ± 1,7	97,8 ± 1,1	98,1 ± 1,2	98 ± 1,4	98,3 ± 1	0,38	0,13	0,15
PAM (mmHg)	87 ± 8	89 ± 12	73 ± 5	102 ± 12	85 ± 15	76 ± 7	**< 0,05**	0,55	0,36
EtCO2 (mmHg)	32,1 ± 5	34 ± 6	34 ± 4	30,8 ± 2,8	35,6 ± 8	31,5 ± 3,6	0,26	0,61	**< 0,05**

### A21. Preoperative anemia and iron supplementation in patients undergoing non-cardiac surgery. The experience of a local health agency in liguria in 2021

#### Accornero L.^3^, Piazza M.^2^, Schenone S.^1^, Liggieri L.^2^, Casanova S.^3^, Muzio L.C.^3^, De Bellis A.^3^, Paruta L.^3^, Mentore B.^1^, Bonfiglio M.^3^

##### ^1^ASL 4 Chiavarese - Direzione sanitaria Aziendale ~ Lavagna ( Genova ) ~ Italia, ^2^ASL 4 Dipartimento dei Servizi - S.C Centro Trasfusionale ~ lavagna ( Genova) ~ Italia, ^3^ASL 4 Chiavarese - Dipartimento Cardiologico e dell'Emergenza Urgenza - S.C Anestesia e Rianimazione ~ Lavagna ~ Italy

###### **Correspondence:** Accornero L.

Preoperative anemia increases postoperative morbidity and mortality and the risk of perioperative transfusions in surgical patients [1-2-3]. The most common treatable cause of preoperative anemia is iron-deficiency. Multidisciplinary team (composed of an anesthesiologist, a surgeon and a Transfusion Centre physician) determines a specific plan for optimise the patient’s own blood volume and minimise blood loss through PBM (Patient Blood Management) implementation. There are various techniques to ensure this, for example intravenous iron intravenous (IV) infusion.

Aim of the present observational study is to follow the PBM application and the requirements of blood trasfusions. We studied patients urdergoing elective non-cardiac surgery. Inclusion criteria comprehend: oral iron is ineffective or not tolerated; severe anemia; pre-operative time is at least 4 weeks. PMB is managed with an internal procedure.

Surgical interventions decreased in the first half of 2021 due to pandemic response, conversely increased in the second half of 2021 due to post-pandemic surgery recovery. Noteworthy, blood-stocks were reduced in the whole 2021 due to the distancing amongst donation sessions. In period 1, number of patients (P) who undertaken pre-operative laboratory tests were 1.740; number of pre-operative anemic patients (P) (Hb<12-13 mg/dL) were 366 (21,0%); number of patients (P) who undertaken PMB were 21 (21/366, 5.74%); number of blood bags consumed (N) were 517; number of surgical interventions (N) were 1.623. In period 2, number of patients (P) who undertaken pre-operative laboratory tests were 3.530; N of of pre-operative anemic patients (P) (Hb<12-13 mg/dL) were 511 (14,5%); number of patients (P) who undertaken PMB were 53 (53/511, 10.37%); number of blood bags consumed (N) were 633; number of surgical interventions (N) were 3.168. Current best practice guidance supports the routine administration of pre-operative IV iron to treat anemia despite limited evidence. Detection of pre-operative anemia is critical for the management of surgical patients. Our small study showed that there are two prevalent groups of anemic patients (young women and old men), and probably the “one size fits all” treatment may not be correct. While clinicians should continue to strive to identify and treat pre-operative anemia (the strong cooperation between Anesthesiology Intensive Care and Transfusion Centre boosted the PMB increment and the blood bags consumption sparing), further research is required for identify preoperative hemoglobin level target in women and men, the timing of intervention and how to approach the groups that could benefit most.

References

1. Miles LF, Richards T. Hematinic and Iron Optimization in Peri-operative Anemia and Iron Deficiency. Curr Anesthesiol Rep. 2022. 12:65-77

2. Richards T, Baikady RR, Clevenger B, Butcher A, Abeysiri S, Chau M, Macdougall IC, Murphy G, Swinson R, Collier T, Dyck LV, Browne J, Bradbury A, Dodd M, Evans R, Brealey D, Anker SD, Klein A. Preoperative intravenous iron to treat anaemia before major abdominal surgery (PREVENTT): a randomised, double-blind, controlled trial. Lancet 2020; 396: 1353–61

3. PATIENT BLOOD MANAGEMENT ITALIA - http://pbm.centronazionalesangue.it/pagine/patient-blood-management-italia.html

**Fig. 1 (abstract A21).**
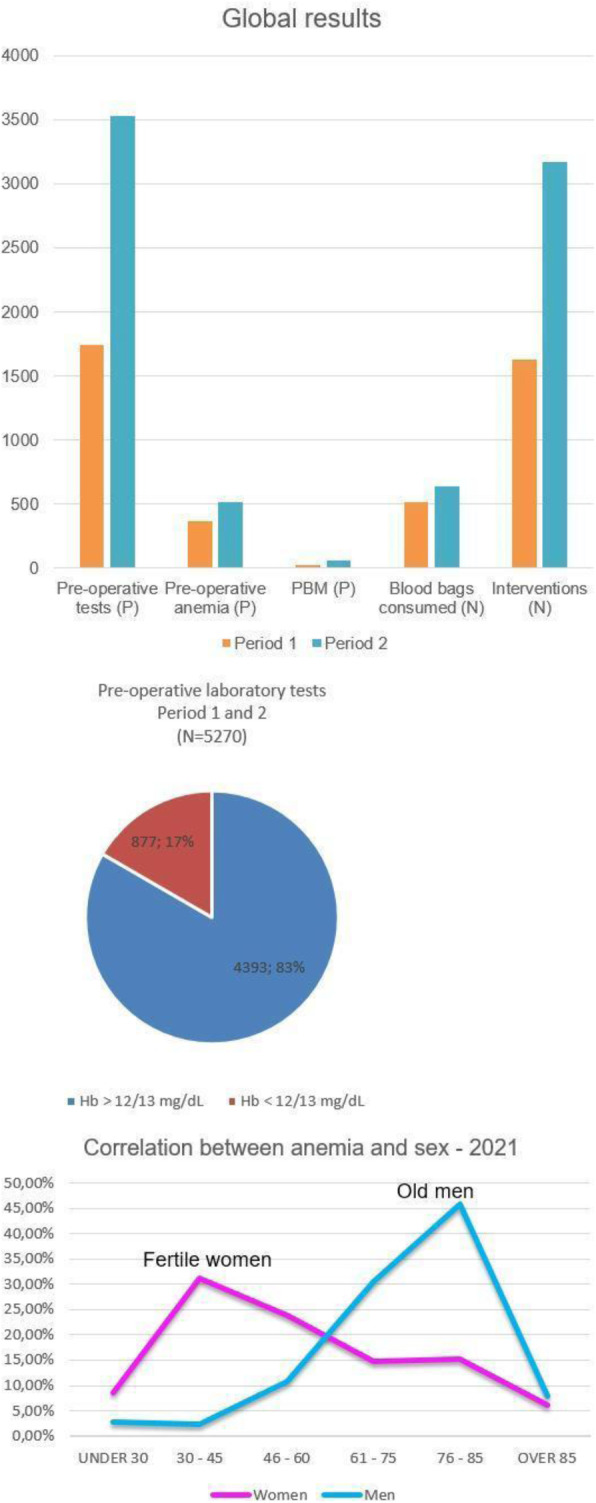
See text for description

### A22. Ancient grain bread rich in iron, zinc, flavonoids and alpha lipoic acid as a novel preoperative functional food for suppressing the progression of cardiovascular remodeling in infarcted male rats

#### Casieri V.^1^, Matteucci M.^1^, Ercoli L.^3^, Passino C.^3^, Ricci L.^2^, Lionetti V.^1^

##### ^1^Unit of Translational Critical Care Medicine, Scuola Superiore Sant'Anna ~ Pisa ~ Italia, ^2^Departmen of Life Sciences, University of Siena ~ Siena ~ Italia, ^3^Scuola Superiore Sant'Anna ~ Pisa ~ Italia

###### **Correspondence:** Casieri V.

BACKGROUND: The number of patients who require non-cardiac surgery following an acute myocardial infarction (AMI) is likely to increase due to aging of surgical population and risk for postoperative heart failure (HF) is increased as effective secondary prevention is missing. Nutritional preconditioning with sustained dietary intake of functional food targets an increase in the functional reserve preoperatively to optimize postoperative cardiac recovery even when the patients are not malnourished. However, a one-size-fits-all approach to nutrition is inappropriate and new personalized functional foods are necessary. Functional bread (FB) made from iron- and zinc-biofortified “Gentil Rosso”, a Tuscan ancient grain, contains higher levels of bioavailable iron (+180%), zinc (+640%), flavonoids (+1000%) and alpha lipoic acid (+50%) compared to regular bread (RB). We hypothesized that the regular intake of normocaloric diet supplemented with FB prevents HF after MI in both sexes.

MATERIALS AND METHODS: Adult male and female Wistar rats were subjected to permanent ligation of left anterior descending coronary artery and fed for 6 weeks with normal diet (3.344 kcal/g, 6.2% fat, 18.7% protein, 51% carbohydrate) supplemented with FB (AMI+FB, n=10 males, n=6 females) or normal bread (AMI+RB, n=10 males, n=6 females). Intraperitoneal glucose tolerance test (IPGTT) and cardiac echocardiography were performed before and after diet. The infarct scar size, capillary and arteriolar density were quantified histologically. The myocardial inflammatory response was evaluated by ELISA assay of interleukin (IL)-1α, -1β, -2, -10 and -4.

RESULTS Basal plasma glucose levels and IPGTT did not change in both sexes. The decline in left ventricular (LV) ejection fraction of male AMI+FB rats was significantly smaller (-12±1%) compared to male AMI+RB animals (-40±5%). Yet, no differences were detected between female AMI+FB and AMI+RB. The LV infarct scar size of male AMI+FB rats was smaller than male AMI+RB animals (30.5±3 vs 42 ± 3.3%, p<0.001), but no differences were detected in treated female rats. The capillary and arteriolar density in the LV border zone of male AMI+FB rats were higher than untreated male animals (+16.3 vs. +15.35%). We did not find any difference in capillary and arteriolar density in the LV border zone of treated female rats. Finally, lower levels of IL-1α, -1β, -2, -10 and -4 were expressed in the LV border zone of treated male infarcted rats (p<0.01). Conversely, cytokine expression in the remodeled LV region was significantly higher in female AMI+FB animals.

CONCLUSIONS: Regular intake of normal diet supplemented with FB hampered cardiac remodeling and limited LV function decay in male, but not female infarcted rats. Bio-fortified bread can be a valid preoperative functional food for suppressing adverse post-AMI cardiac remodeling in a personalized manner.

### A23. Effect of crystalloids with different SID on hemodilution and PH in patients undergoing major surgery: the crysid physiologic study

#### Dell”Anna A.M.^1^, Dominedò C.^2^, Cicetti M.^1^, Festa R.^1^, Lamacchia R.^1^, Cisterna I.^1^, Filetici N.^1^, Giannì G.^1^, Michi T.^1^, Rossi M.^1^, Antonelli M.^1^

##### ^1^Fondazione Policlinico Universitario A. Gemelli IRCCS, Dipartimento di scienze dell’emergenza, anestesiologiche e della rianimazione ~ Roma ~ Italia, ^2^Azienda Ospedaliera San Camillo Forlanini, Dipartimento di shock e trauma ~ Roma ~ Italia

###### **Correspondence:** Lamacchia R.

**Background**

Over the last few years, many studies have shown that the amount and the type of fluid administered during anesthesia may affect the clinical outcome in terms of morbidity and mortality^[1]^.

Emerging data suggest that intravenous administration of fluids can produce acid base disturbances with possible clinical consequences^[2]^. Available crystalloids can be classified according to their Strong Ions contents. SID is defined as the difference between strong cations and anions.

The aim is to test the hypothesis that the administration of fluids with different electrolytic composition has an impact on the plasma pH of patients undergoing vertebral surgery.

**Methods**

This is a single center, prospective, clinical trial performed in the Operating Room of Policlinico A. Gemelli of Rome during major Spine Surgery.

Patients undergoing vertebral arthrodesis were considered eligible if they were under 70, with ASA<3, with estimated surgery duration of at least 3 h. Patients were randomly assigned to receive sodium chloride solution 0.9% (SID 0), or balanced crystalloids as lactated Ringer’s solution (SID 28) or Crystalsol® solution (SID 50).

In each group patients received maintenance fluid therapy 1-2 ml/kg/h and fluid boluses when required by clinical evidence. In particular we administered 2 fluid boluses of 10 ml/kg and 20 ml/kg at least 1 h apart from each other. Data regarding acid base balance and plasma chemistry were collected after anesthesia induction as Time Point 0 (T0), 5 min after the first bolus (T1), 1 h after the first bolus (T2), immediately before the second bolus (T3), 5 min after second bolus (T4), 1 h after second bolus (T5). Urinary chemistry data were collected at T0, T2, T3 and T5.

The primary outcome was evaluate, in vivo, the effect of crystalloids administration with different SID on acid base equilibrium. PH and SID variations will be considered.

The second aim was to analyze the renal response to acid base disturbances caused by different crystalloid infusions and different volumes.

**Results**

We enrolled 45 patients, 15 for each group, whose median age 50 (40-65), including 21 males (46%) with median ASA 2 (1-2). The anesthesia had a median duration of 250 min (205-342). (Table 1)

As regards the primary endpoint, the plasma pH of the patients who received Crystalsol® was significantly higher than the other 2 groups starting from T2 (Figure 1), in particular 1 h after the second bolus the pH was 7.42 (7.38- 7.45) in Crystalsol® group vs 7.40 (7.35-7.43) lactated Ringer's group vs 7.34 (7.32-7.36) in the saline solution group (*p* <0.001). The paCO_2_ is not different between the 3 groups.

The plasmatic SID was significantly higher in patients who received Crystalsol® *p* <0.001 and chloremia was significantly lower in patients who received Crystalsol® (*p* <0.001).

The sodium load administered during surgery was not different in the 3 groups, while the chlorine load was significantly higher in patients who received saline solution than in patients who received lactated Ringer's or Crystalsol® (388 [305-439] vs 316 [218-362] vs 255 [177-310] *p* <0.001 respectively) .

Concern urinary changes, we recorded a significant increase in chlorine excretion in patients who received saline solution compared to those who received Crystalsol® (86 [46-146] vs 31 [20-86] *p* <0.02 respectively).

Urinary SID was significantly higher in patients who received Crystalsol® than in the group that received Ringer or saline solution (102 [55-112] vs 14 [4-51] vs 23 [11-33] *p* <0.001).

**Conclusions**

Our study has shown that administration of fluid with different electrolyte composition has a significant impact on plasma pH and urinary elimination of electrolytes.

Further studies are needed to evaluate the clinical relevance of these physiological changes in the perioperative setting.

**References**

1. Silva JM, et al.: The effect of excess fluid balance on the mortality rate of surgical patients: a multicenter prospective study. Critical Care 2013, 17:R288.

2. Prough DS, Bidani A (1999) Hyperchloremic metabolic acidosis is a predictable consequence of intraoperative infusion of 0.9% saline. Anesthesiology 90:1247–1249.

**Fig. 1 (abstract A23).**
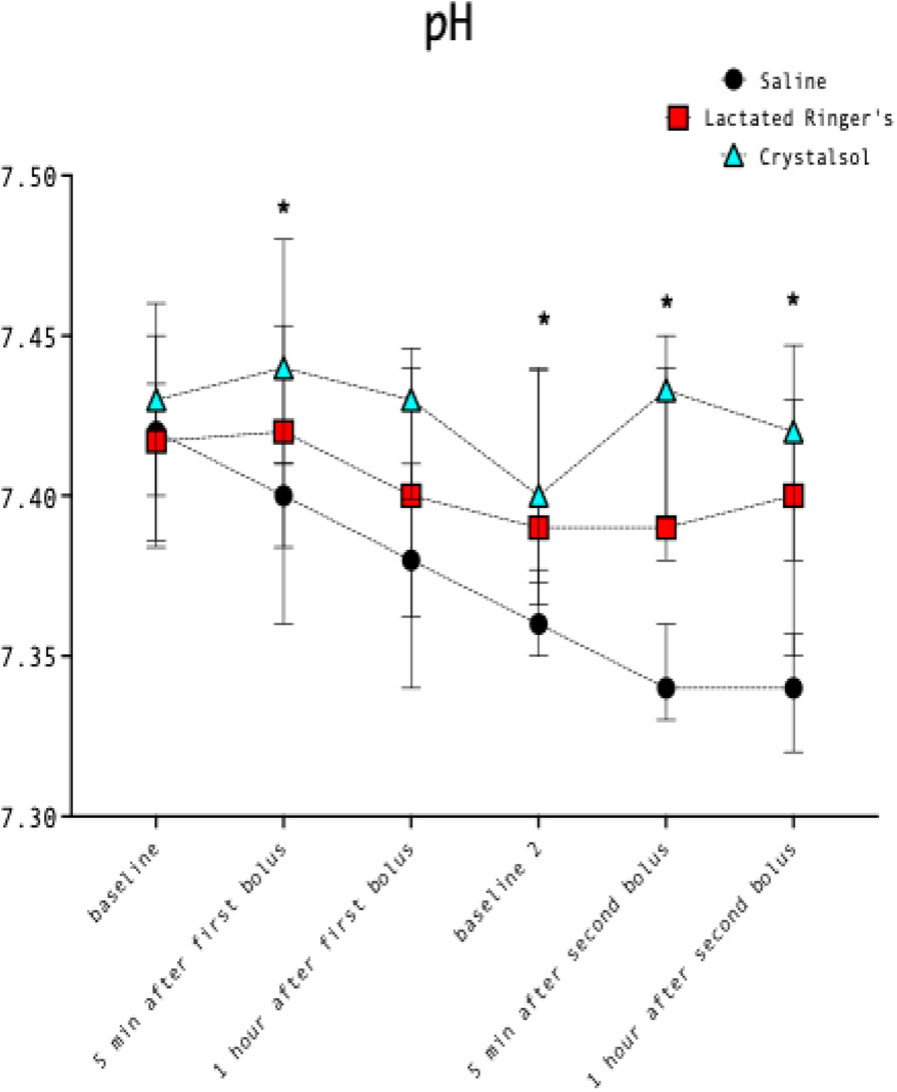
See text for description

**Table 1 (abstract A23).** See text for description

**Age, year median (IQR)**	**50 (40-65)**
**Sex, male n (%)**	21 (46.7)
**Weight, kg median (IQR)**	70 (61-85)
**Height, cm median (IQR)**	170 (163-168)
**BMI, kg/cm**^**2**^ **median (IQR)**	25 (22-27)
**ASA, median (IQR)**	2 (1-2)
**Anesthesia duration, min median (IQR)**	320 (270-400)
**Surgery duration, min median (IQR)**	250 (205-342)
**Creatinine, mg/dl median (IQR)**	0.77 (0.69-0.91)

### A24. Opioid-free blended anaesthesia (spinal anaesthesia and local anaesthetics intravenous infusion) in laparoscopic colon surgery

#### Boselli A., Cappelletto D., Manfrini A., Gagliardi G.

##### Dipartimento di Emergenza-Urgenza - UOC Anestesia e Rianimazione ~ Rovigo ~ Italy

###### **Correspondence:** Boselli A.

Background

Excessive opioids doses during general anaesthesia can cause post operative adverse effects like respiratory depression, stypsis, PONV, immunosuppression, addiction and resistance, hyperalgesia and delirium, producing a longer hospitalization of surgery patients.

Purpose

Our purpose is to develop a protocol about opioid free anaesthesia with lidocaine infusion and spinal anaesthesia.

Methods

We have selected ASA 1 patients with laparoscopic hemicolectomy indication. We have made spinal anaesthesia T12-L1 using levobupivacaine 0.5% 1.5 ml and then we have started general anaesthesia (propofol 2 mg/Kg, lidocaine 1 mg/Kg, rocuronium 0.6 mg/Kg). The maintenance has been driven by infusion of propofol (6 mg/kg/h) and lidocaine (1.5 mg/kg/h), with anaesthesia depth monitoring. We have prescribed Paracetamol and NSAIDs for the post operative pain management.

Conclusions

All patients have received opioid free anaesthesia with no opioid use in 24 hours after surgery, good pain control and fast intestinal motility recovery. Adverse effects and periprocedural complications haven’t been noted.

### A25. Passive leg raising (PLR) and mini-fluid challenge (MFC): volume optimization in major surgery: prospective study using the starling TM monitor

#### Suppressa M.^1^, Foti L.^1^, Iacopetti G.^1^, Zaccaria R.^2^, Villa G.^1^, Barbani F.^1^, Baldini G.^1^, Romagnoli S.^1^

##### ^1^Health sciences department, section of anesthesiology, intensive care and pain medicine, Careggi University Hospital, Firenze, Italy ~ Firenze ~ Italy, ^2^Health sciences department, section of anesthesiology, intensive care and pain medicine, University Hospital Meyer, Firenze, Italy ~ Firenze ~ Italy

###### **Correspondence:** Suppressa M.

Introduction

Guidelines recommend 2-h fasting for clear liquids to ensure euvolemia before surgery. Few methods are available to assess fluid responsiveness in awake spontaneously breathing patients: Passive Leg Raising (PLR) and mini-fluid challenge (mFC) are among them. Fluid Responders (FRs) are those who increase Stroke Volume (ΔSV) > 10% after PLR or mFC. The Starling TM SV Monitor (Cheetah Medical-Baxter) identifies FRs thanks to bioreactance technology.

Objectives

To evaluate fluid responsiveness preoperatively by PLR, and intraoperatively by mFC.

Methods

Preoperative PLR test was performed to patients undergoing major surgery before the anesthesia induction. FRs received 250 ml of balanced crystalloid solution and fluid responsiveness was re- tested at the end of the bolus and again until a non-FR state was achieved. Baseline intraoperative fluid therapy was set at 5 mL/kg/h until the end of surgery. Hourly, mFCs with 100 ml of crystalloids balanced solutions were performed and FRs received the 250 ml-fluid bolus. Blood [Hb]- concentration was measured before surgery and hourly.

Results

Fifty-nine patients were enrolled. Forty-four % of the patients were FRs. Average fasting time was 13.8 (4.13)h in FRs and 13.45 (2.99)h in non-FRs (p=NS). After the first crystalloid bolus, 16 (61.5 %) FRs became non-FRs. Intraoperatively, 117 mFCs were performed in the first 3 hours and 18 FRs were found (15.4%). In all FRs, after the bolus, the mFC test was re-performed and no patient resulted to be FR. The [Hb] variation before and after 60 minutes from surgery start was 0.51 (0.61)g/dL for FRs and 0.26 (0.48)g/dL for non-FRs.

Conclusions

Preoperative fasting does not correlate with PLR test. After having tested for fluid responsiveness and corrected volemia, [Hb]-concentration can be used as a sensitive indicator of fluid expansion- restriction in non-bleeding patients. PLR and mFC tests, are easy, reliable and may be efficient tools to guide volume optimization during major surgery.

### A26. Fibromyalgia and acute perioperative pain management: a case report for new future approaches

#### Manzoni S., Grasso A., Sibilio A., Ciaravola M., Fiorito R., Piccinocchi R., Scafuto A., Ferraro F.

##### Università degli Studi della Campania Luigi Vanvitelli, Dipartimento della Donna, del Bambino e . di Chirurgia Generale e Specialistica ~ Napoli ~ Italy

###### **Correspondence:** Manzoni S.

Fibromyalgia is a multifactorial rheumatic disorder of unknown aetiology, affecting 2.7% of the world population (1M:3F). This illness manifests itself with widespread chronic pain, asthenia, cognitive, gastrointestinal and urinary disturbances, altered thresholds of nociception, proprioception and auditory perception, dysmenorrhea, vaginismus, and mood and sleep disorders. At the CNS level, levels of glutamate, substance P and endogenous opioids are significantly elevated, biogenic amines are reduced, the hypothalamic-pituitary-adrenal axis is dysregulated, pain modulation through descending pathways is altered (with impaired serotonergic-noradrenergic activity), and there is low responsiveness to exogenous opioids.

Diagnosis (often late) is clinical. No diagnostic biomarker is available, and chronic pain symptoms are managed through patient education, some nonpharmacologic treatments, and the use of analgesics (acetaminophen and NSAIDs), antidepressants, gabapentinoids, weak opioids, and cannabinoids. Little is known about acute pain control. In the perioperative period, gabapentinoids are recommended but are not particularly effective.

We report our experience regarding anaesthesia and pain management during hemorrhoidectomy in a 44-year-old patient, BMI 22.1, smoker, suffering from rheumatoid arthritis, hydrosadenitis, fibromyalgia, ankylosing spondylitis, dermatography, being treated with Humira, evaluated with a METS >4 and ASA II. Following the onset of autoimmune disorders, the patient reported poor sensitivity to local anaesthesia and an episode of awareness under general anaesthesia. Based on the history, clinical data, and patient interview (anxious state resulting from past anaesthesia), balanced general anaesthesia with a laryngeal mask (LMA) was arranged.

Premedication was with intravenous Midazolam 2mg. In the operating room: monitoring vital parameters, induction of general anaesthesia with Fentanyl 200mcg and Propofol 240mg, LMA number 4 placement. The patient's clinic suggested rapid pharmacological metabolism, especially of opioids. Maintenance of anaesthesia was with Sevorane (MAC1) and Fentanyl 300mcg; antibiotic prophylaxis with Cefazolin 2g and antiemetic with Dexamethasone 4mg; ventilation on PSV. Towards the end of the surgery, Paracetamol 1g, Contramal 100mg, Ketorolac 30mg, and Ondansetron 4mg were administered.

The procedure lasted about half an hour, and recovery from anaesthesia was uneventful. However, upon regaining consciousness, the patient was highly agitated and complained of intense pain (NRS 9) at the surgery site. Midazolam 1mg and Clonidine 50mcg intravenously were administered with little benefit.

Due to the persistence of pain symptomatology, despite substantial therapy performed (Fentanyl500mcg+Paracetamol1g+Contramal100mg+Ketorolac30mg+Clonidine50mcg+Midazolam1mg+Toradol30 mg), refractory boluses of Morphine totalling 6mg were performed in about an hour and ice was placed in loco. The return of symptomatology occurred about an hour from the end of the operation. For postoperative pain management was applied, over the next 48 hours, an elastomeric containing Ketorolac 90mg, Contramal 300mg, Ondansetron 8mg and saline solution at 50ml setting the infusion at 2 mL/h over 24 hours. Postoperatively, pain control was optimal.

In conclusion, standard procedures are found to be poorly effective for the same intervention in fibromyalgia patients. In particular, an increased need for opioids was observed. Given the current lack of knowledge, there is a need to effectively create new protocols to manage acute pain in this category of patients.

Informed consent was obtained

### A27. Total intravenous anesthesia (TIVA) VS. balanced anesthesia in acute kidney injury associated to surgery: a prospective observational study

#### Bussolati E., Palmieri M., Ragazzi R., La Rosa R., Verri M., Volta C.A., Scaramuzzo G., Spadaro S.

##### Intensive Care Unit, Department of Translational Medicine, University of Ferrara ~ Ferrara ~ Italy

###### **Correspondence:** Bussolati E.

BACKGROUND: Acute kidney injury (AKI) is an adverse event frequently associated to surgery. It is mainly related to the type of surgery, reduction in renal blood flow, intraoperative hypotension and use of nephrotoxic drugs. Anesthesia’s role in the AKI development is still debated. The potential nephrotoxicity due to Compound A present in Sevoflurane has been studied for a long time with contrasting results 1,2. The aim of this study is to evaluate the role of general anesthesia’s techniques (TIVA vs. balanced) on the development of surgical-associated AKI in patients undergoing major surgery.

MATERIALS AND METHODS: This is a single-center prospective cohort study. We included patients ≥18 years old undergoing major surgery under general anesthesia lasting ≥2h and excluded patients with a preexistent history of AKI, kidney transplantation or under renal replacement therapy. Demographic and clinical-anamnestic data were collected, in addition to surgical data, anesthetic technique and data regarding the development of AKI in the first 72 h following surgery, as defined by the KDIGO criteria (increase in serum Cr ≥ 0.3mg/dl in 48h or urinary output ≤0.5ml/kg/h for at least 6 hours) 3. Qualitative variables were expressed as number (%), quantitative variable as mean ± SD or median (IQR) based on the distribution of data, verified by the Shapiro-Walk test. Parametric (T-test) and non-parametric (Pearson’s chi-square test) tests were used to compare the two group (AKI/no AKI), and p values <0.05 were considered significative. A multivariate logistic regression was used to evaluate the risk factors independently associated to AKI.

RESULTS: We enrolled 131 patients undergoing general (49.6%), vascular (25.2%), thoracic (13%), urological (1.5%) and gynecological (0.8%) surgery. Of them, 44/131 (33.6%) patients developed postoperative AKI; 36/44 of them (81.8%) received balanced general anesthesia with halogenated anesthetic and 8/44 (18.2%) received TIVA (p = 0.004). The multivariate logistic regression analysis (Table 1) showed that age (p = 0.017), duration of surgery (p = 0.046) and the balanced general anesthesia (p = 0.02, RR = 3.05) were independently associated to the development of postoperative AKI.

CONCLUSIONS: Balanced general anesthesia (halogenated + intravenous) is independently associated with an increased risk of developing acute kidney injury in patients undergoing major surgery.

BIBLIOGRAPHY:

1. Bang, J. Y., Lee, J., Oh, J., Song, J. G. & Hwang, G. S. The Influence of Propofol and Sevoflurane on Acute Kidney Injury After Colorectal Surgery: A Retrospective Cohort Study. Anesth Analg 123, 363–370 (2016).

2. Fukazawa, K. & Lee, H. T. Volatile Anesthetics and AKI: Risks, Mechanisms, and a Potential Therapeutic Window. J Am Soc Nephrol 25, 884 (2014).

3. Kidney Disease: Improving Global Outcomes (KDIGO). Acute Kidney Injury Work Group. KDIGO clinical practice guidelines for acute kidney injury. Kidney Int Suppl 2:1 (2012).

**Table 1 (abstract A27).** Risk factors associated to postoperative AKI

Variable	Relative Risk	*P value*
Age (years)	1.05	***0.017***
Weight (kg)	0.99	0.83
BMI (kg/m^2^)	1.07	0.37
Intraoperative fluid balance (ml)	1	0.51
Balanced general anesthesia	3.05	***0.025***
Duration (min)	1	***0.046***
Colloid infused (ml)	1	0.80
Haemocomponents infused (ml)	1	0.76

Risk factors associated to postoperative acute kidney injury. *BMI* body mass index. *P value* considered significative if < 0.05

## Anestesia loco-regionale

### A28. External oblique intercostal block combined with tap block ensures complete abdominal analgesia for abdominoplasty surgery: completing the puzzle

#### Ruggiero A.^2^, Costa F.^1^, Strumia A.^2^, Remore L.M.^1^, Pascarella G.^2^, Sarubbi D.^2^, Longo F.^2^, Tenna S.^3^, Cataldo R.^1^, Agrò F.E.^2^

##### ^1^Unit of Anaesthesia, Intensive Care and Pain Management, Department of Medicine, Fondazione Policlinico Universitario Campus Bio-Medico , via Álvaro del Portillo 21, 00128 Rome, Italy ~ Roma ~ Italia, ^2^Unit of Anaesthesia, Intensive Care and Pain Management, Department of Medicine, Fondazione Policlinico Universitario Campus Bio-Medico ~ Roma ~ Italia, ^[3]^Università Campus Bio-Medico, Plastic, Reconstructive and aesthetic surgery ~ Roma ~ Italia

###### **Correspondence:** Ruggiero A.

Background

Abdominoplasty surgery is usually performed under balanced general anesthesia (GA). In abdominal surgery, lateral tap block has been used as intra- and post-operative pain control, even if this technique does not successfully cover all of the belly region.

Recent studies have demonstrated the continuity between the thorax and abdomen fascial planes, giving the external oblique intercostal block great expectation in analgesia of the upper abdominal wall.1

Methods

Written informed consent was collected from a 60-year-old female, candidate for abdominoplasty surgery. She had previously undergone a bioenterics intragastric balloon and had no other comorbidities. The external oblique intercostal block was performed, using a linear probe positioned between the midclavicular and anterior axillary lines, at the level of the sixth rib. The transducer was then rotated to obtain a paramedian sagittal oblique view. 15 mL of 0,5% ropivacaine was injected between the plane of the external oblique and the intercostal muscles bilateral. Transversus abdominis plane block with posterior access was then performed to ensure complete analgesia of the lower segments as well with 15 mL of 0,5% ropivacaine bilateral. Then GA with a laryngeal mask was performed.

Results

The patient showed stable vital signs throughout the procedure. In the recovery room, NRS was zero. The following day, she reported a score of 3 on a scale from 1 to 10. No additional opiates were needed.

Conclusion:

The subcostal external oblique plane block has proved to be an excellent technique for peri-postoperative pain control for upper abdominal segments.

References

1. Elsharkawy H, Kolli S, Soliman LM, Seif J, Drake RL, Mariano ER, El-Boghdadly K. The External Oblique Intercostal Block: Anatomic Evaluation and Case Series. Pain Med. 2021 Nov 26;22(11):2436-2442. DOI: 10.1093/pm/pnab296. PMID: 34626112.

**Fig. 1 (abstract A28).**
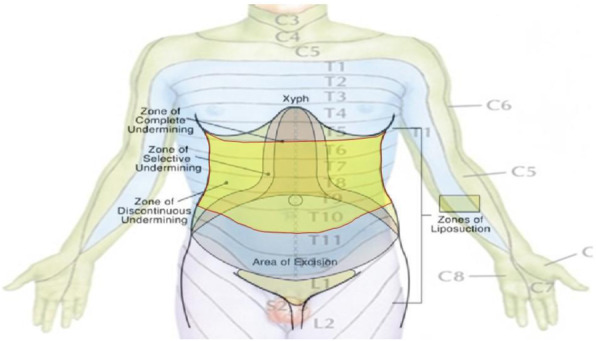
See text for description

### A29. ESP-block and general anesthesia in a patient undergoing spine surgery for hyperkyphosis D5-L3: case report

#### Borrelli G.^1^, Maria Luisa D.P.^1^, De Divitiis D.^1^, Palmieri F.B.^1^, Galdo V.^1^, Calabrò G.^2^

##### ^1^U.O.C. Anestesia e Rianimazione P.O. San Francesco d'Assisi- ASL Salerno ~ Oliveto Citra ~ Italia, ^2^U.O.C. Ortopedia e Traumatologia P.O. San Francesco d'Assisi- ASL Salerno ~ Oliveto Citra ~ Italia

###### **Correspondence:** Borrelli G.

Background

Hyperkyphosis is the condition in which the natural convex curvature of the spine is accentuated. Treatment varies according to the causes that generate it; surgery is indicated when: the curvature exceeds 80 ° Cobb; worsening and disabling pain occurs; the spine is unstable and the progression is constant and fast.

Case report

Patient B.B., female, 71 years old, 68 kg, 168 cm. In medical history: arterial hypertension, ischemic heart disease (hypoperfusion of the anterior wall and stenosis of 50% of the LADA) and anxiety-depressive syndrome. She is admitted to the Orthopedics Department for degenerative dorsal hyperkyphosis and L2 fracture with acupressure pain at the level of the spinous processes and paravetebral dorsolumbar region with positive Lasegue sign on the right and left, difficulty in assuming prone position, DTR AAII bilaterally hypoelicitable, predominantly hyposthenia on the left with reduction of motility. Therefore, she is a candidate for L3-D5 posterior vertebral arthrodesis and stabilization surgery with pedicle screws.

In pre-op room midazolam 2mg i.v. and PONV prophylaxis (pantoprazole 40mg i.v, metoclpramide 10mg i.v.) is administered in peripheral venous access (20G) and a second PVC is inserted (18G).

In the operating room patient is monitored with: ECG, SpO2, NIBP, BIS / Entropy, TOF.

Induction: fentanyl 3mcg/kg, propofol 2mg/kg, rocuronium 0.6mg/kg. At TOF 0 we proceed to orotracheal intubation with 7.5 i.d. armored ETT. Mechanical ventilation in VC-Autoflow mode with Vt 460ml, PEEP 5cmH2O, RR 14 acts/min, I: E = 1: 1.7. The patient is placed prone on the operating table. Maintenance: desflurane (MAC between 0.8 and 1) and remifentanil in TCI (Minto, Ce) and rocuronium boluses equal to 15% of the initial dose at T3 of TOF.

We proceed to bilateral ESP-Block at L3-level with ultrasound-guided technique (convex probe) and atraumatic needle 100mm with caudo-cranial direction, 25ml per side of ropivacaine 0.5% are administered for the block, following the diffusion of the anesthetic.

Before cutaneous incision (carried out 23 min after the block), paracetamol 1g i.v and ketorolac 30mg i.v..

The remifentanil infusion, started with a Ce of 3ng/ml, after about 10min it is reduced to 1.5ng/ml and after 1h it is reduced to 1 ng/ml until it is interrupted at the beginning of the closing phase and begins TAPO with elastomer (2ml/h for 24h) with: ketorolac 90mg, clonidine 150mcg and metoclopramide 10mg. To reverse neuro-muscular blockade, sugammadex 2mg / kg was used.

Total surgery duration: 4h40min. Total anesthesia duration: 5h15min. Time of complete awakening after returning to the supine position: 8min. There was only one episode of significant and hypotension (quickly resolved). After awakening, patient patient was kept in recovery room for 30min and discharged to the ward with spontaneous breathing (FiO2 21%), stable and NRS 3. In the first 24 hours paracetamol 1g i.v. every 6h; then every 8h the next day, finally as needed.

Informed consent has been obtained.

Conclusion

The ESP-block for spine surgery is a valid tool for pain control during and after surgery, as part of a multimodal strategy aimed at minimizing opioid consumption and to allow fast track surgery.

### A30. US-guided pudendal nerve block (USG-PNB) as a sole anesthetic tecnique in patients undergoing hemorroidectomy: a preliminary experience

#### Corso R.M., Aiello L., Maitan S.

##### Anestesia e Rianimazione, Dipartimento Chirurgico, Ospedale GB Morgagni-L. Pierantoni ~ Forlì ~ Italia

###### **Correspondence:** Aiello L.

Background:

Hemorrhoidectomy is a very common surgical procedure, however burdened by a very significant postoperative pain[1]. General or spinal anesthesia using intrathecal morphine is widely used. However there is no strong evidence of the best anesthetic technique[2].

Materials and methods:

Ten consecutive patients scheduled for hemorrhoidectomy were enrolled. After ASA standard monitoring, patients were positioned in the lithotomy position and sedation with propofol TCI started under BIS® monitoring. Then, USG-PNB was perfomed bilaterally using 21gauge 85mm (Echoplex, Vygon) nerve stimulator needle under ultrasound guide. On each side, under aseptic condition, Ropivacaine at the dosage of 0.5%, 20ml was administered. Postoperative scheduled analgesia consisted of Acetominphne 1000mg iv with Tramadol 100mg as rescue analgesia. NRS was used to assess the intensity of pain which was measured between 6 and 24 h postoperatively.

Results:

Postoperatively, all patients had an NRS of less than 4 both at 6 and 24 hours and none needed rescue analgesia. All patients were discharged on postoperative day.

Conclusion:

In times of cost containment and resource optimization, the optimal management of day surgery is fundamental. Hemorrhoidectomy is typically performed in a day-surgery clinic, however suboptimal management of postoperative pain is common leading to increased length of stay and opioid consumption[3]. The pudendal nerve block has already showed a benefit in terms of postoperative analgesia when added to spinal anesthesia[4], but conflicting results have been reported when used alone. Literature data showed the effectiveness of guided US-block compared to finger guided transvaginal pudendal nerve block[5]. In our experience, the use of USG-PNB has allowed optimal pain control without the use of opioids, achieving the goal of day surgery. The technique appears more cost effective than spinal or general anesthesia, avoiding the risks associated with subarachnoid anesthesia and opioids.

References:

[1] Medina-Gallardo A, Curbelo-Peña Y, De Castro X, Roura-Poch P, Roca-Closa J, De Caralt-Mestres E. Is the severe pain after Milligan-Morgan hemorrhoidectomy still currently remaining a major postoperative problem despite being one of the oldest surgical techniques described? A case series of 117 consecutive patients. Int J Surg Case Rep. 2017;30:73-75.

[2] Sammour T, Barazanchi AW, Hill AG; PROSPECT group (Collaborators). Evidence-Based Management of Pain After Excisional Haemorrhoidectomy Surgery: A PROSPECT Review Update. World J Surg. 2017;41(2):603-614.

[3] Lu PW, Fields AC, Andriotti T, Welten VM, Rojas-Alexandre M, Koehlmoos TP, Schoenfeld AJ, Melnitchouk N. Opioid Prescriptions After Hemorrhoidectomy. Dis Colon Rectum. 2020;63(8):1118-1126.

[4] Di Giuseppe M, Saporito A, La Regina D, Tasciotti E, Ghielmini E, Vannelli A, Pini R, Mongelli F. Ultrasound-guided pudendal nerve block in patients undergoing open hemorrhoidectomy: a double-blind randomized controlled trial.Int J Colorectal Dis. 2020;35(9):1741-1747

[5] Naja Z, El-Rajab M, Al-Tannir M, Ziade F, Zbibo R, Oweidat M, Lönnqvist PA. Nerve stimulator guided pudendal nerve blockversusgeneral anesthesia forhemorrhoidectomy. Can J Anaesth. 2006 Jun;53(6):579-85

### A31. Thymic resection via median sternotomy: ultrasound-guided bilateral parasternal block could be effective?

#### Pagani G.^1^, Giordano C.^2^, Brambillasca P.^3^, Perletti S.^1^, Gera E.^1^, Mulas E.^2^, Rasella B.^2^, Cadei M.^2^, Mario C.^2^

##### ^1^Resident of Anesthesia and Intensive Care, Department of Medical-Surgical, Radiological Sciences and Public Health, University of Brescia, Brescia, Italy ~ Brescia ~ Italia, ^2^Anesthesia and Intensive Care Unit, Emergency and Critical Care Department, ASST Papa Giovanni XXIII, Bergamo, Italy ~ Bergamo ~ Italia, ^3^Anesthesia and Intensive Care Unit, Emergency and Critical Care Department, Fondazione IRCCS Cà Grande Ospedale Maggiore Policlinico, Milano, Italy. ~ Milano ~ Italia

###### **Correspondence:** Pagani G.

A 30-year-old male presented ataxia, nystagmus, blurred vision, right-ear hearing loss and a positive Romberg test with 10 kg weight loss.

Anti-HU paraneoplastic syndrome was suspected and intravenous immunoglobulin started with slight improvement of neurological symptoms.

Total body PET scan was performed which showed hyperfixation area in the anterior-superior mediastinum, indicative of thymoma. After a written informed consent was obtained, the patient underwent thymectomy via median sternotomy. Impaired pulmonary function which can lead to pulmonary infection and atelectasis due to severe postoperative pain is shown in the literature.

We examined the efficacy of ultrasound-guided parasternal block (Us-PsB), a novel regional anesthetic technique, in preventing these complications, proved to deliver analgesia to the antero-medial chest wall, by blocking the anterior cutaneous branch of the intercostal nerves and consequently ventilation improvement.

After induction of general anesthesia, with the patient in the supine position, we performed the Us-PsB using a high-frequency linear transducer (12-MHz).

The probe was placed longitudinally 2 cm lateral to the sternal border, visualizing the 2nd intercostal space, Pectoral Major muscle(PMM), External Intercostal muscle (eiM), the second rib and the pleura. The in-plane approach was applied with a 22-gauge, 100-mm needle. A solution of 30 ml Ropivacaine 0,2% and, as adjuvant, dexmedetomidine75 mcg and dexamethasone 4 mg were injected into the interfascial plane between PMM and eiM at the level of the 2nd intercostal space bilaterally.

Anesthesia was maintained with desflurane 0.9 MAC and intraoperative hemodynamic stability was observed.

Before the end of surgery, intravenous injection of acetaminophen 1 gram and ketorolac 30 mg were administered.

After surgery and extuation, hemodynamic stability was maintained and the patient reported prolonged pain relief. Pain score(Numeric Rating Score -NRS- scale of 0-10 where 0=no pain and 10=maximum pain) was used to assess postoperative pain. Postoperative analgesia consisted of acetaminophen 1 gram and ketorolac 30 mg,both every 8 hours in the first 24 hours, while no rescue therapy was needed.

In the first 48 postoperative hours, the patient reported no pain at rest(NRS 0/10) with slight pain on movement (NRS 2/10). No discomfort, neurological symptoms or other complications were recorded in the postoperative period.

Us-Psb and the co-administration of local anesthetic with dexmedetomidine and dexamethasone,as adjuvants, in this particular case for a thymic resection via median sternotomy provided adequate and prolonged analgesia for up to 48 hours with no side effects.

US-PSb and the use of dexmedetomidine and dexamethasone as adjuvants appeared to be an effective approach for post-operative analgesia in this surgery, but future studies will be needed.

### A32. Ultrasound-guided quadratus lumborum block type II vs erector spinae plane block on postoperative pain and opioid consumption after laparoscopic adrenalectomy

#### Perletti S.^1^, Brambillasca P.^2^, Consuelo M.^2^, Personeni N.^2^, Gera E.^2^, Cadei M.^2^, Mulas E.^2^, De Gaetano P.^2^, Pagani G.^2^, Rasella B.^2^, Giordano C.^2^

##### ^[1]^Resident of Anesthesia and Intensive Care, Department of Medical-Surgical, Radiological Sciences and Public Health, University of Brescia ~ Brescia ~ Italia, ^[2]^Anesthesia and Intensive Care Unit, Emergency and Critical Care Department, Fondazione IRCCS Cà Grande Ospedale Maggiore Policlinico ~ Milano ~ Italia

###### **Correspondence:** Perletti S.

Ultrasound guided Quadratus Lumborum Block type II (QLB II) and Erector Spinae Plane Block (ESPB) are novel techniques which prove to be effective for postoperative analgesia in patients undergoing laparoscopic abdominal surgery.

We evaluated analgesic effectiveness of these interfacial plane blocks in two patients scheduled to undergo right laparoscopic adrenalectomy for adrenal tumor, evaluating postoperative pain and cumulative opioid requirement at 24th hours.

Written informed consent was obtained. The first patient was a 66 years old male with hypertension and chronic obstructive lung disease. The second, a 67 years old male with hypertension and insulin-dependent diabetes. We induced general anesthesia with fentanyl 1.5 mcg/Kg, propofol 2 mg/Kg and rocuronium 0.6 mg/Kg in both patients. After induction, we proceed with loco-regional techniques.

In case 1, ultrasound guided QLBII was performed with the patient in supine position. A curvilinear ultrasound probe was placed cephalad and parallel to the iliac crest and moved posteriorly until Quadratus Lumborum muscle was clearly identified. A 100 mm Pajunk needle was inserted in the plane with lateral to medial direction. We injected 20 ml of 0,325% Ropivacaine and 4 mg of dexamethasone, as an adjuvant, bilaterally in the fascial plane between the quadratus lumborum muscle and the latissimus dorsi muscles.

In case 2, we performed ultrasound guided ESPB. The patient was in the left lateral position for the surgery. The right T12 transverse process was identified using a high-frequency linear probe. A 100 mm Pajunk needle in-plane was inserted and 25 ml of 0.325 % ropivacaine and 4 mg of dexamethasone, as an adjuvant, were injected in the deep plane of the erector spinae muscle.

Maintenance of anesthesia was achieved with sevoflurane (0,8 MAC). Acetaminophen 1 gram intravenous was administered before patients awakening in the operating room.

In the postoperative period, we reported pain intensity using the NRS scale at 24th hour after surgery and the opioid requirements. In case 1, analgesia was provided with acetaminophen 1 g 8 hourly, ketorolac 30 mg 8 hourly and tramadol 100 mg 12 hourly with NRS >3.

Patient in case 2 had prolonged pain relief (NRS < 3) and required 3 g of acetaminophen and ketorolac 90 mg, sparing opioid use.

No complications were recorded in the postoperative period in both patients. In our case reports the patient receiving ESPB had a decreased postoperative opioid consumption in the first 24 hours and reduced NRS scores compared to QLBII.

In literature there is no case report comparing ESP and QLBII in this surgery. The excellent control of somatic and visceral pain obtained with ESPB is likely due to the spread of local anesthetic that, despite it being unpredictable and not guaranteed, is focused on a specific level of thoracic paravertebral space. However, the comparison of ESPB and QLB-II in this surgery is still a topic to be evaluated by further studies in order to confirm whether ESPB could be more effective than QLBII for laparoscopic adrenalectomy.

### A33. Top/Subcostal QLB: a top block for top pain control in a laparoscopic nephrectomy

#### Remore L.M., Costa F., Strumia A., Cataldo R., Pascarella G., Di Folco M., Ruggiero A., Sarubbi D., Longo F., Agrò F.E.

##### Unit of Anaesthesia, Intensive Care and Pain Management, Department of Medicine, Fondazione Policlinico Universitario Campus Bio-Medico ~ Roma ~ Italia

###### **Correspondence:** Remore L.M.

Background and aims

Quadratus Lumborum Block (QLB) is based on the injection of local anesthetic (LA) into the toracolumbar fascia (TLF) surrounding the quadratus lumborum muscle. The spread of LA along the TLF into the thoracic paravertebral space and transversalis fascia provides analgesia without neuraxial-associated hypotension; this is mostly effective for patients undergoing abdominal and hip surgeries. There are four types of QLB based on injection sites: lateral, posterior, transmuscular and intramuscular. Transmuscular QLB, or QLB3, can be performed at L4 and L2 level using the subcostal approach. Our aim is to assess the efficacy and viability of the subcostal QLB approach for laparoscopic nephrectomy.

Methods

We selected a patient listed for a laparoscopic nephrectomy, male, ASA score II, BMI 27. After obtaining consent we performed bilateral QLB with subcostal approach. We injected a solution of 30ml ropivacaine 0,37 mg/ml for each block (a total of 225 mg ropivacaine) to ensure optimal spread and proper anesthetic concentration. A bolus of 100 μg fentanyl was the only opioid administered for general anesthesia induction. We used BIS and NOL Pain Response Monitor during surgery to assess the need of further opioids administration. The primary outcome was the consumption of opioids in the first 24h after surgery. The secondary outcome measured were heart rate and median arterial pressure during the surgery, intraoperative consumption of remifentanil, VAS score at 1, 3 and 6 hours after surgery, Bruggeman comfort scale (BCS) at 12 and 24 hours and presence of side effects.

Results

No opioid administration was needed during surgery, VAS scores were never over 2, BCS always between 3 and 4 and no side effects were observed. We prescribed an analgesic therapy comprised of paracetamol 1g tid and ketorolac 30mg tid in the post-operative period. No other painkillers or opiates were administered.

Conclusion

This regional anesthesia strategy seems to be promising and satisfactory, providing optimal post-operative analgesia and easier intra-operative management. Studies are needed to implement this strategy and compare its outcomes to traditional general anesthesia.

**Fig. 1 (abstract A33).**
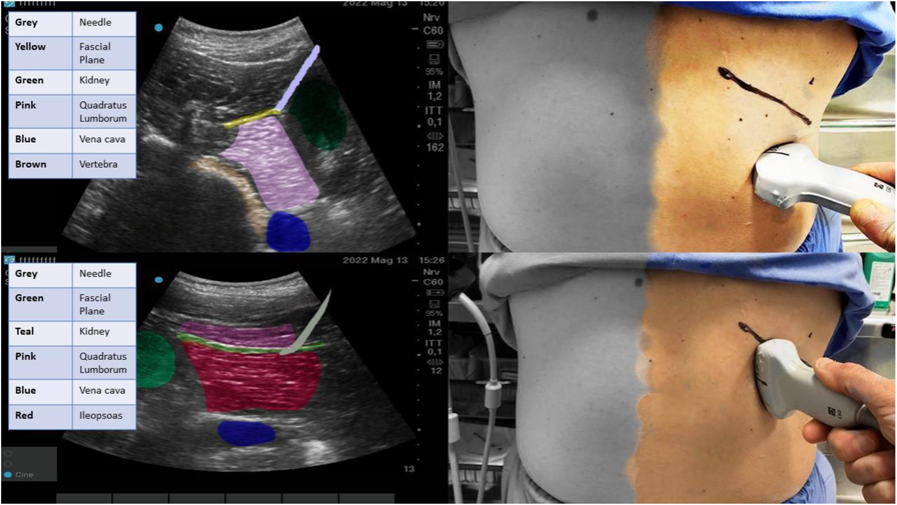
See text for description

### A34. Sedative and hemodynamic effects of target-controlled infusion of propofol and dexmedetomidine using ultrasound-guided regional anesthetic technique for mastectomy and axillary dissection

#### De Gaetano P.^2^, Perletti S.^2^, Personeni N.^2^, Pagani G.^2^, Gera E.^2^, Giordano C.^1^, Mulas E.^1^, Rasella B.^1^, Cadei M.^1^, Brambillasca P.^3^, Mario C.^4^

##### ^1^Anesthesia and Intensive Care Unit, Emergency and Critical Care Department, ASST Papa Giovanni XXIII ~ Bergamo ~ Italia, ^2^Resident of Anesthesia and Intensive Care, Department of Medical-Surgical, Radiological Sciences and Public Health, University of Brescia ~ Brescia ~ Italia, ^3^Anesthesia and Intensive Care,Emergency and Critical Care Department, Fondazione IRCCS Cà Grande Ospedale Maggiore Policlinico ~ Milano ~ Italia, ^4^Director of Anesthesia and Intensive Care Unit 1, Emergency and Critical Care Department, ASST Papa Giovanni XXIII ~ Bergamo ~ Italia

###### **Correspondence:** De Gaetano P.

It has recently been demonstrated that general anesthesia in breast surgery could promote metastasis of cancer cells and, moreover, determines peri-operative complications. Therefore, the identification of different anesthesiological approaches to breast surgery could be relevant. We introduced in our clinical practice a standardized sedation protocol. Intra-operative a highly selective alpha-2 adrenergic agonist, dexmedetomidine(DEX), was used and a load of 1 μg/kg/h in 15 minutes, followed by a continous infusion of 0,6 - 0,3 mcg/kg/h, was administered. In association, intravenous sedation using target controlled infusion(TCI) of propofol with Schnider pharmacokinetic model was started. The effect-site target concentration of propofol was set and was maintained at 1.5 μg/ml. Before surgery, we performed the ultrasound Serratus Plane Block(US-SPB), with injection of 20 ml of 0,75% ropivacaine and 0,5 mcg/kg of DEX in the fascial plane deep of the serratus anterior muscles at the fifth rib level and, subsequently, ultrasound- Pectoralis nerve(US- PECS 1), injecting 10 ml of 0,75% ropivacaine and 0,5 mcg/kg of DEX in the fascial plane between the pectoralis major and minor muscles at the third rib level. We observed the sedative and hemodynamic effects and peri-operative complications in 7 patients with the mean age of 65 years, severe obesity, affected by anxious depressive syndrome or psychiatric illness and other diseases, ASA III (Imagine 1), undergoing radical mastectomy with axillary dissection from June to August 2021. During surgery, oxygen saturation, respiratory rate, end-tidal carbon dioxide (EtCO_2_) monitoring, non-invasive blood pressure and consciousness with Bispectral index- BIS, used to monitor depth of anesthesia (ranges from 0 -equivalent to EEG silence- to 100 -patient conscious), were continuously measured and evaluated. After surgery, a quick awakening was recorded and the patients were transferred to a recovery room where blood pressure and oxygen saturation were continuously monitored for at least 30 minutes until meeting discharge criteria. In the first 24 hours prolonged pain relief, Numeric Rating Scale-NRS 2 (0 indicates no pain and 10 indicates the worst pain), no anxiety and post-operative complications were observed (Image 2). Only 3 g of acetaminophen were administered and shorter recovery time were reported. They were discharged on the following day after surgery.

Nor hypercarbia neither hypoxia or apnea events and hemodynamic stability, no pain, excellent comfort and no peri-postoperative complications were reported.

We believe that this sedation protocol could be an effective method that doesn’t cause excessive plasma concentration of both sedatives drugs, no respiratory depression, no anxiety and hypotension. This drug combination ensure intra-operative spontaneous breathing and hemodynamic stability with excellent comfort for the patients, without peri-operative complications. Further studies are necessary to externally validate findings.

**Table 1 (abstract A34).**
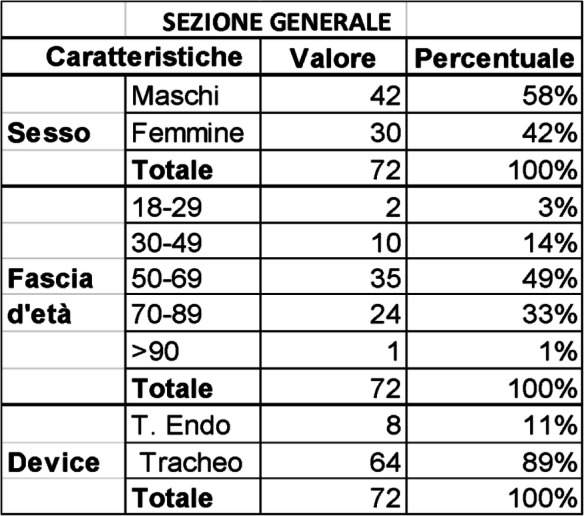
Patients characteristics and comorbidity

**Table 2 (abstract A34).**
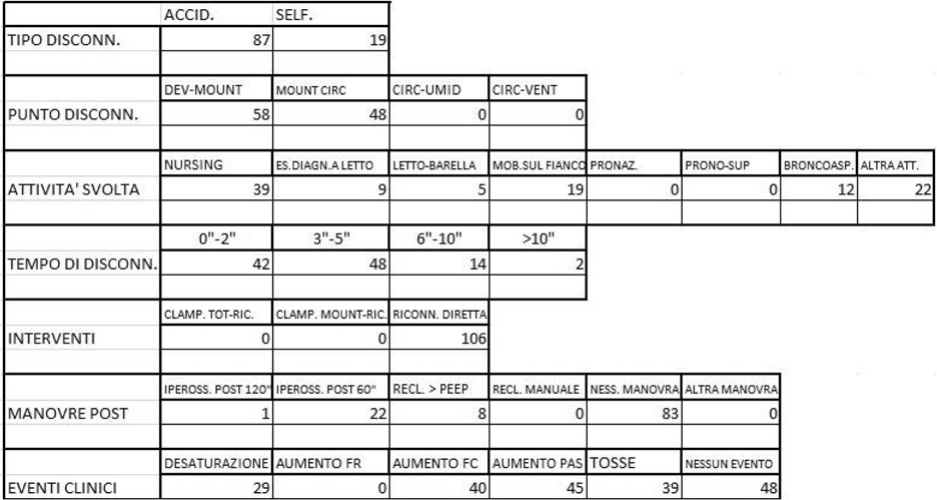
Results of sedation associated to ultrasound-guided thoracic fascial plane block

### A35. Fascial plane blocks for intra and post operative analgesia after breast augmentation vs general anesthesia: a randomized clinical trial

#### D'Errico C.^1^, Sabatella E.^1^, Frangiosa A.^1^, Belfiore F.^1^, Di Costanzo M.^2^

##### ^1^Dipartimento di Anestesia e Rianimazione, AORN Cardarelli ~ Napoli ~ Italia, ^2^AOU L. Vanvitelli ~ Napoli ~ Italia

###### **Correspondence:** D'Errico C.

Background and objectives: We chose PECs I and ESP block as fascial block in breast augmentation surgery with implant of subpectoral protheses, one of the most common surgical procedures in this field.

Methods: We selected 20 patients ASA I-II undergoing breast augmentation for aesthetic purposes. They were randomly divided in 2 groups: the general anesthesia group (GA group, n=8) and the fascial block group (FB group, n= 12). Both groups were premeditated with midazolam 5mg IV. The GA group received general anesthesia with: propofol IV (2mg/kg IBW), rocuronium bromide IV (0,6mg/kg IBW), remifentanil in continuous IV administration (between 0,15-0,25mcg/kg IBW/min). Endotracheal intubation was performed with armed cuffed tube (for women with a diameter between 7.0 - 8.0 mm and for men between 7.5 - 8.5 mm) with mechanical ventilation using SpO2 >97% as an endpoint and sevoflurane tritrated with BiSpectral Index (BIS) values. The FB group received Erector Spinae Block bilateral with injection of 20 ml of 0.75% ropivacaine for each side with a 22G 70mm needle, paraspinal level T5-T6. To reach an appropriate intraoperative analgesic regimen with the insertion of subpectoral prostheses, PECS I block was added injecting 10 ml of 0.75% ropivacaine for each side into the fascial area between the Pectoralis Major muscles (PMm) and Pectoralis minor muscles (Pmm) with a 22G 70mm needle. The patients of the FB group were in spontaneous breathing, in case of SpO2 <94% we complemented ventilation with a LMA insertion previous administration of propofol 1- 2mg/kg IBW. In case of incomplete block coverage (NRS>6) fentanyl IV between 0,5 and 1 mcg/kg IBW was given.

In the operating room, all patients received continuous standard monitoring (PA, FC, ECG with at least 2 leads displayed, SpO2) and BIS™ monitor. Post operative pain was evaluated with NRS and its management was performed for the GA group with Acetaminophen 1g IV TID and continuous infusion of Contramal 400mg and Ondasentron 8mg in the 24h following the surgery. A rescue therapy was planned for the GA group with Toradol IV 30mg if NRS> 4.

Results and discussion: We assessed post operative pain with NRS scale at 1, 2, 6, 12 and 24 hours intervals. During surgery the hemodynamic parameter of both groups were similar. In the 24h following the surgeries for the FB group a rescue analgesia was administered only to 2 patients and opioid drugs weren’t required. In the GA group, despite the continuous infusion, opioid were needed, thus confirming that the analgesic duration and stability of a Fascial Plane Block technique was longer. The rates of nausea and vomiting were higher in GA group than in IFB group. No block-related complications were recorded.

Conclusions: Fascial nerve blocks provide effective perioperative pain relief after aesthetic breast surgery and is associated with reduced need for analgesic drugs and post operative pain, reduction of PONV episodes with a safety profile regarding block-related complication.

Combined use of ESP and PECS I provide superior postoperative analgesia in patients undergoing breast augmentation with insertion of subpectoral prostheses and shortens hospital stay.

**Fig. 1 (abstract A35).**
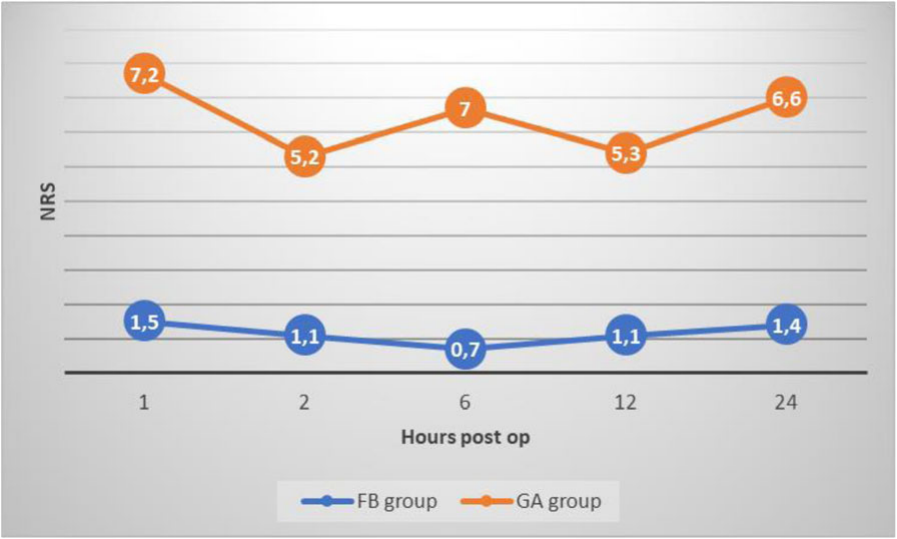
See text for description

### A36. Observational preliminary study: use of dexmedetomidine in patients undergoing orthopedic surgery of upper limb in loco-regional anesthesia

#### Zumpano A., Caruso M.T., Tangari R., Bonadio F., Monardo A.

##### Ospedale Giovanni Paolo II, dipartimento di Anestesia e Rianimazione ~ Lamezia Terme ~ Italia

###### **Correspondence:** Zumpano A.

Background. Dexmedetomidine is a very selective agonist of alpha2-receptors, used in continous infusion. Sedative effects are mediated by inhibition of the activity of Locus Coeruleus, which is the main noradrenergic centre, located in the brainstem. Administered in monotherapy dexmedetomidine hasn’t effect on respiratory drive and this make the drug extremely safe in the sedation of patients undergoing surgery in loco-regional anesthesia.

Outcomes. Main outcome an assessment of dexmedetomidine sedative effects and its hemodynamic stability in patients undergoing orthopedic surgery during loco-regional anestesia. The secondary outcome of the study was the evaluation of a possible use of non-invasive specific monitoring of sedation level to determine the most appropriate infusion dosage of dexmedetomidine.

Patients and methods. The enrollment period considered was October 2021-February 2022. 10 patients were included in the study, all adults candidates for orthopedic surgery of the upper limb performed in loco-regional anesthesia with an ultrasound-guided peripheral nerve block of brachial plexus.

The non-invasive monitoring system Conox® were used for the evaluation of sedation level. The study protocol involved the application of monitoring immediatly in the pre-op room. Dexmedetomidine infusion was started using a dedicated venous line and a syringe-pump. The start dosage was 0,7mcg/kg/h and the sedation target considered was qCON 80-70, so the infusion dosage was modulated on the target qCON value. Local anestetic used were: Levobupivacaine 0,5% and Lidocaine 1% for a total volume of 20ml.

Conox® monitoring was maintained in place for the assessment of the timing of recovery of basal values. NRS (Numerical Rating Scale) was used for evaluation of analgesia. Administration of Paracetamol 1g or Diclofenac 75mg it was expected in NRS >3.

Results. Patients didn’t report disconfort during their stay in pre-op room, while performing the peripheral block, during surgery and in recovery room. Severe bradycardia was observed in 60% of cases and has been resolved giving atropine 0,5mg iv. Variations in blood pressure were not observed. Episodes of desaturation were observed in 40% of cases, in particular were patients affected by moderate obesity ad OSAS. In follow-up period patients required Paracetamol 1 g iv or Diclofenac 75mg iv only after 14-24hrs for NRS between 4-7; NRS >3 was observed in the first 12hrs after surgery.

Conclusion. Analyzing the collected data we can claim that a sedation with continuous infusion of dexmedetomidine could be useful in support of the techniques of loco-regional anesthesia for interventions of medium-long duration. The timing of reaching the target value of qCON does not make its use advantageous for surgery of short duration. We can also conclude that monitoring of level of sedation used has allowed us to modulate accurately the dosage of the infusion, minimizing side effects and ensuring adequate sedation during all procedures.

### A37. Intrathecal morphine associated to tap block in laparoscopic robot-assisted resection of gastrointestinal stromal tumors (LRA-GIST): our experience

#### Aiello L.^1^, Gori A.^1^, Ione E.^2^, Corso R.M.^1^, Maitan S.^1^

##### ^1^Anestesia e Rianimazione, Dipartimento Chirurgico, Ospedale GB Morgagni-L. Pierantoni ~ Forlì ~ Italia, ^2^Department of Morphology, Surgery and Experimental Medicine, Section of Anesthesia and Intensive Care, University of Ferrara ~ Ferrara ~ Italia

###### **Correspondence:** Aiello L.

Background:

GISTs are less aggressive than tumors elsewhere, especially those in small intestine[1]. Literature report feasibility and benefits of minimally invasive surgery, in terms of outcome and low mortality and morbidity rates[2]. Robotic surgery, provides favorable peri-operative results compared to open and laparoscopic surgery[3].

Materials and methods:

Prospective observational study of ten consecutives patients scheduled for LRa-GIST; the average age of the patients was 68±2 years and they did not show substantial differences in anthropometric characteristics. Patient informed consent was obtained one week prior to surgery. All patients underwent standard monitoring. Before general anesthesia, patients received spinal analgesia with 100mcg of morphine and Levobupivacaine 7,5mg in total volume of 3ml in saline solution at the T9–T10 level, using a 25 Gauge Withacre needle. General anaesthesia was induced with fentanil 0.2μg/kg and propofol 2mg/kg and rocuronium 0.6mg/kg. Then, modified US-TAP block was performed using a total of 40ml of Ropivacaine 0.5%[4]. Anesthesia was then maintained with Desflurane adjusted according to the BIS value. Intraoperatively no other opioids were administered. Neuromuscular block status was monitored by datex-Ohmeda monitor stimulator. At the end of surgery and under TOF suggestion, 2mg/kg of Sugammadex was administered. Acetominophen at the dose of 1gr, was administered 15minutes before the end of the surgery. Acetominophen 1gr every 8 hours daily was planned for postoperative analgesia. At rest and dynamic post-operative NRS was measured at 6,12,18,24,36,48 and 72hours postoperatively.

Results:

Very low opioids dose were administered to all patients. In the first 24hours, all patients received Acetominophen 3gr as scheduled. In the following 2days, no pain relief was needed and no patients complained of PONV.

Conclusion:

The benefit of the TAP block in robot-assisted laparoscopic surgery and the beneficial effect of intrathecal administration of low-dose morphine had already been demonstrated[4-5]. Literature, largely suggests the use of opioid sparing anaesthesia to improve patient’s outcomes and to reduce costs. We conclude that this anesthesiological approach for LRa-GIST resection, is safe, well tolerated, provides intraoperative lower opioid requirements, ensures postoperative analgesia and reduces PONV, according to ERAS protocol. However, further larger studies are necessary to clarify the effective role of different anesthesiological strategies in LRa-GIST.

References:

1. Miettinen M., Lasota J. Review gastrointestinal stromal tumors:pathology and prognosis at different sites. Semin Diagn Pathol 2006; 23: 70–83

2. Nguyen S.Q., Divino C.M. Laparoscopic management and long term outcomes of gastrointestinal stromal tumors. J Am Coll Surg 2009; 208(1): 80–86

3. Desiderio J., Trastulli .S, Cirocchi R., et al. Robotic gastric resection of large gastrointestinal stromal tumors. Int J Surg 2013; 11(2):191–196

4. Dal Moro F., Aiello L., Pavarin P., Zattoni F. Ultrasound-guided transversus abdominis plane block (USTAPb) for robot-assisted radical prostatectomy: a novel ‘4-point’ technique—results of a prospective, randomized study. Journal of Robotic Surgery. https://doi.org/10.1007/s11701-018-0858-6

5. Gori A., Aiello L., Bellantonio D., Pitrè C., Dima V., Piccinno M., Corso R.M., Maitan S.. Intrathecal morphine and abdominal wall blocks reduces analgesic consumption and postoperative nausea and vomiting (PONV) in robotic-assisted laparoscopic prostatectomy (RALP). RAPM 2019;44(Suppl 1):A1–A27. 10.1136/rapm-2019-ESRAABS2019.470

### A38. Comparative study of locoregional anesthesia (ESP block + serratus plane block + parasternal block) versus postoperative intravenous analgesia for total mastectomy

#### Giurazza R.^1^, Falso F.^1^, Coppolino F.^1^, Pota V.^1^, Sansone P.^1^, Pace M.C.^1^, Corcione A.^2^, De Rosa R.C.^2^, Passavanti M.B.^1^

##### ^1^AOU "Luigi Vanvitelli" - Scuola di Specializzazione in Anestesia, Rianimazione, Terapia Intensiva e del Dolore ~ Napoli ~ Italia, ^2^AORN dei Colli - Dipartimento di Area Critica ~ Napoli ~ Italia

###### **Correspondence:** Giurazza R.

**Background:** Breast cancer is the most common cancer in women. Breast surgery can be very painful in the postoperative phase, leading to high consumption of analgesics (NSAIDs and opioids). This is even more accentuated in case of total mastectomy, with implantation of breast tissue expanders. In recent years, locoregional anesthesia has significantly improved postoperative pain control and complications, with lower use of postoperative analgesics and better patient compliance.

**Methods:** We performed a prospective comparative study of women, undergoing total mastectomy with implantation of expander due to breast cancer. The patients were randomly allocated to two groups: (1) group A, receiving intraoperative analgosedation (LMA) using desflurane and remifentanil and postoperative analgesia with acetaminophen 1 g q8h, ketorolac 90 mg/die, tramadol 200 mg/die and metoclopramide 20 mg/die; (2) group B, receiving preoperative ultrasound-guided ESP block (level T4 with levobupivacaine 0.375% 20 mL + dexamethasone 4 mg ), serratus plane block (levobupivacaine 0.25% 20 mL + dexamethasone 2 mg) and parasternal block (levobupivacaine 0.25% 10 mL + dexamethasone 2 mg) and intraoperative sedation (LMA) with desflurane only and without remifentanil. In both groups, in case of postoperative pain, morphine 5 mg iv was used as rescue medication. We examined postoperative pain with NRS at emergence of anesthesia (t_0_), at 6 hours (t_1_), at 12 hours (t_2_) and at 24 hours (t_3_), as well as use of rescue medication for pain in both groups. Data were compared using Mann-Whitney U test and statistical significance was accepted if p value was <0.05. Informed consent to publish was obtained by all participants to the study.

**Results:** A total of 24 patients were included in the study, 11 in group A and 13 in group B, with no significant differences of age and ASA status between the two groups. In group A and group B, mean NRS at t_0_ was 5.4 vs 2.3 (p value .0003), at t_1_ 4.4 vs 2.1 (p value .0006), at t_2_ 3.7 vs 2.6 (p value .034), at t_3_ 3.4 vs 2.7 (p value .11, not statistically significant), respectively. Mean morphine rescue use was significantly higher in group A than in group B (respectively, 7.7 mg vs 1.3 mg, p value .024).

**Conclusions:** Our experience, although limited, shows that pain NRS in women undergoing total mastectomy with implantation of breast tissue expanders is significantly lower in those treated with locoregional anesthesia than in those who received standard postoperative intravenous analgesia. The statistical significance for NRS is lost 24h after the end of surgery, probably because at this time the effect of locoregional anesthesia is vanished. Moreover, in the locoregional anesthesia group mean consumption of opioids is significantly lower, with fewer side effects and better safety profile.

**Table 1 (abstract A38).** See text for description

	Group A	Group B	p value
	(mean ± SD)	(mean ± SD)	
**NRS t**_**0**_	5.4 ± 1.4	2.3 ± 1.5	0.0003
**NRS t**_**1**_	4.4 ± 1.6	2.1 ± 0.9	0.0006
**NRS t**_**2**_	3.7 ± 1.3	2.6 ± 0.9	0.034
**NRS t**_**3**_	3.4 ± 1.4	2.7 ± 0.7	0.11

### A39. An innovative ultrasound-guided dorsal penile nerve block for pediatric urologic surgery

#### Pilia E.^1^, Rufini P.^2^, Cumbo S.^2^, De Donato F.^3^, Ricci Z.^4^

##### ^1^Department of Medical Science and Public Health, University of Cagliari, Cagliari, Italy ~ Cagliari ~ Italia, ^2^Department of Anesthesiology and Critical Care Medicine, Pediatric Intensive Care Unit, Meyer Children's Hospital, Florence, Italy ~ Firenze ~ Italia, ^3^Department of Anesthesia and Intensive Care, IRCCS San Raffaele Scientific Institute, Milan Italy ~ Milano ~ Italia, ^4^Department of Health Science, University of Florence, Florence, Italy ~ Firenze ~ Italia

###### **Correspondence:** Pilia E.

Background

In pediatric population, surgery of penis is one of the most common urologic procedures and it causes significant intra and postoperative pain. We propose an innovative ultrasound (US) technique named reversal US-guided dorsal penile nerve block (R-US-DPNB). Our aim is to evaluate the efficacy and safety of R-US-DPNB in pediatric penile surgery compared to Dalens DPNB.

Materials and methods

After establishing general anesthesia, we performed the RUS-DPNB technique using a linear ultrasound probe covered with transparent film and sterile gel. The probe was positioned along the transverse plane, between the root of the penis and the scrotum, slightly angled in a cranial direction towards the pubic symphysis (Fig 1A shows penile US visualization). The anatomical structures are mirrored, and, for this reason, we named this technique reversed US-guided block. An echogenic atraumatic needle (22Gx5cm) is than inserted lateral to the probe and the use of an in-plane technique allows to see the needle tip as a hyperechoic structure while slowly advancing to the target (Fig 1B-C). After a negative aspiration test, an injection of 2 mL of local anesthetic (1 ml LA per each side for children weighing up to 10 kg and then an additional ml for every 10 kg) is performed, separating the deep fascia of the penis from the adjacent tissues (Fig 1D). The needle is then slowly retracted to inject LA to the other side (Fig 1E) and to obtain the spread of LA all-around penile structures (Fig 1F). The block is followed by the application of 2 mL of topical 2% Lidocaine to the foreskin to ensure frenulum analgesia. We evaluated opioid requirements for hemodynamic variations associated with pain, time of execution, complications rate and opioid administration. Postoperative pain has been evaluated using the VAS/FLACC scale.

Results

We retrospectively evaluated 63 patients: 40 patients received R-US-DPNB and 23 patients received Dalens DPNB block.. In the R-US-DPNB group the 12.5% of patients showed hemodynamic variations vs 70% in the Dalens DPNB group (p<0.0001). Fentanyl bolus requirement in Dalens DPNB group was higher (11% vs 77%, p=0.0002). LA volume used to perform the block was reduced in R-US-DPNB (0.13 ml/kg vs 0.34 ml/kg, p<0.0001). The Dalens group showed shorter execution times (3 min vs 2 min, p=0.0001). No serious complications were observed in any patient. Overall postoperative pain control was similar in both groups, even if R-US-DPNB showed lower pain scores 3h after surgery (17% vs 30% with a score>0, p=0.0006).

Conclusions

The intraoperative analgesic effect of R-US-DPNB is higher than Dalens DPNB, is associated with a lower failure rate and a lower use of LA volume to perform the block. The postoperative analgesia of R-US is comparable to Dalens DPNB. We propose R-US-DPNB as a valid and safe alternative to landmark approach.

**Fig. 1 (abstract A39).**
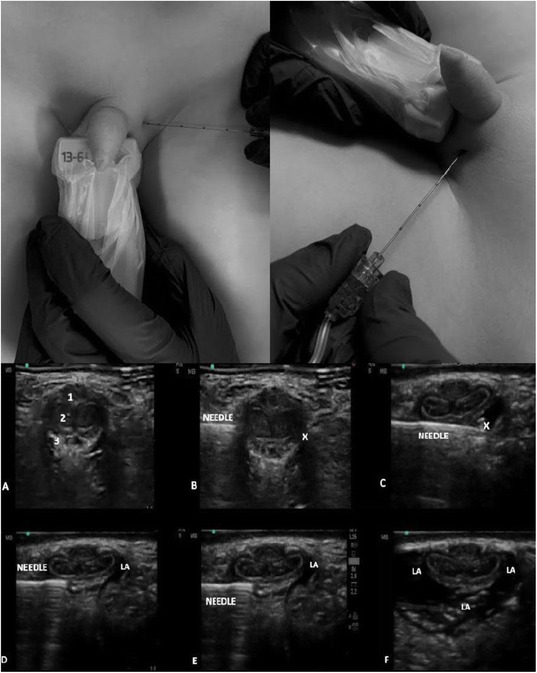
R-US-DPNB technique performed using a linear ultrasound probe (13-6 MHz, 38mm, SonoSite). The corpus spongiosum (1). The two corpora cavernosa (2). Arteries, veins, and the two dorsal nerves of the penis run within the Buck’s fascia (3). X: target. LA: local anesthetic

### A40. "Peng block" vs "FNB + ONB" in the patient with femur fracture who is not a candidate for neuraxial anesthesia

#### Gargano F.^1^, Bellezze A.^1^, Ruggiero A.^1^, Strumia A.^1^, Galderisi A.^1^, Borrelli G.^2^, Citriniti V.^1^, Carassiti M.^1^, Agrò F.E.^1^

##### ^[1]^nit of Anaesthesia, Intensive Care and Pain Management, Department of Medicine, Fondazione Policlinico Universitario Campus Bio-Medico ~ Roma ~ Italia, ^[2]^U.O.C. Anestesia e Rianimazione P.O. San Francesco d'Assisi ~ Oliveto Citra (Salerno) ~ Italia

###### **Correspondence:** Bellezze A.

Background

Locoregional anesthesia is widely used in orthopedic surgery, particularly in traumatology, where surgical indications are relatively common, especially in the elderly, and are associated with significant morbidity and mortality 1.

Ensuring proper anaesthesia in high-risk patients who have suffered a proximal femur fracture can be difficult.

The ideal blocking technique should provide complete analgesia of the hip joint without leading to muscle weakness.

First described in 2018, the PENG block (Pericapsular Nerve Group Block) is an analgesic technique used predominantly in total hip arthroplasties for postoperative analgesia with the advantage of motor sparing 2.

Clinically, the efficacy of the PENG block for analgesia in patients with hip fracture has already been demonstrated 3. The study aims to evaluate the anesthetic efficacy of the PENG block.

Materials and methods

The study population consisted of 40 patients with a proximal femur fracture, who were not candidates for neuraxial anesthesia and underwent reduction and synthesis surgery at the UCBM Trauma Unit.

According to a randomization model, the patients were divided into two groups: In the first group, a PENG block was performed 30 minutes before the patient was placed on the operating table, with Ropivacaine 0.5% (20 ml). In the second group, FNB (Femoral Nerve Block) with Ropivacaine 0.5% (15 ml) and ONB (Obturator Nerve Block) Ropivacaine 0.5% (5 ml) were performed.

All patients received the same multimodal analgesia protocol, in addition, skin access point infiltration with Ropivacaine 0.5% (20 ml) was performed.

Outcomes were recorded using the NRS scale as the primary outcome, intra- and postoperative opioid consumption was also collected, and a ROM study was performed to assess joint flexibility.

Results

Ten cases were collected and randomized into the two intervention groups. Applying the t -student for the NRS scale of pain measured at 30 minutes, recovery room, 6,12, and 24 hours after the anesthetic block, there was no difference between the arithmetic averages of the two groups in all pain outcomes. There was no difference in opioid consumption and no need for increased sedation.

Conclusions.

Finally, no superiority was demonstrated for the PENG group, but being preliminary data together with the small number of studies on the anesthetic efficacy of this technique, not finding any particular differences between the averages is a satisfactory result and gives good grounds for the assumption.

After the study, we expect similar results for the other outcomes measured.

Finally, we demonstrate the use of PENG as a valid alternative to femoral and obturator blockade.

1. Lee DJ, Elfar JC. Timing of hip fracture surgery in the elderly. Geriatr Orthop Surg Rehabil. 2014 Sep;5(3):138-40.

2. Berlioz BE, Bojaxhi E. PENG Regional Block. [Updated 2021 Mar 25]. In: StatPearls [Internet]. Treasure Island (FL): StatPearls Publishing; 2021 Jan-. Available from: https://www.ncbi.nlm.nih.gov/books/NBK565870/

3. Girón-Arango L, Peng PWH, Chin KJ, Brull R, Perlas A. Pericapsular Nerve Group (PENG) Block for Hip Fracture. Reg Anesth Pain Med. 2018 Nov;43(8):859-863.

**Fig. 1 (abstract A40).**
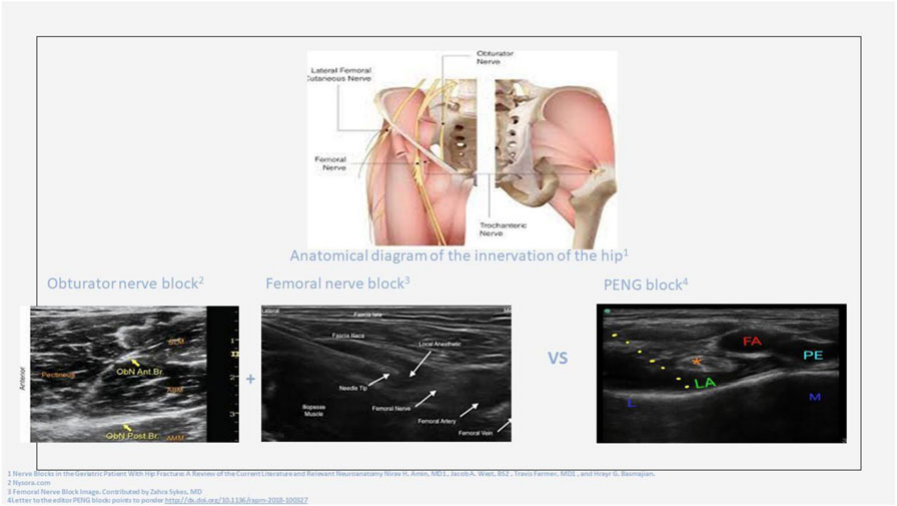
See text for description

### A41. Bilateral ultrasound-guided quadratus lumborum block combined with rectus sheath block for elective open abdominal aortic surgery: a pilot study

#### Natta S.^3^, Melchiorri C.^1^, Bevacqua C.^2^, Caironi P.^4^, Balagna R.^1^

##### ^1^Department of Anaesthesia and Intensive Care, A.O.U. Città della Salute e della Scienza ~ Torino ~ Italia, ^2^Humanitas Ospedale Gradenigo ~ Torino ~ Italia, ^3^University of Turin; Department of Anasthesia and Intensive Care, AOU Città della Salute e della Scienza ~ Torino ~ Italia, ^4^University of Turin; Department of Anaesthesia and Critical Care, A.O.U. San Luigi Gonzaga ~ Orbassano (TO) ~ Italia

###### **Correspondence:** Natta S.

**Background**

Epidural analgesia (EA) has proven superior to systemic opioids for postoperative management of patients undergoing elective open abdominal aortic surgery in terms of pain control, reduced cardiac and respiratory complications, and ICU length of stay [1]. However, EA has some well-defined contraindications (*i.e.,* bleeding disorders, medications, sepsis, history of spinal surgery, severe spinal arthrosis), and aortic surgery itself carries additional risks related to the intraoperative need for anticoagulation. In this setting, fascial plane blocks recently emerged as alternative analgesic techniques when epidural catheterization has failed or is contraindicated. In particular, quadratus lumborum block (QLB) showed efficacy in postoperative multimodal pain strategies in different abdominal surgical procedures [2,3].

In this pilot study, we evaluated the efficacy of bilateral QLB combined with rectus sheath block (RSB) in managing postoperative pain after elective open abdominal aortic surgery.

**Materials and methods**

Nine male patients [median age: 72 years (range 56-79)] undergoing elective open abdominal aortic surgery were selected to receive bilateral posterior QLB combined with RSB before surgical incision. Standard anaesthetic management was inhaled general anaesthesia. QLB and RSB were performed under US-guidance, bilaterally injecting 20 ml of 0,375% ropivacaine and 10 ml of 0,375% ropivacaine, respectively. Intraoperative need for opioids was evaluated case-by-case. All patients received 1g paracetamol and 100mg tramadol before the end of surgery. ICU early postoperative monitoring was performed, providing paracetamol 3g/day and tramadol 200 mg/day as postoperative analgesia. Pain was evaluated with the numeric rating scale (NRS; range: 0-10) at awakening (NRS-0), and at 6 (NRS-6), 12 (NRS-12), and 24 (NRS-24) hours after surgery, as well as the need for rescue analgesic medications (intravenous morphine).

**Results**

No complications were observed while performing QLB (Figure 1A). All patients were admitted to ICU, but one was excluded from our analysis because of prolonged (>24 hours after surgery) sedation. Mean NRS resulted as follows: NRS-0 1.38 (SD 2.56), NRS-6 0.00 (SD 0.00), NRS-12 0.38 (SD 1.06), and NRS-24 0.00 (SD 0.00). NRS-0 scored >4 in two patients, thus requiring morphine rescue analgesic dose (Figure 1B). Three patients required intraoperative opioid infusion (sufentanil, mean dose: 0,17 mcg/kg/h), but no significant difference was observed in terms of NRS 0 (p=0.82) and NRS 12 (p=0.48) compared to the other group. Of note, NRS-6 scored identical within the two groups (Table 1).

**Conclusions**

Bilateral QLB combined with RSB in a multimodal analgesia regimen setting can be considered an effective alternative to epidural analgesia for postoperative pain management in elective open abdominal aortic surgery patients. Based on this data, we aim to develop a prospective randomized clinical trial to evaluate QLB and RSB use compared to systemic opioids for postoperative pain management of elective abdominal aortic surgery patients.

Written informed consent for the publication was obtained from every participant.

**Acknowledgements:** None.

**References**

1. Guay J, Kopp S. Epidural pain relief versus systemic opioid-based pain relief for abdominal aortic surgery. Cochrane Database Syst Rev. 2016 Jan 5;2016(1):CD005059. doi: 10.1002/14651858.CD005059.pub4. PMID: 26731032; PMCID: PMC6464571.

2. Ueshima H, Otake H, Lin JA. Ultrasound-Guided Quadratus Lumborum Block: An Updated Review of Anatomy and Techniques. Biomed Res Int. 2017;2017:2752876. doi: 10.1155/2017/2752876. Epub 2017 Jan 3. PMID: 28154824; PMCID: PMC5244003.

3. Elsharkawy H. Quadratus Lumborum Blocks. Adv Anesth. 2017;35(1):145-157. doi: 10.1016/j.aan.2017.07.007. Epub 2017 Oct 3. PMID: 29103570.

**Table 1 (abstract A41).** Case series characteristics

Patients *	Age	IO opioids §	NRS 0h	NRS 6h	NRS 12h	NRS 24h
**Case 1**	73	No	0	0	0	0
**Case 2**	63	No	6	0	0	0
**Case 3**	72	Yes	5	0	0	0
**Case 4**	75	No	0	0	0	0
**Case 5**	71	No	0	0	0	0
**Case 6**	79	No	0	0	3	0
**Case 7**	56	Yes	0	0	0	0
**Case 8**	71	Yes	0	0	0	0

*IO* intraoperative, *NRS* numeric rating scale

§: intraoperative sufentanil

*: Case 9 was excluded due to prolonged (>24 hours) post-operative sedation precluding NRS assessment

**Fig. 1 (abstract A41).**
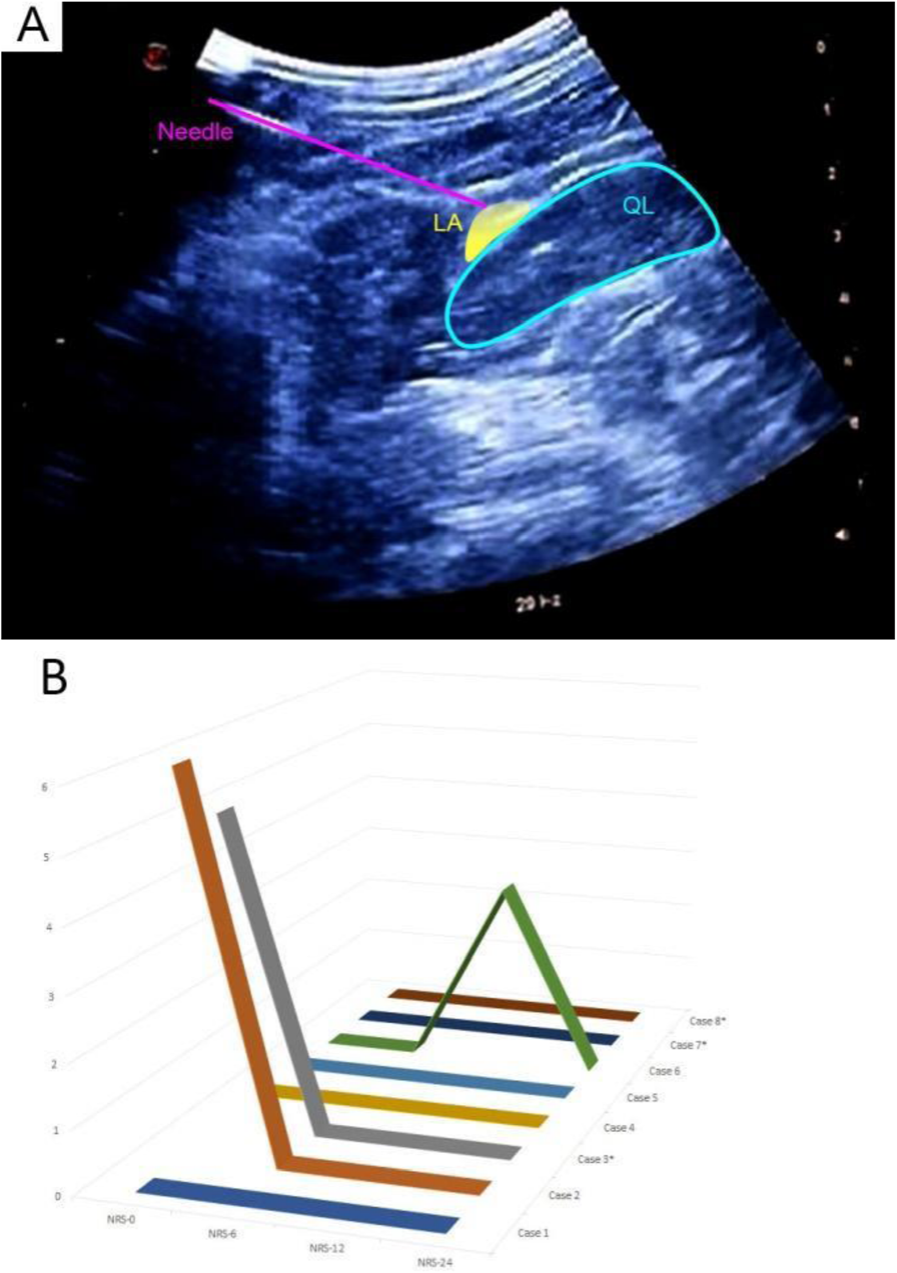
US-guided posterior QLB (A) and patients NRS score (B). A) LA: local anaesthetic; QL: quadratus lumborum; B) *: patients receiving intraoperative sufentanil

### A42. Parturient's satisfaction about labour analgesia: an audit

#### Salatto P.P., Pugliese P.L., Fede N.R., Meola S., Lacerenza A., Di Biase S., Ambruosi A., Curlo N., Contarato C., Ricci E.P., Cinnella G.

##### Azienda Ospedaliero-Universitaria Ospedali Riuniti ~ Foggia ~ Italia

###### **Correspondence:** Contarato C.

INTRODUCTION

Labour analgesia (LA) is considered worldwide as the gold standard for peripartum pain relief and for the newborn’s protection.

LA was introduced in Italian essential levels of assistance (LEA) since march,18th, 2017, but till now its application is patchy all over Italy.

In our academic hospital, LA has been introduced in 2009 and since then the procedures’ number has continuously increased, despite the refusal amongst pregnant women.

Aim of this study is to perform an audit of last three years (2019-2021), in terms of women’s satisfaction and causes of refusal.

MATERIALS AND METHODS

In our department, LA procedural algorithm includes a preliminary interview with the parturient at 37-38 weeks, during which general information on the procedure are provided and informed consent is obtained.

LA is performed with neuroaxial analgesic techniques according to hospital’s protocols.

All women undergoing spontaneous delivery, completed a questionnaire asking for their satisfaction about the procedure, peripartum pain’s levels, eventual adverse effects and reason for not performing LA, if indicated. Answers were collected by a 10-point visual-analogue (VAS) scale.

RESULTS

LA increased from 339/1566 spontaneous deliveries (21.6%), in 2009, to 759/1350 (56,2%) in 2021.

In the study period , there were overall 7732 childbirth, but only 5200 women underwent the preliminary interview, while the remaining 2532 did not mainly because of woman refusal or organization issues.

Among the 7732 deliveries, 3048 (39,4%) were caesarian sections, and 4684 (60,6%) spontaneous deliveries ( 2513 LA and 2171 without LA, NS). Of note, 32.6% women that had consented to LA did not undergo the procedure because they changed their mind (21,6%), or due to excessive dilatation at the anesthesiologist’s arrival or switch to caesarian section (11%).

The questionnaires show that in 2397 (95.4%) cases LA was a pleasant or more than satisfactory experience, while only 116 (4.6%) experienced side effects such as back pain, headache or nausea and vomiting. The wide majority of those who performed LA would do it again (95.3%). Moreover, 61% of those women who refused it , stated that they would do it in a following pregnancy, due to unsustainable pain.

CONCLUSIONS

Our data show that: a) LA is confirmed as a safe procedure; b)misinformation, also accompanied by clinical, cultural or religious issues, do represent the major cause of refusal to LA; c) the best way to increase LA diffusion, is to optimize the collaboration between the obstetric and anesthesiologist’s teams, possibly since pregnancy beginning.

BIBLIOGRAPHY

Pugliese PL, Cinnella G, Raimondo P, et al. Implementation of epidural analgesia for labor: is the standard of effective analgesia reachable in all women? An audit of two years. Eur Rev Med Pharmacol Sci. 2013 May;17(9):1262-8

Marucci M, Cinnella G, Perchiazzi G, Brienza N, Fiore T. Patient-requested neuraxial analgesia for labor: impact on rates of cesarean and instrumental vaginal delivery. Anesthesiology. 2007 May;106(5):1035-45. doi: 10.1097/01.anes.0000265165.06760.c2.

### A43. Pulsed radiofrequency’s role in chronic pain from uncomplicated total knee arthroplasty

#### Barone M.S.^1^, Balato G.^2^, D”Abrunzo A.^1^, Diglio P.^1^, Zecchino A.^1^, Russo I.^2^, Iacovazzo C.^1^, Vargas M.^1^, Buonannno P.^1^, Lo Grieco N.^1^, Coviello A.^1^

##### ^1^Department of Anaesthesia and Intensive Care, University Hospital Federico II Naples, Italy ~ Naples ~ Italy, ^2^Department of Orthopaedics and Traumatology, University Hospital Federico II ~ Naples ~ Italy

###### **Correspondence:** Barone M.S.

Background

Knee arthroplasty surgery has shown a success rate greater than 90% after 15 years from surgery. However, 20% of patients report persistent pain with functional limitation and the painful symptoms don’t derive from arthroplasty complications in one fifth of them [1,2]. Patients with painful total knee arthroplasty (TKA) use more and more often NSAIDs, opioids or steroids, with numerous side effects. Our study aims at investigating the efficacy of ultrasound-guided pulsed radiofrequency (RFP) of saphenous, sciatic and obturator nerves in this population of patients, considering prevalently anterior localization of knee pain.

Materials and methods

Between March 2022 and May 2022, 10 patients with uncomplicated painful TKA were enrolled at University Hospital Federico II Pain Medicine Department in Naples. The evaluation of the results was performed with three scales: Numerical Rating Scale (NRS), Brief Pain Inventory (BPI), Patients' Global Impression of Change (PGIC) Scale, administered before treatment (T0), and after 1-3-6-12 months from the procedure (T1, T2, T3, T4 respectively). The choice of target nerve was made by dispensing, under ultrasound guide, a test dose of 5mL of lidocaine 2%, after local anaesthesia, considering as a positive response a reduction of at least 50% on the NRS scale ten minutes later. The three nerves were examined in order of anatomical involvement starting with saphenous nerve, followed by sciatic nerve and obturator nerve. The first nerve with positive test response was selected as procedure target. Ultrasound-guided RFP was performed in asepsis (Figure 1), after local anaesthesia, with the following parameters: 42 ° C, 45Volt, 2Hz for 7 minutes, sensory and motor tests were done before stimulation (Figure 2). At the end 5mL of 0.4% lidocaine were administered (Figure 3).

Results

The study is still in progress. To date, 10 patients who have previously received test dose have been enrolled. The choice of target fell on saphenous nerve in 9 patients, 50% NRS reduction in 5 of them and 60% NRS reduction in the remaining 4; only 1 case was necessary to recruit sciatic nerve with 80% NRS reduction. Obturator nerve was not selected in any case. From 10 enrolled patients, 5 underwent RFP: 4 targeting the saphenous nerve and 1 targeting the sciatic nerve. Evaluated at T1, all patients reported NRS <4, with significant improvements in life quality according to BPI and PGIC, without using of painkillers.

Conclusions

Ultrasound-guided RFP may represent safe and effective approach to treatment of chronic pain from uncomplicated TKA, avoiding the use of long-term harmful drugs. More applications may be needed to get lasting relief; follow-up, still in progress, will be useful to define the duration of procedure's effectiveness.

References:

1. Wylde V, Hewlett S, Learmonth ID, Dieppe P. Persistent pain after joint replacement: Prevalence, sensory qualities, and postoperative determinants. Pain 2011; 152:566-572.

2. Wylde V, et al. Chronic pain after total knee arthroplasty. EFORT Open Rev. 2018;3(8):461–70.

**Fig. 1 (abstract A43).**
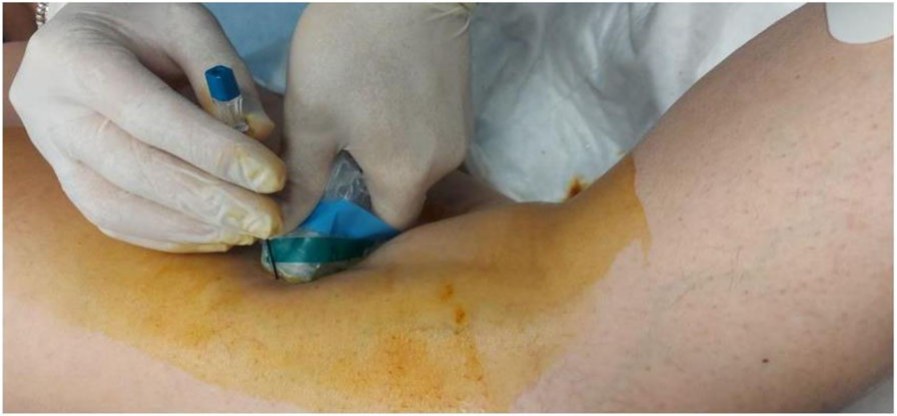
Probe positioning with needle

**Fig. 2 (abstract A43).**
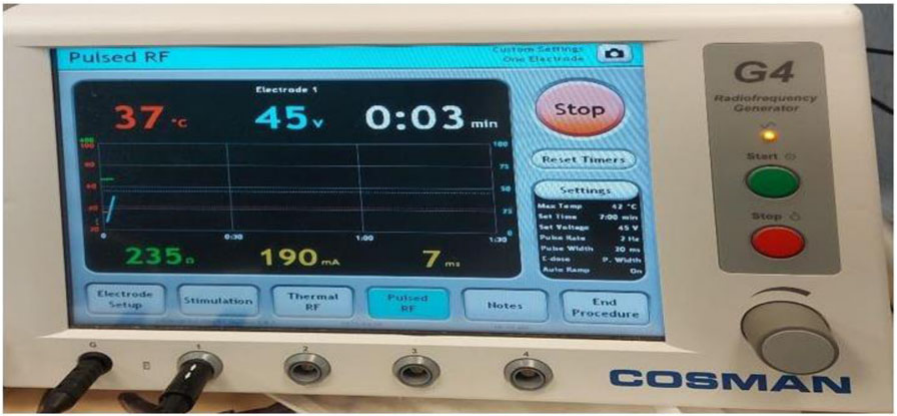
Parameters RFP

**Fig. 3 (abstract A43).**
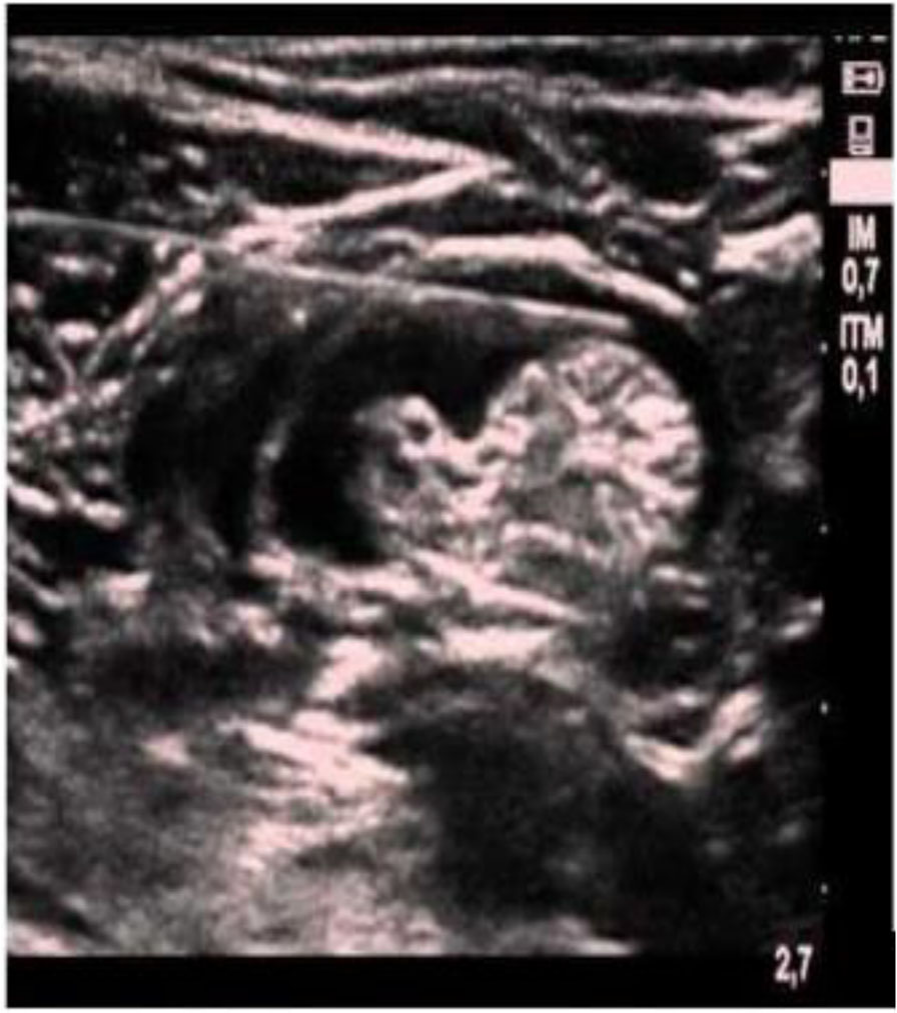
Saphenous nerve after local anaesthetic infiltration at the end RFP procedure

### A44. Use of the dual subsartorial block (DSB) for the control of postoperative pain in patients undergoing knee arthroprothesis

#### Fruncillo A., Baccari L., Chiumiento C., D”Angelo L., Giordano D., La Rocca M.R., Malvasi A., Chiumiento F.

##### Asl Salerno- UOC Anestesia e Rianimazione Ospedale Maria SS. Addolorata ~ Eboli ~ Italy

###### **Correspondence:** Fruncillo A.

The saphenous nerve is a terminal sensory branch of the femoral nerve which innervates the medial portion of the lower limb up to the ankle and foot and which, through infrapatellar branches, is responsible for the innervation of the knee joint. Several anesthetic approaches have been described for saphenous nerve block along its path from the inguinal region to the medial malleolus and, in recent years, the use of ultrasound guidance has improved the success rates of these procedures by considerably reducing postoperative pain and facilitating walking and early discharge. Block of the femoral, obturator and sciatic nerves, responsible for the innervation of the anterior, posteromedial and posterior regions of the knee, respectively, provides complete analgesia in their respective territories but is associated with unwanted motor block of the quadriceps, adductors and hamstrings . Due to this side effect, such blockages are not recommended in the ERAS protocol for knee surgery. The dual subsartorial block combines two subsartorial blocks: the block in the triangle of the distal Scarpa and the block in the adductor canal. From July 2021 to May 2022, 34 Dual subsartorial blocks were performed for post-knee arthroplasty analgesia. The block was always performed at the end of surgery with the patient in the supine position and with the thigh externally rotated to facilitate the positioning of the ultrasound probe and the needle. The first injection is performed using Ropivacaine 0.25% 20 ml and Dexamethasone 4 mg at the level of the distal femoral triangle and targets directly the saphenous and vastus medial nerves. In the second injection, ropivacaine 0.25% 20 ml + dexamethasone 4 mg was used perivascularly under the vastus adductor membrane. Injected at this level, the mixture will travel along the femoral vessels to block the popliteal plexus and then the posterior divisions of the obturator, tibial, common peroneal and sciatic nerve (posterior region of the knee). Patients were evaluated postoperatively at 3-6-12-18-24h. The average duration of the analgesic coverage was 19 +/- 4h. During the first 24h, in 11 cases it was necessary to administer NSAIDs (ketorolac) 1 fl iv x 3 / 24h due to lack of analgesia of the posterior compartment only. In 14 cases, paracetamol was administered as needed for pain (Vas 3-4) at active mobilization. In 5 cases Paracetamol+ketorolac x 3 . Nobody needed iv morphine. This technique can provide excellent analgesia and help in walking and early discharge while avoiding motor block and continuous or intermittent bolus administration of NSAIDs and / or IV opiates. A greater number of cases will most likely allow to refine the technique and further reduce the need for IV NSAIDs. Bibliography Comparing Analgesic Efficacy of a Novel Dual Subsartorial Block Using Two Different Volumes in Patients Undergoing Total Knee Arthroplasty: A Prospective, Double-Blind, Monocentric, Randomized Trial Kartik Sonawane, Hrudini Dixit, Tuhin Mistry and J. Balavenkatasubramanian The Optimal Analgesubramanian The Total Analges Knee Arthroplasty Thomas Fichtner Bendtsen, MD, PhD, * Bernhard Moriggl, MD, PhD, † Vincent Chan, MD, ‡ and Jens Børglum, MD, PhD

**Fig. 1 (abstract A44).**
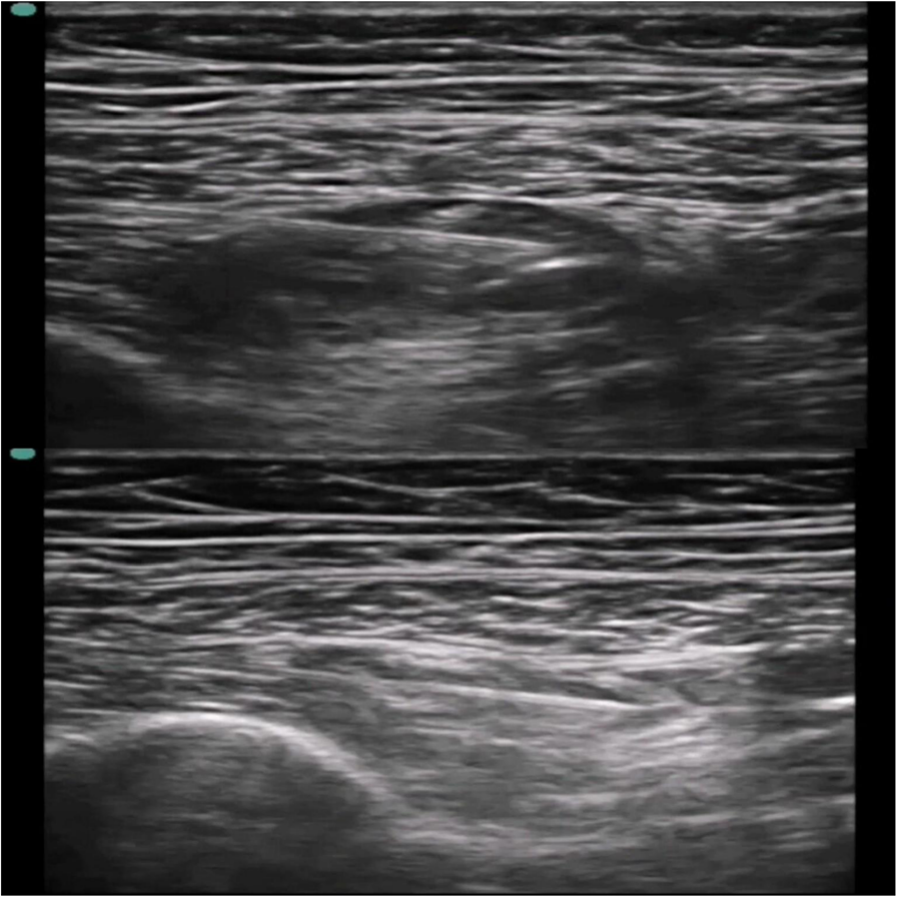
See text for description

### A45. Analgosedation associated with local anesthesia vs serrato and parasternal Blocks vs PCS II associated with parasternal block in S-ICD defibrillators implants

#### Baccari L., Fruncillo A., Russo P., Barra F., Cammarano G., Cuofano P., Chiumiento C., Chiumiento F.

##### Department of Critical Area, ASL Salerno ~ SALERNO ~ Italy

###### **Correspondence:** Baccari L.

Background

In recent years there has been an increase in number of S-ICD subcutaneous defibrillator implants in patients with dilated ischemic cardiomyopathy. Poorly managed chest wall pain contributes to increased incidence of pulmonary complications: hypoventilation, atelectasis, pneumonia. Therefore, it is important to treat surgical chest pain adequately and to reduce opioid administration. The purpose of this study is to compare the effectiveness of analgosedation + local anesthesia vs serratus block + parasternal vs the association of PECSII + parasternal in the implantation of S-ICD subcutaneous defibrillators.

Materials and Methods

From March 2020 to January 2022, we enrolled 80 patients over 60 who underwent implantation of S-ICDs with the twoincision technique. All patients were premedicated with fentanyl 1ɤ/kg and midazolam 0.03 mg/kg. We divided population studied into three groups: 30 AL group (analgosedation + local anesthesia), 25 SAP group (serrato block + parasternal), 25 PECS group (PECSII + parasternal). The AL group was treated with target propofol TCI between 1.4-1.6/ml iv + fentanyl 1ɤ/kg + multiple infiltrations of local anesthetic along the surgical site up to a maximum of 40 ml ropivacaine 0.5%; SAP group was treated with ultrasound-guided serrate block, using ropivacaine 0.5% 25 ml + dexamethasone 4 mg in combination with parasternal block using isobaric levobupivacaine 0.5% 10 ml and in addition propofol TCI with target between 0.6-0.8 y / ml; PECS group was treated, in eco-guide, with PECSII using ropivacaine 0.5% 25 ml + dexamethasone 4 mg with parasternal block using isobaric levobupivacaine 0.5% 10 ml and in addition propofol TCI with target between 0.6-0.8 y / ml. We evaluated patient compliance through request for additional intraoperative analgesia, need for postoperative analgesia (paracetamol, morphine, tramadol, ketoprofen) in relation to NRS at 1, 6, 12 hours, total volume of anesthetic administered, quality perceived by surgeons.

Results

No patient had any complications. Patients in SAP Group required less additional intraoperative analgosedation during tunneling than other two groups. PECS group required local infiltrations along tunneling. In the intraoperative group AL group required: higher propofol target in TCI, greater amounts of fentanyl and local anesthetic than other two groups. Regarding postoperative pain assessed with VAS scales (no significant differences were observed at 1, 6, 12 hours between SAP and PECS group, while AL group required postoperative analgesia. The surgeons rated the anesthetic-analgesic quality on average as very good in the cases of SAP group and sufficient in PECS group.

Conclusions

AL group required greater doses of intravenous anesthetic and analgesic and greater doses of intraoperative local anesthetic as well as postoperative analgesia than other two groups. SAP group ensured better intraoperative analgosedation during tunneling than PECS group. The analgesic-analgesic quality perceived by the surgeons was better for SAP group than for PECS group

References

1.Sepolvere G,Fusco P,Tedesco M,Scimia P. Bilateral ultrasound-guided parasternal block for postoperative analgesia in cardiac surgery: could it be the safest strategy?Reg Anesth Pain. 2020;Vol 45: 316-317

2.Marrone F,Paventi S,Tomei M,Bosco M.153 Ultrasound-guided serratus anterior plane block (US-SAP block) and ultrasound-guided parasternal block (US-PsB) for s-ICD implantation in severe dilated post-ischaemic cardiomyopathy: a case study.Regional Anesthesia & Pain Medicine 2021;Vol70:A80-A81

**Fig. 1 (abstract A45).**
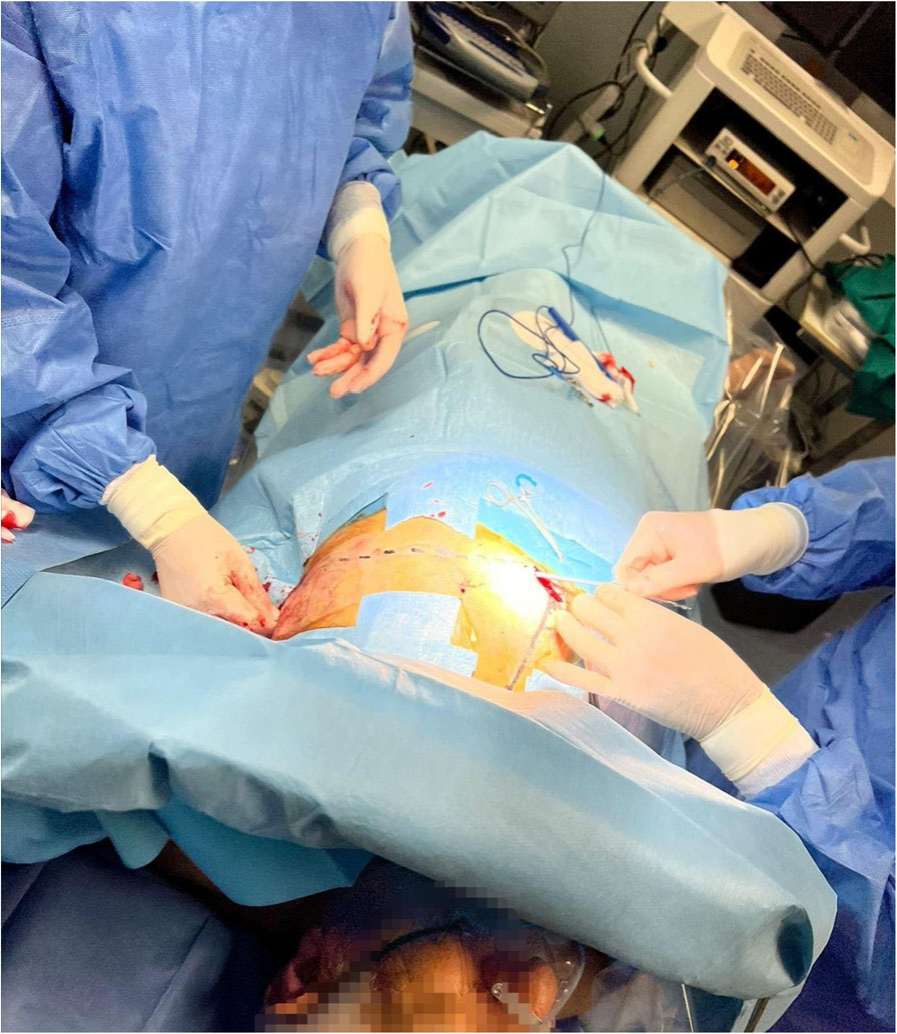
See text for description

### A46. Continuous erector spinae plane block (CESPB) for analgesia in thoracotomy pulmonary lobectomy

#### Rusca G., Gasti G., Bianco M., Truzzi G., Uva A., Mereto N.

##### S.C. Anestesia e Rianimazione, Ospedale Villa Scassi, ASL 3 ~ Genova ~ Italy

###### **Correspondence:** Rusca G.

**Background**

Fascial plane blocks are finding more and more indications for perioperative pain management. These techniques also allow to reduce the administration of opioids, in line with the ERAS protocols. One of the most painful surgeries at the thoracic level is thoracotomy, for which the gold standard remains the thoracic epidural anaesthesia, which guarantees excellent levels of analgesia and a good functional recovery in the post-operative period. However, this technique has to face complications and contraindications, such as bleeding disorders or a low platelet count.

**Case report**

A 78-year-old man diagnosed with lung cancer was scheduled for a VATS right lower lobe lobectomy. At the preoperative blood count PLT 92,000/mcL, so we opted for an US guided right cranio-caudal ESPB at T2, with administration of 10 ml 0.9% saline (SF) to start the hydrodissection between the transverse process and erector spinae muscle, in prone position. Subsequently, we injected ropivacaine 0.375% 20 ml and dexamethasone 4 mg, and placed a catheter to maintain analgesia in the postoperative period. After induction of general anaesthesia (GA), VATS surgery started but conversion to anterolateral thoracotomy was necessary due to the presence of numerous adhesions resulting from a previous sternotomy, and an excessive bleeding. We administered fentanyl 50 mcg as an analgesic during induction of GA, then we rapidly interrupted the infusion of remifentanil due to hypotension and bradycardia even at doses lower than 0,05 mcg/kg/min during surgery. We administered paracetamol 1g, ketorolac 30mg and, in the ESPB catheter, a bolus of SF and ropivacaine as before induction of GA 45 minutes before awakening the patient. After the bolus, we started an infusion of ropivacaine 0.2% 300ml through an elastomeric pump (10 ml/h) connected to the ESPB catheter until chest drainage removal. Upon awakening, the patient referred NRS 5/10, so we administered fentanyl 50mcg with benefit and the patient was transferred to the ward. There, we started monitoring NRS values, administration of rescue painkillers, PONV episodes and the quality of night rest for the following 72 hours. The postoperative analgesic therapy included paracetamol 1g tid and the elastomeric pump for the cESPB just described. As rescue painkillers: ketorolac 30mg if NRS> 4, tramadol 100mg if NRS> 6. In the postoperative period, tramadol 100 mg was administered 3 hours after awakening (NRS 4/10) and ketorolac 30 mg at 28 and 44 hours. PONV did not occur, sleep quality was satisfactory. In POD 1 the patient was mobilized and underwent physiotherapy. The chest drain was removed in POD 6, and the patient was discharged in POD 8.

Informed consent to publish had been obtained.

**Conclusion**

Continuous thoracic fascial plane blocks can be a useful tool in the management of postoperative pain, ensuring a significant decrease in the need to administer opioids, thus following the opioid sparing perspective indicated by the ERAS protocols.

### A47. Post laparotomic hysterectomy analgesia: ultrasound tap block and endovenous analgesia

#### Sicilia R., Manzione N., D”Elia A., Tozzi U., Pisapia A., Arminio D., Landri P.A., Buonavolontà C., Chiumiento C., Chiumiento F.

##### PO Battipaglia Santa Maria della Speranza ~ Salerno ~ Italy

###### **Correspondence:** Sicilia R.

INTRODUCTION

Locoregional anesthesia is strongly recommended whenever possible to complement classical analgesia techniques as part of the multimodal approach to pain.Patients undergoing laparotomic total hysterectomy complain of significant postoperative pain. Precisely to reduce the frequency and intensity, the TAP block with injection of local anesthetic is often used in the transversus abdominis plane or TAP (plane located between the oblique and transverse muscles) where, among others, the nerve branches of the intercostal nerves T9T12 and of the first lumbar root of L1.

MATERIALS

The purpose of our work was to evaluate the effectiveness of the TAP block performed before the surgical incision in patients undergoing total laparotomic hysterectomy compared to conventional analgesia with opioids and intravenous NSAIDs in the postoperative period. We evaluated 20 patients aged between 45 and 80 years with an ASA 2-3, performed under general anesthesia (diprivan 2 mg / kg, fentanyl 2 gamma / kg, esmeron 0.6mg / kg, desflurane mac 1). the patients were divided into two groups:

T: 10 patients treated with bilateral injection before surgical incision of levobupivacaine 0.375 mg 20 ml at the level of the TAP

M: 10 patients treated postoperatively with 2ml / h analgesic pump with morphine 10 mg, ketorolac 90 mg, metoclopramide 10 mg iv.

We assessed the intensity of pain over 24 hours by measuring it every 6 hours according to the NRS scale (Numeric Rating Scale scale from 0 to 10) and the appearance of PONV in the post-operative period.

RESULTS

The analgesic efficacy of the two methods measured with the NRS scale showed progressive pain relief after 6 h for both, with a greater reduction in intensity (value <5 to 12 h) for the group treated with TAP block compared to the group with Morphine (value of 6 to 12h). PONV complications were 21% in the TAP group and 54% in the Morphine group. The use of the TAP block, therefore, in addition to having shown a greater antalgic efficacy than the use of intravenous opiates, has made it possible to reduce respiratory complications,reduce paralytic ileus,promote earlier nutrition and earlier mobilization.thus facilitating his resignation.

CONCLUSIONS

Although the small number of patients treated,it can be concluded that the use of TAP Block as an analgesic strategy in patients after total hysterectomy seems to have more satisfactory results in terms of pain intensity and postoperative complications than in patients treated with intravenous opioids.In addition,the ultrasound guide made it possible to view the TAP and to control the precise infusion of local anesthetic by reducing its quantity and therefore its toxicity.

BIBLIOGRAPHY

• Trasversus Abdominis Plane Block: a evaluable option for postoperative analgesia? A topical review Petersen, O. Mathiesen, H Torup, J.B. Dahl; Acta Anaesthesiol Scand2010; 54: “The 529-535

• “Epidural versus continuous Trasversus Abdominis Planecatheter technique for postoperative analgesia after abdominal surgery” RaoKadam V, Van Wijk RM, Anaesth Intensive Care 2013 Jul; 41 (4): 476-81

### A48. The use of ultrasound in neuraxial anesthesia: an approach for trainees

#### D”Abrunzo A.^2^, Barone M.S.^2^, Diglio P.^2^, Russo I.^1^, Iacovazzo C.^2^, Vargas M.^2^, Buonanno P.^2^, Logrieco N.^2^, Coviello A.^2^

##### ^1^Department of Orthopaedics and Traumatology, University hospital Federico II ~ Naples ~ Italy, ^2^Department of Anesthesia and Intensive Care, University hospital Federico II ~ Naples ~ Italy

###### **Correspondence:** D”Abrunzo A.

Objectives: Ultrasound approach, performed in central nerve blocks, represents an essential resource to the good clinical perioperative practices. Traditionally, neuraxial blocks are performed using a combination among anatomic landmarks, tissue perception during the needle advance and the leakage of the cerebrospinal fluid [1,2]. Even if the spinous processes are reliable anatomic landmarks, they are not always recognisable in obese patients and in ones with spinal deformity or undergone spinal surgery [3]. In the same way, the estimation of an intervertebral level, that is based on the identification of the Tuffier’s line, may not be accurate in many patients. Even though there are still conflicting results on the superiority of the ultrasound approach compared to other techniques, the pre-procedural ultrasound imaging can be considered an efficient way to overcome unexpected technique difficulties [4]. The aim of this study is to analyse the potential benefits of pre-procedural ultrasound compared to conventional techniques with landmarks in terms of: number of punctures, number of redirections, time needed for single procedure and total procedure time.

Materials and methods: From December 2021 to May 2022, at the Department of Orthopaedics and Traumatology of the University hospital Federico II of Naples, patients undergone knee or hip arthroplasty, in neuraxial anesthesia, were enrolled. Patients were randomized into two groups. In the first group (G1) patients were subjected to neuraxial procedure, after an ultrasound approach, through two scans (transverse and parasagittal). In this way, the skin puncture mark was identified. In the second group (G2), the neuraxial procedure was performed through identification of anatomical landmarks (Touffier’s line and spinous processes). For both groups, the procedures were performed by the same twenty trainees, all with less than 50 procedures performed.

Results: 93 patients were enrolled in two groups, G1 (48 patients) and G2 (45 patients). Demographic differences (age, gender, BMI) were not observed between the two groups. Both groups were comparable in terms of foreseen procedural difficulties. Each trainee performed about 2.3 procedures for each group. The number of punctures was 1.25 for G1 compared to 1.82 for G2; the number of redirections were 0.9 for G1 compared to 2.46 for G2 (figure 1). The average procedure time was 40s for G1 compared to 99s for G2 (Figure 2). The average time for ultrasound scanning was 67s; thus, the total procedure time for G1 was 107s compared to 99s for G2 (figure 3). Tutor intervention was never necessary for G1, on the contrary, G2 needed it 7 times.

Conclusions: Ultrasound approach can be a good solution for operators with low experience in neuraxial anesthesia in terms of number of punctures, redirections and procedural time. In addition, the total procedure time (ultrasound scan + procedure) is superimposable between the two groups. Ultrasound approach proved to be more effective because the tutor intervention was not necessary.

**Fig. 1 (abstract A48).**
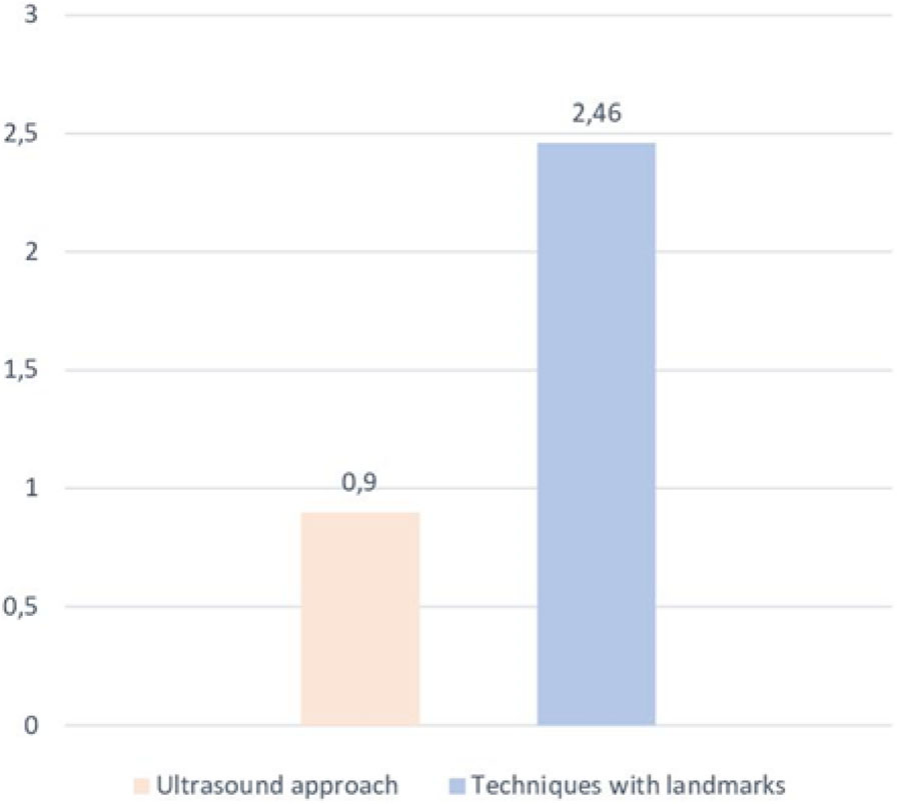
Number of redirections

**Fig. 2 (abstract A48).**
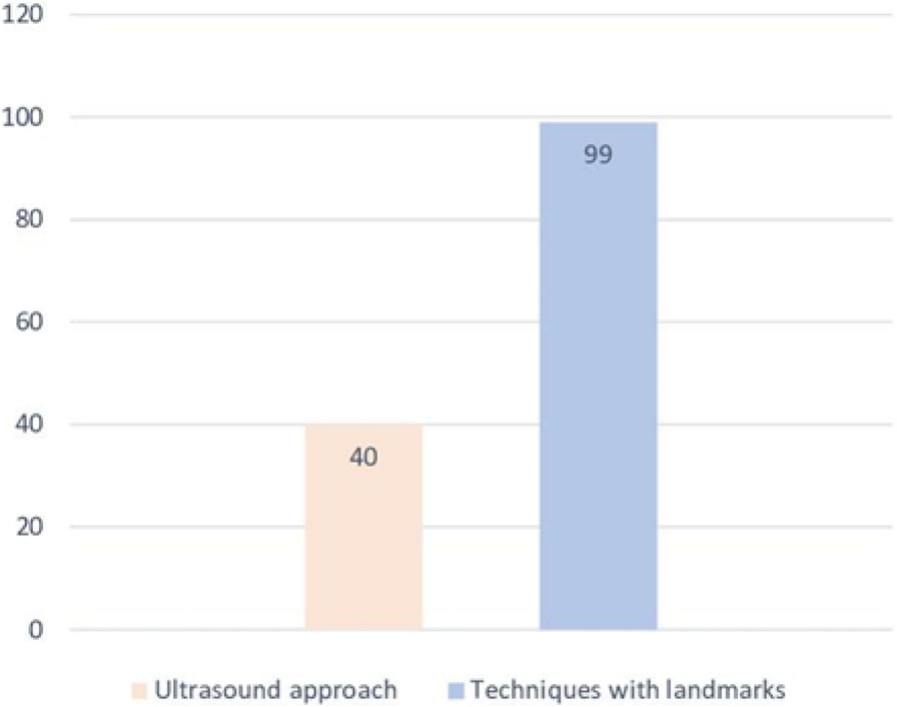
Single procedure time

**Fig. 3 (abstract A48).**
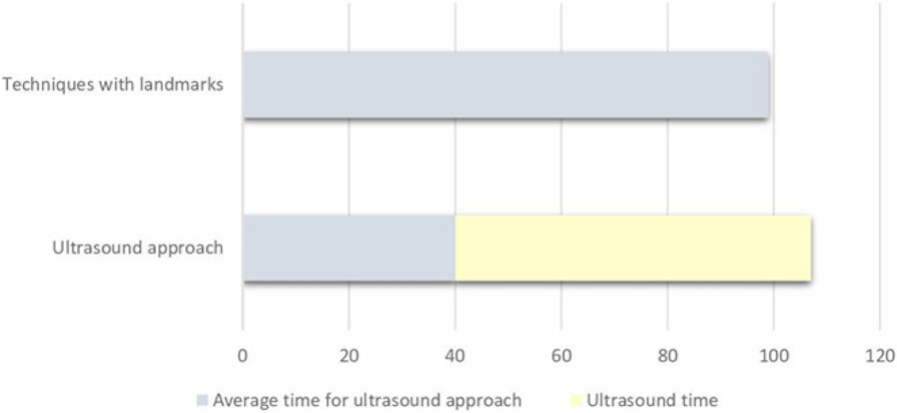
Total procedure time

### A49. Comparison between paravertebral block (PVB) associated with continuous patient-controlled epidural analgesia (PCEA) VS PCEA for colorectal cancer surgery eras protocol

#### Angellotti L., Gianpasquale L., Salvatelli M., Sucre M.J., Cesarano N., Ragone R.

##### Anesthesia and Intensive Care Department. San Leonardo Hospital ~ Castellammare di Stabia (Napoli) ~ Italy

###### **Correspondence:** Angellotti L.

Introduction

The paravertebral block and the PCEA are locoregional analgesic procedures that allow you to avoid the risks and complications related to general anesthesia. We describe the utility and efficacy of the PVB + PCEA technique vs the PCEA alone in the enhanced recovery after surgery (ERAS) protocol, as an alternative without the use of opioids, in the treatment of acute post-operative pain. -abdominal surgery in oncological abdominal surgery.

Methods

We report our initial one-year experience, January 2021-February 2022. 11 patients underwent intestinal resection for K of the transverse colon, right or left colon, anterior rectal resection. PVB was performed by administering 10 ml of 0.375% ropivacaine per segment, under ultrasound guidance. The adequacy of the block was assessed using a pinprick test. The PCEA was conducted by positioning an epidural catheter for analgesic purposes for the continuous infusion of Ropivacaine 0.25% at 5 ml / h with an Ambit ™ PCA pump. The efficacy, as an average NRS, was assessed in the immediate postoperative period, at 6 and 12 hours after the end of the operation. Vital parameters and the incidence of adverse events were monitored: nausea, vomiting, respiratory depression, itching.

Results

The average NRS in the immediate postoperative period was comparable to that found at 6 hours after surgery (average NRS = 3.0), as regards the PVB + PCEA association; The NRS 6 hours after surgery was higher than that in the immediate postoperative period (4.0 vs 3.0) for the PCEA alone. The average NRS at 12 h was 3.9 vs 4.5 respectively for PVB + PCEA vs PCEA. No adverse events were found.

The PVB-PCEA association was effective in 70% of patients (NRS <3).

Conclusions

PVB-PCEA is a safe, effective and reliable analgesic technique that offers numerous advantages over other approaches in ERAS, reporting greater, although not statistically significant, efficacy to the PCEA alone. The available armamentarium of locoregional analgesic techniques is added to ensure pain control in abdominal cancer surgery patients having the need for other cases to determine significant superiority.

Bibliografia

1. Ann Surg 2015;261:1153–1159

2. J Clin Anesth. 2020 aprile 28;64:109850

3. ICAR21-ABS-4267-101-117627-20210605181821

### A50. Supra-inguinal fascia iliaca Block VS PENG Block + Femoral nerve block for femur neck fracture: perioperative efficacy

#### Caputo C., Salvaggio I., Grandino D., Penna G., Vinciguerra A., Ricci F.

##### Ospedale San Paolo ASL Roma 4 ~ Civitavecchia ~ Italy

###### **Correspondence:** Caputo C.

Background: femur neck fracture surgery often reveal several challanges related to “very elderly patient”, such as the difficulty in performing Subarachnoid Anesthesia (SA), due to pain and lacking in compliance. Therefore, performing a preoperative peripheral nerve block (PNB) could be beneficial, not only for a better managemet of postoperative pain, but also to improve patient’s comfort to accomplish SA. In this study we compare two PNB tecniques: supra-inguinal Fascia Iliaca Block (SIFIB) vs PENG Block + Femoral nerve Block (PENG+FB)

Material and methods: between October 2021 and April 2022, 128 patient undergoing femur neck fracture surgery with SA were enrolled and randomized in two groups: SIFIB and PENG+FB. Both PNB were performed under ultrasound guide whit 30 ml of Ropivacaine 0,375% (SIFIB 0,375% 30 ml; PENG 0,375%15 ml + FB 0,375%15 ml). Pain level was evaluated at T0 (entrance in surgery room), T1 (10 minutes after PNB execution), T2 (transfer to surgery bed in sitting position) and T3 (SA execution);Numerical rating scale (NRS) was used with compliant pantient; with non compliant patients Non Verbal Pain Indicators (NVPI), such as lament, anxiety, search for antalgic position, were considered as discomfort alert. Fentanyl 50 mcg and Propofol 10 mg were administered if NRS > 5 or if a NVPI made SA performing difficult or impossible. PNB efficacy was evaluated as requirement of intra-venous sedation. Moreover, we compare performing time of both PNB.

Results: groups are comparable in age, sex and fracture type. No differences have been detected in pantients who required intra-venous sedation (SIFIB 9,6%; PENG+FB 11,52 % p > 0,05). A statistically significant difference resulted in PNB performing time (SIFIB 107,78±30 sec. ;PENG+FB 200,79±21,78 sec p < 0,05). No adverse events occured in both groups.

Discussion and conclusions: for very elderly patient, convetional methods for pain evaluation are often not reliable: therefore, we consider to evaluate PNB efficacy in terms of intra-venous sedation requirement more appropriate; although no differences have been revealed in number of patients requiring IV sedation between two groups, performing time of SIFIB was shorter: this could be considered convenient for pantient’s peri-operative comfort. Identifying a safe and easy-performing PNF could lead to a widespread protocol application for early management of pain in femur neck fracture.

References:

[1] Del Buono R et al. Pericapsular nerve group block: an overview. Minerva Anestesiol. 2021

[2] Wang YL et al. Ultrasoung guided, direct suprainguinal injection for fascia iliaca block for total hip artroplasty: a restrospective study.World Jin Clin cases 2021

**Fig. 1 (abstract A50).**
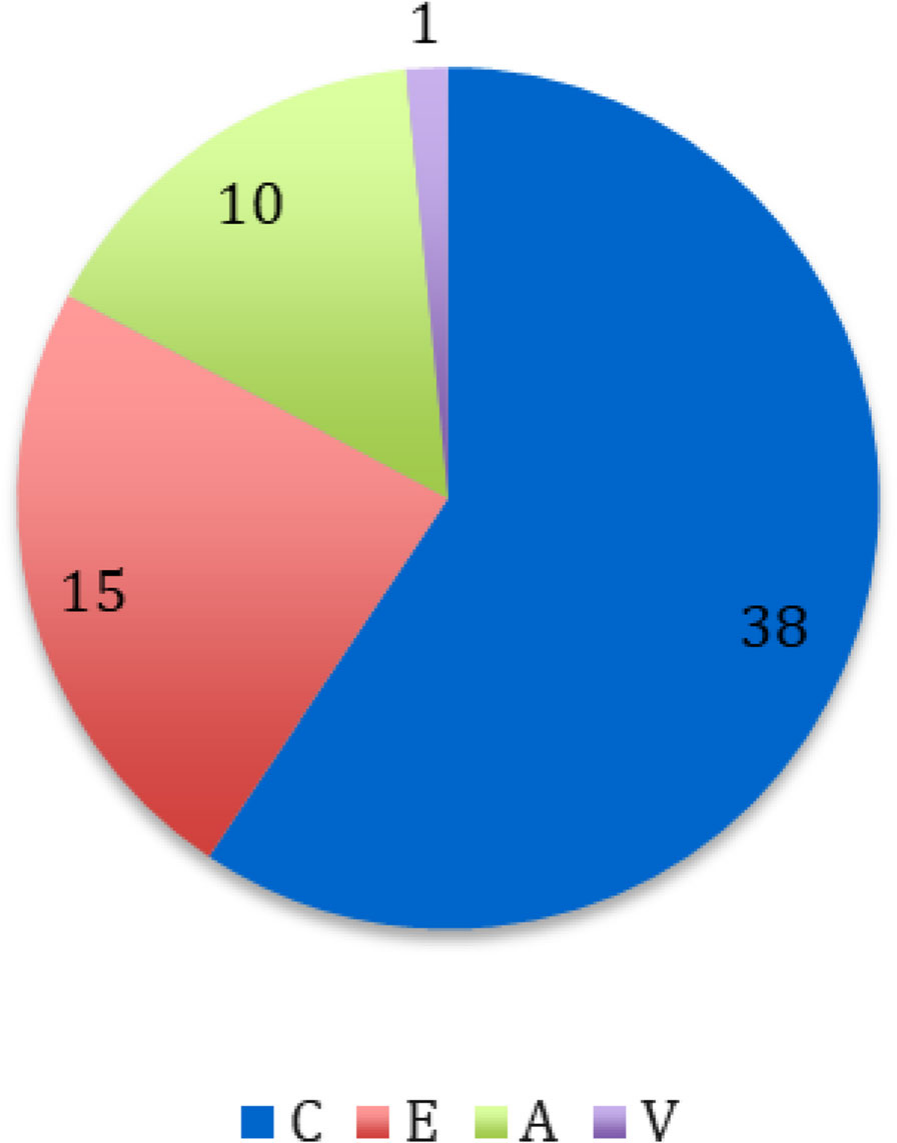
Femur fracture surgery (SIB)

**Fig. 2 (abstract A50).**
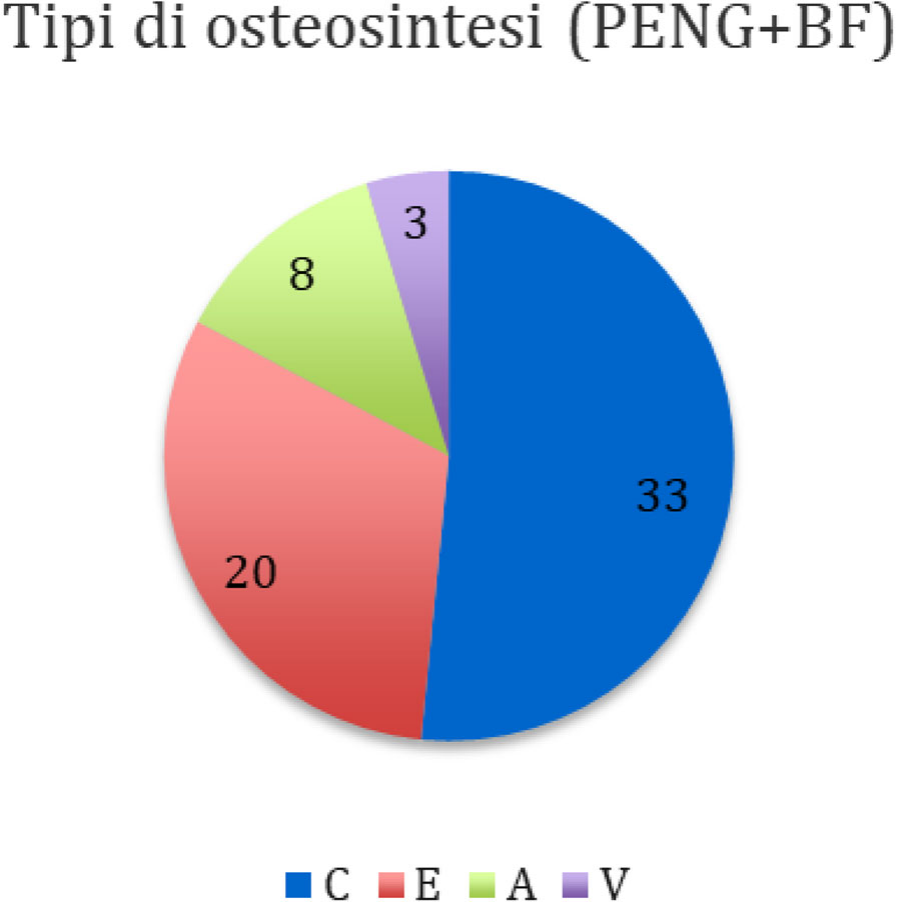
Femur fracture surgery (PENG+ BF)

**Table 1 (abstract A50).**
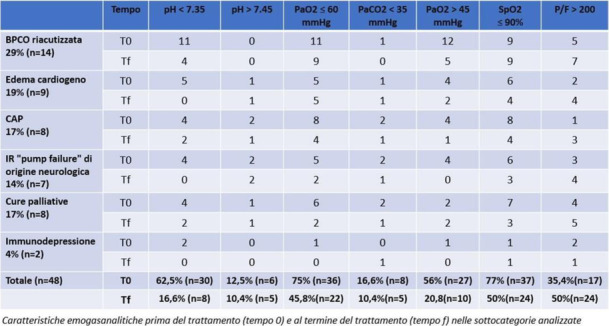
Sedation need

### A51. The use of dexmedetomidi as an adjuvant (NEURAXIAL) in interscalenic block of the brachial plexus in shoulder surgery

#### Toma M., Del Sordo F., Di Matteo S., Sanzò C., Araosta F.

##### Department of Anesthesia and Intensive Care, "San Pio da Pietrelcina" Hospital - Vasto (CH) ~ Vasto ~ Italy

###### **Correspondence:** Toma M.

ABSTRACT

Background: Dexmedetomidina (DEX) is a widely used anesthetic belonging to agonists with high selectivity for α2 adrenergic receptors, showing synergy with local anesthetics used in locoregional anesthesia and facilitating postoperative pain control. However, it has not received US Food and Drug Administration (FDA) approval for neuraxial administration.

Objective: In an orthopedic referral center for shoulder surgery we used DEX as a neuraxial adjuvant in interscalenic block.

Study design: controlled trial

Settings: Orthopedics operating room, "San Pio da Pietrelcina" Hospital - Vasto

Methods: Seventy patients scheduled for elective arthroscopic shoulder surgery were enrolled in this study. Ultrasound-guided interscalenic brachial plexus (ISB) block was performed prior to general anesthesia using 10 ml of 0.5% levobupivacaine and 10 ml of 2% lidocaine in combination with 100 (group <50 years) or 25 μg of DEX (over 50 age group). The third group (NO DEX group) was not given the drug. The primary outcome was the duration of analgesia with the use of the numerical pain rating scale (NRS) and the consumption of additional analgesics during 48 hours after ISB. Secondary outcomes include the duration of motor and sensory block, haemodynamic variables, sedative effect, and additional opioids during general anesthesia.

Results: The NRS in the DEX group was better than in the NO DEX group with a statistically significant value of 24 hours from the ISB (p <0.05) as well as the consumption of additional analgesics. All secondary results are favorable to the DEX group, primarily the statistically significant duration of the sensitive block (p..0.05).

Limitations: The most important limitations of the study are the reduced number of cases, the heterogeneity of the groups, the greater presence of patients soon over 50, the reduced number of operations to reduce the fracture of the humerus.

Conclusion: This study demonstrates, in line with other precedents, that DEX appears to be the best neuraxial adjuvant available, whose safety and manageability also makes it useful in trauma interventions. It is hoped that guidelines on the subject will be published soon after the inclusion of DEX as a neuraxial drug.

### A52. Low-concentration of levobupivacaine subarchnoid anesthesia for femur neck fracture surgery with endomedullary rod in very elderly patients: our experience

#### Papi V.^1^, Rossi L.^1^, Marzilli A.^1^, Grandino A.^1^, Ricci M.^2^, De Tommaso O.^1^

##### ^1^Ospedale S.Paolo ASL Roma4 ~ Civitavecchia ~ Italy, ^2^Ospedale Sant' Andrea, facoltà di medicina e psicologia ~ Roma ~ Italy

###### **Correspondence:** Papi V.

Background: subarachnoid anesthesia (SA) is the most used tecnique in femur neck fracture surgery. Performing a preoperative peripheral nerve block (PNB)[1] and selective subarachnoid anesthesia (SSA) [2] allows to reduce the dose of local anesthetic needed to achieve an appropriate sensitive and motor block. However, very elderly patients are often affected by several comorbidity such as arthrosis, stiffness, bedsore, cognitive impairment and lack of compliance; in this kind of patient, SSA (in lateral position) could be difficult to perform, and does not guarantee an adequate perioperative comfort (surgical position needs non fracturated leg in “flexo-abduction”). In this study, we describe our experience of SA performed with low concentration of levobupivacaine: this allows using of low dose of local anesthetic to achieve an adequate bilateral motor and sensitive block.

Materials and methods: 20 patients undergoing femur neck fracture surgery with intramedullary rod. A PNB was performed to reduce postoperative pain (Ropivacaine 0,375% 30 ml). After 20 minutes SA was performed in sitting position with levobupivacaine 0,25% (7,5 mg, 3 ml); inter-vertebral space L4-L5 was identified by ultra-sound guide. Patients were immediatly arranged in supine position. Sensitive and motor block level was assessed trough Pinprick test and Bromage Score after 10 and 15 minutes; patients were arranged in surgical position. Sensitive and motor block level was assessed again at the end of surgical procedure. Variations >15% from basal value of mean arterial pressure and cardiac rate were recorded, such as need for intra-venus sedation due to block fail, and adverse events connected to SA.

Results: mean surgical procedure duration was 38±15 minutes. After 10 minutes had an adequate level of sensitive block (T12: 80%; T10: 20%) and motor block (Bromage 3. 10%, 2. 75%, 1. 15%). After 15 minutes a cranial progression of block was observed, both sensitive (T12: 10%, a T10: 65%, a T8: 25%) and motor (Bromage 4: 5%, 3: 70%, 2: 10%). At the end of surgical procedure, sensitive block persisted in all patient whit a further, slight cranial progression (T12: 5%, T10: 65, T8: 30%), whereas motor block was partially regressed (Bromage 4: 5%, 3: 25%, 2: 60%, 1: 10%). No intra-venous sedation was needed, no variations in mean arterial pressure and cardiac rate and no adverse events were recorded

Discussion and conclusions: according to our experience, performing SA whit low concentration levobupivacaine is effective and safe in femur neck fracture surgery with intramedullary rod. Low dose of local anesthetic in high volume allows to achieve an adequate sensitive and motor block, avoiding emodinamic adverse events. This ensure a good perioperative comfort for patients, avoiding lateral position (essential to achieve a selective block) and making surgical position more comfortable.

Bibliography:

[1] Guay J, et al. Peripheral nerve blocks for hip fractures in adults. Cochrane database Syst Rev.2020

[2] G B Pappalardo et al. Complication of superselective subarachnoid anesthesia with Hyperbaric bupivacaine: experience with 355 patients in general and orthopedic surgery. Panminerva med.1997 Mar

**Table 1 (abstract A52).** Sensitive block onset and progression

	T12	T10	T8
10minuti	80%	20%	0%
15minuti	10%	65%	25%
Fine intervento	5%	65%	30%

**Table 2 (abstract A52).** Motor Block onset and progression

	Bromage 4	Bromage 3	Bromage 2	Bromage 1
10 minuti	15%	75%	10%	0%
15 minuti	0%	10%	85%	5%
Fine intervento	10%	60%	25%	5%

**Fig. 1 (abstract A52).**
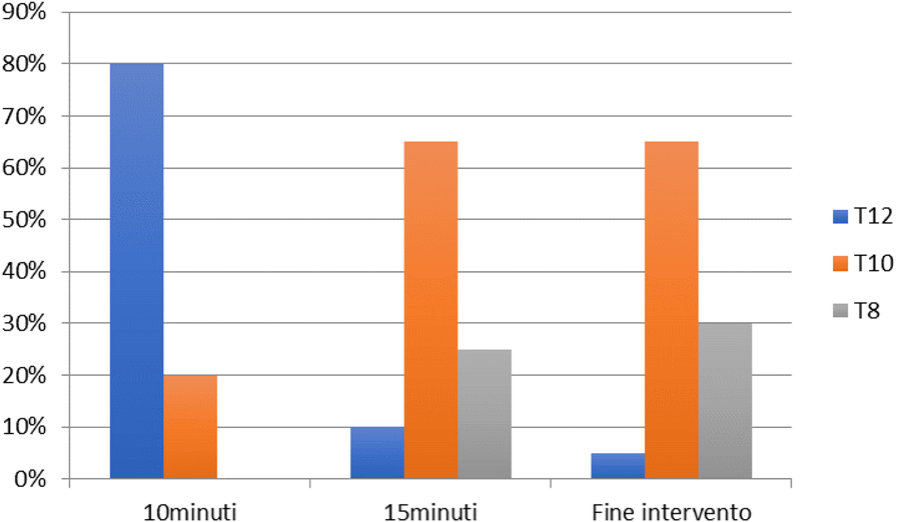
Sensitive block onset and progression

**Fig. 2 (abstract A52).**
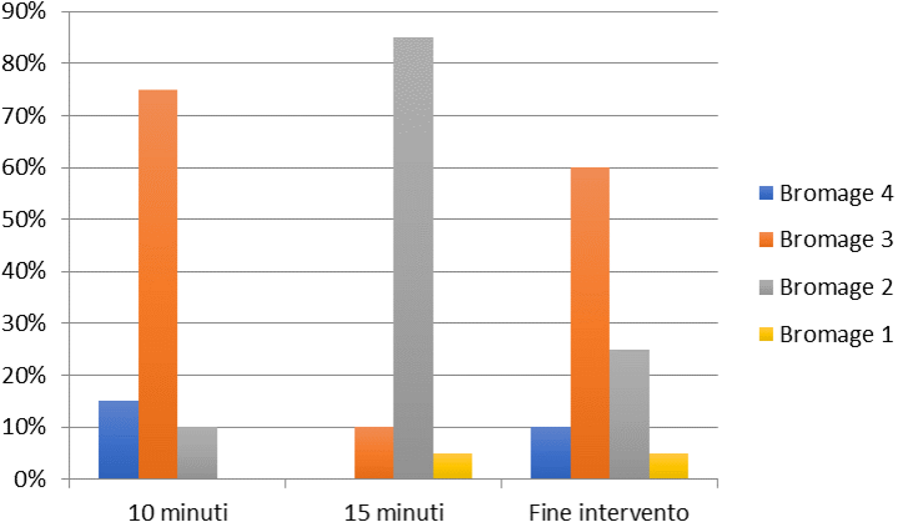
Motor Block onset and progression

## Anestesia nel paziente fragile

### A53. Awake anaesthesia for radical mastectomy in severely ill patient: the role of PECS I-II, SAP and parasternal block

#### Aiello L.^1^, Gori A.^1^, Ione E.^2^, Corso R.M.^2^, Maitan S.^1^

##### ^1^Anestesia e Rianimazione, Dipartimento Chirurgico, Ospedale GB Morgagni-L. Pierantoni ~ Forlì ~ Italia, ^2^Department of Morphology, Surgery and Experimental Medicine, Section of Anesthesia and Intensive Care, University of Ferrara ~ Ferrara ~ Italia

###### **Correspondence:** Aiello L.

Background:

Breast cancer is becoming the leading cause of cancer morbidity and mortality among women around the world[1]. In countries with a high-performance healthcare system, early-stage breast cancer can often be cured[2]. PECS blocks provide effective intra- and postoperative analgesia for breast surgery representing a valid alternative to general anesthesia particularly in high risk patients [3].

Case report:

A 76 years-old severely ill woman was scheduled for left radical mastectomy. She was obese (BMI 36), affected by a severe chronic respiratory failure in Long-Term Oxygen Therapy, chronic heart failure with moderate aortic stenosis, chronic kidney disease and diabetes mellitus (ASA 4). Written informed consent was obtained from the patient.

After ASA standard and BIS monitoring, moderate sedation by Midazolam 0.5mg and O2 supplementation was applied. Ultrasound guided PECS I-II, SAP and parasternal block were performed (B-Braun Ultraplex360® 50mm 22G needle) with Ropivacaine 0.375%: 15ml were injected for PECS I-II, 8ml for SAP, and 4ml for parasternal block. At the end of the surgery Acetominophen 1gr and Ondansetron 4 mg iv were administered. Postoperative scheduled analgesia consisted of Acetominophen 1gr 3 times/day, Tramadol 50mg iv as rescue drug. NRS was used to assess pain and measured at 6 and 72h postoperatively. In the first 72 hours after surgery, only 100 mg of tramadol were administered to maintain NRS <4, no antiemetic agent was necessary. The patient’s vital signs were stable during surgery. During post-operative period, the patient reported good pain control, no nausea or vomiting, and early mobilization.

Conclusions:

Opioid-based general anaesthesia, is usually performed for radical mastectomy, however this classical approach may not be adequate especially in critically ill elderly patients due to its known adverse effects on patient outcome[3,4]. Regional anesthesia it is a key element of any ERAS program and can allow to avoid not only opioids but even the invasiveness of general anaesthesia [5].

This case report suggests the idea that the combination of different thoracic wall blocks could represent a valid alternative to conventional approach, especially in high anesthetic risk patients undergoing breast cancer surgery reducing the perioperative risk and ensuring a good pain control.

In this case report, we describe the use of thoracic wall blocks for awake breast surgery in a high risk patient.

References

1. Gierisch J.M., Coeytaux R.R., Urrutia R.P., Havrilesky L.J., Moorman P.G., Lowery W.J., et al. Oral contraceptive use and risk of breast, cervical, colorectal, and endometrial cancers: a systematic review. Cancer Epidemiol Biomarkers Prev 2013;22(11):1931–43

2. El Saghir N.S., Anderson B.O. Breast cancer early detection and resources: where in the world do we start? Breast 2012;21(4):423–5

3. Blanco R. The ‘pecs block’: a novel technique for providing analgesia after breast surgery. Anaesthesia, 2011, 66, 840–852

4. Fletcher D., Martinez V. Opioid-induced hyperalgesia in patients after surgery: A systematic review and a meta-analysis. Br J Anaesth 2014; 112:991-1004

5. L Jungqvist O., Scott M., Fearon K.C. Enhanced recovery after surgery: a review. JAMA Surg. 2017;152(3):292-298.

### A54. Peripheral nerve blocks for hip fracture in a super-old patient

#### Ferraccioli D.^1^, Fiume D.^1^, Bruno E.^2^, Liciani G.^1^, Galletti M.^1^

##### ^1^Sant'Eugenio Hospital ~ Rome ~ Italia, ^2^University of Rome Tor Vergata ~ Rome ~ Italia

###### **Correspondence:** Ferraccioli D.

Background

Hip fracture is the most frequent cause of hospitalization in orthopedic field. Italy is one of the oldest countries in the world, with 173.1 over65 people every 100 young people under the age of 15. It is estimated that in 2050 people over65 will represent more than 12% of the entire population [1]. A systematic review of 72 studies carried out in 63 different countries revealed that Italy is among the countries with the highest incidence of hip fractures, registering an annual incidence of over 300 per 100,000 inhabitants for women, and over 150 for men [2].

Case Report

We present a 96-year-old male patient undergoing a gamma nail for left subtrochanteric fracture via three targeted surgical accesses. His medical history includes systemic arterial hypertension, chronic heart failure, paroxysmal atrial fibrillation, subarachnoid hemorrhage, non-alcoholic steatohepatitis, chronic renal failure, COPD, current infection from Covid-19 with bilateral pneumonia and respiratory failure treated with oxygen therapy. ASA status 4. Sleepy, arousable, collaborating patient. Mild tachypnea, 95% SpO2 in oxygen therapy in 40% VMK. Stable hemodynamics. LMWH therapy in progress. We opt for a locorregional anesthesia with femoral and femorocutaneolateral nerve block with ultrasound guide and ENS. Ropivacaine 0.5% 120mg and Mepivacaine 1.5% 300mg. The sciatic nerve was not elicitable from ENS, so a trauma injury was suspected. No pain to mobilization maneuvers. The duration of the intervention was 60 minutes. Pain was noted at the end, and 4h 12h and 24h after surgery. VAS score was 1-1-2-1, respectively. The patient was discharged on the fourteenth postoperative day. Informed consent to publish had been obtained.

Conclusion

The approach performed in this case report identifies the possibility of having a less invasive and impacting anesthesic approach than a general or subarachnoid anaesthesia, especially in debilitated patients or in whom it is contraindicated. A recent review confirms the early movement and the reduction of complications with the use of the analgesic block, perioperative delirium decreasing, with a strong recommendation in the use of locorregional anesthesia in hip fracture surgery [3]. A case report describes an hip fracture treated with nerve blocks, but with dexmedetomidine in continuous infusion [4]. The anesthetic management of elderly patients with hip fracture is a challenge. Locorregional anesthesia could be the right answer in super-old patients, but other studies are required.

References

1 Piscitelli P, Neglia C, Feola M, Rizzo E, Argentiero A, Ascolese M, Rivezzi M, Rao C, Miani A, Distante A, Esposito S, Iolascon G, Tarantino U. Updated incidence and costs of hip fractures in elderly Italian population. Aging Clin Exp Res. 2020 Dec;32(12):2587-2593

2 Kanis JA, Odén A, McCloskey EV, Johansson H, Wahl DA, Cooper C; IOF Working Group on Epidemiology and Quality of Life. A systematic review of hip fracture incidence and probability of fracture worldwide. Osteoporos Int. 2012, 23:2239-2256

3 Guay J, Kopp SL. Peripheral nerve blocks for hip fractures in adults. Meta-Analysis Cochrane Database Syst Rev 2020 Nov 25;11(11)

4 Ishida Y, Ogura F, Kondo S, Toba Y. Successful peripheral nerve block under dexmedetomidine sedation for femoral neck fracture fixation in a 97-year-old patient. Case Reports BMJ Case Rep 2021 Apr 26;14(4)

### A55. The impact of hospitalization after major surgery on elderly frail patients: preliminary results from a monocentric study

#### Fracazzini M.^1^, Moretto F.^1^, De Vita N.^1^, Scotti L.^6^, Viarengo V.^1^, Sola E.^1^, Vecchio G.^1^, Jubran Y.^1^, Gentilli S.^4^, Romito R.^3^, Volpe A.^2^, Leigheb M.^5^, Della Corte F.^1^, Vaschetto R.^1^

##### ^1^AOU Maggiore della Carità di Novara dipartimento di Anestesia e Rianimazione ~ Novara ~ Italia, ^2^AOU Maggiore della Carità di Novara dipartimento clinica urologica ~ Novara ~ Italia, ^3^AOU Maggiore della Carità di Novara dipartimento Chirurgia Generale 2 ~ Novara ~ Italia, ^4^AOU Maggiore della Carità di Novara dipartimento di Chirurgia Generale 1 ~ Novara ~ Italia, ^5^AOU Maggiore della Carità di Novara dipartimento di Ortopedia e Traumatologia ~ Novara ~ Italia, ^6^AOU Maggiore della Carità dipartimento di Medicina Traslazionale ~ Novara ~ Italia

###### **Correspondence:** Fracazzini M.

Background

Surgery and anesthesiologic risk are greater in elderly frail subjects and global performance status worsening after major surgery may occur with a large impact on these patients’ and caregivers’ life. Nowadays, 30 percent of surgery is conducted on patients ≥ 70 years old. Primary objective is to describe the impact at three months after hospitalization following major surgery on the performance status of elderly frail patients. Secondary objective is to evaluate the necessity of domiciliary care assistance and re-hospitalization or rehabilitation center admissions.

Materials and methods

This monocentric, prospective, observational study was conducted on patients ≥ 70 years old, undergoing a major surgery in general surgery, urology, orthopedics at Maggiore della Carità University Hospital, Novara, Italy, having a General Practitioner listed in the Local Health Authority of Novara. The Barthel index was used to evaluate patient functional status as activities of daily living (ADLs) and mobility and the mini mental state examination (MMSE) for cognitive status. The variation of the Barthel index and MMSE from prior to surgery to three months after surgery (T3) were evaluated as primary endpoints. Data from May 2020 to January 2022 were collected and the three-month follow-up was concluded in April 2022.

Results

One-hundred patients undergoing major surgery were included, 60 in general surgery, 37 in urology, three in orthopedics. Patients’ median age was 76 (73-81) years and 60% were males. Most of patients were classified as American society of anesthesiologists (ASA) II or III and main comorbidities were hypertension, cardiac and respiratory diseases. Main diagnoses were colorectal and prostate cancer, and surgery was laparoscopic or robotic assisted in 62 cases. Surgery was conducted under balanced general anesthesia in 99 cases, in seven cases associated to a locoregional technique, and mean duration of anesthesia was 300 (±130) minutes. Postoperative analgesia was administered to all patients, mainly with elastomeric pump (64 patients, 66%) or through epidural catheter (30 patients, 31%). Twelve patients were admitted to intensive care unit (ICU) after surgery and 33 experienced some postoperative complications. Prior to surgery, Barthel index was 100 (100-100), 60 (60-60) for ADLs, 40 (40-40) for mobility, and MMSE was 28 (26-29). Twenty-six (31%) patients out of the 85 interviewed at T3, experienced a five-point worsening of the Barthel index, 21 (25%) in ADLs and 12 (14%) in mobility, while MMSE deterioration occurred in 4 (5%) cases. Domiciliary care assistance was activated for nine patients and 14 were re-admitted to hospital or rehabilitation centers. At univariable regression analysis, postoperative complications occurrence and ICU admission were significantly associated with the five-point worsening of the Barthel index, while ICU admission influenced the same deterioration in the mobility section and need for domiciliary care assistance or re-hospitalization.

Conclusions

Uncomplicated major surgery does not impact on frailty scores in elderly patients with good performance status prior to surgery. Nevertheless, when postoperative complications occur, performance status get significantly worse at three months.

### A56. Risk assessment of the occurrence of postoperative delirium (POD) in patients undergoing hip fracture surgery: preliminary results

#### Piersanti A., Ferrone G., Della Sala F., Crupi D., Console E., Caputo C.T., Vergari A., Rossi M.

##### Department of Anesthesia and Intensive Care, Fondazione Policlinico Universitario Agostino Gemelli IRCCS ~ Roma ~ Italia

###### **Correspondence:** Console E.

**Background**

Hip fracture is among the main causes of hospitalization and major surgery in the elderly (> 65 years).

Postoperative delirium (POD) represents one of the principal complications, with a variable reported incidence between 13 and 70%.

POD would be associated in a variable number of patients with the development, or worsening of a cognitive-behavioral deficit, the so-called postoperative cognitive dysfunction (POCD), itself related to dementia.

To date, there are only hypotheses in the literature regarding the possible risk factors associated with the appearance of POD. Early recognition of patients at risk could favour the adoption of a more personalized medical approach to improve patients’ outcome.

Aim of the study was early detection of POD and its correlation with adverse outcomes.

**Materials and methods**

Observational cohort study performed in a tertiary university hospital in Rome, Italy. Preliminary 65 patients > 65 years old, with radiological presence of hip fracture were enrolled between December 2021 and April 2022.

Primary objective of this study was identification of patients with POD and evaluation of associated risk factors. Neuropsychological test Confusion Assessment Method-Intensive Care Unit (CAM-ICU) was administered upon arrival and during the first five post-operative days to evaluate the onset of POD; Montreal Cognitive Assessment (MoCA) was administered upon hospital arrival and before discharge to evaluate the onset of POCD.

Secondary objectives were hospital length of stay and 30-days mortality.

**Results**

Of 65 patients (Table 1), 5 (7%) developed new postoperative delirium confirmed by CAM-ICU test results (mean age 83 ± SD 8 years). Three patients developed delirium immediately after surgery, two patients on the fourth day of hospital stay, with a mean duration of 43 ± SD 20 hours. Preoperative MoCA test score of patients who developed POD was not significantly different from the score of the MoCA test administered at discharge (*P* = 0.564, paired *t*-test), although it suggests a pre-existing condition of cognitive impairment (preoperative mean MoCA score 21 ± SD 4 vs mean MoCA score at discharge 19 ± SD 6). Preliminary evaluation of associated risk factors with multivariable Cox proportional hazard model led to inconclusive results (Likelihood ratio test, *P*=0.1). Among the entire cohort twenty patients (31%) had a preoperative diagnosis of severe dementia and showed temporal-spatial disorientation, confusion and emotional instability with fluctuating course since arrival until discharge. Two (3%) patients showed delirium upon hospital arrival because of concomitant severe head injury; one patient solved the condition while the other patient reported sequalae. Median hospital length of stay was 9 days [interquartile range (IQR) 7-12 days]. Four (6%) patients died within the first 30 postoperative days.

**Conclusions**

POD is a common complication of hip fracture, even if in our preliminary experience, incidence of new insurgence was relatively low (7%). On the contrary preexisting neurocognitive derangement was high (31%) and confirmed the higher risk of complicated outcomes. Use of cognitive functional tests could help the anesthetist to manage perioperatively patients at increased risk.

Further research is needed to promptly recognize these patients and reduce POD incidence.

**Table 1 (abstract A56).** See text for description

Characteristic	Total patients (N=65)
Age, years	83 ± 8
Height, cm	164 ± 7
Weight, Kg	65 ± 12
Body mass index, Kg/m^2^	24 ± 4
Female	47 (72)
Male	18 (28)
Level of education:	
None	9 (14)
Elementary school diploma	17 (26)
Lower secondary school diploma	9 (14)
High school diploma	22 (34)
Degree	8 (12)
ASA status:	
1	2 (3)
2	25 (38)
3	36 (56)
4	2 (3)
Medical history:	
Cardiovascular diseases	50 (77)
Permanent atrial fibrillation	11 (17)
Previous coronary artery revascularization	5 (7)
Chronic kidney disease	6 (9)
Diabetes	8 (12)
Hepatic diseases	2 (3)
Neurologic diseases	20 (31)
Major depression	4 (6)
Muscular dystrophy	1 (1)
Parkinson disease	2 (3)
Previous stroke/cerebral hemorrhage	4 (6)
Vascular dementia	9 (14)
Pulmonary diseases	19 (29)
Severe hearing loss	15 (23)
Medical history:	
β-blockers	19 (29)
Angiotensin-converting enzyme inhibitors	39 (60)
Anticoagulants/Antiplatelet agents	31 (47)
Diuretics	25 (38)
Statins	19 (29)
Oral hypoglicemic agents	4 (6)
Insulin	2 (3)
Type of fracture:	
Head/Neck femoral fracture	30 (46)
Pertrochanteric femoral fracture	35 (54)
Type of surgery:	
General anesthesia	30 (46)
Subarachnoid analgesia	35 (54)
Suprainguinal fascia iliaca/femoral nerve block	35 (54)
Time from hospital arrival to surgery, hours	41 (34-48)
Hospital length of stay, days	9 (7-12)
30-day mortality	4 (6)
ADL score	5 ± 1
IADL score	5 ± 3
Preoperative MoCA test score of patients who developed POD (N=5)	21 ± 4
MoCA test score at discharge of patients who developed POD (N=5)	19 ± 6
Preoperative MoCA test score of patients who did not develop POD (N=38)	22 ± 5
MoCA test score at discharge of patients who did not develop POD (N=38)	23 ± 5

Demographic, Baseline, Surgical and Cognitive Characteristics of the Study Population. Data are presented as N (%), mean ± standard deviation or median (interquartile range)

*ASA* American Society of Anaesthesiologists, *ADL* Activities Daily Living, *IADL* Instrumental Activities Daily Living, *MoCA* Montreal Cognitive Assessment, *POD* Postoperative Delirium

### A57. Proximal femur fracture in the fragile patient: case report on relevance to SIAARTI good practices

#### Pieri S., Pepe M.

##### Azienda usl Toscana Sud Est Dipartimento Emergenza Urgenza UOC Anestesia e Rianimazione ~ Arezzo ~ Italy

###### **Correspondence:** Pieri S.

Background: as resalt of world population ageing, hip fractures in the elderly have become a relatively frequent event with significant consequences in terms of morbidity and socioeconomic impact. As supported by epidemiological data, in order to improve the quality of care, the outcome and reduce costs, it is necessary to establish a different approach from the traditional one with a systematic multidisciplinary collaboration that takes care of the patient from the entrance to the emergency room and follows him throughout the perioperative path way until rehabilitation.

Clinical case: we report two clinical cases comparing two patients with intertrochanteric fracture subjected to internal fixator treatment but according to a different perioperative procedure. The first, F.M. 84 years old with a recent episode of delirium and disorientation during hospitalization for heart failure, subjected to early pain control with femoral nerve block performed directly in the ER and repeated in the operating room at the time of surgery, operated within 24 hours from admission to the hospital without further specialist advice as indicated by SIAARTI good practices; the second, G.I. 88 years old, history of ischemic heart disease, arterial hypertension and initial cognitive decline, hospitalized in a medical setting to perform a cardiological consultation, treated with pain reliever therapy only at the patient's request and operated after 5 days of hospitalization.

Results: in the first one, we witnessed a regular post-operative course without complications despite the high risk of the onset of delirium, timely management by the multidisciplinary team and hospital discharge to a rehabilitation center after three days of hospitalization in orthopedics setting. In the second one, the preoperative wait led to perform two blood transfusions to control acute anemization, the development of a state of delirium that made the patient incapacitated further delaying surgery and finally the appearance of bedsores in the sacral area due to the patient's lack of cooperation in initiating rehabilitation with passive mobilization.

Conclusions: in the frail patient with a hip fracture, good pain control from the early stages of hospitalization, the timeliness of surgery within a maximum of 48 hours from hospital admission and patient management by a multidisciplinary team, reserving the use of specialist advice only in cases of acute organ failure, limits the incidence of postoperative complications, reduces the length of hospitalization and promotes a more rapid post-operative recovery limiting the risk of developing disability.

Informed consent to publish had been obtained.

### A58. Major abdominal surgery in patient with idiopathic pulmonary fibrosis: case report

#### Frezza F., Coppolino F., Visconti A., Passavanti M.B., Pota V., Sansone P., Pace M.C.

##### Università degli studi della Campania Luigi Vanvitelli Dipartimento Della Donna, Del Bambino E Di Chirurgia Generale E Specialistica ~ Napoli ~ Italy

###### **Correspondence:** Frezza F.

**Introduction**

Idiopathic pulmonary fibrosis (IPF) is a chronic, progressive disease characterized by fibrotic replacement of lung interstitial tissue with multifactorial immune-inflammatory etiopathogenesis (1). In the management of patients with IPF, in anticipation of major surgery, risk stratification of perioperative of respiratory (acute respiratory worsening, exacerbation, postoperative pneumonia, pneumothorax) and non-respiratory (acute heart failure, pulmonary hypertension, acute myocardial infarction, thromboembolism) complications plays a key role (2). Case presentation: In April 2022 comes to the attention of the U.O.C. of Colon-Rectal Surgery of the University of Campania "L. Vanvitelli", a 58-year-old patient with a past medical history of IPF in treatment with Nintedanib for 5 years reporting the onset, in the last month, of abdominal pain and diarrhea related to the increase in the dosage of the drug. This symptomatology is associated to rigid abdomen and pain on palpation. On CT scan of abdomen and chest, bowel perforation is noted with indication for emergency surgery. In the operating room, subarachnoid anesthesia (T8-T9) with a Whitacre 27 G needle with Bupivacaine 10 mg, Ropivacaine 5 mg, Morphine 100 gamma, Clonidine 20 gamma is administered; an epidural catheter (T12-L1) is also positioned for possible rescue therapy in case of prolonged surgical time and for postoperative pain management. Both procedures are performed with no complications. The patient maintains spontaneous breathing during the procedure with Venturi Mask (MoV) (3 L/min; FiO2 30%) and is sedated with Dexdor under continuous infusion at a dose of 0.7 mcg/kg/h (RASS -1). At the end of surgery, the patient is admitted to the ICU for postoperative monitoring; during her stay, she continues oxygen therapy by MoV with FiO2 30 % (SpO2 92-94 %). For postoperative analgesia support, Ropivacaine (0.15 %, 10 ml/h) is administered via peridural catheter, with recorded Numeric Rating Scale values in the postoperative 12h not >4. The patient is transferred after 72h to Colo-Rectal surgery unit. Discussion: Major abdominal surgeries performed under general anesthesia in patients with IPF can result in the occurrence of several complications; therefore, a proper preoperative setting is important in order to identify the anesthesiologic procedure with the best risk/benefit ratio, taking into consideration preoperative respiratory performance, disease activity status, and comorbidities that may adversely affect the outcome. Conclusion: In our clinical case, given the urgent nature of the surgery and the absence of relative and absolute contraindications to the performance of neuro-axial anesthesia, the option of combined spinal-epidural anesthesia allowed the patient to be maintained in spontaneous breathing without resorting to invasive ventilation, drastically reducing the risk of postoperative respiratory complications.

Informed consent to publish had been obtained. If consent had not be obtained then the abstract should be removed from the supplement.

**BIBLIOGRAPHY:**

1. Martinez FJ, Collard HR, Pardo A, Raghu G, Richeldi L, Selman M, Swigris JJ, Taniguchi H, Wells AU. Idiopathic pulmonary fibrosis. Nat Rev Dis Primers. 2017 Oct 20;3:17074. doi: 10.1038/nrdp.2017.74. PMID: 29052582.

2. Carr ZJ, Yan L, Chavez-Duarte J, Zafar J, Oprea A. Perioperative Management of Patients with Idiopathic Pulmonary Fibrosis Undergoing Noncardiac Surgery: A Narrative Review. Int J Gen Med. 2022 Feb 23;15:2087-2100. doi: 10.2147/IJGM.S266217. PMID: 35237071; PMCID: PMC8882471.

### A59. Postoperative delirium (POD) in patients undergoing major abdominal surgery: an observational study

#### Calonaci G., Angeli E., Piccolo C.V., Cappellini E., Zoppi L., Di Silvestro I., Romagnoli S., Baldini G.

##### School of Anaesthesia, Critical Care and Pain medicine, University of Florence, Florence, Italy. ~ Firenze ~ Italy

###### **Correspondence:** Calonaci G.

Background The incidence of PostOperative Delirium (POD) in patients undergoing major surgery is steadily increasing due to their progressive aging and frailty [12] with a reported incidence up to 84% depending on age and type of surgery. Prevention strategies play a pivotal role in the management of this severe complication and the strict application of Enhanced Recovery After Surgery (ERAS) programs has been demonstrated to be an important aid for POD prevention. The aim of this observational single-center study is to investigate the incidence of POD, related risk factors and post-operative complications in patients undergoing major abdominal surgery under ERAS protocols.

Materials and methods Patients aged≥60 have been enrolled if planned duration of anesthesia was 200 min or longer. Core items of ERAS protocols included: opiod-sparing multimodal analgesia, no premedication, processed electroencephalographic monitoring (pEEG[3])-guided total intra-venous anaesthesia, minimally invasive surgery, minimal use of drainages, early. Neurocognitive testing and anamnesis were recorded preoperatively. Confusion Assessment Method (CAM) and Confusion Assessment Method Intensive Care Unit (CAM-ICU) were administered twice or trice/day at post-operative day 1 to 5.

Results A total of 99 patients were enrolled and 6 of them developed POD (6.1%) during the first 5 post- operative days. Patients who developed POD had a preoperative impairment based on the score obtained at the Mini-Mental State Examination [(POD) 23.5 ± 5.3 points vs (NPODS) 30.2 ± 25.9 points; p=0.04] and a pathological performance of the Short Blessed Test (SBT) in comparison with non-POD (NPOD) patients. Unwanted/accidental burst-suppression at pEEG was significantly longer in POD vs. NPOD patients [(POD) 4 minutes vs (NPOD) 2 minutes; p=0.008]. A second evaluation of neurocognitive test was performed on postoperative day 5. A worst SBT was found in POD vs NPOD patients [(POD) 4 ± 3.3 points vs (NPOD) 1.1 ± 1.7; p < 0.01] together with a worsened Timed Up and Go Test (p=NS). Noteworthy, patients with no POD showed early mobilization after surgery defined as within 48 hours after surgery [(POD) 2 patients (33.3%) vs (NPOD) 74 patients (79.6%), p=0.009]. Patients that devoleped POD have shown an higher incidence of post-operatory complications (i.e. cardiovascular accidents, strokes, etc) [(POD) 3 patients (50%) vs (NPOD) 7 patients (7.5%); p=0.01).

Conclusions The observed incidence of POD in patients undergoing major abdominal surgery under strict ERAS protocols was lower than expected. In patients developing POD a worsening of performance in postoperative SBT compared to the preoperative one, a delayed mobilization, and a higher incidence of complications were observed. The patient’s individual predisposition and frailty, mostly nonmodifiable preoperative risk factors, significantly affect the development of POD.

**References**

1. Maldonado JR. Delirium in the Acute Care setting: characteristics diagnosis and treatment. “Crit Care Clin.” 2008;

2. European Comission. Population Ageing in Europe. Vol 19, 2011;

3. Wildes TS, Mickle AM, Abdallah A Ben, et al. Effect of electroencephalography-guided anesthetic administration on postoperative delirium among older adults undergoing major surgery the engages randomized clinical trial. “JAMA- J Am Med Assoc.” 2019

### A60. Locoregional anesthesia in fragile patient: a valid alternative

#### Cappelletti C., Palomba C., Nardi E., Pavani R., Betti D.

##### U.O. Anestesia e rianimazione, Ospedale San Donato ~ Arezzo ~ Italy

###### **Correspondence:** Cappelletti C.

Introduction

In Italy it is estimated that 12,000 new cases of proximal femur fracture occurred per year, thus representing one of the main social and health problems in developed countries. As anesthetists we face with extremely critical situations because they are mainly "fragile" patients. Furthermore, the latest studies highlight how a delay in the surgical approach (beyond 24/48 h) is associated with an increase in mortality and morbidity rate. We therefore implemented an experimental protocol to perform femoral surgical osteosynthesis using exclusively locoregional anesthesia in 30 patients defined as "fragile" in whom neuraxial anesthesia was contraindicated.

Patients and Methods.

The selection included patients with ASA 3/4; NYHA 3/4; NHFs 6/7; METs <4; altered coagulation pattern and contraindicated subarachnoid anesthesia. After adequate monitoring (NIBP; SpO2; HR, EKG), in supine position, PENG block, femoral nerve block, lateral femorocutaneous nerve block are performed under US-guide in asepsis.

LA: Ropivacaine 0.375%, total volume 35/40ml with Dexamethasone 4 mg as adjuvant.

PENG BLOCK : 80-100mm needle, convex probe, LA 25 ml

FEMORAL NERVE BLOCK : 50mm needle, linear probe, LA10 ml

LATERAL FEMORO-CUTANEOUS NERVE BLOCK : 50mm needle, linear probe, LA 5 ml.

Results

The items considered are: NRS/ PAINAD (t0, 24h, 48h); cardiovascular parameters; intraoperative sedation/analgesia; postoperative delirium; PONV; analgesics in PACU. All patients showed perioperative hemodynamic stability, excellent analgesic control at 48h after surgery (26/30), only 4 patients received rescue dose of Paracetamol 1gr in the immediate postoperative period, minimal intraoperative sedation was required for 3 patients, and no cases of PONV and/or delirium occurred.

Conclusions

The proposed protocol is a possible alternative to general anesthesia in all those patients with surgical indication after femoral fracture in whom neuroaxial anesthesia cannot be performed. The results in terms of reduced timing, analgesia, PONV and delirium prevention have been positive. In accordance with ERAS protocols and SIAARTI Good Clinical Practices, it was shown that where subarachnoid anesthesia is contraindicated, performing echoguided nerve blocks is the most appropriate choice, which is also associated with safety and efficacy.

### A61. Emergency and deferrable urgency surgery in patients with COVID-19 infection at a spoke COVID / no COVID center: cases report

#### Salvatelli M., Liguori G., Angellotti L., Ciceraro S., Sucre M.J., Ragone R., Balia M., Cesarano N.

##### Anesthesia and Intensive Care Department. San Leonardo Hospital ~ Castellammare di Stabia ~ Italy

###### **Correspondence:** Ragone R.

It is of great importance to strengthen infection control and the protection of medical personnel in hospitals through the institutional adoption of precise and well-established plans for surgical procedures of choice and those that cannot be postponed and emergencies on patients who are positive for COVID-19 and suspected of covid. For COVID-19 positive patients who require deferred and urgent acute surgical care, a dedicated treatment path must be implemented with protocols and training of the workforce.

Contextualizing the ministerial indications, the recommendations of the scientific societies and the guidelines of the hospitals on the subject is challenging in a non-covid spoke center which also provides assistance for the covid patient.

38 patients were operated on from March 18, 2020 to April 1, 2022. The interventions carried out concern gynecological surgery (caesarean sections, scrapings) orthopedic surgery (Fractures of the femur) and emergency surgery (ruptured spleen, inguinal hernias, Turb and nephrostomy).

For interventions, the operating room is prepared with the material, equipment, electro-medical devices and procedures necessary for the intervention with the movement of unnecessary equipment. Following the internal Covid path defined for this procedure, the patient is transported on a biocontainment stretcher inside the operating room and an anesthetic-surgical team reserved for treatment, minimizing the exposed personnel. The airways were protected by a closed circuit and intubation performed with a video laryngoscope using the transparent protective box and HEPA filter in the ends of the tubes and breathing circuits.

Suction devices have been widely used to remove body fluids and smoke to prevent aerosolization. The medical staff involved complied with the tertiary protection regulations and wore full PPE. At the end of the operation, the patient is discharged with a mask or intubated and connected to a dedicated transport ventilator, with a HEPA filter in the ends of the respiratory circuit, in a biocontainment stretcher following the dedicated path in Intensive Care for postoperative care in a negative pressure room o in the multidisciplinary Covid area with dedicated nursing staff. The reusable materials were decontaminated, washed / dried and / or disinfected / sterilized and the surfaces were sanitized by personnel equipped with PPE.

The operating room and adjacent area were isolated and the patient's transit, including the elevators, were sanitized immediately after the passage with hydrogen peroxide and silver salts. The waste, the PPE used and the soda lime were sealed in hazardous waste containers and properly disposed of. Fluid and tissue samples sent to the laboratory were adequately isolated and transported. The staff involved used the undressing room available in the operating room, Intensive Care. We waited the time necessary for the safety of the whole route. Management foreseen by the company PDTA and shared by the team.

Bibliography

1. PDTA Covid- 19. Delibera Aziendale Asl Napoli 3 sud N.869 del 22/09/2021 pag 81-88

2. Coccolini F et all. World J Emerg Surg. 2020;15:25

3. Buone pratiche cliniche fase 2 covid 19. 20.05.2020. Siaarti

## Bioetica

### A62. Humanization in intensive care in COVID-19 era: experience of a multiple-bed rooms COVID-19 ICU for patients’ relatives access to intensive care unit

#### Iachi A.^1^, Riforgiato C.^1^, Insorsi A.^1^, Dameri M.^1^, Molin A.^1^, Politi G.^1^, Rossetti G.^1^, Benzini A.^2^, Macori L.^2^, Contu M.^2^, Gratarola A.^3^, Orengo G.^4^, Pelosi P.P.^2^, Patroniti N.A.^2^

##### ^1^IRCCS Policlinico San Martino, Clinica Anestesiologica Terapia Intensiva ~ Genova ~ Italy, ^2^Department of Surgical Sciences and Integrated Diagnostics (DISC), University of Genoa ~ Genova ~ Italy, ^3^Anesthesia and Intensive Care, San Martino Policlinico Hospital-IRCCS for Oncology and Neurosciences ~ Genova ~ Italy, ^4^Policlinico San Martino University Hospital, IRCCS for Oncology and Neuroscience ~ Genova ~ Italy

###### **Correspondence:** Iachi A.

Background

During the last decades, patients’ relatives have been progressively more involved in the process of patients’ care among intensive care units (ICUs). SARS-CoV-2 pandemic upset this paradigm, making ICU environment not accessible to patients’ relatives.

Since the first pandemic wave, guaranteeing patients-to-relatives interaction has proved to be challenging, partially substituted with digital devices.

Nevertheless, limitation to relatives’ visits and its consequences on care and outcomes still represents an unsolved problem in COVID-19 units.

On the 7th of July 2020 a new department for COVID-19 patients became active at the IRCSS San Martino Hospital, adapting rooms of a ward previously dedicated to a cardiac surgery ICU.

During the period between March and December 2020, in accord with Hospital Health Management, operative procedures were implemented in order to both allow patients to be visited by their relatives and involve relatives in patients’ end-of-life care.

Methods

The ICU ward had 10 beds arranged in two two-bed rooms and one six-bed room characterized by an open access (virtual filter) on the common area and large windows on aisles outside. In addition, two rooms previously used as operating theatres were used as a joint common area.

The following strategies and goals were set up for optimization of rooms use: 1) implementation of COVID-free rooms, by patients distribution in different rooms on the basis of negativization ofnasopharyngeal swab tests for SARS-CoV-2 PCR or the presence of other infectious diseases transmitted by physical contact, 2) frequent rooms disinfection using vaporization techniques, 3) relatives access to the common Covid-free area and to rooms patients with negative SARS-CoV-2 test by scheduled visits, 4) re-location of end of life patients with negative SARS-CoV-2 test in a specific operating room to allow relatives visits of their loved ones during end-of-life process without time limits.

Patients’ relatives followed the same DPI wearing Hospital procedures valid for healthcare staff.

ICU staff got in touch with patients’ relatives some days after the ward access in order to check over potential SARS-CoV-2 infection.

Results

In the period between July 2020 and March 2022, 150 patients with positive SARS-CoV-2 PCR test have been admitted in ICU.

The ward opening project has fully reached its functionality on December 2020, meeting all the intended aims. From January 2021 till now, the ward operative project for patients’ relatives have been functional without interruptions and 94 patients have been involved.

All patients’ relatives who were offered the opportunity to participate during end-of-life process accepted the ward attendance (23 patients).

Relatives’ feedback about the ward access project was markedly positive.

Potential infection spreads for SARS-CoV-2 have not been registered between relatives who accessed the ICU.

Conclusions

The multiple-bed rooms organization and the availability of “reservoir” areas committed to the patients’ relocation and end-of-life management demonstrated to be convenient in order to allow ICU re-opening for patients’ relatives, without increasing the risk of infection spreading. In addition, the possibility to manage both negativized and positive patients avoided interruptions in the process of health management.

## Cardio-Toraco-Vascolare

### A63. Multimodal analgesia in thoracic surgery

#### Patrone V., Nanni B., Basso N., Fontanarosa L., Travaglia C., Angeli E., Baldini G., Romagnoli S.

##### Scuola di Specializzazione, Università di Firenze; Azienda Ospedaliero-Universitaria Careggi, Firenze ~ Firenze ~ Italia

###### **Correspondence:** Basso N.

Introduction: Post-operative pain exacerbates patient discomfort and is responsible for many pulmonary and cardiac complications, slow recovery and mobilization. (Enhanced Recovery After Surgery)

1recommends a multimodal approach that combines systemic and regional analgesia techniques to better control pain and reduce the incidence of collateral effects from drug use.

Aim: Our study aims to evaluate the analgesic effect of multiple regional analgesia techniques in patients that undergo elective thoracic surgeries with a thoracotomy approach or videothoracoscopy approach (VATS)2. Materials and methods: This study is an observational, prospective, monocentral study. We enrolled patients that underwent open and videothoracoscopy thoracic surgeries. We evaluated the incidence of post-operative pain in patients where different kinds of analgesia techniques were applied in and out of the operating room. We have registered clinical data through RedCap database.

Results: We have enrolled a total of 113 patients of which 43 underwent a thoracotomy procedure and 70 a VATS procedure. In the first group we compared the post-operative pain in patients with a peridural catheter (PERI) and in those without (N-PERI) that received other regional analgesia techniques such as ESP-block (ESP-b)3 or the intercostal nerve block (BI) or local anesthetic infiltration of the wound (AL). The results have demonstrated statistical differences among the two groups in terms of static NRS (Numeric Rating Scale) over the first 24 hours in those without a peridural catheter that showed a higher NRS (p-value = 0,04). Among the N-PERI group patients that have received either BI or AL showed a higher pain intensity 24 hours after surgery compared to those who had received ESP-b (p-value =0,04).

Among the VATS group, the results show that the NRS score in those who have received ESP-block is statistically and significantly lower than in those who have received other regional techniques, especially in controlling the pain at 24 hours after surgery. Moreover, even though not statistically significant, patients in the BI and AL group, had a higher incidence of requesting rescue analgesia compared to those who have received an ESP-b.

In this study, we have not demonstrated statistically significant differences when comparing the N-PERI group and the VATS group. In addition, our multimodal analgesia protocol has demonstrated a greater clinical efficacy in thoracotomy surgeries and VATS surgeries.

Conclusion: Considering the reduced sample size of our study, the analgesic protocol seems to be effective.

The pain felt by patients with these regional analgesic techniques in open surgeries and with the placement of a peridural catheter or the ESP-block with other adjuvants is comparable. In VATS, instead, ESP-block seems to have a higher efficacy than BI or AL.

### A64. Effectiveness of near-infrared spectroscopy (NIRS) monitoring during carotid endarterectomy (CEA); comparison with transcranial doppler (TCD). Retrospective analysis at varese hospital asst-settalaghi

#### Novazzi C., Guzzetti L., Toso F., Selmo G., Carollo M., D”Onofrio D., Cozzi S., Ghislanzoni L., Bacuzzi A.

##### Asst Settelaghi Ospedale di Circolo S.C. Anestesia e Gestione Blocchi Operatori ~ Varese ~ Italia

###### **Correspondence:** Novazzi C.

Background

The introduction of neuromonitoring during CEA reduced the intraoperative incidence of stroke and prevented acute intraoperative embolism. The aim of neuromonitoring is to detect cerebral hypoperfusion during carotid clamp and to implement adequate periprocedural actions. We perform an investigation about the role of NIRS during CEA, in comparison with TCD, to identify perioperative adverse neurological events.

Materials and Methods

The study included patients undergoing CEA at Ospedale di Circolo Fondazione Macchi Varese ASST Settelaghi. During carotid clamping we recorded the percentage of maximum fall in cerebral saturation, registered trough NIRS ( Medtronic, INVOS ), and the V-MCA reduction, registered trough TCD ultrasound, compared to the baseline value. The cerebral hypoperfusion was defined as V-MCA reduction more than 50% compared to baseline and a decrease in NIRS value greater than 20% compared to baseline. We registered the intraoperative shunt positioning. We defined postoperative neurological events : acute mental disorders, convulsions, focal neurological deficits all accompanied by neuroradiological signs on CT scan or magnetic resonance.

Results

We enrolled 66 patients. 76% of the surgical procedures were performed in loco-regional anaesthesia. We compared the NIRS monitoring accuracy to TCD during carotid clamping. Cerebral hypoperfusion occurred in 6 patients (9%) with a reduction more than 50% in V-MCA; the selective carotid artery shunting was placed intraoperatively in 100% of these patients. 50% of these six patients were on loco-regional anaesthesia and showed sensory changes, which resolved after shunt placement. During clamping, the ipsilateral rSO2 decreased by 30.7% +/- 3.5 compared to the baseline values. Neurological adverse events in the first 7 postoperative days occurred in 3 patients (4.5%): 33.3% of these patients recorded a decrease >50% in V-MCA and >20% in NIRS compared to baseline.

Conclusions

The analysis of our data shows that NIRS monitoring is comparable to TCD to identify cerebral hypoperfusion during CEA. Therefore its use is recommendable and effective. The intraoperative NIRS and TCD values alterations allow an accurate identification of patients who may develop adverse neurological events in the postoperative period. The small population analysed and the lack of intraoperative data, such as blood pressure management and oxygen saturation, are the main limits of this study. Further analysis are necessary.

### A65. Can pancreatic stone protein predict perioperative infection in cardiac surgery?

#### Raimondo P.^2^, De Palma F.^2^, Fiorentino M.^1^, Lenoci S.^2^, Rubino G.^2^, Stripoli A.^2^, Armenise A.^2^, Villani M.A.^2^, Colantuono G.^2^, Fiore G.^2^, Grasso S.^2^

##### ^1^UOC Nefrologia Universitaria - Policlinico di Bari ~ Bari ~ Italia, ^2^Dipartimento di Anestesia e Rianimazione II - Policlinico di Bari ~ Bari ~ Italia

###### **Correspondence:** Raimondo P.

BACKGROUND AND INTRODUCTION

The Surviving Sepsis Campaign described the sepsis’s process as a “life-threatening organ dysfunction caused by a dysregulated host response to infection”.

The resulting organ dysfunction is transitory and self-terminating only if the patient’s immunitary system is able to compensate, otherwise it’s possible that the patient worses and complications may occur. As consequence of this pathological mechanism, a patient underwent on cardiac surgery can present postoperative cardiac dysfunction, respiratory failure, renal and neurological dysfunction, bleeding disorders, altered liver function and systemic inflammatory response as multi-organ failure. Early identification and appropriate management after the development of inflammatory response improve outcomes.

PSP is a protein produced by the during early stages of the development of sepsis, so we want evaluate if Pancreatic Stone Protein (PSP) can predict perioperative infection in cardiac surgery.

MATERIALS AND METHODS

This case series was conducted in our adult cardiothoracic surgery Intensive Care Unit (ICU) at the Teaching Hospital “Policlinico di Bari” in January 2022. Informed consent for retrospective data evaluation was obtained from all patients or their relatives. After the admission, all patients were approached by a resident anesthesiologist, their compliance with inclusion criteria [ASA < 4; age > 18] and exclusion criteria (inability or unwillingness to give informed consent, ASA > 5) was checked and the request finalized.

At admission, baseline blood samples were taken for analysis of PSP, CRP, WBC and other preoperative routine parameters. All other data were collected in a dedicated database and evaluated using R Studio.

RESULTS

For this brief case series, we enrolled 5 patients scheduled for myocardial revascularization. All patients are men with a mean age of 66,8 years, 2 or more risk factors, 6 ± 5,7mean days of length of stay and favorable outcome. Only two patients returned to the ICU. PSP was evaluated in the study population and in singular case in Figure 1.

DISCUSSION AND CONCLUSION

Since 1980 surgical trauma, cardio pulmonary bypass, ischemia-reperfusion injury and release of inflammatory factors have been well documented to induce a complex inflammatory response, so the only useful factor for early treatment of sepsis development is a good marker. In our brief case seires PSP confirms the postoperative inflammatory reaction (Fig 1). This novel marker showed high levels (> 200 ng/ml) in the only 2 patients that returned to the ICU and in only two patients that developed an important inflammatory response after cardiac surgery or for risk factor (end-stage renal disease undergoing dialysis), (Fig. 1). Further research is needed about sensibility, specify and timing, but PSP can become an early marker for postoperative sepsis.

REFERENCES

1 - Prazak , Irincheeva , Llewelyn , Stolz, de Guadiana Romualdo, Graf, Reding, Klein, Eggimann and Ai Que; Accuracy of pancreatic stone protein Open Access for the diagnosis of infection in hospitalized adults: a systematic review and individual patient level meta-analysis. Crit Care (2021) 25:182

**Fig. 1 (abstract A65).**
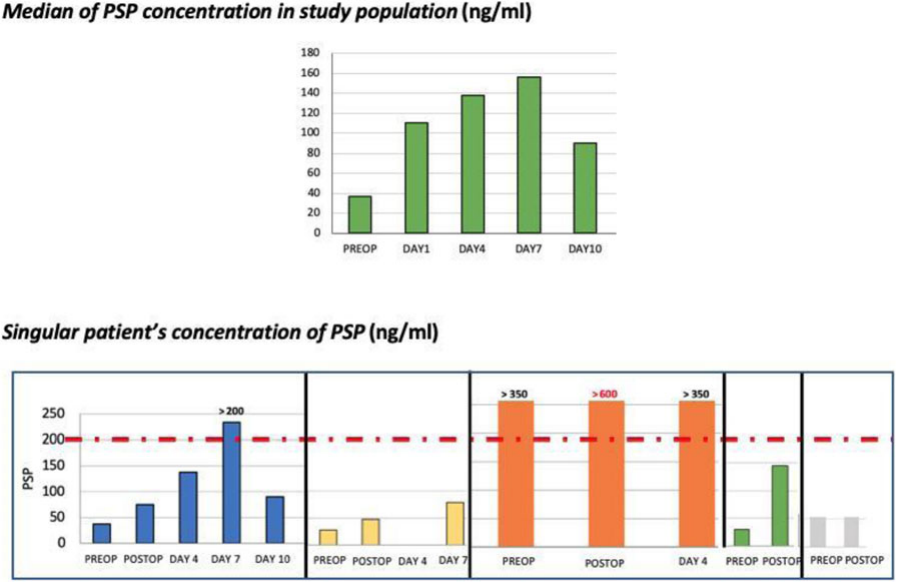
See text for description

### A66. Cardiogenic shock: etiology and treatment with inotropes and vasopressors

#### Battistini L., Colombo C.N., Erba M., Mojoli F., Tavazzi G.

##### Anesthesia and Intensive Care, Fondazione IRCCS Policlinico San Matteo ~ Pavia ~ Italia

###### **Correspondence:** Battistini L.

Background

The term cardiogenic shock identifies a syndrome determined by a primarily cardiac dysfunction with reduced cardiac output and consequent tissue hypoperfusion, which can result in multi-organ failure and patient death [1,2].

Despite recent developments in the diagnosis and treatment, in-hospital mortality ranges from 30% to 60% with about half of deaths within 24 hours [3].

Another aspect to consider is that of the etiological categories. Several years ago, about 81% of cardiogenic shocks were caused by acute coronary syndrome [4], however more recent evidence describes a significant reduction in the rate of cardiogenic shocks caused by acute coronary syndromes, with an increase in non-ischemic causes (35-40%) [5].

Pharmacological therapy with inotropes and vasopressors represents a fundamental aspect, but there is an important lack of consensus among guidelines about their use [6].

The goal of our study is to evaluate the mortality of patients hospitalized with ischemic versus non-ischemic cardiogenic shock and investigate the association inotropic score-mortality and inotropic score-mechanical circulatory support. We also evaluate the association between inotropic score and etiology of cardiogenic shock.

Materials and methods

Retrospective observational study considering patients admitted to the Intensive Care Department of the I.R.C.C.S. Policlinico San Matteo, Pavia, diagnosed with cardiogenic shock >18 years of age. All data about in-hospital outcome, vital signs (at admission, after 24h and discharge), vasopressors and inotropes doses (at ICU admission, mean and maximum dose) and organ supports (Mechanical Ventilation, RRT and Mechanical Circulatory Support) were recorded.

Results

41 patients were enrolled (71.4±10.7 years; 34.1% female) with CS; 53.6% died in hospital. Serum lactate at admission, after 3, 6, 24 and 48 hours were significantly high in patients who died. Maximum dose of epinephrine resulted significantly different between dead and dismissed alive patients. However, doses administered were lower than those described in previous cited studies; furthermore, logistic regression showed no correlation between epinephrine administration and in-hospital mortality. In patients who received MCS mean dose of epinephrine was significantly lower than in patients who didn’t. Finally, when indicated, use of MCS could be useful in reducing epinephrine doses.

Conclusions

This work reports a greater exposition to epinephrine with higher maximum dose in dead patients; however, doses here reported are lower than those described in literature. Furthermore, in-hospital mortality, RRT, MV and MCS seem to be independent of epinephrine administration and all other catecholaminergic drugs used.

**References**

1. Chioncel O, Parissis J, Mebazaa A, et al. Epidemiology, pathophysiology and contemporary management of cardiogenic shock-a position statement from the Heart Failure Association of the European Society of Cardiology. Eur. J Heart Fail 2020; 22:1315-1341.

2. Vahdatpour C, Collins D, Goldberg S, Cardiogenic Shock. J Am Heart Assoc 2019; 8: e011991.

3. Chioncel O, Mebazaa A, Harjola VP et al. ESC Heart Failure Long-Term Registry Investigators. Clinical phenotypes and outcome of patients hospitalized for acute heart failure: the ESC Heart Failure Long-Term Registry. Eur J Heart Fail 2017; 19: 1242-1254.

4. Harjola VP, Lassus J, Sionis A, et al. CardShock Study Investigators; GREAT Network. Clinical picture and risk prediction of short-term mortality in cardiogenic shock. Eur J Heart Fail 2015; 17:501-509.

5. Berg DD, Bohula EA, van Diepen S, et al. Epidemiology of Shock in Contemporary Cardiac Intensive Care Units. Circ Cardiovasc Qual Outcomes. 2019; 12: e005618.

6. McDonagh TA, Metra M, Adamo M et al. 2021 ESC guideline for the dianosis and treatment of acute and chronic heart failure. Developed by the Task Force for the diagnosis and treatment of acute and chronic heart failure of the Europe Society of Cardiolody (ESC). With the special contribution of the Heart Failure Association (HFA) of the ESC. Eur J Heart Fail 2021; 42:3599-3726.

### A67. Evaluation of postoperative delirium with CAM ICU score in patients taking selective serotonin or selective serotonin-noradrenergic reuptake inhibitors undergoing to elective -on pump cardiac surgery

#### Zanza C.^1^, Romenskaya T.^2^, Martuscelli E.^6^, Dealessi M.^3^, Virtuani R.^3^, Savioli G.^4^, Savarese B.^2^, Maconi A.G.^5^, Racca F.^2^, Longhitano Y.^1^

##### ^1^Foundation of “Ospedale Alba-Bra” and Department of Emergency Medicine, Anesthesia and Critical Care Medicine, Michele and Pietro Ferrero Hospital, Verduno, Italy ~ Verduno ~ Italia, ^2^Department of Anesthesia and Critical Care Medicine, Azienda Ospedaliera “SS Antonio e Biagio e C. Arrigo”, ~ Alessandria ~ Italia, ^3^Department of Internal medicine Asl AL Ss Antonio e Margherita hospital ~ Tortona ~ Italia, ^4^Department of Emergency Medicine, IRCCS Fondazione Policlinico San Matteo ~ Pavia ~ Italia, ^5^Department of Research and Innovation-Research Training Innovation Infrastructure. Azienda Ospedaliera SS Antonio e Biagio e Cesare Arrigo ~ Alessandria ~ Italia, ^6^Department of Emergency Medicine-Section of Anesthesia and Critical Care St Giacomo Hospita ~ Novi Ligure ~ Italia

###### **Correspondence:** Romenskaya T.

INTRODUCTION

The postoperative delirium (POD) must be differentiated from Emergence Delirium (ED) which is a transient alteration of the mental state in patients who are waking up and has an incidence between 8 and 20%, very frequent in young patients.

SSRIs and SNARIs are widely prescribed in general population. Antidepressant drugs, in particular SSRIs and SNARIs, through a modification of some neuronal biochemical pathways, associated with the effects of CPB (CARDIO-PULMONARY BY PASS) may be the architects of POD, in this case the literature is a bit weak in quantifying and defining the delirium associated with SNARI and SSRI, moreover many studies have included patients with many comorbidities that are themselves risk factors, creating an underlying bias.

Our study wants to demonstrate that SNARI and SSRI can protect against delirium, selecting patients with few comorbidities and excluding those with underlying pathologies that are the source of a clinical bias.

AIM

The main purpose of our study is to evaluate the incidence of POD in on-pump cardiac surgery between 2 populations (SSRI-SNARI on - SSRI-SNARI off) via CAM-ICU SCORE in the 48 hours following awakening in the ICU. Among the various secondary endpoints, the following will also be evaluated:

1. Correlation with ICU-Length of Stay;
2. correlation with Hospital-Length of Stay;
3. bleeding in ml / day;
4. reoperation;
5. request for mechanical ventilation in intensive care;
6. 28 days-mortality;
7. infections.

MATERIALS AND METODS

This project is a non-profit prospective multicenter observational clinical study. The overall duration of the study is 24 months, starting from April 2021. The enrollment of the study will last at least 24 months, starting from the date of enrollment of the first patient. The collected data were reported in the paper Data Collection Form (CRF).

RESULTS

We collected 126 consecutive patients from June 1, 2021 to October 23, 2021 who were afferent to the Hospital of Alessandria. The mean age of the participants was 70.15 years. The study population consisted of 61% male and 39% female. 89% were in ASA 4. The 40% of patients were in the NYHA 3 group (51 patients out of 126), as well as for the NYHA 4 group. A total of 67% (85 patients in the sample) were not on any neurological/psychiatric therapy. Of the sample, 33% (41 of 126 patients) were taking neurological/psychiatric therapy, of these 24% were taking SSRI/SNRIs and 59% were taking benzodiazepines (BDZ). The incidence of POD in patients taking SSRI/SNRI therapy was 30% predominantly on day 0 (67%) vs 20% in patients without any neurological/psychiatric therapy. The incidence of POD in patients taking BDZs was 54% more frequently on day 0 and day 2.

CONCLUSIONS

According to the preliminary analysis, it could be hypothesized that SSRIs/SNRIs may have a protective role in the occurrence of postoperative delirium in patients undergoing on-pump cardiac surgery.

The complete database analysis collected from this multi-center study should be performed to have definitive data.

### A68. Assessment of platelets function with impedance aggregometry in patients undergoing carotid thromboendarterectomy: a preliminary report

#### Barucco G.^2^, La Banca R.^1^, Russetti F.^1^, Cara” G.^1^, D”Amico F.^1^, Sofia R.^1^, Mainieri N.^1^, Monaco F.^2^

##### ^1^Universita' Vita E Salute San Raffaele ~ Milano ~ Italia, ^2^Ospedale San Raffaele ~ Milano ~ Italia

###### **Correspondence:** La Banca R.

**Background:** Ischemic stroke is the third most frequent cause of death and permanent disability. Atherosclerotic emboli from the carotid arteries account for 30% of all ischemic strokes. Carotid thromboendarterectomy (TEA), treating the carotid stenosis, is effective in preventing these thromboembolic events. Notably, 10% of patients undergoing carotid TEA shows transient and permanent thrombosis and/or stroke. Nevertheless, different studies demonstrate that some patients treated with ASA or clopidogrel show inadequate response to these drugs, so called resistance or low-response [1,2].

**Endpoints:** Aim of the present study was to assess the platelet function with the Multiplate ® Analyzer (Roche, Basel, Switzerland) impedance aggregometry in patients undergoing TEA.

**Methods:** We retrospectively analyzed data from 50 patients undergoing carotid TEA at the San Raffaele Scientific Institute between November 2020 and October 2022. The population is depicted in table 1. Before the intraoperative administration of heparin, 1.6 ml of blood was colleted in hirudin-coated tubes. Then 300μl blood was pipetted and diluted with NaCl 0.9% in a 1:1 ratio. Platelet aggregation was determined thereafter in response to stimulation with 20μl of thrombin receptor activator peptide 6 (TRAP test), adenosine-5′-diphosphate (ADP test) and arachidonic acid (AA) and expressed in arbitrary units (Area under the curve [AUC]). In order to test the ability of the of TRAP, ADP and ASPI to predict the primary outcome, we generated a receiver operating characteristic (ROC) curve. The Youden index (sensitivity+specificity-1) was used to find the optimal cut-off values. The AUC and the exact binomial 95% CI were calculated (fig 1).

**Results:** TRAP, ADP and ASPI were effective in predicting the primary outcome with an AUC of 0.857 (95% CI 0.727- 0.940; p<0.001) , 0.853 (95% CI: 0.722- 0.938;p<0.001 )and 0.781 (0.651-0.894;p=0.008) respectively. According to the Youden index, the optimal criterion for TRAP, ADP and ASPI was 150 AUC with 100 % sensitivity and 79% specificity, 69 AUC (sensitivity 100% specificity 72%), 30 AUC (sensitivity 83% specificity 70%).

**Conclusion:** Patients with higher degree of platelets aggregation assessed with TRAP, ADP and ASPI are at risk of thromboembolic events.

Bibliografy

1. Ball STE, Taylor R, McCollum CN. Resistance to Antiplatelet Therapy Is Associated With Symptoms of Cerebral Ischemia in Carotid Artery Disease. Vasc Endovascular Surg. 2020 Nov;54(8):712-717. doi: 10.1177/1538574420947235. Epub 2020 Aug 28. PMID: 32856558; PMCID: PMC7555613.

2. Kazimi AU, Weber CF, Keese M, Miesbach W. The Pre- and Postoperative Prevalence and Risk Factors of ASA Nonresponse in Vascular Surgery. Clin Appl Thromb Hemost. 2021 Jan-Dec;27:10760296211044723. doi: 10.1177/10760296211044723. PMID: 34609920; PMCID: PMC8642110.

**Fig. 1 (abstract A68).**
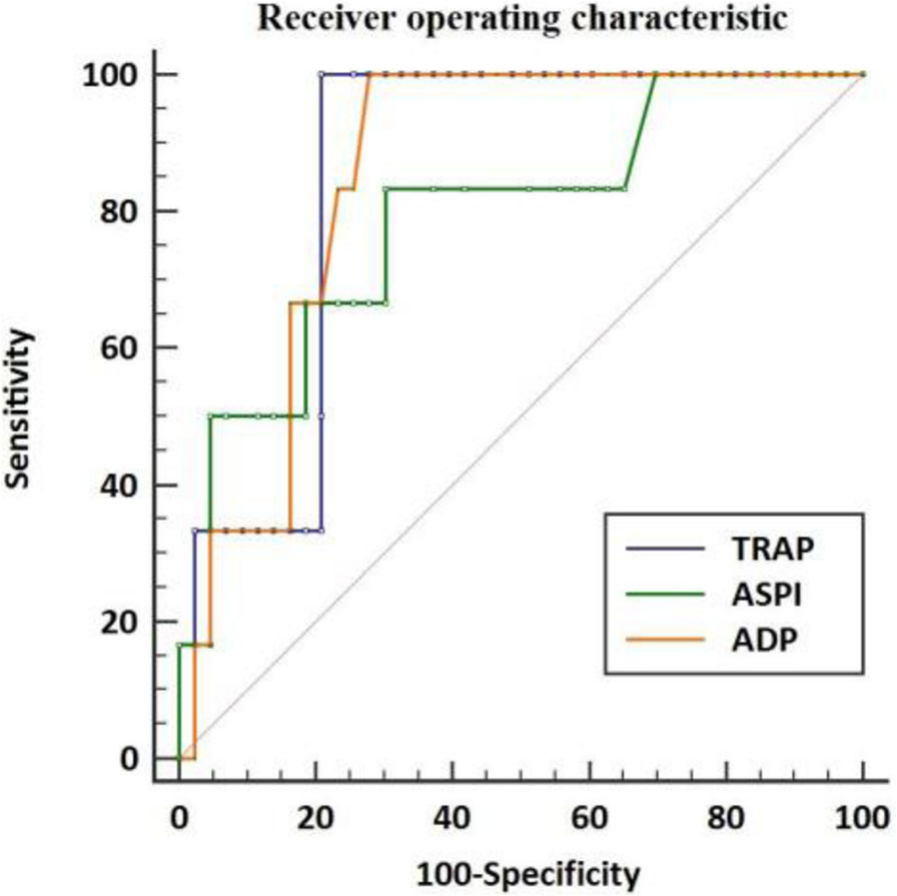
See text for description

**Table 1 (abstract A68).** See text for description

Demographic data	Population
	(n=50)
Age, years	73,5 (± 8)
Male sex, n (%)	32 (64)
Hypertension, n (%)	36 (72)
Diabetes, n (%)	17 (16.4)
Active smokers, n (%)	3 (6)
CKD, n (%)	6 (12)
CAD, n (%)	13 (26)
AF, n (%)	17 (34)
Controlateral stenosis (%)	12(10)
Cretinine mg/dL (%)	1,015 (± 0,42)
Hb g/dL (%)	13,05 (± 2,54)
PLT 10^9/L (%)	216 (±72,8)
Antiplatelets drugs	50 (100)
ASA	28
Clopidogrel	9
ASA+ NOACS	3
DAPT	10

Data are presented as median (±standard deviation) or as n (%)

*CKD* chronic kidney disease, *CAD* coronary artery disease, *AF* atrial fibrillation, *Hb* Hemoglobin, *PLT* Platelets count, *ASA* acetylsalicylic acid, *NOACs* novel oral anticoagulants, *DAPT* dual antiplatelet therapy

### A69. Non-intubated video-assisted thoracic surgery (NIVATS): the experience of Policlinico Umberto I, Rome

#### Brisciani M.^1^, Piazzolla M.^1^, De Paolo D.^1^, Tozzi P.^1^, Zullino V.^1^, Bruno K.^1^, Ferrante F.^2^, Anile M.^2^, Vannucci J.^2^, Venuta F.^2^, Ruberto F.^1^, Pugliese F.^1^

##### ^1^Dipartimento di Chirurgia Generale e Specialistica Paride Stefanini, Anestesia e Rianimazione, Sapienza Università di Roma ~ Roma ~ Italia, ^2^Dipartimento di Chirurgia Generale e Specialistica Paride Stefanini, Chirurgia Toracica, Sapienza Università di Roma ~ Roma ~ Italia

###### **Correspondence:** Brisciani M.

Background: Non-intubated video-assisted thoracic surgery (NIVATS) has aroused interest in the recent decade to minimize invasiveness of anesthetic procedures, encouraging an enhanced recovery after surgery, reducing complication related to general anesthesia, intubation and one lung ventilation (OLV).

We report our preliminary experience in performing NIVATS in a selected cohort of patients.

Methods: Patients with surgical indication to minor thoracoscopic surgery, such as resection of solitary peripheral pulmonary nodules less than 2 cm, pleurodesis, pleural biopsy, pleural decortication and lung apicectomy underwent NIVATS between September 2021 and March 2022 at Policlinico Umberto I, Rome. We included patients with ASA < 3, age between 18 and 85 years, without anticipated difficult airway management and contraindications to perform locoregional anesthesia.

The anesthetic protocol included premedication with Lidocaine 2% 3-5 ml by nebulization 30 minutes before the induction; sedation with preserved spontaneous breathing by propofol 2-4 mg/kg/h and remifentanil 0.05-0.1 mcg/Kg/min continuous infusion administration was applied to achieve bispectral index (BIS) value between 40 and 60 and maintain a physiologic respiratory rate of 10-12 breaths for minute. Supplementary oxygen administration for all the duration of anesthesia and surgery was provided using High-Flow Nasal Cannula (HFNC) with oxygen flow of 40-60 l/min and Fraction of Inspired oxygen (FiO2) titrated to avoid desaturation and hypoxemia. HFNC was replaced by Venturi Mask FiO2 0.5 in the recovery room.

Unilateral ultrasound-guided Erector Spinae Plane (ESP) block at T4-T5 was performed as analgesic strategy using Ropivacaine 0,5% 20 ml and 4 mg desametasone, acetaminophen 1 g iv was administered before the induction and continued 1gr TID in the postoperative period.

Intraoperative and postoperative blood gas analysis data were collected; we evaluated also: duration of surgery, recovery from anesthesia, postoperative fasting time, chest tube duration, recovery of deambulation, perioperative complications, pain evaluation by NRS (evaluated at the admission to recovery room, at 24 h and 48 postoperative hours) and hospital length of stay.

All data are reported as mean ± standard deviation.

Results: Six patients (56,7 ± 23,7 years old) underwent NIVATS.

Blood Gas Analysis showed a stable intraoperative P/F ratio (293 ± 150) with moderate intraoperative hypercapnia (paCO2 61 ± 4 mmHg), pH (7,30 ± 0,03) promptly recovered at the end of the anesthesia (paCO2 46 ± 3 mmHg, pH 7,4 ± 0,02).

Duration of surgery was 37 ± 22 min. Recovery anesthesia time (time between the end of surgery and the admission to recovery room) was 10 ± 3 min. Chest tube duration was 2,3 ± 1,3 days. Postoperative fasting time was 8,3 +- 4,3 hours. Hospital length of stay was 4 ± 2 days. NRS values were 3,8 ± 2, 1,8 ± 1, 1±1 at the admission in recovery room, 24 h and 48 postoperative hours respectively. No perioperative complications or need of conversion to general anesthesia were reported.

Conclusion: Although the small sample size, in our preliminary experience NIVATS seems to be a safe and feasible approach in selected patients, in accordance with literature.

### Abstract 70. Carotid endoarterectomy in awake patients : tollerability and sactisfaction. a case series

#### D”Amicis V.^1^, Cincinelli A.^2^, Rottola C.^2^, Manco E.^2^, Galata” M.^2^, Bergamino M.^2^, Dasso S.^2^, Solari S.^2^, Sivori F.^2^, Bonfiglio M.^2^

##### ^1^UNIVERSITA' degli studi di Genova ~ GENOVA ~ Italia, ^2^ASL4 LIGURE ~ LAVAGNA ~ Italia

###### **Correspondence:** D”Amicis V.

**Introduction**

Carotid endarterectomy (EAC) can be performed

1. under general anesthesia (AG) with instrumental intraoperative brain monitoring and placement of selective endoluminal shunt as needed or with routine endoluminal shunt
2. in local or locoregional anesthesia (ALR) in which intraoperative brain monitoring with selective endoluminal shunt placement as needed (the patient is awake and responds to simple commands).
3. under general anesthesia to cooperating patient (AGPC) which combines the advantages of both types of anesthesia.

For the third anesthetic choice, there is a strong research recommendation regarding patient satisfaction with the AGPC method, as can be seen from the LG "Diagnosis and treatment of extracranial carotid obstructive steno pathology and prevention of cerebral stroke" published in the National Lines System Rome guide, 1 October 2021. The purpose of this study is therefore to describe the tolerability and satisfaction of our patients who underwent carotid endarterectomy under general anesthesia in collaborating patient (AGPC) from January 1, 2022 to May 30, 2022.

**Materials and methods**

Since January 2022 we have applied to all patients treated for carotid TEA the method (AGPC) which involves the use of a general anesthesia, maintained with Remifentanyl, in which the patient is superficialized by hypnosis during the clamping phase of the carotid so to make possible the clinical evaluation of his motor response at the operator's request, combined with the execution of the superficial cervical plexus block to ensure effective post-operative analgesia (Fig. 1).

Anesthetic scheme used:

- Propofol -> 1,0 – 1,5 mg/kg
- Succinilcolina -> 1,0 – 1,5 mg/kg
- Remifentanil -> 0,25 mcg/kg/min (titration as needed)
- Sevoflurano -> 0.20% - 0,25%
- Lidocaina -> 4% spray -> 5 ml
- Blocco -> Ropivacaina -> 0,35 mg
- Anesthesia at the incision site -> Lidocaina -> 2%

Within 4 days of the operation, we submitted to all operated patients the anesthesia assessment survey ("QUALITY CONTROL IN ANESTHESIA FOR CAROTID SURGERY (CAROTID TEA") (Tab.1).

In addition to the standard monitoring of the bloody PA, SPO2, BIS, ECG, for some patients it was possible to use an intraoperative brain monitoring system (bilateral regional cerebrovascular oxygen saturation - INVOS) designed to quantify the hypoxic insult following the clamping phase.

**Results**

We enrolled a total of 14 patients, 2 of whom didn’t receive the survey as they underwent Shunt for clinical reasons.

**Conclusions**

he advantages that can be appreciated from the use of this method are the reduction of the psychological and surgical stress of the intervention for the patient, the possibility of maintaining a favorable hemodynamics and absolute control of the airways by the anesthetist.

References

a) LG “ Diagnosi e trattamento della patologia steno ostruttiva carotidea extracranica e prevenzione dell'ictus cerebrale” pubblicata nel Sistema Nazionale Linee Guida Roma, 1 ottobre 2021

b) Superficial versus combined (deep and superficial) cervical plexus block for carotid endarterectomy.Ivanec Z, Mazul-Sunkol B, Lovricević I, Sonicki Z, Gvozdenović A, Klican K, Krolo H, Halapir T, Novotny Z.Acta Clin Croat. 2008 Jun;47(2):81-6.

**Table 1 (abstract 70).** See text for description

Question	Confort	Disconfort
Before I was taken to the operating theatre I felt:	Sleepy	Agitated/nervous 2/12
	Quiet 10/12	
I was aware of the type of anaesthesia I would receive:	Yes 11/12	No 1/12 (language barrier)
In the operating theatre the environment was:	Comfortable 9/12	Uncomfortable because of:
		Noises
		Smells
		Cold 3/12
		Cold 3/12
		light
		Voices
		More
I found the staff:	Kind/helpful 12/12	Not kind/not available
	Attentive to my needsi 12/12	Not attentive to my needs
	Qualified 12/12	Unqualified
	More	More
In the operating theatre I experienced discomfort due to:	I do not remember feeling any discomfort 8/12	Thirsty 1/12
		Nausea/vomiting 2/12
		Shivers/tremor 2/12
		Difficulty in waking up
		Sleepy legs
		Difficulty in breathing
		Difficulty in speaking 3/12
		Surgical positioning
		Pain during anaesthesia manoeuvres 2/12
The intraoperative awakening provided by the anaesthetic technique adopted was:	Quiet and peaceful 10/12	Traumatic 1/12
	Acceptable 1/12	
In the recovery room, immediately after surgery:	I felt no pain 6/12	I felt a strong pain 1/12
	I felt bearable pain 5/12	I felt unbearable pain
I received prompt drug treatment to alleviate the complaints I was complaining of:	Yes 9/12	Only in part 1/12
		No
		No answer 2/12
After surgery, the wait to return to the ward seemed to me:	Normal 12/12	Long/too long
My overall assessment of the operating room experience is:	Positive 12/12	Negative
Would you repeat the operation with the same anaesthetic technique if necessary?	Yes 10/12	No 2/12
After surgery:	I was fine 6/12	I have suffered for:
		Pain 1/12
		Nausea/Vomiting 2/12
		Shivers/tremor 1/12
		Thirsty 2/12
		Sleepy legs
		Difficulty in waking up
		breathlessness 1/12
		Partial paralysis of the face 1/12
		Difficulty in speaking 1/12 (pre-existing deficit)
Do I judge the information I received from the anaesthetist before my operation to be complete?	Yes 10/12	Only in part 2/12 (language barrier)
		No
Of the anaesthesiological treatment received:	I am completely satisfied 10/12	I am unsatisfied
	I am partially satisfied 2/12	I am totally unsatisfied

### Abstract 71. Enhanced recovery pathways for video-assisted thoracoscopic surgery (VATS): an experience from Maggiore Hospital, Bologna

#### Scardua J.^1^, Santarelli S.^2^, Degli Esposti E.^1^, Solli P.^3^, Brandolini J.^3^, Kalamukai K.^3^, Riccheo A.^2^

##### ^1^Azienda Ospedaliero-Universitaria di Bologna Policlinico Sant'Orsola-Malpighi ~ Bologna ~ Italia, ^2^Ospedale Maggiore AUSL Bologna, Dipartimento di Emergenza ~ Bologna ~ Italia, ^3^Ospedale Maggiore AUSL Bologna, Dipartimento Sperimentale chirurgie generali e specialistiche oncologiche dell'IRCCS ~ Bologna ~ Italia

###### **Correspondence:** Scardua J.

**Background**

Enhanced Recovery After Surgery (ERAS) pathways in lung cancer surgery are associated with reduced complication, length of stay (LOS) and hospital costs. In this study we show the application of ERAS recommendation^1^ on VATS pulmonary lobectomy, with an adjunctive focus on the comparison between intravenous ketorolac and ibuprofen for postoperative analgesia.

**Methods**

49 patients were enrolled. They received detailed preoperative counselling (including smoking cessation and rehabilitation programs) and underwent surgery on day of admission or the day after, following fasting guidelines for solids (6h) and fluids (2 h). Preanesthetic medication was avoided. Intraoperative management included site-effector target-control infusion of short-acting agents (sufentanil 0,15-0,20 ng/ml and propofol 3,5-4,5 mcg/ml), and intercostal nerve block (levobupivacaine 0.5% 100 mg and dexamethasone 4 mg) performed by the surgeon, under direct vision, at the beginning of the procedure. They all received antibiotic prophylaxis according to local protocols prior to skin incision, active warming with forced air blankets, lung isolation with double-lumen tubes, protective ventilation strategies and restrictive fluid regime. Neither transurethral catheter nor nasogastric tube were positioned. At the end of surgery, a single chest tube was positioned and connected with a digital drainage system. Upon discontinuation of sufentanil, a morphine 0.05 mg/kg starter bolus was performed. They were all extubated at the end of surgery and transferred to Recovery Room (RR) before dismission to ward. 19 patients received intravenous postoperative analgesia with ketorolac 30 mg TID, in combination with paracetamol 1 g TID and morphine rescue dose of 5 mg for NRS > 3. The remaining 31 received ibuprofen IV 400 mg TID instead of Ketorolac. They were all prescribed PONV prophylaxis, oral fluid and diet on the same day of surgery and physiotherapy on day one.

Data were collected on: age; sex; BMI; ASA score; duration of surgery, NRS on leaving the RR and at 24-48-72 h; use of rescue medication; loss from chest drain on leaving the RR and at 24-48-72 h; time for chest tube removal, start of physiotherapy, LOS.

**Results**

Median VATS duration was 154 min on ibuprofen group and 130 on ketololac group; for each, median time for chest tube removal and LOS was of 3 and 5 days, respectively. All patients started physiotherapy on day one.

The statistical analysis revealed the superiority of ibuprofen on NRS at 24 h (p-value 0.041; Mann-Whiley test = 0.023) along with a reduction in drainage at 24 h (p-value= 0.01; Mann-Whiley test = 0.01).

No significant differences on demand for rescue medication or PONV medication was observed.

**Conclusions**

There is emerging evidence of efficacy of ERAS pathways in thoracic surgery. In our experience these recommendations lead to a satisfying pain management, early mobilization of patients and short LOS. In addition, ibuprofen proved to be comparable to ketorolac, and even more effective at 24 h post-surgery; it also showed a better safety profile and allowed to extend treatment, in line with AIFA indications, up to 72 h.

^1^ Batchelor TJP, Rasburn NJ, Abdelnour-Berchtold E, Brunelli A, Cerfolio RJ, Gonzalez M et al. Guidelines for enhanced recovery after lung surgery:

recommendations of the Enhanced Recovery After Surgery (ERASVR ) Society and the European Society of Thoracic Surgeons (ESTS). Eur J Cardiothorac Surg 2019;55:91–115

**Table 1 (abstract 71).**
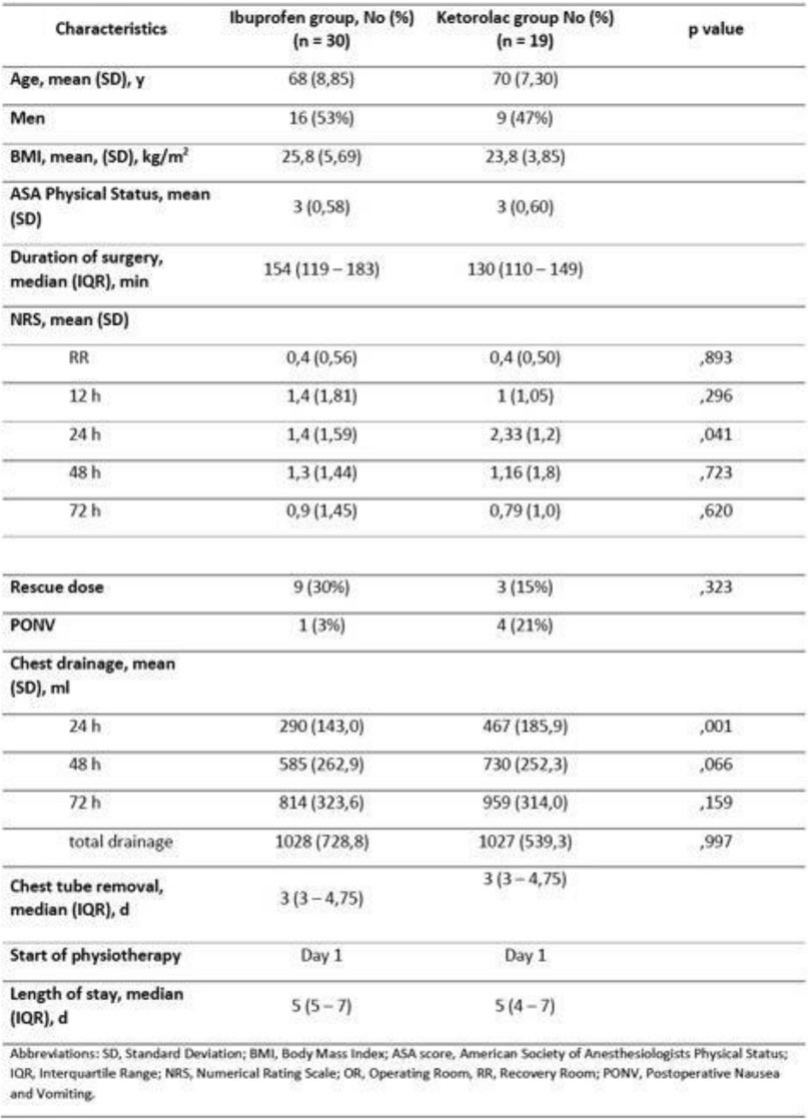
Baseline Demographics and Characteristics of Study Population

### A72. Mitral valve systolic anterior motion in robotic thoracic surgery as cause of “covered” hemodynamic shock: a case report

#### Barucco G.^1^, Palermo P.^2^, D”Amico F.^2^, Bohane S.M.^2^, Bonizzoni M.^2^, Casuale V.^2^, Spoto P.^2^, De Santis M.^2^, Gervasini M.^2^, Lezzi M.^2^, Monaco F.^1^

##### ^1^Ospedale San Raffaele ~ Milano ~ Italia, ^2^Universita' Vita E Salute San Raffaele ~ Milano ~ Italia

###### **Correspondence:** Barucco G.

**Background**: Systolic anterior motion (SAM) describes the dynamic movement of mitral valve (MV) anterior leaflet during systole towards the left ventricular outflow tract (LVOT)(1). The interaction between MV and LVOT leads to mitral regurgitation and LVOT obstruction (LVOTO) which, in turn, is associated with hemodynamic instability and shock. When it occurs intraoperatively if not promptly recognize may lead to a complicated perioperative course. Its treatment may be counterintuitive and require fluid administration and beta-blockade after the diagnosis performed by the transesophageal echocardiography (2). Here, we report for the first time, a case of a patient with SAM with a severe degree of LVOTO undergoing robotic lobectomy and its challenging intraoperative management.

**Case report:** We present a case of systolic anterior motion in 70-years-old man undergoing left pulmonary lobectomy. Informed consent to publish had been obtained. Past medical history included chronic kidney disease (CKD). On arrival, basal heart rate was 45 beats/min. Transthoracic echocardiography (TTE) revealed hypertrophic cardiomyopathy with dynamic obstruction of left ventricular outflow tract. TTE underestimates obstruction of LVOT with a gradient of 40mmHg. During surgery, patient was monitored with TEE. TEE revealed a gradient ranging between 60 and 160 mmhg (fig1). Treatment strategies applied were preload optimization without fluid overload, ultra-short acting beta blockers and vasopressors (fig 2). With regard to pain management paravertebral and interscalenic block were preferred over epidural analgesia in order to avoid vasodilatation. Patient reported a good quality of recovery and no pain the day after surgery assessed with QoR-15 score and NRS.

**Conclusion:** Management of patients with SAM in thoracic surgery requires a dedicated and skilled team together with high-impact treatment strategies that go beyond current guidelines. Finally, once again the present case report shows that TEE has a pivotal role in the management of complex cases even in the context of thoracic surgery.

Bibliography

1. Caselli S, Martino A, Genuini I, et al. Pathophysiology of dynamic left ventricular outflow tract obstruction in a critically ill patient. *Echocardiography*. 2010;27(10):E122-E124.

2. Manabe S, Kasegawa H, Arai H, Takanashi S. Management of systolic anterior motion of the mitral valve: a mechanism-based approach. Gen Thorac Cardiovasc Surg. 2018 Jul;66(7):379-389.

**Fig. 1 (abstract A72).**
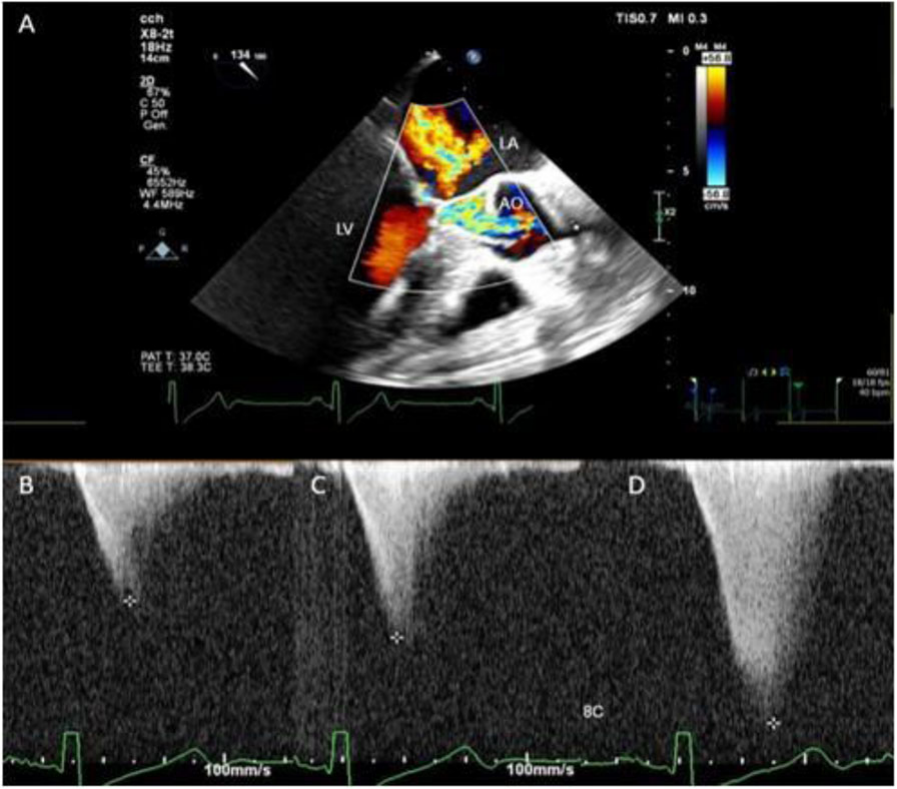
See text for description

**Fig. 2 (abstract A72).**
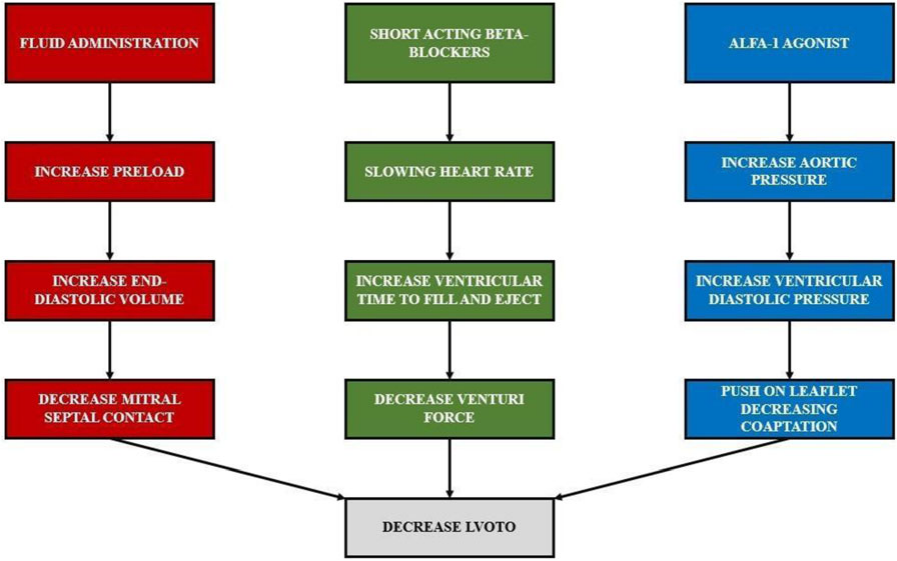
See text for description

### A73. General anesthesia versus monitored anesthesia care in patients undergoing endovascular repair of thoraco-abdominal aortic aneurysms

#### Barucco G.^1^, Russetti F.^2^, Faustini C.^2^, La Banca R.^2^, Fresili S.^2^, D”Amico F.^2^, Zaraca L.^2^, Dormio S.^2^, Monaco F.^1^

##### ^1^Ospedale San Raffaele ~ Milano ~ Italia, ^2^Universita' Vita E Salute San Raffaele ~ Milano ~ Italia

###### **Correspondence:** Barucco G.

In “endovascular era” of vascular surgery also the treatment of thoraco-abdominal aortic aneurysm (TAAA) is begun the therapeutic choice to treat patients who were inoperable with open approach (1) .Nevertheless little is known about anesthesiological management.

**Materials and methods**

The aim of this study is to value the relationship between type of anesthesia and shot term outcomes comparing patients undergoing visceral step (FEVAR/BEVAR) in general anesthesia (GA) and with monitored care anesthesia (MAC).

Methods: 144 patients undergoing elective fenestred/branched endovascular aortic repair (F/Bevar) from 2014 to 2021 at San Raffaele Hospital were retrospective analyzed.

All patients undergoing BEVAR/FEVAR received a standardised perioperative management (2). Routine monitoring (invasive arterial pressure, central venous pressure, pulse oximetry, five-lead ECG, seriate arterial blood gas analysis, urine output) was set in all patients.

The anaesthetic technique was chosen according to the anaesthesiologist’s and surgeon’s preference taking into account patient’s compliance. Demographic characteristics are reported in tab 1.

GA was induced with propofol 1.5-2 mg/kg, fentanyl 1 mcg/kg and rocuronium 0.6 mg/kg and maintained with desflurane/sevoflurane to achieve a Bispectral Index (BIS) between 40-60. Sedation was performed with remifentanil infusion of 0.05-0.1 mcg/kg/min and a bolus of 0.1 mg/kg of midazolam. A rescue dose of midazolam was administered to achieve BIS between 70-80 and a Richmond Agitation-Sedation Scale 0/-2.

Fluids, vasopressors (norepinephrine) and inotropes were administered to maintain a target of mean arterial pressure above 80 mmHg to avoid spinal cord ischemia

Primary endopoint included short term complications about patients undergoing GA and MAC

Secondary endpoint included hypotension, need of inotropes/vasopressors, time spent in operating room and admission in intensive care unit. All data were analyzed with SPSS.

Continuous variables are reported as mean ± standard deviation or as median with interquartile ranges (IQR). The differences between continuous variables were assessed with a t-test or with a Mann-Whitney. Categorical variables were compared with the χ-square test or the Fisher exact test when appropriated and expressed as numbers and proportions (%).

**Results**

As shown in tab 2 GA group showed more pulmonary complications.

The time spent in the operating theatre was longer and showed greater lactates in GA group while pH was more acidotic in MAC group with higher CO2 value.

The number of patients requiring in inotropes and the inotropic score were significantly higher in GA than sedation.

Conclusion: General anesthesia could be associated with more pulmonary complications and higher inotropic drug’s requirement. Further study are necessary to confirm these data.

Bibliography

1 Tenorio ER et al . Endovascular repair for thoracoabdominal aortic aneurysms: current status and future challenges. Ann Cardiothorac Surg. 2021 Nov;10(6):744-767

2 Bertoglio L, et al Elective Multistaged Endovascular Repair of Thoraco-abdominal Aneurysms with Fenestrated and Branched Endografts to Mitigate Spinal Cord Ischaemia. Eur J Vasc Endovasc Surg. 2020 Apr;59(4):565-576

**Table 1 (abstract A73).** Baseline clinical characteristics

Overall population	General Anesthesia	Sedation	p-value
*(n=124)*	*(n=62)*	*(n=62)*	
Age, years	73 [67-77]	73 [68-77]	0.418
Male sex, n (%)	47 (76)	44 (77)	0.832
BSA, (m2)	1.91[1.78-2.02]	1.85 [1.76-2.01]	0.479
SVS/AAVS, n (%)			0.924
0	4 (6)	6 (10)	
1	20 (32)	19 (31)	
2	27 (43)	27 (43)	
3	11 (18)	11 (16)	
ASA, n (%)			0.188
1	0	0	
2	6 (10)	2 (3)	
3	44 (71)	42 (68)	
4	12 (19)	18 (29)	
Creatinine Clearance, ml/min	59.4[42.0-79.3]	58.8 [46.1-77.8]	0.603
EF,%	60[55-60]	60[55-60]	0.309
Current smoking, n(%)	46 (74)	44 (71)	0.42
Hypertension, n (%)	58 (93)	60 (97)	0.34
Dyslipidemia, n (%)	44 (71)	41 (66)	0.56
Diabetes, n (%)	8 (13)	7 (11)	0.50
Obesity grade, n (%)			0.173
0	24 (39)	26 (42)	
1	32 (52)	23 (37)	
2	6 (10)	12 (19)	
3	0	0	
4	0	1 (2)	
GOLD, n (%)			0.994
0	21 (34)	21 (34)	
1	26 (42)	24 (39)	
2	12 (19)	14 (23)	
3	2 (3)	2 (3)	
4	1 (2)	1 (2)	
NYHA, n (%)			0.866
I	14 (23)	11 (18)	
II	26 (42)	25 (40)	
III	21 (34)	25 (40)	
IV	1 (2)	1 (2)	
Previous PTCA/CABG, n (%)			0.757
PTCA	17 (27)	19 (31)	
CABG	0	0	
Both	1 (2)	2 (3)	
Dialysis, n (%)	4 (6)	0	0.590
Antithrombotic therapy, n (%)			0.298
None	3 (5)	2 (3)	
Single platelet inhibitors	45 (73)	39 (63)	
Double platelet inhibitors	7 (11)	6 (10)	
Oral anticoagulant	4 (6)	12 (20)	
Antiplatelet/oral anticoagulant	3 (5)	3 (5)	

*BSA* body surface area; Society for Vascular Surgery/American Association for Vascular Surgery (SVS/AAVS), *ASA* American Society of Anesthesiologists, *EF* ejection fraction; Global Initiative for Chronic Obstructive Lung Disease; *NYHA* New York Heart Association, *PTCA* percutaneous transluminal coronary angioplasty, *CABG* coronary artery bypass graft surgery

**Table 2 (abstract A73).** Perioperative data

Overall population	General Anesthesia	Sedation	p-value
(n=124)	(n=62)	(n=62)	
Operating theatre time, min	363 [300-469]	336 [294-413]	0.04
Surgical time, minute	280[214-365]	266[210-318]	0.257
Number of patients transfused RBC, n (%)	30 (57)	27 (43)	0.589
pH	7.36 [7.33-7.40]	7.31 [7.27-7.38]	0.0001
paO_2_, mmHg	86 [64-111]	78 [63-101]	0.238
paCO_2_, mmHg	43 [39-48]	48 [41-57]	0.034
Sodium bicarbonate, mmol/l	22 [20-24]	22 [21-23]	0.681
Base excess, mmol/l	-2.3 [-4.6/-1.2]	-3.8 [-5.2/-1.3]	0.218
Lactate, mmol/l	2 [1.5-2.8]	1.3 [1.1-1.9]	0.0001
Blood losses, ml	400 [200-600]	250 [100-500]	0.027
Inotropes/vasopressors, n (%)	18 (29)	5 (8)	0.002
Vasopressors inotropic score	0 [0-5]	0 [0-0]	0.01
Admission in ICU, n (%)	14 (23)	7 (11)	0.09
ICU, days	0.66 [0-54-1.47]	2.99 [0.73- 8.71]	0.04
Creatinine peak, mg/dl	1.35 [1.03-1.93]	1.33 [1.14-1.62]	0.975
Creatinine clearance, ml/min	49 [32-68]	48 [38-60]	0.848
Surgical conversion, n (%)	1 (2)	0	1
Pulmonary complications, n (%)	12 (19)	11 (18)	0.04
Neurological complications n (%)	3(5)	3(5)	0.631
Cardiac complications, n (%)	8(13)	10(16)	0.360
Spinal cord ischemia, n (%)	7(11)	7(11)	0.694

*BSA* body surface area; Society for Vascular Surgery/American Association for Vascular Surgery (SVS/AAVS), *ASA* American Society of Anesthesiologists, *EF* ejection fraction; Global Initiative for Chronic Obstructive Lung Disease; *NYHA* New York Heart Association, *PTCA* percutaneous transluminal coronary angioplasty, *CABG* coronary artery bypass graft surgery

### A74. Anesthesiological management during bilateral modified sympathetic cardiac denervation [CSD] in uniportal video thoracoscopy in patients with refractory ventricular arrhythmias

#### Tozzi P.^1^, Bruno K.^1^, Piazzolla M.^1^, Brisciani M.^1^, Crocitti B.^1^, Pugliese F.^1^, Anile M.^2^, Vannucci J.^2^, Cauti F.M.^3^, Capone S.^3^

##### ^1^DAI Emergenza, Accettazione, Aree Critiche e Trauma - Policlinico Umberto I ~ Roma ~ Italia, ^2^DAI Cardio-Toraco-Vascolare e Chirurgia dei Trapianti d'Organo-Policlinico Umberto I ~ Roma ~ Italia, ^3^Cardiac Arrhytmia Unit-Ospedale San Giovanni Calibita ~ Roma ~ Italia

BACKGROUND

The use of sympathetic cardiac denervation (CSD) in ventricular arrhythmias (VA) refractory to medical and ablative therapy arises from studies that highlight the role of the neuroautonomic nervous system in the genesis of these cardiac rhythm abnormalities [Cauti et al.]. Numerous studies have shown a significant reduction in arrhythmic episodes following CSD both in patients with structural organic heart disease (dilated cardiomyopathy) and in patients with genetic disease (LQt syndrome). The modified sympathotomy technique applied at our institute is performed through uniportal video thoracoscopy and is performed biterally in the same operating session, identifying the sympathetic chain and proceeding with ablation from T2 to T4-T5.

The main objective of the anesthetist is to avoid factors that can trigger serious arrhythmias: maintenance of oxygenation [Swanevelder et al.], normocapnia, normothermia, normovolemia, correct pain management and preoperative anxiolysis are extremely important.

Careful titration of anesthetic drugs is required as well as optimization of mechanical ventilation, to maintain cardiovascular stability [Dornan et al.].

MATERIALS AND METHODS

Eleven male patients, mean age 65, 9 years, mean FE at pre-operative ETT 38%

Balanced anesthesia:

* midazolam 0.025 mg / kg in premedication;
* propofol 1.5 mg / kg, fentanyl 3-4 mcg / kg, rocuronium 0.6-0.8 mg / kg for induction;
* sevofluorane (end-tidal concentration 1.5-1.8%), fentanyl 1- 2.5 mcg / kg and rocuronium 0.15 mg / kg bolus on demand;
* sugammadex for complete decurarization on awakening based on TOF.

IOT with bilumen tube, OLV with VT 4-6 ml / kg, PEEP 0, variable RR, I: E 1: 1, fiO2 100%

Post-operative analgesia: paracetamol 1 g iv as needed.

Scheduled hospitalization in the ICU for postoperative monitoring.

Primary endpoints

- appearance of hypotension (PAM <65 mmHg) or severe hypotension (PAM <50 mmHg for at least 1 min [Salmasi et al.]) and AV episodes in the intraoperative

Secondary endpoints

- appearance of AV episodes during hospitalization in UTIPO and ordinary hospitalization;
- appearance of adverse effects due to CSD (skin dryness, skin flush, hyperhidrosis, altered thermoregulation, eyelid ptosis)

RESULTS

Of the 11 patients, 9 underwent bilateral CSD, the remaining 2 unilateral CSD (left).

In only one case (9% of the sample), there were intraoperative AV episodes (VT) and in the same patient 3 AV episodes during hospitalization in PACU.

All patients, in the intraoperative period, presented hypotension (PAM <65 mmHg), while 66% had a severe hypotensive event with MAP <50 mmHg for at least 1 minute.

Three patients (27.2%) experienced AV episodes during routine postoperative hospitalization; of these, two underwent a new attempt at ablation.

No patient had adverse effects related to CSD surgery.

CONCLUSIONS

- High risk of intraoperative hypotensive episodes (even with MAP <50 mmHg) due to poor starting cardiac performance and pre-operative therapy (b-blockers, calcium cantagonists): useful minimally invasive advanced hemodynamic monitoring with "pulse contour" methods
- "Patient tailored” anesthetic conduct effective in the prevention of intraoperative AV
- CSD is currently an operation with few side effects, also by virtue of a surgical procedure that aims at minimally invasive, proposing itself as an alternative to trans-catheter ablation
- need for more cases to achieve statistical significance in the future

### A75. Variables associated with the onset of delirium in heart transplant patients

#### Marianello D., Marcantonio S., Simeone F., Ialongo V., Maglioni E., Scolletta S., Franchi F.

##### Dipartimento cardio-toraco-vascolare ~ Siena ~ Italy

###### **Correspondence:** Marcantonio S.

Introduction

Post-operative delirium (POD-Post Operative Delirium) occurs in 50% of patients undergoing cardiac surgery. The physio pathological bases are multifactorial and they are linked to age and some comorbidities such as neurological diseases. Patients undergoing heart transplantation (HTX-Heart Transplant) are extremely frail and at high risk of developing POD, a complication that could lengthen the need for invasive mechanical ventilatory support and the average ICU stay.

The purpose of this study is to evaluate the incidence and the variables independently associated with the onset of POD in patients undergoing HTX.

Materials and methods:

A retrospective study was conducted on patients undergoing HTX and admitted at Cardio-Thoraco-Vascular Anesthesia and Intensive Care Unit of the University of Siena between 01-01-2016 and 31-12-2020. Patients under the age of 18 were excluded from the study. Anthropometric, clinical and laboratory data were collected and analyzed in the preoperative, intraoperative and postoperative period. A multivariate analysis was performed to identify the factors independently associated with the development of POD defined as positivity to the Confusion Assessment Method-Intensive Care Unit (CAM-ICU).

Results:

48 patients (34 males, 71%) with a mean age of 52 years were enrolled, 32 (76%) developed POD (SI-POD). SI-POD patients were older than those who did not develop delirium (NO-POD) (57 ± 8 years and 49 ± 8 years, SI-POD and NO-POD, respectively; p 0.015). Also, three hours after ICU admission, SI-POD patients had both PaO2 and venous saturation (SvO2) higher than NO-PODs (PaO2 137 ± 37 mmHg vs 97 ± 5mmHg p = 0.02, SI-POD vs NO-POD; SvO2 73 ± 12% vs 58 ± 7%, p = 0.01, SI-POD vs NO-POD). No significant differences emerged between the two groups in terms of mechanical ventilation hours and ICU hospitalization (mechanical ventilation hours 269 ± 277 vs 199 ± 257 hours, p = 0.48, SI-POD vs NO-POD; days of hospitalization 17 ± 12 vs 13 ± 11 p = 0.32, SI-POD vs NO-POD).

The only variable independently associated with the development of POD was PaO2 (OR 1.99 CI 1.01-1.1 p = 0.027). On ROC analysis, the optimum PaO2 cut-off predicting postoperative delirium was 120 mmHg (AUC 0.85, 95% CI 0.73-0.46, p <0.001), with a sensitivity of 80% and specificity of 75%.

Conclusion

Postoperative delirium occurs in a high percentage of heart transplant patients. A PaO2 higher than 120 mmHg is associated with the development of postoperative delirium in cardiotransplant patients.

### A76. Restrictive versus liberal red-cell transfusion strategy in cardiac surgery patients: a meta-analysis with trial sequential analysis

#### Merola F.^4^, La Via L.^3^, Moschitto L.^1^, Morgana A.^2^, Messina S.^2^, Valenti M.R.^3^, Murabito P.^3^, Pappalardo F.^5^, Astuto M.^3^, Sanfilippo F.^3^

##### ^1^Università di Catania, Facoltà di Medicina e Chirurgia ~ Catania ~ Italy, ^2^Università Magna Grecia, Scuola di Specializzazione in Anestesia, Rianimazione, terapia Intensiva e del dolore Magna Grecia ~ Catanzaro ~ Italy, ^3^Azienda Ospedaliero Universitaria Policlinico "G.Rodolico - San Marco" di Catania ~ Catania ~ Italy, ^4^Università degli studi di Catania, Scuola di specializzazione in Anestesia, Rianimazione, Terapia intensiva e del dolore di Catania ~ Catania ~ Italy, ^5^Azienda Ospedaliera di Alessandria ~ Alessandria ~ Italy

###### **Correspondence:** Merola F.

**Background**

Transfusion of red blood cell is very frequent after cardiac surgery. Current recommendations suggest that a restrictive transfusion strategy (transfusion indicated by hemoglobin [Hb] level below 7.0-7.5 g/dL) is as effective as a liberal strategy (variable cut-offs for transfusions ranging between 9.0 and 10.0 g/dL). We conducted a meta-analysis with Trial Sequential Analysis (TSA) to evaluate the effects on mortality of a restrictive transfusion strategy compared to a liberal transfusion strategy in cardiac surgery patients.

**Materials and Methods**

In this preliminary analysis, we screened PUBMED and EMBASE for randomized clinical trials (RCTs), up to January 3rd, 2022. We calculated relative risk (RR) for the all-cause mortality at longest follow-up, using a random-effect model with 95% confidence interval (CI). Meta-analysis was performed with separation in subgroups according to the population age (pediatric or adults). For the TSA, the information size was computed assuming an alpha risk of 5% with a power of 80%. The RR reduction was set at 20%, estimating a mortality in the liberal transfusion group of 45%, according to published evidence.

**Results**

Seven RCTs were included in our analysis (7.888 patients in total). All-cause mortality was not statistically different when the restrictive transfusion strategy was compared with the liberal transfusion strategy (RR 1.03, 95%CI 0.85-1.25, p=0.79, Figure 1). None of the subgroups presented differences in mortality between the two strategies. The TSA conducted on the outcome showed that the sample size required (14.081 patients) was not yet reached, but the Z-line crossed the futility line (Figure 2), demonstrating the robustness of these findings and further investigation does not seem necessary.

**Conclusions**

Current evidence supports the idea that a restrictive strategy for red-cell transfusion after cardiac surgery is safe and does not increase overall mortality as compared to a liberal strategy.

**Table 1 (abstract A76).**
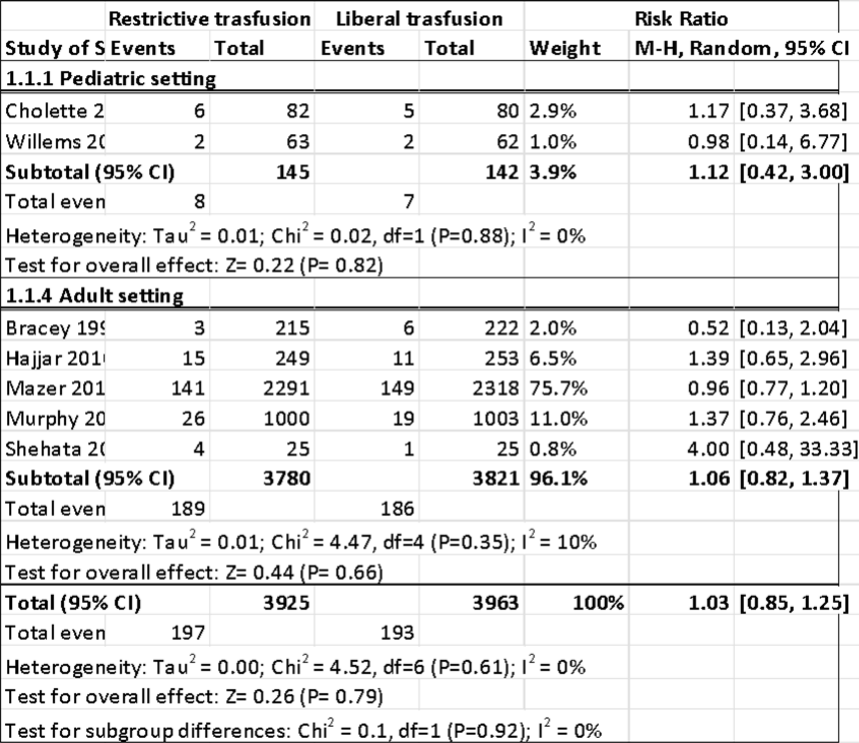
See text for description

**Fig. 1 (abstract A76).**
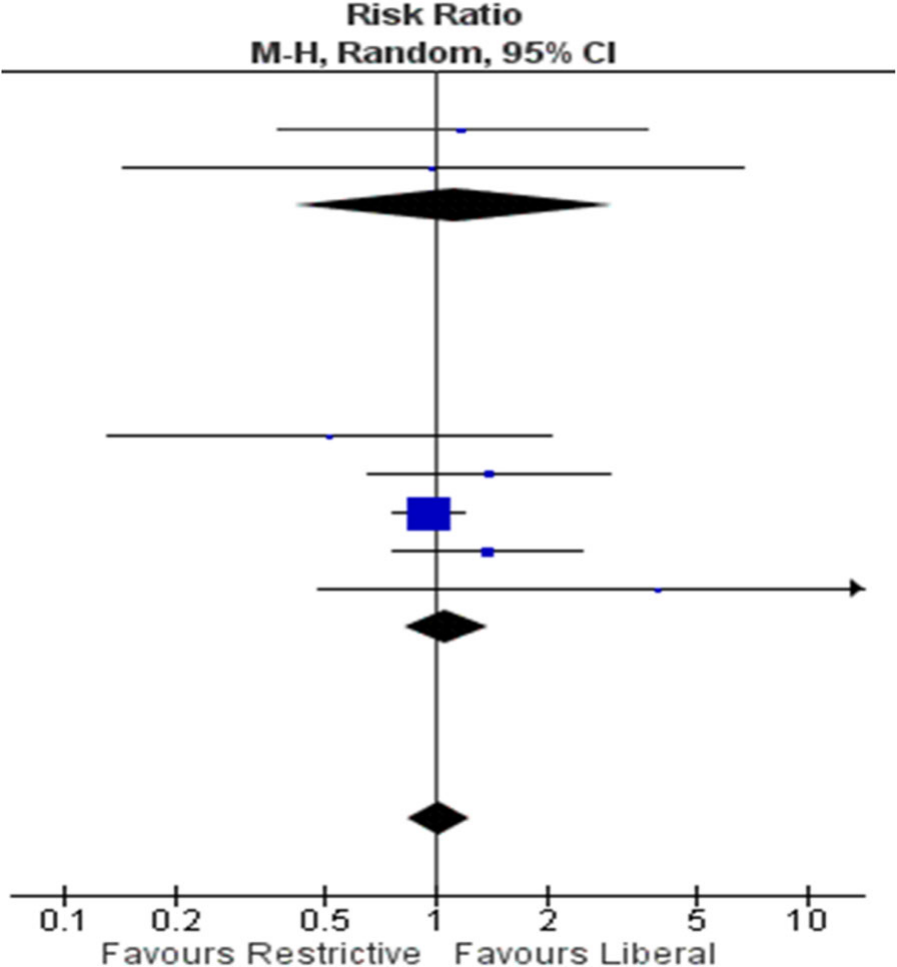
See text for description

**Fig. 2 (abstract A76).**
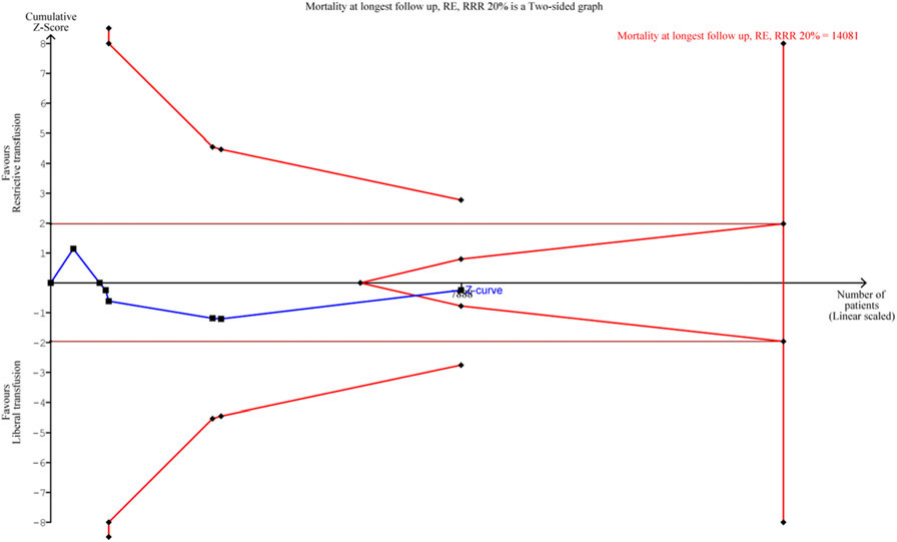
See text for description

### A77. Cardioprotection in cardiac surgery: role of dexmedetomidine

#### Marianello D.^1^, Marcantonio S.^1^, Angeloni C.^2^, Rossi I.^1^, De Matteis F.^[1]^, Giusti S.^1^, Ginetti F.^1^, Maglioni E.^1^, Scolletta S.^1^, Franchi F.^1^

##### ^1^Dipartimento cardiotoracovascolare ~ siena ~ Italy, ^2^Anestesia e rianimazione ~ carpi ~ Italy

###### **Correspondence:** Angeloni C.

Introduction:

Among the strategies aimed at minimizing reperfusion damage resulting from revascularization coronary artery disease, post ischemic pharmacological conditioning obtained with dexmedetomidine has attracted recent interest due to its ability to activate the RISK transduction pathway (reperfusion injury salvage kinase), ATP-dependent mitochondrial potassium channels and increase the production of nitric oxide.

The aim of this study is to evaluate whether dexmedetomidine may have a cardioprotective function after coronary artery bypass grafting (BPAC) with the use of circulation extracorporeal (CEC).

Materials and methods:

A retrospective study was conducted on 54 patients undergoing elective BPAC with use of the CEC between 01.01.21 and 30.09.21 and hospitalized in cardio-thoraco-vascular intensive care (TI) of the University Hospital of Siena. Patients were excluded under the age of 18. The population was divided into two groups: intraoperative use up to extubation of dexmedetomidine (SI-DEX) and routine conduct (NO-DEX). The choice of treatment was carried out by the anesthetist on duty. For capacity assessment cardioprotective treatment of dexmedetomidine, the values of troponin HS were measured upon admission in TI, after 3 and 24 hours. The ejection fraction (FE) was also calculated using the Simpson method biplane to echocardiography at discharge in all patients. A univariate analysis was performed considering a p<0,05 as statistical significance.

Results:

The average age of the enrolled patients was 67 ± 10 years; 45 were male (85%). Twenty-three (47%) patients were included in the SI-DEX group, 31 (57%) in the NO-DEX group. There were no significant differences between the two groups with respect to pre- and intraoperative data and timing of CEC and aortic clamping.

The lactate value at 3 hours after TI admission and the maximum postoperative bilirubin value were lower in the SI-DEX group (lactate, SI-DEX vs NO-DEX 1.6 ± 0.5 vs 2.6 ± 2.3 mmol / L, p = 0.04; bilirubin, SI-DEX vs NO-DEX, 0.69 ± 0.26 vs 1.06 ± 0.62 mg / dL, p = 0.015).

Troponin HS values did not differ between groups. The postoperative FE, measured at the time of discharge from the hospital, was higher in the SI-DEX group (SI-SEX vs NO-DEX 54 ± 5% vs 50 ± 7%, p = 0.026).

Conclusions:

Dexmedetomidine does not modify troponin HS values in patients undergoing elective BPAC. Patients treated with dexmedetomidine had higher EF at hospital discharge. The lower values of lactate and bilirubin could indicate an anti-inflammatory effect of the drug, to be investigated with further studies.

## Dolore acuto

### A78. ANI-V2 nociceptive monitoring in hepatic surgery

#### Martinetti N.M., Malossini S., Rocchi L., Stomeo N., Palma S., Piccioni F., Cecconi M., Greco M.

##### IRCCS Humanitas Research Hospital ~ Rozzano ~ Italia

###### **Correspondence:** Martinetti N.M.

ANI-V2 (Mdoloris Medical System, Loos, France) is a nociceptive monitoring device, which quantifies parasympathetic tone from the R-R interval variability analysis in electrocardiogram. We aimed to understand whether if ANI-V2 could influence intraoperative opioid consumption and improve postoperative analgesia in patients who underwent hepatic resections. Thus, a prospective analysis was conducted on 18 patients divided in two groups, with and without ANI-V2 intraoperative monitoring, respectively.

8 patients were monitored with our standard of care while 10 patients were monitored also with ANI-V2. Our standard monitoring for hepatic resections includes hemodynamic monitoring through an EV1000 system (Edwards Lifesciences, Irvine, Ca, USA), BIS (bispectral index), ECG, pulse oximetry. General anesthesia was induced with propofol, fentanil and rocuronium and maintained with sevoflurane and remifentanil. Analgesia was obtained with before-incision locoregional anesthesia and end of surgery therapy with paracetamol, non-steroidal anti-inflammatory drugs (NSAID) and morphine. Our main outcomes were intraoperative opioid consumption and postoperative analgesia, evaluated with the NRS scale in the post anesthesia care unit. Our secondary outcomes were hemodynamic stability, intraoperative MAC and intraoperative BIS.

The median ANI index was 60 [Q1-Q3: 49.5-66]. Less intraoperative opioids were used in the ANI-V2 group, both for median remifentanil 0.05 [0.03-0.1] vs 0.07 [0.05-0.08] mcg/kg/min and morphine 0 [0-5] vs 5 [3.75-8] mg. Postoperative NRS was better in the ANI-V2 group compared to the standard group 1 [0; 3] vs 3 [2-4] (all P values > 0.05).

As for the secondary outcomes, hemodynamic stability, measured as median mean arterial pressure 82 [70-95] vs 80 [70-94] mmHg and cardiac index 2.6 [2.2-3.1] vs. 2.8 [2.5-3.2] did not differ between the two groups, neither did the intraoperative MAC 0.8 [0.7-0.9] vs. 0.8 [0.7-1] nor the intraoperative BIS 42 [39-47] vs. 40 [40-45].

Our preliminary data suggest that ANI-V2-guided opiods administration might allow a reduction in the intraoperative opioid consumption. The limits of our study are the small sample size and the lack of experience in the interpretation of ANI-V2 numbers.

The introduction of new nociceptive monitoring systems for intraoperative analgesia might lead to a more opioid-sparing anesthesia approach, even in advanced surgery settings such as hepatic surgery.

## Dolore cronico

### A79. Stellate ganglion blocks for the treatment of CRPS type I

#### Laudani A., Gammaldi R.

##### AOU San Giovanni di Dio e Ruggi d'Aragona - UOC Anestesia e Rianimazione ~ Salerno ~ Italia

###### **Correspondence:** Laudani A.

Testo abstract

Complex Regional Pain Syndrome (CRPS) is a multifactorial chronic neurological condition affecting the limbs. The clinical picture consists of severe limb pan associated with varying degrees of sensory, autonomic, motor and trophic impairment. CRPS can be induced by surgical or traumatic nerve injury, but actually in most cases little or no obvious nerve injury can be recognized (CRPS type I). CRPS has a variable course and can lead to significant disability. A 39-year-old immigrant women, caregiver, with a history of domestic violence was referred to Pain Management Service because of progressive and worsening pain in her left upper limb. Pain was reported to be 7/10, on average, and described as deep, burning and shooting; it was associated with hyperesthesia. No specific inciting events were reported in medical history. Physical examination revealed: edema of the whole limb, decreased range of motion especially of the large joints, hair trophic changes, skin redness. Both neurological examination and diagnostics imaging were inconclusive. Based on the Budapest criteria the diagnosis of CRPS was made.

The patient only took NSAIDs occasionally. We started multimodal drug therapy with tapentadol on rising dose up to 300 mg per day, pregabalin 75 mg BID, venlafaxine 37.5 mg BID. On follow-up after 4 weeks, an improvement in pain control (NRS 5/10) and motility was reported. However the patient was not satisfied and agreed to infusion therapy with 100 mg neridronate every three days, four administrations in total. This therapy did not lead to substantial benefits, so a series of stellate ganglion block was proposed. The patient received four US-guided left stellate ganglion blocks with 4 ml of 0.2% ropivacaine. One month after the end of this treatment, the patient reported a better pain control (NRS 3/10) and an improvement in motility, enabling her to follow a physical therapy program.

Informed consent to publish had been obtained.

### A80. Cannabis derivates effects on long standing therapies for non oncologic pain: four years experience in a single center

#### Riso C., Scarano A., Sbaraglia F., Costanzi M., Festa R., Micci D.M., Caputo C. T., Rossi M.

##### Department of Anesthesia and Intensive Care, Fondazione Policlinico Universitario Agostino Gemelli IRCCS ~ Rome ~ Italia

###### **Correspondence:** Scarano A.

Background

Constipation (a bowel movement later than every 3 days) is an adverse effect of many therapy in patients suffering from non-oncologic chronic moderate to severe pain, affecting the quality of life and adherence to treatment, even using newer analgesics such as tapentadol. Several authors suggest introducing cannabis derivates to manage such a setback in the treatment plan, due to their action on multi-dimensional aspects of pain. Sharing this vision, we proposed Cannabis oil THC 19% CBD <1% (Bedrocan) for the outpatients suffering from severe constipation. After four years, we reviewed our database to verify the efficacy of cannabis derivates in a subgroup of subjects undergoing therapy with Tapentadol.

Materials and Methods

We analyzed from January 2018 to January 2022 our internal electronic database of outpatients proposed for cannabis treatment and on chronic therapy with tapentadol as major analgesic. We excluded those suffering from oncologic pain. We review the electronic chart to analyze at one month and three months on cannabis therapy 3 main outcomes: tapentadol dosage reduction, constipation incidence, need of bowel prokinetics

Results

Forty patients treated with tapentadol (mean dose 200 mg/die) and complaining of constipation in almost two outpatient visits, were identified. Indications for therapy were 14 interstitial cystitis, 12 fibromyalgia, 6 atypical facial pain, 4 brachial plexus injuries, and 4 Trigeminal neuralgia. All patients received Bedrocan 6 oral drops per day. After 1 month 25 patients completely dismissed Tapentadol, 14 reduced tapentadol intake (mean 125 mg/die), and one didn’t get relief. No one complained of severe constipation, but 13 patients carried on prokinetics therapy.

After 3 months 38 patients completely dismissed tapentadol, and two patients reduced the intake to 100 mg/die. Constipation was absent in all patients and no one required prokinetics.

Conclusions

The introduction of cannabis derivates in our experience appeared to positively affect the management of chronic non-oncologic pain. Analysis of our database suggests that patients treated with Bedrocan reduced the intake of analgesics associated to an improved bowel motility. The wide spectrum of activities of cannabis derivates on pain, sleep and daily behaviors could in part explain our results. Further studies and higher number of patients are needed to clarify these positive outcomes and to verify their maintenance over time.

References

Camilleri M. Cannabinoids and gastrointestinal motility: Pharmacology, clinical effects, and potential therapeutics in humans. Neurogastroenterol Motil. 2018 Sep;30(9):e13370

Noori A, Miroshnychenko A, Shergill Y, et al. Opioid-sparing effects of medical cannabis or cannabinoids for chronic pain: a systematic review and meta-analysis of randomised and observational studies. BMJ Open. 2021 Jul 28;11(7):e047717

### A81. Efficacy and safety of autologous conditioned serum for the treatment of chronic pain in patients with osteoarthritis: a pilot observational study

#### Ippolito M.^1^, Spurio G.^1^, Compagno V.^1^, Rizzo A.^5^, Di Simone M.^2^, Corsale A.M.^2^, Mazzola G.^3^, Meraviglia S.^2^, Cortegiani A.^1^, Alongi A.^4^, Giarratano A.^1^

##### ^1^Department of Surgical, Oncological and Oral Science (Di.Chir.On.S.), University of Palermo ~ Palermo ~ Italia, ^2^Central Laboratory of Advanced Diagnosis and Biomedical Research (CLADIBIOR), Department of Biopathology and Medical Biotechnologies (DIBIMED), University of Palermo ~ Palermo ~ Italia, ^3^Unit of Transfusion Medicine, University Hospital "Paolo Giaccone" ~ Palermo ~ Italia, ^4^Department of Anaesthesia, Intensive Care and Emergency, Policlinico Paolo Giaccon ~ Palermo ~ Italia, ^5^Department of Anaesthesia, Intensive Care and Emergency, Ospedale Paolo Borsellino ~ Marsala ~ Italia

###### **Correspondence:** Spurio G.

Background: Conditioned autologous serum is a product of blood origin, with fragmented evidence of therapeutic properties in osteoarthritis chronic pain. This pilot observational prospective study aimed to evaluate the efficacy and safety of conditional autologous serum (ACS) in patients with severe chronic pain and grade I-III osteoarthritis and to describe its cytokine content. Materials and methods: We prospectively collected data on consecutive patients affected by osteoarthritis grade I to III and treated with four weekly injections of ACS from 5th November 2020 to 15th June 2021 at the outpatient pain service of Policlinico Paolo Giaccone, Palermo. The primary outcome was pain intensity, measured with the visual analogic scale (VAS) expressed in centimeters (0, “no pain” – 10, “pain as bad as it could possibly be”). Additional outcomes were the severity of symptoms and functional limitation, evaluated using joint district-specific scales (ODI, WOMAC, quickDASH), health related quality of life, assessed using SF-36 and he functional impairment in daily life, evaluated using the Karnofsky performance status. All the outcome measures were evaluated weekly, for the first four weeks, then at one, three and six months, except for SF-36, assessed at baseline and then at one, three and six months from the beginning of the treatment. The study also evaluated concentrations of forty-eight cytokines and chemokines involved in the balance pro-inflammation/anti-inflammation and tissue repair in the ACS. Results: We included 26 patients, mostly female (65.4%), with a median age of 63.5 years [IQR 58.25-73]. A median reduction of VAS of -3 cm [-5;-1.25] was observed six months after the first injection of ACS. The analysis showed a statistically significant difference between the values of VAS (P < 0.01; X2=69.6; df= 6, N = 26), Karnofsky performance status (P < 0.01; X2=25.7; df= 6, N = 26), SF-36 Mental Component Summary (P < 0.01; X2=18.1; df= 3, N = 26) and SF-36 Physical Component Summary (P < 0.01; X2= 13.3; df=3, N = 26) at different timepoints. In addition, a statistically significant difference was found between the values of the WOMAC Index (P < 0.01; X2=22; df= 6, N = 9), ODI (P < 0.01; X2=33; df= 6, N = 12), and quick-DASH (P < 0.01; X2=18.2; df= 6, N = 5) at different timepoints. No adverse events were observed or reported by patients during the entire study period. Notably, IL-1RA median concentration was 471.6 [404.7-747.1] pg/ml and factors involved in tissue repair were detected. Conclusions:The use of autologous conditioned serum led to a significant reduction of pain intensity and improvement of joints function in a cohort of patients with grade I-III osteoarthritis refractory to other treatments. No adverse events were registered. These preliminary findings should be confirmed in studies with adequate design.

**Fig. 1 (abstract A81).**
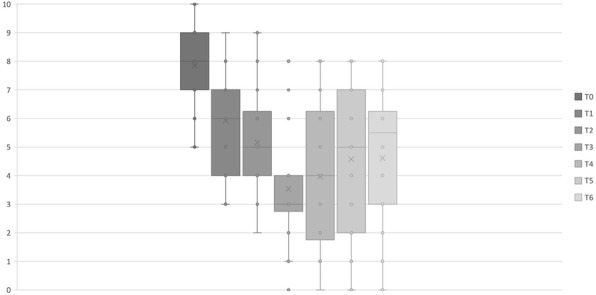
See text for description

### A82. Effect of psychological counseling on quality of life in patients with chronic pain in pharmacological therapy. A pilot study

#### Consoletti L., Padalino S., Rauseo M., Petrone A., Cinnella G.

##### Azienda Ospedaliera Universitaria Policlinico Riuniti ~ Foggia ~ Italia

###### **Correspondence:** Padalino S.

**Introduction**

Carotid endarterectomy (EAC) can be performed

1. under general anesthesia (AG) with instrumental intraoperative brain monitoring and placement of selective endoluminal shunt as needed or with routine endoluminal shunt
2. in local or locoregional anesthesia (ALR) in which intraoperative brain monitoring with selective endoluminal shunt placement as needed (the patient is awake and responds to simple commands).
3. under general anesthesia to cooperating patient (AGPC) which combines the advantages of both types of anesthesia.

For the third anesthetic choice, there is a strong research recommendation regarding patient satisfaction with the AGPC method, as can be seen from the LG "Diagnosis and treatment of extracranial carotid obstructive steno pathology and prevention of cerebral stroke" published in the National Lines System Rome guide, 1 October 2021. The purpose of this study is therefore to describe the tolerability and satisfaction of our patients who underwent carotid endarterectomy under general anesthesia in collaborating patient (AGPC) from January 1, 2022 to May 30, 2022.

**Materials and methods**

Since January 2022 we have applied to all patients treated for carotid TEA the method (AGPC) which involves the use of a general anesthesia, maintained with Remifentanyl, in which the patient is superficialized by hypnosis during the clamping phase of the carotid so to make possible the clinical evaluation of his motor response at the operator's request, combined with the execution of the superficial cervical plexus block to ensure effective post-operative analgesia. (Fig. 1)

Anesthetic scheme used:

- Propofol -> 1,0 – 1,5 mg/kg
- Succinilcolina -> 1,0 – 1,5 mg/kg
- Remifentanil -> 0,25 mcg/kg/min (titration as needed)
- Sevoflurano -> 0.20% - 0,25%
- Lidocaina -> 4% spray -> 5 ml
- Blocco -> Ropivacaina -> 0,35 mg
- Anesthesia at the incision site -> Lidocaina -> 2%

Within 4 days of the operation, we submitted to all operated patients the anesthesia assessment survey ("QUALITY CONTROL IN ANESTHESIA FOR CAROTID SURGERY (CAROTID TEA").(Tab.1)

In addition to the standard monitoring of the bloody PA, SPO2, BIS, ECG, for some patients it was possible to use an intraoperative brain monitoring system (bilateral regional cerebrovascular oxygen saturation - INVOS) designed to quantify the hypoxic insult following the clamping phase.

**Results**

We enrolled a total of 14 patients, 2 of whom didn’t receive the survey as they underwent Shunt for clinical reasons.

**Conclusions**

The advantages that can be appreciated from the use of this method are the reduction of the psychological and surgical stress of the intervention for the patient, the possibility of maintaining a favorable hemodynamics and absolute control of the airways by the anesthetist.

References

a) LG “ Diagnosi e trattamento della patologia steno ostruttiva carotidea extracranica e prevenzione dell'ictus cerebrale” pubblicata nel Sistema Nazionale Linee Guida Roma, 1 ottobre 2021

b) Superficial versus combined (deep and superficial) cervical plexus block for carotid endarterectomy.Ivanec Z, Mazul-Sunkol B, Lovricević I, Sonicki Z, Gvozdenović A, Klican K, Krolo H, Halapir T, Novotny Z.Acta Clin Croat. 2008 Jun;47(2):81-6.

**Table 1 (abstract A82).** See text for description

Mann-Whitney test analysis	Before psychological counselling	After psychological counselling	p-value
SF36-General Health	50 [25-90]	32 [18-49]	0.04
HADS scale	17 [9-19]	8 [4-15]	0.03
Fatigue severity scale	47 [31-59]	28 [12-41]	0.03

### A83. Individualized electroacupuncture for fibromyalgia treatment: a retrospective “before-after” study on pain and quality of life

#### Pitoni S., Del Prete D., Riso C., Scarano A., Vergari A., Catalano A., Ferrone G., Rossi M.

##### Dipartimento di Scienze dell'emergenza, anestesiologiche e della rianimazione ~ Roma ~ Italia

###### **Correspondence:** Pitoni S.

Background

Fibromyalgia (FBM) is a chronic pain syndrome affecting between 2 and 5% of the general population [1]. Its therapy is multimodal, multidisciplinary and comprises non-pharmacological treatments. Acupuncture and electroacupuncture (EA) have a pivotal role in FBM treatment [2]. The aim of this study is to evaluate the impact of EA in patients with FBM.

Materials and methods

This is a “before-after” retrospective study conducted on 18 adult patients (17F/1M, mean age 53± 16yrs) diagnosed with FBM within the previous 3 months and on medical therapies, who have been treated at the Acupuncture Clinic of Gemelli Hospital between 12/2020 and 04/2022. The treatment protocol consisted of a cycle of 6 EA sessions on a weekly basis.

Before and immediately after the EA therapy cycle, at respectively T0 and T1, the following questionnaires were administered: NRS, Brief Pain Inventory (BPI), which is made up of Pain Severity Score (PSS) and Pain Interference Score (PIS), Revised Fibromyalgia Impact Questionnaire (FIQR), Fatigue Severity Scale (FSS), Nicholson McBride Resilience Questionnaire (NMRQ), Patient's Global Impression of Change Scale (PGIC) and SF-36.

Results

Only two patients were not taking drug therapies; five patients took antidepressants and six got opioids. EA produced a reduction of both NRS and BPI. NRS presented a 24.6% reduction from 6.5 ± 1.4 to 4.9 ± 2.0 (p <0.05), while the mean PSS and PIS were both decreased from 6.3 ± 1.5 to 4.9 ± 1.9 and from 7.4 ± 1.4 to 5.9 ± 1.9, (p <0.05). Moreover, the FIQR decreased from 69.6 ± 10.7 to 56.3 ± 18.3 (p <0.05), highlighting an improvement in patients' health status in terms of physical function, overall impact of the disease and severity of associated symptoms. There was no change in terms of fatigue (FSS, 55.1 ± 6.2 vs 50.9 ± 6.8, p> 0.05) or resilience (NMRQ, 32.8 ± 7.1 vs 35.7 ± 5.9, p> 0.05). The self-assessment of the change in the painful condition after EA showed a PGIC of 4.1 ± 1.4 points. The impact of therapy on patients' quality of life is shown in table 1 (SF-36 score). It shows a significant improvement in vitality, mental health and physical pain.

Conclusions

This study highlights the short-term positive impact of EA on pain perception and quality of life of FBM patients. Future studies are needed to evaluate the medium and long-term efficacy of EA to better define the role of this treatment in the therapeutic process of FBM patients, also considering its possible effect on the reduction of drug therapies.

**Table 1 (abstract A83).** See text for description

SF-36 Domains	T0	T1	Sign.
PF	45.7± 22.6	49.2±15.8	ns
RF	3.6± 9.1	9.6± 12.7	ns
RE	26.2± 29.8	23.1± 31.6	ns
VT	17.9± 12.4	30.4± 11.4	p<0.05
MH	35.7± 13.2	48.9± 12.9	p<0.05
SF	33.0± 23.3	43.3± 16.6	ns
BP	19.1± 13.9	36.2± 14.4	p<0.05
GH	30.7± 15.8	35.4± 19.8	ns

### A84. Use of oxygen ozone therapy in the treatment of posterpetic neuralgia refractory to other therapies: a case report

#### Caruso M., Bonadio F., Latella A., Lucia M.T., Monardo A.

##### Anesthesia Resuscitation and Pain Therapy Unit Giovanni Paolo II Hospital ~ Lamezia Terme ~ Italia

###### **Correspondence:** Caruso M.

Background: Posterpetic neuralgia (PEN) is the most common and fearful chronic complication of Herpes Zoster infection. In young subjects it occurs in about 20% of cases, it rises up to 70% in elderly people. Neuropathic pain is usually localized in the skin region where the eruption of the vesicles has occurred, it is felt when they are healing or even a few days before their appearance and it can last several years if it isn’t adequately treated. The most affected dermatomal sites are the thoracic and trigeminal ones. The pain is more frequently continuous, very intense, disabling, stabbing, burning, exacerbated by contact and associated with hyperalgesia and allodynia deriving from unilateral inflammation of the cranial or spinal nerves and their ganglia caused by the neurotropic virus. Tingling and itching may also occur. NPE is associated with depression, insomnia, weight loss and severely impaired quality of life.

Objective: The purpose of this case report is to verify the analgesic effect of Oxygen Ozone (O2O3) Therapy and the improvement of the quality of life in a 47-year-old patient with PEN.

Patient and Methods: The young patient under examination went to our observation for unbearable, stabbing, burning, disabling pain (NRS 10), associated with hyperalgesia and allodynia in the right intercostal region following a Herpes Zoster infection not promptly diagnosed because of the lack of vesicular rash. She was already being treated with Pregabalin 150 mg x 2, Amytriptyline 50 mg/day, Tapentadol 150 mg x 2, Lidocaine-based cream without benefit but with drowsiness, asthenia and continuous nausea.

Eight biweekly and four a week sessions of Oxygen Ozone (O2O3) Infiltrations were performed along the lower costal arch following the course of the spinal nerve starting from the tenth thoracic ganglion. A Stable Ozonoids (23%) and Vitamin E (OzoilE)-based Cream and a Rechargeable Tape with the same Cream were then applied. This Tape gradually releases OzoilE.

Results: The patient found a benefit already after the first sessions with a clear improvement in painful symptoms (NRS 4-5) and quality of life without side effects. The painful area was reduced in extension until it was identified in a single point. Oral therapy was gradually tapered off until it was suspended.

Conclusions: The Infiltrations of Oxygen Ozone along the course of the spinal nerve responsible of posterpetic neuralgia followed by the application of a Stable Ozonoids (23%) and Vitamin E-based Cream and a OzoilE Rechargeable Tape are very effective in pain reduction and improvement of quality of life. This treatment had no side effects.

This case report demonstrates the important actions of O2O3: antiviral (it deactivates the receptors present on the surface); immunomodulating (it increases the ability of macrophages to eliminate invading microrganisms and damaged cells); revitalizing and regenerating (it improves the local blood microcirculation with better oxygen supply and a more rapid elimination of toxic substances, essential for the regeneration of damaged anatomical structures); antinflammatory (it reduces proinflammatory cytokines); analgesic (it releases endorphins and oxidizes algogenic metabolites which induce pain by acting on the nerve endings).

Informed consent to publish had been obtained.

### A85. Neuromodulation by radiofrequency for ganglion impar block in the management of a clinical case of perineal cancer pain not responsive to medical therapy

#### Guarino C.^1^, Caramia R.^1^, Di Bari M.G.^1^, Mele R.^1^, Galizia C.^1^, Bellanova G.^2^, Miccianza A.M.^3^, Giuseppina C.^1^, Fedele O.^1^

##### ^1^Anesthesia, Resuscitation and Pain Therapy Unit, D. Camberlingo Hospital, ASL BR ~ Francavilla Fontana ~ Italia, ^2^General Surgery Unit, D. Camberlingo Hospital, ASL BR ~ Francavilla Fontana ~ Italia, ^3^Internal Medicine Unit – Oncology Day Hospital, D. Camberlingo Hospital, ~ Francavilla Fontana ~ Italia

###### **Correspondence:** Caramia R.

**Background**

Oncological perineal pain can be very disabling for patients who suffer from it, greatly limiting the quality of life. The approach is initially pharmacological and involves the use of anti-inflammatory analgesic drugs, opioid drugs, and a combination of these. In some cases, however, medical therapy is not effective and other therapeutic approaches need to be practiced. The aim of the present paper is to describe the clinical case of a patient with perineal cancer pain who did not find benefit from conventional medical therapy and was treated with ganglion impar radiofrequency neuromodulation.

**Case report**

A 59-year-old woman came to our observation with particularly disabling perineal pain. The patient, in fact, suffered from continuous pain that was greatly exacerbated by the poorly tolerated sitting position.

In anamnesis the woman had a previous recto-sigma resection surgery for rectal carcinoma. At a first evaluation, drug therapy with opioids and anti-inflammatories was set.

At subsequent check-ups, although medical therapy was modified and personalized, there were no significant improvements in the clinical condition, so it was proposed to perform a ganglion impar block. A test block procedure was initially practiced testing the patient's response to definitive therapy. This first approach gave encouraging results with control of the painful symptoms for about a month, for which we proceeded, with a certain awareness of the success, to the definitive procedure by neuromodulation with pulsed radiofrequency at 42 ° C for 6 minutes at 60 V of energy delivered. The procedure made use of radioscopy plus contrast agent (Figure 1) means and sensory and motor stimulation. The woman, after the antalgic therapy session, was re-evaluated after one and three months. She showed a marked improvement in her symptoms and in her mood, for having finally got rid of a pain she could no longer bear. He did not experience any adverse effects and continued antiblastic therapy.

Informed consent to publish had been obtained.

**Conclusion**

In this clinical case, the painful perineal symptoms in a cancer patient were resolved with the ganglion impar block by radiofrequency, after the failure of medical therapy. The test dose practiced was very useful in understanding and predicting whether the woman would respond to the recommended therapy. Ganglion impar neuromodulation by radiofrequency is a reliable and safe technique in expert hands that can give positive analgesic responses in patients with perineal pain syndrome.

**Fig. 1 (abstract A85).**
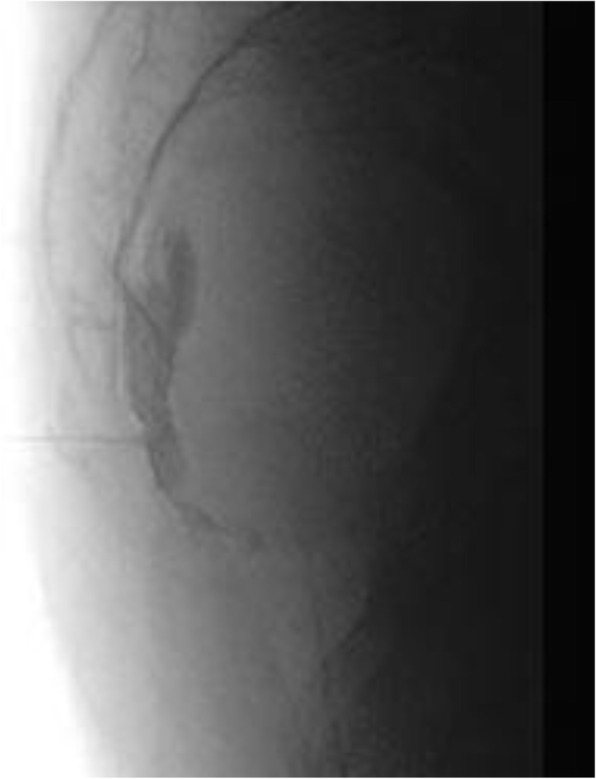
See text for description

### A86. Retrospective evaluation of ganglion impar pulsed radiofrequency as treatment of chronic pelvic pain syndrome

#### Costanzi M., Zappia L., Del Prete D., Concina G., Spinazzola G., Catalano A., Continolo N., Rossi M.

##### Department of Anesthesia and Intensive Care, Fondazione Policlinico Universitario Agostino Gemelli IRCCS ~ Rome ~ Italia

###### **Correspondence:** Costanzi M.

BACKGROUND

Chronic pelvic pain syndrome (CPPS) is a debilitating condition that significantly affects the quality of life and often needs different treatments, from conservative management to neurostimulation. Pulsed radiofrequency (PR), creating an electromagnetic field at the tip of the electrode and interfering with the impulse conduction of neurons, has been proven to be effective and safe in treating neuropathic pain. Although the role of the sympathetic nervous system in neuropathic pain is controversial, impar ganglion has been described as a possible nervous target and injection has shown promising outcomes. Radiofrequency application on the ganglion impar has been described for coccydynia, but the literature about its application specifically in pelvic pain is still poor. This retrospective study aimed to describe a case series CPPS patients with the primary localization of symptoms at the perineal area, treated with a PR and block of the ganglion impar, analysing pain control at 1-2 and 8-12 months as the outcome.

MATERIALS AND METHODS

Data of all patients affected by CPPS treated with PR of ganglion impar from April 2017 to April 2021 at Fondazione Policlinico Gemelli of Rome were retrospectively analysed. Ganglion impar pulsed radiofrequency, according to our internal protocol, was performed with a standard radiofrequency needle and generator (current at 42°C for six minutes, 45-50 V), followed by a local anaesthetic and corticosteroid mixture (bupivacaine and triamcinolone) of 1-2 ml. Pain outcomes were evaluated according to the numeric rating scale (NRS) reported in the medical records, at the beginning of treatment, 60-90 days (first clinic follow-up) and 8-12 months (second clinic follow-up) from the end of the procedure.

RESULTS

Six patients were considered suitable for the final analysis, five female and one male, with a mean age of 53 years and median pain NRS before the procedure of 6.2.

Primary localization of symptoms was consistent in the perineal region (6/6 subjects) but the overall pain distribution extended inconsistently to different areas (low back, leg, groin). The mean duration of pain was 7.7 years, ranging from 18 months to 20 years. One patient underwent a double-needle approach and the rest (5/6) single-needle approach. All patients experienced an NRS value decrease after the procedures at the first FU (median of 2). The second follow-up showed sustained pain relief only in 50% of subjects.

No adverse effects were observed in 5 subjects, one described a local pain at the needle insertion site that was managed with NSAIDS and lasted for three days.

CONCLUSIONS

This retrospective analysis supports the safety and efficacy of PR in terms of short term pain reduction, but a sustained efficacy was detected in only a part of the cohort. Unfortunately, this is a case series, and randomized trials must confirm its results. In conclusion, besides medication management and psychological interventions, ganglion impar pulsed radiofrequency could be an alternative to alleviating chronic refractory pain before proceeding to other more invasive approaches.

References

Pain Physician. 2018 Mar;21(2):147-167

J Pain Res . 2016 Dec 7;9:1173-1177

Medicine (Baltimore). 2021 Jul 30;100(30):e26799.

### A87. Use of ozoile-based cream and rechargeable tape post O2O3 Infiltrations vs O2O3 infiltrations in the treatment of herniated disc back pain: a randomized controlled clinical trial

#### Bonadio F., Caruso M., Latella A., Lucia M.T., Monardo A.

##### Anesthesia Resuscitation and Pain Therapy Unit Giovanni Paolo II Hospital ~ Lamezia Terme ~ Italia

###### **Correspondence:** Bonadio F.

Background: Disc herniation is a vertebral pathology currently very common even among young people. Oxygen Ozone (O2O3) Therapy is the safest and most effective among outpatient treatments; it has a pain-relieving, but mainly curative action: it exerts a decompressive action on the dural sac and nerve roots by dehydrating the pulpy nucleus escaped from the fibrous annulus.

The Cream, under study, is based on highly concentrated Stable Ozonoids (23%) and Vitamin E (OzoilE); the Rechargeable Tape is a device that acts as a transdermal patch: it consists of rechargeable microcells with the same Cream and the active ingredients are released gradually; therefore it combines the classic antalgic-biomechanical action of the Tape with the healing anti-inflammatory and regenerating effect typical of O2O3.

Objective: This study was conducted to verify the additional benefit of applying OzoilE-based Cream and Rechargeable Tape with the same Cream for at least 48 h to ultrasound (US)-guided paravertebral O2O3 Infiltrations.

Patients and Methods: We conducted a randomized controlled trial from September 2021 to December 2021 enrolling two hundred adult patients with herniated disc back pain. Patients were randomized in two groups: the first group included US-guided paravertebral O2O3 Infiltrations, application of OzoilE-based Cream and Rechargeable Tape with the same Cream and the second group included O2O3 Infiltrations alone. The concentration and quantity of O2O3 injected (15 mcg/ml, 10 ml for side) and the frequency of weekly sessions were the same. The pain assessment by NRS scale and the consumption of NSAIDs or opioids in the days following access to the pain therapy clinic were reported in the medical records at each session.

Results: The pain assessed according to the NRS scale and the use of pain relievers were lower in the patients of the first group (US-guided paravertebral O2O3 Infiltrations, application of OzoilE-based Cream and Rechargeable Tape with the same Cream) at each session. The NRS Score was reduced to 3-4 in the first group, to 5-6 in the second one.

Conclusions: The application of OzoilE-based Cream and Rechargeable Tape with the same Cream for at least 48 h offers an additional benefit to the US-guided paravertebral Infiltrations of Oxygen Ozone in terms of pain relief and reduction in the use of painkillers.

## Dolore oncologico e cure palliative

### A88. Early treatment with invasive technique in cancer pain management, impact on patient’s quality of life. A project of study

#### Claroni C.^1^, Vacirca R.^2^, Carraro A.^2^, Bonarrigo C.^1^, Torregiani G.^1^, Covotta M.^1^, Forastiere E.^1^

##### ^1^UOC Anesthesia, Intensive Care and Pain Therapy, IRCCS- Regina Elena National Cancer Institute, Rome ~ roma ~ Italia, ^[2]^UOC Anesthesia, Intensive Care, Policlinico Umberto I, Sapienza University of Rome ~ roma ~ Italia

###### **Correspondence:** Vacirca R.

Background: high or moderate intensity pain hits more than a half of patients with cancer and is not adequately treated way in 1/3 of this patients . Complexity of cancer pain makes right management difficult and the consequences of an incorrect management are far-reaching in a clinical and social way. A multimodal treatment tailored on the patient, and the evaluation of quality of life correlated to different treatment methodologies, must constitute a decisive element in terms of therapeutic choices.

Over the past 30 years, the World Health Organization (WHO) analgesic step ladder has been used to guide the choices management of cancer-related pain, but in the last years the growth of innovative treatment strategies, led to the need to modulate this rigid yet useful system. Benefits would be obtained with interventional techniques (peripheral neural blockade, neuromodulatory device use, neuro-destructive techniques, and intrathecal drug delivery systems) performed in the initial parts of the treatment cycle (before the third step of the WHO scale), rather than applied according to the WHO scale algorithm. Some authors who adopted this approach, reported reduction in pain duration and less opioid consumption, minimizing the risk of opioid related side effects and an improving the overall quality of life .

Our hypothesis is that early application of interventional techniques in oncological patients has an improving effect in the treatment of chronic cancer pain in terms of efficacy and quality of life.

Materials and methods: patients followed by the Cancer Pain Therapy Service of the Cancer National Institute Regina Elena, Rome, with chronic localizable abdominal pain with a value ≥ 7 according to the numeric rating scale (NRS) and a diagnosis of untreatable disease will be randomized into two groups: in the first group patients will be treated with early interventional neuromodulatory techniques, before high opioids dosages. The other group will follow the steps of the WHO scale.

Every patient will receive the European Organization for Research and Treatment of Cancer Quality of life-Core 30 Summary Score (EORTC-QLQ C30) survey to detect quality of life and the NRS. They will receive it before the treatment, after invasive procedure, one month later and six months later.

Primary end point will be the difference of the quality of life questionnaire score between the groups; secondary end point will be difference in the NRS values. The statistic analysis will be based on two groups of patients responding to the including criteria. The sample will be made of 50 patients divided in two sub-sample of equal dimension to select and analyze in 18 months. The sample thus defined is consistent for a confidence interval of 90% and for a margin of error of less than 5%.

Conclusions: In the last two years cancer pain management has deeply changed and interventional procedures became an important part of multimodal analgesia. This study may allow us to consider aspects that fall within the concept of “total pain”, improving interdisciplinary intervention, quality of life and patient’s well-being.

## Donatori multiorgano e Anestesia e Terapia Intensiva nei trapianti d'organo

### A89. First organ harvest in a small provincial hospital with new intensive care unit

#### Ambrogi F., Russo G., Golino L., Caiazzo M., Pascarella S., Tornincasa E., Imperatore F.

##### Unit of Anesthesia and Intensive Care, San Giovanni di Dio Hospital ~ Frattamaggiore ~ Italia

###### **Correspondence:** Ambrogi F.

**Background**

San Giovanni di Dio Hospital is a first level hospital in Frattamaggiore, (metropolitan area of Naples), in Italy. Our Intensive Care Unit (ICU) has been opened three years ago and our experience has been strongly influenced by the Covid-19 pandemic. Users of our hospital are poorly sensitized about organ donation. In Campania, our Italian region, in 2021 there was a smaller number of organ donor utilized (9,7%) compared to the national average, while oppositions to organ donation was higher than national average in 2020 (37,7%) and smaller than national average in 2021 (27,8%), with about 10% reduction of oppositions compared to the previous year [1]. Brain death assessment and consequent organ harvesting is hard to put in place for a small hospital because of the limited resources which a small hospital has at its disposal, but we did it.

**Case report**

In March 2022, a female patient, age 66, was admitted in our hospital for acute neurological syndrome, with GCS < 8 and anisocoria. In anamnesis: hypertension and history of alcoholism. After analgosedation, she was intubated and started neuroprotection. Brain TC showed a massive intraparenchymal hemorrhage. The neurosurgical consultation did not indicate surgery, so the patient was admitted to ICU. In a few hours the patient showed medium mydriasis and non-elicitable photomotor reflex and hemodynamic instability with needing of pharmacological support. Periodically, sedation suspension windows were performed to carry out the neurological evaluation and the examination of brain stem reflexes. When all brain stem reflexes disappeared, brain death assessment was conducted according to guidelines [2-5]. At the end, the patient was pronounced brain dead. When relatives were interviewed as custodians of the patient's wishes, consent for organ and tissue donation was acquired [3, 5]. The patient was eligible for corneas and liver donation. The organ harvesting took place on the same day in the operating room with a beating heart, thanks to the collaboration of our anesthetists and the surgeons of the team of the Regional Transplant Center.

**Conclusions**

Early diagnosis of brain death, anesthetist’s training to recognize early signs of brain pain and the donor management in ICU and operating room proved to be critical to success. Graduality of communication with family members, empathically conducted, to get the death of their relative accepted and then offer the possibility of organ donation at the appropriate time, proved to be important in acquiring their consent. Sensitize the population to the issue of organ donation can limit oppositions and increase the number of donations. In this regard, we feel important the knowledge of the principles of brain death even for people who do not belong to medical and nursing staff. The process of organ donation represents a constant challenge for our anesthetists, both for the need to stay constantly updated and for the management difficulties. Our first organ harvesting represents a milestone for us and outlines a success to be pursued with the increase in donors’ number by our hospital, to raise our quality standards and offer to users.

Informed consent to publish had been obtained.

**References**

1. Attività di donazione e trapianto di organi, tessuti e cellule staminali emopoietiche - Sintesi del Report 2021 - https://www.trapianti.salute.gov.it/imgs/C_17_cntPubblicazioni_463_allegato.pdf

2. Malacarne P, Livigni S, Vergano M, Gristina G, Mengoli F, Borga S, Baroncelli F, Riccioni L, Latronico N et al. Le cure di fine vita e l’anestesista rianimatore: raccomandazioni SIAARTI per l’approccio alla persona morente. Update 2018.

3. Decreto del Ministero della Salute 11 aprile 2008 n 136, GU della Repubblica Italiana 12 aprile 2008.

4. Centro nazionale trapianti: Manuale Corso Nazionale Coordinatori alla Donazione e al Prelievo di Organi e Tessuti, VII Edizione 2012. Pp 151.

5. Nardi G, De Blasio E, Ciraolo R. Linee guida per un Centro di Rianimazione… vent’anni dopo. Terza edizione. Antonio Delfino Editore. Pp 377-395.

### A90. Pediatric domino liver transplantation: anesthesiological management of a rare case study

#### Belmonte G.^1^, Landini S.^1^, Bonaiti S.^2^, Mulas E.^2^, Bossi C.^2^, Giordano C.^2^, Petrò L.^2^, Rasella B.^2^, Benigni A.^2^, Cadei M.^2^, Starita G.^2^, Pirola C.^2^, Mario C.^3^

##### ^1^Resident of Anesthesia and Intensive Care, Department of Medical-Surgical, Radiological Sciences and Public Health, University of Brescia ~ Brescia ~ Italia, ^2^Anesthesia and Intensive Care Unit, Emergency and Critical Care Department, ASST Papa Giovanni XXIII ~ Bergamo ~ Italia, ^3^Director of Anesthesia and Intensive Care Unit, Emergency and Critical Care Department, ASST Papa Giovanni XXIII, Bergamo, Italy ~ Bergamo ~ Italia

###### **Correspondence:** Belmonte G.

In a domino liver transplantation (DLT), the domino donor receives a traditional liver transplant, typically to correct an underlying metabolic disorder, while providing an otherwise anatomically healthy liver to the domino recipient (figure 1-2). The metabolic disorder of the donor would not manifest in the recipient due to sufficient compensation by the presence of the appropriate enzyme in the remainder of the body. An extraordinary pediatric DLT in the Hospital "Papa Giovanni XXIII" of Bergamo was performed. Two selected patients were undergoing this exceptional surgery. The donor patient was a 17-year-old female who had maple syrup urine disease (MSUD), a rare metabolic syndrome, characterized by progressive hepatic encephalopathy and cognitive impairment. Conventional treatments consisted in a protein-restricted diet, with supplementation of essential amino acids. Liver transplantation is curative because it allows sufficient metabolic activity and a normal diet. The recipient patient was a 10-year-old female that presented Joubert Syndrome and an idiopathic liver cirrhosis with portal hypertension. Once written informed consents were obtained, the patients underwent pediatric DLT. In both patients, we have induced intravenous general anesthesia according to hospital protocol: fentanyl 2 mcg/kg, midazolam 0,2 mg/kg, propofol 3 mg/Kg, cisatracurium 0.02 mg/ Kg and vitamin K 1mg/kg. An intra-arterial catheter to continuously monitor BP, central venous catheter, bispectral index (BIS) and cerebral oximetry using near infrared spectroscopy (NIRS) were positioned. The maintenance of general anesthesia was obtained with lower minimum alveolar concentration (MAC 0.6) of sevorane, cisatracurio 2mcg/kg/min, remifentanil 0,08-0,15 mcg/Kg/min and calcium chloride 6 mg/kg/h, while acid base and electrolyte balances were corrected. Furthermore the donor patient required a continuous perioperative infusion treatment of 20% lipofundin 10 ml/h and 10% Glucose 130 ml/h and aminoacidemia dosage every six hours . Thirty minutes before vena cava declamping, we administered metilprednisolone 10 mg/kg. Concerning fluid therapy, we maintained euvolemia administering crystalloids at the speed of 10 ml/Kg/h and 3 ml/Kg of 5% albumin. In the pre-anhepatic phase, to achieve the target values of mean arterial pressure (MAP) between 60 and 75 mmHg and central venous pressure (CVP) between 8 and 12 mmHg, we started a continuous infusion of norepinephrine (0.03-0.15mcg/kg/min) and epinephrine (0.03-0.10mcg/kg/min). In the neo-hepatic phase, hemodynamic stability was observed and vasopressor requirements were quickly decreased in both patients until the amine infusion was stopped. Diuresis always remained valid, with no need to be stimulated with mannitol or furosemide. At the end of the surgery, the correction of coagulopathy, according to the Thrombelastograph (TEG), was not needed and blood glucose and lactate levels were within normal limits (Table 1; Figure 3). Thus, our results show excellent liver function on admission to the Intensive Care Unit, with optimal clinical outcome. The excellent results obtained and the absence of the onset of new metabolic diseases show that DLT could be a lasting success in pediatrics.

**Fig. 1 (abstract A90).**
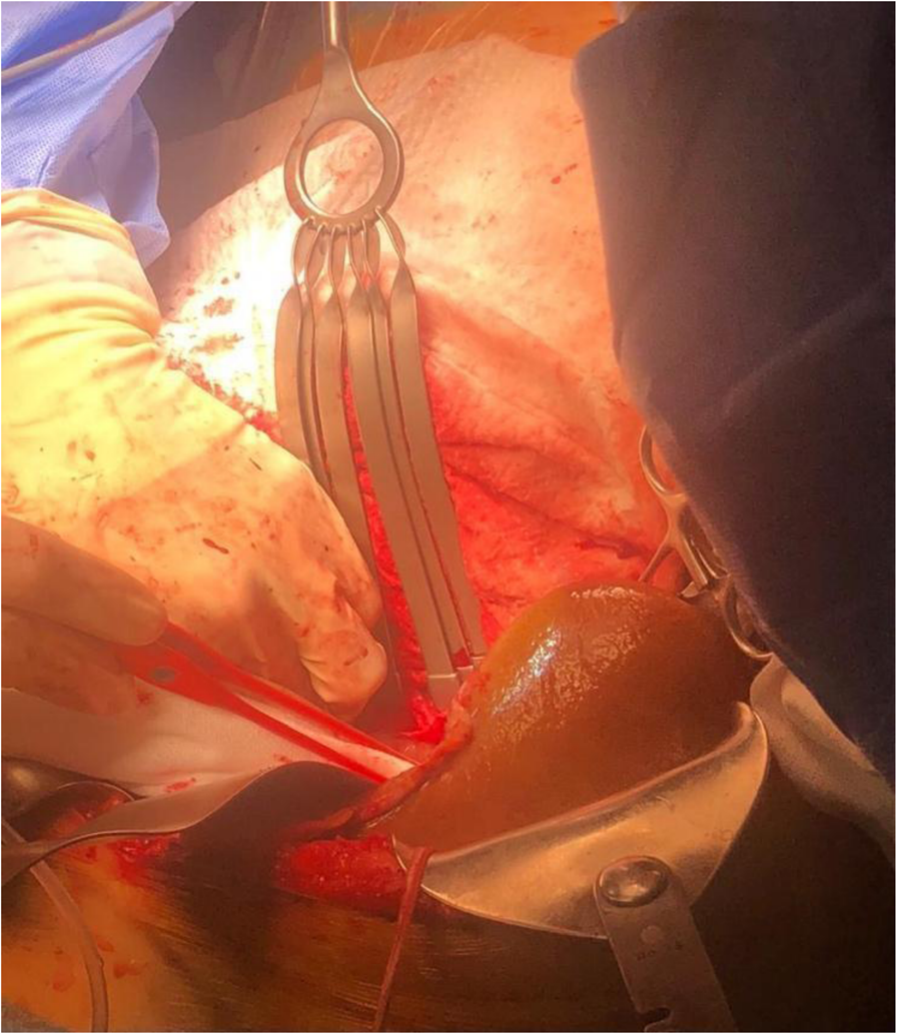
See text for description

**Fig. 2 (abstract A90).**
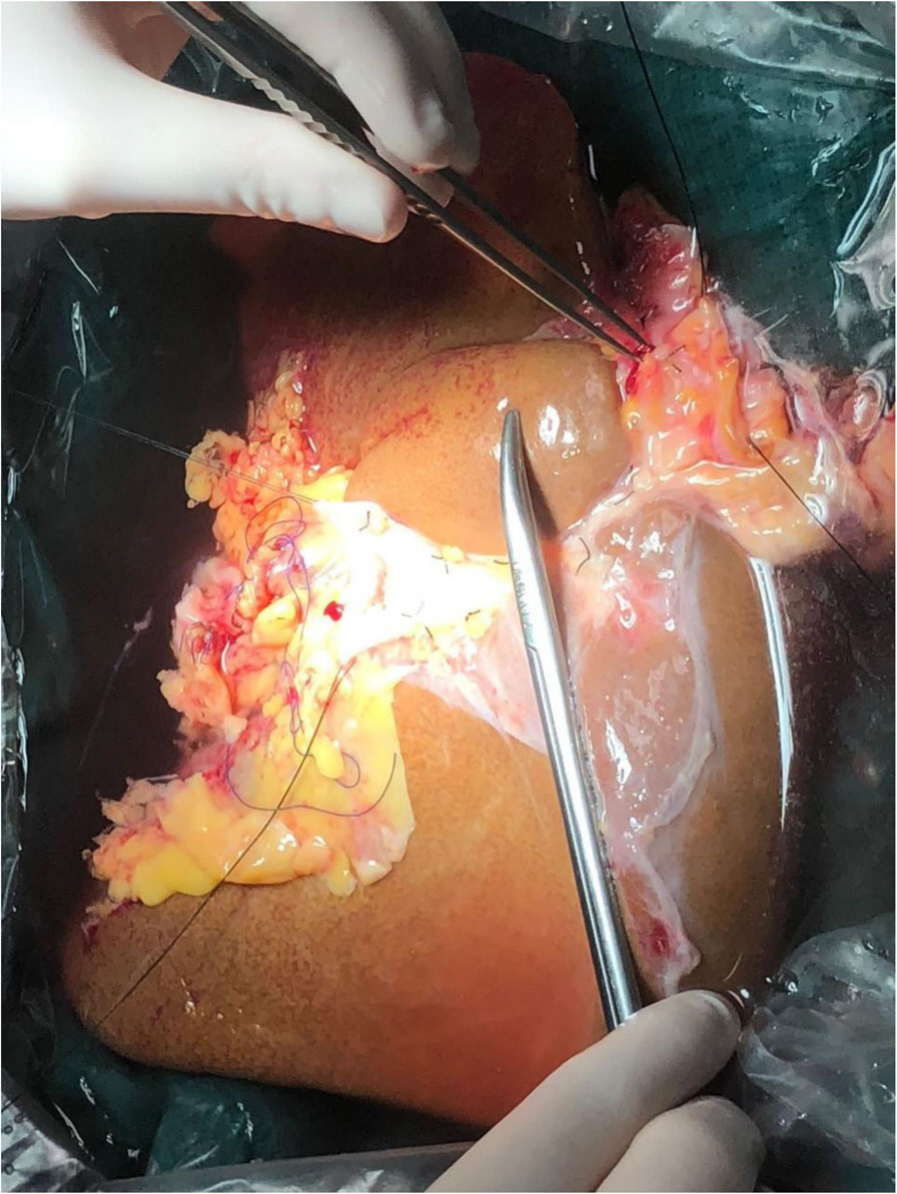
See text for description

**Fig. 3 (abstract A90).**
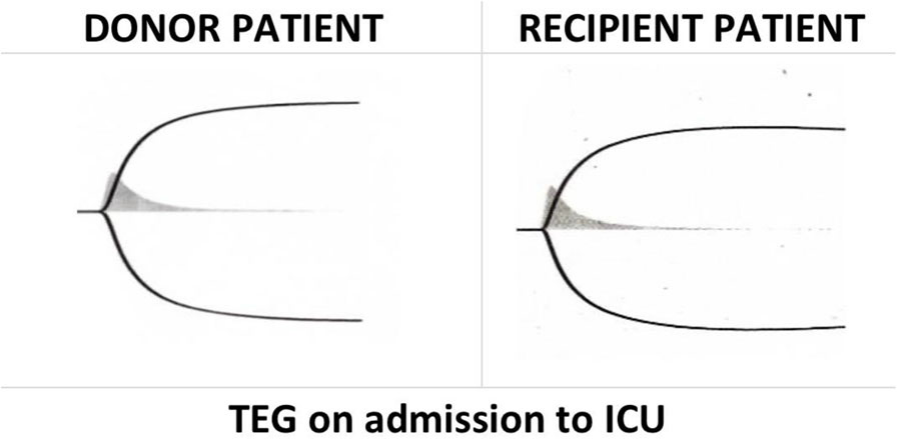
See text for description

**Table 1 (abstract A90).** See text for description

	Donor Patient	Recipient Patient
**PH**	7,28	7,39
**Glicemia**	200 mg/dl	194 mg/dl
**Lactates**	2,65 molto/L	2,04 mmol/L
**Epinephrine**	STOP	STOP
**Norepinephrine**	0,05 mcg/kg/min	STOP

## Emergenze subacquee e iperbariche

### A91. Heavy breathing: a prospective cohort study of pulmonary function following hyperbaric oxygen therapy

#### Brenna C.^1^, Khan S.^1^, Au D.^1^, Schiavo S.^1^, Wahaj M.^2^, Janisse R.^2^, Katznelson R.^1^

##### ^1^Department of Anesthesiology & Pain Medicine, University of Toronto ~ Toronto ~ Canada, ^2^Hyperbaric Medicine Unit, Toronto General Hospital - UHN ~ Toronto ~ Canada

###### **Correspondence:** Schiavo S.

Introduction

Hyperbaric oxygen therapy (HBOT) involves exposure to supra-atmospheric pressures of oxygen, which may lead to oxygen toxicity and impair pulmonary function. The present study seeks to evaluate serial changes in pulmonary function among patients undergoing HBOT.

Methods

This prospective cohort study’s protocol was registered through the US National Library of Medicine. Patients undergoing HBOT from 2016-2021 at a large referral centre in Toronto, ON, underwent pulmonary function testing with a bedside spirometer/pneumotachometer prior to treatment and after every 20 sessions. HBOT was performed using 100% oxygen at 2.0-2.4 ATA for 90 minutes, five times weekly, in a hyperbaric chamber. Patients’ charts were reviewed retrospectively for demographics, comorbidities, medications, HBOT specifications, treatment complications, and pulmonary function performance. Primary outcomes were change in percentage of predicted forced expiratory volume in one second (FEV1%), forced vital capacity (FVC%), and forced mid-expiratory flow (FEF25-75%), after 20, 40, and 60 HBOT sessions. Data was analyzed with descriptive statistics and mixed-model linear regression.

Results

Of 160 enrolled patients, 86 were included in the analysis. The study population comprised 33 females and 53 males with an average age of 57, undergoing an average of 42.5 HBOT sessions (totalling 3666). We observed neither statistically- nor clinically-significant changes in FEV1%, FVC%, or FEF25-75% across serial HBOT treatments. Subgroup analyses stratifying the cohort by pre-existing respiratory disease, smoking behaviour, and treatment pressure similarly did not identify significant impacts of HBOT on pulmonary function.

Conclusions

We found no changes in FEV1%, FVC%, or FEF25-75% after HBOT. Our results suggest that risks of pulmonary oxygen toxicity following HBOT are insubstantial in modern treatment protocols, and pulmonary function is uncompromised by even prolonged courses of HBOT.

**Fig. 1 (abstract A91).**
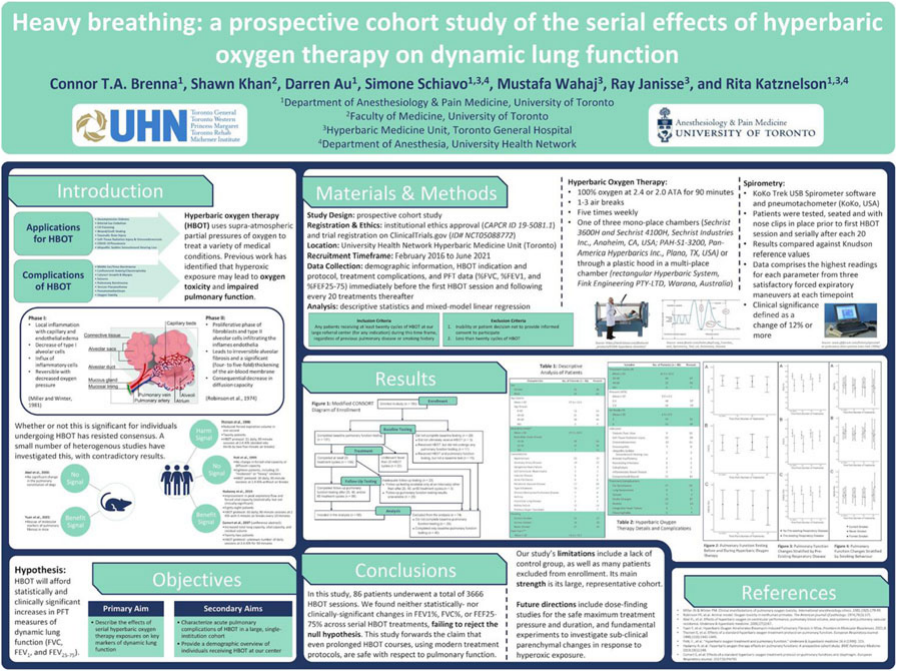
See text for description

## Follow-up/esiti

### A92. Quality of life after recovery from ICU: COVID-19 vs other critical illness. A pilot study

#### Ferrara G.^1^, Rauseo M.^1^, Cardinale F.^2^, Padalino S.^2^, Meola S.^1^, Russo B.^1^, Tullo L.^1^, Al--Husinat L.^3^, Cinnella G.^1^

##### ^1^Policlinico Riuniti, Università degli Studi di Foggia, Anestesia e Rianimazione ~ Foggia ~ Italia, ^2^Università degli Studi di Foggia ~ Foggia ~ Italia, ^3^Department of Clinical Medical Sciences, Faculty of Medicine, Yarmouk University ~ Irbid ~ Jordan

###### **Correspondence:** Ferrara G.

**Background**: After hospital discharge, quality of life (QoL) in medical-surgical ICU survivors is lower than general population. Recent data show that patients affected with COVID-19, after ICU and hospital discharge may present persistence of clinical manifestations such as myalgia, and fatigue together with depressive symptoms that affect patients’ QoL^1^. The aim of this study was to evaluate psychophysical QoL at 3 months after ICU discharge between patients affected by COVID-19 and patients admitted for other causes^2^.

**Materials and Methods**: The study was conducted from September 2021 to April 2022. Three months after ICU discharge, Covid and Non-Covid ICU-survivors underwent for a clinical follow-up of clinical and mental status. Recorded data included: vital parameters at ICU admission and discharge retrieved from medical records, last blood tests collected at hospital discharge, psychophysical tests (SF-36, Barthel index, HADS, Insomnia Severity index, PCL-5 for Post-traumatic disturb, Fatigue severity scale, Physical function in ICU test and Montreal Cognitive) administered the day of follow up. The study team included two senior intensivists, one resident, one medical student and one psychologist. Each evaluation lasted 90 minutes.

**Results**: We enrolled 15 patients (7 post-Covid, 8 Non-Covid). There were no differences at ICU admission between the two groups about sex, age and BMI (p >0.05). There were no differences between the two groups in psychophysical tests (p >0.05). Post-Covid patients had a significantly better nutritional state, general health and change in health (p<0.01; Tab 1).

**Conclusions:** These preliminary data show that ICU COVID-19 patients have a better nutritional state at the 3-months follow-up than patients admitted and discharged from ICU for other causes. Moreover, COVID-19 patients showed a less impaired QoL in terms of wellbeing after their recovery and an overall better perception of general health.

References:

1. Latronico N, Peli E, Calza S, et al., physical, cognitive and mental health outcomes in 1-year survivors of COVID-19-associated ARDS, Thorax, 2021

2. Prevedello D, Fiore M, Creteur J, Preiser JC, Intensive care units follow-up: A scoping rewiew protocol. BMJ Open, 2020; 10(11)

**Table 1 (abstract A92).** See text for description

Mann-Whitney test analysis	ICU COVID-19 pts	ICU Other pts	p-value
Mini nutritional assessment-short form test (SF36)	10 [9-11]	7[6-12]	0.01
SF36-General Health	50 [25-90]	25 [10-40]	0.01
SF36-Change in Health	25 [0-50]	0 [0-25]	<0.01

### A93. Analysis of factors influencing nursing workload in a general ICU using the nursing activities score. A retrospective observational study

#### Pizziconi A.^1^, Di Rocco A.^2^, Lucini A.^3^, Cellamare L.^4^, Lucchini A.^4^

##### ^1^Azienda Ospedaliero - Universitaria di Ferrara ~ Ferrara ~ Italia, ^2^Azienda Sanitaria Regionale Molise - Asrem ~ Termoli ~ Italia, ^3^Centro Cardiologico Monzino ~ Milano ~ Italia, ^4^Ospedale San Gerardo - ASST Monza ~ Monza ~ Italia

###### **Correspondence:** Pizziconi A.

Introduction: The calculation of nursing needs in the intensive care unit (ICU) setting has always been one of the greatest challenges for the nursing profession. Identifying the weight of a patient's care complexity allows, therefore, to quantify the nursing needs proportionate to the satisfaction of his or her needs. Therefore, the objective was to analyze and evaluate through the Nursing Activities Score (NAS) the nursing workload within the General Intensive Care Unit at ASST Monza, taking into consideration three categories of patients (medical, urgent surgical and elective surgical) and to verify which predictive nursing interventions most influence and increase the weight of care load.

Materials and Methods: Retrospective and monocentric observational study conducted at the General Intensive Care Unit of the San Gerardo Hospital in Monza. NAS scores of 2590 patients, admitted from 2014 to 2021, were analyzed.

Results: The mean NAS of the whole analyzed sample turned out to be equal to 66,21±20,82. Determines a higher workload medical patients compared to urgent surgical and elective surgical patients (mean medical NAS (71,82±14,47), mean surgical urgent NAS (67,80±13,48) and average elective surgical NAS (57,37±13,59). The NAS of medical patients varied by pathology type [ECMO 13,77 (11,076 – 16,474), invasive ventilation 8,01 (6.763 – 9,258), vasoactive drugs 8,62 (7,506 – 9,743), Swan Ganz 3.78 (1.481 - 6.04), aortic counterpulsator 3,17 (-7,534 – 13,883), NIV 4,23 (2,771 – 5,7), and tracheostomy -0,25 (-2,439 – 1,931)].

Discussion: The score of medical patients is higher than that of surgical patients. A further subdivision occurs between emergency and elective surgery. In fact, the I:P ratio is 1:3 in electives. ECMO patients find an abundant increase in score. NAS increases as the SAPS II and SOFA scores increase.

Conclusions: Periodically assessing patient workload with the NAS scale allows estimation of the gap between needed and actual staffing. Quantify which patients impact workload the most and objectify the need for care in relation to patient case – mix.

### A94. Post-acute sequelae of COVID-19: 12-months follow-up for adult critically ill survivors

#### Venturi L., Tani C., Bichi L., Spina R.

##### ASL Toscana Centro, S.O.C Anestesia e Rianimazione Ospedale “San Giuseppe” ~ Empoli (FI) ~ Italia

###### **Correspondence:** Venturi L.

Background: Long-term effects of COVID-19 affect more than 10% of patients. This percentage rises in patients who require critical care. Long-term outcomes of the critically ill COVID-19 survivors need to be assessed. The objective of this single-center cohort retrospective study was to describe long-term physical and psychological outcomes, as well as health-related quality of life of critically ill COVID-19 adults who survived intensive care unit (ICU).

Methods: All critically ill COVID-19 adults who survived ICU stay were involved. Patients were visited at 3, 6 and 12 months after ICU discharge. They were evaluated with a standardized assessment at each time of follow-up, addressing health-related quality of life (Glasgow Outcome Scale, GOS), respiratory impairment (Modified British Medical Research Council Questionnaire, mMRC), sleep disorders (0-10 rating scale), fatigue (0-10 rating scale) and mental health disorders (HADS and PCL-5).

Results: Among the 246 patients admitted to our ICU for COVID-19, 158 survived a prolonged ICU and hospital stay and 106 (67%) attended the 3-months follow-up visit. Their median age was 60 years, 79.3% were male, and 51.9% received rehabilitation following hospital discharge. At this follow-up time 74.6% of patients had good recovery (GOS 5) while 25.4% showed a severe to moderate disability (GOS 3-4). Symptomatic patients were 92.4%. 94.9% of them experienced neurological disturbances, 78.5% respiratory impairment and 34.7% psychological symptoms. Fatigue was detected in 78% of cases, and sleep disorders in 68.3%. 65.3% of the patients showed dyspnoea with mMRC≥1. Of the 106 patients who had the 3-month follow-up visit, 93 (87.7%) attended the 6-month evaluation. At this time 81.7% of patients had good recovery (GOS 5) while 18.3% showed a severe to moderate disability (GOS 3-4). 94.6% of the patients were still symptomatic, 86.4% reporting neurological disturbances, 71.6% respiratory impairment and 28.4% psychological symptoms. Fatigue was detected in 69.3% of patients and sleep disorders in 56.8%. Dyspnoea with mMRC≥1 was detected in 50% of cases. At 12 months 48 patients (45.2%) were evaluated. Despite 98% of them having an overall good recovery (GOS 5), 93.7% of the patients were still symptomatic. Neurological, respiratory and psychological disturbances were detected in 73.3%, 48.8% and 17.8% cases respectively. 53.3% of patients referred fatigue and 51.1% sleep disorders. Dyspnoea with mMRC≥1 was detected in 28.9% of cases. Combined symptoms were observed in the majority of cases at each follow-up time.

Conclusion: Despite the majority of patients having an overall good recovery at each time of follow-up, 12 month after ICU-discharge a great part of them did not still get their baseline level of activities back, showing a combination of neurological, respiratory and psychological disturbance. These data are an argument on the need for closed follow-up for critically ill COVID-19 survivors.

### A95. Six- and twelve-month outcome in survivors from critical illness including COVID-19 patients and their family members: a prospective cohort study

#### Minesso R.^1^, Pini S.^1^, Martelli M.^1^, Bertolini R.^1^, Dazzi F.^2^, Corradi F.^2^, Forfori F.^2^

##### ^1^Intensive Care Unit, Emergency Department, Azienda Ospedaliera Universitaria Pisana ~ Pisa ~ Italia, ^2^Intensive Care Unit, University of Pisa ~ Pisa ~ Italy

###### **Correspondence:** Minesso R.

Background: The survival rate of critical patients admitted to the Intensive Care Unit (ICU) has improved in recent years thanks to the advances in expertise and treatment. Despite this positive data, some of the survivors experience impairment in cognition, psychological health, and physical function after treatment in ICUs, which is now recognized as Post Intensive Care Syndrome (PICS). Consequently, also the psychological health of family members of survivors and deceased people may have an acute or chronic worsening, which is recognized as PICS-Family (PICS-F). Moreover, also patients admitted to ICU due to COVID-19 pneumonia may develop the same disabilities, recognized as post-COVID-19 PICS. Even though the syndrome has a relevant impact on the lives of family members and patients, PICS is still often disregarded and therefore not adequately and timely treated. Unfortunately, neglecting this part of care can lead to a reduced quality of life and elevated levels of suffering for patients and their family members.

Materials and methods: A prospective observational cohort study was conducted in the Anesthesia and Intensive Care Unit of Pisa, started in August 2021, and ended in April 2022. Patients were assessed after a 6-month and 12-month period in terms of residual disability (GOSe), overall patient satisfaction with their state of health (QOLIBRI), the perceived health-related quality of life (EQ-5D). At 12 months, symptoms of anxiety and depression (HADS) were assessed too. At 12 months, the caregivers’ symptoms of Post-Traumatic Stress Disorder (PTSS-10) and the burden of caring for the relative (Zarit Burden Interview, ZBI) were studied.

Result: Out of 157 patients contacted after six months, 113 (71.9%) completed the follow-up. In the non Covid group (69 patients) 29% were still severely disabled (GOSe) at 6 months, and 14% perceived their quality of life as poor or bad (QOLIBRI). At 12 months, in the non-Covid group of patients (30), 42% had not returned to their previous job (GOSe) and 6% could not dress and wash themselves independently (EQ-5D). At 6 months, in the Covid group of patients (44), 16% were still moderately disabled (GOSe), and at 12 months 23% stated that they suffered from moderate anxiety or depression (EQ-5D). Moreover, at 12 months, out of 17 interviewed caregivers, 40% perceived a slight burden of caring for the family member (ZBI). 33 patients were interviewed both at 6 months and at 12 months.

Conclusions: After hospitalization, a significant share of patients report mental and cognitive physical disabilities at 6 and 12 months, which negatively impact their quality of life and work activity. At 12 months disabilities remain unchanged in most cases or even worsen in some cases (Figure 1). Family members of ICU patients are also highly likely to develop PTSD symptoms at 12 months after hospitalization.

**Fig. 1 (abstract A95).**
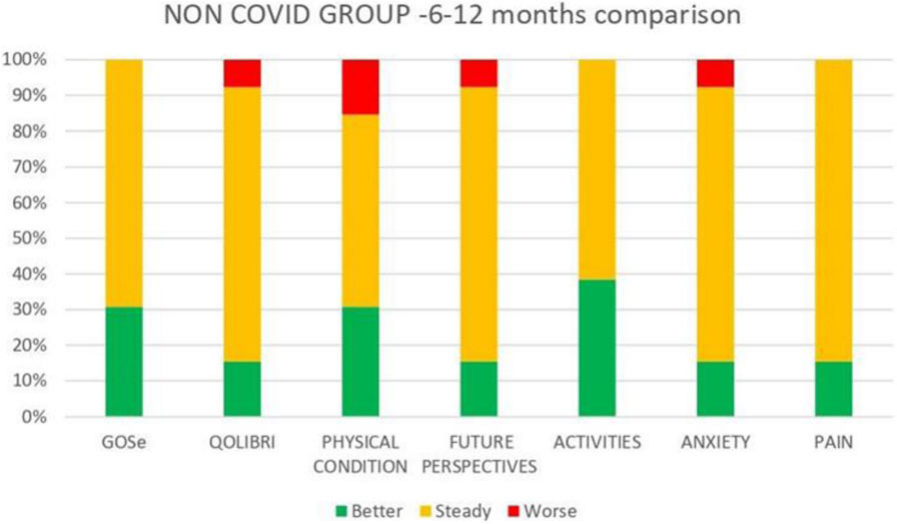
Summary of trends in the non-Covid group

### A96. Tools for assessment of skin risk in intensive care patients: Braden and Jackson Cubbin

#### Riboldi C.^1^, Tamagnini G.^2^, Perez Aguilar V.L.^3^

##### ^[1]^Asst Monza ~ Monza ~ Italy, ^[2]^Policlinico Di Milano ~ Milano ~ Italy, ^[3]^Asst Lariana ~ Cantu' ~ Italy

###### **Correspondence:** Riboldi C.

INTRODUCTION:

Pressure injuries are a largely preventable health problem in all industries health care. Patients admitted to intensive care units (ICUs) are considered ad increased risk of developing pressure injury; in intensive care unit the risk assessment tool most commonly used is the Braden scale as it is considered the most valid. The literature for therapy patients intensive also offers the Jackson / Cubbin scale.

OBJECTIVE:

The work carried out aims to compare the two risk assessment scales, Braden and Jackson / Cubbin, on a sample of patients taken in consideration with certain characteristics to define whether the use of a of the two scales helps to recognize and implement prevention measures by reducing the onset of LDP.

MATERIALS AND METHODS

The study is observational and prospective, conducted on 49 patients recruited in ICU of General Intensive Care at the San Gerardo Hospital in Monza from 1 January 2021 to 31 December 2021.

RESULTS

Through the analysis of the data collected on the patients enrolled in the two groups, it emerged that none variation is statistically significant. The group with 23 patients representing 47% of the sample has a mean Braden scale of 13.16 with a standard deviation of 5.48 and a Jackson / Cubbin scale mean of 23.91 with a standard deviation of 3.77; while the group with 26 patients representing 53% sample has a mean Braden scale of 12.15 with a standard deviation of 2.93 and a scale of

Jackson / Cubbin mean of 23.75 with a standard deviation of 3.14, in both groups the difference is not significant as the p. 0 is not <0.05. In the present study, both scales perform roughly in the same way to area under the measured curve. It is also noted that both scales have a low one specificity and low sensitivity in predicting and / or ruling out the onset of pressure lesions in patients of intensive care.

DISCUSSION

The use of a risk assessment tool is useful for increasing thefrequency and effectiveness of preventive interventions, therefore, determining an evaluation tool is important for intensive care patients as this will increase the quality of nursing care e it will protect patients from unnecessary procedures.

The results obtained from this study show that both scales have the same limitations in the development of pressure injuries; both scales perform more or less in the same way to an area under the measured curve.

CONCLUSIONS

We have come to conclusions that, in the future, it would be desirable to expand the research project by extending it to major clinical realities, too specialists, in order to identify similarities and differences, especially on the basis of the initial diagnosis e the typical specificities of each individual component of the sample examined. Having a more representative sample of patients, a predictive value could be determined more accurately of onset of pressure sores in ICU patients. KEY WORDS Intensive care unite, pressure injury, nursing assesment, critical care patients, Braden scale, Cubbin, Jackson scale.

### A97. Quality of life of COVID-19 patients undergoing ECMO

#### Chreim L.^1^, Bosi C.^2^, Chinali I.^1^, Margosio V.^3^, Lucchini A.^1^, Villa M.^1^, Foti G.^1^

##### ^1^Ospedale San Gerardo di Monza ~ Monza ~ Italy, ^2^Ospedale di Cremona ~ Cremona ~ Italy, ^3^Ospedale di Lecco ~ Lecco ~ Italy

###### **Correspondence:** Chreim L.

Background: The COVID-19 pandemic is one of the biggest public health issues of recent years and its consequences are still unclear. The aim of this study is to analyse the quality of life of COVID-19 patients discharged from the ICU undergoing treatment with intravenous ECMO.

Materials and methods: prospective observational study of a cohort of patients with COVID-19 diagnosis admitted between february 2020 and september 2021 undergoing veno-venous ECMO and included in the Post-Intensive Care Follow Up program. The survey instruments used to assess quality of life were the following: PTSS-10, HADS, EQ-5D-5L, MNA, 6MWT, MRC. The data were obtained through an initial database analysis of the structured Follow Up program. The program was structured by an outpatient visit at 3 and 6 months after discharge and subsequently by a semi-structured telephone interview at 12 months after discharge.

Results: 20 patients were enrolled, 35% (n=7) of whom were women, with a median age of 53 (38- 66) and a BMI of 33.3 (23.2-43.8). The median duration of ECMO treatment was 15 days(4-57) and 90% (n=18) underwent pronation during ECMO. The median PTSS-10 score was 5 (0-42) at 3 months, 5 (0-44) at 6 months and 2 (0-26) at 12 months. That of the HADS was respectively in the three periods 3 (0-10), 3 (0-11) and 2 (0-12) for anxiety; while for depression it was 5.5 (0-13), 6.5 (0-18) and 2 (0-13). The 6MWT was only possible at 3 and 6 months with median distances travelled of 405.5 (150-600) and 420 (405-510) metres respectively. The median MRC score at 3 months was 58 (32-60) and at 6 months 59 (39-60). At 12 months, the nutritional status of the individuals was also assessed and only 17.6 % (n=3) were found to be at risk of malnutrition.

Conclusions: The analysis of these data does not reveal any statistically significant differences in the three periods, and the people who were enrolled for this study did not show any symptoms attributable to the psychological, cognitive and physical deficits that are described in the literature as a direct consequence of the pandemic (PICS, PTSD, anxiety, depression, mobilisation deficits, ICU weakness). Furthermore, the quality of life of these patients is not inferior to that of other persons undergoing ECMO treatment for other types of ARDS.

Key words: ECMO, Follow Up, quality of life.

## Infezioni e sepsi

### A98. Overwhelming post-splenectomy infection (OPSI): case report

#### Zarrillo N., Carbone A., Mattia B., Russo R., Guida A., Perretta D., Francesca M.

##### UOC Anestesia e Rianimazione ~ ASL CASERTA P.O. "San Rocco" Sessa Aurunca ~ Italia

###### **Correspondence:** Carbone A.

**Background:**

Overwhelming Post-Splenectomy Infection (OPSI), also known as post-splenectomy sepsis syndrome, is the major long-term complication of splenectomy. OPSI is a generalized non-specific flu-like prodrome followed by rapid deterioration to full-blown fulminant septic shock within 24-48 hours of the onset. Pneumococcus is the predominant cause of infection post-splenectomy (57-87%); Neisseria Meningitidis, and Haemophilus Influenzae type-B are also common etiologic agents. The prevalence of OPSI following splenectomy is 0.1-0.5%, the mortality rate is 50–70%, and most deaths occur within the first 24 hours. The spleen is crucial in regulating immune homoeostasis through its ability to link innate and adaptive immunity and in protecting against infections. Polysaccharide-specific antibodies activate the complement pathway, thereby promoting the deposition of complement fragments directly on to the capsule and, hence, thrombotic vascular occlusion. This might suggest an association between OPSI and doisseminated intravascular coagulation (DIC). Bacteraemia commonly has an unknown origin; after a brief prodrome characterized by fever, shivering, myalgia, vomiting, diarrhea, and headache, septic shock develops in just a few hours, with anuria, hypotension, hypoglycaemia, and, commonly, purpura fulminans with DIC to multiorgan failure and death.

**Case report:**

We present a case of pneumococcical sepsis-induced purpura fulminans with DIC in an a 54 year old female who have had splenectomy 5 years earlier. The patient received pneumococcal vaccination with in the last five years. The patient arrived in the emergency room following vomiting, diarrhea and subsequent loss of consciousness, she was transferred to the second care at our ICU for the rapid worsening in septic shock and multi-organ failure. At the hospitalization the patient was on mechanical ventilation, not responsive to norepinephrine, anuric and developed in the first 24 hours purpura to the face, upper and lower limbs, and to the trunk until then to manifest flittene and necrosis of the extremities in the days following. Laboratory tests showed a severe coagulopathy with hypopiastrinemia, fibrinolysis, hypofibrinogenemia and alteration of hemostasis, the thromboelastogram showed a Coagulation Index -8.5; the white blood cells were 20.1- PCT>100 -Fibrinogen 70- PLT 61- Creatinine 3.44- Glycemia 72- GOT/GPT 292/182, with normal values of HB and HTC. Broad-spectrum antibiotic therapy with ceftaroline and meropemen was initiated after sampling for culture tests on blood, feces and urine, administration of Ig with high IgM content (Pentaglobin) and treatment of CRRT/HDF in anticoagulation with HP with adsorbent filter oXiris for cytokine removal. A streptococcus pneumoniae was isolated from the blood culture. The patient was extubated after about 30 days and subsequently discharged. The skin lesions after excision of the necrotic areas and dressings are eandate to undergo progressive healing without the need for amputations.

**Conclusion:**

The risk of OPSI in splenectomised patients is more than 50-times higher than in the general population. OPSI is a medical emergency for which only prompt diagnosis and immediate treatment can reduce mortality. The clinical course of OPSI is measured in hours rather than days. The timely identification of OPSI, its immediate management, and vigilant care of splenectomized patients provides them with a better chance of survival.

**Informed consent to publish had been obtained.**

**REFERENCES**

*1. Lorry G. Rubin, M.D., and William Schaffner, M.D. Care of the Asplenic Patient N Engl J Med 2014;371:349-56. DOI: 10.1056/NEJMcp1314291*

*2. Prabhu Dayal Sinwar Overwhelming post splenectomy infection syndrome e Review stud. International Journal of Surgery 12 (2014) 1314e1316 http://dx.doi.org/10.1016/j.ijsu.2014.11.005*

*3. Tahir et al.Post-splenectomy Sepsis: A Review of the Literature. Cureus 12(2) -2020: e6898. DOI 10.7759/cureus.6898*

*4. Luu S, Spelman D, Woolley IJ: Post-splenectomy sepsis: preventative strategies, challenges, and solutions . Infect Drug Resist. 2019, 12:2839-2851. Accessed: February 5, 2020:*

*5. Premawardena C, Bowden D, Kaplan Z, Dendle C, Woolley IJ: Understanding of the significance and health implications of asplenia in a cohort of patients with haemaglobinopathy: possible benefits of a spleen registry. Hematology. 2018, 23:526-530. 10.1080/10245332.2017.1414910*

*6. Leandro Utino Taniguchi, Mario Diego Teles Correia, and Fernando Godinho Zampieri Overwhelming Postsplenectomy Infection: Narrative Review of the Literature SURGICAL INFECTIONS Volume 15, Number 6, 2014 DOI: 10.1089/sur.2013.051*

**Fig. 1 (abstract A98).**
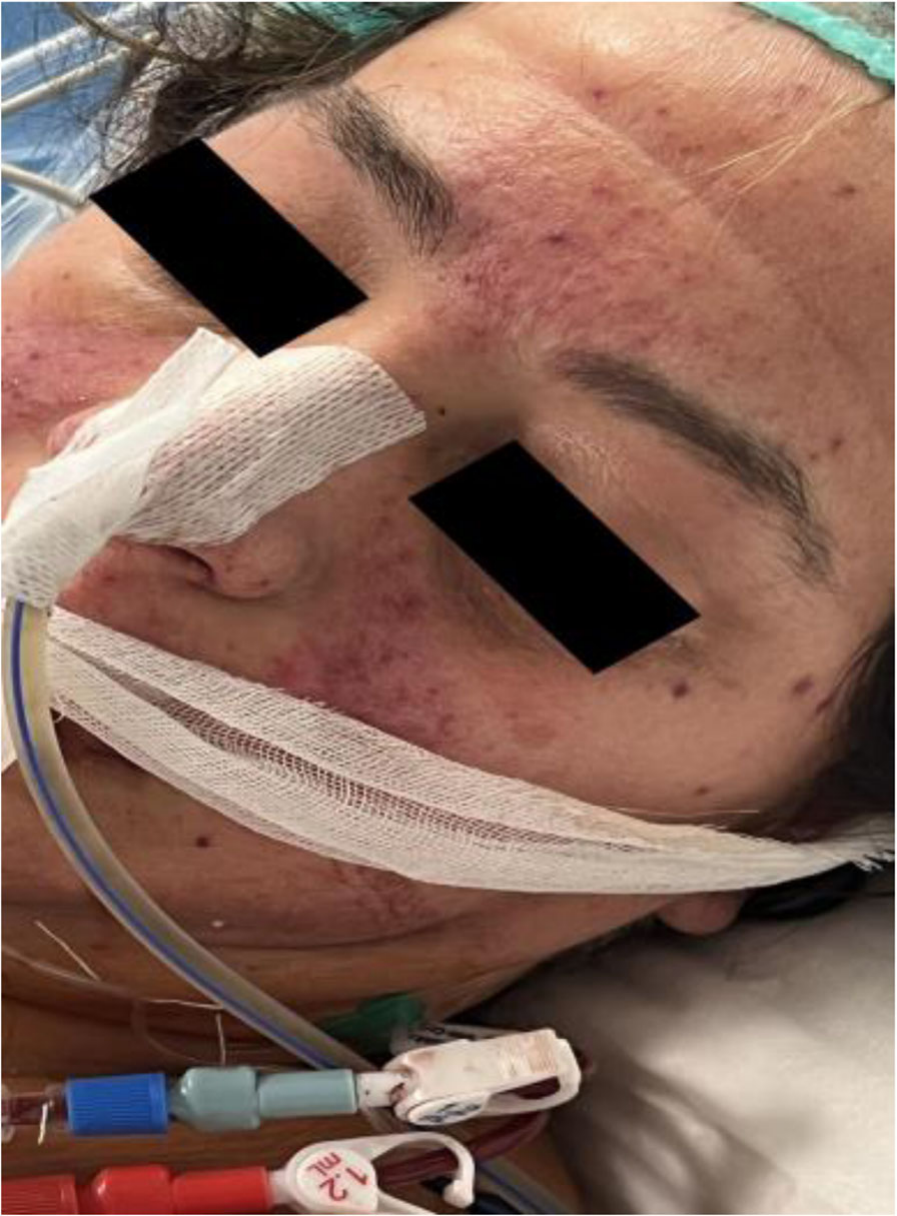
See text for description

**Fig. 2 (abstract A98).**
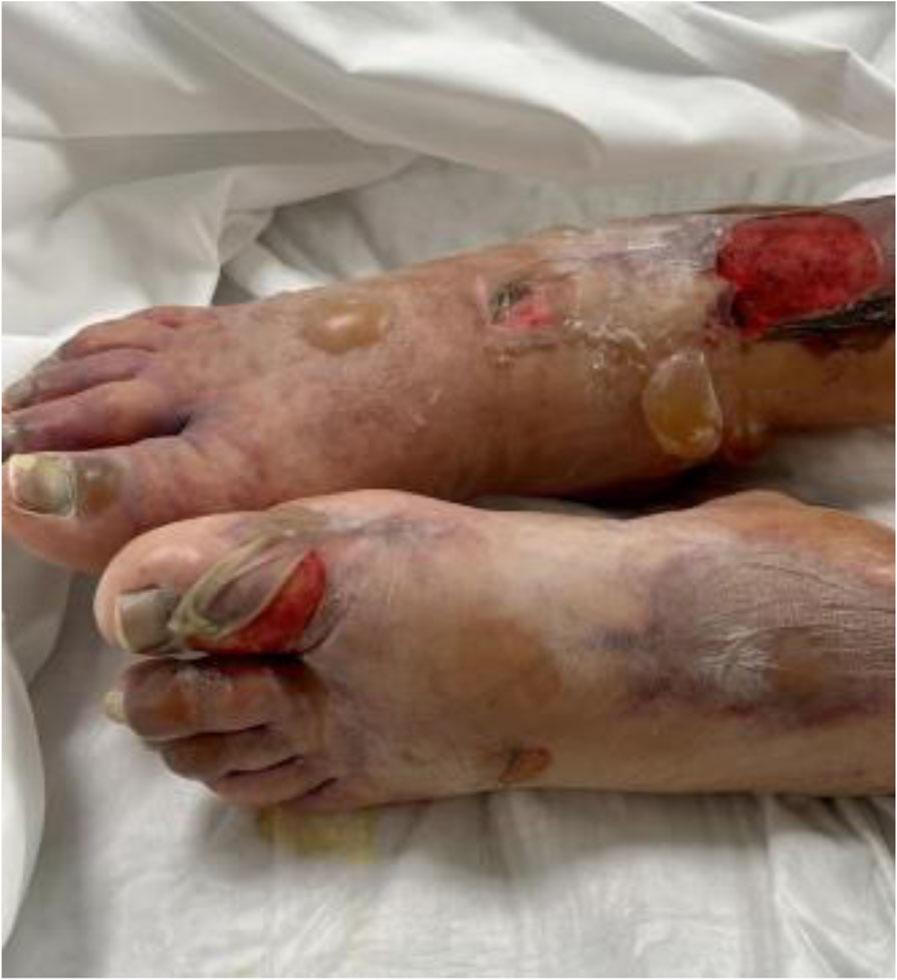
See text for description

**Fig. 3 (abstract A98).**
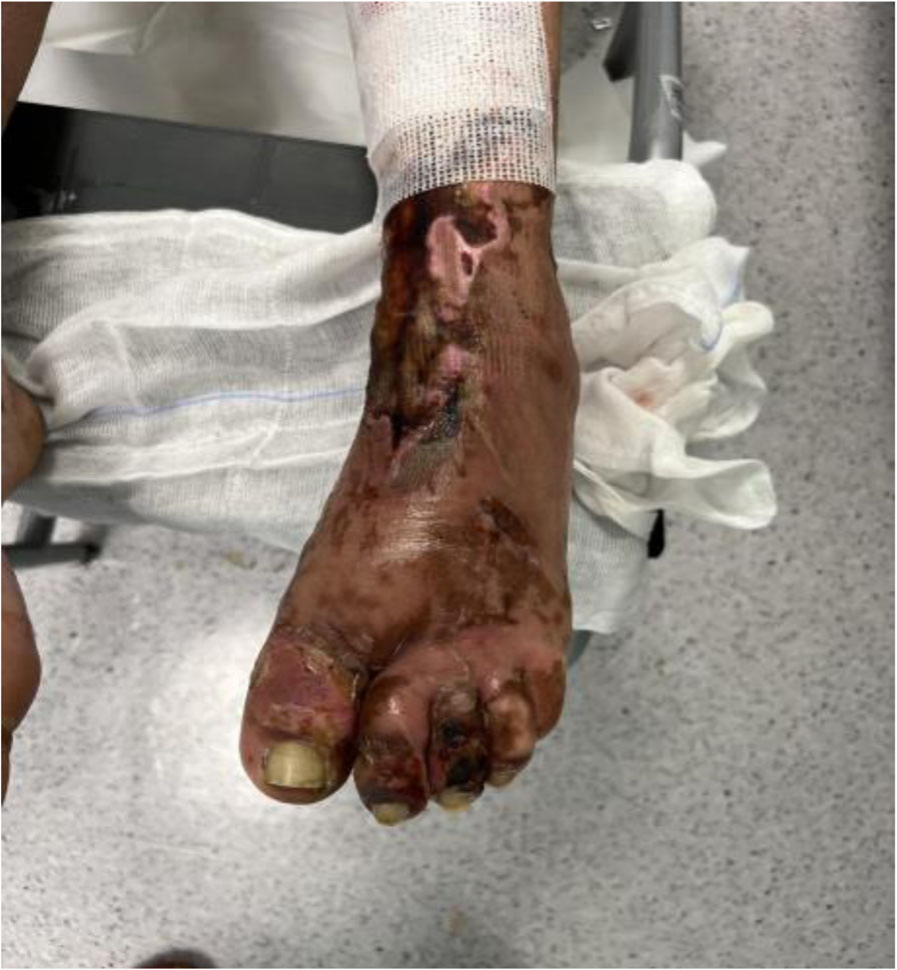
See text for description

### A99. Septic shock and meningitis: let’s not forget leptospirosis!

#### Giurazza R., Minucci C., Capuano M., Acanfora M., De Rosa R.C.

##### AORN dei Colli-Cotugno Napoli – UOC Anestesia, Rianimazione e Terapia Intensiva ~ Napoli ~ Italia

###### **Correspondence:** Giurazza R.

A 38-year-old man, with history of previous adult Still’s disease, HCV-hepatitis and Hashimoto’s thyroiditis, was admitted to the emergency department with fever, chills, hypoglycemia and new onset refractory tonic-clonic seizures. He was transferred to our ICU with a diagnosis of sepsis of unknown origin. Upon arrival patient was intubated and haemodynamically unstable. Physical examination not significant except for bilateral hemorrhagic conjunctivitis. The patient met criteria for septic shock, so a crystalloid fluid challenge and norepinephrine infusion were started. The laboratory exams revealed elevated procalcitonin and C-reactive protein, along with signs of liver impairment and rhabdomyolysis. We performed multiple cultures, all resulted negative, as well as a rachicentesis with clear CSF (22 cells/μL, elevated proteins, normal glucose; negative culture and film array). We started an empirical broad-spectrum antibiotic therapy with ceftriaxone 2 g q12h, ampicillin/sulbactam 2/1 g q6h and fosfomycin 4 g q6h. On day 3, patient was still sedated and intubated. Physical examination of the abdomen revealed an acute abdomen, with a bedside ultrasound and CT-scan showing images of inflamed appendix with signs of perforation. Therefore, the patient urgently underwent diagnostic laparoscopy with appendicectomy. Except for persistence of signs of liver impairment, the postoperative period had a positive trend that ended, on day 8, with extubation. Nevertheless, the extubated and non-sedated patient showed positive meningeal signs, so that a second rachicentesis was done: clear CSF, without cellularity or physical and chemical alterations, negative cultures and film array. An EEG revealed bilateral frontal-central epileptiform abnormalities. The only significant laboratory element for diagnostic purposes was an increase of specific anti-Leptospira IgM antibodies between acute and convalescent serum specimens: leptospirosis! The course of the disease was gradually positive from a systemic and neurological point of view. On day 19, the patient was transferred to a non-ICU ward in our hospital and subsequently was discharged home on day 40. Informed consent to publish had been obtained by the patient.

Although very infrequent in our regions, leptospirosis is considered the most common zoonosis worldwide, caused by the bacteria Leptospira. The natural course of the disease typically evolves in a two-phase manner: (1) an acute septicemic phase of 5-7 days, followed by 1-3 days of improvement, and (2) a delayed systemic inflammatory phase, with recurrence of fever, myalgias, headache and, in rare cases, aseptic meningitis, which is the most important complication of this stage. The typical conjunctival suffusion occurs early in the course of the infection; in some rare cases both rhabdomyolysis and appendicitis have been reported [1,2]. A thorough medical history is crucial to suspect and diagnose leptospirosis and start prompt appropriate antibiotic treatment.

**References:**

1. Rajput D, Gupta A, Verma SS, Barabari GS, Wani AA, Kumar N. Jaundice and Thrombocytopenia in an acute abdomen with concurrent Appendicitis and spontaneous Rectal perforation: An unusual presentation of human leptospirosis. Trop Doct. 2021;51(3):427-431. doi:10.1177/0049475520981298

2. Coursin DB, Updike SJ, Maki DG. Massive rhabdomyolysis and multiple organ dysfunction syndrome caused by leptospirosis. Intensive Care Med. 2000;26(6):808-812. doi:10.1007/s001340051252

### A100. Lymphomatous leptomeningitis is rare but possible!

#### Falso F., Anfora R., Barberio M., Garzia R., De Rosa R.C.

##### AORN dei Colli-Cotugno Napoli – UOC Anestesia Rianimazione e Terapia Intensiva ~ Napoli ~ Italia

###### **Correspondence:** Falso F.

We describe the case of a 50-year-old Caucasian man suffering from high-grade follicular lymphoma and admitted to the emergency department with clinical picture similar to meningitis (severe nuchal headache, vomiting and altered mental status) occurred 48 hours after chemotherapy.

Physical examination showed: Glasgow Coma Scale of 9/15, photophobia, meningismus (lateral recumbency, slight nuchal rigidity, positive Kernig's and Brudzinski’s signs). After brain CT scan (absence of cerebral edema), rachicentesis was performed with aspiration of turbid cerebrospinal fluid. Laboratory analysis of CSF showed pleocytosis with 743 cells/μL (99.3% mononuclear cells), slightly elevated proteins and hypoglycorrhachia, but negative film array. Therefore, an empiric broad-spectrum antibiotic therapy was started *ad interim* with meropenem 2 g tid, linezolid 600 mg bid, fluconazole 400 mg od, acyclovir 750 mg tid, gentamicin 5 mg/kg od. Dexamethasone 4 mg tid was immediately started. Elevation of WBC (up to 34.870/μL with about 95% neutrophils), negative procalcitonin and C-reactive protein, negative multiple cultures (blood, urine, sputum, rectal and nasopharyngeal swab) were observed. HSV-1-DNA, HSV-2-DNA, CMV-DNA, EBV-DNA, HHV6-DNA, HHV8-DNA, parvovirus B19-DNA and VZV-DNA were tested, but their results were negative as well.

The brain CT-scan showed bilateral frontoparietal leptomeningeal contrast enhancement, compatible with leptomeningitis; a subsequent brain MRI confirmed these findings and showed no intracranial masses, thus highlighting suspicion for lymphomatous meningitis.

On day 3, after significant and quick clinical improvement (always valid vital signs and neurological improvement already on the day 2 with GCS 14/15) and negative results of CSF cultures, antimicrobial de-escalation was done and, on day 5, the patient was transferred to the oncohematology ward for prosecution of cure without antibiotic therapy. A few days later, a second rachicentesis with cytofluorimetry and immunophenotyping was performed and confirmed the diagnosis of lymphomatous meningitis. Informed consent to publish had been obtained by the patient.

Lymphomatous meningitis is a rare (5%) secondary manifestation of systemic leukemia or lymphoma (more frequently non-Hodgkin’s); more infrequently it occurs in patients with primary CNS lymphoma. Risk factors include high-grade histology (e.g. Burkitt’s lymphoma), elevated serum LDH and extranodal disease [1]. The most common clinical features are headache, altered mental status, seizures and cranial nerve involvement. Diagnosis is suspected clinically and through brain imaging, and it is confirmed through CSF analysis, showing pleocytosis, elevated protein and decreased glucose levels. Treatment of the disease is palliative, with very low long-term surviving rates, and includes radiotherapy and intrathecal chemotherapy [2].

**References:**

1. Chamberlain MC. Lymphomatous meningitis as a presentation of non-Hodgkin lymphoma. Clin Adv Hematol Oncol. 2011;9(5):419-420.

2. Murthy H, Anasetti C, Ayala E. Diagnosis and Management of Leukemic and Lymphomatous Meningitis. Cancer Control. 2017;24(1):33-41. doi:10.1177/107327481702400105

### A101. Risk factors correlated with mortality in COVID-19 patients during the first pandemic wave

#### Del Gaudio C.^1^, Forfori F.^2^, Isirdi A.^2^, Corradi F.^2^, Malacarne P.^2^, Tonini S.^3^

##### ^1^Università di Medicina e Chirurgia ~ Pisa ~ Italia, ^[2]^Dipartimento di Anestesia e Rianimazione, Ospedale Universitario ~ Pisa ~ Italia, ^3^Istituto di Economia, Scuola Superiore Sant'Anna ~ Pisa ~ Italia

###### **Correspondence:** Del Gaudio C.

INTRODUCTION: The Early identification of high risk patients is of great importance in novel coronavirus disease 2019 (COVID-19).

MATERIALS AND METHODS: We conducted a retrospective, single-centre observational study that included patients admitted to the AOUP's interdepartmental ICU witch covid-19 pneumonia, between March and June 2020. A database was created that includes 26 variables: leukocytes, lymphocytes, neutrophils, NLR, PCR, LDH, CPK, D-dimer, haemoglobin, haematocrit, platelets, troponin, fever, lactates, comorbidities (hypertension, diabetes type II, obesity, cardiovascular and pulmonary diseases, hyperlipidaemia), demographic data (sex and age) and therapeutic devices (IOT, IOT duration, vasopressors and tracheotomy). Was selected the worst value recorded in the first 24 hours after admission to intensive care. Three different statistical analyses were conducted to identify mortality predictors; the descriptive analysis divided the patients into two groups (alive and dead) and was performed a Chi-square test on the ratios for the binary variables and a Willcoxon test on the medians for the continuous variables. Then were applied two supervised learning statistics methods: Random Forest and LASSO. Random Forest is a non-parametric method that orders variables according to their importance in prediction. LASSO is a penalised regression that returns a non-zero value for the relevant variables

RESULTS: 64 patients has been included in the study, the median age was 64,5 years and 49 (76,56%) were male. All three statistical analyses agreed in identifying certain variables as risk factors for mortality and these are: age, hypertension, cardiovascular disease, diabetes type II, hyperlipidaemia, lymphocytes, IOT. (Tabella 1) (Figura 1)

**Table 1 (abstract A101).** List of variables found to be both significant in the descriptive analysis and predictive according to LASSO. A negative coefficient means that as the variable increases, the probability of mortality increases, while a positive coefficient means that as the variable decreases, mortality increases

Variables	Descriptive analysis (*p*-value)	LASSO (Coefficient)
Age	0,0008	-0,6900
Hypertension	0,0275	-0,0891
Cardiovascular disease	0,0205	-0,2909
Diabetes	0,0610	-0,2431
Hyperlipidaemia	0,0940	-0,2796
Lymphocytes	0,0145	0,2975
IOT	0,0035	-0,8340

**Fig. 1 (abstract A101).**
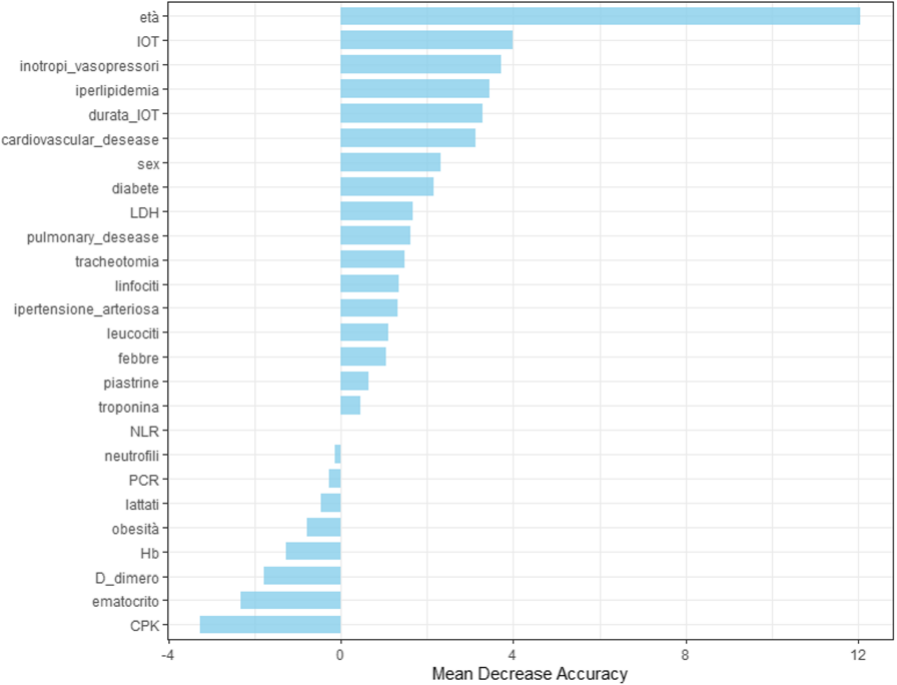
See text for description

### A102. COVID19 associated pulmonary aspergillosis (CAPA) in critically ill patients: epidemiology and outcome

#### Caciagli V., Farinelli C., Gatto I., Biagioni E., Busani S., Talamonti M., Girardis M.

##### AOU Policlinico di Modena ~ Modena ~ Italia

###### **Correspondence:** Caciagli V.

BACKGROUND

Patients with acute respiratory distress syndrome (ARDS) due to viral infection are at risk for secondary complications like invasive pulmonary aspergillosis (IPA). COVID19-associated invasive pulmonary aspergillosis (CAPA) is defined as an IPA in temporal proximity to a preceding SARS-CoV-2 infection. Our study evaluates the incidence and outcome of CAPA in patients admitted to ICU.

METHODS

A retrospective observational cohort study of all adults with microbiologically confirmed SARS-CoV-2 infection admitted to the three COVID19-Intensive Care Units (ICU) at the University Hospital of Modena from February 2020 to June 2021. The cases were classified according to the recent consensus definitions on CAPA (Koehler et al. Lancet Infect Dis 2021)

RESULTS

Possible CAPA was diagnosed in 91 (20,7%) of the 440 patients enrolled in the study. Anti-fungal therapy was initiated only in 45 patients (49,5%) out of 91. In patients with possible CAPA the mortality rate at day 90 was larger (54,9%) than in patients without CAPA (25,5%). The Cox regression analysis adjusted for confounders did not show any relationship (p<0,05) between CAPA occurrence and 90-day mortality.

CONCLUSIONS

Our data indicated that CAPA is common in critically ill COVID-19 patients, but it does not influence patient mortality.

### A103. Outcome comparison between 1st and 2nd pandemic wave in patients with SARS-COV-2 Pneumonia treated with CPAP helmet in non-intensive care wards in varese: observational retrospective study

#### Ferrari F., D”Onofrio D., Da Maccallè M., Selmo G., Guzzetti L., Rossini G., Cozzi S., Ghislanzoni L., Marangoni F., Carollo M., Lanza C., Marcato A., Toso F., Biasoli S., Martignoni F., Bacuzzi A.

##### Ospedale di Circolo - Fondazione Macchi. Dipartimento di Anestesia ~ Varese ~ Italia

###### **Correspondence:** Ferrari F.

**Background**: Since the end of February 2020, Sars-COV-2 virus has spread in the world through a series of epidemic waves. This work compares the hospital mortality between patients hospitalized for respiratory failure due to Covid-19 in Varese during the 1^st^ and 2^nd^ waves.

**Materials and methods**: We considered patients with respiratory failure caused by Sars-COV-2, treated with CPAP helmet during the 1^st^ epidemic wave (from 2/2020 to 4/2020: 163 patients) and the 2^nd^ one (from 10/2020 to 01/2021: 471 patients), who were hospitalized in non-intensive Covid wards of Circolo Hospital in Varese (ITALY). We compared the expected death values, gained from calculation of "4C mortality score" [1], and the actual death values. Additionally we analyzed mortality rate stratified by age.

**Results**: In **Table 1** we calculated the estimated mortality in both waves according to “4C mortality score” while **Table 2** shows the rates of subjects who required transfer to ICU and the corresponding mortality rate. We also reported the value of in-hospital mortality and in particular the rate and number of deaths recorded during the two waves in the Covid wards. The gained P-value shows a statistical significance about In-hospital mortality rate.

The graphs (**Figure 1**) illustrate the comparison between the distribution of survived patients (orange) and dead ones (blue) as a function of age.

**Conclusions**: Expected mortality of the two samples was higher (61.5-66.2%) than actual mortality (31% for the 1^st^ wave, 43% for the 2^nd^ wave). These data support the commendable work carried out by our public health system. The conflicting data are about mortality rate among the 2^nd^ wave (43%) and 1^st^ one (31%). These results are in contrast to the literature [2], whereby the 1^st^ wave was the most lethal; it may be explained by the huge incidence of new Sars-Cov-2 infection cases (till over 250 daily cases / 100 thousand inhabitants) in Varese (ITALY) during the 2^nd^ wave. Furthermore, almost all of the hospitalized patients had the Sars-COV-2 Delta variant, characterized by an intrinsic high mortality due to a several clinical presentation at hospital admission (PaO_2_/FiO_2_ ratio: 240 in the 1^st^ wave vs. 134 in the 2^nd^ wave - Pvalue <0,001). The Figure 1 shows, according to literature [3], that advanced age is associated with a poor prognosis.

**References**:

1. Knight S, Ho A, Harrison E. Risk stratification of patients admitted to hospital with covid-19 using the ISARIC WHO Clinical Characterisation Protocol: development and validation of the 4C Mortality Score. BMJ. 2020; 370: 1-13

2. Chirico F, Nucera G. COVID-19 mortality in Italy: The first wave was more severe and deadly, but only in Lombardy region. Journal of Infection. 2021; 83: e16

3. Levin A, Hanage W, Meyerowitz-Katz G. Assessing the age specificity of infection fatality rates for COVID-19: systematic review, meta-analysis, and public policy implications. European Journal of Epidemiology. 2020; 35: 1123–1138

**Fig. 1 (abstract A103).**
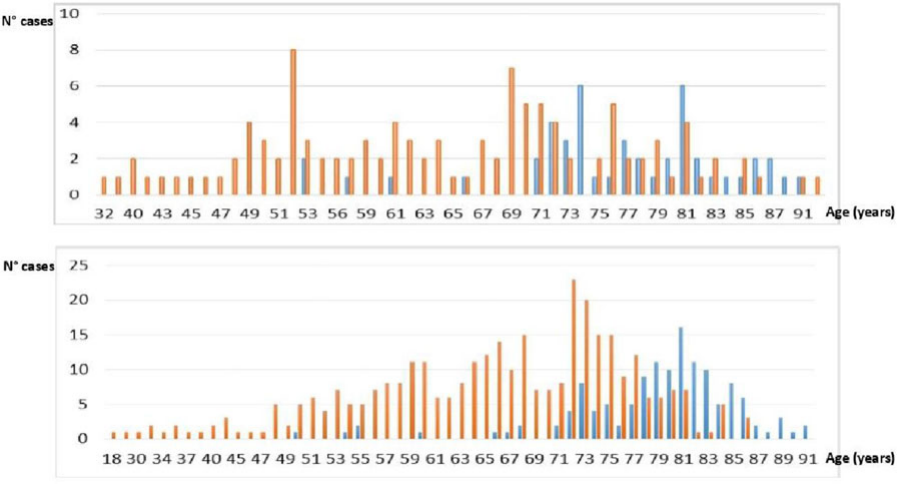
1^st^ and 2^nd^ wave: distribution of survived (orange) and dead (blue) patients as a function of age

**Table 1 (abstract A103).** “4C mortality score” calculation in the two waves

	1^**st**^ Wave	2^**nd**^ Wave
Age	71 yo	72 yo
Sex	M	M
Comorbility	≥2	≥2
Respiratory Rate	28	25
SpO_2_	91%	91%
Glasgow Coma Scale	<15	<15
Urea	42 mg/dl	52 mg/dl
PCR	122 mg/dl	87 mg/dl
**TOTAL**	**61,5-66,2% (17 points)**	**61,5-66,2% (16 points)**

**Table 2 (abstract A103).** Number of patients admitted to ICU and mortality rate

	1^**st**^ Wave	2^**nd**^ Wave	P-Value
**Transfer to ICU**	18% (29)	20% (93)	0,67
**ICU mortality**	51% (15)	77% (72)	**<0,0001**
**In-hospital mortality**	31% (51)	43% (206)	**0.007**

### A104. Retroperitoneal necrosis and perianal gas gangrene due to bladder rupture resulting in life threatening sepsis: a case report

#### Giacon T.A.^1^, Asti N.^1^, Meggiolaro M.^2^

##### ^1^Institute of Anaesthesia and Intensive Care Unit, Padua University Hospital, via V. Gallucci 13, 35125 ~ Padova ~ Italia, ^2^Anaesthesia and Intensive Care Unit, Ss. Giovanni e Paolo Hospital, Sestiere Castello, 6777, 30122 ~ Venezia ~ Italia

###### **Correspondence:** Giacon T.A.

**Background**

We present a very rare case of retroperitoneal necrosis and perianal soft tissues gas gangrene secondary to a bladder rupture caused by a blunt trauma in a 28 years old female patient with ulcerative colitis treated with mesalazine, whose characteristics are reported in (**Table 1)**. Informed consent to publish had been obtained.

**Case report**

The patient arrived at the emergency department (ED) for an accidental coccygeal trauma occurred falling from a stair. No fractures were identified through lumbar spine RX, the diffuse lower abdominal-perianal pain she suffered was misinterpreted as colitis pain from the medical staff and the patient herself, she was therefore dismissed. She was brought back to the ED the following night in a state of hypotension, mild confusion, tachycardia, peritonismus, she reported also stipsis from five days and anuria from the day before. An abdominal CT scan revealed a diffuse retroperitoneal necrosis diffused along psoas muscle up to the limb (**Figure 1)**. Subcutaneous gas was also identified. An explorative laparotomy revealed a bladder rupture and plenty of necrotic and suppurative material which was drained from the retroperitoneum. Bladder suture and a backup cystostomy were performed. She was then admitted to the intensive care unit (ICU) in a septic shock state. Intubated and mechanically ventilated an immediate broad-spectrum antibiotic therapy was started and invasive hemodynamic monitoring through PICCO catheter was used to perform a targeted fluid resuscitation therapy. Norepinephrine infusion up to 0,6 ©/kg/min has been necessary for three days. Further enlargement and ulceration of the perianal, vulvar and limb gas gangrene required surgical toilette and drainage on day five **(Figure 2)**. A targeted antibiotic therapy was started with Colistin, Clindamycin, Meropenem, Tigecycline against Clostridium Ramosum and multidrug-resistant Acinetobacter Baumannii. Intravenous Pentaglobin (IgM- and IgA-enriched immunoglobulin) were administrated for three days as adjuvant therapy. Defecation compromised the sterility of the area so a rectal probe was inserted, but fecal control was later obtained with a surgical colostomy. She maintained high levels of inflammatory markers and leukocytes associated with high fever and altered conscience state for two weeks, then she started to recovery.

**Conclusion**

We presented a case of very rare complication of blunt trauma resulting in bladder rupture, gas gangrene and retroperitoneal necrosis, described in literature only as a complication of invasive procedures [1,2] or of ulcerative colitis [3]. Aggressive and invasive treatment in this young patient allowed us to dismiss her from the ICU after 31 days and to complete a plastic surgery reconstruction on day 60 (**Figure 3**), with no permanent organ damage. Prompt and aggressive hemodynamic interventions and targeted therapy according to actual sepsis guidelines [4] have been determinant in patient’s survival. Hyperbaric oxygen therapy as an adjuvant for the healing of soft tissues gangrene was considered [5], but transportation was too risky. Pentaglobin use in case of inadequate immune response showed good results [6,7] and could have been helpful in this severe sepsis case. Ulcerative colitis could have played a role in terms of bacterial translocation [8]. We believe that this complicated and severe case has been successfully managed in the ICU setting.

**Acknowledgements**

Great help in managing this case came from Dr. Roberto Merenda, Dr. Tommaso Prayer-Galetti and the intensive care staff, the anesthesiologists, plastic surgeons, general surgeons and urologists of Ss. Giovanni e Paolo Hospital, Venice, Italy

1. Amar AD, Ratliff RK. Gas gangrene of urinary bladder and abdominal wall following catheterization. J Urol [Internet]. 1958;80(2):130–1. Available from: http://dx.doi.org/10.1016/S0022-5347(17)66146-9

2. Alonso AH, Fernández ER, Sánchez JP, Escudero RM, Jiménez JT, García EL, Fernández CH. Giant retroperitoneal abscess caused by extraperitoneal bladder rupture after instrumental vaginal delivery. Vol. 158, European Journal of Obstetrics and Gynecology and Reproductive Biology. 2011. p. 368–9.

3. Jackisch T, Freitag M, Ludwig K. Gasbrandinfektion unter immunsuppression bei colitis ulcerosa: Ein fallbericht. In: Zentralblatt fur Chirurgie. 2006. p. 84–7.

4. Evans L, Rhodes A, Alhazzani W, Antonelli M, Coopersmith CM, French C, Machado FR, Mcintyre L, Ostermann M, Prescott HC, Schorr C, Simpson S, Wiersinga WJ, Alshamsi F, Angus DC, Arabi Y, Azevedo L, Beale R, Beilman G, Belley-Cote E, Dellinger L, Cecconi M, Centofanti J, Yataco AC, De Waele J, R. Phillip D, Screening. Surviving Sepsis Campaign: international guidelines for management of sepsis and septic shock 2021, interpretation and expectation. Vol. 49, Critical Care Medicine. 2021. 1063–1143 p.

5. Mathieu D, Marroni A, Kot J. Tenth european consensus conference on hyperbaric medicine: Recommendations for accepted and non-accepted clinical indications and practice of hyperbaric oxygen treatment. Diving Hyperb Med. 2017;47(1):24–31.

6. Cui J, Wei X, Lv H, Li Y, Li P, Chen Z, Liu G. The clinical efficacy of intravenous IgM-enriched immunoglobulin (pentaglobin) in sepsis or septic shock: a meta-analysis with trial sequential analysis. Ann Intensive Care [Internet]. 2019;9(1). Available from: https://doi.org/10.1186/s13613-019-0501-3

7. Nierhaus A, Berlot G, Kindgen-Milles D, Müller E, Girardis M. Best-practice IgM- and IgA-enriched immunoglobulin use in patients with sepsis. Ann Intensive Care [Internet]. 2020;10(1):1–19. Available from: https://doi.org/10.1186/s13613-020-00740-1

8. Jin S, Wetzel D, Schirmer M. Deciphering mechanisms and implications of bacterial translocation in human health and disease. Curr Opin Microbiol [Internet]. 2022;67:102147. Available from: https://doi.org/10.1016/j.mib.2022.102147

**Fig. 1 (abstract A104).**
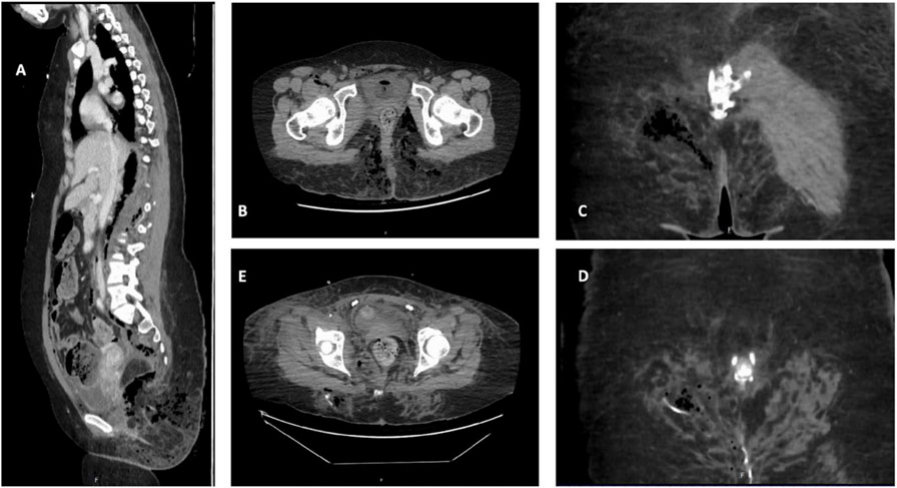
**A** AND **B**) Admission abdomen ct scan showing retroperitoneal necrosis from mediastinum to PSOAS muscle and soft tissues gas gangrene in perianal area; **C**) Gas gangrene in perianal area, mostly on the right side, before surgical toilette; **D** AND **E**) Gas gangrene reduction and necrosis increase after surgical drainage

**Fig. 2 (abstract A104).**
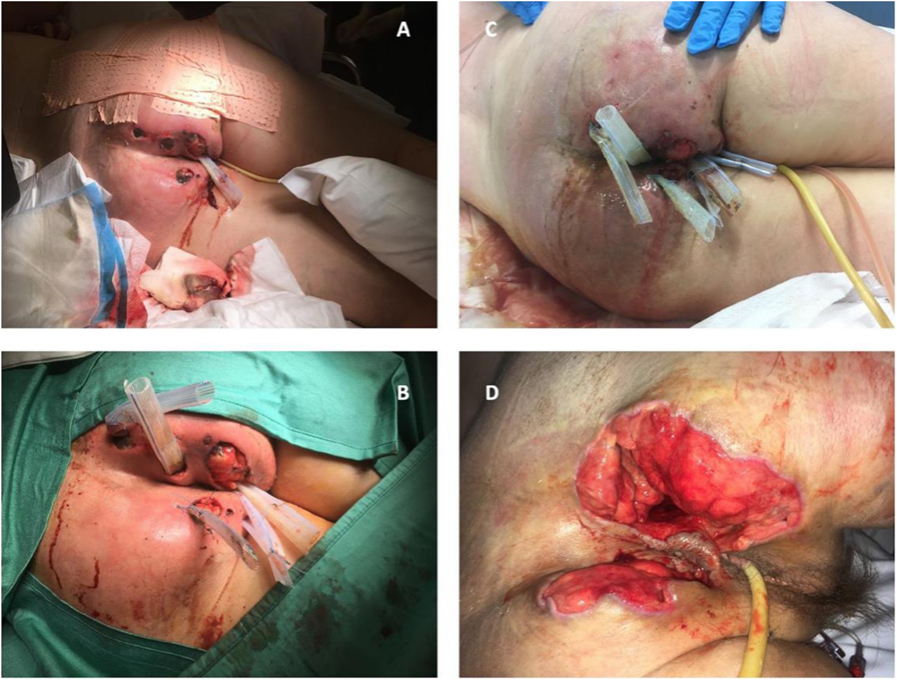
**A**) Perianal and perineal ulcerations on day fivel before surgical toilette; **B**) Operation and easy-flow drain positioning; **C**) Medication theday after surgery; **D**) 4 Days before icu dismission, no further necrosis visible, granulation tissue, and healing wound

**Fig. 3 (abstract A104).**
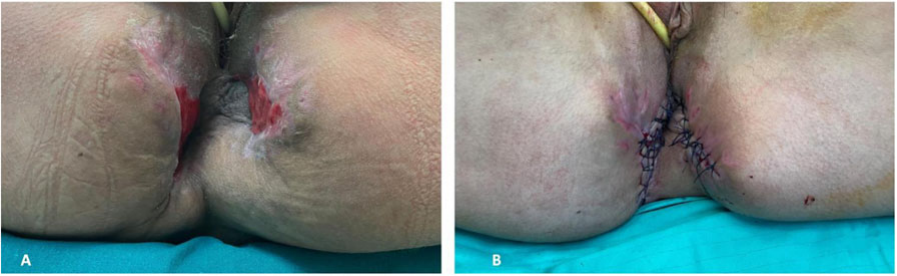
**A**) Before and **B**) After wound suture performed by plastic surgeons 60 days after the admission

**Table 1 (abstract A104).** General characteristics of the patient

Age	28
Sex	Female
Weight (admission ICU)	82 kg
Weight (dismission ICU)	70.5 kg
Height	170 cm
BMI	28.37
Comorbidities	Depression, Ulcerative Colitis, Hypotiroidism, Iron deficiency microcytic Anemia
Medicaments	Levothyroxine, Mesalazine, Delorazepam, Amitriptyline, Perphenazine

### A105. Use of an innovative cuff pressure control and subglottic secretions drainage system in COVID-19 ARDS patients undergoing pronation

#### Lombardi G.^1^, Tanzarella E.S.^1^, Baroni S.^2^, Sarlo F.^2^, Cutuli S.L.^1^, Carelli S.^1^, Cesarano M.^1^, Gennenzi V.^1^, Pintaudi G.^1^, Vargas J.^1^, Grieco D.L.^1^, Urbani A.^2^, De Pascale G.^1^, Antonelli M.^1^

##### ^1^Dipartimento di scienze dell' emergenza, anestesiologiche e della rianimazione; Policlinico Universitario A. Gemelli IRCCS ~ Roma ~ Italia, ^2^Dipartimento di scienze biotecnologiche di base cliniche intensivologiche e perioperatorie; Fondazione Policlinico Universitario A. Gemelli IRCCS ~ Roma ~ Italia

###### **Correspondence:** Lombardi G.

Introduction

Endotracheal tube (ET) cuff pressure (Pcuff) control and subglottic secretions drainage (SSD) are key factors for the prevention of microaspiration and ventilator associated pneumonia (VAP).

Objectives

An observational study to describe microaspiration in patients with Coronavirus disease 2019 (COVID-19) induced Acute Respiratory Distress Syndrome (ARDS) managed with a new Pcuff control and SSD system.

Methods

15 patients with COVID-19 induced ARDS were intubated with AnapnoGuard (AG) ETs and connected to the AG 100 control unit (Hospitech Respiration LTD). The AG 100 system provides continuous ET Pcuff regulation, detecting air leakage trough the cuff by measuring the carbon dioxide (CO2) level in the subglottic space. Additionally, it evacuates SS with dual suction lines and an extra venting line. Alpha-amylase and pepsin levels were detected from tracheal aspirates (TA) collected in the first 72 h after connection to the AG 100 system.

Results

Among 15 patients, 80% were male; median age, SAPS II and SOFA score were 65 [56-76], 35 [29-44], 4 [4-4], respectively. Main airway management and microaspiration details are described in Table1. Baseline characteristics of patients and results are shown in Table 1. Alpha-amylase and pepsin were measured in 85 (100%) and 75 (88%) TA, respectively. The median number of tracheal samplings was 6 [4;7] per patient. Pcuff values were stable between 25 and 30 mmHg with a median volume of daily SS drainage of 31 ml. Oropharyngeal microaspiration (alphaamylase value>1685 UI/l) was diagnosed in 47 TA (55%) and in nine patients (60%) abundant microaspiration was detected (>30% with alpha-amylase>1685 UI/l). Conversely no tracheal secretions samplings showed evidence of gastric microaspiration (pepsin > 200 ng/ml), not even patients with abundant events (>30% of TA with pepsin > 200 ng/ml). There was no correlation between prone positioning and median alpha-amylase levels (1973 UI/l [1159-2983] in 25 TA during pronation vs. 1899 [1092.75-3156.25 UI/L] in 60 TA in supine position; p=0.85).

The incidence of VAP was 40%, with a median mechanical ventilation time to infection of 8.5 days [7.25-9.75]. Stridor after extubation was observed in two patients. No ET misplacements occurred.

Conclusions

The use of AG 100 system has provided effective Pcuff control and SSD in COVID19 ARDS patients undergoing pronation. Oropharyngeal and gastric microaspiration rates were lower than reported with standard ETs. The application of this new technology as a tool for VAP prevention deserves further investigations.

References and Grant acknowledgments

Dewavrin F, Zerimech F, Boyer A, Maboudou P, Balduyck M, Duhamel A, Nseir S.

Accuracy of alpha amylase in diagnosing microaspiration in intubated critically-ill patients. PLoS One. 2014 Mar 6;9(6):e90851. doi: 10.1371/journal.pone.0090851.

PMID: 24603906; PMCID: PMC3946401.

Nseir S, Zerimech F, Fournier C, Lubret R, Ramon P, Durocher A, Balduyck M. Continuous control of tracheal cuff pressure and microaspiration of gastric contents in critically ill patients. Am J Respir Crit Care Med. 2011 Nov 1;184(9):1041-7. doi:

10.1164/rccm.201104-0630OC. PMID: 21836137.

Muscedere J, Rewa O, McKechnie K, Jiang X, Laporta D, Heyland DK. Subglottic secretion drainage for the prevention of ventilator-associated pneumonia: a systematic review and meta-analysis. Crit Care Med. 2011 Aug;39(8):1985-91. doi:

10.1097/CCM.0b013e318218a4d9. PMID: 21478738.

**Fig. 1 (abstract A105).**
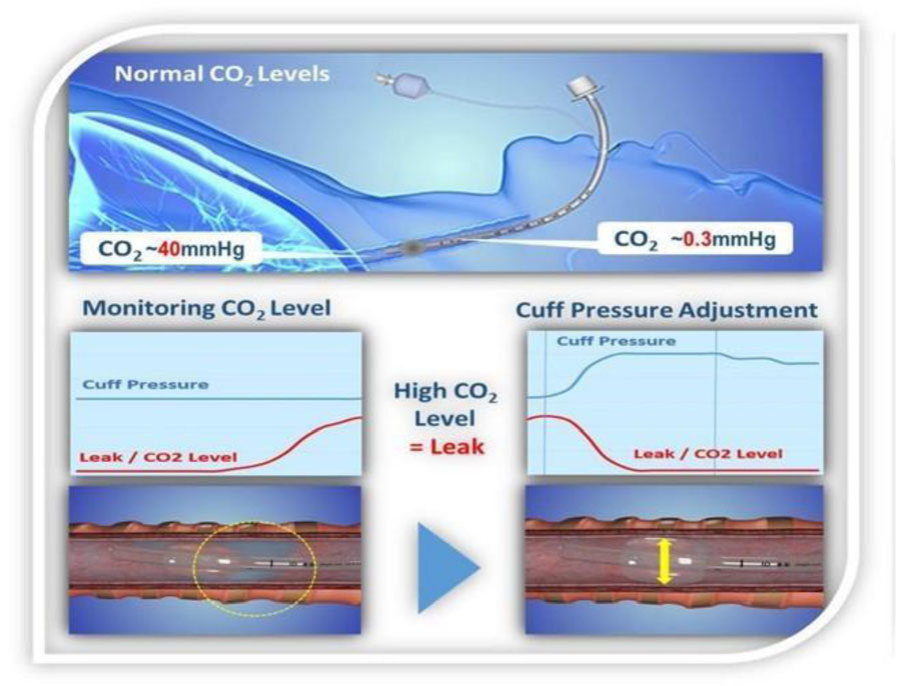
See text for description

**Fig. 2 (abstract A105).**
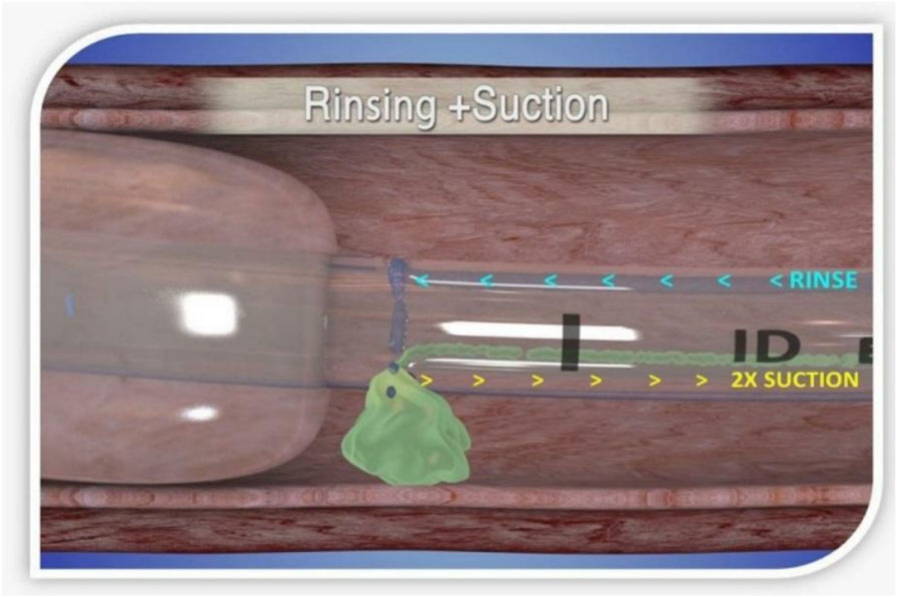
See text for description

**Table 1 (abstract A105).** See text for description

Ventilation and airway management			
*AG connection (days)*	7 [5-10]		
*Pronations per patient (n)*	4 [2-5]		
*Daily ET Pcuff (cmH2O)*	27,3 [26,8-28,1] *SS volume per patient*	170 [150-275] *SS volume per day*	31 [21,5-37]
Microaspiration and outcome			
*alpha-amylase level (UI/L)*	1905 [1132-3145]		
*Oropharyngeal microaspiration(n)*	47 (55)		
*Pepsin level (ng/mL)*	7,8 [7,8-21,45]		
*Gastric microaspiration (n)*	0 (0)		
*VAP (n)*	6 (40)		
*28-day mortality (n)*	4 (27)		

* Categorical variables are expressed in count and percentage; continuous variables are expressed in median and interquartile range

### A106. Preliminary report on early treatment with IGM-enriched immunoglobulin in COVID-19 patients

#### Pascarella S., Russo G., Golino L., Caiazzo M., Ambrogi F., Imperatore F.

##### Unit of Anesthesia and Intensive Care, San Giovanni di Dio Hospital ~ Frattamaggiore ~ Italia

###### **Correspondence:** Pascarella S.

Background

The current Covid-19 pandemic has become a global public health crisis and presents a serious challenge in treatment of severe Covid-19 pneumonia patients. IgM-Enriched Immunoglobulin is an intravenous drug, effective in the treatment of sepsis in immunocompromised patients [1,2]. Collateral effects are represented by a significant increase of pulmonary embolism [3,4]. Aim of our preliminary study was to evaluate the effectiveness of IgM-Enriched Immunoglobulin in the treatment of severe Covid-19 pneumonia patients.

Materials and Methods

We enrolled 9 patients with moderate to severe Covid-19 respiratory failure with absence of pulmonary embolism at the CT scan. IgM-Enriched Immunoglobulin treatment consisted of five days intravenous administration, titrated on body weight. Primary endpoint was to evaluate the modification of inflammation marker (PCR and D-Dimer). Secondary endpoint was to evaluate pulmonary embolism risk.

Results

We observed a significant reduction of PCR and D-Dimer in 8 of 9 patients (89%) after the end of therapy with IgM-Enriched Immunoglobulin. Mortality rate was the same reported in literature. In just one patient we observed an event of pulmonary embolism.

Conclusions

The therapy with IgM-Enriched Immunoglobulin is associated with a significant reduction of inflammation marker and reduction of pulmonary embolism risk. No mortality difference was observed.

References

1. Shankar-Hari M, Spencer J, Sewell WA, Rowan KM, Singer M. Bench-to-bedside review: Immunoglobulin therapy for sepsis - biological plausibility from a critical care perspective. Crit Care. 2012 Dec 12;16(2):206. doi: 10.1186/cc10597. PMID: 22424150; PMCID: PMC3584720.

2. Cui J, Wei X, Lv H, Li Y, Li P, Chen Z, Liu G. The clinical efficacy of intravenous IgM-enriched immunoglobulin (pentaglobin) in sepsis or septic shock: a meta-analysis with trial sequential analysis. Ann Intensive Care. 2019 Feb 6;9(1):27. doi: 10.1186/s13613-019-0501-3. PMID: 30725235; PMCID: PMC6365591.

3. Pollreisz A, Assinger A, Hacker S, Hoetzenecker K, Schmid W, Lang G, Wolfsberger M, Steinlechner B, Bielek E, Lalla E, Klepetko W, Volf I, Ankersmit HJ. Intravenous immunoglobulins induce CD32-mediated platelet aggregation in vitro. Br J Dermatol. 2008 Sep;159(3):578-84. doi: 10.1111/j.1365-2133.2008.08700.x. Epub 2008 Jun 28. PMID: 18565176

4. Pentaglobin 50 mg/ml soluzione per infusione, Foglio Illustrativo: Informazioni per l’utilizzatore. https://farmaci.agenziafarmaco.gov.it/aifa/servlet/PdfDownloadServlet?pdfFileName=footer_000752_029021_FI.pdf&retry=0&sys=m0b1l3#:~:text=Alcune%20reazioni%20avverse%20%28es.%20cefalea%2C%20arrossamento%2C%20brividi%2C%20mialgia%2C,simili%20durante%20la%20somministrazione%20di%20Pentaglobin%2Cinformi%20immediatamente%20il

### A107. COVID-19 variants in ICU: a comparison of the variant profiles delta versus omicron

#### Corriero A.^1^, Ribezzi M.^1^, Mele F.^2^, Angrisani C.^2^, Daleno A.^3^, Ferrara P.^1^, Fioriello M.^1^, Laguaragnella V.^1^, Loconsole D.^4^, Chironna M.^4^, Brienza N.^5^

##### ^1^Unit of Anesthesia and Resuscitation, University of Bari Aldo Moro ~ Bari ~ Italia, ^2^Department of Interdisciplinary Medicine - Section of Legal Medicine, Policlinico di Bari Hospital, University of Bari ~ Bari ~ Italia, ^3^Azienda Universitaria Ospedaliera Consorziale Policlinico Bari, ~ Bari ~ Italia, ^4^Department of Interdisciplinary Medicine - Hygiene Section University of Bari Aldo Moro ~ Bari ~ Italia, ^5^Department of Interdisciplinary Medicine - ICU section, University of Bari Aldo Moro ~ Bari ~ Italia

###### **Correspondence:** Corriero A.

**Background**

The Coronavirus Disease (COVID-19) is a continuing global pandemic caused by severe acute respiratory syndrome coronavirus 2 (SARS-CoV-2). Each pandemic wave was different in terms of infectivity and mortality. This study focuses on critically ill patients with the last two variants, Delta and Omicron, to see if there is a difference between the two groups.

**Materials and Methods**

ICU COVID-19 admissions were recorded daily between 1 October 2021 and 31 March 2022. Data on the patients' demographics, variants, major comorbidities, ICU parameters on admission, and outcomes were analyzed using a univariate and multivariate procedure.

**Results**

Sixty-five patients were enrolled, with 31 (47.69%) belonging to the Omicron group and 34 (52.31%) belonging to the Delta group. The Omicron group had a mortality rate of 52.94 per cent, while the Delta group had a mortality rate of 41.9 per cent. Univariate analysis (table 1) revealed that the Omicron variant was associated with the total number of comorbidities, the Charlson Comorbidity Index, pre-existing pulmonary disease, vaccination status, and acute kidney injury (AKI). The total number of comorbidities was positively associated with the omicron group in stepwise multivariate analysis (table 2), whereas pulmonary embolism was negatively associated with the omicron group.

**Conclusion**

When it comes to the patient in the ICU, Omicron appears to have lost some of the characteristics of the Delta Variant, such as endothelialitis and the more limited cellular tropism. Omicron patients have more comorbidities than delta ones which could be linked to better vaccine protection in the omicron cohort. More research into different therapeutic approaches for treating critical patients with COVID-19 is encouraged.

**Table 1 (abstract A107).** Significant variables in the univariate analysis compared data belonging to Delta and Omicron on ICU COVID admission. Values are means (±S.D.) or number (%). OR: odds ratio; CI: confidence interval.

Variables	Delta	Omicron	OR	CI 95 %	P-value
number of patients (%)	31(47.69%)	34(52.31%)			
total comorbidities, mean (±SD)	2.26(1.90)	4.03(2.83)			0.0047
CCI (points), mean(±SD)	3.16(1.97)	4.20(2.06)			0.0409
pulmonary axis, n (%)	3(9.7%)	12(35.3 %)	5.09	1.28 – 20.29	0.0313
vaccination, n (%)	13(41.94 %)	24(70.59 %)	3,32	1.19 – 9.27	0.0376
AKI, n (%)	3(9.7%)	13(38.24 %)	5.78	1.46 – 22.9	0.0172

**Table 2 (abstract A107).** Significant variables in the Stepwise logistic multivariate analysis were obtained as the main differential factors among patients belonging to Delta and Omicron groups on ICU COVID admission.

Variables	OR	95% CI	Binomial Logistic analysis P-value
Pulmonary Embolism	0.04	0.001 – 0.93	0.0453
total comorbidities	1.51	1.12 – 2.04	0.0068

### A108. Oral lesions and severity of SARS-COV2 infection in intensive care patients: is there a connection?

#### Vestito M.C.^1^, Morelli A.^1^, Pisani D.^1^, Favia G.^2^, Barile G.^2^, Tempesta A.^2^, Novielli G.^2^, Dell”Olio F.^2^, Capodiferro S.^2^, Limogelli L.^2^, Spirito F.^3^, Copelli C.^4^, Moschetta A.^5^, Corriero A.^1^, Carpagnano E.^6^, Chironna M.^5^, Loconsole D.^5^, Centrone F.^5^, Quadri M.F.A.^7^, Tartaglia G.M.^8^, Lodi G.^9^, Ribezzi M.^1^, Brienza N.^5^

##### ^1^University of Bari, Intensive Care Unit 1 ~ Bari ~ Italia, ^2^University of Bari, Department of Interdisciplinary Medicine, Complex Operating Unit of Odontostomatological Diseases ~ Bari ~ Italia, ^3^University of Foggia, Department of Clinical and Experimental Medicine ~ Foggia ~ Italia, ^4^University of Bari, Department of Interdisciplinary Medicine, Operating Unit of Maxillofacial Surgery ~ Bari ~ Italia, ^5^University of Bari, Department of Interdisciplinary Medicine ~ Bari ~ Italia, ^6^University of Bari, Department of Basic Medical Science, Neuroscience, and Sense Organs, Respiratory Diseases Section ~ Bari ~ Italia, ^7^Jazan University, Dental Public Health Department of Preventive Dental Sciences College of Dentistry ~ Jazan ~ Saudi Arabia, ^8^University of Milan, Department of Biomedical, Surgical and Dental Sciences, School of Orthodontics ~ Milano ~ Italia, ^9^University of Milan, Department of Biomedical, Surgical and Dental Sciences, School of Dentistry ~ Milano ~ Italia

###### **Correspondence:** Vestito M.C.

Background

Most SARS-CoV-2 cases are classified as asymptomatic infection or mild illness, but a considerable number of patients develop acute severe respiratory distress and, sometimes, multiple organ failure and death.The infection pathways are expressed in both the salivary glands and the oral mucosal epithelia. Few data and still no evidence are available about the correlation between patient’s comorbidities, oral lesion and COVID-19 severity. The aim of this study is to analyze the relationships between patient comorbidity and oral lesions and their predictive value for COVID-19 degree of severity.

Material and methods

The inclusion criteria were: (1)patients aged ≥18 y, hospitalized in Intensive Care Unit (ICU) or Respiratory Intensive Care Unit (RICU) from January to March 2022 (for minimizing the prevalence of different variants) for COVID-19 at Maxi-Emergencies Hospital, University Hospital Policlinic of Bari; (2) COVID-19 diagnosis based on a positive SARS-CoV-2 nasopharyngeal swab on real-time polymerase chain reaction (RT-PCR) in the presence of clinical and/or radiological signs of COVID-19.

All the patients were visited by two experienced oral pathologists. Patients’ collaborative status clinical conditions, type of ventilation and vital parameters were evaluated; extraoral and intraoral physical examination (IOE) was performed in eight steps accordingly to WHO guidelines.

The oral lesions were classified into 4 groups, following the criteria previously published by the Authors in 2021. Patients’ comorbidities were classified into different macrogroups in order to express every single disease into dichotomic variables matchable with oral lesions.

Results

103 COVID-19 patients were enrolled. The whole sample was infected by the Omicron-1 variant of the SARS-CoV-2 virus. The IOE found oral lesions in 70 patients. The majority of the lesions were treatment-related, followed by SARS-CoV-2-related manifestations. Four lesions were classified as pre-existent conditions, while the remaining 4 were poor oral hygiene related alterations. The 37.9% of the study participants had a negative outcome. Hypertension was the systemic diseases prevalent, followed by cardiac conditions. Patients developing SARS-CoV-2-related oral lesions were about 8 times more likely to have a negative outcome. Evaluating all oral lesions we noticed that the risk to have a severe course was 3 time higher than in patients who did not show any oral manifestation. Moreover, autoimmune comorbidities were found as a strong risk factor to develop severe clinical course.

Conclusion

The current study is the first to analyze the correlation between the appearance of oral manifestations and the COVID-19 patients’ outcome. The preliminary data obtained allow us to hypothesize a role of oral lesions related to SARS-CoV-2 as a prognostic factor for a possible negative outcome. Although further studies supported by histologic findings on a larger number of patients are needed, the first results may lead to consider the examination of the oral lesions as a non-invasive exam for an early prediction factor for the evolution of the disease.

**Table 1 (abstract A108).** Multiple logistic regression analyses to investigate the factors that are associated with severe COVID-19 condition or death (N = 103)

Variable	Coefficient	STD Error	R (Partial)	T	P
Oral Lesions	0,1980	0,09961	0,2261	1,987	0,0496
COVID-19 related Oral Lesions	0,3961	0,1342	0,2844	2,951	0,0040
Autoimmune Comorbidities	0,4105	0,1777	0,1959	2,310	0,0230

### A109. Predictors of bacteremia and death, including immune status, in a large single-center cohort of unvaccinated icu patients with COVID 19 pneumonia

#### Frattari A.^1^, Battilana M.^1^, Ciulli R.^1^, Visocchi L.^1^, Coladonato S.^2^, Mazzotta E.^2^, Polilli E.^2^, Parruti G.^2^, Zocaro R.^1^

##### ^1^Unit of Intensive care, Spirito Santo Hospital, ~ Pescara ~ Italia, ^2^Unit of Infectious Disease ~ Pescara ~ Italia

###### **Correspondence:** Frattari A.

Introduction

A high rate of admission to ICU, combined with the frequent use of immunomodulatory drugs, e.g. steroids and tocilizumab, put patients with COVID-19 pneumonia at high risk of developing super-infections, including ventilator-associated pneumonia, bacterial or fungal blood-stream infections (BSIs), as well as massive reactivation of herpesviridae from pulmonary microbiota, with reports of increased morbidity and mortality during ICU stay. During the SARS-CoV-2 first pandemic waves, death rates in ICU patients with COVID-19 were remarkably high, despite all efforts. Many studies have investigated risk factors for clinical progression and death and few clinical and biochemical predictors of poor prognosis are known at the moment. We investigated the possible role of immune profile at ICU admission, as well as of pulmonary or systemic viral reactivations in cases of persistent inflammation or clinical progression.

Methods

We carried out a retrospective study using linked administrative data and laboratory parameters available at the ICU wards of the regional reference hospital of Pescara, Abruzzo, Italy, March 2020-April 2021. Logistic regressions were used to identify independent predictors of bacteremia and mortality.

Results

Across the first three waves of SARS-CoV2 pandemic in our district (March-May, 2020; June-December, 2020 and January-April, 2021), 442 patients were consecutively hospitalized in the Intensive Care Units, set up within Pescara General Hospital. Patients had a mean age of 64.0 years (SD=12.2), 69.4% being males. In terms of comorbid conditions and risk factors, 91 (21.1%) were diabetic, 157 (36.4%) had hypertensive cardiopathy and 77 (17.9%) were obese. At least one episode of bacteremia was recorded for 191 patients (44.3%), with rates that were significantly different for each of the 3 waves (23%, 71% and 40%, p=0,001, respectively). At multivariate analyses, bacteremia was significantly and independently predicted by viral reactivation, pronation cycles and orotracheal intubation. Interestingly, routine monitoring of rectal and tracheal swabs for alert microorganism did not predict ensuing bacteremia. On the other hand, mortality was strongly and independently predicted by severe lymphocytopenia at ICU entrance, ensuing bacteremia and reactivation of herpes viruses. Conclusions

Our investigation, performed on a large sample of unvaccinated patients with SARS CoV2 pneumonia admitted to the ICU due to acute respiratory failure in the period march 2020 – april 2021 at a single Italian site, provides evidence that patients with SARS-CoV2 immune paralysis and hyperinflammation may suffer ensuing bacteremia and Herpesviridiae reactivation in a large proportion of cases. Most episodes of bacteremia, even due to Acinetobacter spp, were not predicted by microbiological evidence of MDR colonization, suggesting that immune paralysis by advanced SARS CoV2 infection may ease blood translocation of bacteria not detected by routine microbiological monitoring of ICU patients. Our results also suggest an unexpectedly relevant role of Herpesviridiae reactivation in this setting, heralding an unfavorable outcome, easing both bacterial translocation and end stage lung disease.

**Table 1 (abstract A109).** See text for description

Variable	Category	Bacteremia		Mortality	
		OR (95% C.I.)	p>χ^**2**^	OR (95% C.I.)	p>χ^**2**^
Age	Continuous	1.01 (0.99-1.03)	0.1769	1.07 (1.05-1.09)	**<0.001**
Gender	Female	1.00 ( - )		1.00 ( - )	
	Male	1.07 (0.67-1.71)	0.7771	1.55 (0.97-2.49)	0.0711
Bacteremia	No			1.00 ( - )	
	Yes			**2.05 (1.31-3.22)**	**<0.001**
Viral Reactivation	No	1.00 ( - )		1.00 ( - )	
	Yes	**3.28 (1.83-6.08)**	<0.001	**2.29 (1.29-4.19)**	**<0.01**
Pronation	No	1.00 ( - )			
	Yes	**3.36 (2.12-5.37)**	<0.001		
Orotracheal Intubation (IOT)	No	1.00 ( - )			
	Yes	**2.51 (1.58-4.02)**	<0.001		
Lymphocytes	≥0.6x10^3^c/μL			1.00 ( - )	
	<0.6x10^3^c/μL			**2.32 (1.49-3.64)**	**<0.001**

### A110. Synergistic effect of meropenem/vaborbactam plus fosfomycin plus colistin against extensively drug-resistant (XDR) pseudomonas aeruginosa: a case report

#### Cogi E., De Domenico C., Gnesin P.

##### ASST Franciacorta, Ospedale Mellino Mellino di Chiari ~ Chiari (Brescia) ~ Italy

###### **Correspondence:** De Domenico C.

Background

Pseudomonas aeruginosa is a highly pathogenic Gram-negative bacterium which has been recognized as one of the leading nosocomial pathogens worldwide. These infections may be difficult to treat because of the natural resistance of the species, which also has a remarkable ability to acquire further mechanisms of resistance to multiple groups of antimicrobial agents including beta-lactams, aminoglycosides and fluoroquinolones. Among these, carbapenem resistance represents a major concern worldwide. As a versatile opportunistic pathogen, P. aeruginosa has metabolic flexibility and is able to adapt to multiple conditions, including the host immune system, and to survive in medical devices. As a result, P. aeruginosa is actually among the most frequent etiologic agents causing hospital-acquired pneumonia (HAP) and ventilator-associated pneumonia (VAP). The eradication of P. aeruginosa has become increasingly difficult and a bacterial infection is associated with increased morbidity and mortality. However, the initiation of appropriate empiric antibiotic treatment in a timely manner may decrease these negative outcomes.

Case Report

Here, we report the case of a 61-year-old woman who was admitted to the Intensive Care Unit because of septic shock as a result of spontaneous atraumatic urinary bladder rupture. Her past medical history was significant for alcohol abuse, neurogenic bladder and gastrectomy. Her labs were notable for leukocytosis with neutrophilic predominance (WBC 11’980/μL, 89.5% neutrophils), increased C-reactive protein (240 mg/L), procalcitonin (>100 ng/mL) and lactate level (5.5 mmol/L). Amikacin and Piperacillin-Tazobactam were administered with a seemingly beneficial. Two weeks after the ICU admission, the patient required mechanical ventilation again. It was decided to start an antibiotic treatment with Meropenem and the chest TC showed thickening of lung tissue associated with VAP. Three weeks after the ICU admission, the antibiotic treatment was interrupted. However, the patient developed leukocytosis (WBC 8'900 -> 28'000/μL), despite her temperature remaining in the normal range with decreasing level of PCT and RCP.

She developed a ventilator-associated pneumonia due to extensively drug resistant Pseudomonas aeruginosa, which was isolated from BAL samples and oropharyngeal cultures. The bacteria was identified as a strain of a carbapenem resistant Pseudomonas aeruginosa screened as carbapenemase producer by the modified Hodge test and resistant to cephalosporins, fluroquinolones, penicillin + beta lactamase inhibitors and aminoglycosides (except for a low sensitivity to amikacin).

A triple regime of Meropenem/Vaborbactam plus Fosfomycin plus Colistin was successfully administer for 8 days to treat this pulmonary infection. In the following days, a marked improvement in the patient’s condition was observed. Therefore, it was possible to remove the patient from the ventilator and to place her in a General Medicine ward, where she continued her treatment with Colistin aerosol for other 6 days.

Informed consent to publish was obtained.

Conclusion

This case shows that, in selected cases, it might be useful to rediscover old antibiotics and to adopt them as a combination therapy. Furthermore, this case highlights the importance of clinical evidence and microbiological laboratory tests in helping clinicians to choosing the best drugs combination

### A111. Disseminated mucormycosis in intensive care unit: a case report of a challenging early diagnosis

#### Anedda F.^1^, Remiddi F.^1^, Malaspina L.^2^, Mancini V.^2^, Monciatti G.^3^, Coratti G.^4^, Franchi F.^1^, Scolletta S.^1^

##### ^[1]^Department of Medicine, Surgery and Neuroscience ~ Siena ~ Italy, ^[2]^Department of Medical Biotechnologies ~ Siena ~ Italy, ^[3]^Department of Mental Health and Sense Organs ~ Siena ~ Italy, ^[4]^Department of Emergency-Urgency and Transplantation ~ Siena ~ Italy

###### **Correspondence:** Anedda F.

INTRODUCTION: Mucormycosis is a rare fungal infection caused by ubiquitous microorganisms of the order of Mucorales. The incidence is around 1.2 cases/million population, 3.3 cases/100,000 hospitalizations. It mainly affects patients either with uncontrolled diabetes or severely immunocompromised. The most common clinical manifestation is rhino-orbito-cerebral mucormycosis (ROCM), which often presents with fever, headache, pan-sinusitis and progressive involvement of adjacent anatomical structures. Disseminated mucormycosis (DM) with encephalic and pulmonary involvement is rare (13% of cases) and generally is associated with severe immunosuppression. Diagnosis is based on imaging, mycological and histopathological examinations. Treatment option includes surgical debridement and antifungal therapy with Liposomal-Amphotericin B (L-AMB). Survival strongly depends on timeliness of care. DM generally requires admission to intensive care unit (ICU), with a mortality rate around 68%. Furthermore, in patients without risk factors and with lack of characteristic imaging and laboratory findings, an early diagnosis represents a challenge for the intensivist.

CASE-REPORT: A 71-years-old woman went to the emergency department for vomiting and headache for two days. White blood cells were 18,000/mm3, neutrophils 72%, C-reactive protein 6.96mg/L. In her medical history: monoclonal b lymphocytosis without signs of progression. A CT-scan demonstrated sphenoidal sinusitis. An empiric antimicrobial therapy was started. The magnetic resonance imaging (MRI) showed temporal pachymeningeal enhancement and pan-sinusitis. Rachycentesis was not suggestive for infectious meningitis. On the third day, L-AMB was started. On the fifth day the patient became soporous, bilateral mydriasis and visual loss appeared, alongside with mucosanguinous rhinorrhea. The patient was transferred to the ICU and was intubated (Glasgow Coma Scale 8). A new brain MRI showed cortical and intraorbital adipose tissue edema with proptosis. After founding a palatine ecchymotic area, a sphenoidectomy with debridement of sinuses was performed with collection of mucopurolent material positive for S. Epidermidis and C. Albicans. A hematologic re-evaluation was not suggestive for myeloproliferative syndrome. The second rachicentesis proved clear cerebrospinal fluid (CSF), mild protidorrachia and leukorrhea. The CSF cytology denoted positive pas-distasis amoeboid corpuscles, absence of mycetes. Therapy with miltefosine was initiated. EEG was negative. On the sixth day septic shock occurred, with need of vasopressors at maximal dosage. Day seven: fixed mydriasis, at CT-scan cerebral edema obstructing perfusion. On post-mortem histological examination, branched bastoncellular structures with hyphae and spores at the level of sella turcica and meninges were found, as well as cerebral and pulmonary mycotic emboli. Post-mortem diagnosis was “disseminated mucormycosis” (Figure 1).

Informed consent to publish had been obtained. If consent had not be obtained then the abstract should be removed from the supplement.

CONCLUSIONS: The patient presented a rare and rapidly evolving manifestation of mucormycosis with exitus after nine days from the onset of symptoms, despite an appropriate antifungal and surgical therapy. The evolution of this case was even more peculiar considering the absence of risk factors typically associated with severe clinical presentations. Moreover, the non-pathognomonic imaging and mycological findings increased the level of difficulty of an already challenging differential diagnosis. These features, combined with a rapid clinical evolution, prevented the initiation of savage-therapy with posaconazole.

**Fig. 1 (abstract A111).**
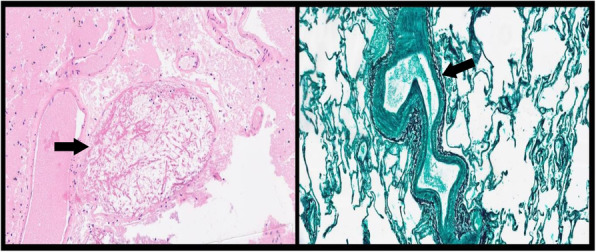
On the left cerebral mycotic embolus (Periodic acid-Schiff stain); on the right pulmonary mycotic embolus (Grocott stain). The black arrows indicate the mycotic emboli

### A112. Role of sepsis markers in the management of KPC-related septic shock: case report

#### Sicilia R., Arminio D., Chiumiento C., Manzione N., Landri P.A., Tozzi U., Pisapia A., D”Elia A., Di Lascio C.A., Buonavolontà C., Chiumiento F.

##### asl salerno PO Battipaglia ~ battipaglia ~ Italy

###### **Correspondence:** Sicilia R.

INTRODUCTION:Sepsis markers are increasingly used in intensive care as an aid to management of severe sepsis and septic shock.

CLINICAL CASE: The patient S.A. by aa. 88 comes from chronic respiratory insufficiency exacerbated by a severe bronchopneumonic process bilateral in a carrier of post-ischemic dilated myocardiopathy with F.E. 20%. In history COPD, Atrial fibrillation, episodes of Ventricular tachycardia resolved with defibrillator implantation, diabetes mellitus.Upon arrival he has a markedly soporous state,qSOFA score of 16, arterial hypotension resistant to the fluid challenge test and requiring infusion of vasopressor amines with doses of norepinephrine that exceeded even 0.5 mcg / kg / min, signs of generalized hypoperfusion with renal insufficiency acute,increased liver function values,cardiac enzymes and BNP in particular,an alteration of all markers of sepsis, including PCT 3.5ng/ml,CRP30mg/dl, Leukocytosis with G.B.61,000/mm3,95%neutrophils,PLT450,000/mm3,Fibrinogen650 mg/dl,Presepsi=2210pg/ml,BioADM>85pg/ml,Renal enkephalin>120pmol/l,dPP3>62ng/ ml,IL-1=25pg/ml,IL-6=43pg/ml,BNP=17.000pg/ml Culture tests show the presence on bronchus aspirate and BAL of Enterococcus faecalis and Klebiella Pneumoniae KPC producer of ESBL(also found on rectal swab)and several colonies of Candida Albicans and Parapsilosis which are also found on all blood cultures carried out and on the urine sample. Start treatment with Meropenem / Vaborbactam at a dosage of 2g * 3 / day in 3 hours for 14 days in monotherapy antibiotic associated with caspofungin 70 mg i.v. as a bolus followed by 50 mg i.v. * 2 for 14 days, with improvement in sepsis indices. I G.B. 56,000 carry with the passing of the days at 10,500, neutrophilia drops to 75%, PCT from 3.5ng/ml initial drops to 0.15,CRP drops to 0.3mg/dl, platelet absent,the Fibrinogen goes to 323mg/dl, he Presepsin drops to 195pg/ml,the BioADM to values <45pg /ml, the BNP goes,compatibly with his already known dilated myocardiopathy, at 732pg/ml, renal enkephalins are <80pmol/l. During hospitalization performs percutaneous tracheostomy. Chest CT scan at the end of treatment shows the disappearance of pulmonary thickening. The patient with home ventilator is sent to a suitable facility.

CONCLUSIONS: Sepsis is a life-threatening clinical syndrome of organic dysfunction caused by a dysregulated response to infection. Continuous monitoring of sepsis biomarkers that current clinical chemistry puts us in has become indispensable disposition, and whose course, in the days of the patient's hospitalization, very often correlates with the severity of the sepsis and / or the efficacy of the antibiotic treatment. Procalcitonin and presepsin compared to the others seem to play a fundamental role in guiding the clinician management of infectious diseases.

BIBLIOGRAPHY

1. Levy MM, Dellinger RP, Townsend SR, et al. The Surviving Sepsis Campaign: Intensive Care Med. 2010;36:222–31

2. Gros A, Roussel M, Sauvadet E, et al. The sensitivity of neutrophil CD64 expression as a biomarker of bacterial infection is patients. Intensive Care Med. 2012;38:445–52.

3. Andaluz-Ojeda D, Bobillo F, Iglesias V, et al. A combined score of pro- and anti-inflammatory interleukins improves mortality

prediction in severe sepsis. Cytokine. 2012;57:332–6.

### A113. The effect of the COVID-19 pandemic on healthcare-associated infections (HAIS) in acute respiratory failure ICU patients

#### Bussolati E.^1^, Quaranta A.^1^, Ragazzi R.^1^, Volta C.A.^1^, Cultrera R.^2^, Scaramuzzo G.^1^, Spadaro S.^1^

##### ^1^Intensive Care Unit, Department of Translational Medicine, University of Ferrara ~ Ferrara ~ Italy, ^2^Infectious Diseases Unit, Department of Translational Medicine, University of Ferrara ~ Ferrara ~ Italy

###### **Correspondence:** Bussolati E.

BACKGROUND: Healthcare-Associated Infections (HAIs) are defined as infections acquired at least 48 hours after hospital admission or within 3 days from discharge. Their incidence in Intensive Care Unit (ICU) is progressively rising, through years, due to aging of patients admitted to the ICU, increase of invasive devices and long lasting antibiotical therapies [1]. There is evidence of a higher incidence of HAIs in ICU COVID-19 patients, reliable to a major use of personal protection equipment, increased workload and presence of healthcare professionals deployed from other areas [2]. Nevertheless, it is still no clear the impact of the COVID-19 pandemic on HAIs in critical ill patients, independently from the SARS-CoV-2 infection. The aim of this study is therefore to compare HAIs in two samples of patients affected by acute respiratory failure requiring mechanical ventilation (MV) during the same period of 2019 and 2020.

MATERIALS AND METHODS: In this monocentric retrospective cohort study we included patients aged 18-90 years old, admitted to the ICU and requiring MV between February and April of 2019 and 2020 (respectively defined as pre-pandemic (PP) and intra-pandemic (IP)). We excluded patients with no insertion of invasive devices, no cultural sampling and with positive cultural isolations on admission. Data regarding clinical variables before ICU admission, ICU ventilation, antimicrobial therapy and cultural isolations have been collected. Qualitative variables have been expressed as number (%) while quantitative variables as mean ± SD and median [IQR] number according to data distribution, verified via Shapiro-Wilk Test. Student’s t-test, Mann-Whitney U test and Pearson’s chi-square test have been used to compare the two samples. P-values < 0.05 have been considered significative.

RESULTS: Ninety-eight patients have been included in the study sample, respectively 45/98 PP and 53/98 IP, whose demographical and clinical aspects are summarized in Table 1. Between PP and IP we found a higher number of patients [28 (62.2%) vs 39 (73.6%), p=0.23] developing HAIs.

The median number of HAIs per patient has grown in the IP group from 1 [0 - 2.5] to 2 [0 - 4.5] (p=0.027) as well as the mean number of cultural samples collected per person (from 8.24 ± 7.79 to 12.94 ± 12.97, p=0.03). Totally 65 microorganisms have been isolated in the PP group while 134 in the IP group. In Table 2 are described the proportions of every isolated microbial family. The increased isolation of Candida spp. (p<0.001) and Enterococcus Faecalis (p=0.047) during the pandemic was found to be significant.

CONCLUSION: During the COVID-19 pandemic we observed an increase in HAIs as well as an increase in the average number of cultural samples collected per patient. An increase in Candida spp. and Enterococcus Faecalis isolations during the pandemic has also been noticed.

BIBLIOGRAPHY:

1. Vincent J.L. et al. International Study of the Prevalence and Outcomes of Infection in Intensive Care Units. JAMA.2009,302,2323–2329

2. Grasselli G. et al. Hospital-Acquired Infections in Critically Ill Patients With COVID-19. Chest.2021,160,454–465

**Table 1 (abstract A113).** Baseline characteristics at ICU admission, comorbidities, entrance diagnosis and clinical features in the study population

Parameter	Pre-pandemic (PP) (***n***=45)	Intra-pandemic (IP) (***n***=53)	*p* value”
Age (years)	71.4 ± 14	68.4 ± 10.7	0.23
Males (number)	27 (60%)	32 (60.4%)	0.97
Weight (kg)	75.7 ± 20.1	80.1 ± 15.2	0.23
Height (cm)	168.6 ± 10	169.8 ± 7.5	0.51
BMI (kg/m^2^)	26.5 ± 5.5	27.8 ± 5.1	0.23
Hypertension (yes)	32 (71.1%)	36 (67.9%)	0.73
Heart Disease (yes)	25 (55.6%)	15 (28.3%)	***0.006***
Pneumopathy (yes)	13 (28.9%)	9 (17%)	0.16
CKD (yes)	13 (28.9%)	3 (5.7%)	***0.002***
DM (yes)	10 (22.2%)	14 (26.4%)	0.63
Immunosuppression (yes	7 (15.6%)	5 (9.4%)	0.36
Smoke			0.54
*Current Smokers*	8 (22.2%)	7 (13.2%)	
*Former Smokers*	9 (25%)	15 (28.3%)	
ICU entrance diagnosis			0.23
*Postoperative Monitoring*	25 (55.6%)	19 (35.8%)	
*Pneumopathy (comprehended COVID-19)*	8 (17.8%)	18 (34%)	
*Septic Shock*	4 (8.9%)	10 (18.9%)	
*Neuropathy*	4 (8.9%)	2 (3.8%)	
*Trauma*	2 (4.4%)	2 (3.8%)	
*Heart Disease*	1 (2.2%)	1 (1.9%)	
*Metabolic Disease*	1 (2.2%)	0 (0%)	
*Other*	0 (0%)	1 (1.9%)	
ICU length of Stay (days)	7.7 ± 8	11.7 ± 10.6	***0.039***
Dead during ICU (yes)	9 (20%)	11 (20.8%)	0.93
Duration of Invasive Ventilation (days)	4.3 ± 5.5	86 ± 7.9	***0.002***

Baseline characteristics, comorbidities, entrance diagnosis and clinical features in the study population, divided for year of admission. Data are expressed as Mean ± SD or number (%), according to the data

*BMI* Body mass index, *CKD* chronic kidney disease, *DM* diabetes mellitus

**Table 2 (abstract A113).** Microbial isolations in blood, respiratory tract and urinary tract samples

***Pre-pandemic (PP)***	***Intra-pandemic (IP)***		
FAMILIES	n° (%)	n° (%)	FAMILIES
*Staphylococcus spp.*	23 (35.4%)	37 (27.6%)	*Staphylococcus spp.*
*Enterobacteriaceae*	11 (16.9%)	31 (23.1%)	*Candida spp. §*
*Pseudomonas aeruginosa*	10 (15.4%)	15 (11.2%)	*Enterobacteriaceae*
Others*	6 (9.2%)	12 (8.9%)	*Enterococcus faecalis §*
*Acinetobacter baumannii*	3 (4.6%)	10 (7.5%)	Others*
*Morganella morganii*	3 (4.6%)	9 (6.7%)	*Pseudomonas aeruginosa*
*Streptococcus spp.*	2 (3.1%)	9 (6.7%)	*Klebsiella spp.*
*Stenotrophomonas maltophilia*	2 (3.1%)	7 (5.2%)	*Stenotrophomonas maltophilia*
*Candida spp.*	2 (3.1%)	2 (1.5%)	*Streptococcus spp.*
*Enterococcus faecalis*	1 (1.5%)	1 (0.7%)	*Acinetobacter baumannii*
*Klebsiella spp.*	1 (1.5%)	1 (0.7%)	*Haemophilus influenzae*
*Haemophilus influenzae*	1 (1.5%)	0 (0%)	*Morganella morganii*
Total number of isolated Microorganisms	65	134	Total number of isolated Microorganisms

*= Legionella Pneumophila, Proteus Mirabilis, Corynebacterium spp., Serratia Marcescens, Actinomyces, Lactobacillus Casei, Citrobacter Freundii, Alcaligenes Xylosoxidans, Propionibacterium Acnes

§: p < 0.05 comparing the relative percentage in the two groups

### A114. A man in a canal and his seroconversion: the story of an atypical pneumonia

#### Buffoli F.^1^, Cogi E.^2^, Gnesin P.^2^

##### ^1^Università degli Studi di Brescia ~ Brescia ~ Italy, ^2^ASST Franciacorta ~ Chiari ~ Italy

###### **Correspondence:** Buffoli F.

A 63-year-old man presents to the ER with a 7-day history of watery diarrhoea, conjunctivitis, arthralgia and fever. He claims he fell accidentally in an irrigation canal and the dynamics are suspicious for ingestion/inhalation of wastewaters. Anamnestically, he had a cholecystectomy and pulmonary TB during childhood. Since vitals are stable, he gets admitted in an ordinary ward where he starts antibiotic therapy with Piperacillin/Tazobactam and Azithromycin. After few hours, he develops a peak of fever with worsening of the respiratory dynamic and growing necessity for ventilatory support. The neurological exam is negative for meningeal symptoms; petechiae of the lower limbs are observed. Lab workup shows severe worsening of renal function with nephrotic syndrome, thrombocytopenia and increase of inflammatory index. The patient is thus intubated and admitted to the ICU, where is subjected to a bronchoscopy due to haematic secretions at tracheal aspiration. The procedure highlights a picture of haemorrhagic alveolitis. At the chest CT there is evidence of ground-glass areas and bilateral parenchymal consolidation [Figure 1: chest CT]. To identify the infection focus, complete cultural exams are performed (blood cultures, urine cultures, faecal cultures, nasal and pharyngeal swabs, BAL), Multiplex PCR of the lower and upper airways and, in sight of the anamnestic data, research for Leptospira, Brucella and Francisella Tularensis. The only positive outcomes were for Campylobacter Coli with regards to the faecal cultures and Staphylococcus Aureus at the nasal swab; no resistance to the present therapy. The serology for Leptospira was negative. In consideration of the haemorrhagic alveolitis with associated nephrotic syndrome, Hantavirus infection is suspected; therefore, new biological samples are collected and sent to a dedicated lab, where the serology for Leptospira is repeated and results borderline (1:100) for L. Icterohaemorragiae and Bataviae Pavia 1. To confirm the infection, serum, blood, BAL and urine Real Time PCR are required, but come out negative. During the hospital stay, two cycles of pronation are required due to poor respiratory exchanges; and since the need for invasive ventilation, a tracheostomic cannula is positioned. Considering the strong clinical suspicion for Leptospirosis, regardless the negative lab results and the improvement of the patient’s conditions, the steroidal and antibiotic therapy are continued. No positivity of the next cultural exams.

After 21 days, the patient is dismissed from the ICU with minimal need for O2 and adequate renal function, with no identification of the source of infection. On day 39 since the beginning of the event, serology for Leptospirosis is repeated; the seroconversion with positivity of the IgM Leptospira Icterohaemorragiae (1:400) allowed to obtain diagnostic confirmation.

Leptospirosis diagnosis happened late mainly due to two elements: on one side, the immediate initiation of adequate antibiotic therapy did not allow for identification of the microorganism in the samples and, on the other hand, the initial state of the disease did not allow for confirmation of the seroconversion.

Lastly, this patient does not stop surprising us: after around 2 months since dismissal, he develops a reactivation of the pulmonary TB, probably correlated with the steroid-induced immunosuppression.

Informed consent to publish had been obtained

**Fig. 1 (abstract A114).**
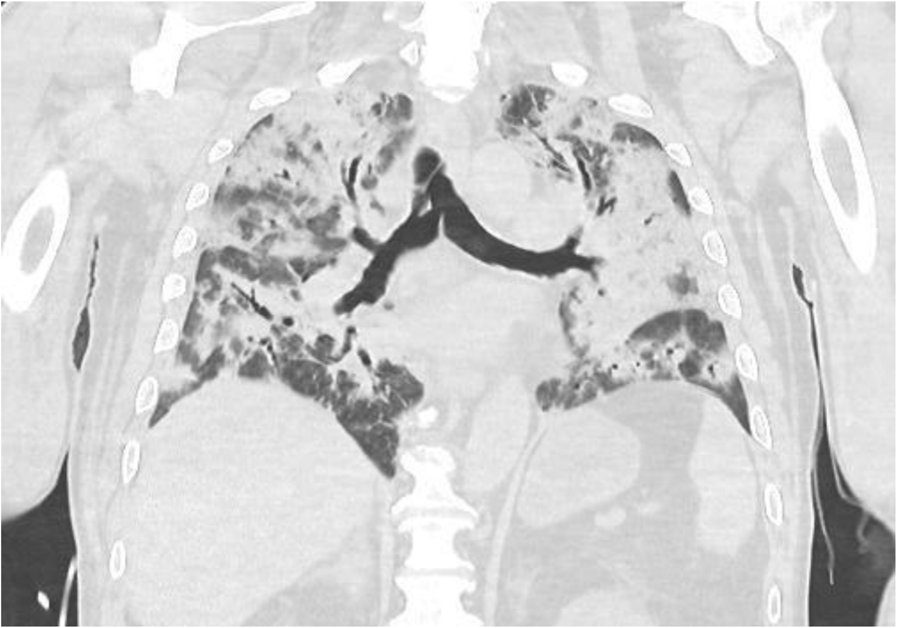
See text for description

## Maxiemergenze e ambiente ostile

### A115. From the tragedy, the first front against the emergency COVID 19: the G8 building of L’AQUILA during the pandemic

#### Petrucci E.^1^, Pizzi B.^2^, Bianchi C.^3^, Marrocco G.^3^, Ceccaroni G.^3^, Marinangeli F.^3^

##### ^1^1Department of Anesthesia and Intensive Care Unit, San Salvatore Academic Hospital of L’Aquila ~ L'Aquila ~ Italy, ^2^2Department of Anesthesia and Intensive Care Unit, SS Filippo and Nicola Academic Hospital of Avezzano ~ L'aquila ~ Italy, ^3^Department of Life, Health and Environmental Sciences ~ L'Aquila ~ Italy

###### **Correspondence:** Bianchi C.

Background

COVID-19 disease was declared a pandemic by the World Health Organization on 11 March 2009. Its rapid spread has involved entire health teams in the rehabilitation, reorganization of the Intensive Care Unit (ICU) and Intensive Care departments and Subintensiv through innovative protocols, isolation systems and unidirectional pathways to minimize intra-hospital contamination and contagion. The city of L'Aquila was hit hard by the spread of the coronavirus 19 especially in the second wave of the pandemic. The purpose of this work is to describe how the tragedy of the earthquake that struck the city of L'Aquila in 2009, provided the experience and the "means" needed to cope with the spread of the new virus.

Materials and methods

After the disastrous earthquake of April 2009, the mobile structure called Building G8, was delivered at the G8 INTERNATIONAL SUMMIT, held in July in L'Aquila. This structure represented the first embryo of the reconstruction of the San Salvatore Regional Civil Hospital, rendered unusable by the earthquake. The G8 Building housed several departments, including ICU and two operating rooms. Later, as the reconstruction of the hospital progressed, the building housed outpatient facilities. At the end of the restoration of all the sanitary areas of the San Salvatore, the modules of the G8 Building would be abandoned. In March 2020, under the emergency weight of the pandemic, the decision to discontinue each module was postponed and the building was equipped within a week to contain in principle 6 respiratory resuscitation stations and 10 subintensive therapy, during the first wave of the pandemic. In the second wave, resuscitation units were increased to a maximum of 18 while subintensivistic units were increased to 30. The peculiarity of the G8 building modules was to be external to the hospital and to have an air intake and filtration system, so as to constitute negative pressure circuits, necessary for the containment of the virus and the protection of healthcare personal. This network was implemented in response to the increase in the number of patients. In addition, each hospital room was equipped with an anteroom to ensure that staff could be decontaminated without polluting areas not used for health care. Each hospital room was equipped with vital signs and respiratory monitoring systems, and a closed-circuit camera with remote control. In addition, a CT station and two operating rooms dedicated to COVID patients were installed next to the building.

Conclusions

The structure in external and modifiable mobile modules in relation to the needs of the moment, and the remote monitoring system, represented a valid system for the protection of personal, since inside the same building the patient areas were clearly separated by double filter zones, with less stress for the workload. In conclusion, the tragedy of the earthquake 2009 provided the experience and the means to meet the new needs of the epidemiologic maxiemergency, keeping basically "clean" the other healthcare environments dedicated to non-COVID patients.

## Medicina critica e dell'emergenza intraospedaliera

### A116. Elevated methhemoglobinemia in acute food poisoning by nitrite ion- case report

#### Spinetto G., Cincinelli A., Bona M., Bergamino M., Bruzzone C., Laureri A., Presi B., Gandolfo C., Bonfiglio M.

##### Anesthesiology - Intensive Care, Local Health Agency n° 4, Lavagna, Liguria Region, Italy ~ Lavagna ~ Italia

###### **Correspondence:** Cincinelli A.

Nitrite is a preservative used in the food industry and a potentially fatal methemoglobinizing agent when taken in toxic doses. In June 2020 the Pavia poison control center recorded different emergencies on the national territory, both relating to acute food poisoning from nitrite. Case report Mrs F.A. 65 aa (in anamnesis a non-symptomatic and clinically stable pulmonary sarcoidosis) after lunch, taken in a public place, syncopated and reported a slight occipital trauma. She was recovered in the emergency room of Lavagna hospital. The patient appeared alert, cooperative, pale, with cyanotic lips and tachycardia. Hb 12.3, Hct 35.9, PLT 199.000, ions in order, troponine negative. EGA: pH 7,44, pO2 141, pCO2 45, P/F 404, Lac 1,4, Hb 11, SBE 6,4, MetHb 54. When asked about what happened, the lady reported that she had taken a portion of salad and a steak of grilled tuna. In accordance with the CAV of Pavia, antidote therapy was started: methylene blue 1 mg/Kg repeatable up to a maximum dosage of 7 mg/Kg/day. In the suspicion of food poisoning due to nitrite, plasma samples were sent to the laboratories of the CAV which confirmed the high concetration of the nitrite ion (indosable) and its catabolite, the nitrate ion (245 micromol/L). The patient was monitored in our ICU for 4 days until the haematic metahaemoglobin values normalized. During hospitalization, the patient always remained spontaneously breathing and always mantained stable vital parameters. Sodium nitrate intoxication is rare. Serious effects can occur, mainly through formation of nitrite and nitric oxide, which can cause methaemoglobinaemia and vasodilatation. Even if the presenting symptoms are mild, it is important to remain cautious since more serious symptoms can occur later. Monitoring of respiratory and haemodynamic status and repeated blood gas analysis in order to detect methahaemoglobinemia are recommended.

Informed consent to publish had been obtained.

**Fig. 1 (abstract A116).**
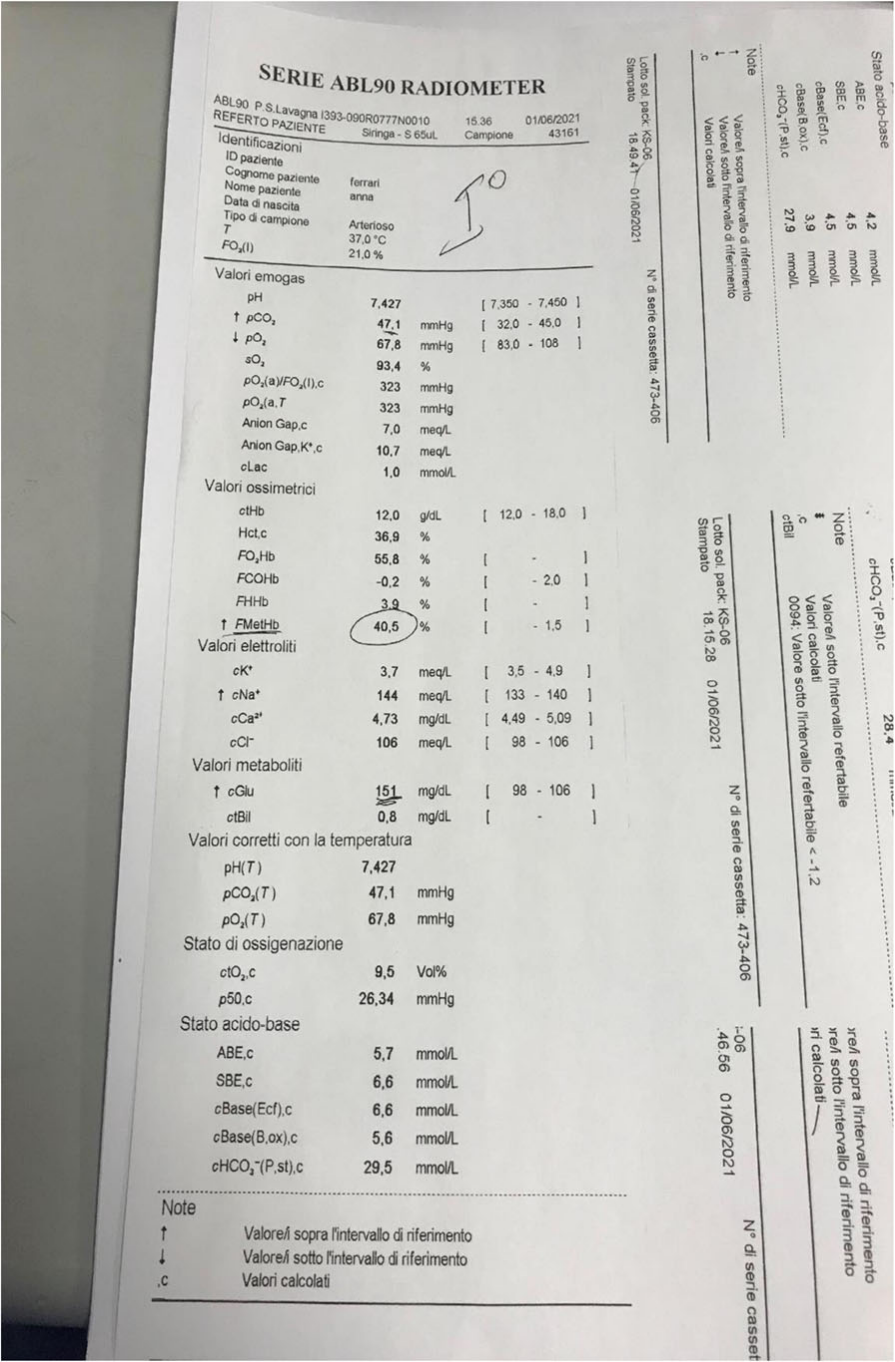
See text for description

### A117. Dramatic clinical worsening in COVID-19 patients before entering public health system. Comparison of vaccinated and not vaccinated patients

#### Tantillo S.^1^, Cilloni N.^1^, Talarico F.^1^, Guarnera M.^1^, Citino M.^1^, Catalano L.^2^, Orlandi P.E.^2^

##### ^1^Terapia Intensiva, Ospedale Maggiore di Bologna ~ Bologna ~ Italia, ^2^Radiologia, Ospedale Maggiore di Bologna ~ Bologna ~ Italia

###### **Correspondence:** Guarnera M.

Background

In these years we have improved progressively the management of Covid pneumonia. Now we know that covid infection is a systemic pathology and we can identify it as thrombotic microangiopathy, but also postmortem with autopsy which had shown cerebral, hepatic, heart and kidney damage.1 We would describe the difference among vaccinated and not vaccinated patients, during hospital and ICU (Intensive Care Unit) admission and the mortality rate and we would evaluate if a longer stay at home could provoke not only a respiratory worsening but also a systemic falling.

Materials and Methods

This is a monocentric retrospective study. We admitted critically ill patients in emergency ward then in ICU with confirmed Sars-CoV-2 pneumonia. Patients were divided in two group vaccinated (vax) and unvaccinated (no vax). We analyzed for every patient: the time between the beginning of Covid-19 symptoms and the admission in Hospital, the Charlson Comorbidity Index (CCI)2, the CT severity score (CT-SS) for the first CT during the admission in hospital, the SAPS II in ICU and D-dimer during admission in ICU. The time of study was from 1 November 2021 and 15 January 2022.

Results

54 consecutive patients were admitted in ICU during the time of study, 36 were no vax (66,6%) while 18 (33,3%) were vaccinated. Vax patients were older with 76 of median age versus 60 for no vax (p0,004) and went in hospital after 4 days of symptoms, instead no vax after 7 days (2-20) (p0,020), and they had a CCI of 5 versus 2 for no vax. Th CT-SS was 10 for Vax and 13 no vax group. Saps II in ICU was 35 for no vax and 39,5 for vax (p0.051). The D-dimer was 3,2 for no vax and 4,07 for vax (p0.6). The BUN (Blood Urea Nitrogen) was 49 for no vax and 71 for vax (p0,03), but it was 44 for no vax survived versus 67 for no vax dead (p0,003) and it was 67,5 for who suffered of pulmonary embolism versus 45,4 for who was without pulmonary embolism (p 0,028). (Figure 1) The mortality was 27,7% for vax and 38,8% for no vax patients (p0,4).

Conclusions

Patients no vax during their stay at home had a fast worsening such that when they were admitted in emergency department and in ICU they had the severity scores and mortality like vax patients although older and with higher CCI. Higher BUN was found in patients who suffered of pulmonary embolism and in patient no vax dead. It could become an early marker of systemic worsening and help clinicians to choose an intensive treatment instead to manage early only the respiratory system.

References

1. Vernuccio F, Lombardo FP, Cannella R et al. Thromboembolic complications of Covid-19: the combined effect of a pro-coagulant pattern and endothelial thrombo-inflammatory syndrome. Clin Radiol. 2020 Nov;75(11):804-810.

2. Lieveld AW, Azijili K, Teunissen BP et al. Chest CT in COVID-19 at the ED: Validation of the COVID-19 Reporting and Data System (CO-RADS) and CT Severity Score: A Prospective, Multicenter, Observational Study. Chest. 2021 Mar;159(3):1126-1135.

**Fig. 1 (abstract A117).**
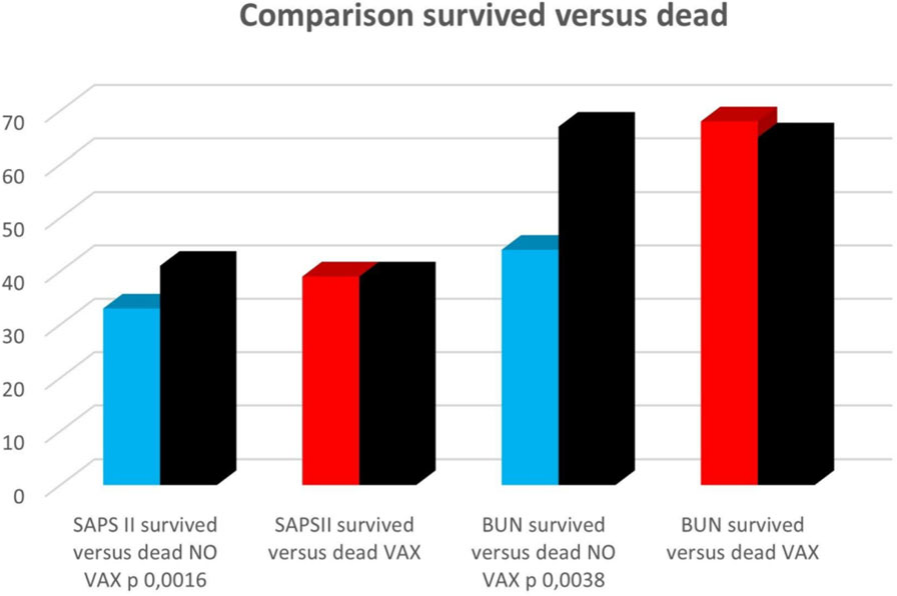
See text for description

### A118. Agreement between subcostal and transhepatic imaging of the inferior vena cava for the evaluation of fluid responsiveness: a systematic review

#### Muscarà L.^1^, La Via L.^2^, Palella S.^1^, Dezio V.^3^, Messina S.^1^, Vasile F.M.P.^2^, Cassisi C.^2^, Tringali E.^2^, Astuto M.^2^, Sanfilippo F.^2^

##### ^1^Department of Anesthesia and Intensive Care, University "Magna Graecia" ~ Catanzaro ~ Italia, ^2^Department of Anesthesia and Intensive Care, AOU "Policlinico - San Marco" ~ Catania ~ Italia, ^3^Department of Anesthesia and Intensive Care, University of Catania ~ Catania ~ Italia

###### **Correspondence:** Muscarà L.

**Background:** Assessment of fluid-responsiveness is a key aspect of daily management in critically ill patients. Non-invasive evaluation of the variation of inferior vena cava (IVC) diameter during ventilation may provide useful information. However, sagittal IVC visualization from the subcostal (SC) region is not always feasible. Alternatively, IVC can be visualized with coronal trans-hepatic (TH) approach.

**Methods**: We performed a systematic search to explore the interchangeability of IVC evaluation with SC and TH views. We searched Medline and EMBASE up to December 8^th^, 2021 to identify prospective studies on the topic. We focused mainly on feasibility, correlation, intra/inter-rater reliability.

**Results**: We included seven studies (population range: 14-131). Four studies were conducted on spontaneously breathing patients/volunteers, two on fully mechanically ventilated patients, and one in a mixed population. We found large heterogeneity regarding the analyses reported. Feasibility of TH imaging was reported between 81% and 100% (four studies). Limits of agreement between SC and TH were large (three studies). Concordance of the IVC collapsibility/distensibility indices are not interchangeable between SC and TH view (four studies). Correlation between diameters measured with SC and TH approach and intra/inter-observer correlation produced variable results (four studies). Two studies described the change of the IVC axes during ventilation, and both reported that most patients had a horizontal elliptical IVC shape.

**Conclusions:** Despite paucity of data and large heterogeneity, an overview of the included studies suggests that TH and SC assessment of IVC size and respiratory variation are not interchangeable. New studies are needed to define the proper cut-offs for fluid responsiveness using TH approach.

**Table 1 (abstract A18).** See text for description

First author, Year Journal	Subjects Position and IVC measurement site Center(s), n of views (operators and experience) Feasibility	Bias; [LoA] for IVC Diameters and variability	Correlation coefficient SC/TH Intra and inter-observer correlation
**Kulkarni AP, 2015 Ind J CCM**	88 ICU patients in shock (all in MV) Supine; IVC measured at the entry of hepatic vein Single center, 175 views; 2 operators with prior IVC workshops, 2-year experience in bedside ultrasound. Feasibility N/R (included only if both views available)	Min: 1.7 mm; [-3.7 to 4.0] Max: 0.27 mm; [-3.6 to 4.0] Distensibility 0.5%; [-16% to 17%]	Authors averaged the values obtained by two operators but did not report correlation.
**Valette X,** **2020** **Echocardiography**	131 ICU patients: 88 (67%) on MV (32 on pressure support, 56 in controlled mode), 5 (4%) on NIV] Semi-recumbent; IVC measured 3 cm from the ICAJ Two centers, 131 views; 2 operators advanced CCE training Feasibility 2D: 94% in SC, 93% in TH. Feasibility M-Mode: 83% in SC, 81% in TH. No views at all in 0.015% (n=2)*	End Insp -0.1 mm; [-8.7 to 8.5] End Exp 0.1 mm; [-7.5 to 7.7] Resp. change 0.2 mm; [-5.3 to 5.8] Collapsibility 2% [-39% to 42%] Distensibility -0.5% [-21.5% to 20.6%]	Pearson correlation between SC and TH -2D: End Exp 0.73; End Insp 0.84; Resp. change 0.86 - M-Mode: End Exp: 0.72; End Insp: 0.81; Resp. change 0.86
**Moreno Garijo J, 2017** **J Cardiothor Vasc Anaesth**	40 CABG patients (all self-breathing) Supine; IVC measured 3 cm from the ICAJ Single center, 80 views; single operator with level II training in trans-thoracic echocardiography and advanced perioperative trans-esophageal echocardiography. Feasibility: N/R	Collapsibility 2.4% [-27.6% to 34.5%]	Pearson correlation between SC and TH Max 0.46; Min 0.55; Collapsibility 0.70 ICC intra-observer -SC 0.98 Max, 0.98 Min, 0.95 Collapsibility -TH 0.99 Max, 0.99 Min, 0.97 Collapsibility ICC inter-observer** -SC 0.95 Max, 0.98 Min, 0.96 Collapsibility -TH 0.97 Max, 0.99 Min, 0.93 Collapsibility
**Saul T,** **2012** **J Emerg Med**	14 Volunteers (self-breathing) Supine; IVC measured 2 cm from the ICAJ Single center, 141 SC sagittal, 138 TH, 138 SC axial; 3 operators, IVC workshop, ultrasound fellowship completed. Feasibility 100%	*Mean measurements are mostly overlapping with differences below 1 mm.*	Inter-observer Person correlation** -SC sagittal 0.68 (average insp-exp) -TH coronal 0.69 (average insp-exp) - SC axial 0.72 (average insp-exp)
**Shah R,** **2018** **Chest**	110 patients (wards/ICU, ED) (self-breathing) Supine; IVC measured 2-4 cm from the ICAJ Single center, 284 views; 3 operators with usual ultrasound training. Feasibility: SC 90%; TH 88%; SC axial 80%. No views at all in 8.1% (n=9)	*Overall mean measurements shows IVC size significantly higher in TH view. Similar results in the subgroup analysis according to FR.*	*Concordance on FR between SC and TH in 66% (sensitivity 62%, specificity 67%). Collapsibility ≥42% in TH showed good predictive value for FR (92%), but values <42% have low predictive value for being non-FR (23%).*
**Finnerty NM,** **2017** **Western J Emerg Med**	39 Volunteers (self-breathing) Supine; IVC measured 2-3 cm from the ICAJ Single center, 351 views (117x3: SC sagittal, TH, SC axial); 3 operators experienced in IVC ultrasound (>150 scans), holding ultrasound certificates. Feasibility N/R	*Mean measurements are mostly overlapping with differences <1 mm for IVC max and <2 mm for IVC min*	ICC Inter-observer -SC 2D: 0.86; M-Mode: 0.78 Max, 0.57 Min, 0.14 Collapsibility -TH 2D: 0.74, M-Mode: 0.68 Max, 0.66 Min, 0.32 Collapsibility
**Yao B,** **2019** **Shock**	67 ICU patients (all MV) Not specified; IVC measured 2-3 cm from the ICAJ Single center, 201 views (SC sagittal, TH, SC axial); number of operators and their training not specified Feasibility: SC 84%, TH 100%	-	*Similar Area under Curve for predicting FR between SC (0.70) and TH (0.69). Higher Area under Curve for IVC diameter ratio (SC axial), 0.83*

### A119. An inconvenient neighbor: a case of acute respiratory failure in the emergency department

#### Caramia R.^1^, Guarino C.^1^, Bonghi I.^2^, Gallicchio A.^1^, Carmela L.^1^, Arces M.^1^, Francavilla S.^1^, Anglani A.^3^, De Matteis A.^4^, Fedele P.^1^

##### ^1^Anesthesia, Resuscitation and Pain Therapy Unit, D. Camberlingo Hospital, ASL BR ~ Francavilla Fontana ~ Italia, ^2^Emergency department, D. Camberlingo Hospital, ASL BR ~ Francavilla Fontana ~ Italia, ^3^Radiology Unit, D. Camberlingo Hospital, ASL BR, ~ Francavilla Fontana ~ Italia, ^4^General Surgery Unit, D. Camberlingo Hospital, ASL BR ~ Francavilla Fontana ~ Italia

###### **Correspondence:** Caramia R.

Bachground

Acute respiratory failure is a rather frequent clinical condition in the emergency department. The causes can be pulmonary or extrapulmonary.

The aim of present paper is to describe the clinical case of a patient who arrived in the emergency department with a respiratory failure due to tracheal compression.

Case report

A 93 year-old woman arrived in the emergency department in coma and respiratory distress. In the medical history he presented obesity, chronic obstructive bronchitis, anxious-depressive syndrome, hypertensive heart disease and diabetes. Family members report that for some time the patient often had an irritating cough and difficulty in swallowing during meals, with episodes like food suffocation, which however resolved in a few minutes. The last episode, which occurred about four hours earlier, never resolved and it was necessary to alert the emergency system.

On physical examination, the patient presented herself in an awakened sleep state, apparently aphasic and without significant motor deficits. There was evident respiratory distress and on chest auscultation there were noises of bronchostenosis.

An arterial blood gas test was characterized by hypoxemic-hypercapnic acidosis. The saturation was correctable with supplemental O2. Bronchodilator therapy was performed, and a brain and chest CT scan was ordered. The result showed the absence of acute events in the brain, the lack of confirmation of the suspicion of aspiration pneumonia but revealed a significant dilation of the esophagus, full of ingests, with important compressive effects on the initial part of the trachea (Figure 1).

A more careful evaluation of the images also by the consultant surgeon led to the diagnosis of the presence of a Zenker's diverticulum (Figure 2).

The patient was therefore classified as acute respiratory failure caused by compressive effects on the trachea. Flumazenil 0.25 mg was administered with improvement of the sleep state (the patient was taking lorazepam chronically), a nasogastric tube was placed with the intent to detain the esophagus, she was placed in a semi-sitting position and levosulpiride 25 mg intravenously was administered.

About two hours after arriving in the emergency room, the patient was more awake and reactive with improvement in respiratory distress and saturation and reduction of bronchostenosis, subsequently improved further until the acute respiratory failure resolved.

Informed consent to publish had been obtained

Conclusion

In this clinical case, respiratory failure was determined by an inconvenient neighbor of the trachea, Zenker's diverticulum of the esophagus, with an initial suspicion of aspiration pneumonia which was unfounded.

**Fig. 1 (abstract A119).**
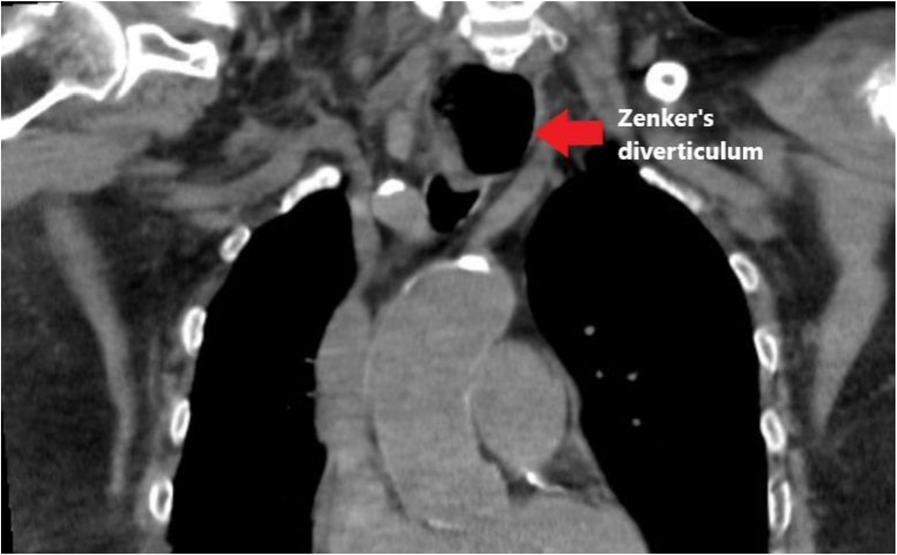
See text for description

### A120. Incidence, risk factors and outcome of COVID19 patients affected by pneumothorax or pneumomediastinum: what do we know so far

#### Travaglia C.^1^, Farronato A.^2^, Romagnoli S.^1^, Peris A.^1^, Voltolini L.^2^, Gonfiotti A.^2^

##### ^1^Dipartimento di Scienze della Salute, Scuola di Specializzazione ARTID, Università di Firenze, Azienda Ospedaliero-Universitaria Careggi ~ Firenze ~ Italia, ^2^Dipartimento di Chirurgia Toracica ~ Firenze ~ Italia

###### **Correspondence:** Travaglia C.

Introduction:Pneumothorax and pneumomediastinum have been frequently reported in COVID19 cases thus complicating the patient's overall health care management and survival rate during the pandemic.

Methods:In this retrospective cohort study we analyzed details of medical records in COVID19 patients who have and have not developed pneumothorax and pneumomediastinum radiological signs and were admitted to two hospitals in Italy, from March 2020 to May 2021. Patients were classified as group A if developed a pneumothorax or pneumomediastinum during their hospitalization and as group B if they did not. However, patients who developed a pneumothorax or pneumomediastinum while mechanically ventilated were not included. All patients of this study had not been vaccinated.

Results:A total of 525 patients with COVID19 were assessed, of which 7 patients developed only a spontaneous pneumomediastinum, 19 patients only a spontaneous pneumothorax and 30 patients developed both. Our statistical data shows an increased mortality rate in those patients who developed a pneumothorax (p value = 0.003), in smokers (p value = 0.002) and in those who had a higher Charlson Comorbidity Index (p = 0.003). Patients who developed pneumothorax were more likely to be admitted in the ICU (p value = 0.01) and intubated (p value = 0.001). Smokers also had a higher intubation rate in our study case (p value =0.04).

Conclusion:Our findings suggest that pneumothorax and pneumomediastinum in COVID19 cases were associated with increasead need of intubation and mechanical ventilation and death. Moreover, smokers and high Charlson Comorbidity Index seem to increase the mortality rate in COVID19.

### A121. Diastolic dysfunction and mortality in COVID-19 patients admitted to ICU: a single-center study

#### Amelio P.^1^, La Via L.^2^, Dezio V.^3^, Vasile F.^2^, Messina S.^1^, Perna F.^2^, Agnello M.T.^2^, Fallico G.^2^, Astuto M.^2^, Sanfilippo F.^2^

##### ^1^Department of Anesthesia and Intensive Care, University "Magna Graecia" ~ Catanzaro ~ Italia, ^2^Department of Anesthesia and Intensive Care, AOU "Policlinico - San Marco" ~ Catania ~ Italia, ^3^Department of Anesthesia and Intensive Care, University of Catania ~ Catania ~ Italia

###### **Correspondence:** Amelio P.

Introduction: SARS-CoV-2 is responsible of the Coronavirus disease 19 (COVID-19) and triggers a multi-systemic infection involving different organs. The lungs are the most affected, but a significant cardiovascular involvement has been repeatedly demonstrated. Left ventricular diastolic dysfunction (LVDD) is associated with mortality and weaning failure in intensive care unit (ICU) patients. Methods: We participated to the international COVID-ECHO study (collaboration between experts in critical care echocardiography) which aimed at characterizing cardiovascular dysfunction by means of advanced echocardiography in COVID-19 patients admitted to ICU. Hereby we present single center data on LVDD assessment (as per latest guidelines), and its association with patient's outcome. Results: Between 06.10.2020 and 18.02.2021, advanced echocardiography was performed in 35 patients, full data on LVDD were available for 26 patients (74%) but 3 were excluded as they had severely depressed ejection fraction (<35%). Of the remaining 23 patients (median age 66 years, BMI 29, 70% males; hypertension 61%, chronic obstructive pulmonary disease 22%, smoking history 22%, chronic kidney disease 17%, diabetes 4%), 16 were mechanically ventilated (70%) and 8 had diagnosis of LVDD (35%). Nine patients survived (39%) and we found no differences in ICU mortality regarding LVDD nor in the single parameters used to diagnose and grade LVDD. However, non-survivors had a trend towards greater incidence of LVDD (50%, vs survivors 11%; p=0.06) and higher E/e' ratio (11.4±3.1, vs survivors 9.3±2.4; p=0.11). Conclusions: In this single center sub-study on COVID-19 patients, assessment of LVDD according to latest guidelines was feasible in two-thirds of the overall cohort. Our results suggest that ICU mortality could be possibly associated with LVDD and higher values E/e' values. The small sample size of patients recruited warrants larger investigations.

**Table 1 (abstract A121).** Evaluation of Left Ventricular (LV) diastolic dysfunction in patients with coronavirus disease admitted to intensive care and receiving advanced echocardiography. A-L: area-length method. MODs: Method of disks

	Overall n=23	Survivors n=9	Non-survivors n=14	p-value
**Tricuspid regurgitation jet (m/sec)**	1.7 ± 0.9	1.5 ± 0.9	1.9 ± 0.9	0.47
**E wave (cm/sec)**	56.3 ± 18.8	64 ± 14.2	69.8 ± 21.4	0.48
**E/e′ ratio**	10.6 ± 2.9	9.3 ± 2.3	11.4 ± 3.1	0.11
**e′ wave (cm/sec)**	7.0 ± 2.3	7.2 ± 2.2	6.8 ± 2.4	0.67
**E/A ratio**	0.92 ± 0.33	0.94 ± 0.33	0.91 ± 0.35	0.87
**Left Atrial volume A-L (ml/m**^**2**^**)**	77 ± 45	70.4 ± 32.4	81.3 ± 52.9	0.48
**Left Atrial volume MODs (ml/m**^**2**^**)**	68 ± 40.4	62.6 ± 29.4	71.5 ± 46.9	0.52
**LV Diastolic Dysfunction**	34.7%	11.1%	50%	0.06
**grade I**	26%	35.7%	11.1%	
**grade II**	8.6%	0%	14.2%	
**grade III**	0%	0%	0%	
**indeterminate**	13%	22.2%	0%	

### A122. Subcostal vs transhepatic approach for inferior vena cava evaluation in healthy volunteers: agreement of m-mode and artificial intelligence

#### Tornitore F.^1^, La Via L.^2^, Noto A.^3^, Valenti R.^2^, Cutuli C.^2^, Dezio V.^4^, Muscarà L.^1^, Messina S.^1^, Astuto M.^2^, Sanfilippo F.^2^

##### ^1^Università Magna Graecia di Catanzaro ~ Catanzaro ~ Italia, ^2^AOU 'Policlinico- San marco' Catania ~ Catania ~ Italia, ^3^Università degli studi di Messina ~ Messina ~ Italia, ^4^Università degli studi di catania ~ catania ~ Italia

###### **Correspondence:** Tornitore F.

**Background**

Changes in inferior vena cava (IVC) diameters according to the respiratory cycle are clinically used to estimate the value of central venous pressure and to anticipate the probability of fluid-responsiveness in critically ill patients. In spontaneously breathing patients, an IVC collapsibility index (CI) higher than 40%-48% (according to various studies) has been used as cut-off for fluid responsiveness. Imaging of the IVC is usually performed via sub-costal (SC) approach with a sagittal view of the vessel. However, the SC imaging may not be feasible under several conditions (laparotomy, mediastinal drains, obesity); in such cases, the coronal trans-hepatic (TH) visualization of the IVC may represent an alternative for the evaluation of fluid-responsiveness. Whether this ultrasound approach for IVC visualization is interchangeable remains debated. Moreover, artificial intelligence (AI) software have been implemented in modern ultrasound machines to perform automated real-time calculation, and the use of this software may be clinically helpful.

**Materials and Methods**

We performed a prospective study aimed at comparing the IVC size and its variation according to the respiratory cycle in the two anatomical sites (SC and TH). We compared results obtained with both standard M-mode calculation and AI assessment in a population of 60 healthy young adults, after obtaining their informed consent. Imaging was obtained by a single experienced operator with *GE Venue Go R2* ultrasound machine model. IVC diameters (min and max) were measured manually by the operator and automatically by the AI. Collapsibility index was measured as (Diameter Max-Min)/Diameter Max. Vital parameters and anthropometric data were also recorded. The Bland-Altman analysis was performed to evaluate the mean bias between measurements and to calculate the limits of agreement (LoA) with confidence interval at 95%.

**Results**

Of the 60 volunteers, two patients did not have both SC and TH windows (3.3%), and for further three (5%) it was not possible to obtain the TH visualization. The mean bias with 95% LoA are shown in table 1 and divided according to the method of calculation.

**Conclusions**

Comparing two different sites of measurement of the IVC, we found clinically significant differences between the estimation of the vessel size and variation over the respiratory cycle in spontaneously breathing volunteers. These differences were apparent with both standard M-mode calculation and with assessment performed with the aid of AI. Although the mean bias seemed reduced by the use of the AI, the LoA remained very large. As per previous smaller studies, our study does not support the interchangeability of SC and TH approach for IVC visualization.

**Table 1 (abstract A122).** See text for description

COMPARISON		Measure	VARIABLE	Bias	LoA (lower and upper 95%)	
M-mode SC	M-mode TH	Single	Collapsibility index	13.9%	-18.1	45.8
			IVC Max diameter	-1.7 mm	-9.6	6.1
			IVC Min diameter	-4.4 mm	-14.5	5.6
AI SC	AI TH	Repeated measures	Collapsibility index	6.9%	-22.8	36.7
			IVC Max diameter	-2.0 mm	-9.3	5.4
			IVC Min diameter	-2.3 mm	-13.3	8.7

### A123. Abdominal wall muscles bleeding following prone positioning in a patient with severe COVID-19 pneumonia: a case report

#### Visciola G., Saracco E., Virno S., Messina G., Viola G., De Rosa R.C.

##### AORN dei Colli, Ospedale Cotugno -U.O.C. Anestesia, Rianimazione e Terapia intensiva ~ Napoli ~ Italia

###### **Correspondence:** Visciola G.

INTRODUCTION

The incidence of venous thromboembolism and other thrombotic events has been widely characterized and described in critically ill patients with confirmed COVID-19 infection. Cytokine storm, endotherlial injury and misregulation, hypercoagualbility have been advocated as the main phatophisiolologic mechanisms underlying the aforementioned thrombotic disorders.

In this cohort of patients the gastrointestinal tract seems to be the most common bleeding site. The hemorragic risk related to prone positioning, however, has not been estimated yet. To date only one paper reports an incidence of 25.4% of hemorragic events of upper airways bleeding and vascular access exit-site. (PMC8166520).

AIM

Unusual bleeding sites has yet to be reported. In this paper we aim to present the case of a 55-year old woman admitted to ICU with severe COVID-19 pneumonia, on low weight molecular heparin (LWMH) and aspirin therapy who developed a major bleeding form the adbominal wall muscles complicated by disseminated intravascular coagulation.

CASE PRESENTATION

A 55-year-old, 65 Kg, patient with no relevant past medical history was admitted to our ICU with severe respiratory failure requiring orotracheal intubation. mechanical and prone position ventilation.

After two cycles of prone positioning PaO2/FiO2 improved >250mmHg and the patient was successfully weaned and extubated after two days. During the weaning the patient began to develop anemia with a value of hemoglobin of 8 gr/dL, most common bleeding sites were ruled out and because of the hemodynamic stability the anemia was supposed to be secondary to the hyperinflammatory status. The day after the patient complained severe abdominal pain, hemoblogin value dropped to 7 gr/dL. A total body CT scan was ordered and a large active hematoma in the left rectus abdominis muscle was revealed, without evidences of hemoperitoneum. Additionally, cervical, mediastinal and inguinofemoral bleeding sites were discovered. The DIC was treated according to our institution protocol, and was most likely triggered by the extensive laceration of the rectus abdominis muscle secondary to pronation manouvers. Despite the improvement in coagulative profile the ventilatoy function began to deteriorate and mechanical ventilation was established again. A follow up CT scan was performed and resolution of the hematoma withouth active bleeding was noted, along with an increase of the lung consolidations. The patient developed a non-hemorragic pancreatitis and died a week later.

Informed consent to publish had been obtained and all the relevant documents were forwarded to our ethical board committe, no further approval was required for publishing this case report.

DISCUSSION

Extensive evidences outline the coagulative profile alterations in COVID-19 patients. In our case the active hematoma could have been a consequence of repeated pronation-supination manouvers. The procedure is safe and effetcitve in this ARDS patients, however it should be performed with utmost care in curarised patients due to risk of iatrogenic traumatism and injruies. In our case the hemorragic status lead to the massive use of blood products, which worsened the inflammatory lung damage and the ventilatory fucntion.

CONCLUSION

The risk-benefit profile of prophylactic anticoagulation therapy should be individualized according to clinical findings. The risk of bleeding, even from unusual sites, must be always ruled out in case or clinically relevant and rapid anemization. Traumatic and invasive maneuvers must be carried out with extreme caution in order to avoid potential damages

**Fig. 1 (abstract A123).**
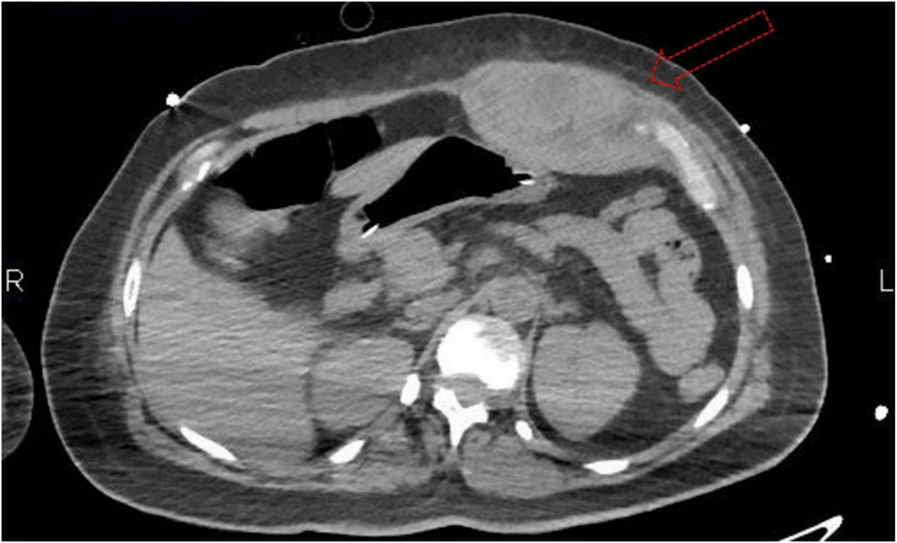
See text for description

### A124. The role of ketamine in refractory severe asthma exacerbations: systematic review of prospective studies

#### Schembari G.^1^, La Via L.^3^, Cuttone G.^2^, Morgana A.^1^, Brancati S.^4^, Cutuli C.^3^, Falcone M.^1^, Leonardi M.^3^, Astuto M.^3^, Sanfilippo F.G.^3^

##### ^1^Università "Magna Grecia", Dipartimento di Anestesiologia & Terapia Intensiva, ~ Catanzaro ~ Italia, ^2^ISMETT - Istituto Mediterraneo per i Trapianti e Terapie ad Alta Specializzazione, Dipartimento di Anestesiologia e Terapia Intensiva ~ Palermo ~ Italia, ^3^Azienda Ospedaliero Universitaria "Policlinico - San Marco", Dipartimento di Anestesiologia e Terapia Intensiva ~ Catania ~ Italia, ^4^Università degli studi di Catania, Dipartimento di Chirurgia ~ Catania ~ Italia

###### **Correspondence:** Schembari G.

**Background and Goal of Study**: Asthma is a heterogeneous disease with wide range of symptoms. Severe asthma exacerbations (SAEs) are characterized by worsening symptoms and bronchospasm requiring emergency department visits. In addition to conventional strategies for SAEs (inhaled β-agonists, anticholinergics, and systemic corticosteroids), another pharmacological option is represented by ketamine. This study was aimed at exploring the role of ketamine in refractory SAEs.

**Materials and Methods:** We performed a systematic search on PubMed and EMBASE up to August 12th, 2021. We selected prospective studies only, and outcomes of interest were: oxygenation/respiratory parameters, clinical status, need for invasive ventilation and effects on weaning**.**

**Results and Discussion:** We included a total of seven studies, five being randomized controlled trials (RCTs, population range 44-92 patients). The two small prospective studies (n=10 and n=11) did not have a control group. Four studies focused on adults, and three enrolled a pediatric population. We found large heterogeneity regarding sample size, age and gender distribution, inclusion criteria (different severity scores, if any) and ketamine dosing (bolus and/or continuous infusion). Of the five RCTs, three compared ketamine to placebo, while one used fentanyl and the other aminophylline. The outcomes evaluated by the included studies were highly variable. Despite paucity of data and large heterogeneity, an overview of the included studies suggests absence of clear benefit produced by ketamine in patients with refractory SAE, and some signals towards side effects.

**Conclusions:** Our systematic review does not support the use of ketamine in refractory SAE. A limited number of prospective studies with large heterogeneity was found. Well-designed multicenter RCT are desirable**.**

**Table 1 (abstract A124).** Summary of the included studies. RCT; Ramdomized Controlled Trial; POS: Prospective Observational Study; K:Ketamine; MV: Mechanical Ventilation; PEEPi:Positive End Expiratory Pressure Intrinsic; Cdyn: Dynamic Compliance; Rsmax: Airway Resistance; PEFR: Peak Expiratory Flow Rate; Ppeak: Pressure Peak; FEV1: Forced Expiratory Volume in 1 second; PRAM: Pediatic Respiratory Assessment Measure; PIS: Pulmonary Index Score; CAS: Clinical Asthma Score

First author, year, Design	N of patients Age (range)	Inclusion Criteria	Ketamine dose(s) Comparison Dose	Outcomes Side effects
Esmailian M, 2018, RCT	92 48 years (34-62)	-	-K: bolus 0,3 mg/kg (16,3%), 0,4 mg/kg (15,2%), and 0,5 mg/kg (17,4%) -Placebo	PEFR before and 1 h after treatment. No side effects reported.
Allen JY, 2005, RCT	68 6 years (2-10)	PIS>8	-K: bolus 0,2 mg/kg + infusion 0,5 mg/kg/h (2 h) -Placebo	PIS score at 0, 30, 60, 90, and 120 minutes. No side effects reported.
Tiwari A, 2016, RCT	48 48 months (16-144)	PRAM≥5 after 2 hour of standard therapy	-K: bolus 0,5 mg/kg (20 min) + infusion 0,6 mg/kg/h (3 h) -Aminophylline: 5 mg/kg bolus (20 min) + infusion 0,9 mg/kg/h (3 h)	∆PRAM in the first 24 h, Hypertension, Tachycardia. No side effects reported
Nedel W, 2020, RCT	45 65 (51-79)	-Adults intubated for bronchospasm -Rsmax≥12 cmH2O/L/s	-K: bolus 2 mg/kg + infusion 2 mg/kg/h -Fentanyl: bolus 1 mcg/kg + infusion of 1 mcg/kg/h	Rsmax, Cdyn, PEEPi, duration of MV at baseline, 3h and 24 h. No side effects reported.
Howton JC, 1996, RCT	44 33 (26-40)	-	-K: bolus 0,1 mg/kg + infusion 0,5 mg/kg/h -Placebo	Respiratory rate, hemodynamic parameters, Borg Score, P/F ratio, FEV1 before and after treatment. Side effects reported.
Petrillo TM, 2001, POS	10 8 (5-16)	CAS>12	-K: bolus 1 mg/kg + infusion 0,75 mg/kg/h (1 h)	CAS, vital signs, PEFR before K administration, within 10 min afetr K administration, and 1 h after infusion. Side effect reported.
Heshmati F, 2003, POS	11 30 (15-40)	-	K: bolus 1 mg/kg + infusion 1 mg/kg/h (2 h)	Ppeak, PaCO2, PaO2 before K administration, 15 min after administration and 2 h after infusion. No side effects reported.

### A125. Implementation of videocalls in caregivers of patients admitted to intensive care: a prospective evaluation of the psychological effects

#### Tornitore F.^1^, La Via L.^2^, Schembari G.^1^, Messina S.^1^, Zuccaro G.^1^, Valenti R.^2^, Tringali E.^2^, Morgana A.^1^, Astuto M.^2^, Sanfilippo F.^2^

##### ^1^UMG di Catanzaro ~ Catanzaro ~ Italia, ^2^AOU 'Policlinico- san marco' catania ~ catania ~ Italia

###### **Correspondence:** Tornitore F.

**Background**: The coronavirus pandemic has caused over 280 million infections and over 5 million deaths to date, with a high percentage of hospitalizations and intensive care unit (ICU) admissions. In the context of the contagiousness of the coronavirus disease (COVID-19), visits by relatives to their loved ones admitted to ICU for severe COVID-19 have been prohibited. Furthermore, considering the ongoing period of restrictions, visits have been also limited for ICU admitting patients negative for COVID-19. This situation has led to an inevitable detachment between patients and their families as a preventive measure to limit further spread of the pandemic. However, the resulting physical detachment could negatively impact on caregivers’ anxiety, depression, and post-traumatic stress disorder (PTSD).

In this context, video communication between patients and their loved ones could reduce the negative effects of such detachment, but the impact of this strategy on levels of anxiety, depression and PTSD disorder are not well-known.

**Methods**: In this prospective study conducted at the ICU of the Policlinico “G. Rodolico” University Hospital (Catania), we evaluated the effects of the introduction of a weekly video-call on the incidence of depression, anxiety and PTSD in the caregivers of hospitalized patients. We initially included caregivers of COVID-19 patients. For subsequent conversion of our ICU to “non-COVID”, we subsequently included caregivers of patients negative for COVID-19 for whom a direct visit to their loved one was not feasible. Assessment of anxiety, depression and PTSD was done using the following validated questionnaires (filled online): Impact of Event Scale (Revised IES-R), Center for Epidemiologic Studies Depression Scale (CES-D) and Hospital Anxiety and Depression Scale (HADS).

Caregivers who answered twice to the questionnaire were included. In particular, the first questionnaire was completed before the initial video-call, while the second was completed before the second video-call, at least one week apart.

**Results**: We included 20 caregivers (of them n=12 COVID-19 patients) from 17 patients (of them n=11 COVID-19 patients). A total of 11 patients survived (n=9 in the COVID-19 group and n=2 in the “non-COVID” group). Table 1 shows the characteristics of patients and caregivers. Regarding ventilatory support in the included patients, both conditions before and after the video-calls are shown.

The average results of the questionnaires completed by caregivers between the two video calls showed no significant difference in terms of depression (CES-D and HDAS-D), anxiety (HDAS-A) and PTSD (IES-R). These results were observed both in the entire study population (Table 2) as well as in the two subgroups analyzed according to admission diagnosis (separated into COVID-19 and “non-COVID”, Table 3).

**Conclusions**. Our preliminary results showed that a video call implementation strategy between caregivers and patients admitted to the ICU did not show an improvement in terms of the risk of depression, anxiety and PTSD. The results appear similar regardless of ICU admission diagnosed with COVID or not. Our pilot study remains exploratory and limited to a small sample. Studies of larger samples of caregivers in the context of the pandemic could b**e** helpful.

**Table 1 (abstract A125).** Demographics of patients and caregivers

Patients			
	All	COVID	Non COVID
**N**	17	11	6
**Age**	64.2 ± 17.7	66.8 ± 13.1	59.3 ± 25
**Males**	14	9 (81.8%)	5 (83.3%)
**Ventilation support at T1**	6 IMV, 9 NIV, 2 HFNC	9 NIV, 2 HFNC	6 IMV
**Ventilation support at T2**	6 IMV, 5 NIV, 6 HFNC	1 IMV, 5 NIV, 5 HFNC	5 IMV, 1 HFNC
**Increased support between T1 and T2**	1	1	0
**Decreased support between T1 and T2**	4	3	1
**Same support between T1 and T2**	12	7	5
**Survivors**	11	9	2
**CAREGIVERS**			
**N**	20	12	8
**Age**	38,2 ± 13,7	36.7 ± 13.8	40,4 ± 14,3
**Males**	9	6 (50%)	3 (37.5%)

**Table 2 (abstract A125).** Results in the overall cohort of caregivers regarding tests for Depression, Anxiety and PTSD between videocalls

TEST	T1	T2	p value
**CES-D**	19.6 ± 10	22 ± 9.6	0.17
**HADS-Anxiety**	8.7 ± 2.4	8.4 ± 3.8	0.67
**HADS-Depression**	9.5 ± 1.6	9 ± 3.9	0.59
**IES-R**	20.9 ± 10.8	23.1 ± 12	0.19

**Table 3 (abstract A125).** Subgroup analysis regarding the tests for Depression, Anxiety and PTSD between videocalls. Caregivers are separated according to the admission diagnosis (patients admitted to ICU with COVID or non-COVID diagnosis)

TEST		T1	T2	p value
**CES-D**	COVID	15.3 ± 7.8	18.3 ± 8	0.13
	NON-COVID	26 ± 9.8	27.4 ± 9.7	0.69
**HADS-Anxiety**	COVID	8.3 ± 2.2	7.2 ± 4	0.37
	NON-COVID	9.4 ± 2.7	10.1 ± 2.8	0,47
**HADS-Depression**	COVID	9 ± 0.9	7.4 ± 3.5	0.15
	NON-COVID	10.1 ± 2.2	11.4 ± 3.3	0.34
**IES-R**	COVID	17.1 ± 9.9	18.2 ± 10	0.45
	NON-COVID	26.6 ± 10	30.4 ± 11.5	0.31

### A126. Agreement of capillary refill time measurements performed at fingerand earlobe in healthy volunteers: a pilot study

#### Messina S.^2^, La Via L.^1^, Noto A.^3^, Triolo T.^2^, Continella C.^2^, Tutino S.^2^, Criscione F.^1^, Hernández Poblete G.W.^4^, Astuto M.^1^, Sanfilippo F.^1^

##### ^1^A.O.U Policlinico "G. Rodolico - San Marco" ~ Catania ~ Italia, ^2^UMG - Università degli Studi "Magna Graecia" di Catanzaro ~ Catanzaro ~ Italia, ^3^AOU G. MARTINO ~ Messina ~ Italia, ^4^Pontificia Universidad Catolica de Chile ~ Chile ~ Chile

###### **Correspondence:** Messina S.

Background

Capillary Refill Time (CRT) is a well-known parameter reflecting peripheral tissue perfusion. It is widely used as part of a simple and structured assessment of perfusion in critically ill patients.

Measurement involves the visual inspection of blood returning to distal capillaries after they have been emptied by pressure applied on the distal phalanx of a finger. However, for critically ill patients undergoing surgery the assessment of their CRT at the finger level is precluded in most cases by presence of surgical drapes. Nonetheless, CRT can be assessed in other sites (i.e. earlobe) that could be accessible during surgery. Whether the values measured at finger level and at the earlobe are interchangeable remains an open question.

Methods

We performed a prospective observational study comparing values of CRT measured in healthy adult volunteers with no pre-existing medical conditions at three different sites and/or position. A single operator performed these measurements by applying firm pressure with a glass microscope-slide in the following sites/positions: 1) ventral surface of the index finger at distal phalanx level (semi-recumbent position, finger at the height of the hip or “finger-flat”); 2) earlobe with the volunteer in semi-recumbent position with a torso elevation of 30° (“earlobe-30°”), and 3) earlobe with volunteers supine (“earlobe-0°). The pressure was increased until the skin was blank and then maintained for 15 seconds. The time for return of the normal skin color was registered with a chronometer and was video recorded for evaluation of inter- and intra-rater variability and for external validation. Vital parameters as well as non-invasive hemodynamic monitoring were performed using the Clearsight® device (Edwards lifescience, CA, USA). The non-parametric Friedman pairwise-test was used to assess between group differences; the Bland-Altman analysis was performed to evaluate the mean bias between measurements and to calculate the limits of agreement (LoA). Correlation between measurements was performed with the Spearman test.

Results

We collected data from 82 volunteers, with a median age of 30 years ([IQR] 27-35). As compared with CRT measured at finger level [median 1.04 sec (IQR 0.8-1.39)], CRT measured at “earlobe-0°” was significantly smaller [0.88 sec (IQR 0.75-1.06); p&lt;0.001], whilst CRT assessed at “earlobe-30°” was similar [1.10 sec (IQR 0.90-1.26); p=0.52].

As shown in the Bland-Altman diagram (figure 1a), when comparing the CRT at “finger-flat” position and at “earlobe-0°”, the Bias was 0.22 sec (standard deviation 0.40) and the LoA were -0.56 (lower) and 1.00 (upper). The same plot analyzing CRT at “finger-flat” position and at “earlobe-30°” showed a smaller Bias (0.02±0.18 sec) and the LoA were -0.33 and 0.37 respectively (Figure 1b).

Conclusions

In healthy volunteers, CRT measured at the earlobe with the head at 30° seems producing similar results to the assessment performed at the finger level, with good accuracy and good precision.

Conversely, the CRT measured at the earlobe with the patient in supine position (0°) produces different results as compared to the finger CRT, with lower accuracy and precision. These preliminary results do not entirely support the interchangeability of CRT assessment at different sites (finger and earlobe), especially when considering the CRT assessed at earlobe with the patient supine (0°), which would be of interest for its possible use in the operating room. Studies on critically ill patients may be useful to confirm these preliminary results.

**Fig. 1 (abstract A126).**
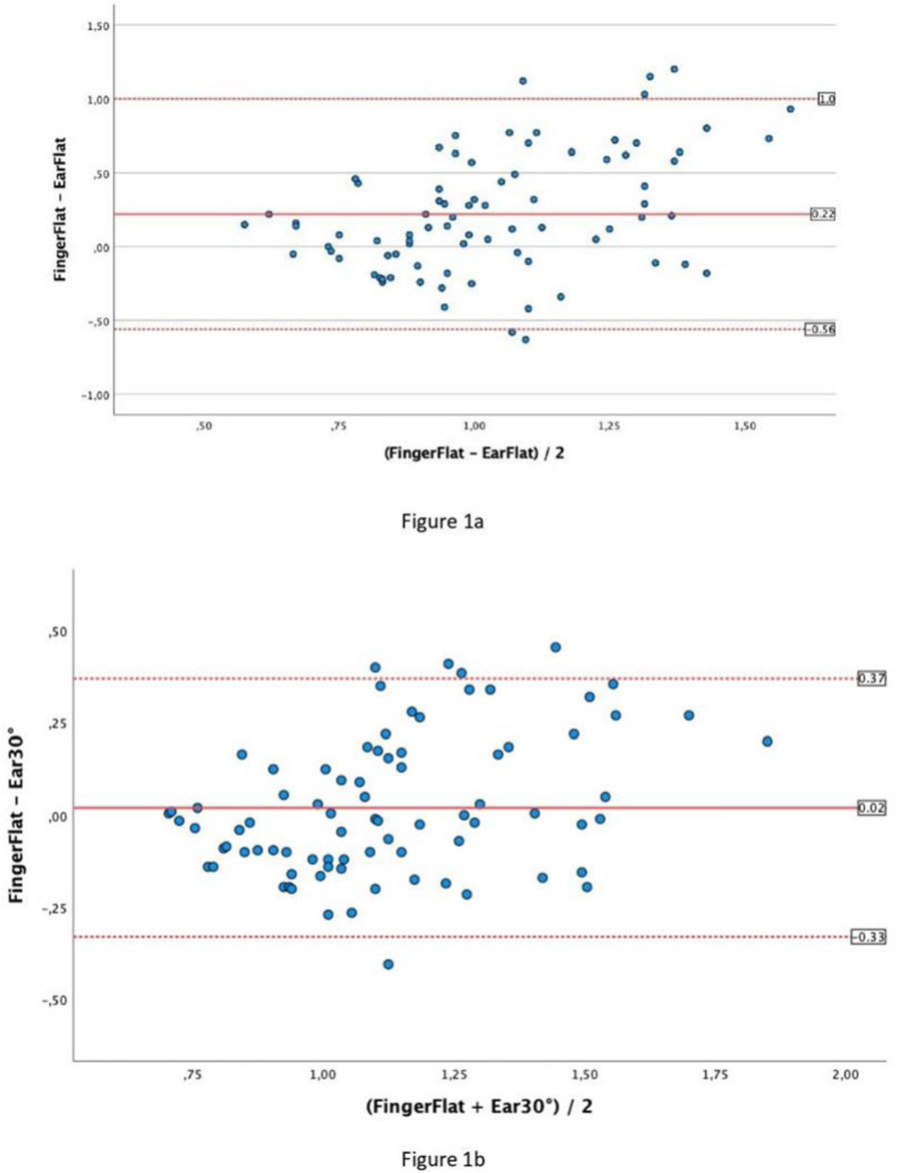
See text for description

### A127. Incidence and factors associated with acute kidney injury in COVID-19 ARDS patients undergoing prone positioning: the PRONE-AKI study

#### La Rosa R.^1^, Scaramuzzo G.^1^, Grechi B.^1^, Ragazzi R.^1^, Marangoni E.^2^, Montanari G.^2^, Alvisi V.^2^, Spadaro S.^1^, Volta C.A.^1^

##### ^1^Azienda Ospedaliero-Universitaria Sant'Anna di Cona, Dipartimento di Medicina Traslazionale e per la Romagna ~ Ferrara ~ Italia, ^2^Azienda Ospedaliero-Universitaria Sant'Anna di Cona, Dipartimento di Emergenza, Unità Operativa Di Anestesia e Rianimazione ~ Ferrara ~ Italia

###### **Correspondence:** La Rosa R.

Introduction: Prone positioning (PP) can improve gas exchange in COVID-19 ARDS patients requiring invasive mechanical ventilation (IMV) [1]. However, how PP affects abdominal organs function remains unclear [2]. Acute kidney injury (AKI) in critically-ill COVID-19 patients can affect 20–40% of patients [3], but it is still unknown if PP can be temporarily associated to its onset. Moreover, no clinical variable is validated to predict PP-associated AKI from bedside.

Objectives: To investigate the incidence of AKI associated to PP, in patients affected by COVID-19 ARDS undergoing IMV and to test if it could be predicted by any clinical variable before PP.

Methods: We analyzed COVID-19 ARDS patients undergoing IMV and admitted to the ICU of the Sant’Anna Hospital (Ferrara, Italy) between March 2020 and March 2021 who underwent at least one PP cycle. For each patient we collected demographic data (BMI, age) baseline clinical data (SAPS-II, PaO2/FiO2 at ICU admission) hemodynamics (SAP, central venous pressure (CVP), HR), cumulative fluid balances, respiratory mechanics, circulating biomarkers (Hb, Hct, creatinine, and lactates) and use of diuretic drugs in the course of PP cycle. Patients were divided into two groups based on the onset of PP-associated AKI, defined as AKI diagnosed using the KDIGO criteria [4] from PP start to 48 hours after resupination. Independent samples t-test was used to test difference between groups while binomial logistical regression was performed to estimate correlation between variables and PP-associated AKI. ROC curves were built to test the predictivity of any significant variable. A p value < 0.05 was considered statistically significant.

Results: We analyzed data from 41 patients. No patient had kidney diseases before ICU. Twenty/41 patients (48%) developed AKI according to the KDIGO criteria, and the diagnosis was made at 27.8 (±19.6) hours after starting PP. No statistically significant differences were found among the two groups in age, clinical gravity at admission, nor in serum lactates, Hgb, fluid balance and diuresis 24 hours before pronation. Nevertheless, CVP was significantly different between the two groups (p = 0.017). The multivariate logistic regression we performed was statistically significant (p < 0.05) and CVP had the best contribution in the model (p = 0.04). The ROC analysis for CVP revealed a good classifying capability, with an AUC of 0.72 (p = 0.012).

Conclusions: AKI can be temporarily associated to PP in COVID-19 ARDS patients undergoing IMV and may be related to hypovolemia. The CVP before PP can potentially predict PP-related AKI with a good accuracy.

References: (1)Scaramuzzo G., et al., Sustained oxygenation improvement after first prone positioning is associated with liberation from mechanical ventilation and mortality in critically ill COVID-19 patients: a cohort study. Annals of intensive care, 2021. (2) Hering R., et al., The Effects of Prone Positioning on Intraabdominal Pressure and Cardiovascular and Renal Function in Patients with Acute Lung Injury. Anesthesia and Analgesia, 2001. (3) Ronco C., et al., Management of acute kidney injury in patients with COVID-19. Lancet Resp Med, 2020. (4) Lameire N.H., et al., Acute kidney injury: an increasing global concern. Lancet, 2013.

**Fig. 1 (abstract A127).**
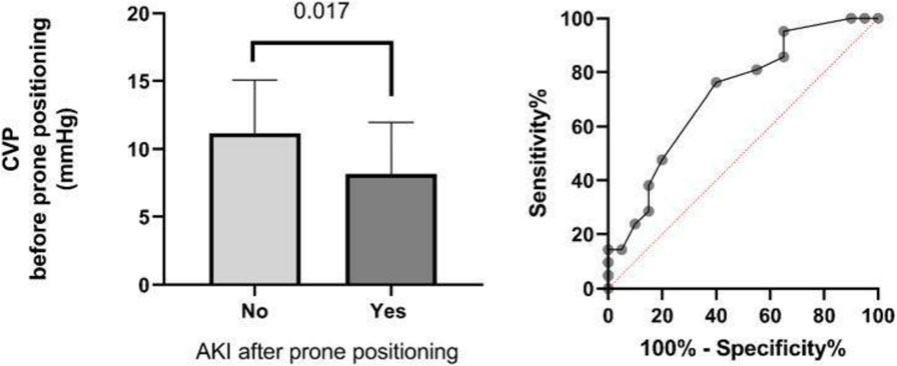
See text for description

### A128. Inhalatory sedation in COVID induced ARDS: effects on compliance

#### Cappellini I., Freschi B., Zamidei L., Campiglia L., Parise M., Consales G.

##### Azienda USL Toscana Centro ~ Prato ~ Italia

###### **Correspondence:** Cappellini I.

Background: Patients affected by severe form of COVID-19 induced respiratory failure (C-ARDS) frequently need deep sedation in order to perform adequate ventilator support. Volatile Anesthetics (Vas) are an alternative to intravenous molecules, allowing to obtain the desired level of sedation. Moreover, they seem to have anti-inflammatory and bronchodilatatory effects very useful in COVID-19 patients.

Methods: In the present paper, we show the results of a retrospective single-center nonprofit observational cohort study. We enrolled patients admitted for C-ARDS to the COVID Intensive Care Unit of Santo Stefano Hospital in Prato during the period March 2020-June 2021 who received to invasive mechanical ventilation. Participants were divided in two categories: those who received Sevoflurane and those sedated with intravenous drugs. A propensity score matching model (PSM) was applied to minimize the differences between the two groups. The Mann–Whitney/Wilcoxon test or t test was used to analyze continuous variables; for categorical variables, Fisher's exact test or chi-square test was used. Values with p < 0,05 were considered significant. Level of sedation (BIS monitoring), respiratory and ventilator parameters (PaO2/FiO2; Pulmonary Static Compliance; PEEP) and biochemical parameters were evaluated on starting of invasive ventilation (T1), 72 hours (T2) and 7 days (T3) later.

Results: a total of 112 patients were enrolled in the study. 56 patients who received inhaled sedation have been matched with 56 participants who received intravenous sedation by means of PSM. An adequate level of sedation was obtained in both groups. The application of the Wilcoxon test showed an improvement in P/F in the sevoflurane group compared to controls at T2 in respect to T1 (mean: T1 148.31 vs. 208.26 at T2), although this difference was not statistically significant (p=0.06); on the seventh day of ventilation an improvement in the P/F ratio was observed (mean: T0 148.31 vs 182.33 in the sevoflurane group at T3 (p value=0.10). Static compliance in the "Sevoflurane" population at three days was statistically significantly higher (mean of 42 compared to 39.95 cmH20/mL at T1 in the Sevoflurane group) than in the "non-Sevoflurane" population (p value = 0.02). At seven days after intubation, static compliance was improved in the "Sevoflurane" population compared to the "non-Sevoflurane" population, but the difference was not statistically significant (p value = 0.1).

Finally, a logistic regression model was applied to the paired sample, relating the mortality (dependent variable) to the treatment used (independent variable). The model showed estimate value of -0.9 with a p value = 0,02. The odds ratio is 0.40, which is a wide 95% confidence interval that does not include the value of 1 (0.18-0.87).

Conclusions: The use of sevoflurane for deep sedation of invasively ventilated CARDS patients is effective and feasible. Sevoflurane sedation induced a relevant amelioration of respiratory mechanics that may have a clinically relevant effect in C-ARDS patients. The results are encouraging regarding pulmonary compliance values three days after starting sedation therapy with sevoflurane.

Further studies will be needed to confirm the potential of this treatment in a randomized clinical trial.

**Fig. 1 (abstract A128).**
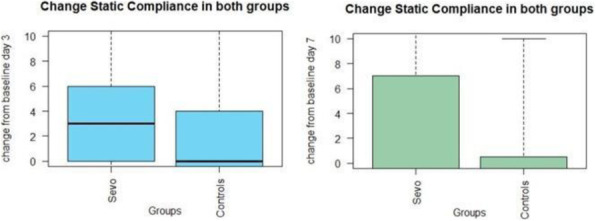
See text for description

### A129. Capillary refill time in healthy volunteers: does the arm position matter?

#### Continella C.^1^, La Via L.^2^, Noto A.^3^, Lanzafame B.^4^, Triolo T.^1^, Cirica G.^1^, Cocimano S.^2^, Hernandez G.^5^, Astuto M.^2^, Sanfilippo F.^2^

##### ^1^Department of Anesthesia and Intensive Care, University "Magna Graecia" ~ Catanzaro ~ Italia, ^2^Department of Anesthesia and Intensive Care, AOU "Policlinico - San Marco" ~ Catania ~ Italia, ^3^Department of Anesthesia and Intensive Care, AOU "Gaetano Martino" ~ Messina ~ Italia, ^4^ASP Siracusa ~ Siracusa ~ Italia, ^5^DeDepartamento de Medicina Intensiva, Facultad de Medicina, Pontificia Universidad Católica de Chile ~ Santiago ~ Chile

###### **Correspondence:** Continella C.

BACKGROUND

Capillary refill time (CRT) is a marker of peripheral perfusion and activation of the sympathetic nervous system. It has been associated with organ failure and mortality in patients admitted to intensive care unit. Its use has been implemented for the management of critically ill patients and showed promising results. The position of the arm during assessment has not been fully standardized. In particular, as compared to a flat position of the arm, its elevation may reduce venous congestion; however, it remains undefined whether this change in finger position may influence CRT measurements to a clinically significant extent.

MATERIALS AND METHODS

In healthy adult volunteers with no pre-existing medical conditions and lying in semi-recumbent position, we performed a single-center prospective study measuring CRT at the ventral surface of the index finger (distal phalanx level) on the right hand. Two measurements were taken: 1) with the index at the level of the hip (“finger-flat”), and 2) after positioning the finger in a 30° unloaded position for 60 seconds. CRT was measured by a single operator applying firm pressure with a glass microscope-slide. Pressure was increased until the skin was blank and kept for 15 seconds. The time for return of the normal skin color was registered with a chronometer. We also video recorded all measurements for subsequent intra- and inter-rater variability and external validation. All together with vital parameters (blood pressure, heart rate, oxygen saturation), we recorded hemodynamic data using Clearsight® (Edwards Lifescience, CA, USA) monitoring. Differences between the two measured CRT were assessed using the Friedman test, then a Bland-Altman analysis was performed to calculate the mean bias and the limits of agreement (LoA). Correlation between measurements was performed with the Spearman test.

RESULTS

We collected data from 82 healthy volunteers, with a median age of 30 years (interquartile range [IQR] 27-35). Median CRT for “finger-flat” group was 1.04 sec (IQR 0.8-1.39), whilst “finger-up” CRT was 1.17 sec (IQR 0.93-1.41; p=0.027). As shown in the Bland-Altman diagram (figure 1), the mean bias between measurements was -0.07 sec (standard deviation 0.3) and the LoA were -0.61 (lower) and 0.47 (upper). We found significant correlation with a Spearman rho 0.70 (95% Confidence Interval 0.56-0.80; p>0.001).

CONCLUSIONS

In healthy volunteers, the CRT measured at the finger distal phalanx in unloaded position produces different results as compared to CRT assessment with the index at the level of the hip; however, this difference is small, we found a good accuracy (small mean bias) with a clinically acceptable precision (LoA). Whether these differences are similar in critically ill patients (i.e. septic) remains to be defined.

**Fig. 1 (abstract A129).**
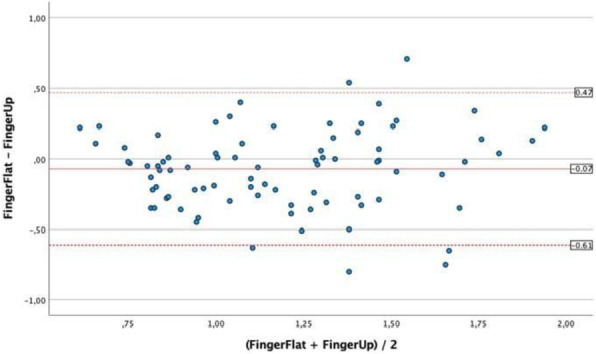
See text for description

### A130. Effects of music therapy on pain and delirium in intensive care unit patients: preliminary results

#### Scannella A.^1^, Cornacchia N.^1^, Clerico A.^1^, Laera V.G.^1^, Wambo V.^1^, Fogli A.^1^, Musolino J.^1^, Di Tizio L.^2^, Vetrugno L.^2^, Maggiore S.M.^2^

##### ^1^Università degli Studi G. d'Annunzio, Chieti ~ Chieti ~ Italia, ^2^Istituto di Anestesia e Rianimazione, Ospedale SS Annunziata, Chieti, ASL 2 Lanciano-Vasto-Chieti ~ Chieti ~ Italia

###### **Correspondence:** Scannella A.

Background:

Pain, agitation and delirium constitute the triad commonly observed in ICU patients.1,2 Several trials showed the adverse effects of prolonged deep sedation, such as development of delirium, prolonged ICU stay, prolonged time on mechanical ventilation, and increase in mortality.3,4 These symptoms are generally treated with analgesics and sedatives. Music therapy (MT) is a non-pharmacological method, complementary to conventional care, for the management of the three symptoms.5–8

Objectives:

The objective of the study was to assess the effects of MT on pain, in terms of perception and of use of analgesics, and on delirium in critically ill patients receiving analgo-sedation.

Methods:

This was a single-center study with three parallel arms, including adult patients, with RASS > -3 or GCS > 9. Exclusion criteria were: hearing loss, known cognitive disorders, acute brain injury, and high expected short-term mortality. Patients were randomly assigned to 3 groups: 1) standardized MT (music playlist created by a music therapist); 2) individualized MT (music playlist based on patient’s preferences); 3) control group without MT.

Music therapy was applied for 60-90 minutes lasting sessions once per day with Sony, MDR-ZX110, Japan headphones. The following parameters were acquired before and after each listening session, and at 24h, 3 days and 7 days after enrollment: vital signs, CAM-ICU (for assessing delirium), RASS (agitation/sedation), BPS or NRS (pain).

Analgesic administration doses and ventilation settings were also acquired. Fisher's exact test or chi-square test were used to evaluate differences in the main outcomes among the three groups; p values <0.05 indicated statistical significance.

Results:

Fifteen patients (males 87.5%, age 61±13 years) were enrolled: 4 in control group, 5 in the standardized MT group, and 6 in the individualized MT group. Mean time on MT was 4.4±4 days. As compared to control group, NRS and BPS showed a reduction in patients receiving standard MT of 14.3% and 1.3%, respectively (p=0.007). Compared to control group, patients receiving individualized MT, experienced a reduction in NRS and BPS of 63.4% and 6.6%, respectively. Cumulative dose of analgesics was not different in the MT groups, although it was reduced by 60% in the standard MT group (p=0.068). CAM-ICU and RASS were not different in the 3 groups.

Conclusions:

These preliminary results suggest that music therapy could reduce pain perception and dosage of analgesics in critically ill patients, without affecting other parameters. Further data are required to assess the role of this nonpharmacological therapy in ICU.

### A131. Intravenous naloxone for perindopril poisoning: a case report

#### Negri K.^1^, Lombardo A.P.^2^, Fassini P.^2^, Mistraletti G.^1^

##### ^1^Dipartimento di Fisiopatologia medico-chirurgica e dei trapianti, Università degli Studi di Milano ~ Milano ~ Italia, ^2^A.S.S.T. Ovest Milanese Ospedale Nuovo di Legnano ~ Legnano ~ Italia

###### **Correspondence:** Negri K.

Background

Perindopril is an angiotensin-converting enzyme inhibitor (ACE-I) commonly used in the treatment of hypertension. ACE-I poisoning leads to multi-organ failure secondary to refractory vasoplegic shock (1). Intravenous (IV) naloxone may revert the prolonged vasodepressor activity of endogenous opioid byproducts.

Case Report

A 50-year old male with a history of hypertension and major depression with previous suicide attempts referred to our intensive care unit (ICU) at Ospedale Nuovo Legnano, for an intentional ingestion of amlodipine (900mg) and perindopril (900mg). Upon arrival in the emergency department, he was alert, oriented, hypotensive and tachycardic; he received 1.5L of intravenous crystalloids and high dosage norepinephrine to maintain a mean arterial pressure >65mmHg. The Regional Poison Centre was informed, and high-dose insulin euglycemia therapy (HEIT) was started (1UI/kg in bolus, followed by 0.5UI/kg*h)(2,3). The patient then underwent endoscopic gastric aspiration and bowel preparation. The patient was admitted to our ICU due to his evolving vasoplegia, requiring increasing vasoactive support with norepinephrine 0.5mcg/kg*min and vasopressin 0.03 UI/min. During ICU stay, he developed lethargy and hypoxemia managed with non-invasive strategies and stage 3 acute kidney injury treated conservatively. A transthoracic echocardiogram revealed normal biventricular function. This management was continued for the first 72 hours of admission with no change in vasoactive support; HIET therapy was weaned progressively as insulin sensitivity improved, a sign of recovery from calcium channel blocker poisoning(4). Given the impossibility of de-escalating vasoactive support, a literature review indicated previous attempts to treat vasoplegia secondary to ACE-I with intravenous naloxone(2,3). We followed the indications proposed by Trivedi et al., administering 0.2mg aliquots of IV naloxone every 5 minutes for a total dose of 1.6mg. His vasopressor requirements halved within the first hour, and within the second-hour, vasopressin was suspended. An infusion of 0.2mg/h of IV naloxone was initiated 3 hours later and continued for 27 hours (5.4mg). Vasopressors were entirely weaned after 10 hours, kidney function and urinary output drastically improved and the patient was discharged from ICU on day 5. No complications of IV naloxone, or usual side effects occurred.

ACE-I overdose manifests with vasoplegia and hypoperfusion induced multi organ failures: severe refractory hypotension, respiratory distress, acute kidney injury and altered mental status(1). Supportive therapies are the mainstay of ACE-I poisoning. Naloxone is a competitive opiate receptor antagonist used for opioid overdose. No recommendation exists for its role in ACE-I poisoning even though previous reports(2,3) show its use in these conditions. As for the case of Trivedi et. Al. our patient presented with a mixed overdose of amlodipine and perindopril and haemodynamic improvements were only seen after IV naloxone administration. The mechanism behind this depends on the interplay between ACE-I and the endogenous opioid system: the antagonism of enkephalinase, which leads to enkephalin accumulation, increases vasoplegia and central inhibition of angiotensin II activity (5).

Conclusions

ACE-I overdose causes profound vasoplegic shock and IV naloxone may be used to overcome the excess vasodepressor activity of endogenous opioids.

Patient informed consent was obtained prior to writing this case report.

Bibliography

1. Lucas C, Christie GA, Waring WS, Rapid onset of haemodynamic effects after angiotensin converting enzyme-inhibitor overdose: Implications for initial patient triage. Emergency Medicine Journal. 2006;23(11):854-7.

2. Trivedi V, Glezerson BA, Chaudhuri D, Davidson M, Doufle G. Naloxone as an antidote for angiotensin converting enzyme inhibitor poisoning: a case report. Canadian Journal of Anesthesia. 2020;67(10):1442-3.

3. Varon J, Duncan SR. Naloxone reversal of hypotension due to captopril overdose. Annals of Emergency Medicine. 1991;20(10):1125-7.

4. Krenz JR, Kaakeh Y. An overview of Hyperinsulinemic-Euglycemic Therapy in Calcium Channel Blocker and β-blocker Overdose. Pharmacotherapy. 2018;38(11):1130-42.

5. di Nicolantonio R, Hutchinson JS, Takata Y, Veroni M. Captopril potentiates the vasodepressor action of Met- enkephalin in the anaesthetized rat. British Journal of Pharmacology. 1983;80(3):405-8.

### A132. Treatment of post-traumatic delirium in a patient with polytrauma

#### Sicilia R., Manzione N., Arminio D., Landri P., Chiumiento C., Tozzi U., D”Elia A., Pisapia A., Buonavolontà C., Chiumiento F., Di Lascio C.

##### ASL Salerno ~ Battipaglia ~ Italy

###### **Correspondence:** Manzione N.

Introduction

Post-traumatic stress disorders affect the outcome of polytrauma patients in the ICU. It is a psychiatric syndrome characterized by alteration of consciousness and attention, global disorder of the cognitive state, psychomotor disorders, disturbances of the emotional sphere, such as anxiety, fear, mood instability, psychomotor agitation. Approximately 23% of ICU polytrauma patients develop PTDS. The syndrome is conditioned both by any central lesions and by the septic state frequent in polytrauma.

Materials and methods

Our work was based on the evaluation of the multimodal pharmacological approach in a case of PTDS at the time of post-tracheostomy pharmacological degresing. In this phase the patient presented: aversive attitude, important psychomotor agitation, structured delirium, hallucinations. , in relation to the clinical importance of the trauma to deeply sedate the patient with Propofol 2.5mg / kg / min and Ultiva 0.08ℽ / kg / min. the subsequent pharmacological weaning was unsuccessful with the reappearance of the PTDS. It was decided to associate Dexmedetomidine with the two drugs previously used. Dexmedetomidine was administered as a continuous iv infusion. at a dosage of 0.6-0.7ℽ / kg / hour, with half-dose reduction of propofol and remifentanil. The following were observed: persistence and maintenance of structured delirium, reduction of aversion, worsening of the state of psychomotor agitation. At this point, also with the skilful collaboration of neuropsychiatric colleagues, it was decided to approach PTDS with Valproic Acid IV 6mg / kg three times a day and in the interval phases with Promazine Hydrochloride IV.After about 4 days of treatment, the symptoms were stopped. PTDS, with slow but continuous cognitive rebalancing.

Results

The results obtained underline the importance of the multi-drug approach to PTDS, the good efficacy of Dexmedetomidine in the treatment of delirium, which in this case were accompanied by drugs such as mood stabilizing Valproic Acid and Promazine.

Conclusions

It is essential to recognize PTDS early and treat it early because post-traumatic delirium considerably lengthens the length of stay in the ICU, resulting in a delay in healing.

Bibliography

Ely EW, Dubois MJ, Truman B, Dial S: intensive care delirium sceening checklist: evaluation of a new screaning tool. Intensive care Med 2001, 27: 859-864

### A133. Rapid response system the organizational challenges after publication of the tuscany region guideline experience of a hospital all to "reorganize"

#### Barneschi C., Pieri S.

##### Azienda USL-Sudest Toscana Dipartimento Emergenza Urgenza UOC Anestesia e Rianimazione ~ Arezzo ~ Italy

###### **Correspondence:** Barneschi C.

According to the Regional Guidelines for the Management of Intrahospital Emergencies (DGRT 272/19), hospitals of Tuscany have gone to a clinical and organizational remodeling of emergency response systems.

The Intra-Hospital Emergency (EI) or Rapid Response System (SRR) was created to ensure an effective and advanced health response to clinical emergencies that can occur in all hospital areas and involve all kinds of hospital users.

Its design includes an afferent branch, founded on surveillance systems, clinical monitoring and identification of uniform alert criteria (through a shared score) and an efferent, which is responsible for the health response to critical events.

Specifically this latest model offers a modulated response to the expected critical nature of the patient, ensuring a proportionally more advanced health response ranging from basic medical response with BLSD approach to advanced MET intervention with ALS.

The realization of the SRR System in my hospital “Ospedale Riuniti della Valdichiana Senese” USL Toscana SUDEST, which began in September 2021 and ended with its activation on April 1, 2022 produced the review of numerous clinical and organizational areas thanks to the collaboration of a wide range of professional figures:

♣ Infrastructural aspects: definition of areas of emergency services, definition of dedicated routes to improve MET response in terms of timeliness and efficiency, the identification of communication systems through the establishment and implementation of the single emergency number (NUEI 2222)

♣ Staff and competences: increase the number of MET members operating in the same service (ICU department), in order to avoid delays in intervention due to the relocation of staff and need for separate activation, as well as a non-homogeneous response of assistance. MET figures share high intensive skills, capacity to manage major clinical emergencies and airway, as well as collaboration and integration with other health professionals both in intra and extra hospital situation. Clinical areas staff must have basic BLSD skills in order to be able to manage the acute clinical deterioration early in advanced response.

♣ Equipment and Technology: organization of instruments, drugs for MET (bag) and trucks with BLSD response for clinical areas.

♣ Operating modes: implementation of EBM-based clinical procedures, with particular reference to the implementation of clinical surveillance systems through the use of NEWS Score, to the activation criteria and then to the expected response type.

♣ Training: implementation of annual training plans for all professionals involved in the emergency plan through the execution of BLSD, ALS courses and new formulation courses such as on-site microsimulations.

The creation of a Rapid Response System represents still today a challenge for health organizations because of the difficulty in coordinating in parallel the various clinical, technical and organizational fields.

### A134. Venous thrombosis in critical patients in multi-purpose intensive care: comparison between the use of pneumatic knee-highs and graduated compression elastic stockings

#### Licata A.^1^, Pizzo M.^1^, Orlandi E.^1^, Santini G.^1^, Coratti G.^2^, Mancini S.^2^, Scolletta S.^2^

##### ^1^Dipartimento di Scienze Mediche, Chirurgiche e Neuroscienze, Università degli Studi di Siena ~ Siena ~ Italy, ^2^Azienda Ospedaliera Universitaria Senese ~ Siena ~ Italy

###### **Correspondence:** Licata A.

Introduction: Among the different complications developed by the critically ill patient in ICU, we ascribe deep vein thrombosis (DVT) and pulmonary thromboembolism (PE). The ecocolorDoppler represents the baseline survey for the diagnosis of DVT, whose prophylaxis is based on the use of anticoagulants and mechanical systems such as graduated compression elastic stockings and/or pneumatic knee-highs.

Objectives: The purpose of the study is to analyze whether the use of pneumatic knee-highs leads to a reduction in the incidence of thromboembolic events compared to the use of graduated compression elastic stockings in patients undergoing drug prophylaxis. The study also evaluates a possible correlation between the SOFA (Sequential Organ Failure Assessment Score) at admission and the development of DVT during hospitalization.

Materials and Methods: The study includes 209 patients, hospitalized from January 2019 to March 2021, at the intensive care of the Anesthesia and Resuscitation Unit DEA and Transplantation of the AOU Senese. The subjects, observed from the beginning of hospitalization until discharge, were divided into four groups with respect to the admission class: medical, surgical, traumatological and cranial and monitored using the echocolorDoppler method with CUS (Compression UltraSonography) once a week.

Results: 13 of 209 patients (6.2%) developed DVT, of which 2 developed PE (0.9%). In the group treated with graduated compression stockings we had 5 DVT (6.8%) and 1 PE (1.4%) on 74 patients, while in the group treated with pneumatic knee-high socks 8 DVT (5.9%) and 1 PE (0.7%) on 135 patients. The analysis did not reveal significant differences in the development of DVT and PE between these two groups (p = 0.774). We detected 1 DVT (3.3%) and no PE on 30 surgical class patients, 4 DVTs (8.9%) and no PE on 45 cranial class patients, 2 DVTs (2.5%) and no PE on 80 patients of the medical class and 6 DVT (11.1%) and 2 PE (3.7%) out of the 54 patients of the trauma class. The analysis did not show significant differences in DVT and PE between these classes (p = 0.165). However, through the Mann-Whitney U test with independent samples, we observed a significant correlation (p = 0.002) between the occurrence of DVT and hospitalization times: the mean days were 9.5 ± 9.2 in the group treated with elastic stockings and 13.4 ± 14.1 in the group treated with pneumatic knee-highs. Furthermore, through a logistic regression analysis, we found a correlation between the development of DVT (p = 0.006) and the entry SOFA, result 5.9 ± 3.0 in the group treated with elastic stockings and 7.3 ± 3.2 in the group treated with pneumatic knee-highs. We searched for a SOFA value to be used as a predictive cut-off for DVT, however the test was not significant for DVT (p = 0.141).

CONCLUSIONS: Our study did not show significant differences in the occurrence of DVT between the two groups, however an increased risk of thrombosis was found with hospitalization time.

### A135. Care pathway of the major burn victim: a clinic case

#### Truzzi G., Mereto N., Bianco M., Rusca G.

##### Ospedale Villa Scassi ~ Genova ~ Italy

###### **Correspondence:** Truzzi G.

Introduction

A "major burn victim" is defined as a patient with burns involving more than 20% of the total body surface area (TBSA) or with a percentage of deep injuries greater than 10% or, in children younger than 18 months of age or elderly, less extensive burns.

The American Burn Association estimates that 45,000 people per year in the U.S. are hospitalized for burn-related injuries; half of which require treatment at Burn Centers.

WHO draws attention to the 180,000 annual deaths, hospitalizations, and disabilities due to thermal injuries.

In 2020, Europe called on member states to have disaster-specific national plans for burn injuries.

Given the epidemiology and standard care complexity for these types of patients, it will be crucial, moving forward, to conceive the care of the "major burn victim" as a comprehensive continuum from the prehospital setting to dedicated intensive care units, accomplished by multidisciplinary collaboration.

Case Study

We describe the case of a 56-year-old man in good health who, due to an electrical panel explosion in a highway tunnel, presented with burns over 60% of the body surface and suspected injury from both smoke inhalation and electrocution. Initial attempts at stabilization and intubation were achieved by the Territorial Emergency physician and the patient was then transferred by helicopter to the Burn Center at Villa Scassi Hospital.

In the shock room, resuscitator and surgeon reevaluated the extent and depth of the injuries, ruling out systemic carbon monoxide and hydrogen cyanide intoxication and electrocution injury.

In the ICU, fluid resuscitation continued (in the first 8 h Ringer's lactate 2 ml/kg/%BBS -burned body surface-). Albumin 4% 1 ml/kg/%BBS was administered over the next 16 h for albuminemia < 25g/L and continued crystalloid 2 ml/kg/%BBS, maintaining diuresis between 0.5- 1 ml/kg/h. On the second day, intakes corresponded to 50% from the first 24 h with target euvolemia.

A series of fibro bronchoscopies were performed in order to stage and monitor inhalation related injuries, early tracheostomy on day 10 and ventilation with 4-8 ml/kg tidal volume, PEEP between 5 and 10 cmH2O, pPlat tolerated up to 35 cmH2O until early thoracic escharotomy (within the 48 h) performed by the surgical team, which will allowed improved protective ventilatory values.

With the dietetics service, dieticians identified specific nutritional targets and appropriate preparations were implemented.

With the Infectious Disease service, blood culture analysis on day 8 returned positive results and BAS for Acinetobacter baumanii. Due to its resistance profile, a course of Cefiderocol, in combination with Colistin aer. and Ampicillin/Sulbactam, was administered to the patient.

During hospitalization, patient underwent balneotherapy with advanced dressings , facilitated by the cooperation of surgeons and anesthesiologists. On discharge, with home care activated and visits scheduled for dressing changes, patient was included in the territorial pain therapy network.

After about 40 days, complete healing was observed.

Discussion and Conclusions

This is an exemplary case of how the expertise and cooperation of different professionals can lead to positive outcomes in highly complex cases of multisystem trauma (burn disease, electrocution risk, high mortality infectious complications). We believe that increasing technical expertise and creating shared pathways between different professional figures can improve the standard of care.

Informed consent to publish had been obtained.

### A136. Anesthetic and surgical management on trauma patient guided by ultrasound approach and monitoring (case report)

#### Cammarata G., Cardì S., Carnemolla V., Cavallo L., Ciccarelli M., Di Falco L., Gulisano L., Rampulla S., Santangelo M., Stazzone C., Stazzone G., Testa M., Rapisarda G.

##### U.O. Anestesia e Rianimazione - P.O. S. Marta e S. Venera, Acireale (ASP-CT) ~ Acireale (CT) ~ Italy

###### **Correspondence:** Cammarata G.

Background

The usefulness of ultrasound approach for the management of patients who arrive at the emergency department (ED) following a traumatic event has been known for decades. The following case report represents a specific example about the importance of ultrasound in anesthetic and surgical management of complex trauma.

Case report

A 23-year-old boy arrived at the ED after a violent car accident. Upon arrival, the patient was understandably painful, moderately tachypnoic and cooperative. The eco-fast study showed a thin layer of right PNX and a conspicuous blood collection in the Morison’s pouch. Due to the hemodynamic stability, it was decided to carry out a further diagnostic study with a CT examination that confirmed the presence of a right PNX and extensive hepatic and right renal lacerations in absence of active blood losses and in conjunction with a gastric cavity hyper-distended by food (Fig. 1a, 1b). X-ray examination documented the fracture of the right elbow that appeared during the inspection to be exposed (Fig 1c). The continuous stability of vital signs and hemoglobin values led abdominal and thoracic surgeons towards a wait-and-see approach. On the contrary, surgical approach was mandatory for the orthopedic surgeons in order to stabilize the exposed upper limb fracture with an external fixation. To make this last procedure possible, we opted for an ultrasound-guided block of the brachial plexus via the right supraclavicular route (Fig 1d) with 20 ml of Mepivacaine 0.5%. In order to evaluate any changes of the thoracic and abdominal lesions described above, a repeated intraoperative ultrasound monitoring was carried out. The same strategy was then used to monitor the lesions in the first hours of the postoperative period.

Conclusion

Ultrasound has increased the probability of peripheral block success avoiding the need of intubation, risky on a full stomach patient, and of chest drainage necessary in the case of mechanical ventilation.

Continuous intraoperative ultrasound evaluation of the thorax and abdomen allowed constant monitoring of the lesions in a period not suitable with other diagnostic methods. Remaining awake, the patient has indirectly contributed to an uninterrupted assessment of his clinical condition, giving to the entire team a continuous feedback regarding the value of a laparotomy abstention choice. This case report confirms the ultrasound value on trauma patient management from arrival at the ED to both intra and post-operative evaluation. Direct and indirect ultrasound contribution guided both the anesthetic and surgical approaches, avoiding unnecessarily more aggressive and risky attitudes.

Informed consent to publish was obtained.

**Fig. 1 (abstract A136).**
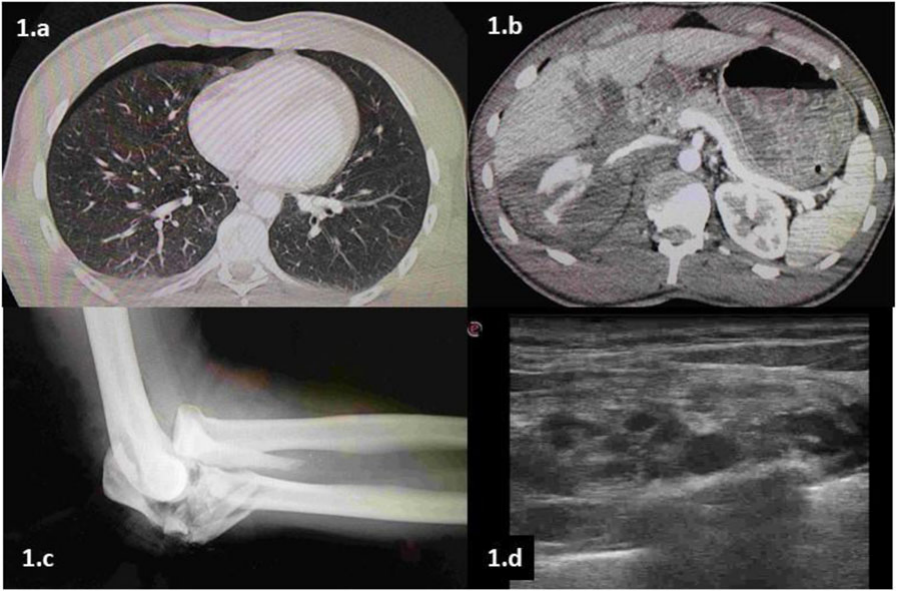
a TC Torace, 1.b TC addome, 1.c Rx gomito dx, 1.d Eco plesso sovraclaveare

## Medicina perioperatoria pediatrica e neonatale

### A137. Oral midazolam before intra-articular corticosteroid injections in juvenile idiopathic arthritis

#### Burrone M.^1^, Guazzi M.^1^, Naddei R.^1^, Spelta M.^1^, Malattia C.^1^, Disma N.M.^2^, Ravelli A.^3^, Consolaro A.^1^

##### ^1^UOC Clinica Pediatrica e Reumatologia, Istituto Giannina Gaslini ~ Genova ~ Italia, ^2^UOC Anestesia e Terapia del dolore Acuto e Procedurale ~ Genova ~ Italia, ^3^Direzione Scientifica, Istituto Giannina Gaslini ~ Genova ~ Italia

###### **Correspondence:** Burrone M.

Introduction

Intraarticular corticosteroid injections (IACI) are widely used in the management of juvenile idiopathic arthritis (JIA) to induce rapid relief of symptoms of active synovitis and to obtain resolution of functional impairment. Midazolam, a short acting benzodiazepine, has been found to be an effective premedication in children by reducing anxiety and mitigating uncooperative behavior with a large margin of safety. Midazolam is primarily administered intravenously; however, recently a new oral solution designated for procedural sedation in children has been introduced. Several studies in this field have shown that a child’s level of anxiety before a painful medical procedure is directly correlated with the amount of pain experienced.

Methods

In this one month pilot study, all consecutive patients who were prescribed one or more IACIs by the caring physician at the study Unit were proposed to participate in the study. Premedication with midazolam was offered to an unselected group of children based on the availability of the procedural anesthesia team of the Gaslini Institute. All children received local anesthesia. Patients in Group 1 received 0.25 mg/kg (maximum 15 mg) of oral midazolam solution and local anesthesia with lidocaine/prilocaine 5% cream, 30 to 45 minutes before IACI; patients in Group 2 received only local anesthesia. Patients’ pain experienced before and at the end of the procedure were assessed using a 0-10 21-circle visual analog scale (VAS). Subjective rating of the ease of performing the procedure by the operator was recorded on a 4 points Likert scale (“Very easy”, “Easy”, “Quite difficult”, “Very difficult”). The level of anterograde amnesia was recorded on a 3 points Likert scale (“No amnesia”, “Partial amnesia”, “Complete amnesia”). During procedure and until two hour after, vital signs and oximetry were monitored and recorded in children receiving midazolam.

Results

Seven patients were enrolled in Group 1 and seven patients in Group 2. Patients receiving only local anesthesia were older (mean age at procedure 16 years for Group 2, 12 years for Group 1, p = 0.04); 28% of patients received multiple IACIs in both groups. Pain level prior the procedure was similar in the two groups (p = 0.7). Patients received midazolam reported less pain (mean score 2.1) compared those receiving local anesthesia alone (mean score 3.4) at the end of the procedure, although the difference was not significant (p = 0.25). In 28% of cases physician described the procedure as difficult in Group 2, compared to 14% in Group 1. All patients receiving midazolam reported some degree of anterograde amnesia (28% reported complete amnesia, 72% partial amnesia) when recalling the procedure. No adverse events were recorded in both groups.

Conclusion

In this small cohort of children. Although not significantly, oral midazolam reduced pain during IACIs and increased the ease of the procedure. Oral midazolam is a safe and effective premedication before IACI in patients who require or prefer sedation and improves the child’s perception of the procedure, by inducing anterograde amnesia. This pilot study opens to further investigation, aiming to contribute to improvement in patient care and outcomes.

### A138. Fluid responsiveness in the pediatric population: is there a role for non-invasive dynamic parameters?

#### De Donato F.^1^, Pilia E.^2^, Fioccola A.^4^, Gobbi L.^4^, Rufini P.^3^, Serio P.^3^, Ricci Z.^4^

##### ^1^Department of Anesthesia and Intensive Care, IRCCS San Raffaele Scientific Institute, Milan, Italy ~ Milano ~ Italia, ^2^Department of Medical Science and Public Health, University of Cagliari, Cagliari, Italy ~ Cagliari ~ Italia, ^3^Department of Anesthesiology and Critical Care Medicine, Pediatric Intensive Care Unit, Meyer Children's Hospital, Florence, Italy ~ Firenze ~ Italia, ^4^Department of Health Science, Section of Anesthesiology and Intensive Care, University of Florence ~ Firenze ~ Italia

###### **Correspondence:** De Donato F.

**Background**

The assessment of the hydration status is pivotal to conduct a safe anesthesia in children and avoid post-operative complications. Typically, intraoperative volume replacement is based on the Holliday and Segar’s formula, heart rate, arterial blood pressure and urine output monitoring [1,2]. Many studies have demonstrated that static parameters are suboptimal predictors of fluid responsiveness [3,4]. Several dynamic parameters have been developed to guide fluid replacement, either assessed from invasive arterial pressure (i.e., pulse pressure variation -PPV-), or non-invasive (i.e., pleth variability index -PVi-) or from ultrasound methodology(i.e., respiratory aortic blood flow velocity -△Vpeak-) [4-7]. The aim of this systematic review is to understand if non-invasive parameters can be used during pediatric non cardiac surgery to identify fluid responders.

**Materials and Methods**

We searched data from PubMed up to May 15^th^, 2022, for peer-reviewed studies comparing non-invasive dynamic parameters to any other method of fluid responsiveness assessment in the pediatric setting.

**Results**

We included 8 prospective observational studies. The parameters analyzed were PPV, stroke volume index (SVI), stroke volume variation (SVV), and △Vpeak. Non-invasive methods were PVi and variations in pulse oximetry plethysmographic waveform amplitude (ΔPOP). Four studies found a correlation between dynamic parameters and fluid responsiveness; four studies failed to discriminate between fluid responders and non-responders [8-15]. The performance of non-invasive methods was fair to low in the analyzed studies (Table 1).

**Conclusions**

In the pediatric population predicting fluid responsiveness is difficult. According to available evidence, △Vpeak is still the ‘golden standard’ to predict fluid responsiveness in children. However, it requires technical skills, and it is not always feasible. Similarly, invasive arterial pressure and PPV are rarely available in children. Due to conflicting data, more studies on non-invasive dynamic parameters are required to establish their ability to predict fluid responsiveness in children.

**References**

1. Bailey AG, McNaull PP, Jooste E, Tuchman JB. Perioperative crystalloid and colloid fluid management in children: where are we and how did we get here? Anesth Analg. 2010 Feb 1;110(2):375-90. doi: 10.1213/ANE.0b013e3181b6b3b5. Epub 2009 Dec 2. PMID: 19955503.

2. Holliday MA, Segar WE, Friedman A. Reducing errors in fluid therapy management. Pediatrics. 2003 Feb;111(2):424-5. Erratum in: Pediatrics. 2003 Apr;111(4):924. PMID: 12563072.

3. Wiesenack C, Fiegl C, Keyser A, Prasser C, Keyl C. Assessment of fluid responsiveness in mechanically ventilated cardiac surgical patients. Eur J Anaesthesiol 2005; 22: 658–65

4. Lee JH, Kim EH, Jang YE, Kim HS, Kim JT. Fluid responsiveness in the pediatric population. Korean J Anesthesiol. 2021 Apr;74(2):188. doi: 10.4097/kja.19305.e1. Epub 2021 Mar 26. Erratum for: Korean J Anesthesiol. 2019 Oct;72(5):429-440. PMID: 33794567; PMCID: PMC8024214.

5. Michard F. Changes in arterial pressure during mechanical ventilation. Anesthesiology. 2005 Aug;103(2):419-28; quiz 449-5. doi: 10.1097/00000542-200508000-00026. PMID: 16052125.

6. Morparia KG, Reddy SK, Olivieri LJ, Spaeder MC, Schuette JJ. Respiratory variation in peak aortic velocity accurately predicts fluid responsiveness in children undergoing neurosurgery under general anesthesia. J Clin Monit Comput. 2018 Apr;32(2):221-226. doi: 10.1007/s10877-017-0013-3. Epub 2017 Mar 16. PMID: 28299589.

7. Desgranges FP, Evain JN, Pereira de Souza Neto E, Raphael D, Desebbe O, Chassard D. Does the plethysmographic variability index predict fluid responsiveness in mechanically ventilated children? A meta-analysis. Br J Anaesth. 2016 Sep;117(3):409-10. doi: 10.1093/bja/aew245. PMID: 27543550.

8. Kim EH, Lee JH, Jang YE, Ji SH, Kim HS, Cho SA, Kim JT. Prediction of fluid responsiveness using lung recruitment manoeuvre in paediatric patients receiving lung-protective ventilation: A prospective observational study. Eur J Anaesthesiol. 2021 May 1;38(5):452-458. doi: 10.1097/EJA.0000000000001387. PMID: 33186310.

9. Julien F, Hilly J, Sallah TB, Skhiri A, Michelet D, Brasher C, Varin L, Nivoche Y, Dahmani S. Plethysmographic variability index (PVI) accuracy in predicting fluid responsiveness in anesthetized children. Paediatr Anaesth. 2013 Jun;23(6):536-46. doi: 10.1111/pan.12139. Epub 2013 Mar 23. PMID: 23521073.

10. Byon HJ, Lim CW, Lee JH, Park YH, Kim HS, Kim CS, Kim JT. Prediction of fluid responsiveness in mechanically ventilated children undergoing neurosurgery. Br J Anaesth. 2013 Apr;110(4):586-91. doi: 10.1093/bja/aes467. Epub 2012 Dec 18. PMID: 23250892.

11. Song LL, Geng ZY, Ma W, Liu YF, Wang DX. Dynamic variables predict fluid responsiveness in pre-school and school children undergoing neurosurgery: a prospective observational study. Transl Pediatr. 2021 Nov;10(11):2972-2984. doi: 10.21037/tp-21-281. PMID: 34976763; PMCID: PMC8649593.

12. Feldman JM, Sussman E, Singh D, Friedman BJ. Is the pleth variability index a surrogate for pulse pressure variation in a pediatric population undergoing spine fusion? Paediatr Anaesth. 2012 Mar;22(3):250-5. doi: 10.1111/j.1460-9592.2011.03745.x. Epub 2011 Dec 6. PMID: 22142032.

13. Ji SH, Song IK, Jang YE, Kim EH, Lee JH, Kim JT, Kim HS. Comparison of pulse pressure variation and pleth variability index in the prone position in pediatric patients under 2 years old. Korean J Anesthesiol. 2019 Oct;72(5):466-471. doi: 10.4097/kja.19128. Epub 2019 Jun 20. PMID: 31216847; PMCID: PMC6781221.

14. Pereira de Souza Neto E, Grousson S, Duflo F, Ducreux C, Joly H, Convert J, Mottolese C, Dailler F, Cannesson M. Predicting fluid responsiveness in mechanically ventilated children under general anaesthesia using dynamic parameters and transthoracic echocardiography. Br J Anaesth. 2011 Jun;106(6):856-64. doi: 10.1093/bja/aer090. Epub 2011 Apr 26. PMID: 21525016.

15. Chen PH, Chan KC, Liao MH, Wu CY. Accuracy of dynamic preload variables for predicting fluid responsiveness in patients with pediatric liver cirrhosis: A prospective study. Paediatr Anaesth. 2020 Apr;30(4**):455-461. doi: 10.1111/pan.13819. Epub 2020 Jan 20. PMID: 319009**69.

**Table 1 (abstract A138).** Parameters, area under receiver operating characteristic (ROC) curve, sensitivity, specificity and threshold

Studies	Year	Setting	Patients n.	Parameters	Area under the ROC	Sensitivity	Specificity	threshold for responders
Kim [8]	2021	Operatory Room	30	△POP PPV PVi	0.58 (0.395-0.76) 0.53 (0.34-0.71) 0.79 (0.60-0.91)	/ / 73.6%	/ / 81.8%	/ / 11.6%
Julien [9]	2013	Operatory Room	54	PVi SVI	0.8 (0.7-0.89) 0.85 (0.77-0.93)	80% 64%	80% 83%	16.5% 25 ml·m^-2^
Byon [10]	2013	Operatory Room	33	PPV PVi △Vpeak	0.54 (0.34-0.74) 0.76 (0.59-0.93) 0.80 (0.64-0.96)	/ 73.3% 86.7%	/ 86.7% 72.2%	/ >11% >11%
Song [11]	2021	Operatory Room	44	PPV SVV PVi △Vpeak	0.68 (0.51-0.83) 0.71 (0.53-0.85) 0.66 (0.47-0.81) 0.75 (0.58-0.89)	55% 50% 80% 60%	79% 88% 63% 92%	11% 9% 13% 12.5%
Feldman [12]	2011	Operatory Room	24	PPV PVi	/ /	/ /	/ /	/ /
Ji [13]	2019	Operatory Room	27	PPV PVi	/ /	/ /	/ /	/ /
Pereira de Souza Neto [14]	2011	Operatory Room	30 (0-6 age group n 19, 6—14 age group)	*0-6 age group* PVi △POP PPV △Vpeak *6-14 age group* PVi △POP PPV △Vpeak	0.63 (0.38-0.84) 0.51 (0.28-0.74) 0.52 (0.29-0.76) 1.0 (0.82-1.00) 0.54 (0.24-0.82) 0.57 (0.27-0.84) 0.6 (0.29-0.86) 1.0 (0.73-1.00)	/ / / / / / / /	/ / / / / / / /	/ / / >10% / / / >10%
Chen [15]	2020	Operatory Room	27	PPV SVV PVi	0.67 (0.52-0.82) 0.68 (0.54-0.83) 0.57 (0.40-0.74)	46.7% 80% /	80.4% 54.4% /	13% 10% /

### A139. Effects of Non-Invasive Ventilation (NIV) and Continuous Positive Airway Pressure (CPAP) on ventilation distribution measured by Electrical Impedance Tomography (EIT) in pediatric sedation

#### Chidini G., Scalia Catenacci S., Marchesi T., Veronese A., Babini G., Montani C., Calderini E.

##### Fondazione IRCCS Cà Granda Ospedale Maggiore Policlinico ~ Milano ~ Italia

###### **Correspondence:** Chidini G.

Background. Spontaneous Breathing (SB) during deep sedation in pediatric anaesthesia could results in a re-distribution of ventilation towards lungs non-dependant areas (ventral areas in supine position) and/or atelectasis [1]. In this setting Non-Invasive Ventilation (NIV) could prevent atelectasis making ventilation more homogeneous and thus increasing functional residual capacity. Electrical impedance tomography (EIT) is a non-invasive, bedside, radiations-free diagnostic tool, feasible in paediatric patients. It allows to study ventilation distribution dividing the lungs in four Region Of Interest (ROI) by measuring distribution of ventilation and the response to maneuvers, such as anaesthesia or PEEP-application [2]. The main EIT related parameters are the Center of ventilation (CoV), Global Inhomogeneity Index (GI) (the difference in impedance between end-inspiration and end-expiration in studied ROIs) and Regional Ventilation Delay (RVD), an index of inhomogeneity in regional inspiratory time constant.

Materials and methods. The aim of this pilot study was to describe ventilation distribution using thoracic EIT in paediatric healthy patients undergoing SB deep sedation receiving NIV.

The primary end-point was RVD. No previous data of similar study are existing, so we are not able to calculate a determined sample size as for pilot physiological studies. Data were analyzed with nonparametric ANOVA with Bonferroni correction (significative if p-value <0.05).

Inclusion criteria were: age> 29d< 10yrs, ASA<2; sedation time >30mins. Were excluded children with chronic or restrictive lung diseases, neuromuscular or metabolic disorders, presence of implantable devices. After ten minutes of EIT recording during SB, standardized sedation was delivered with intravenous fentanyl (1-2 mcg/kg) and propofol 4-8 mg/kg/hr (target BIS 40-60). An oxygen mask was placed to reach a target SpO2 95% without bag ventilation. Thereafter, two NIV 20-mins trials (CPAP5 cmH2O and NIV S/T mode IPAP 10cmH2O EPAP 5 cmH2O) were delivered in a randomized sequence by a turbine ventilator in a single limb configuration with a vented nasal mask with standard monitoring (EKG, SPO2, NIBP)

Results. Were enrolled 13 children, mean age 7±2,8 yrs, Weight 26±11 kg height 123± 9 cm Results are reported in Figure 1. RVD was severly increased by sedation. NIV was able to reduce RVD comparing to SBsed whereas CPAP was not efficient, may be due to a less efficacy in airways stenting during CPAP (Figure 1, A; p<0.05). GI trends was similar to RVD showing the major inhomogeneity in ventilation during SBsed(p<0.05).

Conclusions. Our preliminary data may indicate that NIV applied during SBsed reduces regional delays in dependent lung regions leading to a global improvement of ventilation distribution. Further data are needed to confirm these results.

References

1. Spinelli E., Mauri T., Fogagnolo A. et al. Electrical impedance tomography in perioperative medicine: careful respiratory monitoring for tailored interventions. BMC Anaesthesiology , 2019; 19:140-146

2. Bordes J., Goutorbe P., Cungi P.J et al. Noninvasive ventilation during spontaneous breathing anesthesia: an observational study using electrical impedance tomography. Journal of Clinical Anesthesia, 2016; 34,:420-426

**Fig. 1 (abstract A139).**
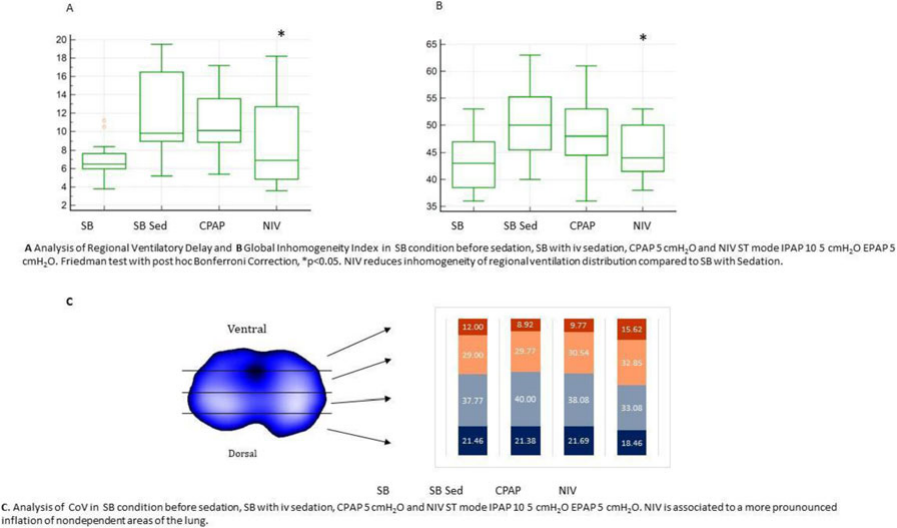
See text for description

## Metabolismo, nutrizione e terapie renali

### A140. Enrichment of personalized parenteralartificial nutrition with smof lipids and arginine in complicated patients undregoing major abdominal surgery

#### Basso A.

##### Aorn Antonio Cardarelli ~ Napoli ~ Italia

Enrichment of personalized parenteral artificial nutrition with SMOF lipids and arginine in complicated patients undergoing major abdominal surgery

Antonietta Basso, Mafalda Amente, Antonio Frangiosa - Cardarelli Hospital of Naples Anesthesia and Resuscitation Service, Postoperative Intensive Care

Background

Personalizing artificial nutrition is now scientifically proven to be one of its cornerstones, and it is fundamentally codifying it, especially since mortality seems to be comparable in patients undergoing enteral and / or parenteral nutrition, with the same quality of nutrients and the right amount of calories.

Case report

We treated 10 patients undergoing elective major abdominal surgery, initial MUST> 2, septic complications in the postoperative predominantly surgical, assessment of needs with the Harris-Benedict formula and calculation of catabolism with 24-hour urine collection for azoturia.

Patients were treated with personalized galenic bags and always enriched with SMOF lipids and 5g of Arginine, we almost always used branched amino acids.

Conclusion:

Better immunological response, we took leukocytes, lymphocytes, interleukin 6, PCR, lactates as parameters, the patient also showed an improved clinical response, and greater hemodynamic stability.

Bibliografy

ESPEN guideline on clinical nutrition in the intensive care unit

ESPEN guideline: Clinical nutrition in surgery

Immune modulation by parenteral lipid emulsions

Geert JA Wanten, Philip C Calder The American Journal of Clinical Nutrition, Volume 85, Issue 5, May 2007, Pages 1171–1184, https://doi.org/10.1093/ajcn/85.5.1171SMOFlipid versus Intralipid in Postoperative ICU Patients ISSN:2374-4448

Enliven Archive | www.enlivenarchive.org 1 2014 | Volume 1 | Issue

Parenteral or Enteral Arginine Supplementation

Safety and Efficacy1–3

Martin D Rosenthal,4,5 Phillip W Carrott,6 Jayshil Patel,7 Laszlo Kiraly,8 and Robert G Martindale8

### A141. Dietary barley Β-D-glucan supplementation protects against heart-brain axis dysfunction in mice: a new approach of perioperative neuro/cardioprotection

#### Baroni C.^1^, Spalletti C.^2^, Agrimi J.^1^, Di Lascio N.^3^, Mastorci F.^3^, Beltrami A.P.^4^, Caleo M.^2^, Lionetti V.^1^

##### ^1^Unit of Translational Critical Care Medicine, Scuola Superiore Sant'Anna ~ Pisa ~ Italia, ^2^Neuroscience Institute, CNR ~ Pisa ~ Italia, ^3^Isntitute of Clinical Physiology, CNR ~ Pisa ~ Italia, ^4^Department of Medicine, University di Udine ~ Udine ~ Italia

###### **Correspondence:** Baroni C.

Background: The heart-brain axis (HBA) is a bidirectional flow of information that modulates tolerance to multiorgan injury in patients at high cardiovascular risk. Synergistic effect of psychosocial stress (PS) with pre-existing obesity, two main cardiovascular and cerebrovascular risk factors, leads to HBA dysfunction that is a cause of poor postoperative outcome. Since pre-operative nutritional interventions promote earlier surgical recovery, we hypothesized that regular dietary supplementation with barley-derived (1.3) β-d-glucan (βG), a natural water-soluble polysaccharide with epigenetic activities, prevents heart and brain injury in mice exposed to risk of HBA dysfunction (HBD).

Materials and Methods: HBD was induced by feeding 10-week-old male wild-type C57BL/6J mice a high-fat diet (HFD) for 18 weeks, which were exposed during the last 14 days of dieting to the PS induced by the resident intruder paradigm. Normal 10-week-old C57BL/6J male mice (CT group, n=5) were compared to age-matched untreated mice with HBD (HBD group, n=6) and mice fed with HFD supplemented with 3% βG from the eighth week of HFD through the end of PS exposure (HBD+βG group, n=6).

Results: HBD+βG showed spatial memory recovery and normalization of anxiety-related traits compared to HBD mice (p<0.05) as assessed by Y-maze and Elevated Plus Maze test. In addition, left ventricular (LV) ejection fraction was significantly improved (+15.68%) in HBD+βG vs. HBD, as assessed by echocardiography. Immunohistochemical analysis conducted on perfused hippocampal slices showed levels of markers of synaptic plasticity (parvalbumin-positive interneurons), neurogenesis (bromodeoxyuridine-positive cells) and astrogliosis (glial fibrillary acidic protein-positive astrocytes) comparable to the CT group, while dentate gyrus volume (Hoechst) did not change in HBD+βG as compared to HBD group. In the same animals, βG prevented cardiomyocyte hypertrophy and myocardial fibrosis compared to HBD hearts. In addition, the number of apoptotic cardiac cells was reduced in HBD+βG as compared to HBD mice (TUNEL+, p<0.01) related to reduced oxidative stress (p<0.01).

Conclusions: Dietary βG supplementation early in the development of HBD simultaneously prevented hippocampal and cardiac injury. While these results are limited to an experimental model of disease, they nevertheless suggest that dietary βG supplementation might be effective for developing a new approach of clinical perioperative neuro/cardioprotection.

### A142. Metformin - Associated Lactic Acidosis (MALA): case series

#### Bianco M., Truzzi G., Rusca G., Salvarezza C., Morra F., Mereto N.

##### SC Anestesia e Rianimazione Ospedale Villa Scassi ASL3 Genovese ~ Genova ~ Italy

###### **Correspondence:** Bianco M.

INTRODUCTION

MALA is a rare clinical condition due to the accumulation of metformin which causes severe lactic acidosis.

The estimated prevalence of MALA is between 2-9 cases/100000 patients/year with a mortality rate that ranges between 30-50%.

METHODS

From January 2021 we admitted to the ICU Ospedale Villa Scassi ASL 3 Genovese 9 patients with suspected MALA. We sampled and valued blood examen (renal function, ions, inflammatory index), emogasanalysis, sign and symptoms of presentation, pharmacological and extracorporeal therapies, anamnesis, home therapy with metformin and discharge.

RESULTS

The average age of the 9 patients was 70 years (range 64-81), BMI 26 kg/m2.

The typical presentation of MALA in the ER was characterized by severe metabolic and lactic acidosis (average pH 7, lactate 21 mmol/l, B£ - 22 mEq/l, HCO3 8 mmol/l), acute renal disfunction (GFR 12, BUN 162 mg/dl) 67% of the patients had hyperkalemia.

ALL of them referred vomiting and/or diarrhea, 22% decreased mental status, none of them had dehydration.

55% had abdominal pain, 33% hypothermia, 88% hypotension, nobody hypoglycemia.

All of them were admitted to the ICU, average stay was of 4 days. 80% received therapy with sodium bicarbonate. 55% were ventilated mechanically, no NIV. 89% received continuous renal replacement therapy (CVVHDF) with citrate anticoagulation. Only in one case we couldn’t start with the treatment due to cardiac arrest.

Mortality was 44%. literature recommends dosage of plasmatic metformin, but we couldn’t achieve it.

CONCLUSIONS

Our experience and the literature’s data show that acute renal failure in diabetics in home therapy with metformin can lead to MALA, severe metabolic and lactic acidosis with high mortality.

Therefore it's important to suspect and recognize as soos as possible in the ER the clinical presentation of MALA, support vital signs and begin extracorporeal treatment

## Neuroanestesia e neurorianimazione

### A143. ICP monitoring: is osnd measurement a useful screening tool for invasive method?

#### Mastria D., Ciccarese A., De Maglio R., Leaci P., Scarascia R., Pulito G.

##### U.O. Anestesia e Rianimazione, PO Vito Fazzi ~ Lecce ~ Italia

###### **Correspondence:** Ciccarese A.

**Introduction**. Indications for ICP monitoring include diffuse brain injury, cerebral contusion, decompressive craniotomy and evacuation of supratentorial intracranial hematoma. The choice of preferring an initial non-invasive monitoring with serial measurements of the optic nerve sheat diameter (OSND, cut off 5.8-6 mm), at the entrance, after 3 hours and then every 6-8 hours, depends on the initial GCS (between 13 and 8), the age of the patient (non-invasive monitoring for age> 70 years), the first CT scan (in patients with GCS <13 and minimal or no signs of injury at the first CT scan, the ICP is initially monitored non-invasively with OSND and invasively only if serial measurements of OSND and the second CT scan show worsening of intracranial hypertension; immediate invasive monitoring in the presence of a diffuse lesion associated with signs of edema at the first CT evaluation; invasive monitoring for GCS <8, extensive frontal contusions and / or lesions close to the brain stem) and by the presence of extracranial trauma that contraindicates the suspension of sedation, (eg severe troracic trauma).

**Clinical case**. A 14-year-old boy, involved in a motorcycle-car accident, comes to our observation. Upon arrival: GCS 8 (E2, V1, M5), isochoric, isocyclic, normoreactive pupils. The first head CT scan is negative. OSND at the entrance: 5.6mm on the right (fig1), 5.8 mm on the left (fig2). The measurements three and six hours after the trauma remain unchanged, such as the neurological examination at the suspension of sedation. 24-hour CT scan of the skull, negative. Continued non-invasive monitoring of PIC with OSND: no change in trend indicating intracranial hypertension. On the third day, the patient performs MRI of the brain, which shows third degree axonal damage in the right parasagittal region, corpus callosum, midbrain, temporo mesial and in the left cerebellar peduncle. The patient on the fourth day was extubated and sent to a neurorehabilitation center on the fifteenth day with a GCS 12 (E4, V 2, M6).

**Discussion and Conclusions.** After a careful literature review on the indications for ICP monitoring and the levels of evidence, in our Institution, neurosurgeons and intensivists shared the decision to use the OSND trend as screening method for invasive method in the situations described above.

**Informed consent to publish had been obtained**

**Bibliography.**

1. Carney N, Totten AM, O'Reilly C, Ullman JS, Hawryluk GW, Bell MJ, Bratton SL, Chesnut R, Harris OA, Kissoon N, Rubiano AM, Shutter L, Tasker RC, Vavilala MS, Wilberger J, Wright DW, Ghajar J. Guidelines for the Management of Severe Traumatic Brain Injury, Fourth Edition. *Neurosurgery* 2017 Jan 1;80(1):6-15. doi: 10.1227/NEU.0000000000001432. PMID: 27654000.

2. Rasulo FA, Bertuetti R. Transcranial Doppler and Optic Nerve Sonography. *J Cardiothorac Vasc Anesth.* 2019 Aug;33 Suppl 1:S38-S52. doi: 10.1053/j.jvca.2019.03.040. PMID: 31279352.

3. Hansen HC, Helmke K. Optic nerve sheath responses to pressure variations. *Intensive Care Med*. 2019 Dec;45(12):1840-1841. doi: 10.1007/s00134-019-05746-3. Epub 2019 Aug 23. PMID: 31444504.

**Fig. 1 (abstract A143).**
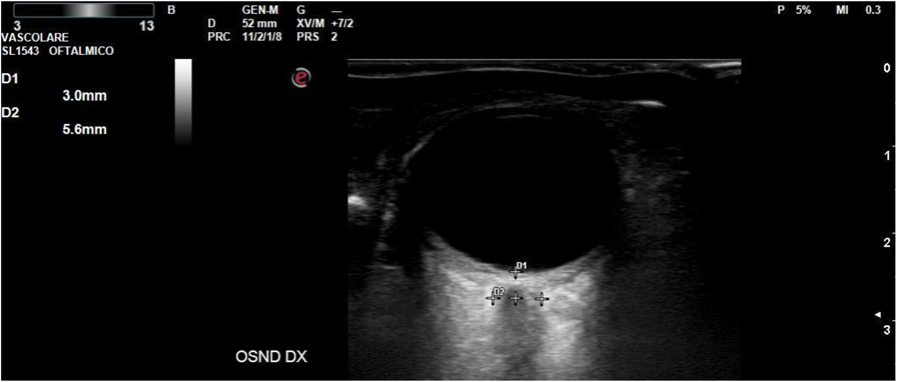
See text for description

**Fig. 2 (abstract A143).**
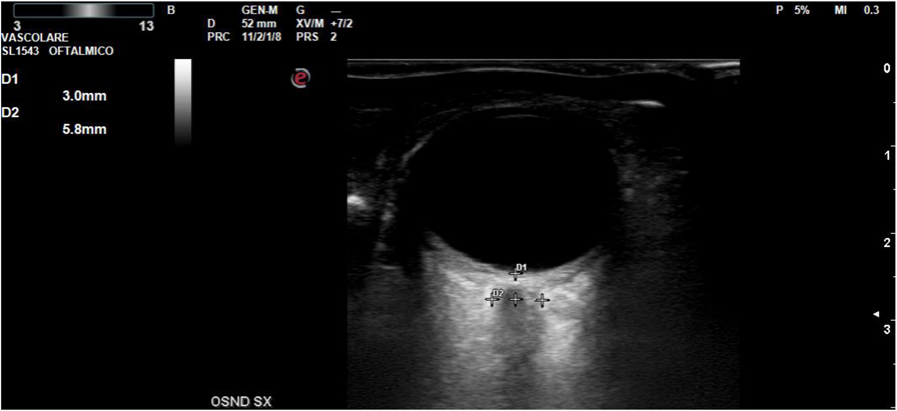
See text for description

### A144. MPLR evaluation in icu brain injured patients

#### Di Pierro F., Cotoia A., Mirabella L., Montrano L., Zimotti T.G., Napolitano L., Correnti D., Cinnella G.

##### Azienda Ospedaliera OO.RR. Foggia ~ Foggia ~ Italia

###### **Correspondence:** Di Pierro F.

BACKGROUND: Hematological indices are often used as prognostic factors in various diseases, but the role of Mean Platelet Volume to Lymphocyte ratio (MPLR) and Neutrophil to Lymphocyte ratio (NLR) in predicting brain injured patients’ outcome is still debated.

PURPOSE: In this study the MPLR and the NLR were evaluated to assess their prognostic role for brain injured patients’ outcome.

METHODS: From May 2021 to May 2022 consecutive brain injured patients admitted in the ICU were recruited. Hemogram, clinical history were recorded at admission. The MPLR and NLR were calculated. Patients were divided in two groups: non survivors in ICU (Group 1) and survivors (Group 0).

RESULTS: 48 patients were enrolled: mean age was 62±19 years, 29 patients (60%) were male. Diagnosis at admission was: Traumatic brain injury 25%, Intracranial hemorrhage 31%, Subarachnoid hemorrhage 17%, Epidural hematoma 2%, Subdural hematoma17%, Acute ischemic stroke 8%. 23 patients died during the hospitalization in the ICU (48%). Mean MPLR was 52 ± 39 in Group 0 vs 109 ± 90 in Group 1, (p=0.003). Mean NLR was 12 ± 8 in Group 0 vs 18 ± in Group 1, (p=0,137).

CONCLUSIONS: Hemogram should be evaluated in brain injured patients in ICU and low MPLR could be associated to a better outcome.

### A145. TEG Management of pulmonary thromboembolism after intracranial haemorrhage: a case report

#### Mastria D., Paiano G., Puscio D., Madaro F., Giaccari L.G., Pulito G.

##### U.O. Anestesia e Rianimazione, P.O. Vito Fazzi ~ Lecce ~ Italia

###### Correspondence: Madaro F.

**Introduction.** Patients with spontaneous intracerebral hemorrhage (ICH) had a stronger and faster clot formation than patients without ICH, demonstrating an activation of hemostasis. Venous thromboembolism is a common complication for patients suffering from a (ICH). TEG provides information regarding the hemostatic function in whole blood rather than measurements of single components of the clotting process such as PT, aPTT or platelet count. Some authors found that TEG values may identify an hypercoagulable state. In particular, they found that the association between maximal amplitude, a marker of platelet function and outcome was even stronger than traditional coagulation and inflammatory markers (D-dimer and fibrinogen), suggesting that maximal amplitude, is a more robust predictor of functional outcome than these other markers.

**Clinical case.** A 50-year-old patient came from a spoke center for cerebral left capsulo-thalamic haemorrhage during endoscopic polypectomy. At the entrance, GCS 10 (E2, V3, M5), right hemiplegia. After intubation, neuroprotection and VTE prophylaxis was initiated with elastic stockings and intermittent pressure therapy. Coagulation tests were normal (PT 75%, INR 1.14, PTT 25 sec, Fibrinogen 204); mild thrombocytopenia (PLT 83x10^3^). On thromboelastography, a pro-thrombotic state was shown, not detected by standard examinations (R 3.8 min, α angle 81^0^, MA 71 mm) (fig1-2). The patient started prophylactic dose LMWH therapy (6000IU) after 24 hours. On III day, for mild hypoxemia with stable hemodynamics, head and chest CT scan was performed, with evidence of massive pulmonary embolism. Blood tests showed increased D-dimers (78281), with no further alterations. Doppler of lower limbs: right popliteal thrombosis. Echocardiogram: PFO. Caval filter was placed, LMHW therapy was increased (6000IUx2). Thrombophilia tests showed MTHFR heterozygosity and hyperhomocysteinemia. The repeated TEG 72 hours after the start of anticoagulant therapy was normal (fig. 3-4). At the angio-CT after 15 days, complete resolution of the pulmonary embolism. The patient was sent to rehabilitation.

**Discussions and Conclusions.** Venous thromboembolism is a serious post ICH complication and most often it results in increased morbility and mortality; appropriate and detailed information on both patient-bound factors (malignancy, ICH, preexisting coagulopathies) and treatment-associated risk factors (type and length of surgery, postoperative immobilization) is needed to assess optimal antithrombotic prophylaxis for neurosurgical patients. POC-based identification of brain injury–induced alterations of hemostasis may provide important information regarding the patient’s risk profile for hemorrhagic or trombothic complications and unfavorable outcome. Therapeutic treatment options can be guided by some POC results, wich seems to be much more sensitive than standard coagulation tests, but further studies are needed to determine the impact of POC test results on treatment strategies in neurosurgery.

Informed consent to publish had been obtained

**Bibliography.**

1) Nyquist P et al. Prophylaxis of Venous Thrombosis in Neurocritical Care Patients: An Executive Summary of Evidence-Based Guidelines: A Statement for Healthcare Professionals From the Neurocritical Care Society and Society of Critical Care Medicine. *Crit Care Med.* 2017 Mar;45(3):476-479. doi: 10.1097/CCM.0000000000002247. PMID: 28085682.

2) Robba C et al. Coagulation management in patients undergoing neurosurgical procedures. *Curr Opin Anaesthesiol*. 2017 Oct;30(5):527-533. doi: 10.1097/ACO.0000000000000496. PMID: 28719459.

3) Yogendrakumar V et al. Venous thromboembolism prevention in intracerebral hemorrhage: A systematic review and network meta-analysis. *PLoS One*. 2020 Jun 24;15(6):e0234957. doi: 10.1371/journal.pone.0234957. PMID: 32579570; PMCID: PMC7314010.

**Fig. 1 (abstract A145).**
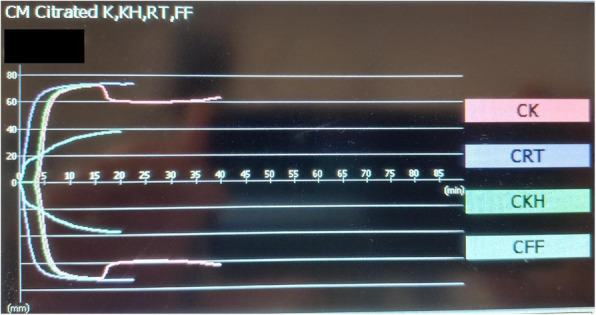
See text for description

**Fig. 2 (abstract A145).**
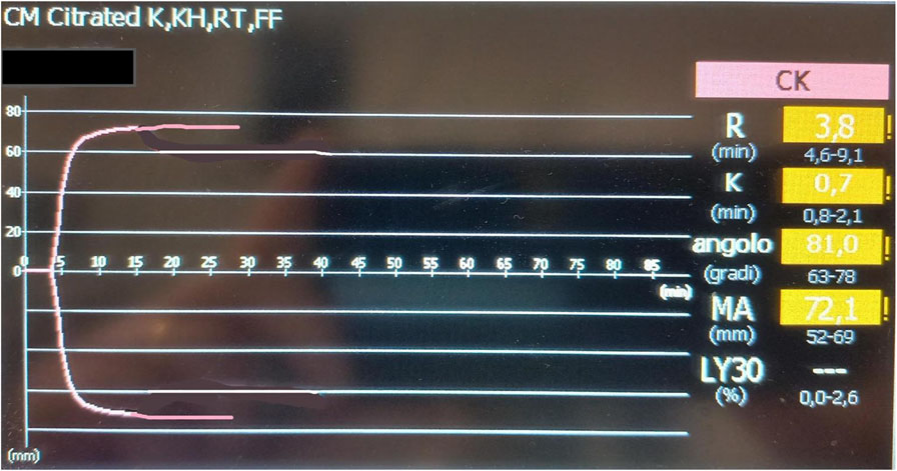
See text for description

**Fig. 3 (abstract A145).**
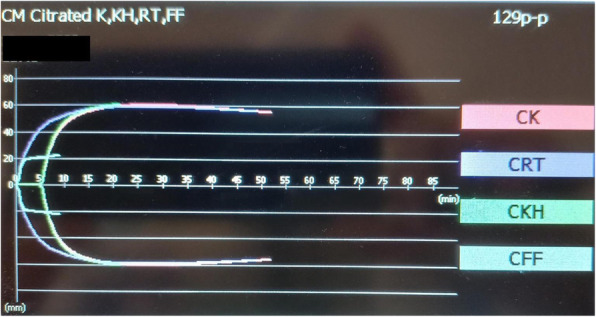
See text for description

**Fig. 4 (abstract A145).**
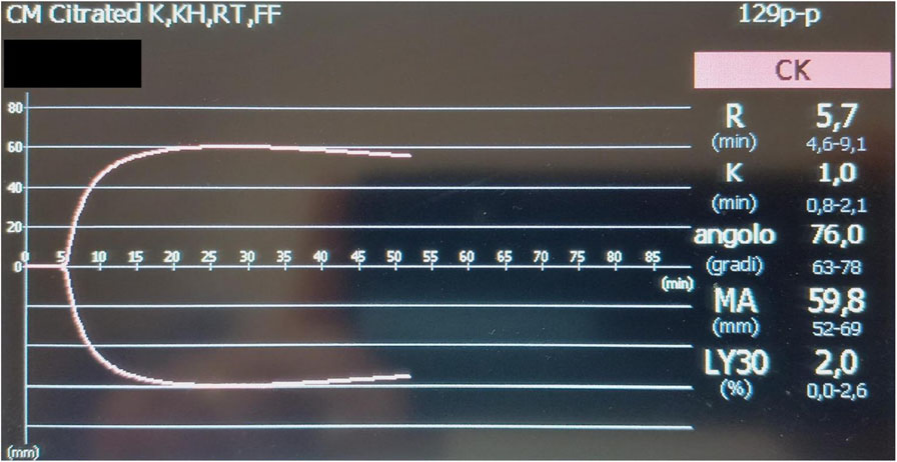
See text for description

### A146. Description of neurological alterations in patients with COVID-19 admitted to intensive care unit: an observational retrospective study

#### Casarotta E., Vitali E., Stanzione G., Antolini R., Pesaresi L., Camillo V., Salvucci Salice A., Giaccaglia P., Gorbi C., Marsili C., Mazzuca R., Marzialetti S., D”Angelo C., Donati A.

##### Università Politecnica delle Marche - Dipartimento di Scienze Biomediche ~ Ancona ~ Italy

###### **Correspondence:** Casarotta E.

**Introduction.** Neurological alterations are frequently observed in critically ill patients admitted to ICU. Numerous can be the risk factors involved, among which age, dementia, hypertension, mechanical ventilation, electrolyte, and acid-base disorders, hypoxemia, infections, and drugs. Patients with COVID-19 admitted to ICU are particularly at risk from systemic inflammation, hypoxemia, high risk of thrombosis, deep sedation, and prolonged mechanical ventilation. The present study aimed to describe the prevalence of neurological alterations in patients with COVID-19 admitted to ICU.

**Methods.** We conducted an observational retrospective single-center study. We considered patients with COVID-19 admitted to our ICU between March 2020 and May 2021. We collected demographic and clinical data. We noted all the neurological alterations reported in medical records. The psychomotor agitation was assessed using the RASS *(Richmond Agitation and Sedation Scale)* scale; the delirium using the CAM-ICU *(Confusion Assessment Method for the ICU).* We defined critical neuroworsening as the presence of either a decrease in pupillary reactivity or a new pupillary asymmetry or a spontaneous decrease in the GCS motor score ≥ 1 point, not explained by imaging.

**Results.** We considered 115 patients. Demographic characteristics of the study population are presented in *Table 1.* Among the comorbidities, no patients received a previous diagnosis of dementia. The prevalence of neurological alterations was 55,6 %. Of the 64 patients with neurological alterations, 50 (43,5 %) had psychomotor agitation or delirium, 17 (14,8 %) critical neuroworsening, and 9 (7,8 %) a worsening of the state of consciousness, not explainable by sedation levels. Comparing patients with neurological alterations and those without, we didn’t observe a significant difference in the average age (59,8 ± 10,7 years vs 61 ± 11,6 years, p = 0,60) and in the *Charlson Comorbidity Index* (2 [1-3] vs 2 [0,5-3], p = 0,99). Not even a significant difference was found in the number of hypoxia episodes (4 [1-12] episodes in patients with neurological alterations vs 2 [0-7] episodes in those without neurological alterations, p = 0,11) and the number of hypotensive episodes (0 [0-3] episodes in patients with neurological alterations vs 0 [0-2], p = 0,19) between the groups of patients during ICU stay. We found a significant difference in the number of days of mechanical ventilation between the two groups (24 [13-42] days in patients with neurological alterations vs 12,5 [7-25] in those without neurological alterations, p < 0,01). We also observed a significant difference in the number of days of treatment with opioids (21 [15-29] days in patients with neurological alterations vs 14 [7,5-23,5] days in those without, p = 0,01).

**Conclusions.** In this preliminary analysis, in patients with COVID-19 admitted to ICU psychomotor agitation episodes, delirium, and neuroworsening has been observed with high frequency. In patients with these neurological alterations, the number of days of mechanical ventilation and treatment with opioids has been significantly higher.

**Table 1 (abstract A146).** See text for description

**Height, cm**	174,3 ± 9,2
**BMI, kg/m2**	29,3 [26,3-34,1]
**Comorbidities, n (%)**	
**Arterial Hypertension**	50 (45.4)
**Obesity**	48 (43,6)
**Type II diabetes**	21 (19,1)
**Cerebral vasculopathy**	3 (2,7)
**Ischemic heart disease**	6 (5,4)
**Chronic Kidney Disease**	2 (1, 8)
**Charlson Comorbidity Index**	1 [2-3]

Data are presented as absolute and percentage frequencies, mean ± standard deviation, median [interquartile range]

*BMI* Body Mass Index

### A147. Neuroprotective role of dexmedetomidine during sedation of neonates with hypoxic-ischemic encephalopathy subjected to NMR

#### Pizzo M.^1^, Wielecka D.^1^, Tinturini R.^2^, Cesare V.^2^, Scolletta S.^3^, Tarantino F.^2^

##### ^1^Dipartimento Di Scienze Mediche, Chirurgiche E Neuroscienze ~ Siena ~ Italy, ^2^Azienda Ospedaliera Universitaria Senese ~ Siena ~ Italy, ^[3]^Dipartimento di Scienze Mediche, Chirurgiche e Neuroscienze; Azienda Ospedaliera Universitaria Senese. ~ SIENA ~ Italy

###### **Correspondence:** Pizzo M.

Background: Hypoxic-ischemic encephalopathy (HIE) is the leading cause of perinatal mortality and subsequent short and long term disability. Today the golden standard treatment is brain hypothermia, which limits tissue damage through a temporary brain hypoperfusion which reduces the vasogenic edema and the release of excitatory neurotransmitters. In this context, the use of dexmedetomidine as sedativ/hypnotic drug can contribute to the global neuroprotective strategy in neonates with HIE thanks to its neuroprotective, synaptoplastic and neurogenic effects.

Materials and methods: A pilot study was conducted to evaluate the neuroprotective role of anaesthetic drugs used in HIE neonates post-hypothermia during sedation in NMR in relation to Arterial Spin Labeling (ASL) imaging sequences. 24 neonates with Sarnat II/III HIE were recruited and subjected to therapeutic hypothermia for 72 hours and subsequently (between day 4 and day 10 post-hypothermia) to NMR diagnostic exam with sedation. Patients were divided into two groups based on the type of sedation received: Dex neonates were sedated with 3-4 y/kg intranasal dexmedetomidine maintained with sevoflurane, Non-Dex neonates were sedated with midazolan or fentanest and sevoflurane. Tissue perfusion in different brain regions was examined using ASL during NMR. To evaluate the overall outcome, vital signs during sedation, duration of hospitalization in neonatal intensive care unit (NICU) and neurological exam before discharge were also analyzed.

Results: Cerebral blood flow was significantly higher in basal ganglia (p<0,008) and right thalamus (p<0,0002) of all neonates in the Non-Dex group to the Dex group. Cortical perfusion was increased in the Non-Dex group, although the result was not statistically signifiant (occipital cortex (p<0,6), left-frontal cortex (p<0,14), right-frontal cortex (p<0.08), right-temporal (p<0.19)). No statistically significant differences were observed in duration of hospitalization in NICU and neurological exam before discharge.

Conclusions: The present study evaluated the neuroprotective effects of dexmedetomidine during NMR sedation in HIE neonates, which resulted in a reduced perfusion of ischemic brain regions and consequelty in a reduced risk of hemorrage and tissue damage, two critical aspects for survival and long term disability. Further studies on a larger population are needed to confirm the results observed in this pilot-study.

## Nuove tecnologie di diagnosi e cura

### A148. An application of the three pillars of digital transformation: the microcirculation network research group

#### Montomoli J.^1^, Giaccaglia P.^3^, Carsetti A.^3^, Donati A.^3^, Hilty M.P.^2^, Ince C.^4^

##### ^1^Department of Anesthesia and Intensive Care, Infermi Hospital, AUSL Romagna ~ Rimini ~ Italia, ^2^Institute of Intensive Care Medicine, University Hospital of Zurich, Switzerland ~ Zurich ~ Switzerland, ^3^Anesthesia and Intensive Care Unit, Azienda Ospedaliero Universitaria Ospedali Riuniti, Ancona, Italy ~ Ancona ~ Italia, ^4^Department of Intensive Care, Erasmus MC, University Medical Center, Rotterdam, the Netherlands ~ Rotterdam ~ Netherlands

###### **Correspondence:** Montomoli J.

The digital transformation is usually considered all about development and acquisition of new technologies to add on the top of the existing infrastructure. On the contrary, it should be considered as an edification of a completely new building where the weight-bearing structure consists of three pillars (Figure). The first pillar consists of the technological organization with an appropriate infrastructure. The second pillar is represented by data quality and quantity acquired by a process fully digitized able to produce reliable data. The third pillar promotes a digital culture and is supported by people able to lead the processes and leverage the results. In the recent years, the assessment of mucosal microcirculation with handheld vital microscopy has been subjected to technological improvements that has opened the path towards its use at the bedside as a promising tool to evaluate tissue oxygenation on a microcirculatory level and guide therapeutic decisions. In particular, in 2019, the first validated software for automatic analysis of images of the microcirculation (MicroTools) was released by our group.1 Before then, videos needed to be analyzed manually by trained operators in a time-consuming process, therefore, not feasible at the bedside. Driven by the desire to be actively part of the digital transformation and moved by the passion for microcirculation monitoring we tried to apply the “three pillars of digital transformation” in the Microcirculation Network Research Group (MNRG). The MNRG has then created the first pillar consisting of an online infrastructure offering free of charge analyses upon the exchange, together with the videos, of fully anonymized clinical information regulated by a collaboration agreement signed by the parties. Centers willing to join the MNRG may apply filling an online form at https://sites.google.com/microcirculation.network/home. All information obtained were then stored in a relational database with limited and regulated access, including quality gates. The entire process warrants a continuously growing dataset with complete information and containing data standardized and of high-quality fulfilling the requirement of pillar two. Finally, the MNRG has the mission to promote a new culture in the field of microcirculation, teaching and supervising researchers and institutions interested in the microcirculation monitoring. Such mission, belonging to pillar three, was realized through the creation of the microcirculation academy (https://www.microcirculationacademy.org/). Nowadays, the MNRG is actively collaborating with 23 centers in 11 countries on 26 different projects and it is continuously expanding. The MNRG strategy consists on short-term approaches (“early wins”) with simple but impactful applications and long-term programs with the more ambitious task of finally bring the technology of microcirculation monitoring at the bedside with defined and validated clinical applications able to guide therapeutic decision with a significant impact on patient prognosis.

References

1. Hilty MP, Guerci P, Ince Y, Toraman F, Ince C. MicroTools enables automated quantification of capillary density and red blood cell velocity in handheld vital microscopy. Communications Biology [Internet]. 2019 Dec [cited 2019 Aug 18];2(1). Available from: http://www.nature.com/articles/s42003-019-0473-8

**Fig. 1 (abstract A148).**
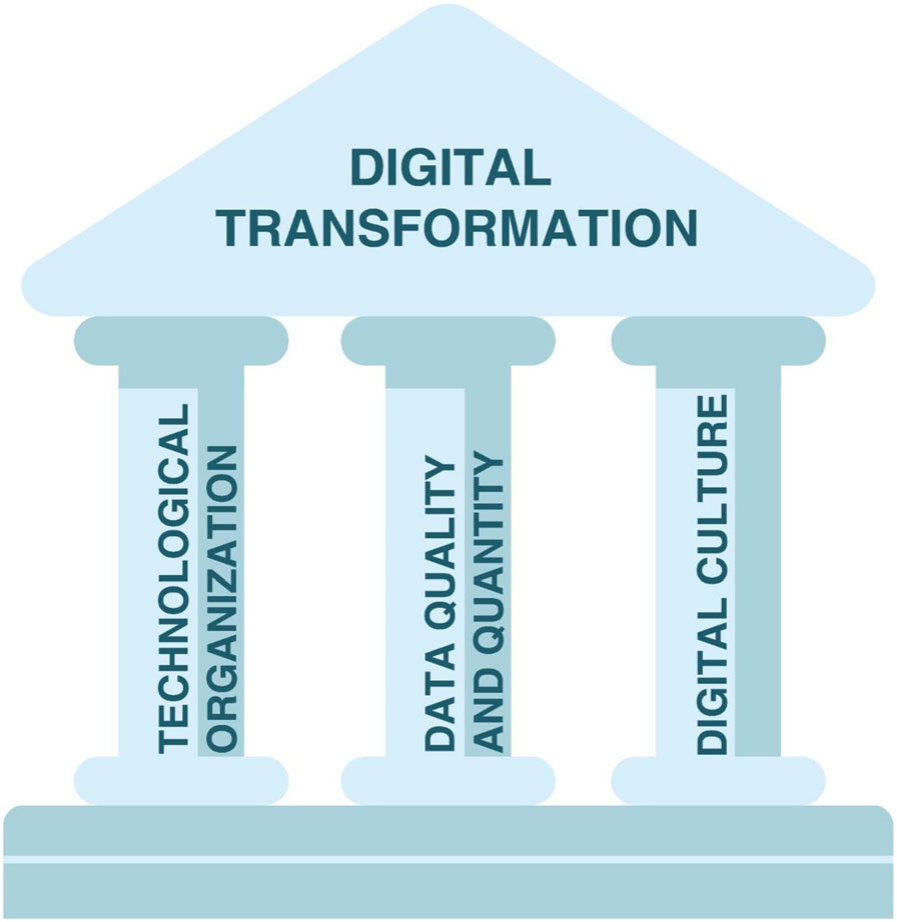
See text for description

### A149. Gene silencing of endothelial Von Willebrand factor hampering SARS-COV-2 infection of human endothelial cells as an innovative way to improve perioperative outcome in long covid patients

#### Furini G.^2^, Fonnesu R.^1^, De Carli A.^1^, Pistello M.^1^, Lai M.^1^, Lionetti V.^2^

##### ^1^Retrovirus Center, Department of Translational Research and New Technologies in Medicine and Surgery, University of Pisa ~ Pisa ~ Italia, ^2^Unit of Translational Critical Care Medicine, Scuola Superiore Sant'Anna ~ Pisa ~ Italia

###### **Correspondence:** Furini G.

Background: A non-negligible number of patients develop up to 15 month after coronavirus disease 2019 (COVID-19) a significant endothelial dysfunction that is accompanied by a residual risk for perioperative cardiovascular complications due to suboptimal tissue oxygen delivery. Severe acute respiratory syndrome coronavirus 2 (SARS CoV 2) persistence has been proposed to be associated with post-infectious disorders. Since augmented levels of endothelial von Willebrand Factor (vWF) lead to endotheliopathy and persist even longer in long COVID patients, we hypothesized that targeting endothelial vWF with short interference RNA (siRNA) prevents severe acute respiratory syndrome coronavirus 2 (SARS CoV 2) spreading to endothelial cells through angiotensin converting enzyme 2 (ACE2).

Method: Gene silencing of vWF or ACE2 was performed in human umbilical vein endothelial cells (HUVEC) by RNA interference (siRNA), while vWF overexpression was obtained by transient pcDNA3.1-WT-VWF plasmid transfection of HUVEC. QRT-PCR evaluated expression levels of ACE2 and vWF in endothelial cells. Immunofluorescent labeling detected changes of surface localization of both endothelial proteins. Wild type and transfected endothelial cells were incubated with SARS-CoV-2 for one hour, and the level of cell infection was assessed by qRT-PCR of viral RNA after 24 / 48 hours.

Results: Nearly 90 ± 2% (p<0.0001) silencing of vWF in viable HUVEC was achieved with vWF-specific siRNA and we observed a significant reduction of SARS-CoV-2 infection of vWF-silenced cells (-56% SARS-CoV-2 RNA, p=0.0058). Interestingly, surface vWF knockdown associated with a significant downregulation of ACE2 expression on the same cells (-55% mRNA expression, p=0.0032). Conversely, vWF overexpression (+130-fold, p=7.4 ·10-7) determined a significant upregulation of ACE2 (+24-fold p=0.028) and increased susceptibility of HUVEC to SARS-CoV-2 infection (+18-fold, p=0.0009).

Conclusion: Endothelial surface vWF knockdown hampered SARS-CoV-2 spreading to endothelial cells through downregulation of surface ACE2 expression. Our findings reveal a hitherto unsuspected role of vWF in preventing of endothelial SARS-CoV-2 infection and might be helpful to design new therapeutic strategy to improve perioperative tissue oxygen delivery in long COVID patients. This is particularly attractive in light of the increasing number of viral mutants that may persist in vessels of different vital tissues.

### A150. Use of photoplethysmographic analysis to detect microcirculatory alterations: COVID-19 detection and stratification

#### Luchini M.^1^, Rossi E.^2^, Aliani C.^2^, Deodati R.^1^, Calamai I.^1^, Spina R.^1^, Bocchi L.^2^

##### ^1^Ospedale san Giuseppe ~ Empoli ~ Italia, ^2^Dipartimento di ingegneria informatica- Università di Firenze ~ Firenze ~ Italia

###### **Correspondence:** Luchini M.

Objective: Microvascular dysfunction has been associated with adverse outcomes in critically ill patient. Despite technological advances in this field, the direct identification of severe microcirculatory alterations remains difficult at bedside. In Coronavirus disease 2019 endothelial damage is the underlying mechanism that links inflammation and thrombosis. The endothelial alteration can lead to important consequences such as microvascular anomalies which in these patient can be important because they are related to the severity of the disease and to the prognosis in the acute phase. Photoplethysmography (PPG) technique is a non-invasive, low cost and user-friendly method and was used in order to carry out the evaluation of microcirculation. The aim of the study was to define a mathematical model representing the microvascular flow starting from a PPG signal in order to detect alterations in patients with COVID-19.

Approach: The photoplethysmographic signal was registered and evaluated in 93 patients with COVID-19 of different severity, classified in relation to the types of ventilatory supports they need, (46: grade 1; 47 grade 2) and in 50 healthy control subjects. In the acquisition process, laboratory parameters were also detected, which are validated , already in the previous literature and clinical activity, as indices of disease severity and microcirculation alteration, such as IL-6 and D-dimer. Each pulse is approximated with a model generated from a multiexponential curve, and a Least Squares fitting algorithm determines the optimal model parameters. Using the parameters of the mathematical model, three different classifiers (Bayesian, SVM and KNN) were trained and tested to discriminate among healthy controls and patients with COVID.

Main results: D-dimer and IL-6 correlate directly with the severity of the disease and the division into classes, indirectly validating the mathematical and classification model applied to the photoplethysmographic wave. The fitting and modelling procedure identifies the optimal parameter set. Five features remain in common between all strategy classification: A3, k1,1, , k2,1 k2,2 and t3. Among these parameters the presence of A3 and k1,1, the risin slope of the systolic curve, could confirm that Covid-19 affects heart, varying its pumping force and velocity of contraction. Furthermore, the presence of two of four parameters releted to the reflected wave could confirm the presence of microvascular abnormalities. The Bayesian classifier obtains promising results, given the size of the dataset, with a 79% of accuracy, 72% of specificity and 87% of sensibility.

Significance: The proposed approach confirms that photoplethysmographic can study microcirculation. This can be applied to open the possibility of introducing a low cost and non invasive screening procedure for the fast detection of COVID-19 disease. Other fields of application for the study of microcirculation are currently under examination.

Fig 1-2: P, models the systolic wave; P. models the diastolic wave; P3 models the reflected wave.

Main results: D-dimer and IL-6 correlate directly with the severity of the disease and the division into classes, indirectly validating the mathematical and classification model applied to the photoplethysmographic wave. The fitting and modelling procedure identifies the optimal parameter set. Five features remain in common between all strategy classification: 43, K1,1,, k21 ka and #3.

Among these parameters the presence of 43 and X,1, the risin slope of the systolic curve, could confirm that Covid-19 affects heart, varying its pumping force and velocity ofcontraction.

Furthermore, the presence of two of four parameters releted to the reflected wave could confirm the presence of microvascular abnormalities. The Bayesian classifier obtains promising results, given the size of the dataset, with a 79% of accuracy, 72% of specificity and 87% of sensibility.

Significance: The proposed approach confirms that photoplethysmographic can study microcirculation.

This can be applied to open the possibility of introducing a low cost and non invasive screening procedure for the fast detection of COVID-19 disease. Other fields of application for the study of microcirculation are currently under examination.

**Fig. 1 (abstract A150).**
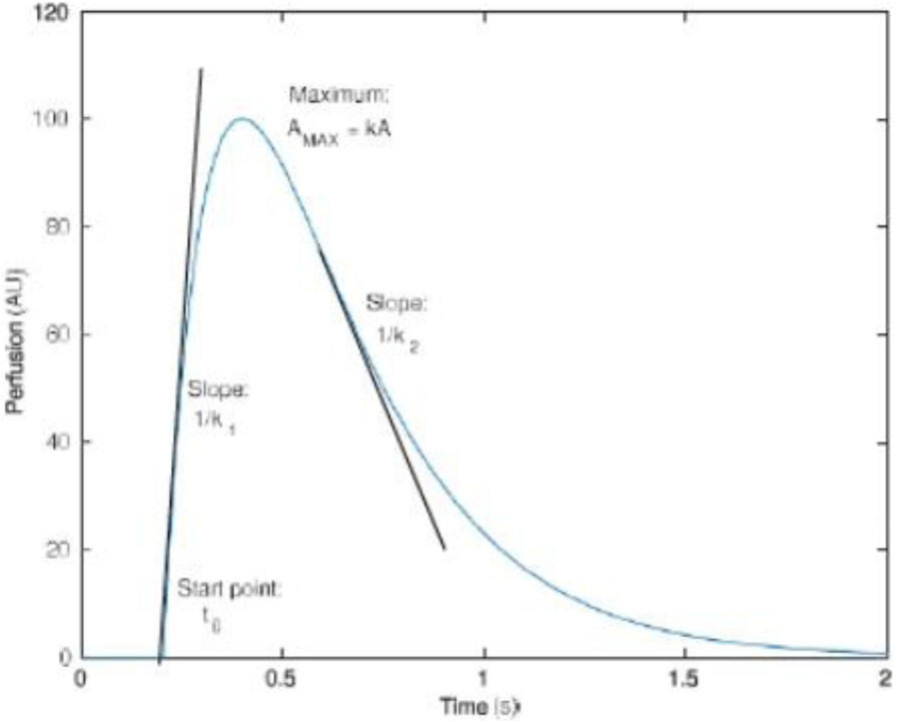
Exponential pulse with graphic representation of the parameters.

**Fig. 2 (abstract A150).**
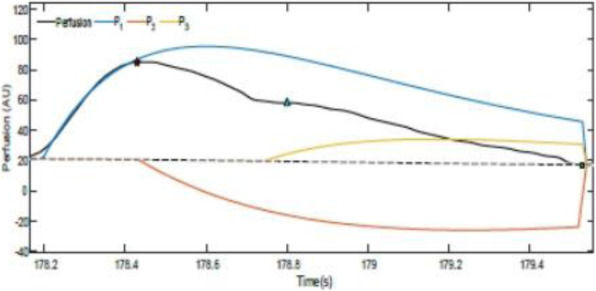
Sample model curve, generated by a proper combination of three exponential pulses.

### A151. Is Nephrocheck® the new troponine I for AKI?

#### Ferrari C.^1^, Mancusi Materi G.^1^, Laterza C.^1^, Prota L.^1^, Sforza A.^1^, Antonelli P.^1^, Perrini M.^1^, Mangini M.^1^, Maringelli G.^1^, Saponaro A.M.^2^, Ribezzi M.^1^, Brienza N.^1^

##### ^1^Unit of Anesthesia and Resuscitation, University of Bari Aldo Moro ~ Bari ~ Italia, ^2^Unit of anesthestesia and resuscitation "L.Bonomo" Hospital ~ Andria (BT) ~ Italia

###### **Correspondence:** Ferrari C.

Background

Acute kidney injury (AKI) occurs in more than 50% of critically ill patients, steadily increasing over the past decades, and turning into a challenge for healthcares worldwide. AKI definition and early identification presents several issues, mostly related to creatinine kinetic and how it is altered during critical illness. The "renal troponin I" may be found in the novel urinary biomarkers insulin-like growth factor-binding-protein 7 (IGFBP7) and tissue inhibitor of metalloproteinases-2 (TIMP-2).This is an observational study designed to assess the capability of [TIMP-2]•[IGFBP-7] and its variation to predict AKI occurrence with the aim of introducing preventive or therapeutic interventions.

Material and methods

This observational longitudinal prospective study included patients admitted to our Intensive Care Unit at “Policlinico” Hospital in Bari. Subjects were included if at least 18 years of age and presented known renal risk factors, including major surgery, shock, sepsis, cardiac pathology, trauma, exposure to nephrotoxic drugs. Exclusion criteria were: age <18 years, documented chronic renal failure (stage >3) or already requiring renal replacement therapy, any AKI stage at the admission, monorenal subjects or admitted after nephrectomy, post organ transplantation, cardiac surgery, ICU stay shorter than 24 hours, recent (<90 days) admission to ICU. For each enrolled patient, two determinations of urinary [TIMP-2]x[IGFBP-7] were performed: the baseline at the admission (T0), and the other after twelve hours (T12). Blood samples for serum creatinine measurement were obtained with the same timing. The markers have been measured using the NephroCheck® test (Astute 140 meter - Astute Medical, San Diego, CA, USA), and expressed in (ng/ml)2/1000. The primary endpoint was a difference in NephroCheck levels at T0 and T12 in patients with and without AKI. Secondary endpoints included sensitivity/ specificity analyses of previously proposed cut-off levels and clinical outcome measures.

Ethical approval for case studies: the present study was based in University of Bari (Italy), in full accordance with ethical principles, including the World Medical Association Declaration of Helsinki and the additional requirements of Italian law. Furthermore, the University of Bari, Italy, classified the study to be exempt from ethical review as it carries only negligible risk and involves the use of existing data that contains only non-identifiable data about human beings.

Results

56 patients were included. AKI was observed in 46.43% (26/56) of patients. Urinary (TIMP-2) x (IGFBP7) levels at T0 significantly differ between patients with and without AKI (mean 0.159+0,220 and mean 0.636+0,986) and also at T12 (mean 0.351+0,388 and mean 1.141+2,060). Serum creatinine was significantly different between AKI and non AKI patients just at T12. Previously proposed cutoff levels (0.3 and 2) showed at any time moderate sensitivity (0,53/0,12 at T0 0,62/0,12 at T12 ) but high specificity (0,87/1 at T0 and 0,6/1 at T12 ) for 0,3 and 2 respectively . Eight patients who developed AKI died during the hospital stay, while all the non-AKI patients survived.

Conclusion

Early assessment of (TIMP-2) x (IGFBP7) urinary in ICU patients can predict the development of renal damage. Due to its high specificity, all ICU patients with (TIMP-2)x(IGFBP7) elevation must not be regarded as inconsequential and a tailored prevention treatment should be performed as soon as possible.

### A152. Intravenous infusion of mesenchymal stem cells for adjuvant treatment of a 44-year-old critically ill patient with COVID 19 presenting lung fibrosis: a case report and 6-months follow up

#### Tani C., Venturi L., Bichi L., Calamai I., Giuntini R., Spina R.

##### ASL Toscana Centro, S.O.C Anestesia e Rianimazione Ospedale “San Giuseppe” ~ Empoli (FI) ~ Italia

###### **Correspondence:** Venturi L.

COVID-19 has become a global public health emergency since patients were first detected in Wuhan, China in December 2019.

Patients with severe/critical COVID-19 usually present a hyperinflammatory state characterized by cytokine storm up to acute respiratory distress syndrome (ARDS) with multi-organ failure and death. In patients who survive ARDS, fibrotic outcomes may persist, with the development of diffuse pulmonary fibrosis.

Despite significant therapeutic advances with greater knowledge and definition of standard protocols, critical COVID-19 remains a life-threatening disease and new therapeutic strategies are urgently needed.

Mesenchymal stromal cells (MSCs), thanks to their potent immunomodulatory capacity, may have beneficial effects on the prevention or mitigation of cytokine storm.

They have been shown to migrate to damaged tissues, exert anti-inflammatory and immunoregulatory functions, promote the regeneration of damaged tissues and inhibit tissue fibrosis.

This is a case report of a 44-year-old female patient with Covid-19 under invasive mechanical ventilation for 75 days in the intensive care unit (ICU), who presented progressive clinical deterioration associated with lung fibrosis. No indication for ECMO support and failure of Novalung support. The patient was treated with bone marrow derived MSCs [1x10^6^ MSC/Kg (2 doses 17 days interval)]. 15 days after the second MSC administration we observed a progressive improvement in gas exchange (Fig.1), Static Compliance (Fig. 2) as well as an increased white blood cell count (decreased neutrophils and increased lymphocytes) (Fig. 3). Radiologic picture significantly improved, too (Fig. 4).

The patient was discharged from the ICU 22 days after the treatment, 93 days from admission.

The patient is currently followed up in our follow-up clinic. Residual disability was assessed using the Glasgow Outcome Scale (GOS) at 3 and 6 months after discharge, identifying a value of 4 and 5 respectively.

Despite a chest CT image showing the presence of pulmonary fibrosis at 3 and 6 months, the patient's clinical evaluation showed mild wheezing and dyspnoea under exertion with Modified British Medical Research Council Questionnaire (mMRC) 1.

Respiratory function tests at 6-months follow-up showed a mild restrictive defect with normal capillary alveolar diffusion.

The regenerative and anti-inflammatory abilities of mesenchymal stem cells can be an innovative approach in repairing damaged organs that improve recovery times and survival rates for critically ill COVID-19 patients.

Informed consent to publish had been obtained.

**Bibliography**

**Mesenchymal stem cells and COVID-19: What they do and what they can do**.Abu-El-Rub E, Khasawneh RR, Almahasneh F, Altaany Z, Bataineh N, Zegallai H, Sekaran S.World J Stem Cells. 2021 Sep 26;13(9):1318-1337. doi: 10.4252/wjsc.v13.i9.1318.

**Stem cell therapy for COVID-19, ARDS and pulmonary fibrosis.** Li Z, Niu S, Guo B, Gao T, Wang L, Wang Y, Wang L, Tan Y, Wu J, Hao J.Cell Prolif. 2020 Dec;53(12):e12939. doi: 10.1111/cpr.12939. Epub 2020 Oct 24.

**Mesenchymal Stem Cell Therapy for COVID-19: Present or Future** Golchin A, Seyedjafari E, Ardeshirylajimi A.Stem Cell Rev Rep. 2020 Jun;16(3):427-433. doi: 10.1007/s12015-020-09973-w.

**Immunomodulatory and Anti-fibrotic Effects Following the Infusion of Umbilical Cord Mesenchymal Stromal Cells in a Critically III Patient With COVID-19 Presenting Lung Fibrosis; A Case Report** Silva KN, Pinheiro PCG, Gobatto ALN, Passos RDH, Paredes BD, França LSA, Nonaka CKV, Barreto-Duarte B, Araújo-Pereira M, Tibúrcio R, Cruz FF, Martins GLS, Andrade BB, de Castro-Faria-Neto HC, Rocco PRM, Souza BSF.Front Med (Lausanne). 2021 Nov 17;8:767291. doi: 10.3389/fmed.2021.767291.

**Fig. 1 (abstract A152).**
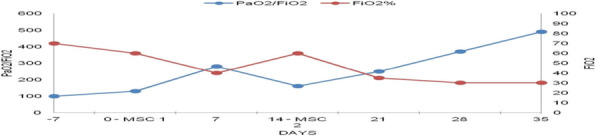
See text for description

**Fig. 2 (abstract A152).**
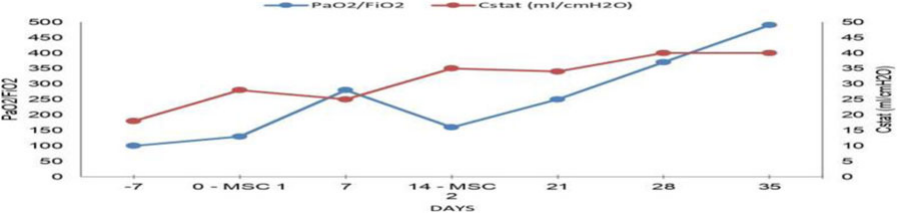
See text for description

**Fig. 3 (abstract A152).**
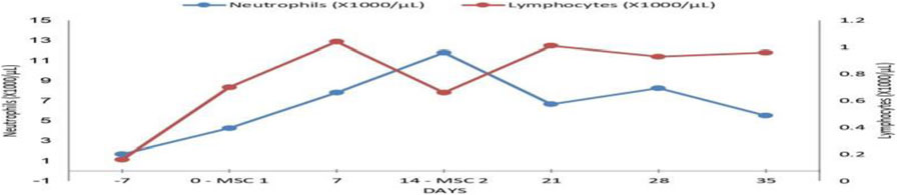
See text for description

**Fig. 4 (abstract A152).**
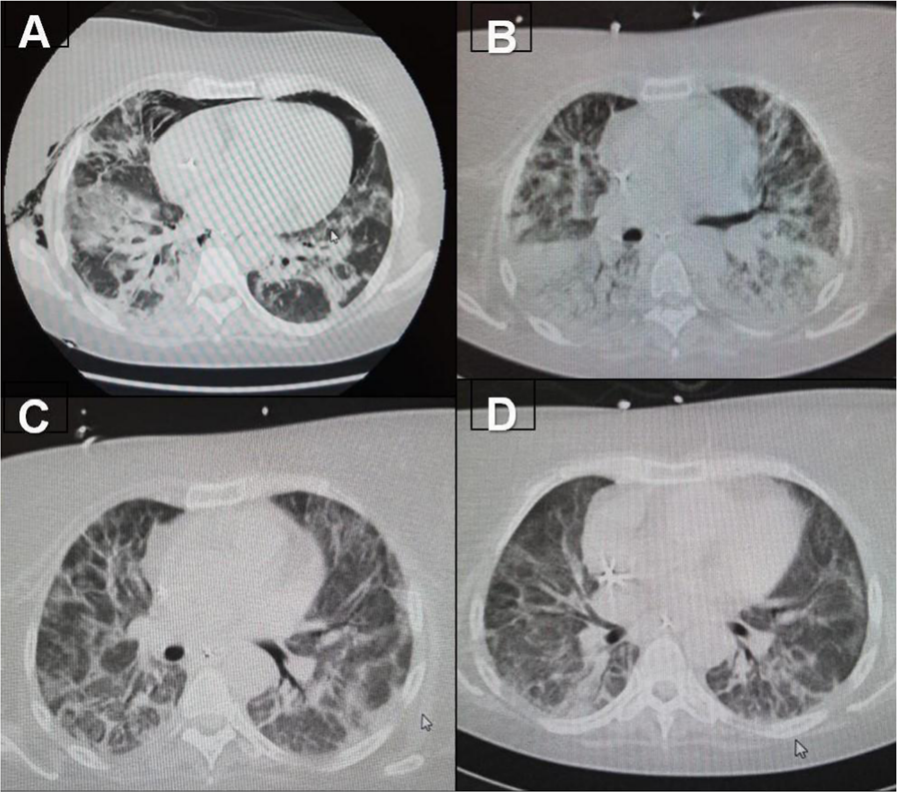
CT scan images. A. ICU-admission; B. before MSC treatment; C. After MSC administration; D. ICU-discharge

### A153. Evaluation by medical staff of electronic health records in use in italian intensive care units: a national survey

#### Saglietti F.^1^, Montomoli J.^2^, Vichi B.^3^, Gottardi B.^4^, Bergamini C.^5^, Gamberini E.^2^, Bignami E.G.^6^, Agnoletti V.^5^

##### ^1^Department of Anaesthesia and Intensive Care, Bassini Hospital, ASST Nord Milano, Cinisello Balsamo, Italia ~ Milano ~ Italy, ^2^Department of Anaesthesia and Intensive Care, Infermi Hospital, Rimini, Italy ~ Rimini ~ Italy, ^3^Department of Medical and Surgical Sciences, Alma Mater Studiorum - University of Bologna, Bologna, Italy ~ Bologna ~ Italy, ^4^Dipartimento di Medicina Traslazionale e per la Romagna, University Hospital of Arcispedale Sant'Anna, University of Ferrara, Ferrara, Italy ~ Ferrara ~ Italy, ^5^Department of Anaesthesia and Intensive Care, Bufalini Hospital, AUSL Romagna, Cesena, Italy ~ Cesena ~ Italy, ^6^Anesthesiology, Critical Care and Pain Medicine Division, Department of Medicine and Surgery, University of Parma, Parma, Italy ~ Parma ~ Italy

###### **Correspondence:** Saglietti F.

Introduction

Adoption of electronic health records (EHRs) is increasing worldwide in the Intensive Care Units (ICUs). Great expectations have accompanied the advent of EHRs in terms of ability in adquiring, interfacing and processing data from various monitoring devices used in the ICUs. EHR carried the expectation to provide clinical decision support systems able to assist clinicians in the process leading to diagnostic-therapeutic decision. However, some studies have shown that these expectations have been disregarded [1]. There is no national data regarding the evaluation of EHR by Italian intensivits. We, therefore, carried out a survey addressed to Italian intensivists, aimed at investigating the degree of satisfaction regarding EHRs and some specific technical features.

Materials and methods

The survey was carried out on a voluntary basis using a specific form that could be filled online at the link: https://forms.gle/x6N4ErTsseiDWR5j7. The survey was launched on the 17th of May 2022 and widespread via the major social media, instant messaging, emails, and text messages. The analysis in the present study includes data collected to the 27th of May 2022. The responses of both consultants and residents were extracted. The overall evaluation of the experience with the EHR was expressed with a score from 1 ("very bad") to 10 ("excellent") while the specific characteristics with a score from 1 ("not at all satisfied") to 5 ("fully satisfied").

Results

Of 817 survey forms completed in the timespan, 644 (78.8%) questionnaires were obtained from doctors (61% consultants, 39% residents) with 58.4% of the participants working in a university hospital and 54.1% in an ICU adopting an EHR. Among doctors who do not use an EHR, 97.6% would like to have one and 54.6% know at least one type of EHR. The overall evaluation of EHRs was positive for both consultants and residents (Figure 1A and 1B). Differently, for all 9 specific functionalities investigated, the average satisfaction was lower than 3 out of 5 (Figure 1C). Differences in overall assessment were also found between the different types of EHR in use in ICU with an average score that varied between a maximum of 8.0 (SD = 1.4) and a minimum of 5.6 (SD = 2.2).

Conclusions

Despite of the evaluation of EHR in use in Italian ICUs by intensivits is overall positive, EHRs still present major gaps in regard of management and accessibility of data acquired during the patient's hospitalization.

References

1. Melnick ER, Sinsky CA, Krumholz HM. Implementing Measurement Science for Electronic Health Record Use. JAMA [Internet]. 2021 Apr 5 [cited 2021 May 13]; Available from: https://jamanetwork.com/journals/jama/fullarticle/2778440

**Fig. 1 (abstract A153).**
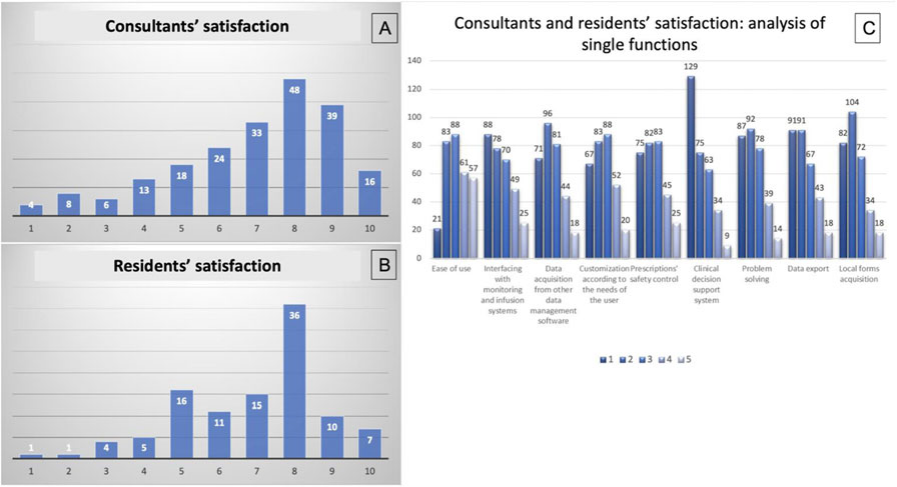
See text for description

### A154. Nursing evaluation of electronic health record in the Italian intensive care units

#### Gottardi B.^1^, Montomoli J.^2^, Saglietti F.^3^, Vichi B.^4^, Di Giandomenico S.^2^, Dominici S.^2^, Baschetti E.^2^, Scaramuzzo G.^1^, Ragazzi R.^1^, Bignami E.G.^5^, Agnoletti V.^6^, Gamberini E.^2^

##### ^1^Department of Translational Medicine for Romagna, University Hospital Arcispedale Sant'Anna, University of Ferrara, Ferrara, Italia ~ Ferrara ~ Italy, ^2^Department of Anaesthesia and Intensive Care, Infermi Hospital, Rimini, Italy ~ Rimini ~ Italy, ^3^Department of Anaesthesia and Intensive Care, Bassini Hospital, ASST Nord Milano, Cinisello Balsamo, Italia ~ Milano ~ Italy, ^4^Department of Medical and Surgical Sciences, Alma Mater Studiorum - University of Bologna, Bologna, Italia ~ Bologna ~ Italy, ^5^Department of Anaesthesia and Intensive Care, Maggiore Hospital, AOU di Parma, Parma, Italia ~ Parma ~ Italy, ^6^Department of Anaesthesia and Intensive Care, Bufalini Hospital, AUSL Romagna, Cesena, Italia ~ Cesena ~ Italy

###### **Correspondence:** Gottardi B.

**Introduction**

Electronic Health Record (EHR) is a supporting tool for assistance activities that spread in Intensive Care Units (ICUs) in the last few years. EHRs substantially modified the work routine of both medical and nursing staff in order to improve patient care and to simplify the management of data flow. However, at the present time there is no survey analyzing the nursing staff satisfaction about EHRs. We, therefore, conducted a national survey with the aim of describing the nursing staff opinion on ease of use and performance of EHR.

**Materials and methods**

The survey was disseminated electronically (https://forms.gle/biwBAAQMGMPjYeXA6) through social media and web channels from May 17^th^ to 27^th^ 2022. In the present survey, the compilations by nurses working in Italian ICU have been taking into account. The overall assessment of the experience with EHR was expressed with a score from 1 (“very bad”) to 10 (“very good”), while the specific characteristics were evaluated with a score from 1 (“not at all satisfied”) to 5 (“fully satisfied”). Ethical approval was not required, but consent to use the collected data was obtained (European General Data Protection Regulation, 2016/679).

**Results**

In the considered period, 167 forms were completed by nurses (20% of the total number of completed questionnaires). Fifty-one % of the nurses who responded works in University hospitals, while the remaining 49.1% works in ordinary hospitals. The considered population comes from 15 different regions, in particular 68.9% is from North Italy (Emilia Romagna, Friuli-Venezia Giulia, Liguria, Lombardia, Piemonte, Trentino-Alto Adige, Veneto), 24.6% from Central Italy (Marche, Lazio, Toscana, Umbria), and 6.6% from South and Islands (Abruzzo, Campania, Puglia, Sicilia). No answers came from 5 regions (Valle d’Aosta, Molise, Basilicata, Calabria, Sardegna). Twenty-five % (n=42) of the nurses works in an ICU that uses paper based clinical records; in particular, the percentage of ICUs that use paper based records is almost 100% in the South of Italy and Island and while is 17% in the North. The staff who don’t use EHR claims in 45% of cases to know at least one model and all of them declares that they would like to adopt an EHR. The 83% of participants using an EHR provided an overall evaluation with a rating ≥6 (Figure 1). On the contrary, the degree of satisfaction was lower for the 9 single analyzed functionalities, with a rating of 3/5 (Figure 2).

**Conclusions**

The diffusion of EHR is still limited in Italy. Despite the good overall judgement of nurses, the EHR show that they’re not performing in the specific tasks they were designed for.

**Fig. 1 (abstract A154).**
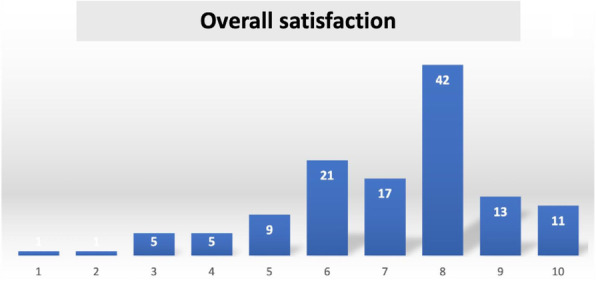
See text for description

**Fig. 2 (abstract A154).**
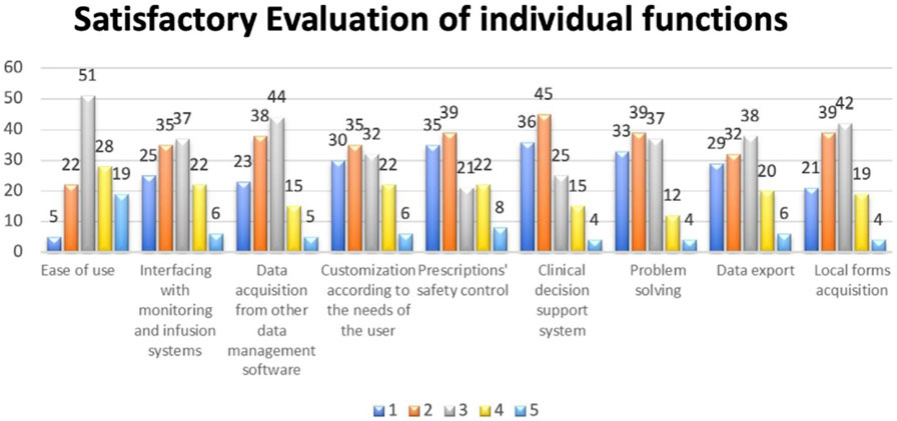
See text for description

### A155. Distribution of the electronic health record among Italian intensive care units: results from a survey across the country

#### Vichi B.^1^, Montomoli J.^2^, Saglietti F.^3^, Bergamini C.^4^, Tonetti T.^1^, Villa G.^5^, Ranieri V.M.^1^, Gamberini E.^2^, Agnoletti V.^4^, Bignami E.G.^6^

##### ^1^Department of Medical and Surgical Sciences, Anesthesia and Intensive Care Medicine- Alma Mater Studiorum-University of Bologna, Bologna, Italy ~ Bologna ~ Italy, ^2^Department of Anaesthesia and Intensive Care, Infermi Hospital, Rimini, Italy ~ Rimini ~ Italy, ^3^Department of Anesthesia and Intensive Care, IRCCS San Raffaele Scientific Institute, Milan, Italy ~ Milano ~ Italy, ^4^Anesthesia Department, Bufalini Hospital, Cesena, Italy ~ Cesena ~ Italy, ^5^Department of Anaesthesia and Intensive Care, University of Florence , Italy ~ Firenze ~ Italy, ^6^Anesthesiology, Critical Care and Pain Medicine Division, Department of Medicine and Surgery, University of Parma, Parma, Italy ~ Parma ~ Italy

###### **Correspondence:** Vichi B.

INTRODUCTION:

In the last few years, Electronic Health Record (EHR) has been gradually introduced in the Italian Intensive Care Units (ICUs). However, neither guidelines nor guidance documents belonging to national or regional government bodies have regulated the familiarity with those tools. Instead, this has been appointed to single Hospitals’ choices or even to Departments themselves. This has led to an heterogenous spread of the EHR across Italy and a different care management for patients and administrative processes. As far as we know there are no data published about the distribution of the EHR among the Italian Hospitals. The purpose of our survey is to provide the first analysis about the distribution of the EHR across the country.

METHODS:

Online survey administered to physicians, clinical fellows and nurse staff working into the Italian Intensive Care Units (ICUs) by using social networks such as Facebook, Twitter, LinkedIn, WhatsApp and emails, lasting between May 17th-27th, 2022.

RESULTS:

The total amount of the completed surveys is 817 with a 100% of completeness. The answers are coming from Healthcare Professionals employed in 19/20 Italian regions considering 60.2% from the North, 20.2 % from the Centre, and 19.5% from the South and the Islands of the Country (Figura 1a). Fifty-eight % of the participants are employed in a University-Hospitals and consist of consultants (48%), residents (31%), and nurses (21%). An EHR is used by 54.1% of those who answered the survey with similar percentages between North and Centre while the proportion of ICUs with an EHR in the South is much lower (Figure 1b). The 45.9% report they currently use paper based medical record and the large majority would like to acquire an EHR. Among them, 41.5% report their institution is planning to adopt an EHR in the next 12 months.

CONCLUSIONS:

The EHR is used by nearly a 50% of those who participated in the survey, with a lower proportion for the South compared with the North and the Centre of Italy. More than the 40% of the Healthcare Professionals who are currently using an EHR, reported they are planning to acquire one in the next 12 months.

**Fig. 1 (abstract A155).**
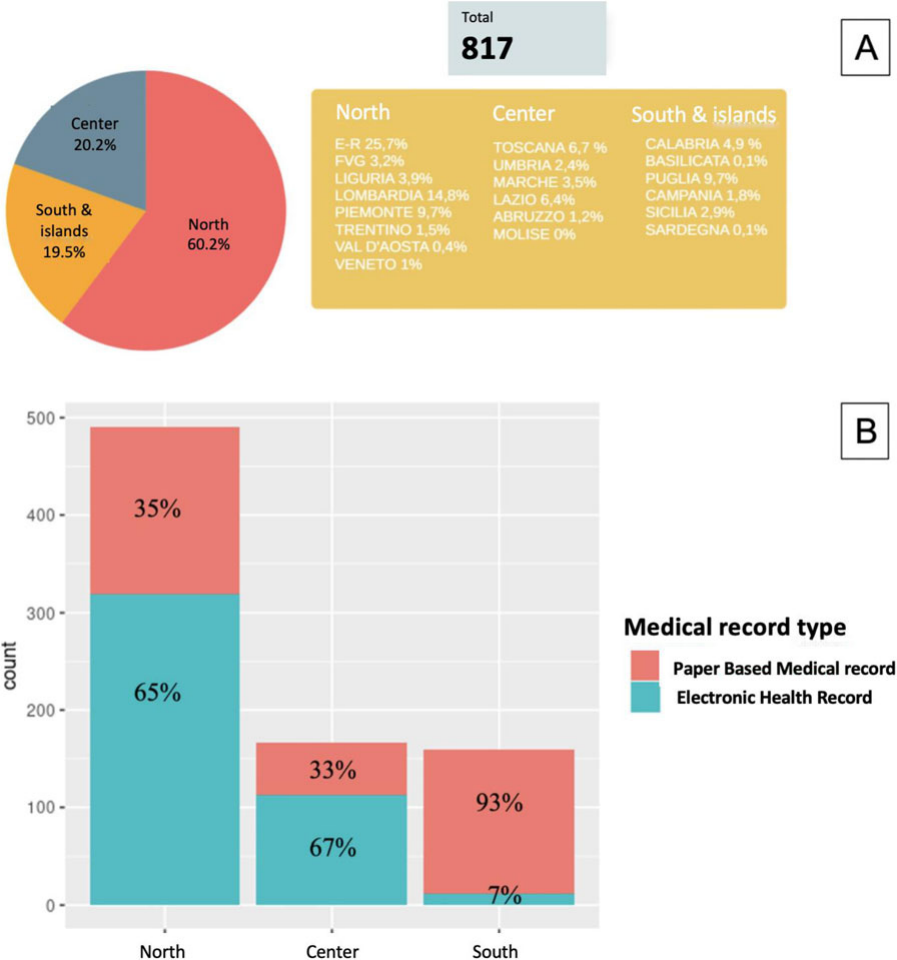
See text for description

## Ostetrica e perinatale

### A156. Use of plasma factor VII in cardiac arrest from massive post caesarean hemorrhage

#### Gammaldi D.

##### "Tortorella" - Private Hospital ~ Salerno ~ Italy

Use of Plasma Factor VII in Cardiac Arrest from Massive Post Caesarean Hemorrhage

Case Report

Domenico Gammaldi - “Tortorella” Private Hospital - Salerno

Vittoria Gammaldi - “Federico II” University - Naples

Anna Mercogliano - “Villa dei Fiori” Private Hospital - Acerra

Lyophilized blood clotting factor VII (ProvertinUm®) belongs to the blood clotting factors in the category of vitamin K-dependent antihemorrhagics.

It is used in bleedings caused by congenital or acquired coagulation disorders due exclusively or in part to a deficiency of factor VII (spontaneous or traumatic bleeding; surgery; hepatic coma)

Massive postpartum hemorrhage, partly underestimated, is the cause of the hypovolemic Cardiac Arrest described in this case report.

Primary postpartum bleeding is commonly defined as blood loss of more than 500 ml in the first 24 hours after vaginal delivery, and more than 1.000ml after Cesarean Section (secondary PPH: bleeding between 24 hours and 12 weeks after childbirth). It is based on the estimate of blood loss with three levels of severity: minor PPH (loss between 500 and 1.000 ml; greater between 1.000 and 1.500 ml and massive when one or more of the following criteria are applicable: more than 1.500 ml of persistent blood loss and / or signs of clinical shock and / or transfusion of 4 or more units of concentrated red blood cells (Arulkumaran 2009, ACOG 2006, NICE 2014).

Immediate CPR maneuvers (crowned with almost immediate success) followed by prompt surgical treatment (Hysterectomy) associated with the prompt administration of plasma Factor VII (ProvertinUm®) and Factors II, IX and X (Protromplex®), followed by FFP and CRBC, allowed to complete the intervention which was followed by a few hours post-operative course in resuscitation with awakening of the patient and discharge after three days.

- Cardiac Arrest in the Operating Room: Resuscitation and Management for the Anesthesiologist: Part 1 Vivek K. Moitra, MD, * Sharon Einav, MD, † Karl-Christian Thies, MD, ‡ Mark E. Nunnally, MD, § Andrea Gabrielli, MD, ∥ Gerald A. Maccioli, MD, ¶ Guy Weinberg, MD, # Arna Banerjee, MD, ** Kurt Ruetzler, MD, †† Gregory Dobson, MD, ‡‡ Matthew D. McEvoy, MD, ** and Michael F . O'Connor, MD, FCCM§§
- Cardiac Arrest in the Operating Room: Part 2 — Special Situations in the Perioperative Period Matthew D. McEvoy, MD, * Karl-Christian Thies, MD, FRCA, FERC, DEAA, † Sharon Einav, MD, ‡ Kurt Ruetzler, MD, §∥ Vivek K. Moitra, MD, FCCM, ¶ Mark E. Nunnally, MD, FCCM, # Arna Banerjee, MD, * Guy Weinberg, MD, ** Andrea Gabrielli, MD, FCCM, †† Gerald A. Maccioli, MD , FCCM, ‡‡ Gregory Dobson, MD, §§ and Michael F. O'Connor, MD, FCCM∥∥
- SNLG Post Partum Hemorrhage-2020

### A157. PRES: a differential diagnosis option to consider for headache after c-section in a patient without hypertension. a case report

#### Colasanti A., Casali M., De Masi G., Bizzarri A., Ferialla S.

##### Azienda Ospedaliera "Santa Maria" ~ Terni ~ Italia

###### **Correspondence:** Colasanti A.

Background

A high incidence of posterior reversible encephalophathy syndrome (PRES) has been observed in women with pre-eclampsia and pregnancy related hypertension on imaging. However this association has been documented mostly after convulsions occurred.The essential features of Posterior Reversible Encephalopathy Syndrome (PRES) are headache, mental changes, seizures and visual symptoms . It usually completely reverses with treatment, although permanent sequelae are possible in case of delayed or missed diagnosis.

Case report

We present the case of a White Female 32-year-old woman, 2 gravida, 1 para, that comes to our Ob-Gyn Department in February 2022 for an elective casearean section at 40 weeks of gestation. The pregnancy developed physiologically. No signs or reports of hypertension,no oedema to lower limbs,no signs of any pregnacy-related pathology.

She has a C-section under spinal anaesthesia, performed with a 25-Gauge pencil point needle, 12 mg of hyperbaric bupivacaine and 2.5 mcg of sufentanil. No complications were referred during surgery and in the post-operative period. She is discharged in the 3^rd^-day as protocol. She cames back after 4 days as scheduled, to check the surgical suture.

10days after the c-section, she is admitted to our Ob-Gyn emergency service referring a severe headache and dyplopia. She is taken in charge, evaluated by the gynaecologist and by the obstetric dedicated consultant anaesthetist,immediately treated as suspected PDPH (post dural puncture headache) and scheduled to perform an urgent neuroimaging assesment on the next day,given the peculiarity of the presentation. Analgesic therapy with NSAIDs every 8 hrs. is prescribed and no opioid therapy was required. The head MRI shows the pattern of PRES associated with vasogenic oedema. The neurological examination that didn’t find any deficit in strength or sensitivity or in the cranial nerves.

She undergoes an oculistic and ophthalmological consultation too,which reveales a slight deficit of the right temporal visual hemifield.

On the 9^th^-day after admission the patient undergoes a control brain MRI which confirmed a complete regression of the vasogenic oedema and is discharged without further therapy.

**Conclusions**

Although PRES is usually associated with definite pathological conditions, it is not always the case, as was for the patient here described, who had no predisposing factors in her past clinical history. PRES usually presents with acute non specific features and it can be misdiagnosed with other serious diseases, since the physician will be helped by the knowledge of this syndrome to promptly start diagnostic workup and treatments, and avoid permanent neurological deficits.

Informed consent to publish had been obtained

**Keywords**

Posterior reversible encephalopathy syndrome,pregnancy, vasogenic edema

**References**

1. Chao,AS.,Chen,YL.,Chang,YL. Et al. Severe pre-eclamptic women with headache.is posterior reversible encephalopathy syndrome an associated concurrent finding ? BMC Pregnancy Childbirth 20,336 (2020). 10.1186/s12884-020-03017-4

2. Achar SK,Shetty N,joseph TT. Posterior reversible encephalopathy syndrome at term pregnancy. Indian J Anaesth.2011;55(4):399-401. doi:10.4103/0019-5049.84856

3. Mehta M,Van Der Meer A,Sharma Posterior reversible encephalopathy syndrome (PRES):a rae complication of pre-eclampsia. Archives of Disease in Childhood-Fetal and Neonatal Edition 2012;97:A56-A57

4. Rajeshwari.K.S:,Agarwal,V.,Satish,S. et al. an usual presentation of posterior reversible encephalopathy syndrome, a case report. Egypt J neurol Psychiatry Neurosurg 57,3 (2021). 10.1186/s41983-020-00252-6

5. Liman Tg,Bohner G, Heuschmann PU,Scheel M, Endres M, Siebert E. Clinical and radiologicaldifferences in posterior reversible encephalopathy syndrome between patients with preeclampsia-eclampsia and other predisposing diseases. Eur J neurol. 2012jJul;19(7):935-43.doi:10.1111/j.1468-1331.2011.03629.x. Epub2012 Jan 17. PMID:22248235

6. Erez O, Romero R,Jung E, ChaemsaithongP,Bosco M Suksai M,Gallo DM, Gotsch F. Preeclampsia and eclampsia: the conceptual evolution of a syndrome. Am J Obstet gynecol.2022 Feb;226(2S): S786-S803. doi:10.1016/j.ajog.2021.12.001. PMID:35177220; PMCID:PMC8941666.

### A158. A novel continuous labor pain monitoring system: needs and perspectives

#### Calabrese A.^1^, Compagnone C.^2^, Taddei M.^1^, Trombi G.^2^, Bellini V.^1^, Bignami E.^1^

##### ^1^Anesthesiology, Critical Care and Pain Medicine Division, Department of Medicine and Surgery, University of Parma ~ Parma ~ Italia, ^2^2nd division of Anesthesiology and intensive care, Parma University Hospital, Parma, Italy ~ Parma ~ Italia

###### **Correspondence:** Compagnone C.

Background

Effectiveness of labor analgesia is usually monitored by means of unidimensional pain scales administered to the patient at predefined time intervals and by clinical testing of analgesia extension. Although reliable, these methods don’t provide information on neuraxial block onset and duration, nor on pain reemergence pattern. Continuous evaluation of contraction pain is expected to be more sensitive and accurate in describing such parameters. Evidence suggests that a different approach from today’s standard is desirable, and alternatives have been proposed. Strategies consisting in more frequent pain scale administration1, 2 or their compilation by the patient have been proposed for evaluation of analgesia onset3. By being insistently asked to elaborate or write down information on their pain, patients could feel uncomfortable and the attempt of providing better analgesia could in turn interfere with a very personal experience. Another weakness of these strategies resides in their hit-and-miss approach to analgesia monitoring, that would be much less accurate in comparison to a continuous patient input based method - a tool we are currently lacking.

Project

We are working on the development of a patient operated device that enables parturients to input onset and termination of contraction pain and its severity in real time by a simple and intuitive interface, such as a button or smartphone application, paired to a Uterine Activity Monitor. Caring clinicians could therefore be provided with a telemetry-like tool for constantly and consistently keeping track of ongoing labor analgesia treatments and be able to titrate therapy accordingly. Such a tool could be used to derive a relation between pain duration (Pd) as input by the parturient, and contraction duration (Cd) as monitored through tocography, thus providing a contraction index (Pd/Cd), a dimensionless parameter for monitoring individual response to administration of analgesia during labor. Other measurements could investigate the latency of pain onset after contraction initiation and its variations after administration of analgesia. In the graph below, we show the variables we intend to study and the hypothetical interactions between them (Figure 1).

Conclusion

Real time labor pain monitoring would assist practitioners in customizing analgesia based on moment-to-moment parturients’ feedback. Key features of the device are ease of use, discreteness and ability to transmit remotely parturients’ inputs, allowing better utilization of staff resources and the possibility of more privacy for the patient. We expect that analysis of data obtained by this real time continuous monitoring could have a positive impact on clinical practice. Possible applications could be tailoring analgesia based on individualized pain thresholds and more precise application of patient centered protocols based on the semi-objective data collected. This multimodal continuous monitoring could allow more objective comparisons of different strategies (e.g. proactive-reactive, preemptive), techniques (e.g. epidural, combined spinal-epidural, dural puncture epidural) and pharmacologic regimens of labor analgesia.

**References**

1. Tan H Sen, Reed SE, Mehdiratta JE, et al. Quality of labor Analgesia with Dural Puncture Epidural versus Standard Epidural Technique in Obese Parturients: A Double-blind Randomized Controlled Study. Anesthesiology. 2022;136;(5):678-687.

2. Chau A, Bibbo C, Huang CC, et al. Dural puncture epidural technique improves labor analgesia quality with fewer side effects compared with epidural and combined spinal epidural techniques: A randomized clinical trial. Anesth Analg. 2017; 124(2):560-569.

3. Benjamin D. Grant, Chelsey A. Smithb, Philip E. Castlec, D, Michael E. Scheurere and RR-K. Labor Analgesia Onset with Dural Puncture Epidural Versus Traditional Epidural Using a 26-Gauge Whitacre Needle and 0.125% Bupivacaine Bolus: A randomized Clinical Trial. Physiol Behave. 2017;176(5):139-148.

**Fig. 1 (abstract A158).**
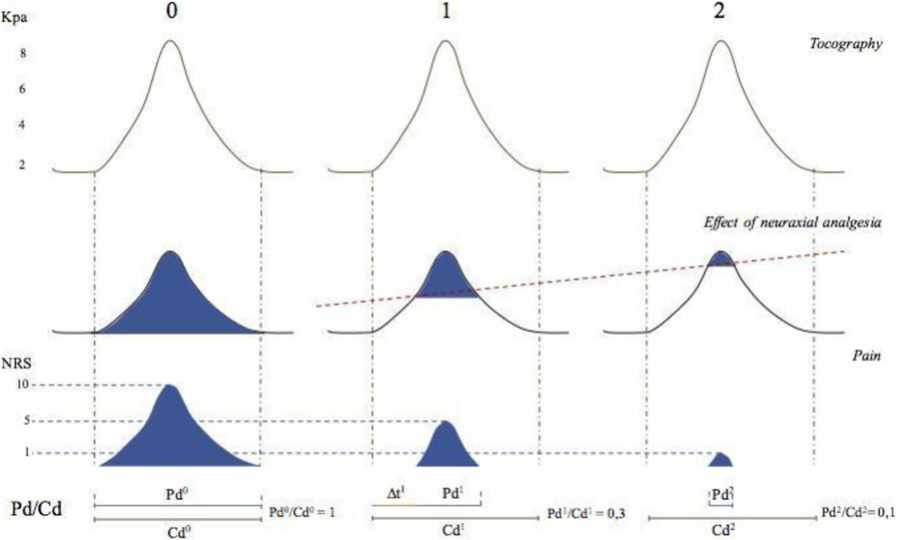
Variables to be studied and hypothetical interplays and interactions between them: 0 basal condition; 1, 2: After onset of neuraxial blockade; Pd: Pain duration; Cd: contraction duration; Pd/Cd: contraction index; ∆ t: time from onset of contraction to onset of pain

### A159. Severe COVID and pregnancy: a case report

#### Maio M.^2^, Giunta F.^2^, Bertolino E.^2^, Corno E.^2^, Guerriero F.^1^, Quaglia S.^2^

##### ^1^AOU Città della Salute e della Scienza- Anestesia e Rianimazione 3 ~ Torino ~ Italia, ^2^AOU Città della Salute e della Scienza- Anestesia e Rianimazione 4- PO S. Anna ~ Torino ~ Italia

###### **Correspondence:** Giunta F.

Severe Acute Respiratory Syndrome Coronavirus-2 (SARS-CoV-2), may lead to respiratory failure, multi-organ dysfunction, and death. Management of Covid infection in pregnant women is based on case reports, case series, and observational studies. There are few data focused on managing pregnant patients with severe COVID19. We report the case of a pregnant woman with COVID-19 related ARDS. CASE HISTORY On December 2021 a 35-years-old, 32 weeks pregnant woman was admitted to second level hospital for cough and fever. Medical history included hypertension, obesity (BMI 42) and diabetes on insulin therapy. She was not vaccinated. Molecular swab tested positive for Covid infection. Steroid therapy was started and she was transferred to Sant'Anna Hospital. She was afebrile, with 96% oxygen saturation with 2l/min of oxygen on nasal canula, respiratory rate (RR) >20 breaths/minute. Chest X-ray (fig1) showed presence of multiple extensive consolidation type densities mainly peripheral. On 5th day due to impaired respiratory exchanges and the poor response to the High Flows trial (p/f < 250), the patient underwent an emergency caesarean section. The cesarean section was performed under locoregional anesthesia with HNIV (pressure support of 8 cmH2O, PEEP 6 cmH2O, FiO2 0.6), without complications. The clinical conditions of the twins at birth were satisfactory (weight: 1900 and 1800 g, APGAR score: 8-8 and 7-8 the first and the second twin, respectively). Then she was transferred to ICU. She was immediately NIV dependent, and kept in Rodin position alternating lateral decubitus with initial improvement in respiratory exchanges. On 7th day the patient's respiratory condition worsened (pH 7.39 pCO2 45, pO2 74, HCO3- 26) despite non invasive ventilation optimization and increase in fiO2 up to 100%. Bed-side lung ultrasounds showed no signs of pleural effusion or pneumothorax. Rapid sequence intubation was performed with analgosedation and curarization. Lung-protective ventilation was started; tidal volume of 6-8 ml/kg, PEEP was set to get the best driving pressure, FiO2 was set in order to achieve a PaO2 >70 mmHg and RR set to keep PaCO2 in normal range. Patient started a trial of prone ventilation with partial and unsatisfactory improvement of respiratory exchanges. No response was obtained with the recruitment maneuvers. iNO 20 ppm was started as rescue therapy with an increase in p/f values up to 160. The following day, due to the p/f value below 100 and respiratory exchanges not responsive to maximal therapies, the patient was placed in veno-venous Extra Corporeal Membrane Oxygenation (ECMO) and transferred to ECMO center. At the CT scans chest (fig 2) was observed presence of intraluminal filling defects such as pulmonary embolism in the left lower pulmonary artery and extensive, bilateral parenchymal thickenings. On the 17th day ECMO was removed. On the 23th day a surgical tracheostomy was performed to facilitate weaning. On the 28th day the patient was transferred to the second level hospital and quickly discharged.(fig 3) CONCLUSION The early transfer to a third level center ensured a prompt intensification of maternal and neonatal treatment thanks also to the multidisciplinary nature of these centers.

Informed consent to publish had been obtained.

**Fig. 1 (abstract A159).**
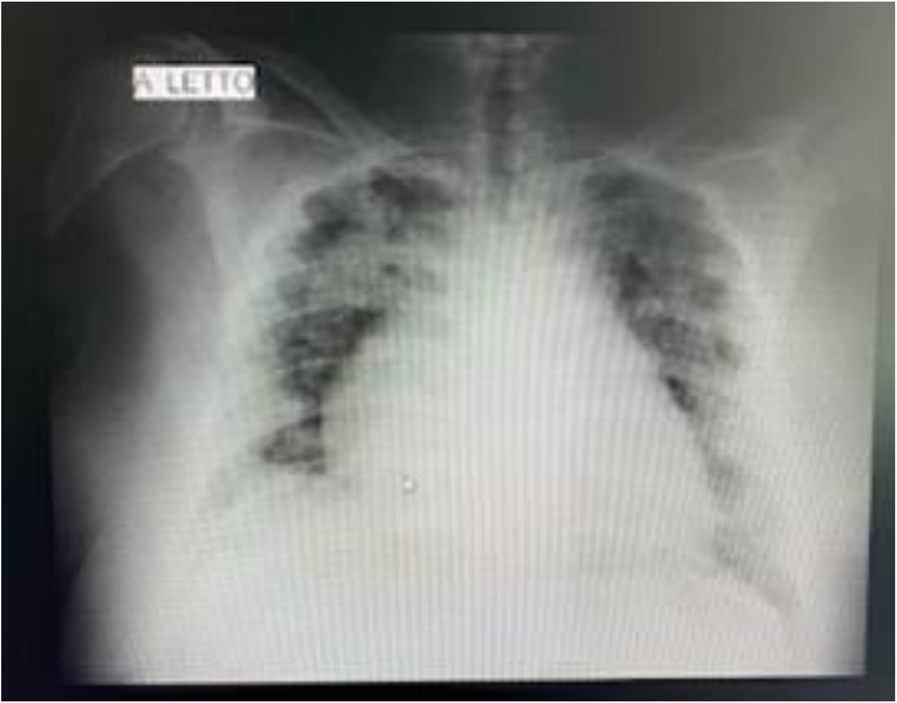
See text for description

**Fig. 2 (abstract A159).**
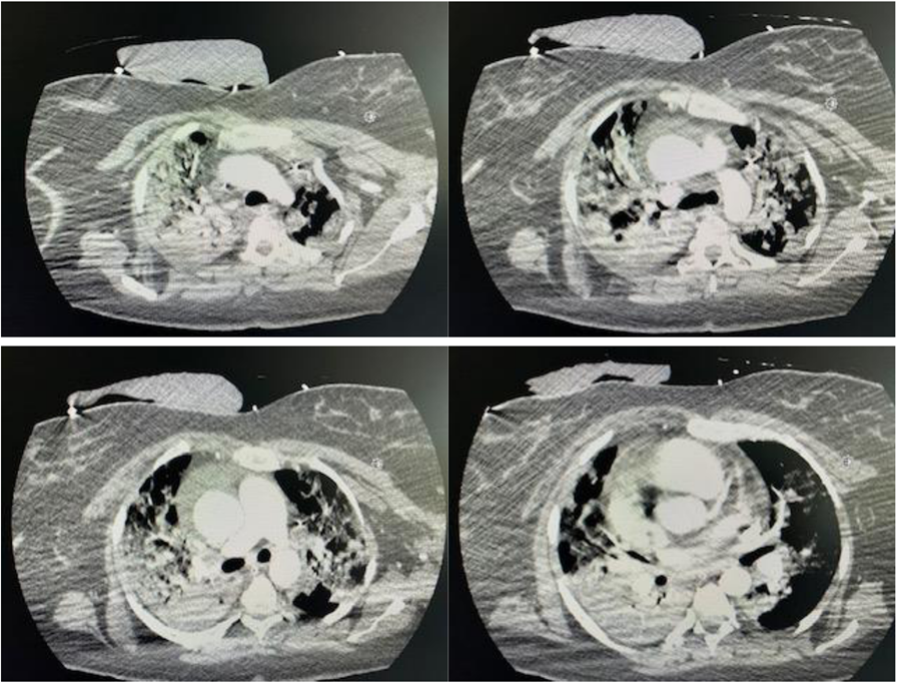
See text for description

### A160. Bilateral erector spinae plane block (ESPB) versus posterior quadratus lumborum block (P-QLB) for postoperative analgesia after caesarean section: an observational prospective study

#### Di Muro M., Zanfini B.A., Biancone M., Catarci S., Frassanito L., Capone E., Draisci G.

##### IRCCS A. Gemelli University Polyclinic Foundation, Department of Anesthesiology, Intensive Care and Emergency Medicine ~ Roma ~ Italia

###### **Correspondence:** Di Muro M.

Background

Management of pain after caesarean section (CS) represents an important anaesthesiologic issue, since it is often suboptimal, leading to delayed functional recovery and chronic pain. Currently, the postoperative analgesic strategy mostly relies on intrathecal morphine (ITM) and multimodal analgesic regimen[1], but the need for opioid sparing techniques is emerging. Paraspinal fascial plane blocks, as QLB[2] and ESPB performed at T9 level[3], have been proposed, because of their demonstrated effect on visceral and somatic pain. The aim of our study is to assess the efficacy, the feasibility and the safety of bilateral ESPB compared to bilateral p-QLB for the management of postoperative pain after CS conducted under spinal anaesthesia without ITM. The primary outcome endpoint is Numerical Rating Scale for pain at rest (NRSr) and on movement (NRSm) at 12 hours. Secondary outcomes include: total morphine consumption in the first 24 hours after surgery; time to first opioid request; NRSr and NRSm score at 0, 2, 6 and 24 hours; vital signs; any adverse event.

Methods

The study is an observational comparative study, including a prospective cohort (the ESPB group) and a historical control group (the p-QLB group[4]) from our institution. For each group, 13 parturients ASA2 with normal singleton pregnancy scheduled for elective CS under neuraxial anaesthesia without ITM gave informed consent to treatment data. Both group were performed at the end of surgery with an anaesthetic mixture of ropivacaine 0.375% + epinephrine 5 mcg/mL 20 mL each side. NRSr and NRSm were assessed at prespecified time points, together with vital signs, morphine consumption, time to first analgesic request. Any adverse event was reported.

Results

No difference in NRSr and NRSm was reported at any time interval between the two groups. Total morphine consumption and time to first analgesic request did not differ between groups. No adverse event occurred.

Conclusions

ESPB performed at T9 level seems as effective and safe as p-QLB in providing analgesia after elective CS under neuraxial anesthesia without ITM. ESPB may be easier to perform than p-QLB, therefore representing a promising technique to be implemented in postoperative pain management protocols.

Trial registration

ClinicalTrials.gov ID NCT05348083

References

1. Roofthooft E, Joshi GP, Rawal N, Van de Velde M, Joshi GP, Pogatzki-Zahn E, et al. PROSPECT guideline for elective caesarean section: updated systematic review and procedure-specific postoperative pain management recommendations. Anaesthesia. 2021;76(5).

2. Tan H Sen, Taylor C, Weikel D, Barton K, Habib AS. Quadratus lumborum block for postoperative analgesia after cesarean delivery: A systematic review with meta-analysis and trial-sequential analysis. Vol. 67, Journal of Clinical Anesthesia. 2020.

3. Kendall MC, Alves L, Traill LL, De Oliveira GS. The effect of ultrasound-guided erector spinae plane block on postsurgical pain: A meta-analysis of randomized controlled trials. BMC Anesthesiol. 2020;

4. Zanfini BA, Biancone M, Famele M, Catarci S, Lavalle R, Frassanito L, Piersanti A, Olivieri C, Lanzone A, Draisci R, Draisci G. Comparison of ropivacaine plasma concentration after posterior Quadratus Lumborum Block in Cesarean Section with ropivacaine with epinephrine vs. plane. Minerva Anestesiol. 2021: 979-986.

### A161. Α-2 agonists as compared to fentanyl as adjuvants for spinal anesthesia in patients undergoing elective cesarean section: a systematic review and meta-analysis with trial sequential analysis

#### Triolo T.^1^, La Via L.^2^, Bartolotta N.^3^, Cirica G.^1^, Continella C.^1^, Lanzafame B.^4^, Perna F.^2^, Murabito P.^2^, Astuto M.^2^, Sanfilippo F.^2^

##### ^1^Department of Anesthesia and Intensive Care, University "Magna Graecia" ~ Catanzaro ~ Italia, ^2^Department of Anesthesia and Intensive Care, AOU "Policlinico - San Marco" ~ Catania ~ Italia, ^3^Ospedale Buccheri La Ferla ~ Palermo ~ Italia, ^4^ASP Siracusa ~ Siracusa ~ Italia

###### **Correspondence:** Triolo T.

Background: Elective cesarean section (CS) is usually executed using spinal anesthesia (SA), which requires the use local anesthetic (LA), possibly combined with adjuvant drugs. We performed a systematic review and meta-analysis aimed at studying the advantages of α-2 agonists as compared to fentanyl during SA for CS.

Materials and methods: We screened PubMed and EMBASE for randomized controlled trials (RCTs). We calculated the mean difference (MD) for continuous outcomes, and the relative risk (RR) for dichotomous outcomes, using a random-effect model with 95% confidence interval (CI). We performed a Trial Sequential Analysis (TSA) assuming an alpha risk of 5% with a power of 80%. The primary outcome was the time to first rescue analgesia.

Results: Eight RCTs were included. Time to first rescue analgesia was significantly longer when the α-2 agonists were used (MD 85.9 min [95%CI 23.8, 147.9]; p=0.007). Duration of sensory block was also different in the α-2 group (MD 40.5 [95%CI 20.21,60.7]; p<0.0001), while no differences were found for onset of sensory block and onset and duration of motor block. Rates of shivering and nausea or vomit were significantly lower in the α-2 agonist group, while risk of hypotension or respiratory depression were not different. The TSA on the primary outcome suggests the need of further research before drawing conclusions.

Conclusions: α2-agonists seem to increase the time to first rescue analgesia and to prolong the duration of sensory block when used as adjuvants to LA in CS patients. Also, α2-agonists may reduce the incidence of shivering and nausea or vomit.

**Table 1 (abstract A161).**
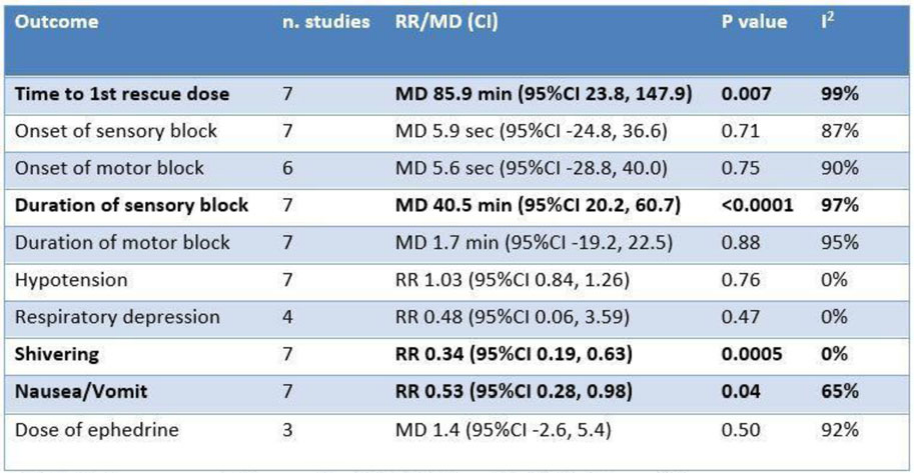
Summary of the results. *RR*: Relatice risk; MD: Mean difference

### A162. Impact of epidural analgesia on caesarean section rates according to robson group cassification system

#### Palladino T., Ricciardi P., Vaira P., Sciannammè N., Ritrovato P., Villani M.

##### IRCCS Casa Sollievo della Sofferenza, Anestesia e Rianimazione I ~ San Giovanni Rotondo (Fg) ~ Italia

###### **Correspondence:** Palladino T.

INTRODUCTION

The role of epidural analgesia in increasing the incidence of caesarean sections is still controversial. The objective of this paper is to evaluate the potential association between Epidural Analgesia (EA) and caesarean sections (CS), according to Groups 1, 2A, 3 and 4A of Robson Ten Group Classification System (TGCS), in Obstetrics and Gynaecology Unit in San Giovanni Rotondo (South - Italy).

METHODS

We collected data of all women who delivered in our Unit from January 2021 to April 2022 and analysed incidence of CS in Groups 1, 2A, 3 and 4A of TGCS. We assessed CS among patients who received EA during labour and compared among those who did not receive EA, for each of the aforementioned Groups.

RESULTS

Epidural analgesia is not statistically associated with increasing caesarean sections in Groups 1, 3 and 4A. Conversely, Epidural Analgesia is significantly associated with lower CS rate for Group 2A (nulliparous singleton cephalic term women in induced labour) (21.1% vs 46.3%, p = 0.0003).

CONCLUSIONS

Our data have shown that CS rate is significantly lower for nulliparous women at term who have been induced and receiving epidural analgesia during labour. We might assume a protective role of EA against caesarean sections in our Group 2A. Further researches will evaluate the differences between receiving EA and not receiving EA Groups in term of clinical characteristics and labour management.

**Table 1 (abstract A162).** See text for description

TGCS GROUPS				CS IN LABOUR		
POP		EA	NO EA	EA	NO EA	
**1**	222	150 67.6%	72 32.4%	13 8.7%	3 4.2%	*0.224*
**2A**	220	166 75.5%	54 24.5%	35 21.1%	25 46.3%	**0.0003**
**3**	303	101 33.3%	202 66.7%	3 3.0%	3 1.5%	*0.38*
**4A**	125	63 50.4%	62 49.6%	4 6.3%	3 4.8%	*0.71*
**TOT**	870	480 55.2%	390 44.8%	55 11.5%	35 8.7%	*0.18*

### A163. Hybrid telemedicine path for pregnancy anesthesiological assistance

#### Bellini V.^2^, Serini C.^1^, Craca M.^2^, Panizzi M.^2^, Trombi G.^2^, Compagnone C.^2^, Beretta L.^1^, Bignami E.^2^

##### ^1^Department of Anesthesia and Neurointensive Care, San Raffaele Scientific Institute, 20132 Milan, Italy. ~ Milano ~ Italia, ^2^Anesthesiology, Critical Care and Pain Medicine Division, Department of Medicine and Surgery, University of Parma, Viale Gramsci 14, 43126 Parma, Italy. ~ Parma ~ Italia

###### **Correspondence:** Compagnone C.

Introduction

A pregnancy care program is essential for any health facility with an obstetrics unit. In this multidisciplinary process, the anesthesiologist has multiple functions. Not only labor pain relief or ensure safety in emergency settings, but it also participates in all the program's objectives, including the promotion of health and the humanization of assistance [1]. Following Covid pandemic explosion and its Ministerial recognition, more and more medical disciplines are approaching telemedicine, which is considered the digital evolution of medicine. Anesthesia and Intensive Care are also no exception, so much so that the term Tele-Anesthesia has been coined [2]. In this context, these techniques demonstrate a dual role: on the one hand, optimizing the organization, on the other, increasing the possibilities of precision medicine. However, everything must be done by respecting and guaranteeing the highest level of safety. Our project aims to explore the potential role of telemedicine in the context of this care path. With this new proposal, we expect to reduce hospital admissions for pregnant women by decreasing the potential infectious risk and promoting a care program closer to their needs while maintaining good quality and safety of care and satisfaction for the patients themselves.

Study Project

The project consists of a multicentre observational study, the main objective of studying a definitive "hybrid" path of anesthetic care pathway for pregnancy. With this new program, women interested in childbirth analgesia will face two phases:

1st TELE - INFORMATION. Patients will participate in informative interview groups via a common online platform to record the event. At the end of the information, the anesthetist will answer any doubts and questions of the participants.

2nd TRADITIONAL VISIT. After the informative interview, the patients will book a single visit for the anesthetic evaluation, during which the consent signature will also be performed, as agreed during the informative report.

This proposal is supported by an experience of 50 cases in the era of Covid pandemic lockdown. The meetings were of 12-15 full-term pregnant women aged 25-35 years, with percentages of foreigners under 10%. The complete anesthetic examination and the expression of the signed informed consent was conducted on the day of the entry of the pregnant woman during labor. This first preliminary moment, initially dictated more by the need for pandemic containment, confirmed the project's feasibility and demonstrated the absence of important problems related to this methodology.

Conclusions

The role of telemedicine will have increasing weight in modern medicine; this will also happen in our speciality where we already have the first positive experiences. Taking advantage of these methods within the pregnant women pathway could bring significant advantages, both in the organizational and public health fields, without changing the degree of clinical safety or satisfaction. Our project could provide a preliminary basis for this theory. The following step will be to develop the entire path in telemedicine. The degree of satisfaction of the participants will be assessed before and after.

**References**

1. https://www.salute.gov.it/imgs/C_17_pubblicazioni_573_allegato.pdf

2. Bellini V, Valente M, Gaddi AV, et al. Artificial intelligence and telemedicine in anesthesia: potential and problems. Minerva Anestesiol. 2022 Feb 14. doi: 10.23736/S0375-9393.21.16241-8.

**Fig. 1 (abstract A163).**
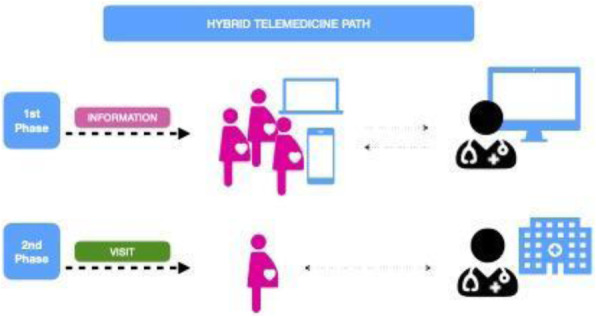
Hybrid Telemedicine Path

### A164. Anesthesiologic management of an obstetric patient with subaortic stenosis: a case report

#### Pisoni M., Pistidda L., Secchi F., Bazzoni C., Balata A., Mura P.P., Pasero D.

##### AOU Sassari Anestesia e Rianimazione ~ Sassari ~ Italy

###### **Correspondence:** Pisoni M.

**Background**

The first choice anesthetic technique for cesarean section is neuroaxial anesthesia [1] due to the possible risks connected with the general anesthesia and perioperative patient management [2]. Nevertheless, neuroaxial anesthesia can be contraindicated in specific clinical conditions [3,4].

**Case Report**

A 41-year-old pregnant woman with symptomatic subaortic hypertrophic stenosis (Figure 1), NYHA II-III, EF 60%, moderated mitral and tricuspid regurgitation with increased atrio-ventricular gradient (extimated PAPs=53 mmHg) and hyperthyroidism, was admitted to hospital for CTG monitoring at 34° weeks of pregnancy. After multidisciplinary evaluation, the team planned an elective cesarean section. Surgery took place in a safe setting under a team management of cardiac anesthesiology and cardiac surgery. In the OR, standard and invasive cardiac monitoring was applied prior to general anesthesia induction. Rapid intubation sequence was performed using Fentanyl, Thiopentone sodium and Rocuronio. In addition, Transesophageal Echocardiography (TEE) was performed intraoperatively, in order to ensure a more reliable monitoring of the cardiac function and of the hemodynamic parameters. General anesthesia was maintained with inhaled Sevofluorane. Hemodynamic parameters remained stable both during induction and intraoperative phases (Figure 2). The patient was awakened in the cardiac intensive care unit without any complication and she was moved to gynecology ward in the same day. At birth, infant had an APGAR score of 9 at 1’ e 5’.

**Conclusion**

Although neuroaxial anesthesia is the best choice for elective cesarean section, it should be avoided in high risk cardiac patient with severe aortic stenosis, due to the high risk of hemodynamic instability. General anesthesia with appropriate hemodynamic monitoring is safer and it should be preferred. Moreover, Thiopentone sodium might be a good option for the induction of general anesthesia in order to maintain hemodynamic stability, when Etomidate is not available. Transesophageal ecocardiography is mandatory for diagnosis and staging, in pregnant patients with aortic stenosis undergoing a non-cardiac surgery according to the American Heart Association guideline [4]. Furthermore, intraoperatively TEE monitoring allowed us to optimize the hemodynamic and pharmacologic management of the patient and could be suggested as an elective monitoring in complex cases such as the present one.

Written informed consent for publication of their clinical details and/or clinical images was obtained from the patient (*BioMed Central consent form*)

**Reference**

1. Apfelbaum JL, Hawkins JL, Agarkar M, Bucklin BA, Connis RT, Gambling DR, et al. Practice Guidelines for Obstetric Anesthesia: An Updated Report by the American Society of Anesthesiologists Task Force on Obstetric Anesthesia and the Society for Obstetric Anesthesia and Perinatology ∗. Anesthesiology. 2016.

2. Guglielminotti J, Landau R, Li G. Adverse Events and Factors Associated with Potentially Avoidable Use of General Anesthesia in Cesarean Deliveries. Anesthesiology. 2019;130(6):912–22.

3. Arendt KW, Lindley KJ. Obstetric anesthesia management of the patient with cardiac disease. Int J Obstet Anesth. 2019;37(September 2018):73–85.

4. Nishimura RA, Otto CM, Bonow RO, Carabello BA, Erwin JP, Guyton RA, et al. 2014 AHA/ACC guideline for the management of patients with valvular heart disease: Executive summary :A report of the american college of cardiology/american heart association task force on practice guidelines. Vol. 129, Circulation. 2014. 2440–2492 p.

**Fig. 1 (abstract A164).**
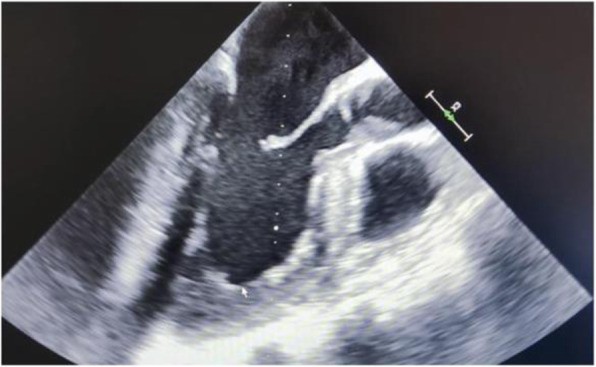
Subaortic Stenosis

**Fig. 2 (abstract A164).**
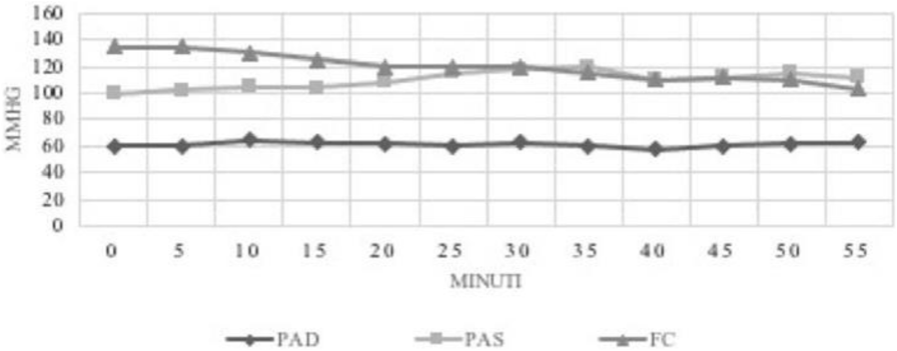
Perioperative hemodynamic parameters

### A165. Spontaneous hepatic hemorrhage (HSH) in hellp syndrome: a case report

#### Urti L.^1^, Yurikova A.^1^, Maio M.^1^, Zuccolin S.^2^, Quaglia S.^1^

##### ^1^Citta' Della Scienza E Della Salute Di Torino Presidio OSP. S. Anna ~ Torino ~ Italy, ^2^università degli studi di Torino ~ Torino ~ Italy

###### **Correspondence:** Yurikova A.

The spontaneous hepatic hemorrhage (SHH) is a rare and potentially fatal event of the HELLP syndrome, it occurs in the 1-2% patients suffering from the syndrome.

Mortality of pregnant women with rupture of the hematoma can reach up to 60-86% and fetal mortality rate reaches 56-75%.

The incidence of SHH is 1:40000-1:225000.

The pathogenesis is not certain, probably due to endothelial microvascular damage possibly connected to eutocic childbirth, determining deposition of fibrin and obstruction of the hepatic sinusoidal vessels, with consequent increase in the values of liver enzymes, areas of necrosis, intrahepatic hemorrhages and formation of subcapsular hematomas with a tendency to spontaneous rupture.

Case report:

A 31-year-old pregnant woman at 37w + 3 EG in para 1001, history of renal lithiasis with need for right ureteral stent and lithotripsy, a previous spontaneous birth complicated by EPP.

Access to the emergency room for acute pain in the right hypochondrium, normal blood chemistry, except thrombocytopenia, normal blood pressure.

Initially admitted in moderate complexity. After 24 hours Intrauterine fetal death with marked worsening of the maternal pain symptoms, so emergency caesarean section is indicated. Forced position in left lateral decubitus, subarachnoid anesthesia is performed.

After fetal extraction, massive haemoperitoneum occurs starting from the upper abdominal area with severe haemorrhagic shock, need for conversion to general anesthesia, amino support, blood components, surgical hepatic patching, vac therapy application, total blood loss 7000 ml.

To date, surgical follow-ups continue.

The inclusion in a III level structure allowed the multidisciplinary approach which influenced the good outcome of this clinical case.

Informed consent to publish was obtained.

## Sicurezza, qualità e rischio clinico

### A166. The face under the mask in icu: can a difficult verbal communication cause a legal dispute?

#### Alampi D.^1^, De Gregori F.^2^

##### ^1^Sapienza University, Department of Clinical and Surgical Translational Medicine, Unit of Anesthesia and Intensive Care Medicine, Rome, Italy ~ Roma ~ Italia, ^2^ICOT ~ Latina ~ Italia

###### **Correspondence:** Alampi D.

**Background**

Human relationships are based on communication. The information represents the concept expressed by one interlocutor and received by the other. This flow of information can be diverted in each step.

In intensive care the doctor-patient relationship, and more often doctor-relative, finds a fundamental cornerstone in correct communication. Intensivists must give correct and exhaustive information without throwing the listener into discomfort. They must make sure that the message has been received and ask for feedback. We know that even non-verbal communication is important to vehicle emotions.

Non-verbal communication is represented by the movements of the body, hands and face. Surgical mask partially hides the face preventing the correct interpretation of the facial expression [1].

Most of the muscles involved in primary emotions are in the part of face we can’t see under the mask.

In this way it is foreseeable that non-verbal communication is not very effective.[2]

We tried to know how much we can explain when our face is covered by a surgical mask without the help of mimic expressions.

**Materials and methods**

We proposed to one intensivist and one rehabilitation technician to simulate a situation of dialogue between the doctor and the relative, recording the interviews. The intensivist and the rehabilitator played doctors role in turn. We enrolled ten volunteers which played relatives role.

The interview took place in a dedicated room, with chairs, so people could sit or stand.

The doctor-character had to communicate a diagnosis with a bad prognosis to the relative-character. After the interview the relative character filled out a short questionnaire about the clinical information received and the sensations experienced.

Then everybody looked at recordings to analyze the interview from an external point of view.

**Results**

The volunteers answered the questions with similar percentages even if the interview with the doctor was less convincing. Answers to questionnaire are reported in table 1 and table 2.

We note that volunteers didn’t understand clinical news as well as we wanted. In most cases they failed to interpret the doctor's feelings. Reviewing their interviews, the doctor and the rehabilitator noticed attitudes and words that they would have changed if they had seen each other before.

**Conclusions**

Communication is a process of exchanging messages that integrates verbal and non-verbal signals. The deficiency of one of the two components affects the exact interpretation of the communicative message. The emotions cause facial changes especially of the mouth, but surgical mask covers our lips. Furthermore the mask affects the volume and tone of the voice.

In the simulated interviews the doctor was considered distant, hasty and not very empathetic. Even doctors have recognized modifiable and improvable attitudes.

Bad communication undermines the therapeutic alliance. This leads to a distrust of the doctor paving the way for possible legal proceedings.

**References**

1. Alampi D. Mirror, mirror I ask, what hides behind the doctor’s mask? Minerva Anestesiol. 2022 Jan-Feb;88(1-2):82-84. 2. Erika L.R., Ekman P. What the face reveals. Basic and applied studies of spontaneous expression using the facial action coding system (FACS). Oxford University Press. 2020

**Table 1 (abstract A166).** Rehabilitator-doctor

	YES	NO
Did you understand the clinical news?	74%	26%
Did you have enough time to ask questions?	18%	82%
Did the doctor seem available to you?	56%	44%
Do you remember what you were told?	70%	30%
What emotion did you see on the doctor's face?	Impotence	86%
	Indifference	67%
	Rush	71%
	I don't know	81%

**Table 2 (abstract A166).** Intensivist-doctor

	YES	NO
Did you understand the clinical news?	65%	35%
Did you have enough time to ask questions?	16%	84%
Did the doctor seem available to you?	52%	48%
Do you remember what you were told?	71%	29%
What emotion did you see on the doctor's face?	Impotence	75%
	Indifference	78%
	Rush	69%
	I don't know	85%

### A167. The unresolved problem of accidental ventilation circuit disconnections: let’s not hide behind a finger

#### Bardacci Y.^1^, Baldassini Rodriguez S.^1^, Rasero L.^2^, Bambi S.^3^

##### ^1^Azienda Ospedaliero Universitaria Careggi ~ Firenze ~ Italy, ^2^Dipartimento Scienze Della Salute Umana - Unifi ~ Firenze ~ Italy, ^[3]^~ Firenze ~ Italy

###### **Correspondence:** Bardacci Y.

**BACKGROUND**: In the Intensive Care Unit (ICU) setting, healthcare workers often are chellanged by accidental disconnections of the mechanical ventilation (MV) breathing circuits.. The complications that can arise from a circuit disconnection are changes in vital signs and coughing episodes causing discomfort and stress in the patient and potential problems related to the loss of positive end expiratory pressure (PEEP). Among the changes in vital signs (VS) accidental disconnections can cause increased heart rate (HR), blood pressure (BP), respiratory rate (RR), and a reduction of peripheral oxygen saturation (SpO2). The aim of this study was to reveal the rate of accidental disconnections from breathing circuits, identifying the causes, t,the parts of breathing circuits that are interested concerned, ttime of disconnections and the interventions carried out during and after these events.

**MATERIALS AND METHODS**: A prospective observational study was performed on a sample of 72 consecutive patients admitted to the ICU of Careggi University Hospital in Florencefrom November 2018 to February 2019. The disconnection events were recorded on a speficic data sheet. The data sheet is divided into a patient’s information general section, the characteristics of the disconnection events, and a specific section for the recording of VS a.

**RESULTS**: 106 disconnection events were recorded. 48 events (45%) did not cause changes in VS while in the other 58 events (55%) some problems occurred. 87/106 events (82%) were due to maneuvers carried out by healthcare operatorswhile the remaining 19 (18%) were caused by the patients. Among the 87 events caused by operators, 39 events (45%) were generated by nursing inteerventions; in particular mobilization and postural changes caused a total of 19 detachments from breathing circuit (22%). In 31/106 cases (29%), nurses had to perform post-disconnection maneuvers to restore VS. The actions to resolve the disconnection, the post-disconnection interventions and the clinical consequencesresulting from the disconnection events were also recorded. Relevant the section inherent to the vital signs (*Figure 1,2,3,4*) that sees both the HR and the RR altered after the disconnection as well as the BP and the SpO2 that change substantially to the occurrence of disconnection events.

**DISCUSSION**: These results allowed us to identify the most frequent typologies, times and disconnection points as well as the influence of these events on patients’VS. Although the disconnections from breathing circuits are still considered as low (or not) impact events, they actually exert influence on physiological functions withpossible negative consequences for patients.

**CONCLUSIONS**: Studies on the physiopathological impact of disconnections should be designed and performed in future desirable. The scientific community should be attentive to this typology of events. This study has some limitations, but highlighted many points of improvement of patients’ care, to prevent and limit , accidental disconnection events.

1. Bambi S, Rodriguez SB, Lumini E, Lucchini A, Rasero L. [Unplanned extubations in adult intensive care units: an update]. Assist Inferm Ric. 2015 Jan-Mar; 34(1):21-9.
2. Bouza C, Garcia E, Diaz M, Segovia E, Rodriguez I. Unplanned extubation in orally intubated medical patients in the intensive care unit: a prospective cohort study. Heart Lung. 2007 Jul-Aug;36(4):270-6.
3. Bambi S. *Le estubazioni non pianificate nelle terapie intensive: quali implicazioni per l’assistenza infermieristica?* Assistenza infermieristica e ricerca, 23 (1). pp. 36-47.
4. Janowski MJ. Accidental disconnections from breathing systems, what FDA found--and what you can do about it. Am J Nurs. 1984 Feb;84(2):241-4.
5. Larsen R. La respirazione artificiale. Basi e pratica. 2nd ed.: Springer; 2011.
6. Torre R. Paziente critico nelle patologie respiratorie. 1st ed. Milano: Poletto; 2006.
7. da Silva PS, Fonseca MC. Unplanned endotracheal extubations in the intensive care unit: systematic review, critical appraisal, and evidence-based recommendations. Anesth Analg 2012 May;114(5):1003-1014.
8. Kiekkas P, Aretha D, Panteli E, Baltopoulos GI, Filos KS. Unplanned extubation in critically ill adults: clinical review. Nurs Crit Care 2013 May;18(3):123-134.
9. Chiang A, Lee KC, Lee JC, Wie CH. Effectiveness of a continuous quality improvement program aiming to reduce unplanned extubation: a prospective study. Intensive Care Med 1996; 22: 1269-71.
10. Maguire GP, DeLorenzo LJ, Moggio RA. Unplanned extubation in the intensive care unit: a quality-ofcare concern. Crit Care Nurs Q 1994; 17: 40-7.
11. Curry K, Cobb S, Kutash M, Diggs C. Characteristics associated with unplanned extubations in a surgical intensive care unit. Am J Crit Care 2008 Jan;17(1):45-51; quiz 52.
12. Tanios MA, Epstein SK, Livelo J, Teres D. Can we identify patients at high risk for unplanned extubation? A large-scale multidisciplinary survey. Respir Care 2010 May;55(5):561-568.
13. Chang LY, Wang KW, Chao YF. Influence of physical restraint on unplanned extubation of adult intensive care patients: a case-control study. Am J Crit Care 2008 Sep;17(5):408-15; quiz 416.
14. de Groot RI, Dekkers OM, Herold IH, de Jonge E, Arbous MS. Risk factors and outcomes after unplanned extubations on the ICU: a case-control study. Crit Care 2011;15(1):R19.
15. Tanios M, Epstein S, Grzeskowiak M, Nguyen HM, Park H, Leo J. Influence of sedation strategies on unplanned extubation in a mixed intensive care unit. Am J Crit Care 2014 Jul;23(4):306-14; quiz 315.
16. Gardner A, Hughes D, Cook R, Henson R, Osborne S, Gardner G. Best practice in stabilisation of oral endotracheal tubes: a systematic review. Aust Crit Care 2005 Nov;18(4):158, 160-5.
17. Jarachovic M, Mason M, Kerber K, McNett M. The role of standardized protocols in unplanned extubations in a medical intensive care unit. Am J Crit Care 2011 Jul;20(4):304-11; quiz 312.
18. Aiken LH, Sloane DM, Bruyneel L, Van den Heede K, Sermeus W; RN4CAST Consortium. Nurses' reports of working conditions and hospital quality of care in 12 countries in Europe. Int J Nurs Stud 2013;50:143-53.
19. Kane RL, Shamliyan TA, Mueller C, Duval S, Wilt TJ. The association of registered nurse staffing levels and patient outcomes: systematic review and meta-analy- sis. Med Care 2007;45:1195-204.
20. A.P.Adams, Breathing system disconnections, British Journal of Anaesthesia 1994; 73: 46-54.
21. *Gallagher A. Ethical issues in patient restraint. Nursing Times; 2011, 107, 9: 18-20.*
22. *Alberti F. La Contenzione fisica nei reparti ospedalieri: strumento terapeutico? Considerazioni etiche, medico-legali e giuridiche. Professione. Cultura e pratica del medico d’oggi; 2007, 7: 10-14.*
23. *Gasparini S, Nosenzo M, L’endoscopia bronchiale, Associazione Italiana Pneumologi Ospedalieri, 2014, 5-77.*
24. *Biondino A., Scagnetti T. Assistenza Respiratoria Domiciliare – Il paziente adulto tracheostomizzato in ventilazione meccanica a lungo termine. Ed Universitalia, 2013.*

**Table 1 (abstract A167).**
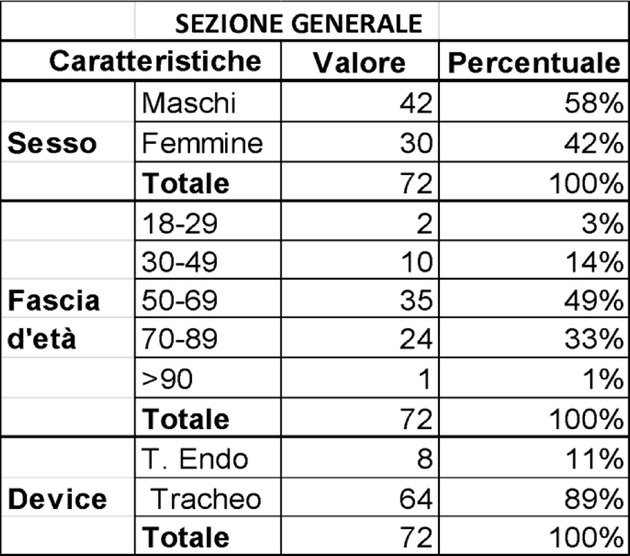
Sezione Generale, caratteristiche dei pazienti

**Table 2 (abstract A167).**
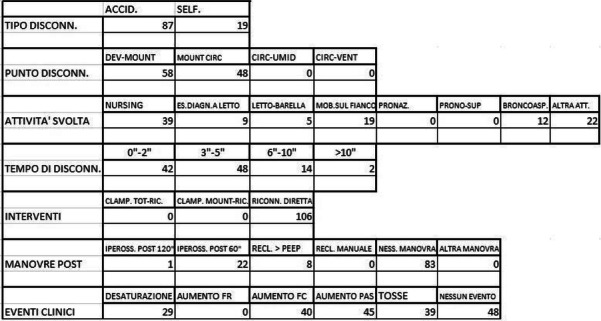
Sezione specifica, statistiche delle disconnessioni non programmate

**Table 3 (abstract A167).**
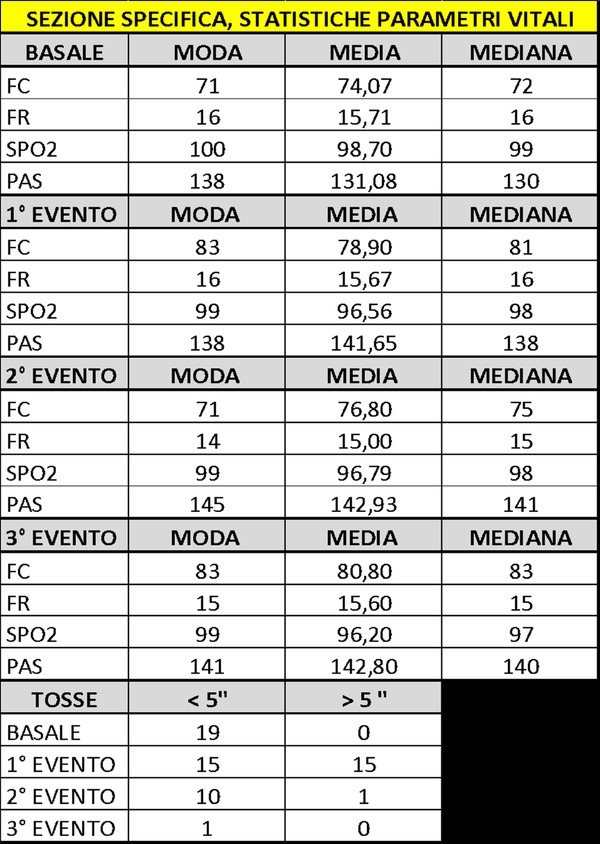
Sezione specifica, statistiche parametri vitali

**Fig. 1 (abstract A167).**
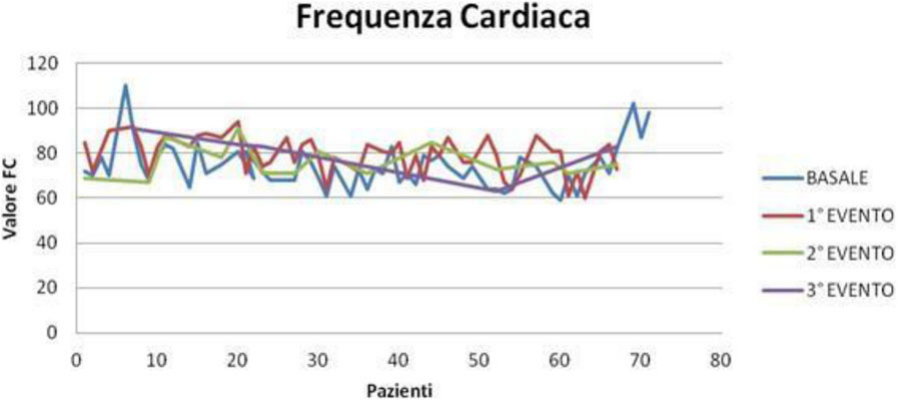
See text for description

**Fig. 2 (abstract A167).**
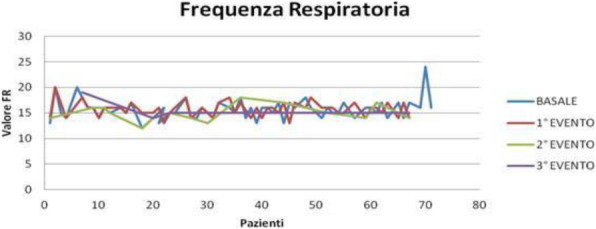
See text for description

**Fig. 3 (abstract A167).**
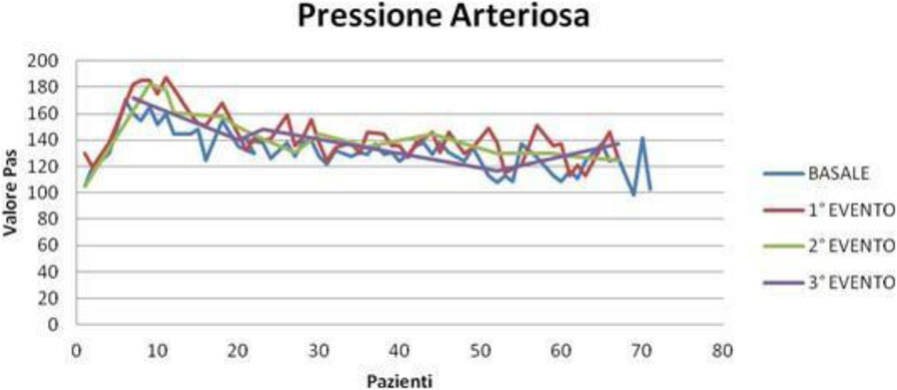
See text for description

**Fig. 4 (abstract A167).**
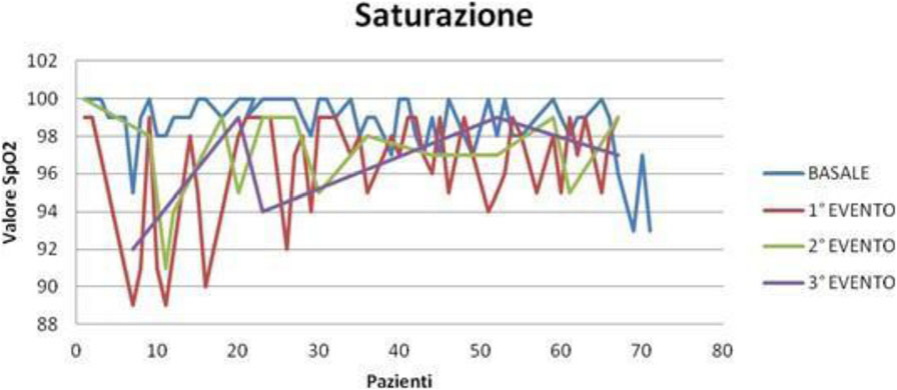
See text for description

### A168. Routine use of videolaryngoscopy as a clinical risk mitigator in tracheal intubation of adult and pediatric patients: the risk management experience of e. profili hospital in Fabriano

#### Pisello E.^1^, Ciuffreda M.^2^, Silvestri J.^1^, Basso U.W.^1^, Caimmi M.^2^, Galante D.M.G.^3^, Piangatelli C.^2^

##### ^1^Università Politecnica delle Marche ~ Ancona ~ Italy, ^2^Anaesthesia, Resuscitation, Intensive Care and Pain Management Unit, ASUR Marche AV2 ~ Fabriano ~ Italy, ^3^Anaesthesia, Resuscitation, Intensive Care and Pain Management Unit ~ Cerignola ~ Italy

###### **Correspondence:** Pisello E.

***BACKGROUND*:** Airway management in tracheal intubation represents one of the crucial issues in current anaesthesiological practice, in which risk management plays an essential role. At the moment, videolaryngoscopy is considered the main technique to facilitate tracheal intubation and reduce its complications. In the operating block of Profili Hospital in Fabriano videolaryingoscopy has been the routine practice since November 2021.

***OBJECTIVES:*** Evaluation of the routine use of videolaryngoscopy as a mitigator of clinical risk and unexpected difficulties occurring during tracheal intubation in the adult and pediatric surgical setting. Comparison between Fremantle Videolaryngoscope Scoring System, parameters and preoperative scores predicting difficult intubation such as El-Ganzouri Risk Index in adult patients and Colorado Pediatric Airway Score in the pediatric ones.

***METHODS:*** Preliminary prospective observational study of 368 patients (304 adults and 64 children) undergoing surgery, assessed through the previously mentioned scores and classifications (Table 1.)

***RESULTS AND DISCUSSION:***

- First attempt tracheal intubation achieved in 94,73% of adults and 93,75% of children, without using any additional device.
- 17,43% of adults had an El Ganzouri Risk Index predicting a difficult intubation, but showed a complete or partial videolaryngoscopic view in 77,36% and 20,75% respectively; a first attempt tracheal intubation was obtained in 92,45% of cases.
- 82,56% of adults had an El Ganzouri Risk Index predicting an easy intubation, but showed none or partial videolaryngoscopic view in 0,39% and 15,93% respectively; tracheal intubation required a second attempt in 4,78% of cases.
- 9,37% of children had a Colorado Pediatric Airway Score predicting a difficult intubation, but showed a complete videolaryngoscopic view in 100% of cases; a first attempt tracheal intubation was obtained in 66,66% of cases.
- 90,62% of children had a Colorado Pediatric Airway Score predicting an easy intubation, but showed a partial videolaryngoscopic view in 3,44% of cases; tracheal intubation required a second attempt in 3,44% of cases.
- No intubation was impossible, regardless of the difficulties predicted by preoperative parameters and scores and the videolaryngoscopic view obtained.
- All difficult tracheal intubations not predicted by parameters and scores were successfully performed (16,33% and 3,44% in adults and children, respectively).

***CONCLUSIONS:*** Routinary use of videolaryngoscopy has encouraged and optimised teamwork, including training; reduced the time spent in the operating room and the use of additional devices for managing difficult airways; completely cut out clinical risk of difficult intubations, eliminating impossible ones and their clinical and organisational consequences, especially in pediatric patients, a particularly high risk category in airway management; made it possible to overcome the limits of individual parameters and difficult intubation predictor scores such as the El Ganzouri Risk index and the Colorado Pediatric Airway Score, allowing us to easily manage any unexpected difficult airway; permitted the hypothesis of abandoning, for the near future, scores and parameters predicting difficult intubation, with huge benefits on the time spent in the preoperative evaluation of the surgical patient.

**Table 1 (abstract A168).** Results

Adult Patients			Pediatric Patients		
VARIABLE	EVALUATION	N° (%)	VARIABLE	EVALUATION	N° (%)
Gender	Male Female	183 (60,19) 121 (39,80)	Gender	Male Female	35 (54,68) 29 (45,31)
Age (years)	16-60 > 60	153 (50,32) 151 (49,67)	Age (years)	3-6 7-12 13-16	39 (60,93) 10 (15,62) 15 (23,43)
BMI	< 30 >= 30	251 (82,56) 53 (17,43)	BMI	< 30 >= 30	63 (98,43) 1 (1,56)
Neck circumference (cm)	< 40 >= 40	122 (40,13) 182 (59,86)	Neck circumference (cm)	< 30 >= 30	35 (54,68) 29 (45,31)
Mouth opening (cm)	< 4 >= 4	41 (13,48) 263 (86,51)	Chin characteristics	Normal size Small, moderately hypoplastic Markedly recessive Extremely hypoplastic	59 (92,18) 4 (6,25%) 1 (1,56) 0 (0)
Thyromental distance (cm)	< 6 6 -6,5 > 6,5	4 (1,31) 56 (18,42) 244 (80,26)	Interdental distance between the front teeth (cm)	< 1 1-2 2-4 > 4	0 (0) 0 (0) 6 (9,37) 58 (90,62)
Mallampati class	I II III-IV	112 (36,84) 136 (44,73) 56 (18,42)	History of OTI and OSAS	Previous OTI not difficult Never OTI, no OSAS OTI difficult or OSAS Failed OTI, emergency tracheostomy or sleeping prone	5 (7,81) 51 (79,68) 8 (12,5) 0 (0)
Neck movement (degrees)	< 80 80-90 > 90	12 (3,94) 90 (29,6) 202 (66,44)	Uvula	Tip visible Partially visible Not visible, only soft palate Soft palate not visible at all	58 (90,62) 6 (9,37) 0 (0) 0 (0)
Possibility of mandibular protrusion	Yes No	275 (90,46) 29 (9,53)	Head flexion-extension (degrees)	> 120 60-120 30-60 < 30	63 (98,43) 1 (1,56) 0 (0) 0 (0)
Body weight class (Kg)	< 90 90-110 > 110	245 (80,59) 50 (16,44) 9 (2,96)	Modifiers	Prominent front teeth Macroglossia Extreme obesity Mucopolysaccharidosis	5 (7,81) 2 (3,12) 1 (1,56) 0 (0)
History of difficult intubation	Uncertain None Certain	10 (3,28) 293 (96,38) 1 (0,32)	Colorado Pediatric Airway Score (COPUR)	5-7 8-10 12 14 16 > 16	58 (90,62) 5 (7,81) 1 (1,56) 0 (0) 0 (0) 0 (0)
El Ganzouri risk index	< 4 >= 4	251 (82,56%) 53 (17,43%)	View of the vocal cords with videolaryngoscopy	Total Partial None	62 (96,87) 2 (3,12) 0 (0)
View of the vocal cords with videolaryngoscopy	Total Partial None	251 (82,56) 51 (16,77) 2 (0,65)	Intubation difficulty	Easy (OTI at first attempt) Difficult (IOT on the second attempt or additional devices required) Impossible	60 (93,75) 4 (6,25) 0 (0)
Intubation difficulty	Easy (OTI at the first attempt) Difficult (OTI at the second attempt or required additional devices) Impossible	288 (94,73) 16 (5,26) 0 (0)	Videolaryngoscope and blade size	Medcaptain blade 1 Medcaptain blade 2 Medcaptain blade 3 Medcaptain blade 3D Medcaptain blade 4	13 (20,31) 31 (48,43) 15 (23,43) 1 (1,56) 4 (6,25)
Videolaryngoscope and blade size	Medcaptain blade 3 Medcaptain blade 3D Medcaptain blade 4	63 (20,72) 30 (9,86) 211 (69,40)			

## Simulazione

### A169. Post-graduate training on advanced trauma life support through high-fidelity simulation

#### Del Pozo A.C., Ebm Cecconi C., Fusili N., Pugliese L., Calabrò L., Brusa S.

##### Humanitas University Simulation Center ~ Milano ~ Italia

###### **Correspondence:** Brusa S.

Introduction

Unified critical care post-graduate training within residency programs is of vital importance for the correct patient management and patient safety. We created a high-fidelity simulation program of monthly activities for the training of residents of all the different specialties of our hospital, designed to educate them on core topics and procedural skills related to the critically polytrauma patients. The aims of our study were to monitor residents' knowledge and efficacy of the activity proposed using the latest guidelines for the management of critically polytrauma emergencies; to evaluate the effectiveness of the take home message and to evaluate residents’ level of satisfaction after their simulation performances.

Description

……. medical residents from twenty-one specialties participated in the activities. The activities consisted in the running of high-fidelity simulation clinical scenarios dedicated to polytrauma patients. To evaluate the baseline knowledge of the participants on these topics, a pre-simulation test was electronically delivered to participants before the activity. The same test was re proposed after the activity was completed. Comparison of the outcomes of the pre- and post-activity tests was done to evaluate simulation-based medical education impact on

residents’ knowledge and effectiveness of the take home messages. Finally, a survey to get participants feedback was administered to evaluate their level of satisfaction with the proposed activity. The instrument was validated by an expert, and the reliability calculated for each item. Responses were measured on 5-point Likert-scale items.

Results

The media of the correct answer in the pre-activity test was 7.65 while it was 9.26 in the post-activity test. Comparison between the media of the pre-test and the post-test was very significant (P= 0,000156). Residents’ satisfaction level express in the survey feedback was very satisfactory, expressing all of them their interest in having more high-fidelity training during their training. Participants underlined their satisfaction regarding the acquired knowledge, the possibility of applying non-technical skills during the activity, the level of satisfaction was 4.6/5 regarding the possibility offered of a real multidisciplinary training. Participants’ level of achievement of goals was 4.7/5.

Discussion

High-fidelity simulation is a potent method for multidisciplinary training and education on Advanced Trauma life support topics for medical residents. As a method, the high-fidelity simulation resulted in a high participants’ satisfaction level and showed a very significant potential for the acquirement of knowledge in the post graduate learning.

### A170. The rationality of multidisciplinary post-graduate training through high-fidelity simulation

#### Del Pozo A.C., Ebm Cecconi C., Pugliese L., Calabrò L., Brusa S.

##### Humanitas University, Simulation Center ~ Milano ~ Italia

###### **Correspondence:** Brusa S.

Introduction

In November 2021 more than 18000 new Medical Residents were incorporated to the Italian medical system, a considerable number after years of getting into specialty was almost a chimera.

Inscriptions to the Medicine Schools increased by the 21% while residency positions have shown an increase of 150% since 2016.

On the other hand, the new mode of access to the medical residencies is through a single ranking, meaning that depending on the ranking candidate’s position, they are allocated to a hospital and a specialization for which, sometimes, they have not had previous inexperience.

Hence there is a need to rethink the training of postgraduate doctors, with the aim of facilitating them the learning of a wide variety of clinical scenarios reinforcing patient outcomes and safety.

The use of high-fidelity simulation learning method is recognized for having a high impact in the learning process among trainees through experiential learning with reflection on action. Unified critical care training within residency programs is a necessity. We develop a high-fidelity simulation-based annual curriculum designed to educate residents of all specialties on core topics and procedural skills, in a transversal manner among all disciplines caring for critically ill patients. The main goal of this activity was to address the need for consistent, safe, efficient, and unified critical care training within post-graduate medical education.

Material and methods:

Residents from twenty-two adult specialties were invited to this annual program. Participation to the monthly

appointment was not mandatory. Scenarios covered a broad array of complex critical care topics facing all adult specialties and reinforced important system-specific initiatives. To analyze participants’ clinical performance perception, self-reported confidence, procedural and communication skills, overall satisfaction, and interdisciplinary team working capacity we use an anonymous, non-mandatory survey that was delivered after the competition of the activities.

Results:

We obtained 258 answers. The survey showed an overall satisfaction level of 4.8/5. Residents stated that simulation activity allowed them to analyze their own behavior and that their knowledge was challenged by the curriculum (4.5/5). They acquired confidence in patient management strategies (4.3/5), simulation activities stimulated their non-technical skills as situation awareness, team working and decision making and communication (4,5/5). All residents expressed their interest in having more simulation teaching during their residency programs.

Discussion:

Purposeful focus on curricular development that integrates basic, clinical, and procedural content, while promoting the development of interdisciplinary relationships and the practice of critical thinking skills, is vital for successful postgraduate education and patient safety.

This program required a vast number of resources and commitment for the different disciplines. Each residency program embraced this curriculum by enabling their learners to attend the activities, by proposing the topics of their own interest, by the participation during the ideation of the clinical scenarios and by the involvement of their seniors’ tutors during the debriefings. In an era of limited time for devoted bedside teaching and the requirement of maximum efforts to ensure patient safety, the development and implementation of this curriculum has filled a need within our system for unified resident education.

### A171. Impact of simulation-based post-graduate training on leadership and teamworking skills during in-hospital emergencies

#### Ebm C.^1^, Del Pozo A.C.^1^, Brusa S.^1^, Pugliese L.^2^, Calabro L.^2^

##### ^1^Humanitas University ~ Milano ~ Italia, ^2^ICH Humanitas Research Hospital ~ Milano ~ Italia

###### **Correspondence:** Ebm C.

Introduction: Dealing with in-hospital emergencies presents many cognitive and system challenges, and teamwork is an essential functional component to manage time-pressured, critical and rapidly changing tasks inherited during such high-risk, low probability events. Anesthetists attending medical emergencies often resume the role of the team leader, working with a diverse set of often “previously-unknown” team members. In such situations, maximizing patient safety and reducing medial errors depends not only upon technical expertise and individual clinical skills, but also on variety of non-technical skills such as teamwork, decision making and decisive leadership within the group. Simulating high-risk, low probability events may have the ability to recognize and address structural and cognitive bias before applying them in the real world. Our objective of this study was to evaluate the impact of an interdisciplinary simulation-based curriculum on acquired confidence level in working and collaborating as a team during in-hospital emergencies.

Methodology: We created simulation-based events with the objective to train residents in recognizing and managing common and rare emergencies. Transversal topics such as management of the dyspneic patient or the septic patient were chosen to encourage participants in using various perspectives and approaches when collaboratively solving the clinical case. We used high-fidelity simulators in a staged room to accurately replicate the hospital setting and increase realisms. After the events, participants were asked to self-evaluate the useability, transferability of the course, as well as their acquired confidence in decision making and leadership, via a 5-point Likert scale.

Results: During six events, 133 residents from thirteen medical specialties participated to interactively solve twelve clinical emergencies. The overall satisfaction level was remarkably high (4,8/5), with appreciation for interdisciplinary work (4,9/5) scaling highest. Residents positively evaluated their acquired ability for teamwork (4,7/5), and decision making (4,7/5). 95% of all respondents stressed the importance of including simulation-based training during the medical residency program (4,9/5).

Conclusion: Our study documents the positive effect of an interdisciplinary, simulation-based training curriculum on the learners’ ability to collaboratively deal with medical emergencies. Simulation provides an effective way to increase the capacity to work in a team, make decisions, jointly solve clinical emergencies and as such, leads to a high satisfaction level among young residents. Collaborative training involving an interdisciplinary team seems to be a save and enjoyable alternative to the classic “learning on the patient” approach.

**Fig. 1 (abstract A171).**
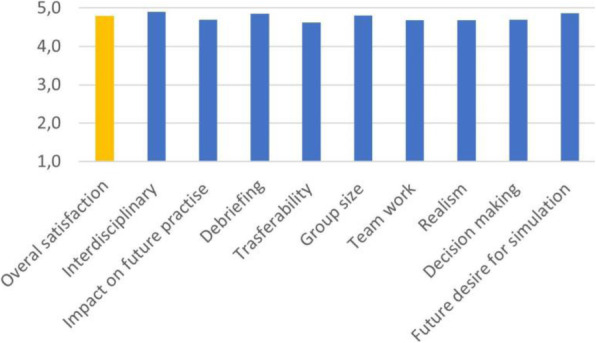
See text for description

### A172. Effectiveness of a remote simulation training using ventsim© versus standard teaching to increase knowledge on mechanical ventilation among trainees: a randomized controlled trial

#### Ippolito M., Simone B., Spinuzza E., Catania T., Ingoglia G., Milazzo M., Raineri S.M., Giarratano A., Gregoretti C., Cortegiani A.

##### Department of Surgical, Oncological and Oral Science (Di.Chir.On.S.), University of Palermo, Italy. ~ Palermo ~ Italia

###### **Correspondence:** Ippolito M.

Introduction: Training is essential to deliver safe mechanical ventilation. Free or open sources simulators are rare, and they often have old technical requirements limiting their use in current educational programs. VentSim© is a free online software, simulating basic modes of mechanical ventilation and patient - ventilator interaction. The aim of this trial was to assess the effectiveness of a rapid remote simulation-based training using VentSim© in association with remote standard teaching versus remote standard teaching alone to increase knowledge and skills on mechanical ventilation among residents in anesthesia and intensive care.

Materials and methods: For the purpose of this single centre, randomized controlled trial, all the trainees in Anesthesia, Intensive Care and Pain Medicine at University of Palermo were invited to participate and voluntarily randomized to receive a four-hours remote simulation-based training using VentSim©, in association with remote standard teaching, or remote standard teaching alone. The primary outcome was the number of correct answers to a 50 multiple choice questionnaire. Participants’ satisfaction was also assessed through a 5-points Likert scale agreement on 5 sentences. The number of correct answers (primary outcome) and overall agreement on satisfaction statements (additional outcome) in the two groups were compared using t- test for independent means, if normal distribution confirmed through Shapiro-Wilk test. Mann Whitney U test was used in case of non-normal distribution of the data. Statistical significance was accepted at p-value <0.05 and all tests were 2-tailed.

Results: A total of 183 residents were included in the study; of which 91 were randomized to intervention and 92 to control group. The residents randomized to the intervention group were 53% female and had a median age of 29 years [27-30]. Those randomized to the control group were 64% female and had a median age of 28 years [27-30]. The two groups were composed of 34% participants who had already worked in ICU for at least three months before this educational program, and 66% who did not, as per stratification of randomization. The outcomes were analyzed on a total of 148 residents who completed the final questionnaire. The rate of lost to follow up was 19%. The number of correct answers was similar between the two groups (28.6 ± 8.6 vs. 27.6 ± 9; P= 0.49). The intervention group rated 5 [4-5] the utility of integrating simulation in routine training of residents. No significant effect for the intervention was found in the sub-analyses on question categories and in the subgroup analysis according to previous clinical experience in ICU.

Conclusions: A remote simulation-based course on mechanical ventilation with VentSim© did not significantly improve the competencies of trainees in anaesthesia and intensive care, in comparison with remote standard teaching alone, at the end of a short educational cycle. Further studies should assess the efficacy of face-to-face simulation-based educational program on mechanical ventilation using VentSim©, also evaluating the best numerosity of groups of residents to optimize such learning experiences.

**Fig. 1 (abstract A172).**
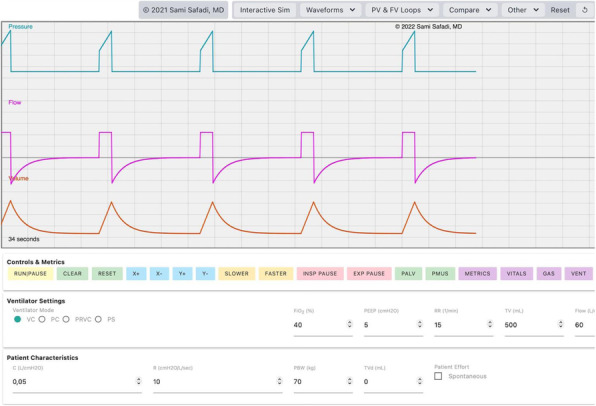
See text for description

### A173. Safety project - simulation approach for education and training in emergency

#### Caporusso R.R.^1^, Mirabella L.^1^, Barberio F.^1^, De Pascale G.^1^, Ferrando C.^2^, Filipescu D.^3^, Lazarovici M.^4^, Dieckman P.^5^, Cinnella G.^1^, Colantuono F.^1^, Gomez L.^2^, Ibanez C.^2^, Perdomo J.M.^2^, Darriba S.^2^, Stefan M.^3^, Florescu C.^3^, Cornel R.^3^, Grujic K.^4^, Normand C.^5^, Stomer U.E.^5^, Gulsrud T.O.^5^, Vatland N.^5^, Guadagni A.^6^, Pasta G.^6^, Scrocco A.^7^, Danesi L.^8^, Callero N.^8^, Drabauer L.^9^, Flores R.^10^, Santa A.^10^, Mendes S.^10^, Pinto P.^10^

##### ^1^Università degli Studi di Foggia, Italia, ^2^University Clinic Hospital of Barcelona Study Group, Spain, ^3^“Prof. Dr. CC. Iliescu” Emergency Institute for Cardiovascular Diseases study group, Romania, ^4^Ludwig-Maximilians University of Munich Study Group, Germany, ^5^University of Stavanger Study Group; Norway, ^6^ValueDO, Italy, ^7^Infotech, Italy, ^8^Laerdal, Italy, ^9^Alpha Medical Concepts, Austria, ^10^Take the Wind, Portugal

###### **Correspondence:** Caporusso R.R.

*SAFETY project Study Group3

The current teaching method, based on the "study, look, do, teach" algorithm, lacks in safety for the healthcare staff and patients. Healthcare staff (as doctor and nurses) are catapulted from university classes to wards, without the necessary procedural expertise to manage patients and situations. In this way the errors, usually present in the learning process, directly affect the patients.

Simulation-based healthcare training can be a valuable tool for better clinical practice; it provides a safe, controlled environment in which problem-based learning is developed and competences, technical and non-technical skills, are practiced in high-standard

The SAFETY (Simulation Approach for Emergency Training in emergencY) Project, selected among the 216 and financed by the European Erasmus + Program, aims to renew the university training offer in the field of Emergency Medicine, with the application of the teaching method in simulation, using both physical tools (eg mannequins) and virtual patients (eg simulated patient).

The five European universities (pic.1), partners of the project, and the companies that develop tools suitable for simulation, are working together to design new teaching courses composed of theoretical and practical modules, to be included in university programs (pic.2). The theoretical module will be delivered in e-learning mode, while the practical module will consist in training with simulation tools. The practical module will also be recorded and attached to the e-learning output.

With the SAFETY Project it will be possible to standardize health education, improving the quality and efficiency of education and training in the Emergency Medical fields.

Furthermore, the objective of the SAFETY Project is to improve health management in Europe, regardless of each national health system, starting with education and teaching; providing the dissemination and implementation in health training of standardized protocols and high-level skills, allowing the creation of a European Training Network focused both on the acquisition of communication skills and non-technical behaviors, as well as technical skills, in order to prevent and reduce errors in healthcare.

The SAFETY project intends to develop a new training path in the field of Critical and Emergency Medicine using simulation as a teaching methodology.

The aims and objectives of this cooperation are:

- The standardization of teaching methodology in simulation and learning in the field of emergency medicine enhanced by technology;
- Standardization of the emergency team by clearly specifying the role of medical and paramedical personnel;
- To structure the debriefing phase of simulation training through tailor-made skills and abilities conveyed by a team of qualified and specialized psychologists;
- To improve the effectiveness in the use of simulation devices;
- To obtain open access and easy to replicate teaching material, through the guidelines provided;
- To increase the practical experience of recent graduates and therefore the quality of the training offered, in order to:
- reduce the transition costs of the time spent introducing a new member of the emergency staff who is not yet practiced (having mainly acquired theoretical training);
- Improve patient safety;
- Involve university partners in European networks for further cooperation in research and education.

**Fig. 1 (abstract A173).**
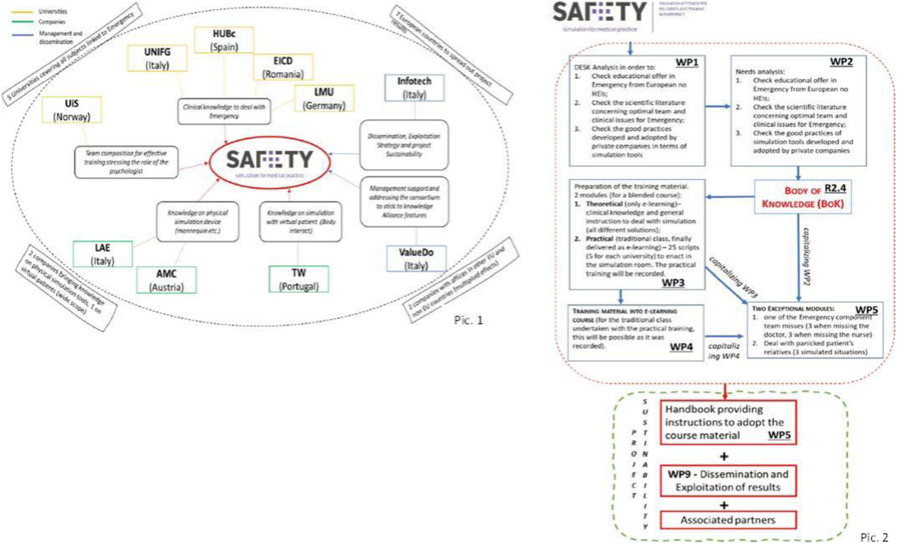
See text for description

### A174. Safety project - simulation approach for education and training in emergency WP2 analysis

#### Barberio F.^1^, Mirabella L.^1^, Caporusso R.R.^1^, Ferrando C.^2^, Filipescu D.^3^, Lazarovici M.^4^, Dieckman P.^5^, Cinnella G.^1^, Colantuono F.^1^, Gomez L.^2^, Ibanez C.^2^, Perdomo J.M.^2^, Darriba S.^2^, Stefan M.^3^, Florescu C.^3^, Cornel R.^3^, Grujic K.^4^, Normand C.^5^, Stomer U.E.^5^, Gulsrud T.O.^5^, Vatland N.^5^, Guadagni A.^6^, Pasta G.^6^, Scrocco A.^7^, Danesi L.^8^, Callero N.^8^, Drabauer L.^9^, Flores R.^10^, Santa A.^10^, Mendes S.^10^, Pinto P.^10^

##### ^1^Università degli Studi di Foggia Study Group, Italia, ^2^University Clinic Hospital of Barcelona Study Group, Spain, ^3^“Prof. Dr. CC. Iliescu” Emergency Institute for Cardiovascular Diseases study group, Romania, ^4^Ludwig-Maximilians University of Munich Study Group, Germany, ^5^University of Stavanger Study Group, Norway, ^6^ValueDo, Italy, ^7^Infotech, Italy, ^8^Laerdal, Italy, ^9^Alpha Medical Concepts. Austria, ^10^Take the Wind, Portugal

###### **Correspondence:** Barberio F.

SAFETY Project Study Group3

Background

The SAFETY (Simulation Approach for Emergency Training in emergencY) project, selected from 216 and funded by the European Erasmus+ Program, aims to renew educational offerings in the field of Emergency Medicine.

In Work Package 2, a survey was conducted on the state of the art of teaching in the field of critical and emergency medicine, and a needs analysis of students and academics. Two ad hoc questionnaires were used for this information, targeting students in the health professions (medicine, nursing, midwifery) and academics in the same field, to probe the same subject areas from the two respective perspectives.

Through the questionnaires, the state of the art of critical care and emergency medicine teaching, teaching deficiencies (theoretical and practical), and the willingness to explore topics concerning emergency medicine through the use of simulation courses were assessed.

Differences between the group of students and academics were also assessed.

Materials and Methods

The population was represented by:

- Students in the 5th and 6th year medical degree programs;
- Students from the 3rd year nursing and midwifery degree programs;
- Physicians in Specialty Training enrolled in the 1st and 2nd year;
- Academics (University Professors, Researchers, Tutors, Specialists in the field of Emergency Medicine).

Results

No.1464 Students and no.288 Academicians took part in the survey (Fig.1)

They were assessed (Fig. 2):

- University educational offerings (10 questions)
- Teamwork (15 questions)
- Health Emergency Management (6 questions)
- Technical Skills learning (8 questions)
- Learning in Simulation (1 question)

Students feel that they have acquired good skills in patient management, good training in teamwork and communication, and overall have a higher opinion of their own abilities than academics feel.

Both groups appreciate the e-learning mode.

Academics showed more awareness about the importance of working in teams and communicating effectively in emergencies, compared to students.

The main finding that emerges is the importance attached to the teaching method in simulation by both groups. Both students and academics rated Technical Skills and Non-Technical Skills learning above 4.5 (Likert scale) when developed in simulation.

Subsequent factor analysis showed that for students: as the amount of training received and preparation obtained increases, there is greater demand for e-learning courses; in Northern Europe there is greater demand for courses on Team Working; older students prefer courses on Non-Technical Skills, and students enrolled in Medicine courses require more courses based on emergency and team management.

These results confirm that higher quality training makes students more confident in their approach to patients.

With reference to academics, it was found that as years of experience increase, courses on emergency management and Technical Skills are considered more useful.

This highlights how academics tend to prefer the use of simulation for teaching Technical Skills and communication, which are crucial in managing an emergency.

**Fig. 1 (abstract A174).**
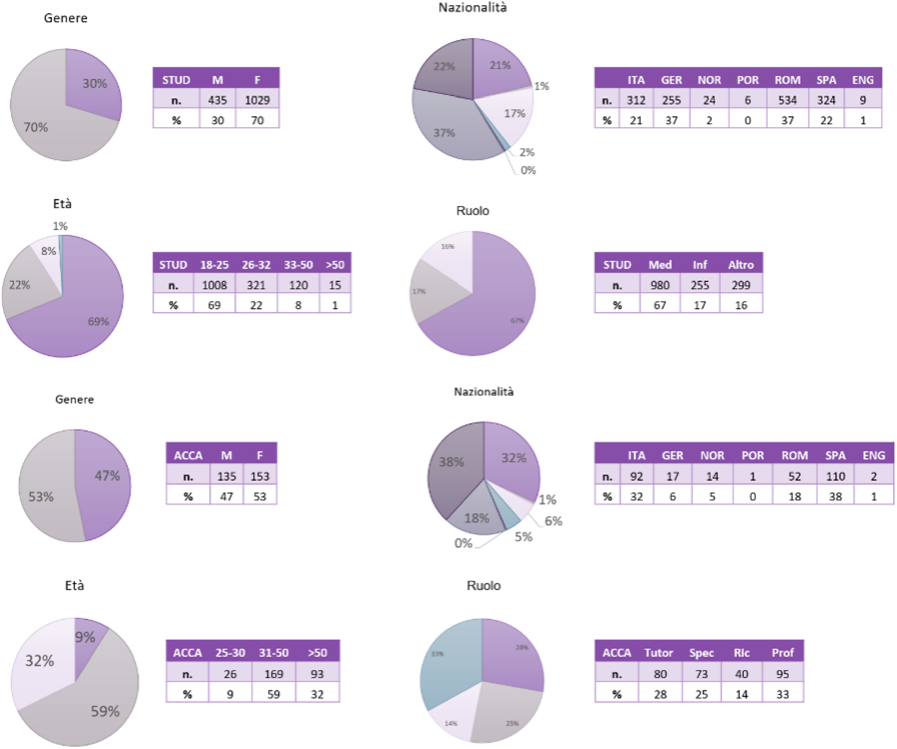
See text for description

**Fig. 2 (abstract A174).**
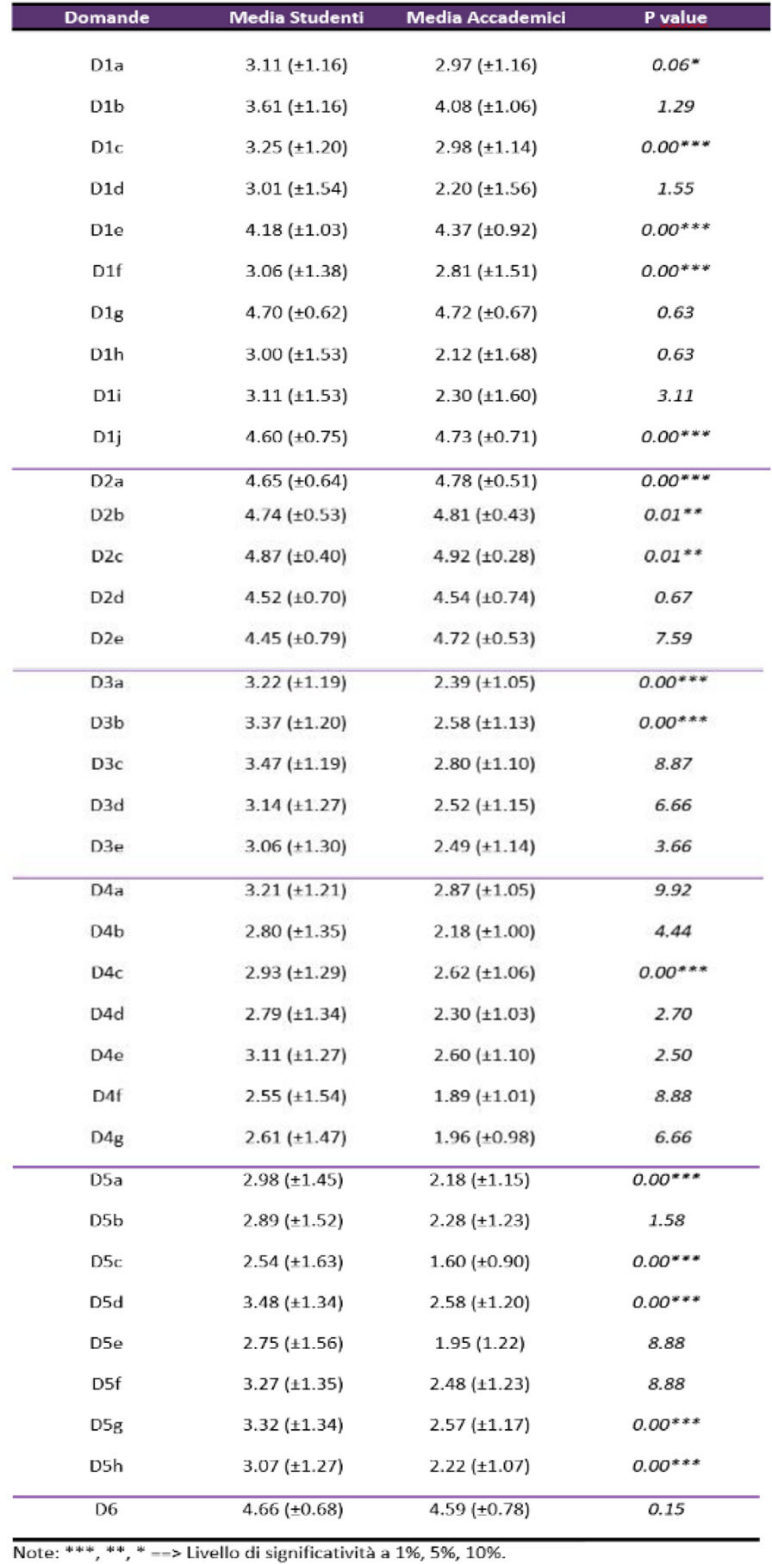
See text for description

## Tecniche invasive e interventistiche

### A175. Diagnostic accuracy, inter-rater variability and learning curve of three echocardiographic views for the tip location in adults: a prospective observational cohort study

#### Gori C.^4^, Cingolani E.^4^, Elisei D.^1^, Iacobone E.^1^, Pinelli F.^2^, La Greca A.^3^, Biasucci D.G.^5^

##### ^1^Ospedale di Macerata ~ Macerata ~ Italia, ^2^Azienda Ospedaliero-Universitaria Careggi ~ Firenze ~ Italia, ^3^Fondazione Policlinico Universitario A.Gemelli-IRCSS-Università Cattolica del Sacro Cuore ~ Roma ~ Italia, ^4^Azienda Ospedaliera San Camillo-Forlanini ~ Roma ~ Italia, ^5^Dipartimento di Scienze Cliniche e Medicina Traslazionale. Università degli Studi di Roma "Tor Vergata". ~ Roma ~ Italia

###### **Correspondence:** Gori C.

a) Introduction:

Intra-cavitary ECG (Ic-ECG) is the standard of care for tip location of central venous catheters. However, it is not applicable in patients with active pacemaker or supraventricular arrythmias. Ultrasound-based tip location has been proposed as alternative in these cases. Primary aim of our study is to evaluate accuracy and inter-rater variability of subcostal bicaval, subcostal 4-chambers and apical 4-chambers windows for tip location compared with Ic-ECG.

b) Methods:

Multicenter observational prospective cohort study conducted from June to October 2021. All adult patients undergone CVC insertion and given informed consent were included. The tip location was evaluated with transthoracic echocardiography and bubble test using three echocardiographic views compared with the Ic-ECG.

c) Results:

Fifteen patients were enrolled. Only one misplacement occurred. The subcostal 4-chambers view was the most easily obtained (87%), with a steep learning curve and the best agreement. Apical 4-chambers and subcostal bicaval views were obtained in 80% and 60% of cases respectively. The subcostal 4-chambers also showed the highest sensitivity. At least one of the three echocardiographic views was feasible in all patients enrolled.

d) Discussion & conclusions:

The TTE with bubble test is a safe and accurate bedside method for tip location. It can be easily applicable in most patients, even when IC-ECG cannot be used. The subcostal 4-chambers view seems to be the easiest, especially in ventilated patients. Further sampling will be needed to confirm these preliminary results and to investigate also intra-rater variability.

### A176. Measurement method for insertion of a peripherally inserted central catheters in a pronated patient

#### Longo F., Strumia A., Sammartini D., Sammartini E., Pascarella G., Costa F., Carassiti M., Cataldo R., Sarubbi D., Migliorelli S., Agrò F.E.

##### Unit of Anaesthesia, Intensive Care and Pain Management, Department of Medicine, Fondazione Policlinico Universitario Campus Bio-Medico ~ Roma ~ Italia

###### **Correspondence:** Sammartini D.

PICCs (Peripherically inserted central catheters) are amongst the most employed central venous catheters for medium/long term pharmacological treatments. During the insertion of a PICC, the length of the catheter is the most relevant aspect of the procedure. There exist several methods to estimate the exact length of the device. One of the most employed methods is the Peres rule, later modified by Pittiruti, that consists in measuring the catheter from the point of insertion to the jugulum and then adding 10cm if the venepuncture is performed on the right limb or 15cm in case of the left limb.

However, patients are not always in a supine position. During the pandemic, it has occurred to insert PICCs in patients in prone position due to the dysfunction of CVCs inserted after the pronation or to the accidental removal of CVCs during the pronation manoeuvers. In this case, since there are no reference points, measuring the length of the device can be a difficult procedure, and studies that examine appropriately this aspect have not yet released.

The purpose of our study is to find an anthropometric method, to be used in prone position, similar to the Peres one. We hypothesize that the jugulum corresponds to the T2 or T3 spinous process and therefore that an applicable method is the **place of insertion** - T2 spinous process plus 10 cm.

**Methods**. We have compared TC MPR reconstructions in sagittal in 50 patients aged 18-70 years and we have traced reference transverse lines from the top of the sternum manubrium in order to see the structure that posteriorly corresponds to the jugulum. Later on, we compared the length obtained with this procedure with that obtained following the modified Peres method.

**Results**. In most cases (45 cases over 50) the sternal border posteriorly corresponded to the T2 spinous process. From the comparison of these techniques, the length is, on average, 2cm in excess in relation to the one esteemed by the modified Peres method.

**Conclusions**. An anthropometric method that can be applied to a prone patient in order to esteem the exact length of a PICC, consists in measuring the distance between the exit site and the T2 spinous process, and then adding 8cm for right limb and 13cm for the left one. In order to validate our thesis, we hope for other studies of higher level of evidence.

**Fig. 1 (abstract A176).**
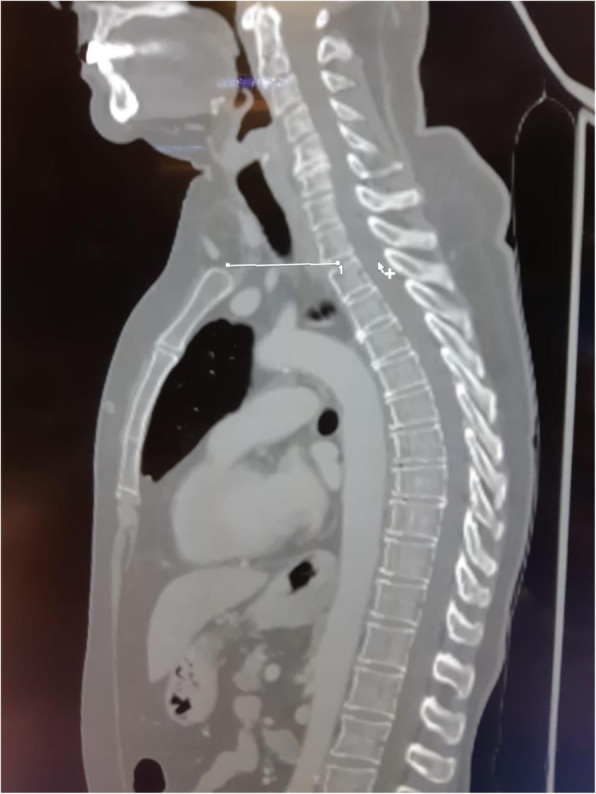
See text for description

**Fig. 2 (abstract A176).**
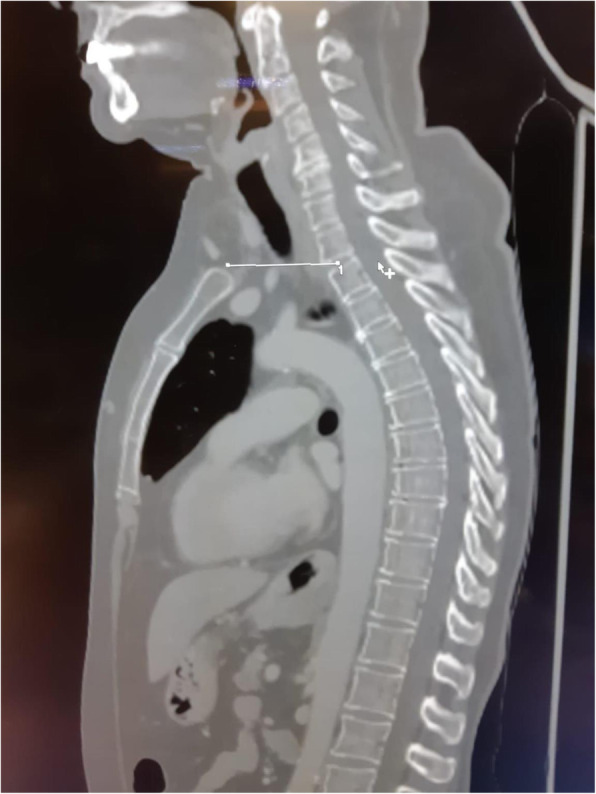
See text for description

### A177. Effect of electroacupuncture on brachial plexus post-traumatic neuralgia. a case report

#### Del Prete D., Riso C., Ferrone G., Piersanti A., Pitoni S., Antonicelli F., De Riso M., Rossi M.

##### Department of Anesthesia and Intensive Care, Fondazione Policlinico Universitario Agostino Gemelli IRCCS ~ Roma ~ Italia

###### **Correspondence:** Del Prete D.

**Background**

Brachial plexus injury is a serious peripheral nerve injury that severely disables upper limbs and affects patients' daily life and work Acupuncture and Electroacupuncture (EA) have traditionally been used to treat neuropathic pain. However, there is still lacking evidence as regard to their effects on pain following traumatic nerve and plexus lesions. Neurotmesis after brachial plexus injury also causes movement disorders of the denervated muscles and loss of sensory function in the skin.

**Case report**

A 51-year-old woman who reported left humeral fracture with severe brachial plexus injury, was proposed for EA after several failed drug therapies in the previous months. Before treatment, symptoms included severe pain with NRS 9/10 (Numeric Rating Score from 0 with no pain to 10 with the worst pain ever felt) and persistent tingling at the left hand 8/10. The nerve conduction studies performed ten months after the injury, showed the absence of sensory potential evoked from ulnar nerve and the median nerve, together with an amplitude reduction of compound muscle action potential (CMAP) registered from abductor pollicis (median nerve). We delivered acupuncture treatment once per week for six weeks, followed by a four-week acupuncture-free time, and then another six sessions of EA, again once a week. The primary endpoints were changes in NRS and EMG findings. Twenty-eight days after the first 6-weeks treatment the patient reported a gradual but significant improvement in terms of pain (4/10) and tingling (3/10). (Table 1). This physical well-being lasted up to the control at 6 months, without assuming any analgesic drug. Sensory nerve conduction examination, performed one month after the last acupuncture session, confirmed the absence of sensory potential evoked from median nerve (third digit). Interestingly, a sensory action potential was evoked by ulnar nerve (fifth finger), even if with reduced amplitude. Motor nerve conduction studies showed a CMAP amplitude reduction registered from abductor pollicis (median nerve) and abductor digiti minimi (ulnar nerve), which were reduced of 70% and 30%, respectively, when compared to the controlateral hand. In the second EMG, the examination involved more motor nerves than the first and fewer artifacts were visible on the sensory nerves than in the first exam. There was no involvement of the conduction velocities, while the appearance of the SAP (sensory nervous action potential) evoked by the ulnar nerve (stretch V finger-wrist) was detected.

**Conclusion**

Low-frequency and low-intensity EA could be effective for a significant and long lasting pain relief, joined to a nerve functional recovery based on EMG findings. However large trials with different electrical current modalities could clarify the role and mechanism of EA on peripheral nerve pain treatment. Informed consent to publish had been obtained by patient.

**References**

S Zhang et al. Electroacupuncture attenuates neuropathic pain after brachial plexus injury Neural Regen Res. 2014 Jul 15;9(14):1365-70.

**Table 1 (abstract A177).** Numeric Rating Scale (NRS) 0/10 and hand tingling (HT) 0/10 during the 1^st^ and 2^nd^ treatment. Absolute number showed before starting the session from the first session to the control visit 28 days after the end of treatment

**1**^**st**^	**Session 1**	**Session 2**	**Session 3**	**Session 4**	**Session 5**	**Session 6**	**Control**
**NRS**	09/10	08/10	06/10	06/10	07/10	05/10	04/10
**HT**	08/10	07/10	07/10	07/10	06/10	05/10	03/10
**2**^**ND**^	**Session 1**	**Session 2**	**Session 3**	**Session 4**	**Session 5**	**Session 6**	**Control**
**NRS**	06/10	04/10	04/10	03/10	03/10	03/10	03/10
**HT**	04/10	04/10	03/10	03/10	02/10	02/10	02/10

### A178. Quality of life in women with breast cancer undergoing neoadjuvant chemotherapy- comparison between picc and a new device, the PICC-port

#### Fundaró A., Morgantini C., Barbani F., Selmi V., Balsorano P., Villa G., Romagnoli S., Pinelli F.

##### Dipartimento di Scienze della Salute, Scuola di Specializzazione ARTID, Università di Firenze; Azienda Ospedaliero-Universitaria Careggi, Firenze ~ Firenze ~ Italia

###### **Correspondence:** Fundaró A.

Introduction

A safe and effective medium/long term vascular access is an essential part of the chemotherapeutic treatment in patients with breast cancer. Among vascular access both totally implanted venous access port (TIVAP) and PICC are routinely used devices for neoadjuvant chemotherapy.

While many studies were conducted to assess the rate of complications, very few studies - basically retrospective ones - aimed to evaluate the difference in terms of quality of life between the two types of access.

Purpose

To prospectively evaluate the quality of life in women with medium or long term vascular access and the rate of complications related to these devices in this population.

Methods

We prospectively recruited 198 women between May 2019 and November 2020 (102 PICC and 96 PICC PORT) in our center “Centro Accessi Vascolari” at Careggi, University Hospital of Florence, Italy. Indications to PICC placement was intravenous treatment for less than 6 months. Alternatively, if treatment was expected to be for longer than 6 months, a PICC-port was implanted.

The inclusion criteria were women with breast cancer with age > 18 years and indications to a medium- or long- term vascular device and need for chemotherapy.

The exclusion criterium was the denial of the patient.

This study was approved by our regional Ethical Commitment in May 2020.

Informed consent was obtained by all participants.

We collected records about the study population, their comorbidities, procedural data and complications.

In order to evaluate the aesthetics and the psychological burden of the device we submitted the QASSIC questionnaire to all patients enrolled at 12 months after the insertion.

Statistical analyses were performed with SPSS software (version 26).

Results

The two groups resulted comparable for age and BMI.

The overall score of device satisfaction was less in PICC group (p< 0.01);

VAS at the moment of the insertion was comparable in the groups. Thrombotic complication rates in PICC-port and PICC group were 3,13% and 2.94% respectively, and do not differ significatively.

No infection was recorded in the PICC group, whereas a 2,08% was registered in the PICC-port group (p>0.05). Hematomas (included inactive ones) were 11,4 % and 0.98% in PICC-port group and PICC group respectively (p 0.01).

Conclusions

PICC-ports are associated with less discomfort and pain compared to PICC, with comparable complications except for inactive hematomas.

Limitation of the study is the small sample size of patients enrolled. However, our results demonstrate a superiority of PICC-ports compared to PICCs in terms of quality of life in women with breast cancer undergoing neoadjuvant chemotherapy.

Bibliography

-Li Ma, Yueping Liu. Totally implantable venous access port systems and associated complications: A single-institution retrospective analysis of 2,996 breast cancer patients. Mol clin Oncol. 2016 Mar; 4(3): 456-460.

-Burbridge B., Chan I., Bryce R., Lim H., Stoneham G., Haggag H., Roh C. Satisfaction and Quality of Life Related to Chemotherapy with an Arm Port: A Pilot Study. CARJ. 2016;67:290–297.

### A179. Association between filtration fraction and sofa score improvement in septic patients performing CKRT with an acrylonitrile based membrane: an observational study

#### Fioccola A.^1^, Edo M.T.^1^, Gori A.^1^, Villa G.^2^, Ricci Z.^3^, Romagnoli S.^2^

##### ^1^Dipartimento di Scienze della Salute, Scuola di Specializzazione ARTID, Università di Firenze; Azienda Ospedaliero-Universitaria Careggi, Firenze ~ Firenze ~ Italia, ^2^Dipartimento di Scienze della Salute, sezione di Anestesiologia e Terapia Intensiva, Università di Firenze ~ Firenze ~ Italia, ^3^Dipartimento di Anestesia e Terapia Intensiva, Ospedale Pediatrico Meyer, Firenze ~ Firenze ~ Italia

###### **Correspondence:** Fioccola A.

BACKGROUND: Septic shock is a common syndrome in intensive care unit and the leading cause of acute kidney injury (AKI) and death or disability. Hypercytokinemia and high levels of endotoxin might have a role in the dysregulated host response to an infection that characterizes sepsis pathophysiology as cause of multiple organ failure. Over the years various adjuvant therapies have been developed to modulate the immune response to limit organ damage while treating sepsis. Among them, extracorporeal blood purification (EBP) therapies have been applied for over 30 years with conflicting results[1]. EBP is thought to play a role in the modulation of pathophysiology of sepsis by removing pathogens and associated molecular patterns by removing pathogen associated and damage associated molecular patterns (PAMPs and DAMPs). Different studies have shown the importance of several factors that can ameliorate patients’ prognosis when starting an EBP, such as the choice of an appropriate current dose (20-25 mL/kg/h as suggested by KDIGO guidelines) [2] and the nature of the membrane, with a particular attention on their unselective adsorptive capability for PAMPs and DAMPs. In this observational prospective study we investigated the association between organ dysfunction (as Sequential Organ Failure Assessment (SOFA) score) and several treatment settings in septic patients under continuous kidney replacement therapy (CKRT) with a modality of continuous veno-venous hemodiafiltration (CVVHDF).

METHODS: Forty-nine septic and septic shock patients treated in CKRT-CVVHDF with an acrylonitrile membrane (AN69ST or oXiris) and recorded on the ARRT registry (www.arrt.eu) were included for the analysis. We considered an observation time of 48 hours from the treatment initiation. Correlations between treatment settings at time 0 and trends of SOFA score (ΔSOFA) have been investigated with R software (version 4.0.3), using the Pearson correlation coefficient.

RESULTS: We found a statistically significant correlation between the filtration fraction (FF) and the ΔSOFA (0-48h) (Correlation coefficient -0.234; 95% CI -1;-0.00462 [p = 0.0468]). In our analysis, none of the other single parameters considered was associated in a statistically significant manner with the amelioration of the SOFA score.

CONCLUSIONS: The amelioration in terms of multi-organ failure in patients performing treatments with a higher filtration fraction may suggest the potential role of this latter in improving organ function in septic patients undergoing EBP. The results of our observation on 49 treatments in CKRT-CVVHDF performed with an acrylonitrile membrane may suggest a potential role of a higher filtration fraction to enhance adsorptive properties of the entire bulky of these hemodiafilters, ameliorating patients' prognosis. Larger, randomized, controlled studies are needed to confirm our observations.

BIBLIOGRAPHY

1. Vincent JL. New therapies in sepsis. Chest. 1997 Dec;112(6 Suppl):330S-338S. doi:10.1378/chest.112.6_supplement.330s. PMID: 9400898.

2. Khwaja A. KDIGO clinical practice guidelines for acute kidney injury. Nephron Clin Pract. 2012;120(4):c179-84. doi: 10.1159/000339789. Epub 2012 Aug 7. PMID: 22890468.

**Fig. 1 (abstract A179).**
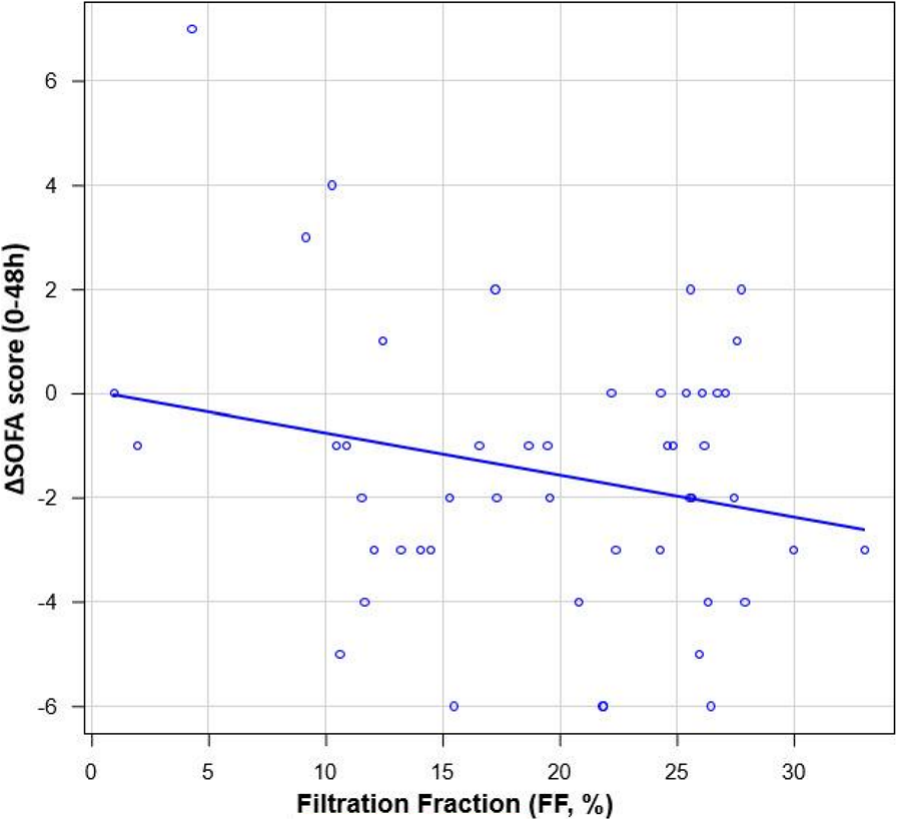
See text for description

### A180. Awake veno-venous extracorporeal membrane oxygenation in severe covid-19 acute respiratory distress syndrome: a single center experience

#### Nardelli P.^2^, Pieri M.^2^, Bonizzoni M.A.^2^, Delrio S.^1^, Maimeri N.^2^, Scandroglio A.M.^2^

##### ^1^Department of Anesthesia and Intensive Care, University of Udine ~ Udine ~ Italia, ^2^Department of Anesthesia and Intensive Care, IRCCS San Raffaele Scientific Institute ~ Milano ~ Italia

###### **Correspondence:** Scandroglio A.M.

Background

Veno-venous extracorporeal membrane oxygenation (VV ECMO) has been extensively used worldwide in COVID-19 when severe refractory respiratory failure threatened the survival of otherwise healthy subjects[1]. Managing patients undergoing V-V ECMO with an awake strategy – which implies early discontinuation of mechanical ventilation, mobilization and ambulation- is an attractive strategy to avoid deconditioning and may improve outcome[2,3]. The aim of this study was to describe experience and outcomes of awake VV ECMO due to COVID-19 at a national referral center.

Methods

We performed an observational study of all patients undergoing V-V ECMO for COVID-19 up to October 31st 2021 at a tertiary care center in Italy. VV ECMO institution was performed according to the Italian ECMOnet protocol[4] for refractory acute respiratory distress syndrome. All ECMO patients were evaluated for awake ECMO by the ECMO team. Once hemodynamic stability was achieved, sedation was lifted according to our awake ECMO strategy (figure 1) and active physical therapy was started. The patient was evaluated for extubation once fully awake and cooperative. A personalized daily rehabilitation program including early mobilization (chair and walking) was implemented with the assistance of physical therapists.

Results

Twenty-seven patients underwent VV ECMO treatment due to COVID-19. Awake ECMO was feasible in 15 patients (60%), 5 during the first wave (5/12, 42%), 10 during the second wave (10/13, 77%), following implementation of early referral pathways for patients. Mean age was 50+-15 years and 10/15 (67%) patients were male. Dual lumen cannulation was used in the majority of patients (20/25, 80%), most of which could fulfil the awake strategy (13/20, 65%). Weaning from ECMO was achieved in 13/15 patients (87%) managed with the awake strategy, while the other two were listed for lung transplantation due to the absence of lung function recovery. VV ECMO duration was 36+-17 days and 6/15 patients (40%) could walk during ECMO. Disability-free survival at 90-day follow-up was observed in 13/15 (87%) patients managed with the awake ECMO strategy: all these patients had full recovery of lung function. Intensive care unit stay was 54+-21 days. No complication related to mobilization during ECMO was recorded.

Conclusions

An awake approach was safe and feasible in patients with COVID-19 supported with V-V ECMO and associated with generally favorable outcomes in a high-volume, tertiary care center. All patients successfully weaned from VV ECMO had complete recovery of lung function.

References

1.Barbaro RP, MacLaren G, Boonstra PS, Combes A, Agerstrand C, Annich G,et al. Extracorporeal membrane oxygenation for COVID-19: evolving outcomes from the international Extracorporeal Life Support Organization Registry.Lancet 2021;398:1230–8

2.Langer T, Santini A, Bottino N, Crotti S, Batchinsky AI, Pesenti A,et al."Awake" extracorporeal membrane oxygenation (ECMO):pathophysiology, technical considerations, and clinical pioneering.Crit Care 2016;20:150

3.Crotti S, Bottino N, Ruggeri GM, Spinelli E, Tubiolo D, Lissoni A,et al.Spontaneous Breathing during Extracorporeal Membrane Oxygenation in Acute Respiratory Failure.Anesthesiology 2017;126:678-687

4.Patroniti N, Zangrillo A, Pappalardo F, Peris A, Cianchi G, Braschi A,et al. The Italian ECMO network experience during the 2009 influenza A(H1N1) pandemic:preparation for severe respiratory emergency outbreaks.Intensive Care Med 2011;37:1447-57

**Fig. 1 (abstract A180).**
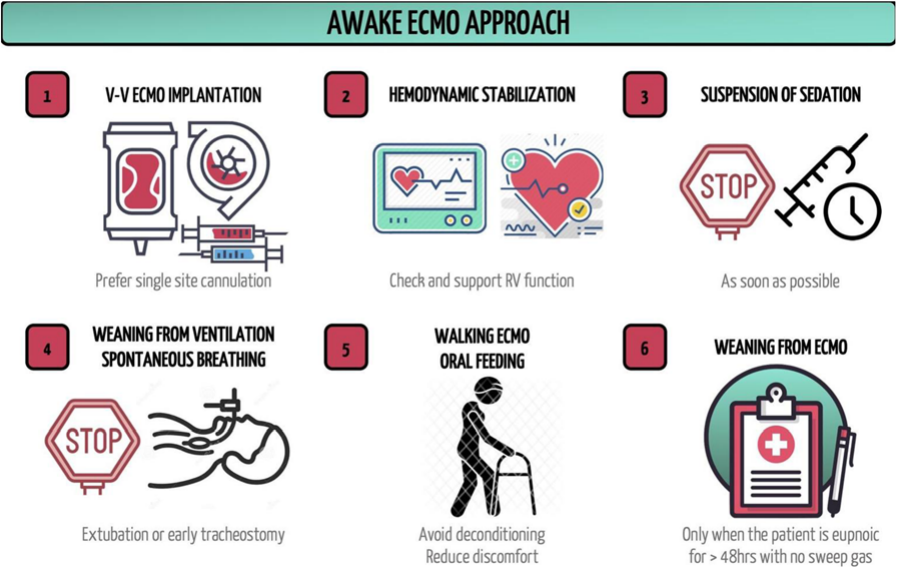
See text for description

## Trasfusione, emostasi e trombosi

### A181. Coagulopathies and trauma: abik’s case

#### Coppola P.^1^, Di Franco S.^1^, Alfieri S.^1^, Faraone E.^1^, Galluccio V.^1^, Ciampa A.^2^, Lanna A.^1^, Caruso A.^1^, Fiorillo A.^1^, Narni Mancinelli V.^1^, Lustrino M.^1^, Maione A.^1^, Vincenti R.^1^

##### ^1^UOC Anestesia e Rianimazione OO.RR. Area Nolana ASL Napoli 3 Sud ~ Nola ~ Italia, ^2^Azienda Ospedaliera di Rilievo Nazionale e di Alta Specialità San Giuseppe Moscati ~ Avellino ~ Italia

###### **Correspondence:** Coppola P.

Background

Major traumas are always a challenge for resuscitators^1^. In the complexity of this pathology, among the most fearful complications there are coagulopathies, due to “bad” therapeutic choices, to the trauma itself, or to pre-existing pathologies^2^. Despite the urgency, one should never forget to analyze the anamnesis^1^.

Case report

Male, 15 y.o., came for road trauma. Nothing relevant revealed in anamnesis, moderate obesity, language barrier (from Bangladesh, no cultural mediator).

Total body CT showed post-traumatic laceration of the left kidney, for which urgent and non-delayable surgery was indicated. Patient was subjected to left nephrectomia. Stable parameters during surgery, no need for transfusions.

On the first day the patient was extubated, but later in the afternoon, due to the presence of about 500 ml of blood in the surgical drain, a new CT scan was performed, and the patient was subjected to exploratory laparotomy with positioning of packing and Vac-Therapy. Blood and plasma were infused. Apparently, the cause of the bleeding did not appear to be of surgical origin. Removal of VAC-THERAPY and packing after 72 hours.

The first hypothesis for the bleeding was trauma coagulopathy. By going through the family anamnesis, patient’s brother was suffering from haemophilia type B, X-linked recessive coagulopathy, less common than haemophilia A, which leads to the deficit of the coagulation factor IX^3^. Up to now, patient had never presented symptoms, nor had he shown changes in the coagulogram, except for a slight lengthening of the APPT, already present at hospitalization. On our new measurement, on 2nd day, the factor IX appeared reduced (19% V.N. 60-130%), so a plasma and human coagulation factors infusion was performed. The haemocoagulative aspect seemed stable in the following days, with the APPT value always slightly above the normal value, and consolidation of a hematoma in the left renal area. Furthermore, a problem concerning the adjustment of enoxaparin dosage arose, necessary for prolonged bed rest. Therapeutic dosing resulted in new bleeding, so the drug had to be reduced to a minimal dose.

Due to difficult weaning, the patient was tracheotomized on the 17th day. Despite the recombinant factor IX therapy, the procedure led to blood dripping at the level of the breach, with a new need for blood and plasma. Reduction of bleeding in the 22nd day. The patient was decanulated on the 28th day. Next day, given the stability of all parameters, he was transferred to the surgical department. Informed consent to publish had been obtained.

Conclusion

Trauma is a pathology that leads to an alteration of human homeostasis, including coagulation, and it can reveal pre-existing pathologies. We must always keep in mind the importance of anamnesis, including family history, which can guide clinical choices even in emergency situations.

References

^1^Petrosoniak A, Hicks C. Resuscitation Resequenced: A Rational Approach to Patients with Trauma in Shock. Emerg Med Clin North Am. 2018 Feb;36(1):41-60

^2^Kornblith LZ, Moore HB, Cohen MJ. Trauma-induced coagulopathy: The past, present, and future. J Thromb Haemost. 2019 Jun;17(6):852-862

^3^Dolan G, Benson G, Duffy A: Where are we now and what does the future hold? Blood Rev. 2018 Jan;32(1):52-60

### A182. Covid-19 pneumonia: clinical, laboratory and instrumental Intensive Care Unit (ICU) features and long-term outcomes. the experience of an Italian II level centre

#### Pazzi M.^1^, Conti D.^1^, Pasquinelli F.^2^, Venni A.^1^, Zanfino L.^1^, Tofani L.^3^, Carpi R.^2^, Pavoni V.^1^

##### ^1^SOC Anestesia e Rianimazione Ospedale Santa Maria Annunziata, Azienda USL Toscana Centro ~ Firenze ~ Italia, ^2^Department of Radiology, Santa Maria Nuova Hospital, Azienda USL Toscana Centro ~ Firenze ~ Italia, ^3^Department of Statistics, Computer Science, Applications. University of Florence ~ Firenze ~ Italia

###### **Correspondence:** Pazzi M.

**Background**

COVID-19 spectrum disease can progress from asymptomatic infection to severe pneumonia and acute respiratory distress syndrome (ARDS) with high morbidity and mortality rates. Recent literature reports a high risk of Post-Intensive Care Syndrome (PICS) marked by physical, cognitive and mental impairments. Furthermore, a close correlation between prothrombotic laboratory alterations and COVID-19 severity has been suggested and increased D-Dimer (DD) levels have been reported as a negative prognostic factor.

Although Viscoelastic tests (VETs) have been used to detect hypercoagulability, their exact role in COVID-19 management as prediction tools for clinical deterioration and ICU admission has yet to be elucidated

The aim of the study is to evaluate short and long term (6 months) functional, respiratory and psychological outcomes in a ICU COVID-19 patients group.

Furthermore, any relationship between ROTEM profiles and ICU adverse outcomes has been investigated.

**Materials and Methods**

In this single-centre retrospective, observational study, 26 critically ill COVID 19 pneumonia patients admitted to ICU of Santa Maria Annunziata Hospital (Florence) between March 2020 and May 2021 were enrolled. Six-months after ICU discharge a multidisciplinary follow-up, including outpatient visits, ROTEM and blood gas analysis, respiratory function tests, pulmonary CT and psychological assessments was scheduled.

Results

The sample showed a high prevalence of male (n=21, 80%), obesity (n=13, 50%) and hypertension (n=13, 50%) and in 26.9% (n=7) of the patients group mechanical ventilation (MV) has been performed.

At ICU admission, a prothrombotic pattern, with high DD (3494±6829 ng/ml) and fibrinogen levels (678,3±52,6 mg/dl) was documented. A modest increase of MCF in EXTEM (72,4±4,7 mm) and FIBTEM (30±6,9 mm) was instead depicted. (**Table 1**)

No correlation has been highlighted between ROTEM prothrombotic alterations at ICU admission and adverse outcomes such MV, venous thromboembolism (VTE) and COVID-19 associated coagulopathy (CAC).

At 6-month follow-up, complete normalization of ROTEM profile emerged, except for a modest increase of CT INTEM **(Table 1**)

PICS was diagnosed in 20 patients (76.9%). However, no correlation between clinical, laboratory or instrumental ICU features and long-term physical or cognitive disabilities was detected.

As concerns follow-up respiratory evaluations, CT scans highlighted a significant increase in residual ventilated lung volume (RLV) (3.31 L vs 5.20 L, p <0.001) (**Figure 1**), and arterial oxygenation index improvement occurred (P/F 153±90 vs 416±53, p<0,001). Spirometric parameters, instead, resulted persistently altered in 12.5% of patients (moderate obstructive lung disease).

Conclusions

In the study group, ROTEM analysis at ICU admission highlighted a modest prothrombotic pattern with subsequent complete normalization at 6 months.

Hypercoagulability is a common ROTEM feature but it appears to increase neither VTE risk, intubation rate, or long term disability incidence.

Respiratory tests resulted normalized at follow-up visit, with significant improvement of both laboratory e instrumental data.

Similarly, ICU complications such as MV and VTE as well as CAC diagnosis do not appear to have affected functional and cognitive outcome at 6 months, when independently assessed.

Although more studies are needed to early identify PICS, the high prevalence (76,5%) of this syndrome suggest that follow-up program in all ICU COVID 19 patients should be scheduled.

**Fig. 1 (abstract A182).**
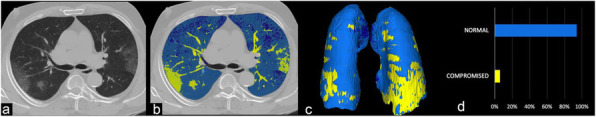
Quantitative lung CT analysis of an 81-year-old male patient affected by COVID-19. Example of Residual Ventilated Lung Volume

**Table 1 (abstract A182).** Variation in coagulation and respiratory parameters between ICU admission and 6 month follow up

	***References***	***ICU admission***	***6 month follow-up***	***P-value***
***ROTEM PARAMETHERS***				
***EXTEM***				
*CT (s)*	38–79	79,8±13,7	74,5±44,8	0,566
***CFT (s)***	**34–159**	**50,1±14,9**	**89,6±63,7**	<0,0001*
***A5 (mm)***	**34–55**	**56,7±7,4**	**45,6±9,3**	<0,0001*
***A10 (mm)***	**43–65**	**66,2±6,1**	**55,8±9,3**	<0,0001*
***MCF (mm)***	**50–72**	**72,4±4,7**	**64,1±8,3**	<0,0001*
***ML (% 60)***		**10,3±4,6**	**10±10,9**	0,0164*
**INTEM**				
*CT (s)*	100–240	183,3±28,5	247,2±294,9	0,6475
***CFT (s)***	**30–110**	**50,9±14,9**	**91,2±90,22**	<0,0001*
***A5 (mm)***	**38–57**	**54,5±7,5**	**43,8±12,2**	<0,0001*
***A10 (mm)***	**44–66**	**64±6,4**	**53,5±12,8**	<0,0001*
***MCF (mm)***	**50–72**	**70±5,2**	**60,3±12**	<0,0001*
**FIBTEM**				
***MCF (mm)***	**9–25**	**30±6,9**	**14,8±4,7**	<0,0001*
**RESPIRATORY FUNCTION**				
***Residual Lung Volume (L)***	**≥5 l**	**3,3±1,5**	**5,2±0,9**	0,0009*
***Pa02/FiO2***	**≥300**	**153±90**	**416±53**	**0,0001**

### A183. Changes over time of thromboelastometry findings in elderly patients with proximal femoral fracture complicated by venous thromboembolism

#### Gianesello L., Rossi V., Guerri I., Travagli E., Catinelli S., Boccaccini A.

##### Department of Anesthesia and Intensive Care, Orthopedic Anesthesia, University-Hospital Careggi ~ Firenze ~ Italia

###### **Correspondence:** Rossi V.

**Background.** Venous thromboembolism (VTE) [i.e., deep vein thrombosis (DVT) and pulmonary embolism (PE)] is a common complication following femoral fractures in elderly patients with morbid and potentially fatal consequences [1-2]. The pharmacological prophylaxis of VTE is based on the use of anticoagulants which often find limitations due to the increased risk of bleeding in the fracture site. Standard coagulation tests do not predict VTE. Rotational thromboelastometry (ROTEM) evaluates specific aspects of the hemostatic mechanisms through a dynamic analysis; it has been indicated to detect hypercoagulable and hypofibrinolytic states associated to symptomatic VTE [3]. At present, to our knowledge there are no studies defining the change over time of prothrombotic state in elderly population after femoral fractures. Therefore, we conducted a prospective study aim to evaluate the evolution of the hemostatic balance analyzing ROTEM parameters in patients with proximal femoral fracture complicated by VTE compared to those without.

**Materials and methods.** This single center study included 40 elderly patients with proximal femoral fracture. Bilateral Doppler ultrasonography was performed in each of the included patient at the hospital admission (T0) and 5 days after (T5). Standard laboratory assays and ROTEM parameters were recorded and compared between patients with DVT (DVT group) and patients without DVT (no DVT group) at T0, 3 (T3) and 7 (T7) days later. A p value <0.05 was considered statistically significant.

**Results.** No patient sustained proximal or distal DVT at T0. At T5, the incidence of distal DVT was 17.5%, consensual on the fractured side in 6 patients, while one patient presented bilateral DVT. There was no evidence of proximal DVT. No patient experienced episodes of PE. Among standard coagulation parameters, only platelets count was significantly increased in Group DVT compared to no DVT group, in all evaluation times. At T0, ROTEM analysis showed clot formation time (CFT)-EXTEM significantly reduced in patients who developed DVT compared to those who did not, while parameters A5, A10 and maximum clotting firmness (MCF) were found to be significantly higher in patients with DVT than in no DVT. Moreover, DVT group demonstrated a hypofibrinolytic state (ML 60) higher than no DVT group at T0 (p=0.001) and T3 (p=0.0001). No differences were found in ROTEM parameters at T7 between two groups, except for an increase of clotting time (CT)-INTEM in DVT patients (p=0.003) (Table 1).

**Conclusions.** ROTEM analysis reflects an early hypercoagulative and low fibrinolytic states in elderly patients with femoral fracture complicated by asymptomatic DVT. This procoagulant condition seems to return comparable to patients without DVT after a week.

**References**

1. McNamara I, Sharma A, Prevost T, et al. Symptomatic venous thromboembolism following a hip fracture. Acta Orthop 2009; 80 (6):687-92.

2. Anderson FA Jr, Spencer FA. Risk factors for venous thromboembolism. Circulation. 2003;107 (23 Suppl 1):I9–16.

3. Holcomb JB, Minei KM, Scerbo ML, et al. Admission rapid thromboelastography can replace conventional coagulation tests in the emergency department: experience with 1974 consecutive trauma patients. Annals of surgery 2012;256:476-486.

4. McNamara I, Sharma A, Prevost T, et al. Symptomatic venous thromboembolism following a hip fracture. Acta Orthop 2009; 80 (6):687-92.

5. Anderson FA Jr, Spencer FA. Risk factors for venous thromboembolism. Circulation. 2003;107 (23 Suppl 1):I9–16.

6. Holcomb JB, Minei KM, Scerbo ML, et al. Admission rapid thromboelastography can replace conventional coagulation tests in the emergency department: experience with 1974 consecutive trauma patients. Annals of surgery 2012;256:476-486.

**Table 1 (abstract A183).** ROTEM parameters at T0, T3 and T7 in patients with DVT and those without

	Reference	T0 no DVT	T0 DVT	p-value	T3 no DVT	T3 DVT	p-value	T7 no DVT	T7 DVT	p-value
**INTEM**										
**CT, s**	*100-240*	159.7±13.4	163.7±17.2	0.682	168.8±19.5	161.7±9.7	0.484	166.8±25.5	183.8±11.8	0.003*
**CFT, s**	*30-110*	69.7±17.9	60.8±24.2	0.101	59.1±16.6	45.4±6.0	0.023*	52.1±19.1	47.1±15.6	0.312
**A5, mm**	*38-57*	49.5±6.3	54.7±7.2	0.064	54±7.6	61.1±3.0	0.029*	60.7±6.6	56.2±17.7	0.934
**A10, mm**	*44-66*	59.4±5.9	63±4.5	0.108	63.6±7.0	70.0±2.2	0.035*	69.5±5.3	6.8±19.6	0.819
**MCF, mm**	*50-72*	65.1±4.9	69.7±4.4	0.069	69.1±6.6	74.2±1.9	0.057	74.2±4.3	68.5±15.4	0.819
**ML 60, %**	*3-15*	7.4±1.7	5.8±1.6	0.03*	7.9±1.5	6.4±1.1	0.02*	7.3±1.9	7.5±1	0.837
**EXTEM**										
**CT, s**	*38-79*	58.4±6.4	54.2±4.7	0.10	53.8±5.9	58.1±5.8	0.137	56.5±8.1	59.0±9.8	0.522
**CFT, s**	*34-159*	116.1±33.0	92±9.3	0.01*	87.1±28.6	71.7±21.8	0.129	72±18.1	65.1±10.4	0.421
**A5, mm**	*34-55*	42.3±7.2	49.7±1.9	0.001*	49.1±7.8	54.4±7.1	0.078	54.9±5.8	58±4.8	0.172
**A10, mm**	*43-65*	54.1±6.9	60.8±1.5	0.001*	60.7±7.2	65.2±5.8	0.092	65.6±5.4	68±4.6	0.273
**MCF, mm**	*50-72*	62.2±5.6	67.5±1.7	0.03*	66.1±6.7	70.2±5.0	0.114	70.7±4.7	72.5±3.9	0.494
**ML 60, %**	*3-15*	8.6±1.7	6.1±1.4	0.001*	7.4±1.5	4±1.9	0.0001*	8.2±1.2	7.5±0.9	0.460
**FIBTEM**										
**MCF, mm**	*9-25*	17.4±5.6	18.2±3.6	0.381	26.6±6.5	27.8±3.1	0.366	25.8±6.7	25.2±2.0	0.836

*CT* clotting time, *CFT* clot formation time, *A5* amplitude of clot firmness measured in millimeters at 5 minutes after start of clot formation, *A10* amplitude of clot firmness measured in millimeters at 10 minutes after start of clot formation, *MCF* maximum clot formation, *ML* maximum lysis, *DVT* deep vein thrombosis

Data are expressed by mean ± SD, *p value<0.05

### A184. Ten year experience with a local protocol for the management of massive hemorrhage in trauma patients

#### Frattari A.^1^, Battilana M.^1^, Ciulli R.^1^, Quaglietta A.^2^, Accorsi P.^2^, Zocaro R.^1^

##### ^1^Unit of Intensive care, Spirito Santo Hospital, ~ Pescara ~ Italia, ^2^Unit of Transfusion medicine ~ Pescara ~ Italia

###### **Correspondence:** Battilana M.

Hemorrhagic shock consistently represents the second-leading cause of early deaths among the heavily injured trauma patients, and a cause of potentially preventable death. Indeed, shock and coagulopathy upon arrival in the emergency room are independently associated with massive transfusion and increases mortality. Management of trauma-induced coagulopathy is based mainly on early support of coagulation with tranexamic acid, fibrinogen substitution and blood transfusion. To evaluate the efficacy of the protocol on mortality and blood red cell and plasma consumption, we conducted a retrospective analysis of intensive care consumptions in 2012, before protocol adoption, and from 2014 through 2021. Here we report on our 10-year experience with the implementation of our local protocol for early management of massive haemorrhage, to reduce both rates of blood and plasma transfusion and early mortality.

Methods

Based on the Italian Early Coagulation Support Protocol, a multidisciplinary group of local physicians promoted a treatment protocol early in 2013 for massive haemorrhage in trauma. The aim was to implement the timely use the available technical resources to promote the early supplementation of fibrinogen, to reduce both the time interval between trauma and coagulation support and the amount of plasma and blood units needed. Briefly, the protocol facilitates early ER notification of major trauma in advance of transportation; assessment of shock index upon arrival, rapid laboratory assessment of lactates and base deficit, haemoglobin and coagulation with fibrinogenemia. Low volume resuscitation was used to guarantee tissue oxygenation. Upon detection of one or more of: Systolic Blood Pressure <100 mmHg, lactates >5.5 mmol/L, base deficit <-6mmol/L, Haemoglobin <9gr/dL or bleeding at early FAST, the massive transfusion protocol was activated: 1g of tranexanic acid in 10min, followed by 1g over 8h; 2 units of 0-RBC and 2g of fibrinogen were initially transfused; then 4 additional units (1200cc) of RBC and 1 unit of plasma (700cc) were infused until availability of laboratory measurements. Additional fibrinogen (2g) was infused whenever fibrinogen levels were <1.5g/L. Meanwhile, surgical attention of the bleeding source was pursued. RBC/plasma in the same ratio, platelets (for counts <100,000/mmc) and fibrinogen were further infused until bleeding control and satisfactory (>1.5g/L) fibrinogen levels. Finally, calcium levels were corrected throughout the procedure, together with acidosis and hypothermia. Coagulation checks were repeated every 90m.

Results

Since 01/01/2014, to 31/12/2021, among 499 patients hospitalized due to trauma, we retrospectively identified those with an ISS > 15 and red blood cell transfusion > 3U. Massive haemorrhage was present in 126 patients. Consumption of blood components was significantly reduced across the years as described in figure 1. Death within first 24 hours dropped from 4/10 (2012 , 40% mortality) to 1 /11 (2021, 9.1%).

Conclusions

Implementing a local protocol for the management of massive haemorrhage contributed to significantly reduce early mortality due to coagulopathy, due to homogenizing the global approach to these patients. Consumption of blood components was significantly reduced in parallel.

**Fig. 1 (abstract A184).**
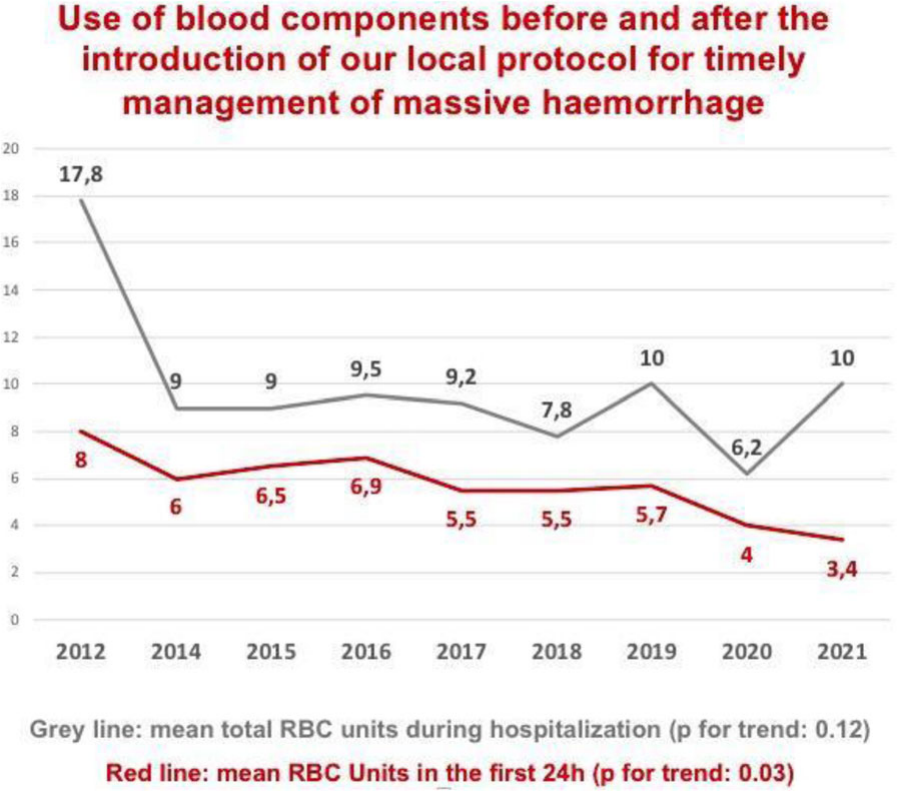
See text for description

### A185. Impact of rotational thromboelastometry (rotem) on clinical course and outcomes of covid -19 critically Ill patients: a retrospective single centre observational study

#### Pazzi M.^1^, Gemmi E.^1^, Conti D.^1^, Zanfino L.^1^, Tofani L.^2^, Pavoni V.^1^

##### ^1^SOC Anestesia e Rianimazione Ospedale Santa Maria Annunziata, Azienda USL Toscana Centro ~ Firenze ~ Italia, ^2^Department of Statistics, Computer Science, Applications. University of Florence ~ Firenze ~ Italia

###### **Correspondence:** Pazzi M.

**Background and Aims**

Recently a close correlation between prothrombotic laboratory alterations and COVID-19 severity has been suggested and increased D-Dimer (DD) levels have been reported as a negative prognostic factor. Although Viscoelastic tests (VETs) have been used to detect hypercoagulability (characterized by acceleration in clot formation time (CFT) and increased maximum clot firmness (MCF), their usefulness in clinical management of critically ill COVID-19 patients has yet to be elucidated. Our study aimed to identify the relationship between ROTEM profiles and adverse outcomes (mortality, needing for Mechanical Ventilation (MV), Venous Thromboembolism (VTE), COVID-19 Associated Coagulopathy (CAC)) in critically ill COVID-19 patients.

**Materials and Methods**

100 consecutive patients admitted to the intensive care unit (ICU) with severe COVID-19 pneumonia, between October 1, 2020 May 31, 2021 were enrolled in Florence, Santa Maria Annunziata Hospital. (Table 1).

**Results**

We enrolled a population with a significantly higher prevalence of male (80%), hypertension and diabetes. As concerns standard coagulation tests and ROTEM profile, a prothrombotic pattern was documented, with high DD (> 6 VN) and fibrinogen levels. A modest increase of MCF in EXTEM and FIBTEM was instead depicted probably due to early administration of "intermediate" LMWH dosage before ICU admission in moderate respiratory failure patients. Regarding ICU mortality, statistically significant differences were found between DD values and platelet counts. ROTEM analysis highlighted any hypercoagulability difference but Maximum Lysis (ML) values resulted statistically different in EXTEM and INTEM. (Table 1) A sub-group analysis demonstrated hypofibrinolysis in MV patients too. However, as concerns MV rate, the univariate analysis showed a possible association with hypercapnia (OR 1.11, 1.03-1.2, p = 0.0053) and leukocytosis (OR 1.12, 1.02-1.23, p = 0.0181).

Patients with VTEs (n=20) showed higher DD values (10732 ± 1703 ng/ml vs. 3773 ± 747 ng/ml, p=0,010319); conversely no correlation was highlighted in terms of ROTEM hypercoagulability or hypofybrinolisis. No statistical difference was found between CAC (n=29) vs no CAC (n=71) patients. Lastly, survival analysis allowed us to identify hypofibrinolysis (ML EXTEM; HR 0,87, 0,81-0,95, p=0,0011and INTEM ; HR 0,9, 0,84-0,97, p=0,0083)as a possible risk factor.

**Conclusions**

In our COVID-19 population, NOT-survivors had higher DD values, lower platelet counts and a more pronounced hypofibrinolytic ROTEM pattern than survivors. Hypofibrinolysis occurs more frequently in patients requiring MV, although it does not result as a possible risk factor for intubation. Similarly, hypercoagulability is a common ROTEM feature but it appears to increase neither VTE risk, intubation rate, or mortality probably due to early administration of intermediate-dose LMWH. Therefore, we could hypostasize that hypofibrinolysis, but not hypercoagulability, resulted in worse outcomes in terms of mortality risk, MV and VTE.

29% of patients met CAC criteria but no significant difference in mortality, MV rate, VTE and ROTEM parameters was revealed. Further studies are highly recommended in order to define the usefulness of VETs as a tool for multiparametric prognostic assessment. However, based on current data, ROTEM analysis could represent a helpful tool for predicting ICU admission risk and worst outcome as well as bleeding risk in critically ill patients.

Bibliography

1. G Lippi EF. D-dimer is associated with severity of coronavirus disease 2019: a pooled analysis. Thromb Haemost. 2020 May 1;120(5):876–8.

2. Lorini FL, Matteo M Di, Gritti P, Grazioli L, Benigni A, Zacchetti L, et al. Coagulopathy and COVID-19. Eur Heart J Suppl [Internet]. 2021 Oct 9 [cited 2021 Oct 21];23(Suppl E):E95.

3. MT Ganter CH. Coagulation monitoring: current techniques and clinical use of viscoelastic point-of-care coagulation devices. Anesth Analg. 2008;106(5):1366–75.

4. D Whiting JD. TEG and ROTEM: technology and clinical applications. Am J Hematol. 2014 Feb 1;89(2):228–32.

5. V Pavoni LGMPAHLS. Derangement of the coagulation process using subclinical markers and viscoelastic measurements in critically ill patients with coronavirus disease 2019 pneumonia and non-coronavirus disease 2019 pneumonia. Blood Coagul Fibrinolysis. 2021;32(2):80

**Table 1 (abstract A185).** See text for description

TOTAL POPULATION (N=100)		SURVIVORS (N=50)	NOT-SURVIVORS (N=50)	P
DEMOGRAPHIC FEATURES				
AGE (YEARS)	**66 ± 17**	**68,3**	**63,8**	**0,00241**
MALES (N; %)	**80; 80%**	**36**	**44**	**n.s.**
BMI (KG/M^2)	**28,5 ± 4,6**	**27,7**	**29,1**	**n.s.**
COMORBIDITIES (N, %)				
OBESITY KG/M^2	**38, 38%**	**22**	**16**	**n.s.**
HYPERTENSION (N, %)	**56, 56%**	**26**	**30**	**n.s.**
DIABETES (N, %)	**20, 20%**	**8**	**12**	**n.s.**
COPD (N, %)	**11, 11%**	**4**	**7**	**n.s.**
PREVIOUS AMI(N, %)(N, %)	**11, 11%**	**2**	**9**	**n.s**
ICU ADMISSION				
SOFA SCORE	**5,9 ± 2,5**	**5,9**	**3,6**	**0,00012**
SAPSII SCORE	**36,4 ± 9,5**	**37,2**	**27,7**	**0,00011**
SYMPTOMS’ ONSET TO ICU ADMISSION DELAY (DAYS)	**10 ± 6,7**	10,2	10	n.s.
PAO2/FIO2	**135 ± 75**	**148**	**131**	**n.s.**
ICU STAY				
ICU STAY (DAYS)	**13 ± 11**	**7**	**19**	**n.s.**
MECHANICAL VENTILATION (N, %)	**64**	**19**	**45**	**0,0013**
THROMBOEMBOLIC COMPLICATIONS (N, %)	**20, 20%**	**11**	**9**	**n.s.**
LABORATORY DATA AT ICU ADMISSION				
WBC (10^9/L)	**10.2 ± 5.2**	**8,96**	**12,52**	**n.s.**
PTL (10^9/L)	**246 ± 107**	**225,7**	**267,1**	**0,01443**
DD NG/ML	**5089 ± 1035**	**6192**	**3985**	**0,00554**
PT%	**70 ± 15,2**	68,8	71,5	**n.s.**
APTT SEC	**30.3 ± 8.5**	30,1	29,8	**n.s.**
FIBRINOGEN MG/DL	**640 ± 112**	616,6	664,3	**n.s.**
ROTEM PARAMETERS AT ICU ADMISSION				
CT INTEM SEC	**177.4 ± 28**	176,9	177,8	**n.s.**
CFT INTEM SEC	**53.1 ± 22.8**	56,1	50,2	**n.s.**
MCF INTEM MM	**69.8 ± 6**	69,5	70	**n.s.**
ML INTEM %	**8.9 ± 4.2**	**7,9**	**10**	**0,01467**
CT EXTEM SEC	**81.9 ± 29**	85,8	77,8	**n.s.**
CFT EXTEM SEC	**54.2 ± 21.6**	58,9	49,6	**n.s.**
MCF EXTEM MM	**72 ± 5.8**	71,6	72,4	**n.s.**
ML EXTEM %	**9.3 ± 4**	**8,2**	**10,5**	**0,00384**
MCF FIBTEM MM	**29.8 ± 8.5**	29,7	29,9	**n.s.**

### A186. Effects of red blood cell transfusions on renal blood flow and renal function in critically ill patients with moderate anemia

#### Chiavieri V.^1^, Fogagnolo A.^2^, Mari M.^1^, Benetto G.^1^, Volta C.A.^1^, Spadaro S.^1^

##### ^1^Department of Translational medicine for Romagna, University of Ferrara, Ferrara, Italy ~ Ferrara ~ Italia, ^2^Department of Anesthesia and Intensive Care Medicine, Azienda Ospedaliera Universitaria di Ferrara, Ferrara, Italy ~ Ferrara ~ Italia

###### **Correspondence:** Chiavieri V.

Introduction

The effect of red blood cells (RBC) transfusion on renal circulation on humans is not elucidated yet. Zafrani et al. demonstrated that RBC transfusion had a beneficial effect on kidney microvascular oxygenation in rat model [1]. Renal resistive index (RRI) was reported to independently predict Acute Kidney Injury in patients with septic shock and to be a dynamic marker of renal vascular properties. The aim of this study is to show the effects of RBC transfusion on renal blood flow, as detected by changes in RRI, in critically ill patients with moderate anemia. Furthermore, we hypothesized that the increase in creatinine values during ICU stay could be lower in transfused patients.

Methods

Critically ill patients who experienced Hemoglobin levels between 7 and 9 g/dL were included in the study. RBC transfusion was decided by physician. Posterolateral approach was used for kidney ultrasound. The peak systolic velocity (Vmax) and the minimal diastolic velocity (Vmin) were determined from an interlobar or arcuate artery by pulse wave Doppler, and the RRI was calculated as (Vmax - Vmin)/Vmax. RRI was calculated from both kidney and the worst values were used for analysis. RRI and RVSI were calculated before intervention (T1) and 2 hours after intervention (T2). The increase in creatinine (∆Creatinine) was calculated as the maximum creatinine value during the first 72 hours of ICU stay minus the value at ICU admission.

Results

Twenty-three patients were enrolled in the study. Of those, 12 were transfused whereas 11 were not. The study groups did non differ for baseline demographic and clinical variables, including age, SOFA score and hemoglobin. Median creatinine values at study inclusion was 1.0 [0.7 – 1.7] mg/dL in patients who received RBCT and 1 [0.8 – 1.2] mg/dL in patients who did not (p=0.88). Median RRI was 0.69 [0.65 – 0.74] at T1 and 0.68 [0.66 – 0.70] at T2 (p=0.23). After study intervention, only patients transfused lowered their RRI, from 0.71 [0.66 – 0.76] to 0.68 [0.66 – 0.70], p=0.038; accordingly, in the mixed model analysis RBC transfusion was significantly associated with RRI reduction (p=0.03). Transfused patients had lower increase in creatinine values (mean increase 0.3±0.08 vs 0.9±0.05, p=0.03)

Conclusion

In critically ill patients with moderate anemia, RBC transfusion is associated with increased renal blood flow, as detected by RRI. Moreover, transfused patients had a lower increase in creatinine values during the first 72 hours of ICU stay.

References

1. Zafrani L, Ergin B, Kapucu A, Ince C. Blood transfusion improves renal oxygenation and renal function in sepsis-induced acute kidney injury in rats. Crit Care. 2016 Dec 20;20(1):406.

**Fig. 1 (abstract A186).**
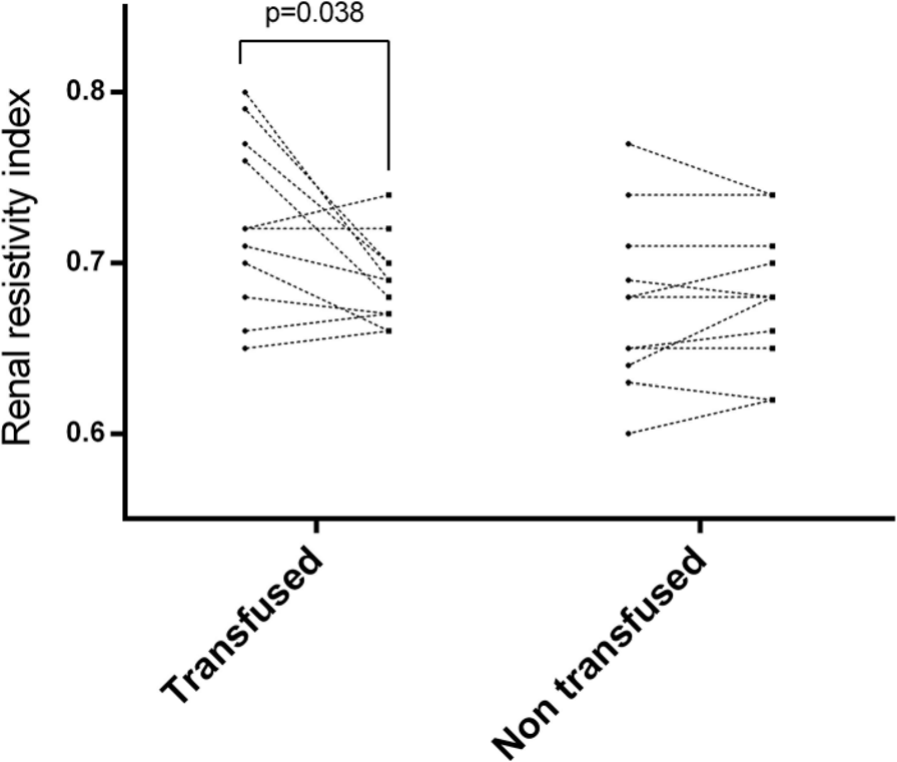
See text for description

## Ventilazione invasiva e non invasiva

### A187. A comparison of aerosol delivery performance using two nebuliser types during adult simulated mechanical ventilation

#### Fernandez Fernandez E.^1^, O”Sullivan O.^1^, Raftery S.^2^, Mac Giolla Eain M.^2^, Joyce M.^2^, Macloughlin R.^2^

##### ^[1]^Medical Affairs ~ Aerogen Ltd ~ Ireland, ^[2]^R&D, Science & Emerging Technologies ~ Galway ~ Ireland

###### Correspondence: Fernandez Fernandez E.

Background

Nebulisers are used to aerosolise therapeutics for patients with pulmonary illnesses. The amount of drug delivered to the lung can vary depending on nebuliser type used. This study compares the aerosol performance of a vibrating mesh nebuliser (VMN) and jet nebuliser (JN) during simulated adult mechanical ventilation, in terms of tracheal dose, nebulisation time, and residual drug volume and concentration.

Material & Methods

3mL of 2mg/mL salbutamol (GSK, IRE) was aerosolised using both a VMN (Aerogen Solo, Aerogen, IRE) and JN (Cirrus2, Intersurgical, UK). The VMN was positioned on the dry side of the humidifier (MR850, F&P, NZ) and the JN on the inspiratory limb within a dual limb circuit (RT111, F&P, NZ) during simulated mechanical ventilation (Servo-I, Maquet, SWE) (V_T_: 500mL, 15 BPM, I:E: 1:1). The JN was operated at a gas flow rate of 8LPM and run until sputter + 1-minute. A filter (303EU, Vyaire Medical Inc., USA) was placed between the endotracheal tube (8.0mm ID, Flexicare Medical, UK) and the test lung (Reservoir bag, Intersurgical, UK). Drug dose and residual concentration were determined by quantifying the drug mass captured on the filter and remaining in the medication cup post nebulisation using UV-spectrophotometry at 276nm. Residual volume was determined gravimetrically. Testing was performed at N=5. Tracheal dose is expressed as a percentage of the nominal dose placed in the nebuliser medication cup. Paired T-tests were used to determine significance (p ≤ 0.05).

Results

Conclusions

Results show that the use of a VMN over a JN results in a statistically significantly greater quantity of aerosol delivered to the simulated adult patient (p = 0.000) in a shorter period (p = 0.001). A statistically significant quantity of drug remained in the JN medication cup post nebulisation (p = 0.000) and was more concentrated, an increase of 0.51mg/mL, compared to the original drug concentration.

**Table 1 (abstract A187).** Mean ± SD Nebuliser Performance

	VMN	JN	P-Value
**Tracheal Dose (%)**	21.10 ± 2.20	4.23 ± 0.40	0
**Dose Time (min:sec)**	05:41 ± 0:35	07:23 ± 0:14	0.001
**Residual Volume (%)**	1.23 ± 0.34	68.30 ± 2.90	0
**Residual Concentration (mg/mL)**	N/A	2.51 ± 0.06	N/A

### A188. Safe higher tidal volumes and lower peep levels in ards patients managed with transpulmonary pressure guided mechanical ventilation

#### Grillandini C.^2^, Calamai I.^1^, Valente S.^2^, Landi A.^3^, Giuntini R.^1^, Romagnoli S.^2^, Spina R.^1^

##### ^1^Ospedale San Giuseppe, Dipartimento di Anestesia e Rianimazione ~ Empoli ~ Italia, ^2^Università degli Studi di Firenze ~ Firenze ~ Italia, ^[3]^Ospedale Bolognini, Dipartimento di Emergenza ~ Bergamo ~ Italia

###### **Correspondence:** Grillandini C.

BACKGROUND: Acute respiratory distress syndrome (ARDS) is a frequent reason for admission in Intensive Care Units (ICUs), with high mortality rate and, despite progressions in knowledge and pathophysiology, complex management. Actual recommendations of mechanical ventilation in ARDS include low tidal volumes (6-8mL/kg PBW) and a plateau pressure below 30cmH2O. A difficult task is the selection of the best individualized positive end expiratory pressure (PEEP) in order to prevent atelectasis avoiding alveolar overstretching. Transpulmonary pressure has been proposed as a marker of ventilator induced lung injury and can be used to set individualized ventilation parameters.

The aim of the present study is to investigate if a ventilation strategy based on the analysis of transpulmonary pressures can lead to better outcome than the use of ARDSNet PEEP/FiO2 tables.

MATHERIALS AND METHODS: Retrospective before-after study which compares ventilation parameters in patients managed according to transpulmonary pressure measurements versus the ARDSNet PEEP/FiO2 table.

All non-surgical patients with moderate to severe ARDS admitted to the ICU of the San Giuseppe Hospital in Empoli (Firenze) between 2014 and 2018 and invasively ventilated for at least 4 days were included in the study. Clinical features and ventilation parameters were recorded and, for statistical analysis, the enrolled patients were divided into two groups: the first group of patient ventilated according to the ARDSNet PEEP/FiO2 tables (No-PTP) and the second group of patients ventilated following the measured transpulmonary pressures (PTP). The collected data were processed through the statistical program Quick Calc GraphPAD calculator.

Primary endpoint was to understand if transpulmonary pressure guided ventilation would lead to a reduction in the ICU mortality rate.

RESULTS AND DISCUSSION: Transpulmonary guided ventilation was used in 54/285 (18.9%) of the analysed cases. A statistically significant difference between the two groups was found in the severity of respiratory failure at ICU admission: 44.4% of the PTP group patients presented severe ARDS (PaO2/FiO2 ratio lower than 100) versus 15.6% of the No-PTP group patients. In the PTP group, tidal volumes (Vt) were significantly higher and PEEP were significantly lower than expected. No significant statistically differences were found in the mortality rate.

The present study shows that in daily clinical practice measurement of PTP can be useful, especially in the more severe cases, in which the compliance with the theoretical limits imposed by traditional ventilation strategies does not allow to solve conditions of hypoxemia and/or respiratory acidosis.

CONCLUSION: A transpulmonary pressure guided ventilation can be used to overcome the limits imposed by the "baby-lung" concept, approaching a personalized ventilation.

Limitations: retrospective, observational study, which compares two not randomized groups of patients, different in numerosity and clinical characteristics at admission in ICU. Further prospective studies are needed to confirm our observations.

**Fig. 1 (abstract A188).**
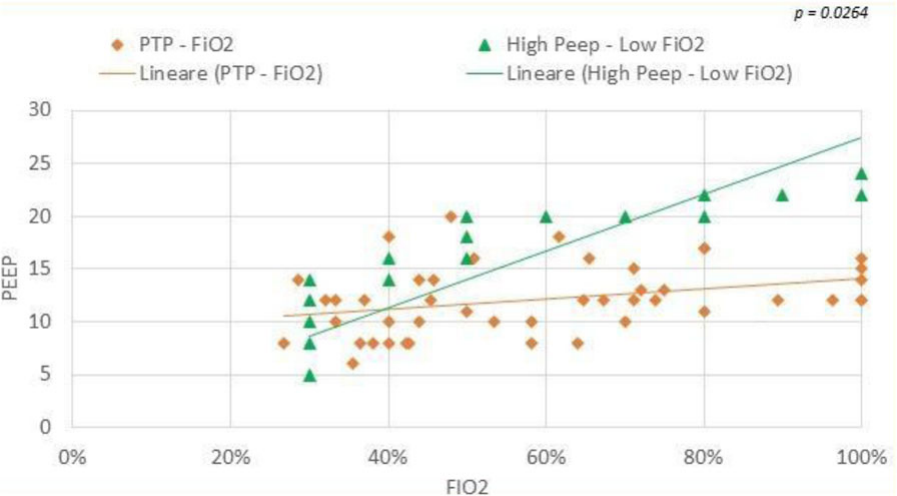
See text for description

### A189. Ultrasound diaphragmatic effects of high flow nasal cannula compared with venturi mask and helmet cpap in acute hypoxic respiratory failure

#### Pozzi T.^1^, Formenti P.^2^, Coppola S.^2^, Chiodaroli E.^2^, Chiumello D.^2^

##### ^1^Dipartimento di fisiopatologia Medico-Chirurgica e dei Trapianti, Università degli Studi di Milano ~ Milano ~ Italia, ^2^SC Anestesia e Rianimazione, Ospedale San Paolo – Polo Universitario, ASST Santi Paolo e Carlo, Milan, Italy ~ Milano ~ Italia

###### **Correspondence:** Pozzi T.

Background

In recent years, new devices that deliver totally conditioned gas through a nasal cannula at high flow have emerged as a safe and useful supportive therapy in many clinical situations. Few studies investigated the effects of HFNC on ultrasound diaphragmatic function. The aim of this study is to evaluate the diaphragmatic effects of high velocity nasal insufflation compared to venturi mask and helmet continuous positive airway pressure (CPAP) in patients with AHRF.

Material and methods

Single-center cross-over clinical study. 20 patients with ARF admitted to the ICU in spontaneous breathing assisted with standard oxygen were enrolled. Each patient was entered in the three study phases with the same set FIO2 and flow (40 L/min) for 60: venturi oxygen facial mask, HFNC, helmet CPAP. At the end of each phase, we collected arterial blood gas analysis data, respiratory rates, hemodynamics, and diaphragm ultrasound. we measured the esophageal pressure swings during inspiration (DPes) and the driving transpulmonary pressure (DPL).

Results

Diaphragmatic excursion did not significantly differ between the different devices (1.4 [0.9 – 2.1] 1.8 [1.1 – 2.2] 1.6 [1.1 – 1.8] cm, p= 0.02) even if a positive trend was detected by using HFNC and Helmut CPAP as compared to venturi mask. The thickening ratio was significantly lower during venturi oxygen mask support, and it improved both during HFNC and helmet CPAP (26 [18 – 34] 35 [30 – 42]* 37 [33 – 43]* %, p< 0.001). During HFNC, DPes were significantly lower than with the venturi oxygen facial mask (7.8 [6.4 – 9.2] 5.9 [4.6 – 7.4]* 5.0 [3.9 – 6.1]*, p <0.001).

Conclusions

HFNC influence diaphragmatic function both in terms of motion and contraction as compared to venturi mask. The study confirms some previous observation regarding their physiologic effects in patients with AHRF including improved gas exchange, dynamic compliance, transpulmonary pressures, and lowered respiratory effort.

**Fig. 1 (abstract A189).**
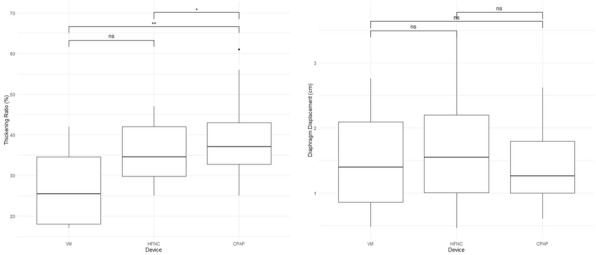
See text for description

### A190. Indipendent lung ventilation with a double-lumen endotracheal tube and extracorporeal CO2 removal in a severe unilateral lung desease: a case report

#### D’Andrea G., Ballanti C., Maldarelli F., Romano H., Alessandri F., Pugliese F.

##### Department of Anesthesiology, Critical Care and Pain Medicine, “Sapienza” University of Rome, Policlinico Umberto I, Rome, Italy ~ Roma ~ Italia

###### **Correspondence:** D’Andrea G.

Background

In the acute respiratory distress syndrome (ARDS) mechanical ventilation is often unable to provide adequate oxygenation. In some cases the use of independent lung ventilation (ILV) associated with extracorporeal CO2 removal (ECCO2 R) becomes a good tool to restore gas exchanges. [1, 2]

Case report

A 49-year-old woman was admitted to the emergency room with a case of bowel obstruction complicated by acute respiratory failure probably caused by aspiration pneumonia. It was initially treated by non-invasive ventilation until admission to intensive care unit, however, her oxygenation and hemodynamic got worse. Despite conventional protective mechanical ventilation the acute respiratory failure a right pneumothorax occurred and it was aggravated by a broncho-pleural fistula. Progressive atelectasis of the damaged lung resulted in a severe hypoxemia and hypercapnia refractory to conventional mechanical ventilation, therefore independent lung ventilation (ILV) was performed using a double-lumen endotracheal tube (DLT). This unconventional mode of mechanical ventilation allowed to differently ventilate the affected lung with minimal tidal volume (Vt) and low inspiratory plateau pressure and to prevent hyperinflation of the non-affected lung improving lung perfusion. In order to avoid respiratory acidosis and the increase of PaCO2 due to the reduction of Vt, ECCO2R (blood flow raised up to 350 ml/h) has been started. After 5 days there was the recruitment of the atelectatic right lung, and significant improvement in oxygenation and respiratory compliance.

The patient returned to spontaneous breathing with high flow nasal cannula (HFNC) alternating with conventional oxygen therapy; her clinical conditions improved and she underwent to surgical repair of her bronchopleural fistula. After 34 days she was transferred to the pneumology department and discharged after rehabilitation.

Conclusion

ECCO2R is an effective support therapy to be added to mechanical ventilation, limiting its invasiveness and side effects in patients with ARDS. Also, ILV results as a valid treatment option, especially in cases of refractory respiratory failure in which the pathophysiology of the left and right lungs differs markedly.

References

1. Anantham, D., R. Jagadesan, and P.E. Tiew, Clinical review: Independent lung ventilation in critical care. Crit Care, 2005. 9(6): p. 594-600.

2. Camporota, L. and N. Barrett, Current Applications for the Use of Extracorporeal Carbon Dioxide Removal in Critically Ill Patients. Biomed Res Int, 2016. 2016: p. 9781695.

### A191. An Italian survey of noninvasive ventilation: when, where and how is used

#### Rocca E.^1^, De Vita N.^1^, Moretto F.^1^, Gregoretti C.^2^, Carlucci A.^3^, Cortegiani A.^2^, Crimi C.^4^, Mattei A.^5^, Scala R.^6^, Scotti L.^1^, Navalesi P.^7^, Vaschetto R.^1^

##### ^1^Department of Translational Medicine, University of Piemonte Orientale ~ Novara ~ Italia, ^2^Department of Surgical, Oncological and Oral Science (Di.Chir.On.S.) ~ Palermo ~ Italia, ^3^Department of Medicina e Chirurgia, Università Insubria Varese-Como ~ Varese ~ Italia, ^4^Respiratory Medicine Unit, Policlinico “G. Rodolico-San Marco” University Hospital ~ Catania ~ Italia, ^5^Cardio-Thoracic Department, AOU Città della Salute e della Scienza, Molinette Hospital ~ Torino ~ Italia, ^6^Pulmonology and Respiratory Intensive Care Unit, S. Donato Hospital ~ Arezzo ~ Italia, ^7^Department of Medicine, University of Padova ~ Padova ~ Italia

###### **Correspondence:** Rocca E.

Noninvasive ventilation (NIV) is recognized as a valid strategy to avoid intubation in acute hypercapnic (AHCRF) and hypoxemic patients (AHRF). Its use has been increased in the last two decades and nowadays is widely used in selected patients both inside and outside intensive care unit (ICU). NIV failure varies between 8% in hypercapnic and 50% in hypoxemic patients. In acute “de novo” AHRF its undue prolongation may generate patient self-inflicted lung-injury, delaying intubation and worsening patient outcome. Patient comfort is a major factor for NIV success. Choosing the proper interface and duration of NIV are key factors both for patient’s compliance and onset of pressure ulcers, a frequent NIV related side effect. Rates for the incidence of facial pressure ulcers varies from 2 to 31% depending on used masks, length of NIV treatment, ventilator setting, humidification and preventive measures adopted.

A web survey of 18 multiple questions has been sent to 47 physicians, with high expertise in dealing with NIV, working in Italian hospitals. Among the 31 replies, 8 were from community hospitals and 23 from university hospitals. 42% work in ICUs, 29% in intermediate respiratory care units, 23% in medical wards and 7% in emergency departments. Nearly half of them (45%) has more than 20 beds in their unit, 41.9% has 11 to 20 beds and 12.9% less than 10 beds. In 7 unita (23%) more than 200 patients are ventilated per year and in 9 units (29%) between 101 and 200. NIV is mainly applied in AHCRF while its application for AHRF (acute “de novo” weaning or preventing post-extubation respiratory failure) is less frequent.

42% use the high pressure-driven ventilators in double limb non vented configuration (with active expiratory/inspiratory valves) in more than 40% of the patients, while 29% would prefer the turbine-driven in single limb vented configuration (without valves but with an expiratory port). Oro-nasal mask (39%) and full-face mask (54%) are the most used interfaces, mainly because of patient comfort and easy fitting. Regarding the circuit choice, 52% of the physicians would prefer a double-limb circuit with a non-vented mask and 81% of them would use active humidification. 52% of them answered that from 11 to 30% of their patients treated with NIV present a pressure sore lesion or a skin breakdown, despite 77% routinely apply wound care dressing to prevent those related effects. In addition, we investigated the use of a rotational strategy protocol for interfaces during NIV. Surprisingly we found that the practice is not so uncommon with 41.9% physicians using a rotational protocol in their units. However, the timeline to rotate the interface was not homogeneous among the centers.

In Italian hospitals NIV is a frequent treatment choice for many scenarios of AHCRF or AHRF and is performed both in intensive and non-intensive units. The ventilator’s preference varies from the high pressure-driven to the turbine-driven, mainly in double limb non vented configuration. Wound care dressings are mostly applied to prevent pressure sore lesions and skin breakdown, but their incidence is still high.

**Fig. 1 (abstract A191).**
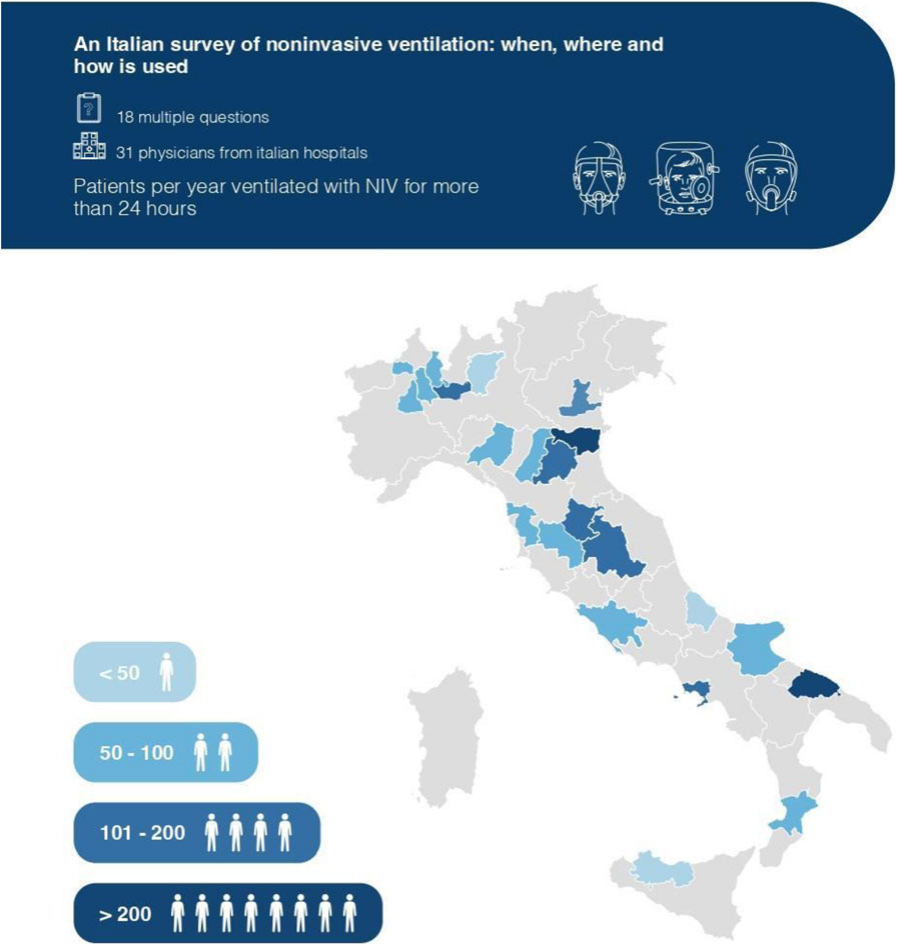
See text for description

### A192. Non- invasive assessment of work of breathing to predict extubation outcome

#### Di Mussi R., Murgolo F., Bellino G., Carpaneto D., Colucci P., Civita A., Dalfino L., Grasso S.

##### Dipartimento delle Emergenze e dei trapianti d'organo (DETO) Università degli studi di Bari "Aldo Moro" ~ Bari ~ Italia

###### **Correspondence:** Di Mussi R.

INTRODUCTION

Identifying candidates for successful extubation is important in clinical practice. The decision is made by considering several parameters simultaneously among which the most important are oxygenation, spontaneous tidal volume and respiratory rate, rapid shallow breathing index (RSBI) in addition to clinical observation. Some of these parameters are surrogates for work of breathing (WOB), because WOB is difficult to measure non-invasively at bedside. The proportional assist ventilation plus (PAV +) technique, however, allows continuous calculation of work of breathing during spontaneous breathing.

OBJECTIVES

To non-invasively measure WOB through the PAV+ technique immediately prior extubation and to compare it with the extubation outcome.

METHODS

We recorded physiological parameters and PAV-derived WOB immediately prior extubation in 109 patients considered ready to be extubated by their attending physicians. WOB was measured through a brief PAV+ trial (30 min) with minimal assistance (i.e. 15%). We defined WOB LOW (< 0.3 Joule/liter), NORMAL (0.3-0.7 Joule/liter) and HIGH (> 0.7 Joule/liter).

RESULTS

Among the studied patients, WOB was high in 24 (22 %), normal in 83 (76.2%), low in 2 (1.8 %). Figure 1 shows the impact of the WOB class on the extubation outcome

CONCLUSIONS

In patients with high WOB before extubation, the rate of extubation success rate was significantly lower (21 %) than in patients with normal WOB (67.5 %). This suggests that non-invasive WOB measurement (as allowed by the PAV+ technique) could usefully support the decision making process to predict the outcome of extubation.

**Fig. 1 (abstract A192).**
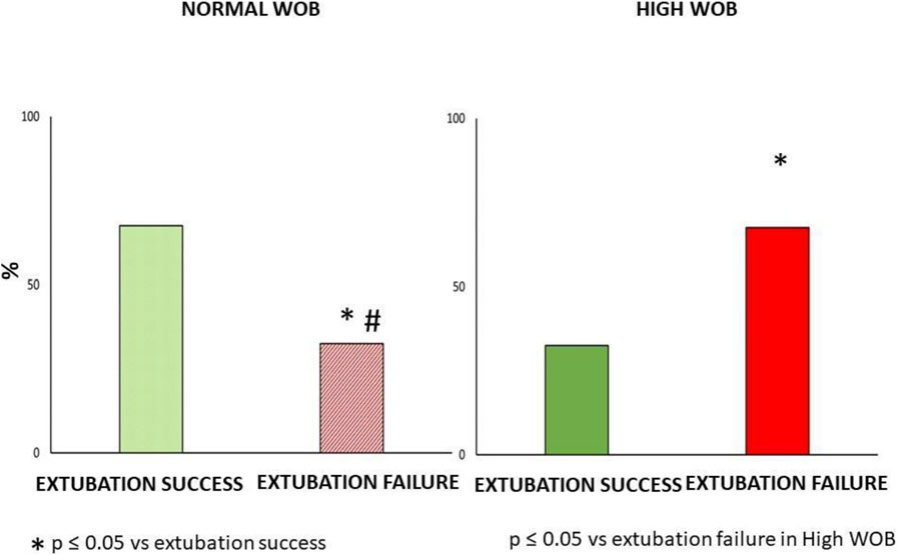
See text for description

### A193. Can heart – lung - respiratory muscles ultrasound evaluation play a role to predict weaning in critically ill patients?

#### Benetto G.^1^, Pasa G.^1^, La Rosa R.^1^, Marangoni E.^1^, Alvisi V.^1^, Cricca V.^1^, Grasso S.^2^, Volta C.A.^1^, Spadaro S.^1^, Fogagnolo A.^1^

##### ^[1]^Department of Anesthesia and Intensive Care Medicine, Azienda Ospedaliera Universitaria di Ferrara, Ferrara, Italy ~ Ferrara ~ Italia, ^[2]^Department of Emergency and Organ Transplantation, Section of Anesthesia and Intensive Care, “Aldo Moro” University of Bari, Bari, Italy ~ Bari ~ Italia

###### **Correspondence:** Benetto G.

Background

Several studies investigated the role of specific clinical predictors of weaning success, but it is determined by a combination of different aspects that can result in success or failure [1,2]. Indeed, we know that unresolved lung disease, cardiac dysfunction, loss of respiratory and core muscle strength can play a role in a failure weaning to mechanical ventilation (MV). We hypothesized that a combined score that include heart, lung, and respiratory muscle ultrasound (US) evaluation could be able to predict the ability of weaning success. Furthermore, we describe weather the days of MV before weaning trial may affect the relevance of each clinical variable evaluated.

Methods

Critically ill patients undergone at least 48 hours of MV and ready to spontaneous breathing trial (SBT) were included in the study. The spontaneous breathing trial was performed in pressure support ventilation with a clinician-set PEEP ≤ 5 cm H2O and FiO2< 40%. In addition to the parameters traditionally used for the evaluation of weaning (RSBI), we performed ultrasound evaluation of diaphragm, lung parenchyma, cardiac function, and the strength of the hand grip.

We evaluated the diaphragmatic parameters (thicknening fraction,TF, and diaphragmatic displacement, DD) and TFmax (TF during forced breathing / TF during tidal respiration) to identify the inspiratory reserve. Weaning success was considered as liberation from MV within 72 hours.

Results

Sixty patients were enrolled; of those, 39/60 were successfully weaned from MV. Weaning success group had lower LUS, higher DD, higher TFmax, lower E/e’, higher TAPSE and higher hand grip strength. The integrated weaning score showed an AUROC of 0.89 to predict weaning success, with 85% specificity and 85% sensitivity for a score >3. (Figure 1) The combined score outperformed each single clinical variable. When considering the days of mechanical ventilation before weaning trial, we observed that LUS score was mostly relevant in patients with MV≤72 hours, E/e’ and TAPSE between 72 hours and 7 days, whereas TFmax and hand grip strength showed the best prediction ability after 7 days of MV. (Figure 1)

Conclusion

A combined score that taking to account the US evaluation of lung, heart and respiratory muscles seems a valuable tool to predict weaning success. Furthermore, each variable can be affected by duration of MV prior to the SBT.

References

1. Bouhemad et al. 2020, Esteban et al. 1994, A comparison of Four Methods of Weaning Patients from Mechanical Ventilation, February 9, 1995 , N Engl JMed1995; 332: 345350.

2. Arnaud W. Thille , Irene Cortés- Puch , Andrès Esteban, Weaning from the ventilator and extubation in ICU, Critical care 2013.

**Fig. 1 (abstract A193).**
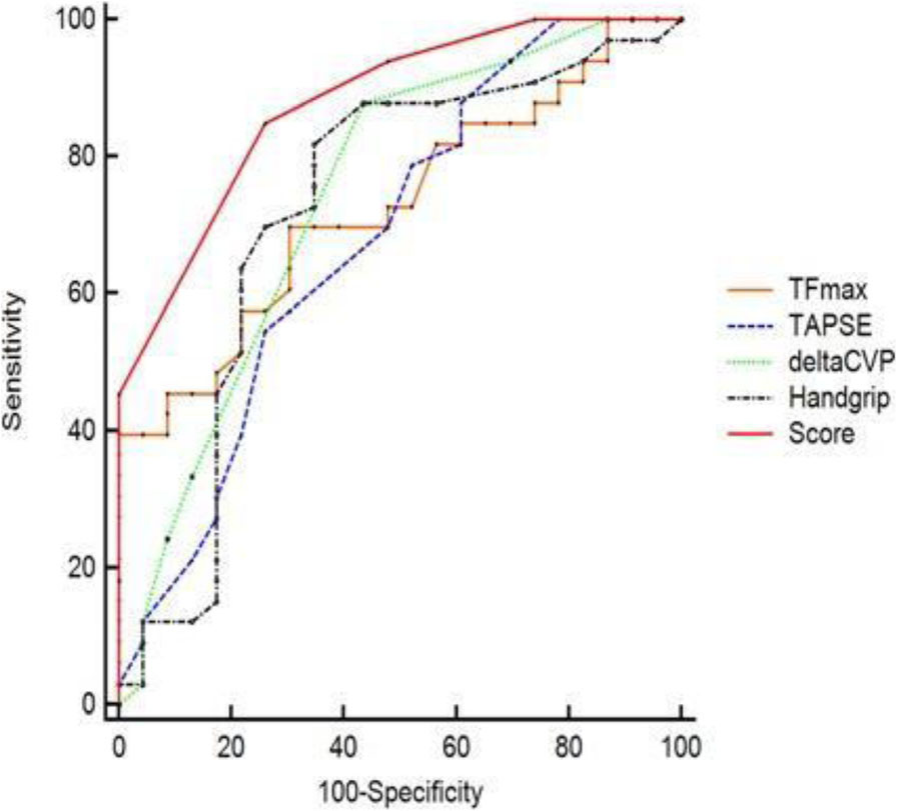
See text for description

### A194. Barotrauma in patients with SARS-COV-2 pneumonia in icu: epidemiology, clinical features and mortality

#### Riforgiato C., Iachi A., Insorsi A., Dameri M., Politi G., Molin A., Rossetti G., Gratarola A., Carosio E., Solaini C., Muschiato U., Rosso G., Polleri E., Pelosi P.P., Patroniti N.A.

##### Department of Surgical Sciences and Integrated Diagnostics (DISC), University of Genoa, Genoa, Italy. Anesthesia and Intensive Care, San Martino Policlinico Hospital-IRCCS for Oncology and Neurosciences, Genoa, Italy. ~ Genova ~ Italia

###### **Correspondence:** Riforgiato C.

Background:

Several multi- and single-center observational studies have shown a high incidence of barotrauma (pneumothorax, pneumomediastinum, and subcutaneous emphysema) in patients with Sars-CoV-2 pneumonia. The present study aims to analysed patients admitted to the Covid-ICU of Policlinico IRCCS San Martino of Genova with Sars-CoV-2 infection who developed barotrauma.

Materials and Methods:

All 139 patients admitted the COVID ICU, between March 2020 and October 2021 with Sars-Cov 2 were included. Diagnostic-clinical features of barotrauma, correlation with comorbidities, and other severity indices were evaluated. In addition, mortality was compared with patients who did not have a barotrauma event.

Results:

Twenty-four (17%) patients showed at least one barotraumatic event. Considering the first event 16 developed a pneumothorax (11.5% of the general population), 9 developed pneumomediastinum (5 of them with concomitant pneumothorax), and 11 developed subcutaneous emphysema (8 simultaneously with pneumothorax). Two patients developed pneumothorax at the second event for a total of 18 patients with pneumothorax and an overall incidence of pneumothorax in the population taken into analysis of 13%.

Mortality of patients with barotrauma was significantly higher (63% vs 32%, p<0.005). The mortality rate of patients who presented with pneumothorax as a complication was significantly higher than for other barotraumatic complications with a total of 13 deaths out of 16 events (81.3% mortality rate, p<0.007) and 14 out of 18 events (78%, p<0.001) when second events were also taken into account.

Barotrauma was suspected or found incidentally in 6 patients (3 died, 50%), by clinical worsening in 10 patients (7 died, 70%), and on objective examination in 8 patients (5 died, 62.5%).

Two patients developed barotrauma during O2 therapy (both survived), 9 during CPAP/NIV (6 of whom died, 66.7%), and 13 during invasive mechanical ventilation (7 of whom died, 54%); there was no statistically significant difference in terms of mortality.

The definitive diagnosis of pneumothorax was made by CT scan in 10 cases (43%), ultrasound in 2 cases (8%), and chest X-ray in 11 cases (47%).

The pneumothorax was managed conservatively in 11 cases (48%) and required placement of a chest drain in 12 cases (52%). Conservative treatment was resolving in 9 of 11 cases (82%), while invasive treatment was resolving in 5 of 12 cases (36%) with p<0.05 in favor of conservative treatment.

Pneumothorax presented while on invasive ventilation in 15 (62%) patients and on noninvasive ventilation (CPAP) in 9 (37%) patients, of which intubation was required in 6 (75%) patients.

Finally, the management of pneumothorax was effective in 14 cases (61%) and required other solutions in 9 cases (39%); all patients in whom management was not resolving died.

Conclusions:

A high incidence of barotrauma was found in both mechanically ventilated and noninvasively ventilated patients. The development of barotrauma, especially when characterized by pneumothorax, is associated with reduced survival. Ventilation at the time of the event and the treatment implemented (conservative or invasive) are not associated with differential mortality.

### A195. Efficacy of non-invasive mechanical ventilation in ordinary hospital wards by the in-hospital medical emergency team

#### Remiddi F.^1^, Gambino I.^1^, La Rosa E.^1^, Cazzato S.^1^, Galgani B.^2^, Mastrocinque E.^2^, Franchi F.^1^, Scolletta S.^1^

##### ^1^Department of Medicine, Surgery and Neuroscience ~ Siena ~ Italy, ^2^Department of Emergency-Urgency and Transplantation ~ Siena ~ Italy

###### **Correspondence:** Remiddi F.

Background

Non-invasive mechanical ventilation (NIMV) is one of the first therapeutic choices of different types of acute respiratory failure, due to its effectiveness in reducing the need for intubation. NIMV can deliver, by means of non-invasive devices (helmet or mask), PEEP alone (CPAP) or PEEP with pressure support. This technique of ventilation is usually performed in intensive care unit (ICU), though it has been gradually extended to ordinary hospital wards (OHW). In our hospital, “Azienda Ospedaliera Universitaria Senese” (AOUS, which consists of about 700 beds), the management of NIMV in ordinary wards is carried out by the in-hospital Medical Emergency Team (MET), consisting of anesthesiologists and nurses.

The aims of the study were to assess, in various subgroups of patients, a) the efficacy of NIMV in terms of improvement of blood gas and clinical parameters, b) the patient's respiratory autonomy upon discontinuation of NIMV in OHW, and c) the mortality.

Materials and methods

This retrospective observational study included 48 patients treated with NIMV in OHW for acute respiratory failure during a six-months period (October 2020 - March 2021). A number of clinical parameters, including blood gas analysis values (e.g., pH, PaO2, PaCO2, PaO2/FiO2) and MEWS (modified early warning score), were collected at three times (time 0, after 24 hours, and at the end of treatment).

Results

The various subgroups are presented in Table 1. Thirtysix (75%) patients achieved weaning from NIMV, 2 (5.5%) of them required admission in ICU for monitoring, but 6 (16.6%) patients died due to their severe underlying comorbidities. Among the patients who were not weaned (n=12) from NIMV, 9 (75%) died and the remaining 3 (25%) were discharged from the hospital but had to continue intermittent NIMV at home. Only 1 patient (2%) required intubation. Changes of pH and MEWS during NIMV were significant over time and between groups (Table 2). Mortality in the “palliative care” group was 50% (4/8), 37% (3/8) in the group with pneumonia, 28% (2/7) in the group with “pump failure” respiratory type, 28% (4/14) in the chronic obstructive pulmonary disease group, and 22% (2/9) in the group with cardiogenic pulmonary edema.

Conclusions

Our study showed that pH and PaCO2 were significantly improved with NIMV. These results confirm the efficacy of this ventilation technique in the treatment of respiratory failure and prevention of intubation, even when applied outside the ICU. The role of MET is pivotal, mostly when it is activated by early and specific clinical criteria. This can allow anesthesiologists either avoiding intubation or transferring the patients to ICU.

**Table 1 (abstract A195).**
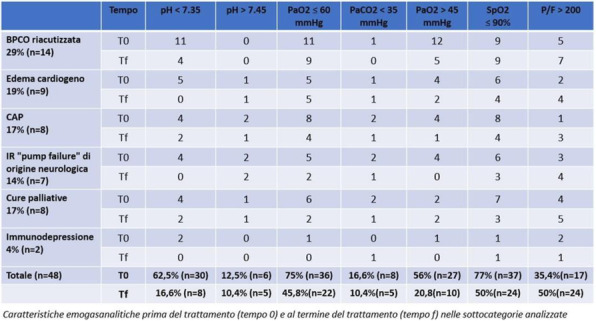
See text for description

**Table 2 (abstract A195).**
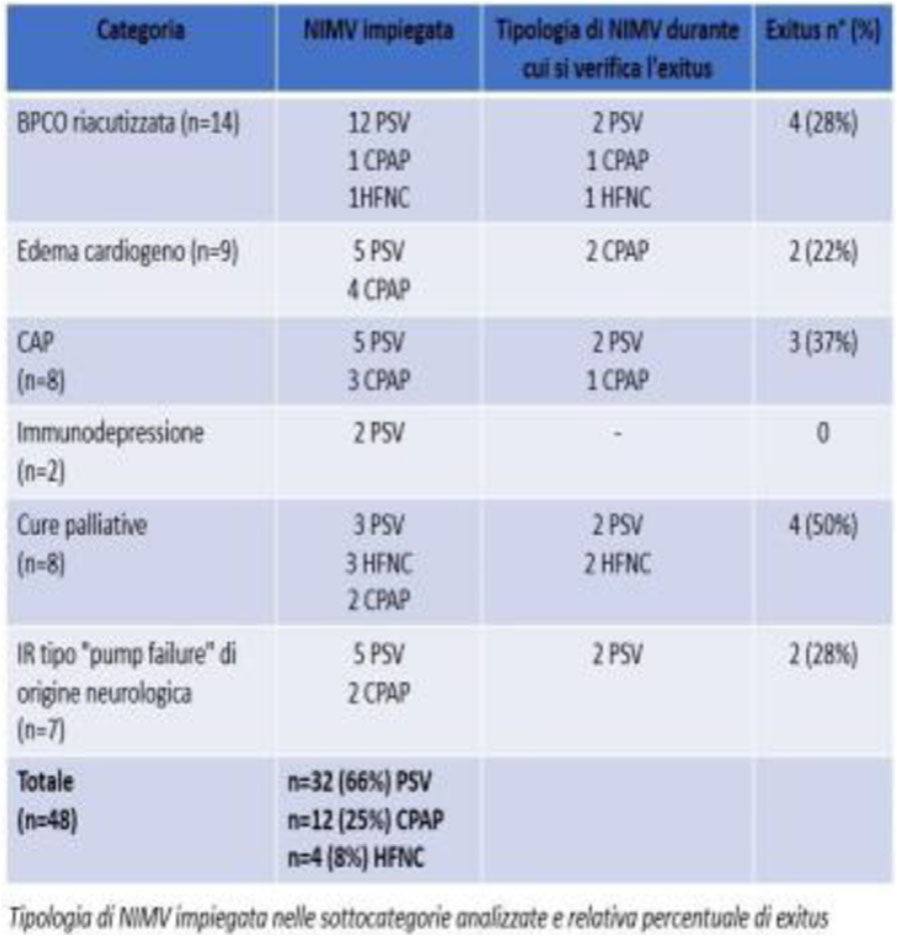
See text for description

**Table 3 (abstract A195).**
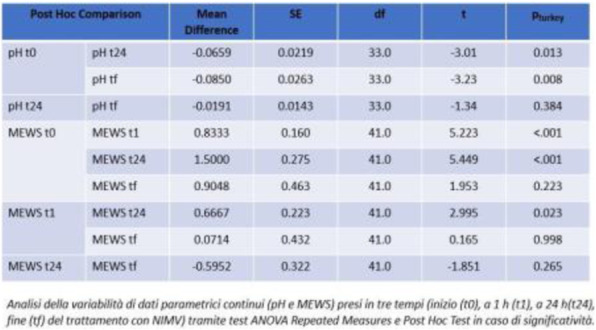
See text for description

**Table 4 (abstract A195).**
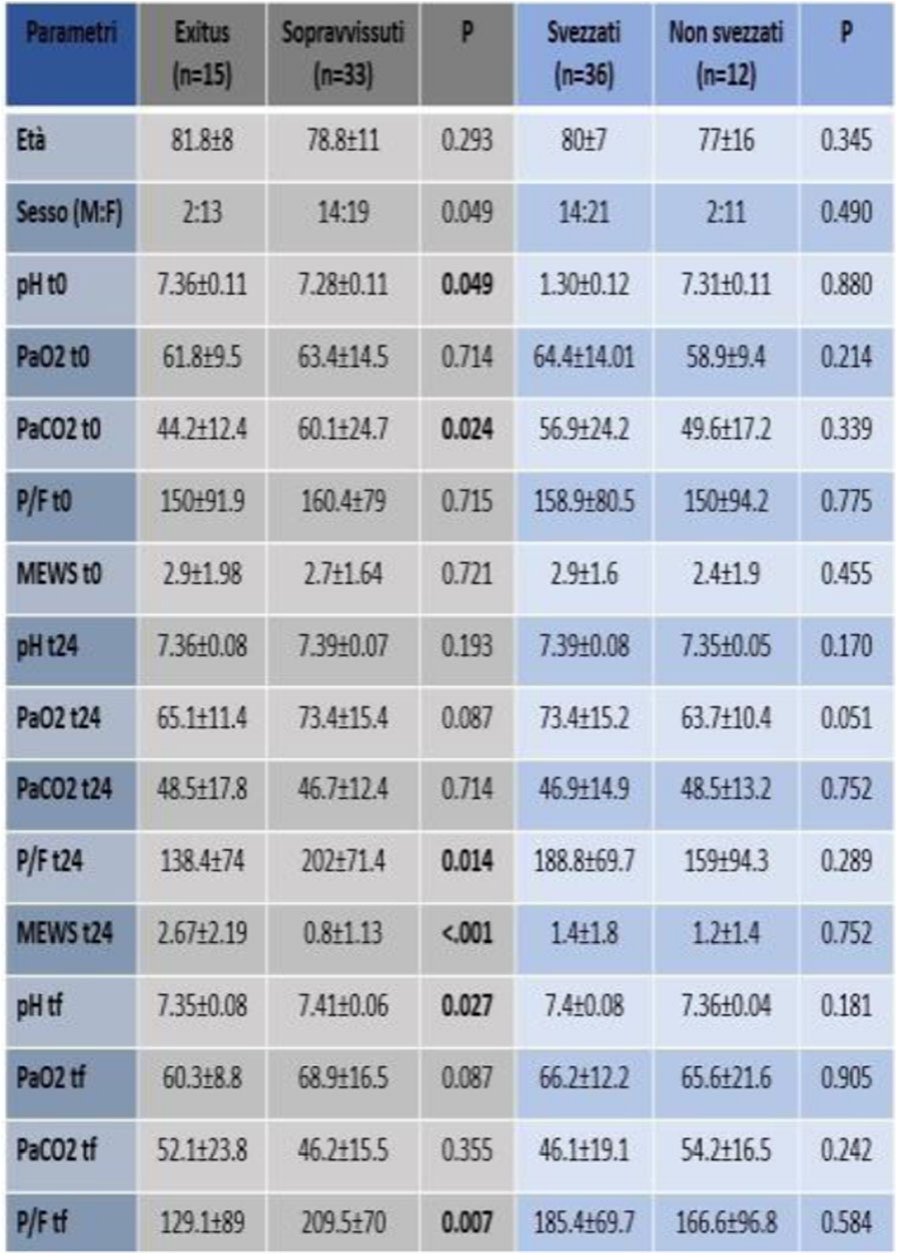
See text for description

### A196. Performance analysis of three heated humidifiers during cpap delivered by helmet and full face mask

#### Fiorillo C.^1^, Vitale D.^2^, Giacchè D.^3^, Ravasio G.^3^, Foti G.^4^, Lucchini A.^4^

##### ^1^AOU Policlinico di Modena Terapia Intensiva Polivalente ~ Modena ~ Italy, ^2^Fondazione IRCCS Ca' Granda Ospedale Maggiore Policlinico Terapia Intensiva Neonatale ~ Milano ~ Italy, ^3^ASST Ospedale Papa Giovanni XXIII TI Neurochirurgia ~ Bergamo ~ Italy, ^4^ASST Monza Ospedale San Gerardo ~ Monza ~ Italy

###### **Correspondence:** Fiorillo C.

Background

Providing a high flow of medical dry gases, above 30L/min, the humidifying and heating capability of the upper airways can be compromised.

During continuous positive airway pressure (CPAP) delivered by helmet or full face mask by using continuous flow systems, active humidification is highly suggested for non invasive mechanical ventilation (NIV), as it provides a better conditioning of fresh medical gases and improves patient comfort and adherence to treatment.

The aim of this study was to evaluate temperature, absolute and relative humidity of respiratory gases given during CPAP within helmet and face mask by using three different humidifiers in their NIV settings.

Materials and methods

We enrolled 16 healthy subjects of different ages, weights and heights, divided in two groups of eight people each: the first group received CPAP delivered by helmet for 10 mins, the second one received CPAP through full face mask, using a circuit provided of a reservoir. Both groups repeated the test with all three humidifiers (Dimar TurboH2O, Hamilton H900, Fisher&Paykel 810). We measured temperature, absolute and relative humidity using an hygrometer inside both interfaces.

Results

Data analysis shows that each humidifier is able to give an absolute humidity of 10 g/m3 H2O, whereas none of them reaches 100% of relative humidity, with a median temperature of 30°C.

During each test with helmet CPAP we did not observe condensation phenomena, otherwise by using mask CPAP there have been a few cases with a small amount of fogging.

Results are shown in table 1 and table 2.

Discussion

Without any humidification, the measured temperature, close to 28°C, is similar to the one generated by the upper airway under spontaneous breathing, but this is not sufficient to reach the appropriate humidity threshold.

The best performance during helmet CPAP is Hamilton's, which deliveres a higher relative humidity, although the set temperature is higher than the ones given by Fisher&Paykel and Dimar.

During mask CPAP, Fisher&Paykel is the one which obtains higher relative humidity values: his set-up, however, is fixed and cannot be modified as in the other two humidifiers.

It would be preferable to have a humidifier that has the chance to manually set the temperature, after careful assessment of patient's clinical condition, and keep it at the lowest level while maintaining an absolute humidity of at least 10 g/m3.

Conclusions

The best humidification in CPAP is achieved when the relative humidity is close to 100% at the lowest possible temperature.

Humidifiers tested with their temperature settings in NIV mode allow to reach at least the 10 g/m3 absolute humidity recommended in the literature to be achieved, even at high flows: however, they struggle to condition medical gases, by flowing them through the humidification chamber, to 100% relative humidity and do not maintain the performance declared in the data sheets.

**Table 1 (abstract A196).** Helmet CPAP

	Temperature C°	Relative Humidity %	Absolute Humidity g/m^3^
Without humidifier	27,50 (1,18)	16,65 (8,37)	4,18 (1,72)
F&P810^TM^ (set 1-26-29°)	29,7 (0,49)	35,99 (5,94)	10,96 (2,21)
Hamilton H900 (set NIV 30°+2)	30,7 (0,45)	60,81 (5,28)	20,31 (1,17)
Dimar TurboH_2_O (set NIV 28°+2)	29,92 (0,52)	43,96 (4,80)	13,26 (1,62)

**Table 2 (abstract A196).** Full face mask CPAP

	Temperature C°	Relative Humidity %	Absolute Humidity g/m^3^
Without humidifier	28,41 (1,32)	3,15 (0,83)	0,76 (0,20)
F&P810^TM^ (set 1-26-29°)	29,31 (1,07)	57,11 (4,20)	16,79 (1,13)
Hamilton H900 (set NIV -30°+2)	30,91 (0,80)	46,75 (3,94)	14,88 (1,06)
Dimar TurboH_2_O (set NIV -28°+2)	28,90 (0,39)	45,10 (5,19)	12,84 (1,35)

## Vie aeree

### A197. Neck echography for routine assessment of patients undergoing nonemergency percutaneous tracheostomy: a case report of aberrant right subclavian artery variant

#### Querio S., Vella P., Zambianchi C., Vignazia G., Donato P., Vaschetto R., Della Corte F.

##### Università del Piemonte Orientale, Dipartimento di Medicina Traslazionale, Anestesia e Terapia Intensiva ~ Novara ~ Italia

###### **Correspondence:** Querio S.

Aberrant right subclavian artery (ARSA) is an anatomical variant of the homonymous artery due to alteration of vessels development during the embryogenesis of thoracic arteries and has an estimated prevalence of 0.5% - 2%. Other anatomical variants can be associated with the presence of ARSA.

Percutaneous dilatational tracheostomy for patients being weaned off mechanical ventilation is a standard intensive care unit (ICU) procedure. Most serious bleeding complications are related to variations of vascular anatomy.

Recently, ultrasound (US) has emerged as a potentially useful tool to assist percutaneous dilatational tracheostomy (PDT) and to reduce procedure-related complications. Principal complicating factors can be the presence of a short neck, deviated trachea, massive goiter, previous neck surgery, obesity, edema and subcutaneous emphysema.

Results of previous studies have demonstrated the benefits of US imaging during the PDT procedure in identifying relevant cervical anatomy and aberrant vasculature in order to avoid immediate vascular complications, but these benefits are limited to the picture site.

We present a clinical case of a 23-year-old male admitted to the ICU after a major trauma. He was sedated and intubated on the scene. A full- body CT scan showed many lesions, among which was seen a fracture of the left femur.

After all the surgical and medical procedures and various sedation windows with poor neurological recovery, we found a cerebral subacute ischemic foci with diffuse embolic patterns in supra and sub-tentorial areas due to a patent foramen ovale without shunt. Consequently, we decided to perform a percutaneous tracheostomy.Explorative ultrasound of the neck performed before the procedure showed the right common carotid artery developed from the left aortic arch and ran to the right and upward in front of the thyroid and the trachea. So we decided for a surgical tracheostomy.

In the operating room once the tracheal axis had been isolated, the variant anatomy of the right internal carotid was displayed. This vessel had cranial from left to right direction, straddling the trachea. Tracheostomy was performed between the second and third tracheal rings and a cuffed cannula with diameter 8 was positioned, without complications.

We finally transferred the patient to another hospital for weaning and recovery.

We suggest that US evaluation and perioperative US guidance in PDT should be used to evaluate cervical anatomy in its entirety, beyond the picture site, in order to guide tracheal puncture in real time. This seems to improve safety and efficacy of PDT and to allow the identification of anatomical variations of the neck.

A recognition of unconventional vascular anatomy, such as the ARSA, and an evaluation by color or spectral Doppler enable a selection of an appropriate alternative puncture site or of an elective open surgical approach.

**Fig. 1 (abstract A197).**
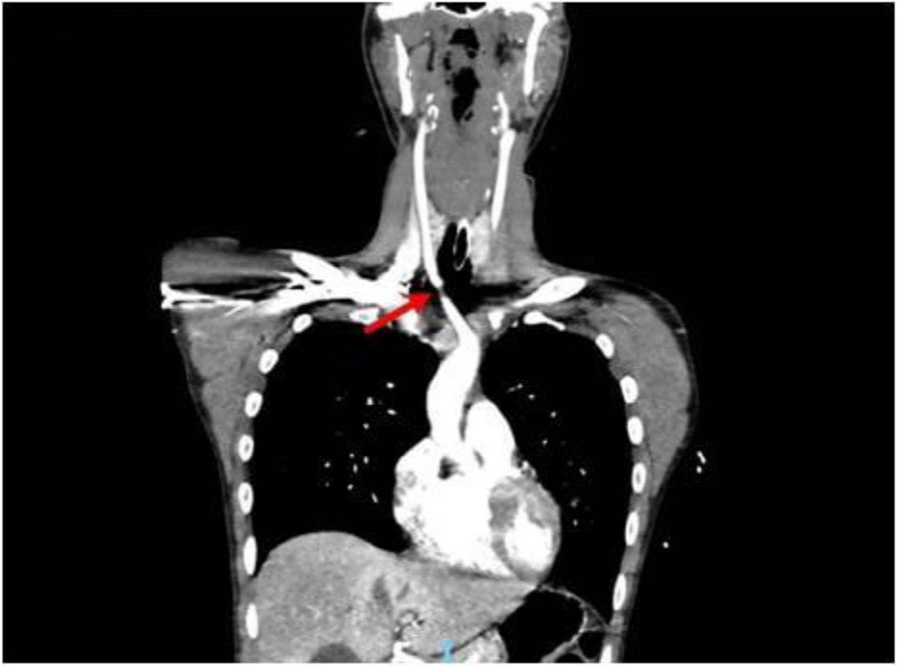
Computed Tomography of the patient shows the subclavian artery crossing the trachea

### A198. Accidental discovery of a massive aortic aneurysm during bronchoscopy

#### Balagna P.^1^, Patrucco F.^2^, Balbo P.E.^2^, De Vita N.^1^, Moretto F.^1^, Donato P.^1^, Della Corte F.^1^, Vaschetto R.^1^

##### ^1^Intensive Care Unit 1, Emergency Department, AOU Maggiore della Carità ~ Novara ~ Italia, ^2^Respiratory Diseases Unit, Medical Department, AOU Maggiore della Carità ~ Novara ~ Italia

###### **Correspondence:** Balagna P.

**Keywords**

Bronchoscopy, aortic aneurysm, pulmonary atelectasis

**Background**

Pulmonary atelectasis is a complete or partial collapse of the lung. It is a common breathing complication especially in invasively ventilated patients. Bronchoscopy can help in diagnosing and treating the problem[1]. The causes of atelectasis are broad and can be due to endobronchial blockages, or external pressure on the lung. Among the latter, thoracic aortic aneurysms may compress lung structure resulting in the collapse of part of the lung as balloon-like bulge closely related to the bronchus [2]. The incidence of thoracic aortic aneurism is estimated to be 5.3 per 100,000 person-year [3] and in most cases is asymptomatic. In severe cases, the compression of the bronchus can lead to a reduction of the ventilated area, thus impaired gas exchange. Although uncommon, we report a case of a massive aortic aneurism causing extrinsic compression of the bronchus.

**Case report**

A 62-year-old women without significant medical history arrived at our emergency department from a peripheral hospital due to hemiplegia and progressive loss of consciousness (GCS 6) requiring intubation and mechanical ventilation. Urgent CT scan showed a massive spontaneous intraparenchymal bleeding for which she underwent an emergency neurosurgical operation after which she has been admitted to our intensive care unit. Chest X-ray has been acquired after central venous catheter placement, which showed partial lung atelectasis around the hilum of the left hemithorax (Fig. 1). Since she was sedated and mechanically ventilated, we decided to perform fiberoptic bronchoscopy with both investigational and curative intent, which revealed some blood in the right bronchus whilst the left side was clear of secretions. However, we noticed an extrinsic compression of the left inferior lobar bronchus resulting in bronchial stenosis (Fig 2). In order to better investigate the cause of the compression, we performed thoracic and abdominal CT angiogram. It showed a massive aneurysm of the descending and abdominal aorta extending up to 25 mm from the aortic bifurcation (Fig 3). The aneurism had a longitudinal extent of approximately 25 cm with a maximum axial diameter of 68 mm x 64 mm measured in the upper abdomen. Moreover, an area of decreased ventilation and minimal pleural effusion has been noticed around the thoracic aneurysm (Fig 4). The aneurysm also caused right kidney atrophy and delayed perfusion to the left kidney, liver and spleen was present. Immediate surgical consultation has been requested.

**Conclusion**

Bedside bronchoscopy is a portable and safe tool that allows easy access to the tracheobronchial tree. Among the differential diagnoses of airway obstruction due to extrinsic compression, it is crucial to comprise thoracic aortic aneurysm as a rare cause of lung atelectasis.

**References**

1. Setu P, Rania F, Rajamurugan S. Bronchoscopy in intubated and non-intubated intensive care unit patients with respiratory failure. Journal of Thoracic Disease. 2021

2. Yap K H, Sulaiman S. Pulmonary atelectasis from compression of the left main bronchus by an aortic aneurysm. Singapore Med J. 2009

3. Ryan G E M, Goncalo S D, Alice L. Incidence and Prevalence of Thoracic Aortic Aneurysms: A Systematic Review and Meta-analysis of Population-Based Studies. Semin Thorac Cardiovasc Surg. 2022

*Informed consent to publish had been obtained.*

**Fig. 1 (abstract A198).**
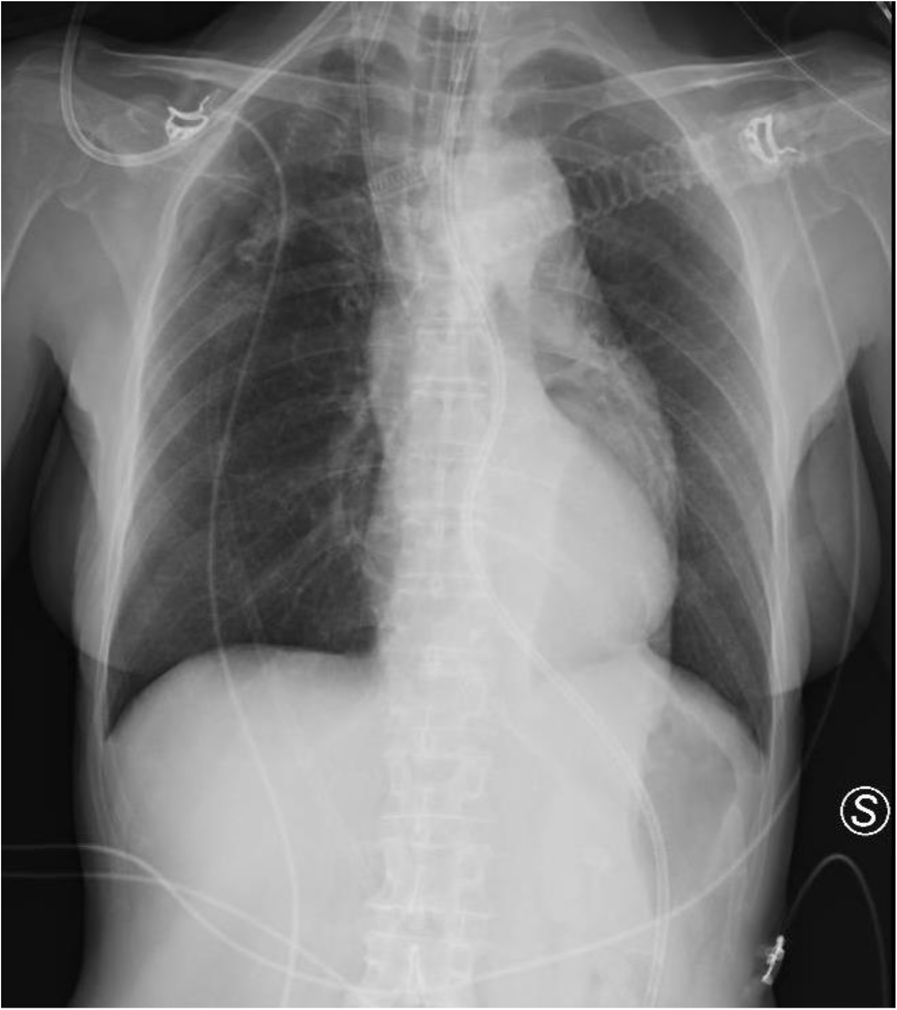
Anteroposterior chest radiograph taken after CVC placement shows partial lung atelectasis around the hilum of the left hemithorax

**Fig. 2 (abstract A198).**
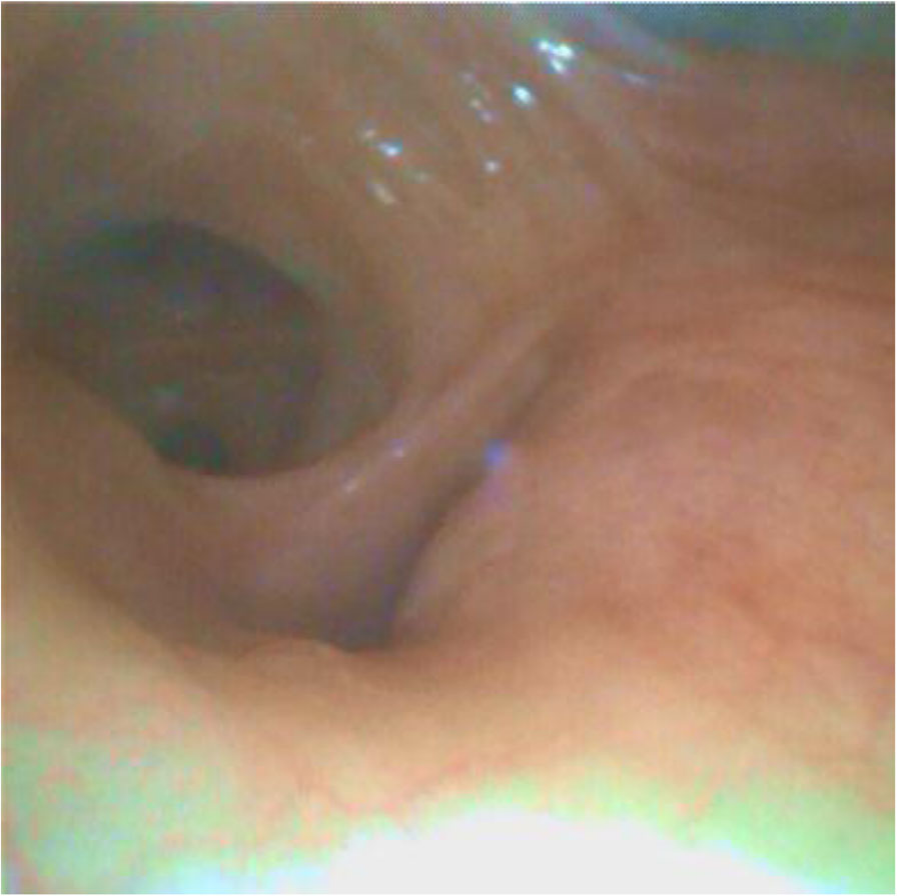
Endobronchial image taken during bronchoscopy shows bronchial stenosis

**Fig. 3 (abstract A198).**
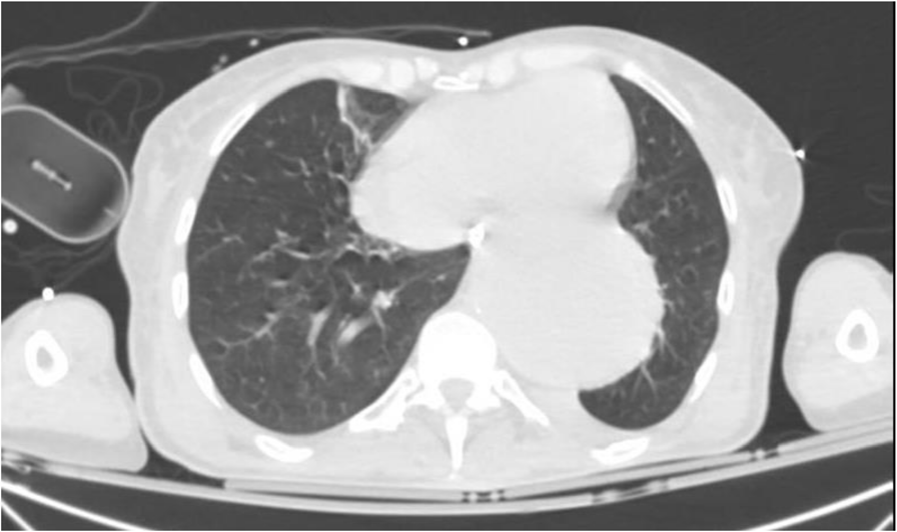
Axial CT image shows a massive descending thoracic aneurysm

**Fig. 4 (abstract A198).**
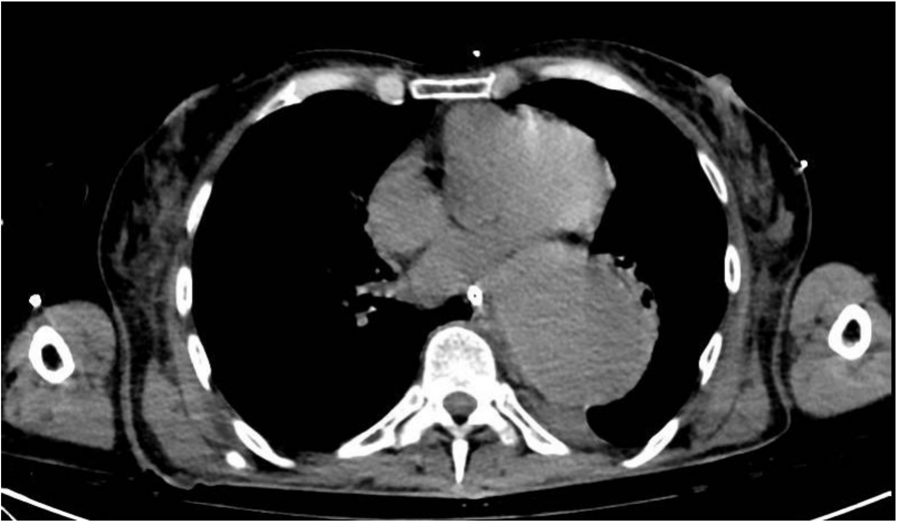
Axial CT image shows the compression of the bronchus by the aneurysm

### A199. Evaluation of long-term complications of tracheostomy in patients discharged from ICU: a monocentric, observational, retrospective study (Valtramore)

#### Bray L., Tozzi P., Marraffa E., Santini F., Padovani F., Alessandri F., Pugliese F.

##### Dipartimento di chirurgia generale e specialistica “Paride Stefanini”, Sapienza Università di Roma, Policlinico Umberto I ~ Roma ~ Italia

###### **Correspondence:** Tozzi P.

Introduction: Long-term post-tracheostomy complications can have a significant clinical impact in patients who have been admitted to an intensive care unit. The literature is not particularly thriving on this issue due to the high mortality rate and the difficulty in finding information after discharge. The present study aims to evaluate the mortality and complications related to tracheostomy in patients who have been hospitalized in intensive care at the Policlinico Umberto I in Rome 6 months after discharge from the intensive care unit.

Methods: The medical records of all patients undergoing tracheostomy in 3 intensive care units of the Policlinico Umberto I in the period between 1 January 2019 and 30 June 2020 were analyzed retrospectively. The technique used (percutaneous or surgical) was evaluated. Data on mortality and late complications related to tracheostomy (stoma infection, stenosis, tracheomalacia and / or other) were obtained by telephone at 6 months after discharge.

Results: In 115 of the 157 patients analyzed, 6 months had elapsed after discharge but 39 (33.9%) of these had died during admission to intensive care. Of the 76 potentially eligible patients, 21 were lost to follow-up, 10 denied consent and 3 were unable to provide the requested information. Of the 42 patients evaluable for long-term follow-up, 16 (37.5%) died after discharge. In the other 26 patients 14 (53.8%) denied having had problems related to tracheostomy, in the remaining 12 cases (46.2%) 5 patients complained of unsightly scarring, 4 stoma infection, 2 dysphagia, 1 hoarseness, 1 difficulty in scarring and there was a single case of tracheal stenosis requiring intervention and placement of endotracheal prosthesis.

Conclusions: The 6-month mortality rate was 65.48%. The incidence of late complications is low but it seems necessary to increase the number of patients to have more certain results. In line with the data in the literature, the most frequent long-term complication was stoma infection (15.4%). Although complications were observed more frequently in patients undergoing surgical tracheostomy, a statistically significant difference was not achieved between the two techniques.

### A200. Direct and video-laryngoscopy vs combined laryngo-bronchoscopy approach for orotracheal intubation in a simulated normal airway scenario

#### Sanfilippo G.^1^, La Via L.^2^, Messina S.^1^, Merola F.^3^, Tornitore F.^1^, Lanzafame B.^4^, Farina M.^2^, Cocimano S.^2^, Astuto M.^2^, Sanfilippo F.^2^

##### ^1^Department of Anesthesia and Intensive Care, University "Magna Graecia" ~ Catanzaro ~ Italia, ^2^Department of Anesthesia and Intensive Care, AOU "Policlinico - San Marco" ~ Catania ~ Italia, ^3^Department of Anesthesia and Intensive Care, University of Catania ~ Catania ~ Italia, ^4^ASP Siracusa ~ Siracusa ~ Italia

###### **Correspondence:** Sanfilippo G.

Background and Goal: Endotracheal intubation (ETI) plays a crucial role during anaesthesia and emergency medicine. The fibreoptic-bronchoscope intubation (CLBI) has been proposed to improve airway management and has shown promising results in simulated airway scenarios. The aim of this study was to evaluate the results of the CLBI approach as compared with direct laryngoscopy (DL) or video-laringoscopy (VL, with McGrath and Glidescope) in a simulated normal airway management scenario. Methods: We performed a survey and a prospective simulation study involving 89 residents of Anesthesia with variable experience in airway management. Residents performed ETI on a Laerdal™ manikin using DL with Macintosh blade, McGrath, Glidescope and CLBI after a video presentation on the devices. Residents were allowed maximum 3 attempts per device and no more than 60 seconds for each attempt. They were also asked to report their experience with all the devices used. The main outcomes were success rate (SR) and time to intubation (TTI) at first attempt. Results and Discussion: As shown in Table 1, DL had significantly higher SR (96.6%) at first attempt than CLBI (51.7%) and VLs (Glidescope, 85.4%; McGrath 67.4%). Intubation failure after three attempt was significantly higher for CLBI (19.1%) as compared to VLs (10.1% with McGrath and 1.1% with Glidescope) or DL (0%). In residents succeeding the intubation, CLBI had significantly higher TTI (41.4±9.7 sec) than other devices (Glidescope 26.6±11.5 sec, McGrath 25.3±12.8 sec and DL 18.2±9.9 sec). Among VLs, Glidescope was superior to McGrath in terms of SR at first attempt (p=0.008), while TTI was not different (p=0.51). Conclusion(s): Under simulated conditions, in residents with variable experience CLBI approach had significantly worse performances as compared to DL with Macintosh blade and to VLSs. Our results suggests that more experience and training is needed before implementing this intubation technique. Analysis of subgroups according to their experience is ongoing.

Reference: Sanfilippo F, Sgalambro F. Use of a Combined Laryngo-Bronchoscopy Approach in Difficult Airways Management: A Pilot Simulation Study. 2019;47:464-70.

**Table 1 (abstract A200).** Summary of results

		Direct Laryngoscope		Glidescope		McGrath		CLBI	
**SR at 1**^**st**^ **attempt**	Overall	92/96 (96%)		81/96 (84%)		64/96 (67%)		48/96 (50%)	
	Junior Senior	56/60 (93%) 36/36 (100%)	0.29	49/60 (82%) 32/36 (89%)	0.40	39/60 (65%) 25/36 (69%)	0.82	26/60 (43%) 22/36 (61%)	0.14
**SR at 2**^**nd**^ **attempt**	Overall	94/96 (98%)		90/96 (94%)		79/96 (82%)		67/96 (70%)	
	Junior Senior	58/60 (97%) 36/36 (100%)	0.53	55/60 (92%) 35/36 (97%)	0.41	50/60 (83%) 29/36 (81%)	0.79	38/60 (63%) 29/36 (81%)	0.11
**SR at 3**^**rd**^ **attempt**	Overall	95/96 (99%)		94/96 (98%)		85/96 (89%)		78/96 (81%)	
	Junior Senior	59/60 (98%) 36/36 (100%)	1.00	58/60 (97%) 36/36 (100%)	0.53	54/60 (90%) 31/36 (86%)	0.74	45/60 (75%) 33/36 (92%)	0.06
**cTTI**	Overall	15.3 [9.4]		25.3 [22.6]		29.8 [67.1]		72.4 [111.9]	
	Junior Senior	16.3 [10.9] 14.6 [7.3]	0.13	28.0 [33.9] 22.8 [14.5]	0.16	31.9 [65.3] 27.4 [61.7]	0.66	98.4 [127.0] 51.5 [72.4]	**0.03**
**uTTI**	Overall	15.1 [9.0]		24.4 [14.8]		22.4 [17.0]		40.5 [15.4]	
	Junior Senior	15.5 [10.1] 14.6 [7.3]	0.22	24.9 [16.4] 22.3 [10.7]	0.34	23.6 [14.6] 19.2 [19.9]	0.90	41.1 [12.5] 39.3 [16.6]	0.76

### A201. Percutaneous tracheostomy with the ciaglia blue rhino technique with the aid of the disposable broncoscope in intensive care: preliminary case studies

#### Sucre M.J., Ciceraro S., Voto G., Generali A., Petrone R., Aperto A., Ambrosio S., Balia M., Ragone R.

##### Anesthesia and Intensive Care Department. San Leonardo Hospital ~ Castellammare di Stabia (Napoli) ~ Italy

###### **Correspondence:** Ragone R.

The purpose of this study was to report our experience over a period of 10 months, from July 2021 to April 2022 with the Ciaglia Blue Rhino method and the aid of the Ambu® aScope™ 4 Broncho Sampler disposable endoscope.

METHOD: The preliminary analysis included 27 adult patients undergoing bronchoscopic percutaneous tracheostomy connected to the high-quality full-HD Ambu® aView™2 Advance monitor. Our tracheostomy procedure involves the ultrasound evaluation of the trachea and as a first step the replacement of the tracheal tube with a second generation I-Gel® supraglottic device, for the management of the airways. The point through which to introduce the needle is searched for by means of the Sucre maneuver: the thumb is pressed on the chosen point (gap between the 2nd and 3rd tracheal ring) and at the same time it is observed from inside the trachea, by means of videoscopic vision, the pressure exerted by the finger and therefore the accuracy of the reference.

The main variables analyzed were the mean time of orotracheal intubation, the duration of the procedure, the level of difficulty (easy, moderately difficult, difficult) and the complications during the execution related to the use of the single-use bronchoscope compared to the traditional use of the videobronchoscope multipurpose.

RESULTS: The mean time to orotracheal intubation was 9.4 days. The average length of the procedure was 14 minutes and 12 seconds. The procedure was evaluated as easy in 50% of cases, moderately difficult in 20% of cases, difficult in 30% of cases. No complications were recorded. Passing the disposable bronchoscope through the I-Gel mask and glottis was made easier by allowing a clear view of the upper trachea as well as transillumination of the anterior wall of the trachea prior to puncture. The display on the anti-reflective touch screen was immediate after switching on, allowing accurate identification of intraluminal structures. The 180 ° position changes and the use with the battery facilitated the positions more easily in reference to the operators than with the traditional video column. Thanks to the disposable bronchoscope, there was always an excellent visualization of the operative field during all phases of the procedure. Tracheostomy was successful in all patients

CONCLUSIONS: Our preliminary experience suggests that percutaneous tracheostomy with the Ciaglia Blue Rhino technique using disposable bronchoscopy is useful, quick and easy to learn. The reliable transfer of images to the screen, with the possibility of 180-degree rotation and with battery life of up to 3 hours, allows for better positioning, depending on the clinical situation and available space. The ability to sample secretions in a closed loop allows for safe handling. This method improves the visualization of the trachea and larynx and prevents the difficulties associated with the use of the multipurpose videobronchoscope such as the long preparation time and accidental injuries of the instrument, guaranteeing absolute prevention of infections.

- Sucre MJ et all. Critical Care 2010, 14(Suppl 1): P226

- De Nicola A et all. Critical Care 2013, 17(Suppl 2):P169

- Tracheotomy. https://it.wikipedia.org/wiki/Tracheotomia#Procedura 2015

